# TOR complex 1 negatively regulates NDR kinase Cbk1 to control cell separation in budding yeast

**DOI:** 10.1371/journal.pbio.3002263

**Published:** 2023-08-30

**Authors:** Magdalena Foltman, Iván Mendez, Joan J. Bech-Serra, Carolina de la Torre, Jennifer L. Brace, Eric L. Weiss, María Lucas, Ethel Queralt, Alberto Sanchez-Diaz

**Affiliations:** 1 Mechanisms and Regulation of Cell Division Research Unit, Instituto de Biomedicina y Biotecnología de Cantabria (IBBTEC), Universidad de Cantabria-CSIC, Santander, Spain; 2 Departamento de Biología Molecular, Facultad de Medicina, Universidad de Cantabria, Santander, Spain; 3 Structural Biology of Macromolecular Complexes Research Unit, Instituto de Biomedicina y Biotecnología de Cantabria (IBBTEC), Universidad de Cantabria-CSIC, Santander, Spain; 4 Josep Carreras Leukaemia Research Institute, IJC Building, Campus ICO-Germans Trias i Pujol, Barcelona, Spain; 5 Department of Biochemistry, Molecular Biology, and Cell Biology, Northwestern University, Evanston, Illinois, United States of America; 6 Instituto de Biomedicina de Valencia (IBV-CSIC), Valencia, Spain; The Institute of Cancer Research, UNITED KINGDOM

## Abstract

The target of rapamycin (TOR) signalling pathway plays a key role in the coordination between cellular growth and the cell cycle machinery in eukaryotes. The underlying molecular mechanisms by which TOR might regulate events after anaphase remain unknown. We show for the first time that one of the 2 TOR complexes in budding yeast, TORC1, blocks the separation of cells following cytokinesis by phosphorylation of a member of the NDR (nuclear Dbf2-related) protein-kinase family, the protein Cbk1. We observe that TORC1 alters the phosphorylation pattern of Cbk1 and we identify a residue within Cbk1 activation loop, T574, for which a phosphomimetic substitution makes Cbk1 catalytically inactive and, indeed, reproduces TORC1 control over cell separation. In addition, we identify the exocyst component Sec3 as a key substrate of Cbk1, since Sec3 activates the SNARE complex to promote membrane fusion. TORC1 activity ultimately compromises the interaction between Sec3 and a t-SNARE component. Our data indicate that TORC1 negatively regulates cell separation in budding yeast by participating in Cbk1 phosphorylation, which in turn controls the fusion of secretory vesicles transporting hydrolase at the site of division.

## Abstract 2

This study reveals how TOR complex 1 (TORC1) regulation of Cbk1 allows eukaryotic cells to coordinate cell division and cell growth, thereby guaranteeing that mother and daughter cells separate only at the end of the cell cycle.

## Introduction

Cells need to control the timing and sequence of different steps during the cell cycle [1–3]. In particular, cell cycle regulatory machinery ensures that events of cytokinesis and cell separation occur in precise order only after cells have segregated sister chromatids towards the opposite poles of the cell [4]. In budding yeast, a recently described checkpoint named “enforcement of cytokinesis order” (ECO) ensures that late processes of cell separation do not occur when early cytokinetic processes are delayed or defective [5].

Cell division is linked to growth and metabolism, although the molecular details are currently incompletely understood. The highly conserved target of rapamycin (TOR) signalling pathway has well-documented roles in regulation of cell growth, nucleotide biosynthesis, lipogenesis, glycolysis, autophagy, and aspects of cell cycle progression [6–8], and thus may provide a key link between cell growth and the cell cycle machinery [9–12]. Indeed, TOR signalling induces stabilisation of the mRNA of a mitotic cyclin, which binds to cyclin-dependent kinase to promote mitosis [13]. However, a specific role for TORC1 in the regulation of cytokinesis and cell separation remains unknown.

Successful mitotic separation of chromosomes by elongation of the anaphase spindle allows activation of the budding yeast mitotic exit network (MEN). This signalling cascade promotes transition from M phase to G1 and initiates cytokinesis [14,15], in which actomyosin ring contraction and plasma membrane constriction are intimately linked and coordinated with the formation a special layer of chitin-rich extracellular matrix named septum [16,17]. After these processes are complete, mother and daughter cells remain linked to each other by the septum, which is immediately severed by daughter cell specific secretion of hydrolytic enzymes, allowing the 2 cells to dissociate [4]. In budding yeast, a signalling pathway called the “regulation of Ace2 and morphogenesis” (RAM) network promotes this final cell separation at the end of the cell cycle. Interestingly, the MEN and RAM pathways are functionally distinct “Hippo” signalling system that has similar components and organisation [4]. These networks, known as Mst/hippo or NDR/LATS signalling after mammalian and *Drosophila* kinases, participate in the control of cell growth, proliferation, and morphogenesis in an immense range of eukaryotes [4]. The budding yeast nuclear Dbf2-related (NDR) kinase Cbk1 is the central regulatory component of the RAM network; previous studies have not identified a role of Cbk1 in the coordination of growth and cell cycle.

Here, we describe analysis of post-anaphase functions of the rapamycin-sensitive TORC1 kinase complex, one of the 2 TOR complexes in *Saccharomyces cerevisiae*. We found a previously unappreciated mechanism in which the MEN kinase Cdc15 and TORC1 play opposing roles in control of cell separation. TORC1 regulates and participates in the phosphorylation of the RAM network kinase Cbk1 to ultimately control cell separation. By mass spectrometry, we found that TORC1 modifies the phosphorylation pattern of Cbk1. In addition, we identified a specific threonine in Cbk1 activation loop that its phosphomimetic version is catalytically inactive and reproduces TORC1 control over cell separation. We identified that TORC1 regulation of Cbk1 may achieve control of cell separation by modulating membrane fusion of secretory vesicles delivering hydrolytic enzymes to the site of division. Finally, we determined that Cbk1 binds and contributes to the phosphorylation of the exocyst component Sec3, which would explain defects in cell separation as Sec3 activates the SNARE complex to promote vesicle-plasma membrane fusion.

## Results

### Hypophosphorylated forms of a TORC1 substrate accumulate during mitosis

**Fig 1:**
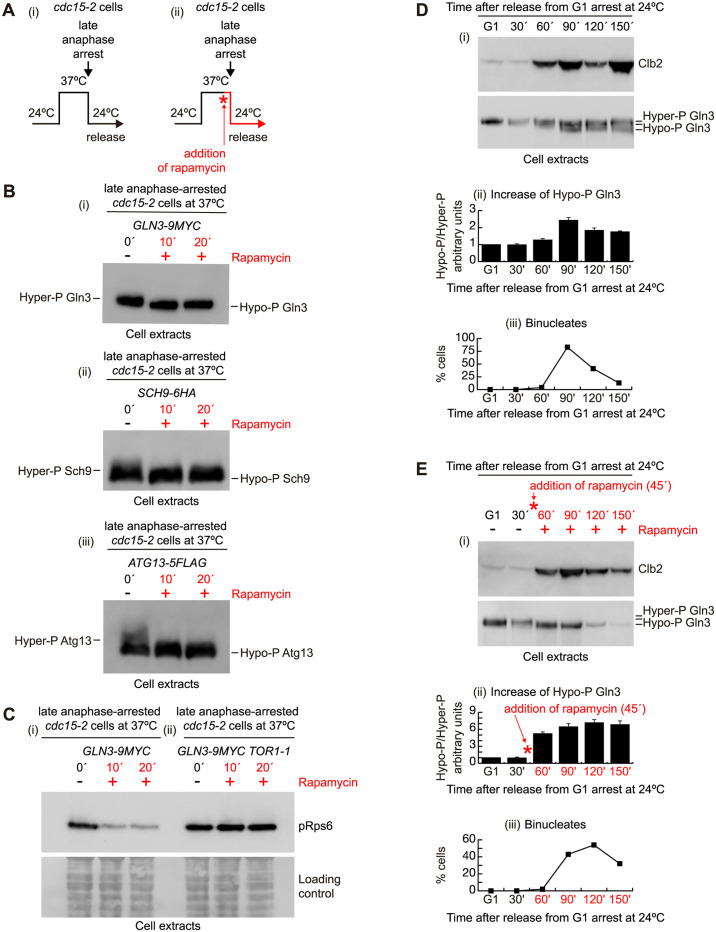
Hypophosphorylated forms of a TORC1 substrate accumulate during mitosis. (A) Schematic representation of experimental set-up in which *cdc15-2* cells were arrested at 37 °C in late anaphase, and 20 min before the end of the block, rapamycin was added to half of the culture (ii). Subsequently, cells were released in the absence (i) or the presence of rapamycin (ii). (B) *GLN3-9MYC cdc15-2* cells (YMF3178) (i) were grown as in the experimental set-up shown in A until the end of the late anaphase arrest. Rapamycin was added and protein extracts were prepared and analysed by immunoblotting at the indicated times while cells were still arrested in late anaphase. In addition, *SCH9-6HA cdc15-2* cells (YMF3782) (ii) and *ATG13-5FLAG cdc15-2* cells (YMF4500) (iii) were grown and protein levels were analysed in the same way as for (i). (C) *GLN3-9MYC cdc15-2* cells (YMF3178) (i) and *TOR1-1 GLN3-9MYC cdc15-2* cells (YMF3260) (ii) were grown and analysed as in B using a phospho specific antibody that recognises phosphorylation on residues Ser235 and Ser236 of the TORC1 target Rps6. (D) *GLN3-9MYC* (YMF3154) and *CLB2-9MYC* cells (YMF3262) were grown at 24 °C in YPD medium and synchronised in G1 phase with mating pheromone before cells were released from the G1 block to allow progression through the cell cycle at 24 °C. Samples were taken at the specified times to prepare protein extracts before the detection of Clb2 and Gln3 by immunoblotting (i). Ratio of hypophosphorylated Gln3 and hyperphosphorylated Gln3 at each time point is depicted (Hypo-P Gln3/Hyper-P Gln3) (ii). Mean of 3 biological replicates and SEM are shown in the graph. In addition, cells were taken to count the proportion of binucleates as cells were progressing through the cell cycle at 24 °C (ii). (E) *GLN3-9MYC* (YMF3154) and *CLB2-9MYC* cells (YMF3262) were grown as in D, but rapamycin was added 45 min after G1 release. Analysis was carried out in the same way as in D. Underlying data for all the graphs can be found in S1 Data file. Raw data for blots can be found in Supporting information (S1 Raw Images). TOR, target of rapamycin.

Rapamycin treatment specifically blocks the activity of TORC1 and promotes an arrest in the G1 phase of the cell cycle [18–20] and may also delay or inhibit other cell cycle phases [13,21,22]. We sought to determine if TORC1 has specific roles in cell division following anaphase. An asynchronous culture of cells harbouring a temperature sensitive mutation in the *CDC15* gene, *cdc15-2*, was grown at 24 °C. *CDC15* encodes for a kinase, member of the MEN pathway, which plays an essential role allowing cells to exit mitosis [4,23]. We synchronised *cdc15-2* cells in late anaphase by raising the temperature at 37 °C for 2.5 h. More than 90% of cells accumulated as large-budded cells. The culture was split evenly in 2 (Fig 1A(i) and 1A(ii)), and rapamycin was added to one half of the culture to inactivate TORC1 for another 20 min while cells were maintained at 37 °C before release at the permissive temperature of 24 °C (Fig 1A(ii)). Cells readily re-enter the M to G1 transition upon shift to permissive temperature. We confirmed that the TORC1 target protein Gln3, a transcription activator responsible for expression of Nitrogen Catabolite Repression-sensitive genes, was rapidly dephosphorylated after rapamycin addition (Fig 1B(i)) as previously described [24]. In addition, we found that inactivation of TORC1 activity promoted rapid dephosphorylation of other known TORC1 substrates too, like Sch9 and Atg13 (Fig 1B(ii) and 1(iii)), as previously reported [25]. Furthermore, phosphorylation of the ribosomal protein S6 (Rps6) has been shown to monitor activation of TORC1 in budding yeast [26]. Using a highly specific antibody [26], we showed that Rps6 is rapidly dephosphorylated in anaphase-arrested *cdc15-2* cells after rapamycin addition (Fig 1C). In budding yeast, TORC1 contains one of 2 kinases, Tor1 or Tor2, responsible for the phosphorylation of its targets [27]. In addition, subunits Lst8 and Kog1 form part of the complex [27]. To confirm that detection of Rps6 phosphorylation was dependent on the activity of kinase Tor1, we used the mutant *TOR1-1*, which is resistant to rapamycin [28]. We arrested *TOR1-1 cdc15-2* cells in late anaphase by shifting cells at 37 °C, and we found that Rps6 phosphorylation was maintained despite the presence of rapamycin (Fig 1C(ii)). Therefore, these experimental data showed that TORC1 was specifically inactivated in *cdc15-2* cells that were blocked in late anaphase after the addition of rapamycin as depicted in Fig 1A(ii). Finally, to determine whether TORC1 inactivation might occur in a normal cell cycle, we released *GLN3-9MYC* cells from a G1 block and study the mitotic cyclin Clb2, which accumulated as cells approaching mitosis and is inactivated in late anaphase (Fig 1D(i) 120′). After finishing the first cycle, cells lost synchrony and Clb2 accumulated quickly (Fig 1D(i) 150′). Using 3 biological replicates, we found that dephosphorylation of Gln3 increased when the number of binucleated cells peaked after G1 release (Fig 1D, 90′), which would indicate that TORC1 is inactivated in anaphase, before Clb2 inactivation was started (Fig 1D(i), 120). As control, we showed that hypophosphorylated form of Gln3 accumulated when rapamycin was added 45 min after G1 release (Fig 1E).

In a normal cell cycle, down-regulation of kinase activity associated to Clb2/CDK complexes depends on Clb2 degradation, as seen in Fig 1D, and Sic1 inhibition. To determine whether inactivation of CDK activity was able to induce a change in Gln3 mobility, we expressed a stable form of Sic1 (Sic1ΔNT) while cells were blocked in G2-M by the presence of nocodazole in the culture (S1A Fig). We found a slight change in Gln3 mobility, which would indicate that inactivation of CDK was unable to drive Gln3 dephosphorylation (S1A(i) Fig). Whereas addition of rapamycin, while cells were arrested in G2-M and CDK was down-regulated, rapidly promoted Gln3 dephosphorylation, which suggest that TORC1 was mainly responsible for Gln3 dephosphorylation (S1A(ii) Fig). Taken together, these experiments suggest that TORC1 might be inactivated during mitosis as it has been described in human cells [29].

### Inactivation of TORC1 rescues specifically division defect of *cdc15-2* cells

**Fig 2:**
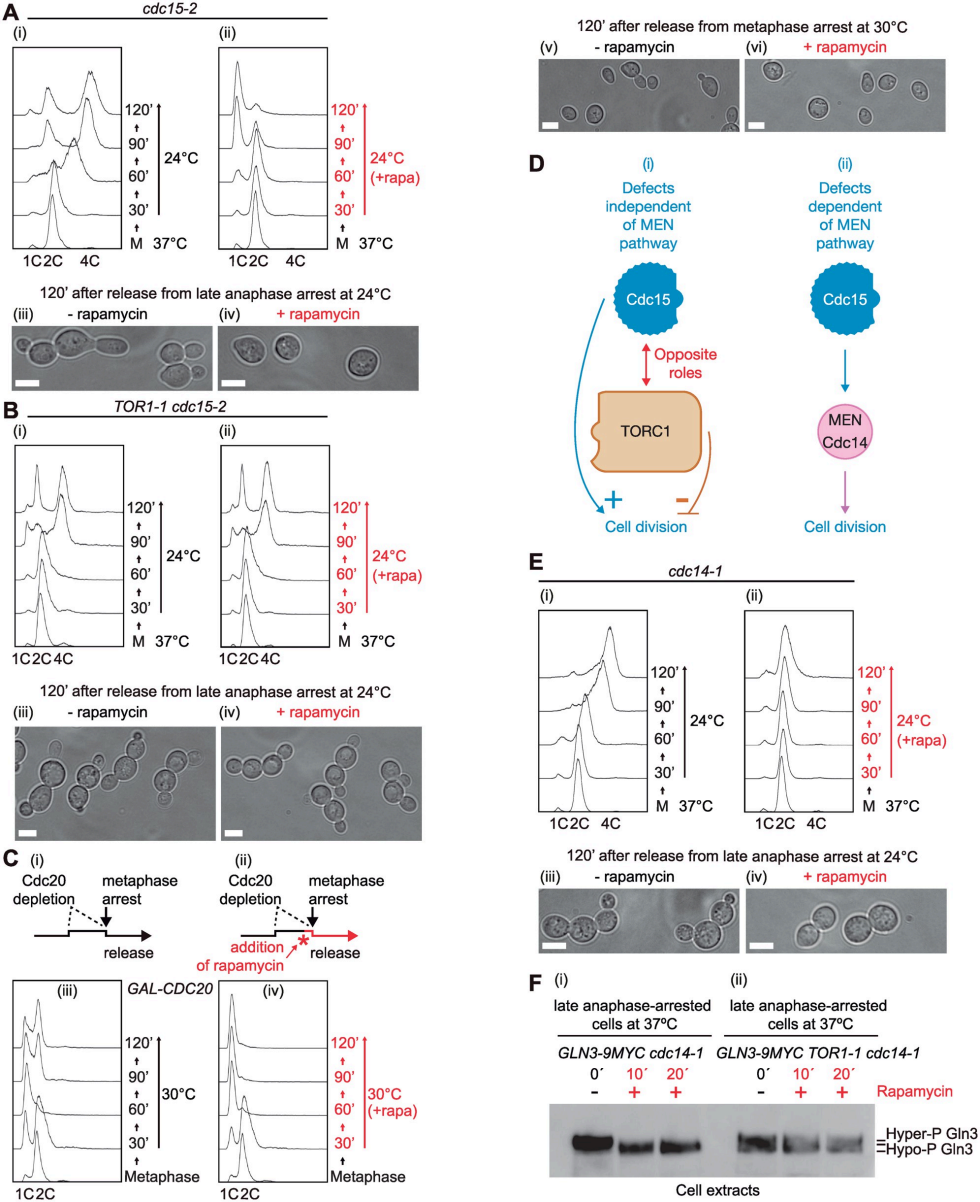
Inactivation of TORC1 in *cdc15-2* cells rescues cell division defect. (A) *cdc15-2* cells (CC2274) were grown in YPD and arrested in late anaphase by shifting the temperature to 37 °C before the addition of rapamycin to half of the culture (ii). Then, cells were released at 24 °C in the absence (i) or presence (ii) of rapamycin. Samples were taken at the indicated times to determine DNA content by FACS analysis. Using light microscopy, we studied cell morphology in the absence (iii) or presence (iv) of rapamycin at the 120 min time point after the release from late anaphase arrest. Scale bars indicate 5 μm. (B) *TOR1-1 cdc15-2* (YMF3240) cells were grown as in A and samples were taken at shown times to determine DNA content ((i) and (ii)) and cell morphology in the absence (iii) or presence (iv) of rapamycin at 120 min after the release from late anaphase arrest. Scale bars indicate 5 μm. (C) Schematic representation of experimental set-up in which *GAL-CDC20* cells (CC5909) were grown in YP raffinose + galactose medium at 30 °C before cells were arrested in metaphase by resuspending in YP raffinose + 0.01% glucose medium and incubated for 3 h. Approximately 20 min before the end of the block, rapamycin was added to half of the culture (ii). Cells were released at 30 °C by the addition of galactose in the absence (i) or presence (ii) of rapamycin. Samples were taken at the indicated times to study DNA content by flow cytometry. FACS profiles and cells pictures are shown in the absence ((iii) and (v)) or presence ((iv) and (vi)) of rapamycin. Scale bars indicate 5 μm. (D) Schematic representation of 2 alternatives for Cdc15 function in our experimental conditions, either independent (i) or dependent of its role in the MEN pathway to release special phosphatase Cdc14, whose role in late mitosis, cytokinesis, and cell separation have been described. (E) *cdc14-1* (YMF3375) cells were grown as in A and samples were taken at the specified times to study cell cycle progression by FACS analysis ((i) and (ii)) and cell morphology in the absence (iii) or presence (iv) of rapamycin at 120 min after the release from late anaphase arrest. Scale bars indicate 5 μm. (F) *GLN3-9MYC cdc14-1* cells (YMF4298) (i) and *GLN3-9MYC TOR1-1 cdc14-1* cells (YMF4456) were arrested at end of anaphase by shifting the temperature at which cells were growing to 37 °C. Subsequently, rapamycin was added and protein extracts were prepared and analysed by immunoblotting at the indicated times while cells were still arrested in late anaphase. FACS graphs can be found in the supplementary FACS file (S1 File). Raw data for blots can be found in supporting information (S2F Raw Images). MEN, mitotic exit network; TOR, target of rapamycin.

To determine the post-anaphase consequences of loss of TORC1 function, we released the rapamycin treated and untreated cells from *cdc15-2* arrest by shifting the cultures back to permissive temperature (24 °C) for 2 h as described for Fig 1A. We found that cells resumed progression through the cell cycle in the absence of rapamycin (TORC1 ON, Fig 2A(i)) or if rapamycin was maintained in the medium (TORC1 OFF, Fig 2A(ii)). Under both conditions, cells completed anaphase as indicated by fluorescence microscopy of spindle dynamics with a version of tubulin fused to GFP (S1B Fig, as described previously in the absence of rapamycin [30]). Unexpectedly, when cells were released at the permissive temperature in the absence of rapamycin, mother and daughter cells stayed together and continued their cell cycle (Fig 2A(i) and 2A(iii)). Despite this failure of mother/daughter separation, both cells grew new buds (Fig 2A(iii)), entered a new round of chromosome replication (Fig 2A(i)), and underwent mitosis (S1B(i) and S1B(iii) Fig), producing multi-budded cells with a DNA content of 4C (Fig 2A(i) and 2A(iii)). In contrast, addition of rapamycin to *cdc15-2* cells rescued the cell division defect and the cells accumulated with a DNA content of 1C (Fig 2A(ii)). Rapamycin blocks budding yeast cells in the G1 phase of the cell cycle [18,20], and cells failed to re-bud (Fig 2A(iv)) and enter new S-phase and mitosis (Figs 2A(ii), S1B(ii) and S1B(iv)). As a control, *cdc15-2* cells were maintained at the restrictive temperature after the addition of rapamycin, instead of releasing them at 24 °C as above (S1C Fig). We found that the sole inactivation of TORC1 was unable to promote the completion of anaphase and cells were kept blocked as large-budded cells with 2C DNA content (S1C Fig). Therefore, as rapamycin specifically inhibits the TORC1 complex [19], these findings suggest that TORC1 might play a role in the regulation of the final steps of cell division after the release from late anaphase arrest (Fig 2A). To confirm that TORC1 function was responsible for that defective cell division, we grew *TOR1-1 cdc15-2* cells in the presence or absence of rapamycin, as in Fig 1A, and found that cell division failed in *TOR1-1 cdc15-2* cells released from late anaphase block, whether or not rapamycin was added (Fig 2B). Taken together, these experiments suggest that TORC1 may regulate some key steps during cell division, dependent upon Tor1 kinase activity, as cells pass from late mitosis to G1.

To investigate whether the MEN kinase Cdc15 shares a role in that control, we examined cells expressing, under the regulatable *GAL* promoter, the activator of anaphase-promoting complex/cyclosome (APC/C), the protein Cdc20. APC/C^Cdc20^ ubiquitinates securin (Pds1 in budding yeast) and marks it for degradation, allowing sister chromatid separation and progression to anaphase [31]. We grew *GAL-CDC20* cells, in which Cdc15 protein was fully functional, asynchronously at 30 °C to subsequently deplete Cdc20 for 3 h, prompting a synchronous metaphase-like mitotic arrest [32] (more than 90% of cells became large-budded cells; Fig 2C). The culture was split evenly in 2 and rapamycin was added for another 20 min to one half to study the result of inhibiting TORC1 activity. Cdc20 expression was resumed at 30 °C in fresh medium containing galactose, inducing the release from metaphase arrest, with or without rapamycin (Fig 2C(i) and 2C(ii)). We found that TORC1 function is unable to block cell division if the Cdc15 kinase was active throughout the experiment (Fig 2C(iii) and 2C(v)), unlike what we had observed in *cdc15-2* cells when Cdc15 had been inactivated (Fig 2A(i) and 2A(iii)).

Cdc15 is part of the MEN signalling cascade that drives the release of Cdc14 phosphatase, which plays key roles in late mitosis, cytokinesis, and cell separation [4,15,33] (Fig 2D). We therefore sought to determine whether the TORC1-dependent block to cell division described above (Fig 2A) was specifically associated with Cdc15 inactivation (Fig 2D(i)), or, on the contrary, was an indirect effect of Cdc15 function depletion attributable to loss of its downstream target Cdc14 (Fig 2D(ii)). We used a strain with a temperature-sensitive mutation in *CDC14*, *cdc14-1*, for comparison with previously grown *cdc15-2*. After growing *cdc14-1* cells at 24 °C, we shifted to 37 °C to inactivate the protein Cdc14-1 and allowed cells to arrest in late anaphase (Fig 2E(i) and 2E(ii)). We split the culture and rapamycin was supplemented to one half of the culture and maintained only in that half till the end of the experiment as in Fig 2A. In contrast to *cdc15-2* cells (Fig 2A(ii) and 2A(iv)), rapamycin inactivation of TORC1 did not rescue the cell division defect of *cdc14-1* cells (Fig 2E(ii) and 2E(iv)). We found that cell division defect also failed in double mutant *cdc14-1 cdc15-2* after the release from late anaphase arrest. Rapamycin treatment did not rescue this defect (S1D(ii) and S1D(iv) Fig), as in single mutant *cdc14-1* (Fig 2E(ii) and 2E(iv)), indicating that Cdc15 function is not associated with Cdc14 for these experimental conditions and that Cdc14 is not functionally related to TORC1. Furthermore, to determine whether Gln3 dephosphorylation after the addition of rapamycin in late anaphase-arrested cells was independent of the phosphatase activity of Cdc14, we grew *GLN3-9MYC cdc14-1* cells as in Fig 1B. We found hypophosphorylated Gln3 when TORC1 was inactivated at 37 °C in late anaphase-arrested cells (Fig 2F(i)). Whereas hyperphosphorylated forms of Gln3 were still found in the rapamycin-resistant *TOR1-1 cdc14-1* cells (Fig 2F(ii)). These finding would suggest that Gln3 dephosphorylation occurred in the absence of Cdc14 function. Taken together, these experiments suggest that loss of Cdc15 activity in anaphase causes defects in late cell division, independently of Cdc15’s role in the release of Cdc14 (Fig 2D), that are rescued by TORC1 inactivation. Thus, Cdc15 and TORC1 might play opposite roles at the end of the cell cycle and TORC1 kinase activity may need to be down-regulated to allow cells to proceed with late cell-cycle events (Fig 2D(i)).

### TORC1 blocks cell separation by promoting Cbk1 phosphorylation

**Fig 3:**
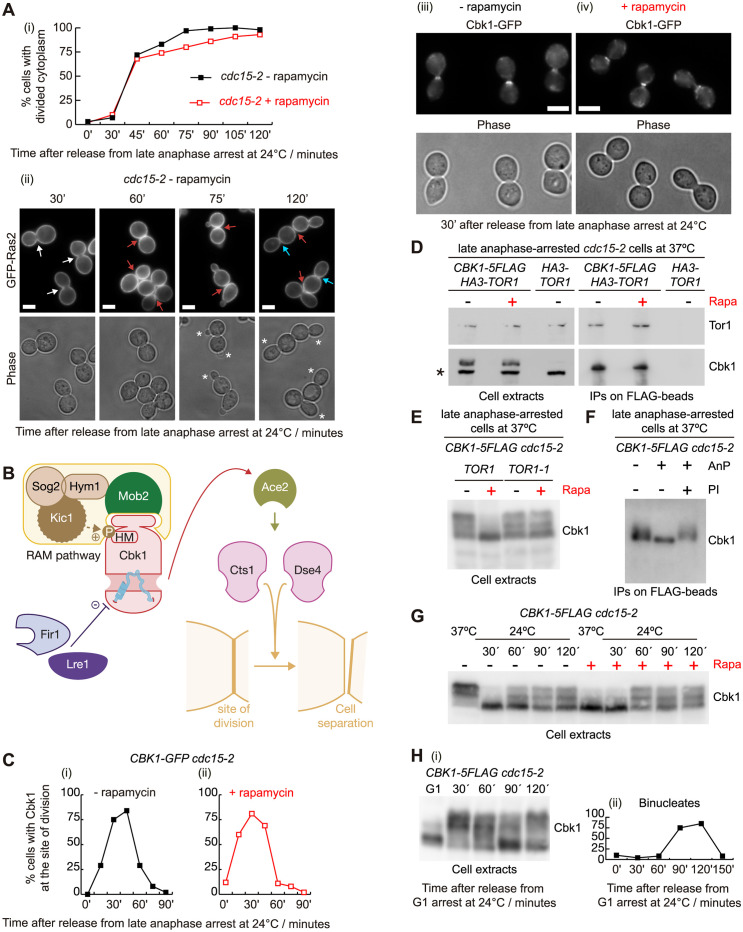
TORC1 regulates and participates in the phosphorylation of Cbk1 to negatively control cell separation. (A) *3GFP-RAS2 cdc15-2* (CC6298) cells were grown as described in Fig 1A and samples were taken at the shown times after the late anaphase release to determine the percentage of the cells with divided cytoplasm (i). Examples of 3GFP-Ras2 tagged cells in the absence of rapamycin are shown for the indicated time points (ii). White arrows indicate undivided cytoplasm, red arrows denote cells where examination of each z-level at the bud neck showed divided cytoplasm, blue arrows correspond to new bud neck with undivided cytoplasm, and new buds are marked with white asterisks. Scale bars indicate 5 μm. (B) Schematic illustration of the RAM network and negative regulators of Cbk1 (Lre1 and Fir1). Upstream factors of the RAM cascade are required to phosphorylate Cbk1 HM on residue 743, which is essential for Cbk1 to turn active. Cbk1 positively regulates the transcription factor Ace by phosphorylation, which promotes the expression of hydrolases Cts1 and Dse4 to finally induce cell separation [4]. (C) *CBK1-GFP cdc15-2* (YMF3513) cells were grown in YPD and arrested in late anaphase by shifting the temperature to 37 °C before the addition of rapamycin to half of the culture. Subsequently, to allow progression through the cell cycle, cells were released in the absence (−) or presence (+) of rapamycin. Samples were taken at the indicated times. Using fluorescence microscopy, the proportion of cells with Cbk1 at the site of division in the absence (i) or presence (ii) of rapamycin was determined. Examples of cells with Cbk1-GFP at the bud neck 30 min after the release at 24 °C are shown in the absence (iii) or presence (iv) of rapamycin. Scale bars indicate 5 μm. (D) *CBK1-5FLAG HA3-TOR1 cdc15-2* (YMF3608) and control cells (YMF3606) were grown at 24 °C in YPD medium and arrested in late anaphase. Rapamycin was added to half of the culture for 20 min. Protein extracts were prepared from cells arrested in anaphase in the absence or presence of rapamycin (YMF3608) or only in the absence of rapamycin (control cells) before immunoprecipitation of Cbk1 on FLAG-beads and detection of the indicated proteins by immunoblotting. (E) *CBK1-5FLAG cdc15-2* (YMF3302) and *CBK1-5FLAG TOR1-1 cdc15-2* (YMF3300) cells were grown in YPD and arrested in late anaphase by shifting the temperature to 37 °C; rapamycin was added (+) to half of each culture. Protein extracts were prepared from cells arrested in anaphase in the absence (−) or the presence (+) of rapamycin and subsequently analysed by immunoblotting. (F) *CBK1-5FLAG cdc15-2* (YMF3546) cells were grown in YPD and arrested in late anaphase at 37 °C before the immunoprecipitation of Cbk1 and incubation with AnP or phosphatase together with PIs before the detection of Cbk1 by immunoblotting. (G) *CBK1-5FLAG cdc15-2* cells (YMF3302) were grown as in (E). Then, cells were released at 24 °C in the absence (−) or presence (+) of rapamycin. Samples were taken at the indicated times and protein extracts were prepared and analysed by immunoblotting. (H) *CBK1-5FLAG cdc15-2* cells (YMF3302) were grown at 24 °C in YPD medium and synchronised in the G1 phase with mating pheromone before the cells were released from the G1 block to allow progression through the cell cycle at 24 °C. Samples were taken at the indicated times to prepare protein extracts before the detection of Cbk1 by immunoblotting (i) and to count the proportion of binucleate cells as cells were progressing through the cell cycle at 24 °C. Underlying data for all the graphs can be found in S1 Data file. Raw data for blots can be found in supporting information (S3 Raw Images). AnP, Antarctic Phosphatase; HM, hydrophobic motif; PI, phosphatase inhibitor; RAM, regulation of Ace2 and morphogenesis; TOR, target of rapamycin.

We aimed to determine whether *cdc15-2* cells were defective in cytokinesis and/or cell separation. First, we investigated whether *cdc15-2* cells performed cytokinesis in the same experimental conditions as described above (Fig 1A). To study actomyosin ring formation and contraction, we used fluorescence microscopy of a GFP-tagged form of the protein Inn1, a component of the actomyosin ring [34] (S1E Fig). We found that Inn1 dynamics were similar in the absence or presence of rapamycin, indicating that cytokinetic machinery itself is not sensitive to the status of TORC1 activity (S1E Fig). To analyse the division of cytoplasm, we monitored plasma membrane dynamics in *cdc15-2* cells expressing, under its own promoter, the small G-protein Ras2 fused to 3 copies of GFP (Fig 3A). We grew *3GFP-RAS2 cdc15-2* cells as in prior experiments (Fig 2A). Live cells were examined over the course of 2 h after the anaphase block and release in the absence or presence of rapamycin (Fig 3A). Cytoplasmic division occurred with similar kinetics after anaphase release in almost all cells, regardless of rapamycin treatment (Fig 3A). We also examined *3GFP-RAS2 cdc14-1* cells to confirm that cells performed cytokinesis but not cell separation after mitotic release, independently of rapamycin addition (S1F Fig). This again showed that TORC1 inactivation rescued cell division defect in *cdc15-2* cells but not in *cdc14-1* cells following release from late anaphase block. Taken together, these experiments indicate that cell division defect observed after the release of *cdc15-2* cells, while TORC1 is functioning, is not due to a cytokinesis failure, but to a cell separation defect, which prompted us to investigate the molecular mechanism.

The budding yeast NDR/LATS kinase Cbk1 is the downstream-most factor of the RAM network that, together with its tightly-bound coactivator Mob2, phosphorylates the transcription factor Ace2 in anaphase [4] (Fig 3B). After mitotic exit Ace2 localises specifically to daughter cell nuclei, where it drives expression of cell separation enzymes that include the chitinase Cts1 and the endoglucanase Dse4 that are secreted at the site of division to promote cell separation [35–37] (Fig 3B). To confirm that depletion of a key cell separation factor like Cbk1 should block the rescue promoted by rapamycin in *cdc15-2* cells (Fig 2A), we generated a yeast strain for which the “auxin inducible degron” (“aid”) cassette [38] was fused to the C-terminal end of *CBK1*, in its own locus, to conditionally inactivate Cbk1 protein (S2A Fig). Then, to confirm that the new *cbk1-*aid mutant was able to inactivate Cbk1 function following the addition of auxins (restrictive conditions), we grew *cbk1-aid* cells asynchronously at 24 °C and found multi-budded cells with a DNA content of 2C or 4C after the depletion of Cbk1 for 6 h (S2B Fig). Subsequently, to confirm that depletion of Cbk1 blocked the rescue of cell separation driven by TORC1 inactivation, we used *cbk1-aid cdc15-2* cells (S2C Fig). Asynchronous culture of *cbk1-aid cdc15-2* was grown at 24 °C before synchronisation in late anaphase by inactivation of Cdc15 at 37 °C (S2C Fig). Once cells were synchronised, Cbk1 was depleted for 50 min. Afterwards, the culture was divided in 2 and rapamycin was added to one half of the culture for 20 min before the release at 24 °C for 2 h. Rapamycin was maintained in the same culture (S2C Fig). Inactivation of TORC1 was unable to rescue cell separation defect of *cdc15-2* cells if Cbk1 had been depleted (S2D(ii) and S2D(iv) Fig). Cells accumulated as large-budded cells with a DNA content of 2C (S2D(ii) and S2D(iv) Fig). To ratify this result, we generated a *cbk1-aid cdc15-2* strain expressing, under the control of its own promoter, an extra copy of Cbk1 containing the mutation D475A, which turns Cbk1 into a catalytically inactive kinase (“kinase dead”) [39] (S2E(i) Fig). This strain was grown as described in S2C Fig. Restrictive conditions (addition of auxins) promoted Cbk1 depletion, leaving cells with Cbk1-D475A to study functional consequences (S2E(ii) Fig). We found that inactivation of Cbk1 catalytic function was, again, enough to prevent rapamycin rescue of the cell separation defect after anaphase release (S2F(ii) and S2F(iv) Fig). As control, inactivation of TORC1 rescued cell separation defects in *cbk1-aid cdc15-2* cells expressing wild-type (wt) version of *CBK1* (S2G(ii) and S2G(iv) Fig).

We aimed to understand the biological consequences of TORC1 inactivation in *cdc15-2* cells after TORC1 had been inhibited and cells were released from anaphase arrest. Firstly, we focused on the study of Cbk1 localization at the site of division. Cbk1 concentrates at the division site following anaphase [36,40,41]. We found dynamics of GFP-tagged Cbk1 were similar in the experimental conditions described in Fig 2A, independently of the addition of rapamycin (Fig 3C). As TORC1 is a kinase, we hypothesised TORC1 could bind and phosphorylate Cbk1. To investigate whether Cbk1 was able to physically interact with TORC1, we synchronised *CBK1-5FLAG HA3-TOR1 cdc15-2* cells in late anaphase at 37 °C and added rapamycin to half of the culture for another 20 min. Subsequently, we pulled down the protein Cbk1-5FLAG and we found that Tor1 interacted with Cbk1 even if TORC1 had been inhibited (Fig 3D). These data would indicate that TORC1 and Cbk1 interact independently of whether TORC1 is active. To determine if Cbk1 protein mobility on SDS-PAGE was altered when TORC1 was inhibited by the addition of rapamycin in *cdc15-2* cells arrested in anaphase at 37 °C, we grew *CBK1-5FLAG cdc15-2* strain at 24 °C and later synchronised cells in late anaphase by raising the temperature to 37 °C as described for Fig 1B. We divided the culture in 2 and rapamycin was added to one half of the culture for 20 min. We found that blocking the ability of TORC1 to phosphorylate its substrates promoted a clear change in the electrophoretic mobility of Cbk1 while cells are arrested in anaphase at 37 °C (Fig 3E, compare *TOR1*—rapamycin and + rapamycin). To confirm that the serine/threonine kinase activity of TOR1 was responsible for that shift, *TOR1-1 CBK1-5FLAG cdc15-2* strain was grown in the same fashion. We found that there was no mobility shift in rapamycin-resistant *TOR1-1 cdc15-2* cells, compare with *cdc15-2* control cells, despite the presence of rapamycin (Fig 3E, compare *TOR1* and *TOR1-1* both + rapamycin). Furthermore, to determine that Cbk1 mobility shift corresponded to phosphorylation, Cbk1-5FLAG was immunoprecipitated from *cdc15-2* arrested in late anaphase by raising the temperature to 37 °C. Then, addition of Antarctic Phosphatase (AnP) to the Cbk1 IP promoted Cbk1 bands to collapse (Fig 3F). This band collapse exhibits similarity to Cbk1’s electrophoretic mobility in the presence of rapamycin while cells are still arrested in late anaphase (compare Fig 2E and 2F). To investigate whether Cbk1 mobility shift varied, depending on TORC1 activity, after late anaphase release, samples were taken every 30 min for 2 h at 24 °C in the presence of the absence of rapamycin (Fig 3G). We found Cbk1 mobility was the same independently of the presence of rapamycin once cells were released. At 24 °C, Cdc15 is active which promotes MEN-triggered Cdc14 phosphatase and leads to dephosphorylation of Cbk1 (Fig 3G). Finally, we arrested *cdc15-2* cells in G1 at 24 °C, then released cells at the same temperature and found that Cbk1 mobility at 90 min (Fig 3H(i)), precisely the time at which cells had initiated anaphase (Fig 3H(ii)), was similar as in anaphase-blocked *cdc15-2* cells at 37 °C after addition of rapamycin (Fig 3G, see 37°C + Rapa). Therefore, mobility changes for Cbk1 and Gln3 both might occur at the onset of anaphase (compare Figs 1D and 3H). Overall, these data indicated that TORC1 interacts with Cbk1, regulates and participates in the phosphorylation of Cbk1. Interestingly, phosphorylation in anaphase blocks the following cell separation in budding yeast, independently if Cbk1 is dephosphorylated after anaphase. Furthermore, Cbk1 mobility shifted at 90 min after G1 release, when our data suggested that TORC1 might be inactivated during mitosis too.

### Function of known Cbk1 regulators is independent of TORC1

Like other NDR/LATS kinases, Cbk1 is positively regulated by autophosphorylation of its activation loop (T-loop) and by Mst/hippo kinase phosphorylation of a C-terminal hydrophobic motif (HM) [4,39,42]. Cbk1 is the downstream-most component of the RAM network, and all other known elements of the cascade (Kic1, Hym1, Tao3, and Sog2) are required for phosphorylation of threonine 743 located on Cbk1 HM [4] (Fig 3B). To determine if constitutive activation of Cbk1 HM site avoided TORC1 inhibition of cell separation, we expressed a version of Cbk1 in which threonine at position 743 was replaced by glutamic acid (*CBK1-T743E*) to mimic phosphorylation of the HM site [42]. We grew *cdc15-2* cells with *CBK1-T743E* as the only copy of Cbk1 in the same conditions as described for Fig 2A. We showed that *CBK1-T743E* was unable to rescue cell separation defects while TORC1 was active (S3A(i) Fig), which would indicate that TORC1 control over Cbk1 might not be only via regulating phosphorylation of HM site. On the other hand, Lre1 and Fir1 proteins have been described to negatively control the activity of Cbk1. Lre1 interacts with Cbk1 and its regulating factor, Mob2, to directly inhibits Cbk1 kinase activity [43] and Fir1 blocks cell separation via its ability to bind Cbk1 [5]. To investigate whether TORC1 inhibition of cell separation was signalled through Lre1 or Fir1, *lre1Δ cdc15-2* and *fir1Δ cdc15-2* yeast strains were cultured as for Fig 2A. We found that the lack of Lre1 or Fir1 is not enough to prevent a defect in cell separation when TORC1 is active in the absence of rapamycin (S3B(i) and S3C(i) Fig). These findings suggested that TORC1 control over Cbk1 could be independent of the described mechanisms that regulate Cbk1 function.

### Identification of TORC1-regulated phosphorylation sites in Cbk1 and its interactors

**Fig 4:**
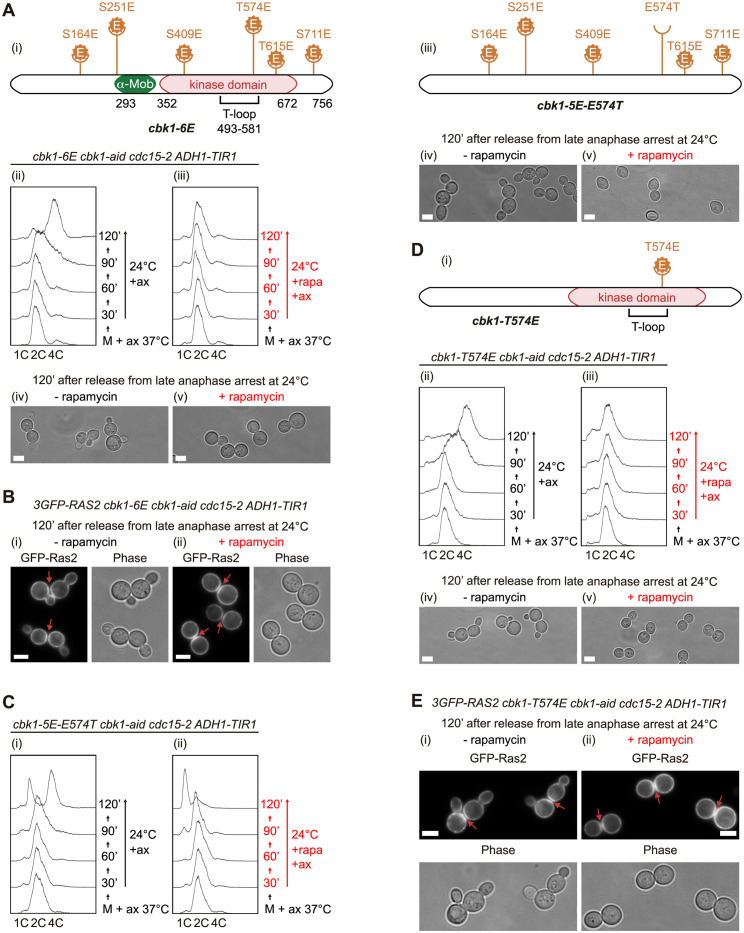
Phosphomimetic Cbk1-T574E shows a TORC1-dependent cell separation defect in the presence of rapamycin. (A) Schematic illustration of *cbk1-6E* mutant in which phosphosites containing serines or threonines followed by prolines were changed to glutamic acid to mimic phosphorylations [42]. “α-Mob” denotes interacting domain with Cbk1 regulatory subunit Mob2. The kinase domain and activation loop (T-loop) are highlighted (i). *cbk1-6E cbk1-aid cdc15-2* (YMF4019) cells were grown in YPD and arrested in late anaphase by shifting the temperature to 37 °C before the addition of NAA and IAA auxins. Subsequently, cells were incubated with DMSO (ii) or rapamycin (iii) for 20 min while still arrested in anaphase. Cells were released in the absence (ii) or presence (iii) of rapamycin and with the NAA and IAA auxins present in the medium throughout the rest of the experiment. Samples were taken at the indicated times to investigate cell-cycle progression by flow cytometry (ii and iii) and cell morphology by light microscopy in the absence (iv) or in the presence (v) of rapamycin at 120 min after the release from late anaphase arrest. Scale bars indicate 5 μm. (B) *3GFP-RAS2 cbk1-6E cbk1-aid cdc15-2* (YMF4079) cells were grown as described in A and samples were taken at 120 min after the late anaphase release. Examples of cells are shown in the absence of rapamycin (i) or in the presence (ii) of rapamycin. Red arrows show mother and daughter cells that have separated their cytoplasm. Scale bars indicate 5 μm. (C) *cbk1-5E-E574T cbk1-aid cdc15-2* (YMF3997) cells were grown as described in A. Samples were taken at the specified times to determine DNA content in the absence (i) or presence (ii) of rapamycin. Schematic illustration of *cbk1-5E-E574T* mutant in which mutated glutamic acid in position 574 was reverted to threonine and the rest of the glutamic acids in Cbk1-6E were maintained (ii). Examples of cells in the absence (iv) or in the presence (v) of rapamycin at the 120 min time point after the release from late anaphase arrest. Scale bars indicate 5 μm. (D) Schematic representation of *cbk1-T574E* mutant in which DNA sequence that encodes for threonine 574 was mutated to be translated as a glutamic acid (i). *cbk1-T574E cbk1-aid cdc15-2* (YMF3944) cells were grown as described in A. DNA content analysis and microscopy were performed as described above. Scale bars indicate 5 μm. (E) *3GFP-RAS2 cbk1-T574E cbk1-aid cdc15-2* (YMF4021) cells were grown as described in A and samples were taken at 120 min after the late anaphase release. Examples of cells in the absence (i) or in the presence (ii) of rapamycin. Red arrows show cells where examination of each z-level at the bud neck showed separated cytoplasm. Scale bars indicate 5 μm. FACS graphs can be found in the supplementary FACS file (S1 File). TOR, target of rapamycin.

To investigate TORC1-dependent changes in phosphorylation profile of Cbk1, first we used mass spectrometry and, second, we expressed phosphomimetic or non-phosphorylatable versions of Cbk1. We grew *cdc15-2* and *TOR1-1 cdc15-2 cells*, both expressing *CBK1-GFP* under the control of its own promoter, in the same conditions as described for Fig 2A and made 2 cell extracts in which TORC1 was active (*cdc15-2* and *TOR1-1 cdc15-2 + rapamycin*) and one in which TORC1 was inactive (*cdc15-2 + rapamycin*). Three biological replicates of each described cells and conditions were performed and compared with 3 biological replicates of untagged control cells. Cbk1 was immunoprecipitated and the isolated material was analysed by mass spectrometry. We found 5 phosphorylation sites in Cbk1 while TORC1 was active: 174, 177, 187, 348, 693, and 743 (Tables 1 and S1). Phosphorylation on residue 743 is regulated by RAM pathway and can be found in anaphase-arrested *cdc15-2* cells [42]. The rest of the phosphorylation sites might be controlled by TORC1. Each phosphorylation site was identified once, which prompted us to follow a second strategy expressing phosphomimetic or non-phosphorylatable Cbk1. A recent study has reviewed all reported direct substrates for TORC1 with emphasis on mammalian TOR (mTOR) [8]. Authors have described that TORC1 common consensus phosphorylation motif contains mainly a proline after serine or threonine (S/TP) [8]. To investigate Cbk1 sites that might be important in the regulation of Cbk1 by TORC1, we decided to investigate a version of Cbk1, previously used [42], in which a subset of serines or threonines followed by proline had been changed to glutamic acid to mimic phosphorylation (*cbk1-6E*) (Figs 4A(i) and S4). We expressed Cbk1-6E under the control of its own promoter in *cbk1-aid cdc15-2* cells and grew them as described for experiment in S2C and S2E Fig. Cells were synchronised in late anaphase, then Cbk1-aid was depleted and cells were left expressing Cbk1-6E as the only copy of Cbk1 to assess the immediate consequences after the release in the absence or presence of rapamycin. We found large budded cells with a DNA content of 2C in the presence of rapamycin (Fig 4A(iii) and 4A(v)), unlike control cells (Fig 2A(ii) and 2A(iv)). Therefore, inactivation of TORC1 was unable to rescue cell separation defects associated with *cdc15-2* cells expressing Cbk1-6E after anaphase release, which was confirmed following the plasma membrane dynamics using fluorescence microscopy (Fig 4B). To identify which site was important for TORC1 regulation, we decided to mutate back one by one all the sites in *cbk1-6E* to the corresponding wt residue (*cbk1-5E-E-site-S/T*). We grew all 6 new mutants in *cbk1-aid cdc15-2* cells and investigated their ability to rescue the cell separation defect in the presence of rapamycin (Figs 4C and S5). We found that *cbk1-aid cdc15-2* cells expressing *cbk1-5E* versions in which T574E was changed back to the wt residue (Cbk1-5E-E574T) accumulated mainly with a DNA content of 1C in the presence of rapamycin (Fig 4C(ii)). In addition, Cbk1-5E-E409S partially rescued too (S5C Fig), unlike the other *cbk1-5E-E-site-S/T* mutants (S5A, S5B, S5D and S5E Fig). We confirmed these results by expressing Cbk1-5E-E574T and Cbk1-5E-E409S in *cbk1Δ* cells (S6 Fig). Both residues are perfectly conserved in a range of fungal species, unlike the other residues (S4 Fig). Taken together, these data showed that residues S409 and T574 might play an important role. Serine 409 is located in the kinase domain and threonine 574 in the activation loop, which may regulate substrate binding with kinase Cbk1 (S5F Fig). Then, using wt *CBK1*, we decided to generate a mutant in which residue T574 was changed to glutamic acid to assess the consequences. We grew *cbk1-T574E cbk1-aid cdc15-2* cells in the same way as described above for Fig 4A and found that cells accumulated as 2C DNA content after the addition of rapamycin (Fig 4D(iii)). Using fluorescence microscopy, we determined that expression of Cbk1-T574E blocked cell separation (Fig 4E), which suggested that T574 could be a TORC1 phosphorylation site that negatively regulates cell separation. This phosphomimetic version of Cbk1 could be used to study how TORC1 activity controls cell separation. A version of Cbk1 in which threonine 574 was changed to alanine was unable to rescue cell separation defect in the absence of rapamycin (S5G(i) Fig), which confirmed that there must be other residues, other than T574, that TORC1 could regulate. To search further for more key residues whose phosphorylation might be controlled by TORC1, we used a version of Cbk1, previously published [42], in which all 9 serines and threonines followed by proline were changed to alanine: Cbk1-9A. Expression of this allele was unable to rescue cell separation defect in the absence of rapamycin (S6E(i) Fig), which would suggest that there must be other sites regulated by TORC1, within Cbk1 sequence or other proteins, that are able to block cell separation in budding yeast. To explore whether TORC1 may participate and control, either directly or indirectly, the phosphorylation of described Cbk1 interactors, we analysed by mass spectrometry the phosphorylation profile of known interactors immunoprecipitated with Cbk1-GFP in the experiment described above for Tables 1 and S1. We identified specific sites for Ace2, Fir1, Kin2, Lre1, Mob2, Ssd1, Tao3, and Yol036w (Tables 1 and S1). All these proteins were previously found to be phosphorylated in a TORC1-dependent manner [44–46]. These findings would indicate that TORC1 activity promotes, either directly or indirectly, changes in the phosphorylation pattern of Cbk1 and some of its interactors.

**Table 1:** Identification of TORC1-regulated phosphorylation sites in Cbk1 and its interactors.

Protein	Score	No of replicas in which phosphorylation was found on *cdc15-2* & *cdc15-2 TOR1-1*+ rapamycin	No of replicas in which phosphorylation was found on *cdc15-2* + rapamycin	Sequence	Peptide position	Protein site
Ace2	84,035	1		LVSGATNSNSKPGSPVILK	10	249
Cbk1	67,997	1		SNGSYSSGLR	1	174
Cbk1	67,997	1		SNGSYSSGLR	4	177
Cbk1	53,777	1		SNGSYSSGLRSVKSFQR	14	187
Cbk1	100,43	1		TRLSLEDFHTVK	4	348
Cbk1	90,149	1		LSSITDTR	3	693
Cbk1	56,468	1		QGGSAPVKEDLPFIGYTYSR	17	743
Fir1	71,349	2		SLPVTPVK	5	6
Fir1	66,879	1		ATEVETSINENTSNISQVSPLNLSFDRPPPLTPEK	19	201
Fir1	77,439	1		ATEVETSINENTSNISQVSPLNLSFDRPPPLTPEK	32	214
Fir1	103,85	1		FFQQFEPSEEPTSPTR	8	394
Fir1	103,95	2		NSGDTNNEDFLKVDTSPVNQSFESR	21	455
Fir1	73,616	2		NGSFLQEISVPSIQIIPDESISHTR	3	552
Kin2	88,507		2	GGSLSPTPEAFNDTR	5	24
Lre1	87,659	1		TNSQLSKDILMGEPGDMVDLSSFVNAQR	3	488
Lre1	170,5	1		DILMGEPGDMVDLSSFVNAQR	15	507
Lre1	78,413		2	KASNETGDLVFSLSQDDDALK	14	527
Lre1	321,13		2	TFHASNSAATSNESWCISDDALGK	11	545
Mob2	263,16		2	NQPLNVAQPPAMNTIYSSPHSSNSR	18	35
Mob2	177,61	1		NQPLNVAQPPAMNTIYSSPHSSNSR	24	41
Mob2	63,037	2		RHSQTSFPAQK	6	59
Mob2	154,84	1		STPQSQQLTSTTPQSQQQEASER	5	69
Ssd1	161,14	3		SQSELTNLMIEQFTLQK	1	40
Tao3	156,67	1		HVSSSFNNKVPLIK	3	300
Tao3	67,456	1		SQQIFTVQHQK	1	1144
Tao3	80,342	1		SDSIETTRTDQTFSFESAPQLYDKK	12	2325
Tao3	74,786	1		TDQTFSFESAPQLYDKK	6	2327
Tao3	113,47	1		TPSNVSFKTHLADSFAVK	1	2350
Yol036W	109,38		1	SATPTSSSTTVTTHLQNIKEEETNDDELTQVDR	3	35
Yol036W	383,02	1		SATPTSSSTTVTTHLQNIKEEETNDDELTQVDRSSPR	35	67
Yol036W	109,38		1	ASTSLPESSTDDISPLREEGK	3	595
Yol036W	117,67	1		ASTSLPESSTDDISPLREEGK	4	596

Identification of phosphorylation sites for described Cbk1 interactors. Experimental details can be found in the legend for S8 Fig. Number or replicates in which phosphorylation was found, whether TORC1 was active or inactive, is indicated. Go to S1 Table for full information.

### Secretory vesicle transport is active and chitinase Cts1 accumulates in *cdc15-2* cells but is unable to promote cell separation in the absence of rapamycin

**Fig 5:**
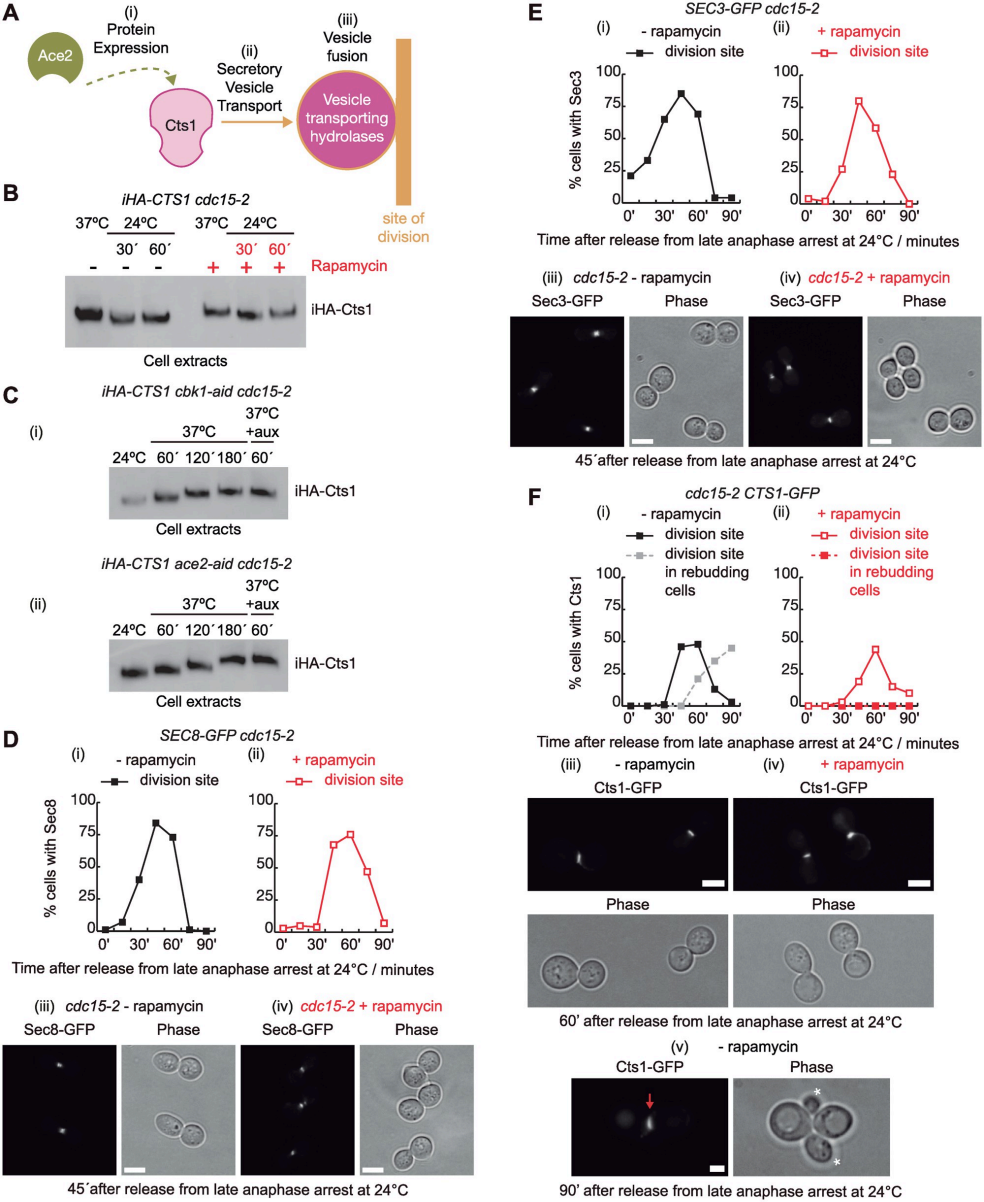
*cdc15-2* cells defective in cell separation accumulate chitinase Cts1 in the absence of rapamycin. (A) Three different hypotheses for what cellular process was affected in *cdc15-2* cells to explain their cell separation defect. (B) *iHA-CTS1 cdc15-2* (YMF3845) cells were grown in YPD and arrested in late anaphase by shifting the temperature to 37 °C before the addition of DMSO (−) or rapamycin (+) for 20 min. Subsequently, cells were released from the anaphase arrest in the absence (−) or presence (+) of rapamycin before protein extracts were prepared from shown time points and analysed by immunoblotting. Raw data for blot can be found in supporting information (S4B Raw Images). (C) *iHA-CTS1 cbk1-aid cdc15-2* (YMF3991) and *iHA-CTS1 ace2-aid cdc15-2* (YMF3969) cells were grown in YPD asynchronously and culture was shifted to 37 °C to allow late-anaphase arrest after inactivation of Cdc15 function. Subsequently, Cbk1-aid and Ace2-aid proteins were depleted. To study iHA-Cts1, protein extracts were collected at the indicated time points, followed by immunoblotting. Raw data for blot can be found in Supporting information (S4C Raw Images). (D) *SEC8-GFP cdc15-2* (YMF4002) and (E) *SEC3-GFP cdc15-2* (YMF4003) cells were grown in YPD and arrested in late anaphase by shifting the temperature to 37 °C before the addition of rapamycin to half of the culture for 20 min. Cells were released to allow progression through the cell cycle in the absence (−) or presence (+) of rapamycin. Samples were taken at the indicated times. Using fluorescence microscopy, the proportion of cells with Sec8 (D) or Sec3 (E) at the site of division in the absence (i) or presence (ii) of rapamycin was determined. Examples of cells with Sec8-GFP (D) or Sec3-GFP (E) at the site of division after 45 min from the release at 24 °C are shown in the absence (iii) or presence (iv) of rapamycin. Scale bars indicate 5 μm. (F) *CTS1-GFPEnvy cdc15-2* cells (YMF4145) were grown in YPD and arrested in late anaphase by shifting the temperature to 37 °C before the addition of DMSO or rapamycin for 20 min. Subsequently, cells were released in the absence (i) or presence (ii) of rapamycin to allow progression through the cell cycle. Samples were taken at the indicated times to establish the proportion of cells with Cts1 at the site of division site in the absence (i) or the presence (ii) of rapamycin. Examples of cells with Cts1-GFP at the bud neck are shown for the 60 min time point after the release at 24 °C in the absence (iii) or the presence (iv) of rapamycin, scale bars indicate 5 μm. Example of cell that have initiated a new cell cycle at 90 min from the release, which can be determined as mother and daughter cells are rebudding, and maintained Cts1-GFP at the site of division (v). Scale bars indicate 2 μm. Underlying data for all the graphs can be found in S1 Data file.

So far, we showed that TORC1 regulates and participates in the phosphorylation of Cbk1, phosphomimetic mutations reproduce TORC1 control, and TORC1 alters the phosphorylation landscape of Cbk1 and its interactors. Next, we aimed to address what cellular process was affected in *cdc15-2* cells to explain their cell separation defect (Fig 5A).

Cbk1 interacts with and phosphorylates the transcription factor Ace2 at its nuclear export sequence, which retains Ace2 in the daughter nucleus [47,48]. Ace2 is required to activate a daughter-specific transcription program that express proteins such as Cts1 and Dse4, whose role is promoting cell separation via the degradation of the septum that joins mother and daughter cells [36] (Fig 3B). Cts1 is an endochitinase that degrades chitin located in the primary septum [49,50], whereas Dse4 is a glucanase that contributes to septum dissolution after cytokinesis [36,37]. We hypothesised that Cbk1 activity might be compromised in anaphase-arrested *cdc15-2* cells and therefore transcription of Ace2-regulated genes could be defective, which would explain defects associated with *cdc15-2* cells (Fig 5A(i)). First, to determine the expression of cell separation enzymes Cts1 and Dse4 in our experimental system, we grew *iHA-CTS1 cdc15-2* and *DSE4-6HA cdc15-2* cells as described for Fig 1A. Surprisingly, Cts1 and Dse4 were detected at the end of the anaphase arrest and for the next 60 min, independently of rapamycin (Figs 5B and S7A). To investigate whether the presence of Cts1 before the release at 24 °C was independent of Cbk1 and Ace2 function, *iHA-CTS1 cbk1-aid cdc15-2* and *iHA-CTS1 ace2-aid cdc15-2* cells were grown asynchronously at 24 °C and temperature was raised to 37 °C to inactivate Cdc15. Subsequently, either Cbk1 or Ace2 were depleted after the addition of auxins. Protein samples were taken to show that Cts1 is stable as it accumulated despite the lack of Cbk1 and Ace2 in anaphase-arrested cells (Fig 5C). Therefore, detection of Cts1 and Dse4 indicated that cell separation defect in *cdc15-2* cells is not due to a failure in the expression of hydrolases. Then, the defective cellular process must be either secretory vesicle traffic towards the site of division and/or the fusion of those vesicles to the plasma membrane. Interestingly, it has been recently described, although no molecular mechanism was detailed, that Cbk1 induces Cts1 secretion independently of Cbk1 role in promoting Cts1 expression, which would suggest a role for Cbk1 in vesicle transport and/or membrane fusion [5] (Fig 5A(ii) and 5A(iii)).

At the end of the cell cycle, Cts1 is transported and localises at the site of division in large-budded cells that have not completed cell separation [17,36]. Transport of secretory vesicles to the site of division requires the octameric protein complex named exocyst [51,52]. Some exocyst components, Sec8 among them, travel with secretory vesicles, whereas others as Sec3 interact with the plasma membrane via its amino-terminal pleckstrin homology (PH) domain. The rest of the exocyst members interact with Sec3 to form the fully assembled exocyst at the plasma membrane. We confirmed that both exocyst components, Sec8 and Sec3, localised at the site of division after *cdc15-2* anaphase arrest and release, independently of the absence or presence of rapamycin (Fig 5D and 5E), which would suggest that secretory vesicle transport is not defective in *cdc15-2* cells after release.

Next, since secretory vesicle transport was functional and to confirm that Cts1 localised at the site of division at the end of the cell cycle (Fig 5A(ii)), we grew *CTS1-GFP cdc15-2* and found that Cts1 accumulated in cells that had failed cell separation and were able to form new buds in the absence of rapamycin (Fig 5F(i), 5F(iii) and 5F(v)), unlike what happened when TORC1 activity was inhibited by the addition of rapamycin, which promoted the localization of Cts1 with similar dynamics as previously described (Fig 5F (ii) and 5F (iv)). Therefore, despite the presence of Cts1 at the site of division (Fig 5F(i), 5F(iii) and 5F(v)), cell separation failed in the absence of rapamycin, which would suggest that a late step in secretory vesicle transport might be defective.

As control, we grew *cdc14-1 iHA-CTS1* cells and found that, as for *cdc15-2*, those cells contained Cts1 after the anaphase arrest and release (S7B Fig). Rapamycin was unable to rescue defect in Cts1 localization at the site of division in *cdc14-1* cells (S7C Fig), which showed that Cts1 is unable to be secreted to the site of division in *cdc14-1* cells. Taken together, *cdc15-2* cells are able to transport Cts1, unlike *cdc14-1* cells, but they might have a problem in the fusion of Cts1 containing vesicles into the plasma membrane, which could explain the cell separation defect associated with *cdc15-2* cells.

### A t-SNARE mutant and phosphomimetic Cbk1-T574E show a cell separation defect despite the accumulation of Cts1 at the site of division in the presence of rapamycin

**Fig 6:**
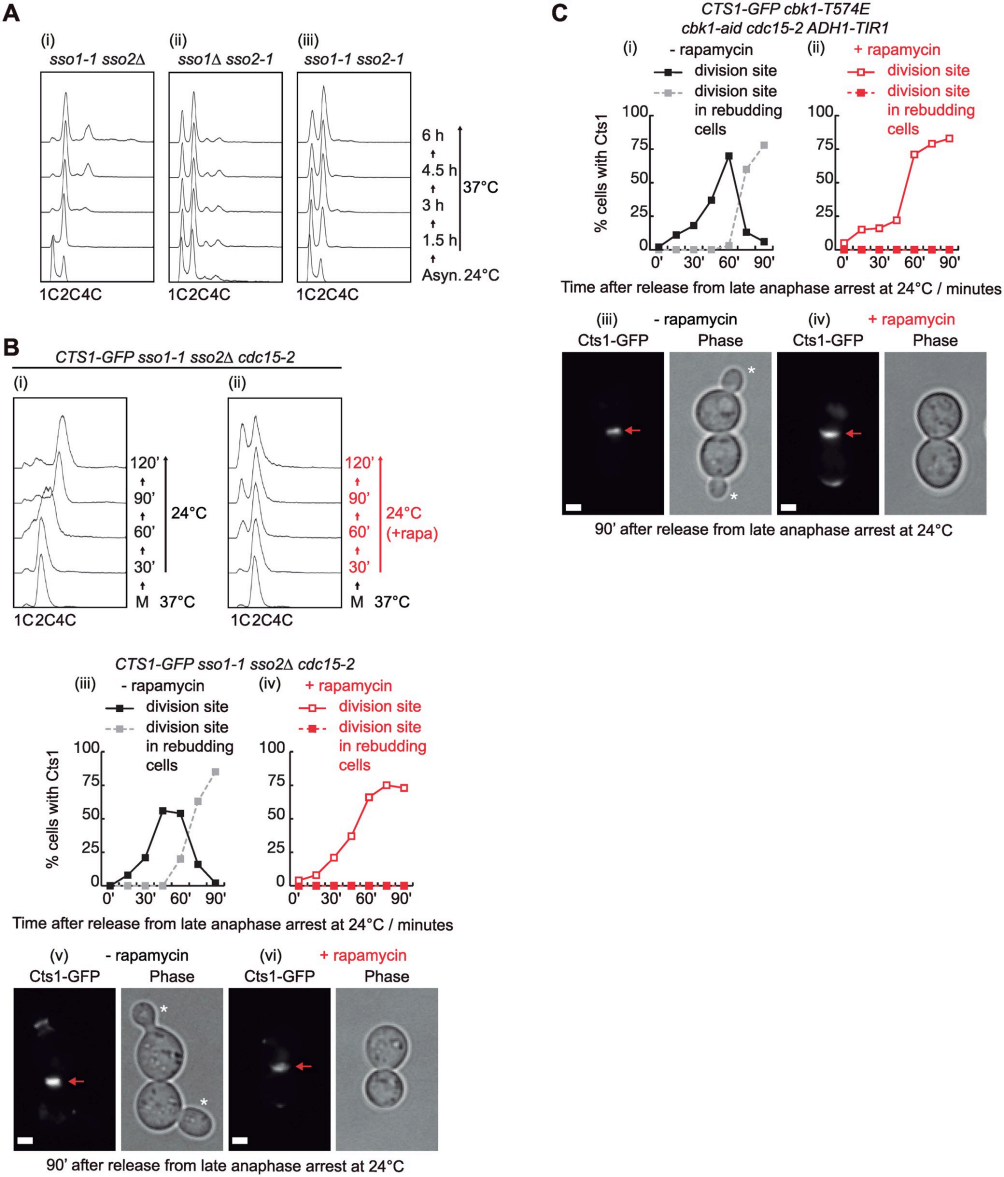
A t-SNARE mutant and phosphomimetic Cbk1-T574E show a cell separation defect despite Cts1 accumulation. (A) *sso1-1 sso2Δ* (H1239) (i), *sso1Δ sso2-1* (H603) (ii), and *sso1-1 sso2-1* cells (H1269) (iii) were grown in YPD medium asynchronously at 24 °C before raising the temperature to 37 °C. Samples were taken at the specified times and fixed with 70% ethanol before analysing them by flow cytometry to follow cell-cycle progression. (B) *CTS1-GFPEnvy sso1-1 sso2Δ cdc15-2* (YMF4218) cells were grown in YPD and arrested in late anaphase by shifting the temperature to 37 °C. Rapamycin was added to half of the culture as indicated and cells were release at 24 °C in the absence (i) or presence (ii) of rapamycin. Using fluorescence microscopy, we studied the proportion of cells with Cts1 at the site of division site in the absence (iii) or presence (iv) of rapamycin. Examples of cells are shown at 90 min after the release at 24 °C in the absence (v) or presence (vi) of rapamycin. Red arrows denote the original division site and new buds are marked with white asterisks. Scale bar indicates 2 μm. (C) *CTS1-GFPEnvy cbk1-T574E cbk1-aid cdc15-2* (YMF4250) cells were grown in YPD and arrested in late anaphase by shifting the temperature to 37 °C before the addition of NAA and IAA auxins. Next, cells were incubated with DMSO or rapamycin for 20 min before they were released in the absence or presence of rapamycin (NAA and IAA auxins were present in medium until the end of the experiment for both conditions). Samples were taken at the indicated times to be analysed by fluorescence microscopy and determine the proportion of cells with Cts1 at the site of division in the absence (i) or presence (ii) of rapamycin. Examples of cells are shown at 90 min after the release at 24 °C in the absence (iii) or presence (iv) of rapamycin. The asterisks indicate new buds and red arrows show the original division site. Scale bar indicates 2 μm. Underlying data for all the graphs can be found in S1 Data file. FACS graphs can be found in the supplementary FACS file (S1 File).

SNARE complex promotes vesicle-plasma membrane fusion at the site of division. To determine whether cell separation defect in *cdc15-2* cells is due to a problem in membrane fusion (Fig 5A(iii)), we used SNARE mutants. Indeed, it has been reported that budding yeast t-SNARE *sso1* and *sso2* mutant cells have a defect in cell separation, suggesting a failure in the delivery of the enzymes that promote separation between mother and daughter cells [53]. We grew previously described mutants, *sso1-1 sso2Δ*, *sso1Δ sso2-1*, and *sso1-1 sso2-1* [53], to find out if any of them might have a defect consistent with cell separation failure at the restrictive temperature (Fig 6A). Only *sso1-1 sso2Δ* cells accumulated with 2C DNA content, and a small proportion of the population with 4C DNA content too (Fig 6A(i)). Subsequently, to study whether inactivation of TORC1 was able to rescue cell separation defect after anaphase arrest and release in *cdc15-2* cells, we grew *CTS1-GFP sso1-1 sso2Δ cdc15-2* cells as previously described (Fig 2A). We showed that there were cells still defective, as part of them accumulated with 2C DNA content, after the addition of rapamycin (Fig 6B(ii)). This is different from what occurred in *cdc15-2* cells with functional membrane fusion system (Fig 2A(ii)). Furthermore, in the presence of rapamycin cell separation is defective despite Cts1-GFP accumulation at the site of division (Fig 6B(iv) and 6B(vi)), unlike what rapamycin promoted in *cdc15-2* cells: Cts1-GFP localization at the site of division and successful cell separation (compare Figs 5F(ii) and 6B(iv)). Absence of rapamycin treatment in *sso1-1 sso2Δ cdc15-2* cells prompted sustained Cts1-GFP localization at the site of division and cell separation defect for cells that were able to continue their cell cycles and re-bud again, like we observed for *cdc15-2* cells if rapamycin was not added (compare Figs 5F(i), 5F(iii), 5F(v)), 6B(iii) and 6B(v). Therefore, compromising fusion of secretory vesicles into plasma membrane blocks the rescue of cell separation defect. Finally, cells expressing the phosphomimetic Cbk1-T574E, which reproduces negative effect of TORC1 control over cell separation, accumulated Cts1-GFP at the site of division after the release from anaphase block, followed by rapamycin addition, in a similar manner to *sso1-1 sso2Δ cdc15-2* cells (compare Fig 6C(ii) and 6C(iv) with Fig 6B(iv) and 6B(vi)). These cells were defective in cell separation although they were able to transport Cts1 to the site of division. Taken together, these findings would suggest that the cell separation defect associated with *cdc15-2* cells after anaphase block and release is due to a failure in membrane fusion at the site of division and Cbk1 plays a key role in that process.

### Phosphomimetic Cbk1-T574E is catalytically inactive and unable to phosphorylate a novel substrate, the exocyst component Sec3

**Fig 7:**
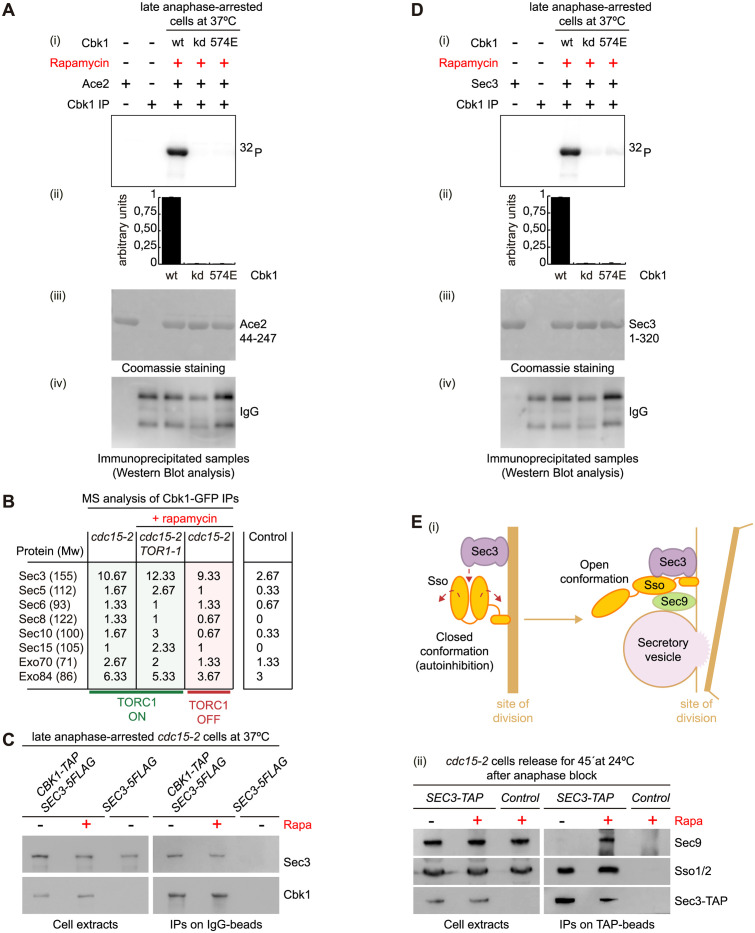
Cbk1 binds to and phosphorylates exocyst component Sec3. (A) Protein extracts were prepared for *CBK1-5FLAG cbk1-aid cdc15-2* (YMF4113), *cbk1-D475A-5FLAG cbk1-aid cdc15-2* (YMF4180), and *cbk1-T574E-5FLAG cbk1-aid cdc15-2* strains (YMF4115). Cells were arrested in late anaphase after having grown at 37 °C. To block TORC1 activity, rapamycin was added for 20 min as indicated. Cbk1-5FLAG was purified by immunoprecipitation using the M2 anti-FLAG monoclonal antibody. The kinase activity of immunopurified Cbk1 was measured as described in Materials and methods. Kinase assay is shown in (i). Measurement of the ^32^P-Ace2-44-247 signal was performed (ii). Quantification of the specific Cbk1 activity was done by normalising the phosphorylation signal to the amount of recombinant Ace2 (iii) and immunopurified Cbk1 (iv). Three experimental replicates were performed. (B) Sec3 peptides were found in mass spectrometry of immunoprecipitation of Cbk1-GFP on ChromoTek GFP-Trap Magnetic beads. Spectral counts for Sec3 were higher than for any other exocyst proteins. Spectral average for 3 different conditions is shown (see S3 Table for more details). (C) *CBK1-TAP SEC3-5FLAG cdc15-2* (YMF4238) and control cells (YMF4240) were grown in YPD and arrested in late anaphase by shifting the temperature to 37 °C, before the addition of rapamycin for 20 min when indicated. Subsequently, protein extracts were prepared and immunoprecipitation of Cbk1 on IgG beads was performed before the detection of the indicated proteins by immunoblotting. (D) *CBK1-5FLAG cbk1-aid cdc15-2* (YMF4113), *cbk1-D475A-5FLAG cbk1-aid cdc15-2* (YMF4180), and *cbk1-T574E-5FLAG cbk1-aid cdc15-2* cells (YMF4115) were grown as described in A. M2 anti-FLAG monoclonal antibody was used to immunoprecipitated Cbk1-5FLAG. A kinase assay (i) was performed with immunopurified Cbk1 from yeast cell extract (iv) and bacterial-expressed and purified His6-MBP-Sec3-1-320 as substrate (iii). Quantification of the specific Cbk1 activity (ii) was done by normalising the phosphorylation signal to the amount of recombinant Sec3 (iii) and immunopurified Cbk1 (iv). Three experimental replicates were performed. (E) Schematic representation of how Sec3 induces conformational change for Sso (autoinhibitory closed conformation to open), allowing the interaction between Sso and Sec9 (i). (ii) *SEC3-TAP cdc15-2* (YMF4221) and control cells (YMF3546) were grown in YPD and arrested in late anaphase by shifting the temperature to 37 °C. Rapamycin was added 20 min before release (+), and 45 min after release, protein extracts were prepared and immunoprecipitation of Sec3 on TAP beads was performed. Detection of the indicated proteins was carried out by immunoblotting using specifically raised sera. Underlying data for all the graphs can be found in S1 Data file. Raw data for blots can be found in supporting information (S5 Raw Images). TOR, target of rapamycin.

To understand further the underlying molecular mechanism by which TORC1 regulates cell separation, we studied whether TORC1 controls Cbk1 kinase activity. We performed an in vitro kinase assay using immunoprecipitated Cbk1 (Fig 7A(iv)) from anaphase-arrested *cbk1-aid cdc15-2* cells in the presence of rapamycin expressing, under its own promoter, either Cbk1 (wt), the catalytically inactive Cbk1-D475A (kd, kinase dead [39]) and Cbk1-T574E mutant that is able to reproduce the cell separation defect associated with TORC1 activity as we described above (Fig 7A(i) and 7A(ii)). A truncated version of Ace2 (Ace2-44-247) was expressed, purified and used as substrate (Fig 7A(iii)). Interestingly, Cbk1-T574E had kinase activity levels similar to the catalytically inactive Cbk1-D475A (Fig 7A(i) and 7A(ii)), suggesting that TORC1 might promote inactivation of Cbk1 kinase activity.

To identify key proteins that could explain the cell separation defect, we analysed the immunoprecipitated material associated with Cbk1-GFP in the experiment described above for Tables 1 and S1. Most of known interactions were maintained similarly in all 3 conditions, independently of TORC1 activity (see S8A Fig and its legend for more details and S2 Table). Interestingly, mass spectrometry analysis found that the number of spectral counts for some members of TORC1 was higher than in the untagged control, especially in *cdc15-2* cells in the absence of rapamycin (see Lst8, Tor1, and Kog1 in S8C Fig and S3 Table).

As cell separation defect in *cdc15-2* cells seemed to be in membrane fusion at the site of division (Fig 6), we focused our attention to find proteins involved in such cellular process. Interestingly, it has been reported that the orthologue of Cbk1 in fission yeast, the protein Orb6, positively regulates exocytosis phosphorylating the orthologue of Sec3 (budding and fission yeast share the same name for this protein) [54]. The exocyst complex mediates the tethering of secretory vesicles to the plasma membrane and activates the SNARE complex to promote membrane fusion [51,52]. Target SNAREs (t-SNARE) are located on the target plasma membrane and interact with v-SNARE situated on the secretory vesicle membrane, which promotes membrane fusion [55]. Therefore, we searched for exocyst components in the immunoprecipitated material from the experiment initially described above for Tables 1 and S1. We found that the spectral count average for Sec3 was higher than for any other exocyst proteins (Fig 7B and S3 Table). This interaction was confirmed subsequently by immunoprecipitation and immunoblotting of anaphase-arrested cells in the absence or presence of rapamycin (Fig 7C). Mass spectrometry analysis was unable to find Cbk1 interacting with SNARE components that promote fusion of secretory vesicle at the plasma membrane (S3 Table). Therefore, our findings would suggest that Sec3 might be a novel substrate of Cbk1 in budding yeast. Scanning Sec3 protein sequence, we located 3 fully conserved NDR/LATS consensus sites at its N-terminus (S18, S32, and S66) and 1 site (S43) only conserved in *Saccharomyces* species (S9A Fig). There are evidences of in vivo phosphorylation for S18, S32, and S43 in phosphoproteomic analysis [56] and, interestingly, sites S32 and S43 have been described to be rapamycin sensitive (https://thebiogrid.org/36739/summary/saccharomyces-cerevisiae/sec3.html) [44,45]. Then, we investigated whether Cbk1 was able to phosphorylate Sec3 (Fig 7D) as we described above (Fig 7A). We found that immunoprecipitated Cbk1 from anaphase-arrested *cdc15-2* cells in the presence of rapamycin phosphorylated the N-terminus of Sec3. We showed that catalytically inactive Cbk1-D475A and Cbk1-T574E, which mimicked TORC1 phosphorylation, were unable to phosphorylate Sec3 (Fig 7D(i) and 7D(ii)). These findings would indicate that Cbk1 controls Sec3 by phosphorylation of its N-terminus, precisely the same end of Sec3 protein that has been described to interact and regulate t-SNARE protein Sso2 to induce membrane fusion [57,58]. The exocyst component Sec3 binds to the t-SNARE protein Sso2 to release it from its autoinhibition conformation and promotes the interaction of Sso2 with the t-SNARE protein Sec9 to induce membrane fusion [57,58]. TORC1, via regulating Cbk1 phosphorylation, might block that step, precisely, to prevent cell separation before mitosis.

### TORC1 activity ultimately alters the dynamics between the exocyst component Sec3 and the SNARE complex

To understand further the underlying molecular mechanism by which TORC1 regulates cell separation, we investigated whether interactions between the exocyst Sec3 and the SNARE components varied in our experimental system. Sec3 binds to t-SNARE Sso2, releases Sso2 from autoinhibition closed conformation to an open conformation, allowing Sso2 to interact with t-SNARE Sec9, which finally promote secretory vesicle fusion into the plasma membrane [55] (Fig 7E(i)). We grew *SEC3-TAP cdc15-2* cells, arrested them at 37 °C and subsequently released them at 24 °C for 45 min in the presence or absence of rapamycin to follow Sec3 interaction with SNARE components (Fig 7E(ii)). We immunoprecipitated Sec3 and determined that Sec3-TAP was able to bind to Sso1/Sso2 independently whether TORC1 was active (- rapamycin) or inactive (+ rapamycin) (Fig 7E(ii)). However, Sec3 interaction with the SNARE component Sec9 was clearly compromised when TORC1 was active (Fig 7E(ii),—rapamycin), which is precisely when *cdc15-2* cells showed a cell separation defect (Fig 2A). Taken all together, our data would suggest that TORC1 might ultimately block the formation of functional SNARE complex that would promote the fusion of secretory vesicle transporting hydrolases to the plasma membrane. TORC1 might prevent exocyst Sec3 to release Sso from its autoinhibitory conformation and provoke a cell separation failure.

## Discussion

**Fig 8:**
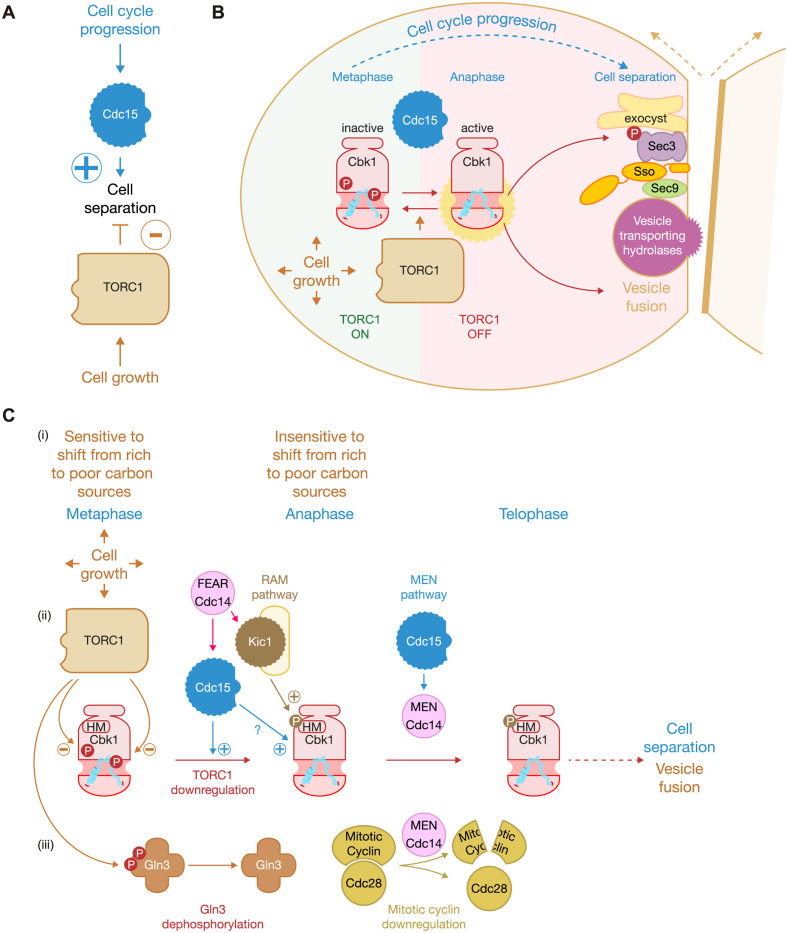
Proposed model for how TORC1 regulates Cbk1 activity. (A) Mitotic kinase Cdc15 promotes cell separation while TORC1 negatively controls cell separation. (B) Interplay between Cdc15 and TORC1 regulates kinase activity associated with Cbk1. TORC1 is active until anaphase, which promotes cell growth and inhibits cell separation. Cdc15 becomes active in anaphase and releases Cbk1 from TORC1-driven inhibition. At this point, Cbk1 turns catalytically active and would eventually regulate phosphorylation of key substrates that promote membrane fusion at the site of division. One of those substrates is Sec3 that induces the interaction between t-SNARE components Sso1/2 and Sec9 to drive the fusion of secretory vesicles into the plasma membrane. Finally, hydrolases contained in secretory vesicles are released to promote cell separation. (C) Proposed model for how the conserved NDR kinase Cbk1 is controlled by TORC1 and Cdc15 during mitosis in budding yeast. (i) Metaphase cells are able to delay mitosis if they are shifted from rich to poor carbon sources causes, whereas anaphase cells seem to be insensitive to that change in carbon sources [63]. (ii) Kinase activity associated with TORC1 negatively regulates Cbk1, while Cdc15, FEAR Cdc14, and the RAM pathway promote Cbk1 activity. Cdc15 and Cdc14 are components of the MEN pathway that is essential for cell to exit mitosis. Cdc14 is the most downstream factor of MEN. The role of Cdc15 to control cell separation is different from its function at the anaphase to telophase transition. (iii) Gln3 (a TORC1 substrate) and Mitotic cyclin (Clb2) are included to depicted timing of events during mitosis: dephosphorylation of Gln3 occurred when percentage of anaphase cells peaked, whereas down-regulation of mitotic cyclins (Clb2) drives the transition between anaphase to telophase and takes place slightly later (experimental data for Gln3 and Clb2 are included in Fig 1D). MEN, mitotic exit network; NDR, nuclear Dbf2-related; RAM, regulation of Ace2 and morphogenesis; TOR, target of rapamycin.

Our data highlight a novel role for the TOR signalling pathway to negatively regulate cell separation in budding yeast that is counteracted by the kinase Cdc15, which is a Mst/hippo like kinase (Fig 8A). In addition to the role of TORC1 in cell growth, it has been shown that TORC1 promotes early steps in the cell cycle like G1 progression and transition into S-phase [59–61]. Later in the cell cycle, TORC1 drives mitosis progression by stabilising mRNA of mitotic cyclin Clb2 [13]. Besides, TORC1 controls other aspects of mitosis in budding yeast as it regulates nuclear localization and activation of the polo-kinase Cdc5, structure and function of microtubules, and mitotic spindle assembly [21,22,62].

We described a novel mechanism by which TORC1 blocks cell separation between mother and daughter cells (Fig 8A and 8B). The mitotic kinase Cdc15 prevents that inhibition and allows cells to separate and start a new cell cycle apart from each other (Fig 8), which explains why inactivation of TORC1, by the addition of rapamycin, rescues unexpectedly cell separation defect in *cdc15-2* cells (S10 Fig). Besides, it explains why cells do not show a cell separation defect if Cdc15 is active after metaphase block and release (Fig 2C). Critical cell cycle transitions are prolonged depending on cell size, which is regulated in response to environmental cues [64]. Shift from rich to poor carbon sources causes prolonged metaphase delay, whereas anaphase cells seem to be insensitive as they are unable to delay mitosis [63] (Fig 8C(i)). Interestingly, we found that dephosphorylation of a TORC1 substrate, Gln3, occurs in anaphase before cells down-regulate mitotic cyclin Clb2 at the anaphase to telophase transition (Figs 1D and 8C(iii)). In addition, inactivation of TORC1 in G2-M arrested cells drove Gln3 dephosphorylation (S1A(ii) Fig), which shows again that Gln3 dephosphorylation we observed before Clb2 depletion depends on TORC1 down-regulation (Figs 1D and 8C(iii)). Therefore, anaphase cells might inactivate the mechanism by which cells coordinate cell growth and cell cycle progression, which would agree with our findings. Functional connections between mTOR and Hippo pathways have been described in mammalian cells in different context other than cell separation [65–68]. Future work will need to determine whether Cdc15 might phosphorylate cell separation substrates that would counteract TORC1 inhibitory phosphorylation (Fig 8B and 8C). This novel function of Cdc15 is different from its well-described role in MEN pathway to release Cdc14 from the nucleolus and drive exit from mitosis as it occurred at 2 distinct moments at the end of the cell cycle (Fig 8C).

TORC1 binds to the NDR kinase Cbk1, controlling and participating in its phosphorylation (Figs 3 and 8). Our data would suggest that TORC1 blocks Cbk1 activity before the onset of anaphase and cells are unable to initiate cell separation (Fig 8B and 8C). At anaphase, FEAR release of Cdc14 dephosphorylates and activates Cdc15 (Fig 8C(ii)), stimulating its ability to promote mitotic exit too [30,69]. Before the end of anaphase, activated Cdc15 would release Cbk1 from TORC1-induced inhibition (Fig 8B and 8C(iii)). Interestingly, Cdc15 has been described to phosphorylate 2 key regulatory phosphorylation sites on the NDR kinase Dbf2, the most downstream kinase in MEN pathway [70]. Mammalian NDR kinases also contain 2 key residues that are required to be phosphorylated [71]. Dbf2 shares structural similarities with Cbk1 and those 2 important residues are conserved [72]. The RAM cascade is required to phosphorylate one of those residues located at the C-terminal HM of Cbk1 (T743), whereas autophosphorylation of the other residue located at the activation loop (S570) is important for Cbk1’s kinase activity [39]. Future experiments should determine how Cdc15 might contribute to Cbk1 activation to counteract TORC1 negative control. On the other hand, FEAR Cdc14 promotes precisely the phosphorylation of Cbk1’s C-terminal HM by RAM pathway (Fig 8C(ii)). Both Cdc15-driven release of TORC1 block and RAM phosphorylation on HM would activate Cbk1 kinase activity in anaphase, which would promote the following cell separation (Fig 8C(ii)). This is a novel mechanism by which cells order the sequence of different cell-cycle steps and coordinate cell cycle with cell growth. In anaphase-arrested cells, the phosphorylation profile of Cbk1 varies depending on TORC1 kinase activity (Figs 3E and S10 and Table 1). TORC1-regulated phosphorylation on Cbk1 must inhibit Cbk1 function in anaphase, which blocks the following cell separation, even though Cbk1 could be dephosphorylated after anaphase. Therefore, Cbk1-dependent phosphorylation of key substrates must occur in anaphase (Fig 8). Strikingly, a single mutation on a conserved residue of Cbk1 activation loop, T574E, is enough to reproduce the cell separation defect in the presence of rapamycin (Fig 4D). It is likely that our mass spectrometry analysis was unable to identify residue T574 as a phosphosite (Tables 1 and S1) since the corresponding peptide generated using trypsin digestion was too large. We found that Cbk1-T574E is catalytically inactive, suggesting that is precisely how TORC1 might negatively control cell separation: inactivation of Cbk1 kinase activity. An alanine substitution on T574 was unable to rescue cell separation defect in the absence of rapamycin, which suggests that TORC1 must participate in the phosphorylation of other residues within Cbk1 or other proteins. In fact, mass spectrometry found changes in the phosphorylation pattern of some key Cbk1 regulators (Fir1, Lre1, Mob2, or Tao3) and some Cbk1 substrates (Ace2 and Ssd1). Indeed, TORC1 and Ssd1 have been described to collaborate to maintain cellular integrity [73]. We managed to find mutants that reproduced TORC1 block on cell separation. However, the generation of non-phosphorylatable mutants that would be able to avoid TORC1 control has turned out challenging as we would have needed to identify all residues regulated by TORC1 within Cbk1 and other proteins.

Since our findings suggested that the cell separation defect associated with *cdc15-2* cells was due to a block in secretory vesicle fusion into the plasma membrane (Fig 8B and 8C(ii)), we searched for proteins involved in that cellular process to understand the molecular mechanism behind TORC1 control. Interestingly, it has been recently suggested a role for Cbk1 in vesicle transport and/or membrane fusion [5]. We determined that Cbk1 binds, controls, and participates in the phosphorylation of the exocyst component Sec3 (Fig 7), as in the fission yeast *Schizosaccharomyces pombe* [54]. Cbk1 phosphorylation consensus sequences were described in Sec3 [48], whose phosphorylation has been described to be rapamycin sensitive [44,45]. Our data would suggest that Cbk1 phosphorylation on Sec3 must occur in anaphase as discussed above and, if TORC1 blocks Cbk1 kinase activity, Sec3 phosphorylation would be defective and mother and daughter cells would be unable to separate (Figs 8 and S10). Sec3 is essential for the tethering of secretory vesicles at the site of division [51,52]. In addition, Sec3 binds to t-SNARE Sso2, releasing Sso2 from an autoinhibition conformation to subsequently induce Sso2 to interact with t-SNARE Sec9 and finally induce membrane fusion [57,58] (Fig 7E(i)). Actually, the exocyst promotes the assembly of SNARE complex and subsequently vesicle fusion (https://doi.org/10.1101/2022.01.16.476540). Cbk1 phosphorylation on Sec3 (Fig 7D) might regulate Sso2 function and its ability to induce membrane fusion via Sec9. In fact, we found that Sec3 interaction with Sec9 was compromised in *cdc15-2* cells in which TORC1 was active after late anaphase block and release (Fig 7E(ii)). We showed that TORC1 regulates and participates in the phosphorylation of Cbk1, which would become catalytically inactive. In turn, Sec3 phosphorylation would be defective, which would alter the function of the t-SNARE complex and prevent membrane fusion, at the site of division, of secretory vesicle transporting hydrolases like Cts1. We generated a phosphomimetic version of Sec3 (Sec3-4E; S18E, S32E, S43E, and S66E), but we were unable to test it as *sec3-aid ADH-TIR1* cells were able to grow under restrictive conditions at 24 °C, which showed that Sec3 depletion was not completely effective (S9B Fig). This prevented us from the use of *sec3-aid* mutant, in combination with *cdc15-2*, to inactivate Cdc15 function at 37 °C and test whether Sec3-4E was able to rescue cell separation defects associated to *cdc15-2* cells. Furthermore, deletion of the first 146 amino acids of Sso1 released the protein from its autoinhibition conformation. Therefore, we tried to investigate whether such deletion might rescue cell separation defects in *cdc15-2* cells. Unfortunately, adding 5xFLAG to the N-terminal end of Sso1 makes the protein unfunctional as *sso2Δ* cells carrying that form of Sso1 are dead (S9C Fig).

Interestingly, Cbk1 binds to paralogs serine/threonine kinases Kin1 and Kin2 (S8A Fig and S2 Table) [74,75], Kin2 is phosphorylated when TORC1 is inactive (Table 1) and, presumably, Cbk1 is active, consistent with our data. Both budding yeast Kin1 and Kin2 kinases regulate late stages of exocytosis via phosphorylation of t-SNARE Sec9 [76,77], suggesting that Cbk1 role in the regulation of membrane fusion might also target Sec9 via Kin1 and Kin2. In addition, the fission yeast ortholog of Cbk1, the protein Orb6, was found also to bind to the kinase Ppk25 whose kinase domain is most similar to Kin1 and Kin2 [54].

Cell cycle machinery guarantees the temporal control of the different steps during the cell cycle. CDK activity drives progression throughout the cell cycle until the end of mitosis and, at the same time, blocks late events during the cell cycle to assure the orderly sequence of events [1–3]. Mitotic CDK blocks cell separation by direct phosphorylation of Ace2 and Cbk1, inhibiting Ace2 nuclear localization. Consequently, key hydrolases involved in cell separation are not expressed. To allow cells to end the cell cycle and start a new one, cells need to inactivate kinase activity associated with CDK, for which MEN signalling pathway plays a key role, especially the phosphatase Cdc14 as the downstream factor of MEN [33,78,79] (Fig 8C). Cdc14 is required to dephosphorylate both Ace2 and Cbk1 and promote cell separation [4]. However, our experimental system allowed us to study specifically TORC1 control over cell separation. This is independent of CDK function over Ace2 and Cbk1, since late anaphase-arrested *cdc15-2* cells accumulated hydrolases like Cts1 and Dse4, even when Ace2 and Cbk1 were depleted (Fig 5B and 5C) and kinase activity associated with CDK is still high in late anaphase. Therefore, Cbk1 induces Cts1 secretion independently of Cbk1 role in promoting Cts1 expression. Our data suggested a role for Cbk1 in membrane fusion at the site of division by regulating the function of exocyst Sec3 and t-SNARE components (Fig 8).

The strategy by which NDR/LATS kinases control vesicle transport and membrane fusion is likely conserved in *S*. *cerevisiae* and *S*. *pombe*, as both yeast cells share the phosphorylation of Sec3 as a target (Fig 7 and [54]). Interestingly, fission yeast exocyst mutants show cell separation defects [80], the fission yeast ortholog of Cbk1, Orb6, regulates exocytosis and it has been proposed that phosphorylation of Sec3 by Orb6, together with exocyst component Exo70, promotes exocytosis to induce cell separation [54]. Furthermore, mammalian NDR1/2 kinases also control vesicle-mediated trafficking and exocytosis [81,82], indicating a universal mechanism for NDR/LATS kinases to regulate cell polarity and exocytosis. In addition, Cbk1 binds and phosphorylates Sec2, essential for post-Golgi vesicle transport [83], although we do not detect any defect in vesicle transport to the site of division as Cts1 is able to localise when Cbk1 activity is compromised (Figs 5F and 6C).

TORC1 plays a key role in the regulation of different steps of the cell cycle among eukaryotes, which suggest a universal mechanism too. Fission yeast cells couple the cell cycle with the nutritional environment [84]. In mammalian cells, TORC1 controls different aspects of the cell cycle, including mitosis [85,86]. Interestingly, it has been reported very recently that in mouse and human cells mTORC1 substrate, S6 Kinase 1, phosphorylates Cdk1, inducing a G2/M cell-cycle arrest to promote DNA repair [87]. TOR activity, Mst/hippo signalling pathways, exocyst, and SNARE complexes are highly conserved in eukaryotes. Defects in the molecular mechanisms they are involved have been described in a variety of human diseases, such as cancer and diabetes. The elucidation of the mechanisms and their coordination will be important to understand human pathology.

## Methods

### Growth of yeast strains

The budding yeast *S*. *cerevisiae* strains that were used in this study were all based on W303 and are listed in S4 Table. Cells were grown in rich medium containing 1% yeast extract, 2% peptone and supplemented with 2% glucose (YPD) unless marked otherwise. For all synchronisation experiments, asynchronous cultures of cells were grown overnight and the following morning cells were counted and diluted to a concentration of 4 × 10^6^ cells/ml before allowing them to grow to a density of 7 × 10^6^ cells/ml. To arrest cells in the G1 phase of the cell cycle, the mating pheromone α-factor (Pepceuticals) was added to a final concentration of 7.5 μg/ml. After 2 h, additional 2.5 μg/ml aliquots of α-factor were added every 20 min and cells were checked using phase contrast microscopy until at least 90% of cells were unbudded. To release cells synchronously from G1 arrest, cells were pelleted, washed twice, and released into fresh medium. To synchronise *cdc15-2* cells in late anaphase, cells were grown at 37 °C until at least 90% of cells were large-budded. To release cells synchronously from late anaphase arrest, cells were pelleted and released into fresh medium at 24 °C in the absence or presence of rapamycin. For experiments with rapamycin, cells were arrested in late anaphase and rapamycin (Sigma, R0395, dissolved in DMSO) was added to a final concentration of 200 nM for 20 min. Control cells were incubated with DMSO (Sigma, D8418) instead of rapamycin.

For experiments with *cbk1-aid* allele (“aid” denotes “auxin inducible degron” system), cells were arrested at late anaphase and IAA (Sigma, I3750) and NAA (Sigma, 317918) auxins, dissolved in ethanol, were added to a final concentration of 500 μm each to allow Cbk1 depletion for 50 min. Next, rapamycin or DMSO were added for another 20 min at 37 °C. To release cells synchronously from late anaphase arrest *cbk1-aid* cells were spun down and resuspended into fresh medium at 24 °C in the presence of IAA and NAA auxins. For experiments with *GAL-CDC20* (Fig 2C), cells were grown overnight in medium containing 2% Raffinose and 2% Galactose. To synchronise cells in metaphase cells were filtered and resuspended in fresh medium containing 2% Raffinose and 0.01% Glucose for 3 h before the addition of rapamycin or DMSO for 20 min and subsequent release from the metaphase arrest by addition of 2% of Galactose.

### Plasmids and cloning

S5 Table lists all plasmids used in this study. The sequence of *cbk1-6E*, including its own promoter and 3′ UTR was subcloned from centromeric plasmid pELW889 (pRS316-*cbk1-6E*) [42] into integrative plasmid pRS306 [88]. Gibson assembly [89] was performed to generate a collection of Cbk1 mutants, together with wt *CBK1* control, using plasmid pRS306-*cbk1-6E* as template and oligonucleotides listed in S6 Table before the final confirmation of the mutations by DNA sequencing. The resulting plasmids were linearized and then transformed into *cbk1-aid* or *cbk1Δ* cells to integrate them into yeast genome. Confirmation of the genome modification was then performed by a series of PCR reactions. The DNA sequence encoding the N-terminal part of Sec3 (residues 1–320) was cloned into the vector pET28 with a TEV cleavable N-terminal 6x-His maltose binding protein (MBP) tag. The expression plasmid for Ace2 (residues 44–247) pMAL-C2x-MBP-Ace2_44-247_ was kindly provided by Dr. Gislene Pereira.

### Flow cytometry and binucleated cell analysis

The content of DNA in cells was monitored by flow cytometry of cells fixed with 70% ethanol and stained with propidium iodide as previously described [90,91]. Flow cytometer used in this study was CytoFLEX (Beckman Coulter). A total of 10,000 cells from each sample were recorded. The gain was adjusted with each asynchronous sample matching 1C population to 50,000 fluorescence intensity units. All cells analysed by flow cytometry were haploid cells, so 1C DNA content corresponds to cells at the G1 phase of the cell cycle. Samples were recorded at slow speed for best resolution and the channel used to measure propidium iodide signal was 585/42 band pass. Data were analysed with Kaluza software (Beckman Coulter). Gating strategy was based on debris clearing using an SSv-vs-PI dot plot and then a histogram overlay (3440–45000 fluorescence intensity range). For binucleated cell analysis, cells were fixed in the same way as described above for flow cytometry with 70% ethanol. Subsequently, cells were stained with Hoechst 33342 (Invitrogen, H21492) and for each time point 100 cells were examined. Binucleated cell analysis was performed with a Nikon A1R Microscope and an Orca R2 camera (Hamamatsu) with objective lens Plan Apo TIRF 100× oil DIC 1.49NA.

### Microscopy

To observe GFP-tagged proteins cells were either fixed with 8% formaldehyde for 10 min and subsequently washed twice with ice-cold PBS or observed live [33]. Phase contrast and fluorescence microscopy images of cells grown in liquid culture were performed with a Nikon A1R Microscope and an Orca R2 camera (Hamamatsu) with objective lens Plan Apo TIRF 100× oil DIC 1.49NA and Nikon 60× PlanApo VC 1.4 N.A., and LightLine single-band filter set FITC Semrock. The illumination source was the Nikon Intensilight C-HGFIE (ultrahigh Pressure 130W Mercury lamp), and we used NIS elements software. In addition, a Zeiss Axio Observer.Z1 microscope, with Zeiss oil immersion 63× and 100× objectives, and a Photometrics QuantEM CCD camera were used. In all experiments, exposure time, sensor gain, and digital adjustments were the same for minus and plus rapamycin experimental samples. The microscopy data were deconvolved using Huygens (SVI) according to the “Quick Maximum Likelihood Estimation” method and a measured point spread function. The deconvolved data set was viewed with ImageJ software [92] (National Institute of Health, United States of America). We examined 100 cells for each time point. Each experiment was performed at least twice. We analysed 9 z-sections with a spacing of 0.375 μm with the exception of RAS2-GFP experiments where 17 z-sections were analysed with a spacing of 0.2 μm to facilitate the examination of whole cells in all experiments.

### Cell lysis, immunoprecipitation from yeast cell extracts, and immunoblotting

To monitor the association of proteins in yeast cell extracts, we used 250 ml samples (2^10^ cells). Frozen cell pellets were ground in the presence of liquid nitrogen, using a SPEX SamplePrep LLC 6850 freezer/mill as described previously [93,94]. We isolated tagged proteins by immunoprecipitation with magnetic Dynabeads M-270 Epoxy (Invitrogen, 14301) coupled at 4 °C to rabbit anti-sheep IgG (Sigma, S-1265) or M2 anti-FLAG monoclonal antibody (Sigma, F3165) as described previously [93,94]. Protein samples were resolved by SDS-PAGE on NuPAGE Novex 4% to 12% or 8% Bis-Tris gels (Thermo Fisher Scientific) with NuPAGE MOPS SDS Running buffer (Thermo Fisher Scientific) or homemade 8% SDS-PAGE gels based on 30%, 37.5:1 Protogel. The resolved proteins were transferred onto a nitrocellulose membrane with the Trans-Blot SD Semi-Dry Transfer Cell (Bio-Rad). We detected the indicated proteins by immunoblotting by using polyclonal anti-FLAG antibody (Sigma, F-7425), M2 anti-FLAG monoclonal antibody (Sigma, F3165), monoclonal 9E10 (anti-MYC), anti-HA antibody (Sigma, H-3663), rabbit anti-sheep IgG (Sigma S-1265), or phospho-Rps6 (Ser235/236) antibody (Cell Signaling Technology, 2211). Crude anti sera raised against Sso1 (also recognised Sso2) and sera against Sec9 were used to identify components of the SNARE complex. Sera for SNARE proteins were kindly provided by Dr. Brennwald. Detection was carried out using homemade ECL Western Blotting Detection Reagents. The chemiluminescent signal was developed with an Amersham ImageQuant 800 Western blot imaging system (Cytiva). Protein quantification for Fig 1D and 1E was performed using Image J software. Protein shift mobility of Gln3 after addition of rapamycin was set as hypophosphorylated Gln3 (Fig 1E(i)).

### Whole-cell protein extraction

Protein extracts were obtained by the trichloride acetic acid (TCA) method. Collected cells were fixed with cold 20% TCA overnight at 4 °C. Cells were washed with 1 M Tris base and resuspended in loading buffer (100 mM Tris-HCl (pH 6.8), 0.2 M DTT, 4% glycerol, 0.002% bromophenol blue) at 95 °C. Then, 1 volume of glass beads (425 to 600 μm of diameter) were added and cells were broken by mechanical lysis with a disrupter (Precellys 24 homogenizer; Bertin Technologies) for 6 cycles at 5,000 rpm. Protein extracts were clarified at 12,000 rpm for 5 min and stored at −20 °C. For western blot analysis, samples were boiled at 95 °C for 5 min, centrifuged 5 min at maximum speed, and loaded to protein gels. Protein samples were resolved by SDS-PAGE on homemade 8% gels. After protein separation by electrophoresis, proteins were transferred to PVDF membranes (Immobilon PVDF, Millipore, IPVH00010) previously activated with 100% methanol. Membranes were blocked with PBS-Tween (PBS, 0.02% Tween-20, A4974) containing 5% milk (Panreac, A0830). Protein membranes were incubated with the α-FLAG clone M2 antibody (Sigma, F3165) for 2 h and with the α-Mouse (1:20,000) secondary antibody (Cytiva, NXA931) for 60 min. After each antibody incubation, membranes were washed 3 times with PBS-Tween-20. For the chemiluminescent detection, membranes were incubated with the Supersignal Pico Plus detection reagent (Thermo Fisher Scientific, 34578) and proteins were visualised with an Amersham imager LAS 4000 (Cytiva).

### Immunoprecipitation, kinase assays, and phosphatase assays

Immunoprecipitation assays were performed using 10^8^ yeast cells, which were resuspended in lysis buffer (50 mM HEPES-KOH (pH 7.5), 70 mM KOAc, 5 mM Mg(OAc)_2_, 10% glycerol, 0.1% Triton X-100, 8 μg/ml of protease inhibitors (leupeptin, pepstatin, aprotinin), 1 mM PMSF, 1× complete protease inhibitor without EDTA (Roche, 11873580001), 4 mM of phosphatase inhibitors (PIs) (β-glycerophosphate, NaF), and 1× PhosStop (Roche, 4906837001). Protein lysates were obtained by mechanical lysis using glass beads in a Bertin disrupter (6 cycles of 10 s at 5,000 rpm), clarified by centrifugation, and incubated with α-FLAG clone M2 antibody (Sigma, F3165) for 2 h. Protein extracts were then incubated for 1 h with protein A-conjugated Dynabeads (Invitrogen, 10002D), after which the beads were washed with lysis buffer at incremental KOAc concentrations (100 mM, 120 mM, 150 mM, and 60 mM NaOAc).

For kinase assays, beads were washed with 10 volumes of lysis buffer and twice with the kinase reaction buffer (20 mM Tris-HCl (pH 6.8), 5 mM MnCl_2_, 150 mM NaCl). The kinase reaction (kinase buffer supplemented with 25 μm ATP, 10 mCi/mL ^32^gamma-ATP, and 1 μg of the corresponding recombinant protein either MBP-Ace2-44-247 or His_6_-MBP-Sec3-1-320) was incubated at 30 °C for 60 min. Kinase assays were terminated by adding SDS-PAGE loading buffer. Proteins were separated by electrophoresis, transferred to nitrocellulose membranes, and radioactivity was detected in a PhosphoImager (TDI). Immunopurified protein was quantified by western blot and the membrane was stained with Coomassie to detect the recombinant substrates. Proteins were quantified using FiJi software [92]. Three experimental replicates were performed for experiments included in Fig 7A and 7D. The value for quantification of each kinase activity associated with wt Cbk1 was normalised to 1 to be able to compare values in 3 replicates.

For the Cbk1 alkaline phosphatase assay, cells were collected and Cbk1-5FLAG was immunopurified as above. The beads were incubated with alkaline phosphatase (0.5 units of AnP, New England Biolabs, M0289S) in 1× AnP buffer at 37 °C for 15 min. To inhibit the alkaline phosphatase 2× PhosStop (Roche, 4906837001) was used. The experiment was repeated 3 times.

### Protein expression and purification

MBP-Ace2-44-247 and His_6_-MBP-Sec3-1-320 were expressed in *E*. *coli* BL21(DE3). We grew bacteria cells in 1 litre of Luria–Bertani (LB) medium and protein expression was induced at an OD_600_ of 0.8 by the addition of 0.5 mM isopropyl-b-D-thiogalactopyranoside (IPTG). Cells were harvested after 16 h of growth at 18 °C. The cell pellet was resuspended in buffer A (50 mM Tris-HCl (pH 8.0), 300 mM NaCl, and 1 mM DTT) containing 0.1 mM phenylmethylsulfonyl fluoride (PMSF), 1 mM benzamidine, 0.5 mg/ml lysozyme, and 25 μg/ml DNAse. Bacteria were disrupted by sonication, and the lysate was cleared by centrifugation at 48,250 g for 45 min at 4 °C. MBP-Ace2-44-247 was purified with amylose affinity chromatography. Approximately 3 ml amylose resin (NEB) were added to the lysate supernatant and incubated for 1 h in batch. The resin was washed extensively with buffer A in a gravity column, and the immobilised protein was eluted by addition of buffer A supplemented with 10 mM maltose. MBP-Ace2-44-247 was further purified by size-exclusion chromatography using a HiLoad 16/600 Superdex 200 column (Cytiva) equilibrated with buffer B (25 mM Hepes (pH 7.5), 200 mM NaCl, and 1 mM DTT). His_6_-MBP-Sec3-1-320 was purified using immobilised metal affinity chromatography, and 2 ml Indigo-Ni resin (Cube Biotech) were mixed with the lysate supernatant, incubated for 1 h in batch, washed extensively with buffer A containing 20 mM imidazole in a gravity column, and eluted with buffer A containing 300 mM imidazole. Concentrated protein was further purified by size-exclusion chromatography (HiLoad 16/600 Superdex 200, Cytiva) in buffer B (25 mM Hepes (pH 7.5), 200 mM NaCl, and 1 mM DTT). Fractions were analysed by 12% SDS-PAGE and appropriate fractions pooled, flash frozen, and stored at −80 °C.

### Sample processing for mass spectrometry

To prepare cell extract, 5 g of frozen cell pellets were ground using a SPEX SamplePrep LLC 6850 freezer/mill. Subsequently, and prior digestion, Cbk1-GFP was immunoprecipitated on GFP-Trap Magnetic beads (ChromoTek, gtd-20). Immunoprecipitated proteins were washed 4 times with 200 mM ammonium bicarbonate (ABC) and resuspended in 6 M Urea/200 mM ABC. Then, the beads were reduced and alkylated with 10 mM dithiothreitol (DTT) and 57 mM chloroacetamide (CAA) for 1 h and 30 min, respectively, and digested with 1 μg of trypsin for 16 h at 30 °C. The tryptic peptides were recovered by pulling-down the beads. The supernatant was recovered to a new, clean tube and the digestion was stopped by adding 10% formic acid (FA). The peptides were then desalted using a PolyLC C18 pipette tip, dried in a speedvac, and stored to −80 °C.

### LC-MS/MS analysis

Digested peptides were reconstituted in 7 μl of 3% acetonitrile (ACN)/1% FA and 6 μl were injected to 300 μm × 5 mm C18 PepMap100, 5 μm, 100Å (Thermo Scientific) at a flow rate of 15 μl/min using a Thermo Scientific Dionex Ultimate 3000 chromatographic system (Thermo Scientific). Peptides were separated using a C18 analytical column (nanoEaseTM M/Z HSS C18 T3 (75 μm × 25 cm, 100 Å, Waters)) with a 120-min run, comprising 3 consecutive steps with linear gradients from 3% to 35% B in 90 min, from 35% to 50% B in 5 min, from 50% to 85% B in 2 min, followed by isocratic elution at 85% B in 5 min and stabilisation to initial conditions (A = 0.1% FA in water, B = 0.1% FA in CH3CN) at 250 nl/min flow rate.

The column outlets were directly connected to an Advion TriVersa NanoMate (Advion) fitted on an Orbitrap Fusion Lumos Tribrid mass spectrometer (Thermo). The mass spectrometer was operated in a data-dependent acquisition (DDA) mode. Survey MS scans were acquired in the Orbitrap with the resolution (defined at 200 m/z) set to 120,000. The lock mass was user-defined at 445.12 m/z in each Orbitrap scan. The top speed (most intense) ions per scan were fragmented by HCD. The MSMS was detected in the Orbitrap (with the resolution set to 30,000). The ion count target value was 400,000 for the survey scan and 10,000 (CID) for the MS/MS scan. Target ions already selected for MS/MS were dynamically excluded for 15 s. Spray voltage in the NanoMate source was set to 1.70 kV. RF lens were tuned to 30%. Minimal signal required to trigger MS to MS/MS switch was set to 5,000 and activation Q was 0.250. The spectrometer was working in positive polarity mode and singly charge state precursors were rejected for fragmentation. Data was acquired with Xcalibur software vs 4.0.27.10 (Thermo Scientific).

### Data analysis: Processing raw files and statistics

The RAW files were processed using the Maxquant 1.6.7.0 software. The peaks lists were searched against a SwissProt Yeast Database (downloaded in August 2020) with the help of the MaxQuant built-in search engine Andromeda. The false discovery rate (FDR) was assessed by using a decoy database [95]. Trypsin was selected as enzyme and a maximum of 2 missed cleavages were allowed. Carbamidomethylation in cysteines was set as a fixed modification, whereas oxidation in methionines and acetylation at the protein N-terminal were used as variable modifications. Additionally, for the identification of phosphosites, we added the phosphorylation in serines, threonines, and tyrosines as variable modification. Searches were performed using a peptide tolerance of 7 ppm and a product ion tolerance of 0.5 Da. Resulting data files were filtered for FDR <1%. Statistical analysis was performed with the help of R (https://cran.r-project.org/) and Rstudio (https://www.rstudio.com/).

### Data analysis: Analysis of interactome

The statistical analysis of the interactome was performed using the SAINT (Significance Analysis of INTeractome) algorithm [96], which converts the label free quantification (spectral counts) for each prey protein identified in an immunoprecipitation experiment of a bait protein into a probability of having a true interaction between 2 proteins.

## Supporting information

S1 Fig *cdc15-2* cells complete mitosis and cytokinesis after anaphase block and release. (A) An asynchronous culture of *GAL-SIC1ΔNT GLN3-9MYC* (YMF4471) was grown at 30 °C in medium lacking galactose. After the addition of nocodazole, culture was synchronised in G2-M phase for 1 generation time. Cells were then transferred to fresh medium containing galactose to allow overexpression of *SIC1ΔNT*. Cells were maintained as well as in nocodazole. Cell extract were made over the course of 2 h to examine Gln3 mobility. Raw data for blots can be found in Supporting information (S1 Raw Images). (B) *TUB1-GFP cdc15-2* (YMF3976) cells were grown in YPD and arrested in late anaphase by raising the temperature to 37 °C before the addition of rapamycin to half of the culture. Subsequently, to allow progression through the cell cycle, cells were released in the absence (−) or presence (+) of rapamycin. Samples were taken at the indicated times. Using fluorescence microscopy, the proportion of cells with anaphase spindles in the absence (i) or presence (ii) of rapamycin was investigated. Examples of *TUB1-GFP cdc15-2* cells at 120 min after the release at 24 °C are shown in the absence (iii) or presence (iv) of rapamycin. Scale bars indicate 5 μm. (C) *cdc15-2* cells (CC2274) were grown in parallel with strains for Fig 2A, but instead or releasing cells at the permissive temperature of 24 °C after the addition of rapamycin like in Fig 2A, cells were maintained at the restrictive temperature of 37 °C in the presence of rapamycin (i). Samples were taken at the indicated times to determine DNA content by flow cytometry analysis (ii) and cell morphology at the end of the experiment (iii). Scale bars indicate 5 μm. (D) *cdc14-1 cdc15-2* cells (CC6441) were grown in YPD and arrested in late anaphase by shifting the temperature to 37 °C before the addition of rapamycin to half of the culture (ii). Then, cells were released at 24 °C in the absence (i) or presence (ii) of rapamycin. Samples were taken at the specified times to determine DNA content by FACS analysis. Using light microscopy, we studied cell morphology in the absence (iii) or presence (iv) of rapamycin at the 120 min time point after the release from late anaphase arrest. Scale bars indicate 5 μm. (E) *INN1-GFP cdc15-2* (YMF3162) cells were grown as in A. Samples were taken at the indicated times. The proportion of cells with total Inn1-GFP signal in the absence (i) or presence (ii) of rapamycin was determined using fluorescence microscopy. The percentage of cells with either medial rings or spots of Inn1-GFP was calculated too. Examples of cells expressing Inn1-GFP 30 min after the release are shown in the absence (iii) or presence (iv) of rapamycin. Scale bars indicate 5 μm. (F) *3GFP-RAS2 cdc14-1* cells (CC6296) were grown as described in A. Cells are shown at 120 min after the release in the absence (i) or the presence (ii) of rapamycin. Red arrows denote cells where examination of each z-level at the bud neck showed divided cytoplasm and new buds are marked with white asterisks. Scale bars indicate 5 μm. Underlying data for all the graphs can be found in S1 Data file. FACS graphs can be found in the supplementary FACS file (S1 File).
(PDF)

S2 Fig Depletion of Cbk1 prevents *cdc15-2* cell-separation rescue after TORC1 inactivation. (A) Schematic illustration of the “auxin inducible degron” system. (i) The Cbk1 auxin degron strain (*cbk1-aid*, where *aid* denotes the auxin inducible degron) contains a version of Cbk1 in which the auxin inducible degron was fused to the C-terminus of *CBK1* in its own locus. Protein expression is under *CBK1* promoter and Cbk1-aid is able to perform wt Cbk1’s functions. The fusion was carried out in a yeast strain in which the F-box protein Tir1 is expressed constitutively under the control of *ADH1* promoter. Tir1 binds to SCF and forms the E3 ubiquitin ligase SCF-TIR1 that recruits the E2 ubiquitin conjugating enzyme [38]. (ii) Following the addition of auxins, the F-box Tir1 is able to specifically recognise the aid tag fused to Cbk1. Then, E2 ubiquitin conjugating enzyme polyubiquitylates aid, which rapidly promotes the degradation of Cbk1-aid by the proteasome (iii). This system allows to study the immediate biological consequences after Cbk1 depletion. (B) *ADH-TIR1* (YJW15) and *cbk1-aid ADH-TIR1* (YMF3657) cells were grown in YPD before the addition of NAA and IAA auxins. Samples were taken at the indicated times to determine cell-cycle progression by flow cytometry. (C) Schematic representation of experimental set-up in which cells were grown in YPD and arrested in late anaphase by shifting the temperature to 37 °C before the addition of NAA and IAA auxins for 50 min. Next, cells were incubated with DMSO (i) or rapamycin (ii) for 20 min while still arrested in anaphase. Cells were released in the absence (i) or presence (ii) of rapamycin, and with the NAA and IAA auxins present in medium throughout the rest of the experiment. (D) *cbk1*-*aid cdc15-2* (YMF3866) cells were grown as represented in C. Samples were taken at shown times to determine cell-cycle progression by flow cytometry ((i) and (ii)) and cell morphology by light microscopy in the absence (iii) or in the presence (iv) of rapamycin at 120 min after the release from late anaphase arrest. Scale bars indicate 5 μm. (E) (i) To study functionally compromised versions of Cbk1, we initially expressed both Cbk1 fused to aid and the altered Cbk1 protein whose sequence had been integrated as an extra copy together with 5′ and 3′ UTRs. Cells are not defective under permissive conditions as viability is supported by Cbk1-aid, whereas after the addition of auxins ((ii) restrictive conditions) Cbk1-aid is depleted and functional consequences of compromised Cbk1 expression could be determined. (F) *cbk1-D475A cbk1*-*aid cdc15-2* (YMF4047) cells were cultured an analysed as in D. (G) As control, *CBK1 cbk1*-*aid cdc15-2* (YMF4082) cells were grown and analysed as in D. FACS graphs can be found in the supplementary FACS file (S1 File).
(PDF)

S3 Fig Function of known Cbk1 regulators is independent of TORC1. (A) *CBK1-T743E cdc15-2* (EW1447), (B) *lre1Δ cdc15-2* (YMF3857), and (C) *fir1Δ cdc15-2* (YMS3792) cells were grown in YPD and arrested in late anaphase by raising the temperature to 37 °C before the addition of rapamycin to half of the culture for 20 min. Subsequently, to allow progression through the cell cycle, cells were released in the absence (−) or presence (+) of rapamycin. Samples were taken at the specified times to determine cell-cycle progression by flow cytometry. FACS graphs can be found in the supplementary FACS file (S1 File).
(PDF)

S4 Fig Conserved phosphorylation sites in Cbk1. The indicated fungal species were compared by CLUSTAL O multiple sequence alignment. Kinase domain and activation loop are marked. Serines and threonines followed by proline in *S*. *cerevisiae* Cbk1 are indicated with an asterisk and the corresponding residue. Those residues were changed to glutamic acid to mimic phosphorylation (*cbk1-6E*) [42]. This mutant was used in this work.
(PDF)

S5 Fig Analysis of the importance of each serine or threonine followed by proline in Cbk1-6E. (A) *cbk1-5E-E164S cbk1*-*aid cdc15-2* (YMF3905), (B) *cbk1-5E-E251S cbk1*-*aid cdc15-2* (YMF3910), (C) *cbk1-5E-E409S cbk1*-*aid cdc15-2* (YMF3995), (D) *cbk1-5E-E615T cbk1*-*aid cdc15-2* (YMF3906), and (E) *cbk1-5E-E711S cbk1*-*aid cdc15-2* (YMF3907) cells were grown in YPD and arrested in late anaphase by shifting the temperature to 37 °C before the addition of rapamycin to half of the culture for 20 min. To allow progression through the cell cycle, cells were released in the absence (i) or presence (ii) of rapamycin. Samples were taken at the indicated times to determine cell-cycle progression by flow cytometry. (F) 3D Cbk1 structure (PDB 4LQS [48]) in which different protein domains are highlighted. Residue T574 in the activation loop, together with residues D475 and S409 in the kinase domain are denoted. (G) *cbk1-T574A cbk1*-*aid cdc15-2* cells (YMF4191) were grown as above. FACS graphs can be found in the supplementary FACS file (S1 File).
(PDF)

S6 Fig Analysis of *CBK1* mutants in *cbk1*-deleted cells. (A) *CBK1 cbk1Δ cdc15-2* (YMF3869), (B) *cbk1-6E cbk1Δ cdc15-2* (YMF3763), (C) *cbk1-5E-E409S cbk1Δ cdc15-2* (YMF4279), (D) *cbk1-5E-E574T cbk1Δ cdc15-2* (YMF4280), and (E) *cbk1-9A cbk1Δ cdc15-2* (YMF3764) cells were grown in YPD and arrested in late anaphase by raising the temperature to 37 °C before rapamycin was added to half of the culture for 20 min. Subsequently, to allow progression through the cell cycle, cells were released in the absence (i) or presence (ii) of rapamycin. Samples were taken at the indicated times to study cell-cycle progression by flow cytometry. Schematic illustration of *cbk1-9A* mutant in which phosphosites containing serines or threonines followed by prolines were changed to alanine to block phosphorylations (iii). FACS graphs can be found in the supplementary FACS file (S1 File).
(PDF)

S7 Fig Chitinase Cts1 is unable to be localised at the site of division in *cdc14-1* cells. (A) *DSE4-6HA cdc15-2* (YMF4029) cells were grown in YPD and arrested in late anaphase by shifting the temperature to 37 °C before the addition of DMSO (−) or rapamycin (+) for 20 min. Subsequently, cells were released from the anaphase arrest in the absence (−) or presence (+) of rapamycin before protein extracts were prepared from shown time points and analysed by immunoblotting. Raw data for blot can be found in Supporting information (S6A Raw Images). (B) *iHA-CTS1 cdc14-1* cells (YMF4088) were grown and processed as in A. Protein extracts were prepared from indicated time points and analysed by immunoblotting. Raw data for blot can be found in Supporting information (S6B Raw Images). (C) *CTS1-GFPEnvy cdc14-1* cells (YMF4231) were grown as in C. Samples were taken at the indicated times to determine the proportion of cells with Cts1 at the division site in the presence (i) of rapamycin. Examples of cells are shown for the 60 min time point after the release at 24 °C in the presence (ii) of rapamycin. Scale bar indicates 5 μm. Underlying data for all the graphs can be found in S1 Data file.
(PDF)

S8 Fig Most of known Cbk1 interactions were maintained in all 3 conditions, independently of TORC1 activity. (A) *CBK1-GFP cdc15-2* (YMF3566) and *CBK1-GFP TOR1-1 cdc15-2* (YMF3565) cells were grown in YPD and arrested in late anaphase by shifting the temperature to 37 °C before the addition of rapamycin for 20 min to both strains. In addition, DMSO was added to one half of *CBK1-GFP cdc15-2*. Untagged *cdc15-2* (YMF3580) and *TOR1-1 cdc15-2* cells (YMF3578) were used as controls. Cell extracts were prepared before the immunoprecipitation of Cbk1-GFP on ChromoTek GFP-Trap Magnetic beads. Isolated material was subjected to analysis by mass spectrometry. Numbers represent spectral average of key Cbk1 functional-related interactors in 3 independent replicates of the experiment. The average is calculated as the sum of all spectral counts for the 3 replicates divided by 3. We found Cbk1 interactions previously described: Mob2 [48] or components of the RAM pathway such as Tao3 [97], Sog2 [98], and Kic1 [99]. Besides, Cbk1 negative regulators Lre1 and Fir1 were also identified [5,43]. Besides, MS found Myo1, a component of the actomyosin ring [43]. Our results showed that Cbk1 interacts with the transcription factor Ace2 [47,48]. Moreover, Cbk1 was bound to Ssd1, an RNA-binding protein that represses the translation of cell wall remodelling proteins until Ssd1 is phosphorylated by Cbk1 [48,100]. Interestingly, TORC1 and Ssd1 have been described to collaborate to maintain cellular integrity [73]. We found interactions with paralogs serine/threonine kinases Kin1 and Kin2 [74,75]. Finally, we detected protein Yol036w of unknown function interacting with Cbk1 [40,74]. Details of other known Cbk1 interactors are shown in S2 Table. (B) *CBK1-5FLAG ACE2-HA cdc15-2* (YMF3585) and control cells (YMF3587) (i), *CBK1-5FLAG MOB2-9MYC cdc15-2* (YMF3838) and control cells (YMF3835) (ii), or *CBK1-TAP SOG2-6HA cdc15-2* (YMF4235) and control cells (YMF4236) cells (iii) were grown in YPD and arrested in late anaphase by shifting to 37 °C before the addition of rapamycin for 20 min when indicated. Subsequently, protein extracts were prepared and immunoprecipitations of Cbk1 on FLAG-beads or IgG beads (as denoted) were performed before the detection of the indicated proteins by immunoblotting. Raw data for blots can be found in Supporting information (S7 Raw Images). (C) Components of TORC1 were found in the same mass spectrometry analysis as in A. Numbers represent spectral average of 3 independent replicates of the experiment.
(PDF)

S9 Fig Sec3 contains conserved NDR/LATS phosphorylation consensus sites. (A) Orthologues of *S*. *cerevisiae* Sec3 in the indicated fungal species were identified by PSI-BLAST searches, and a CLUSTAL O multiple sequence alignment was performed. Conserved NDR/LATS phosphorylation consensus is marked with a red square following consensus in *S*. *cerevisiae* [47] and in *S*. *pombe* [54]. Putative Cbk1 phosphorylation sites were identified in Sec3 by scanning the sequence (S18, S32, S43, and S66). (B) To confirm that Sec3 was essential in the budding yeast strain used in this work, we deleted *SEC3* in diploid cells (YMF4159). Spores that lack Sec3 were able to grow extremely deficiently, although they formed tiny colonies (i). To conditionally inactivate Sec3, we planned to follow the same strategy as we did for the study of Cbk1 mutants that is explained in detail in S2E Fig. We added the “auxin inducible degron” (“aid”) cassette [38] to the C-terminal end of *SEC3*. Then, control cells (*ADH-TIR1*; YJW15) and *sec3-aid ADH-TIR1* cells (YMF4612) were grown at 24 °C on YPD medium before serial dilutions of 50,000, 5,000, 500, and 50 cells were plated on the indicated media and incubated for 3 days (ii). *sec3-aid ADH-TIR1* cells were able to grow under restrictive conditions at 24 °C (ii), which showed that Sec3 depletion was not completely effective. This prevented us from the use of *sec3-aid* mutant, in combination with *cdc15-2* to inactivate Cdc15 function at 37 °C, and test whether phosphomimetic version of Sec3 (Sec3-4E, S18E, S32E, S43E, and S66E) was able to rescue cell separation defects associated to *cdc15-2* cells. (C) To be able to follow version of t-SNARE Sso1 that lacks N-terminal autoinhibition domain, we deleted DNA sequence that corresponds to amino acids 1–146. At the same time, we fused the tag 5xFLAG at the N-terminal of the truncated Sso1 to be able to follow the protein dynamics. As control, we tagged the N-terminal end of wt Sso1 with 5xFLAG. To determine whether 5xFLAG-Sso1 and 5xFLAG-Sso1Δ1–146 were functional, we combined *5xFLAG-SSO1* (i) or *5xFLAG-sso1Δ1–146* (ii) with *sso2Δ*. In both cases, harbouring 5xFLAG version of Sso1 and *sso2Δ* was lethal, which indicates that addition of 5xFLAG disturbed Sso1 function. Individual deletions of *SSO1* and *SSO2* are alive, but inactivation of both t-SNARE Sso proteins is lethal.
(PDF)

S10 Fig Proposed model for how TORC1 might regulate Cbk1 activity in *cdc15-2* cells. (A) Our findings suggest that TORC1 blocks Cbk1 kinase activity in the absence of Cdc15 function while cells are arrested in late anaphase at 37 °C, which would promote a defect in the following cell separation as secretory vesicle transporting hydrolases would be unable to fuse into the plasma membrane. (B) Rapamycin inhibits TORC1 and would induce the accumulation of a hypophosphorylated version of Cbk1 that would be able to phosphorylate its key substrates as the exocyct component Sec3, which regulates the function of the SNARE complex at the site of division to promote fusion of secretory vesicle at the plasma membrane. Hydrolases contained in secretory vesicle are released to promote cell separation.
(PDF)

S1 Table Identification of TORC1-regulated phosphorylation sites in Cbk1 and its interactors. Sheet includes all raw data. Summary could be found in Table 1.
(XLSX)

S2 Table Interactions between Cbk1 and its known interactors. Same conditions as in S1 Table. We found Cbk1 described interactions in *cdc15-2 cells* in the absence (A “*cdc15-2* vs. control”), the presence of rapamycin (B “*cdc15-2* + rapa vs. ctrl”), and *TOR1-1 cdc15-2* cells after the addition of rapamycin (C “*TOR1-1 cdc15-2* + rapa vs. ctrl”). In all cases, cells were arrested in late anaphase after having been grown at 37 °C. Three biological replicates of each described cells and conditions were performed and compared with 3 biological replicates of untagged control cells (ctrlCounts). Cbk1 interactions can be found in: https://thebiogrid.org/35668/summary/saccharomyces-cerevisiae/cbk1.html.
(XLSX)

S3 Table Novel interactions of Cbk1 with components of the exocyst, SNARE and TORC1 complexes. Same conditions as in S1 Table.
(XLSX)

S4 Table Strains used in this study. All based on W303.
(DOC)

S5 Table Plasmids used in this study. (DOC)

S6 Table Oligonucleotides used in this study. (DOCX)

S1 Raw Images Original images supporting blot results for Figs 1B, 1C, 1D, 1E and S1A. (PDF)

S2 Raw Images Original images supporting blot results for Fig 2F. (PDF)

S3 Raw Images Original images supporting blot results for Figs 3D, 3E, 3F, 3G and 3H. (PDF)

S4 Raw Images Original images supporting blot results for Figs 5B and 5C. (PDF)

S5 Raw Images Original images supporting blot results for Figs 7A, 7C, 7D and 7E. (PDF)

S6 Raw Images Original images supporting blot results for S7A and S7B Fig. (PDF)

S7 Raw Images Original images supporting blot results for S8B Fig. (PDF)

S1 File Supplementary FACS file. (ZIP)

S1 Data Source data for the main figures (Figs 1D, 1E, 3A, 3C, 3H, 5D, 5E, 5F, 6B, 6C, 7A and 7D) and supporting figures (S1B, S1E and S7C Figs). (XLSX)

## Abbreviations

ABC: ammonium bicarbonate
AnP: Antarctic Phosphatase
APC/C: anaphase-promoting complex/cyclosome
DDA: data-dependent acquisition
ECO: enforcement of cytokinesis order
FA: formic acid
FDR: false discovery rate
HM: hydrophobic motif
LB: Luria–Bertani
MBP: maltose binding protein
MEN: mitotic exit network
mTOR: mammalian TOR
NDR: nuclear Dbf2-related
PH: pleckstrin homology
PI: phosphatase inhibitor
RAM: regulation of Ace2 and morphogenesis
TCA: trichloride acetic acid
TOR: target of rapamycin
wt: wild-type
