# Supplementary figures and images for: TOR complex 1 negatively regulates NDR kinase Cbk1 to control cell separation in budding yeast
Source: PLoS Biol. 2023 Aug 30;21(8):e3002263. doi: 10.1371/journal.pbio.3002263 (PMC10468069; doi:10.1371/journal.pbio.3002263)

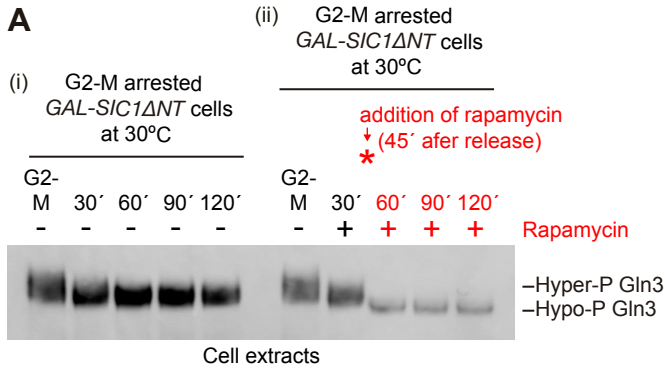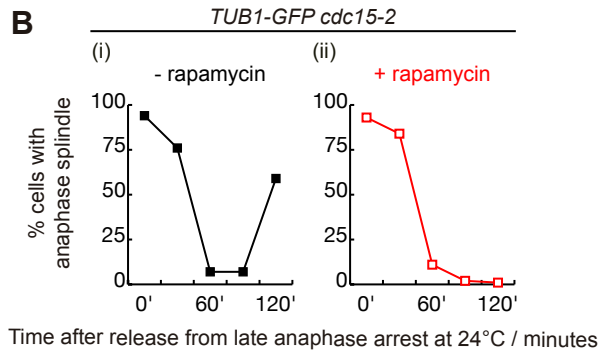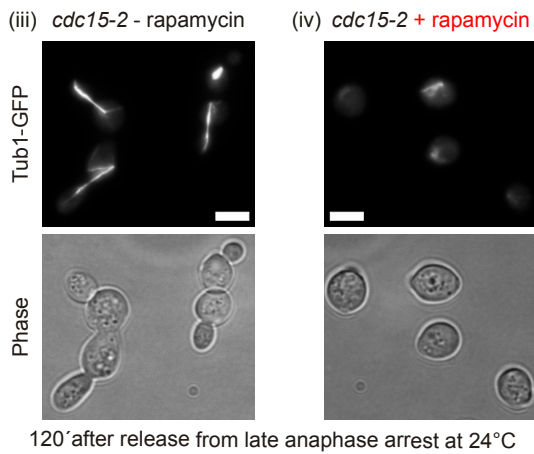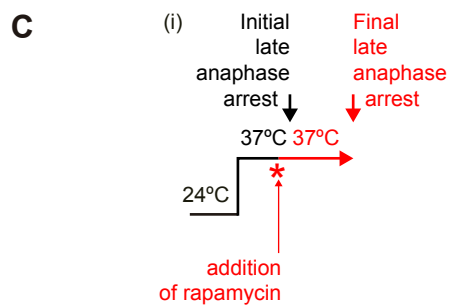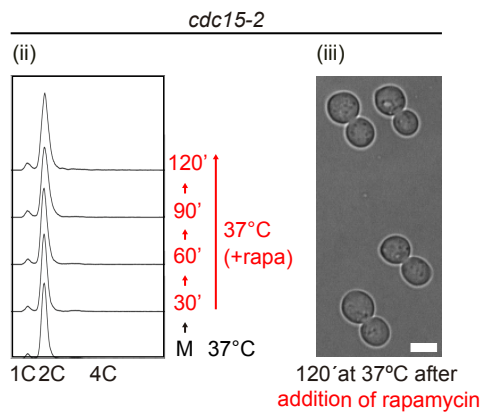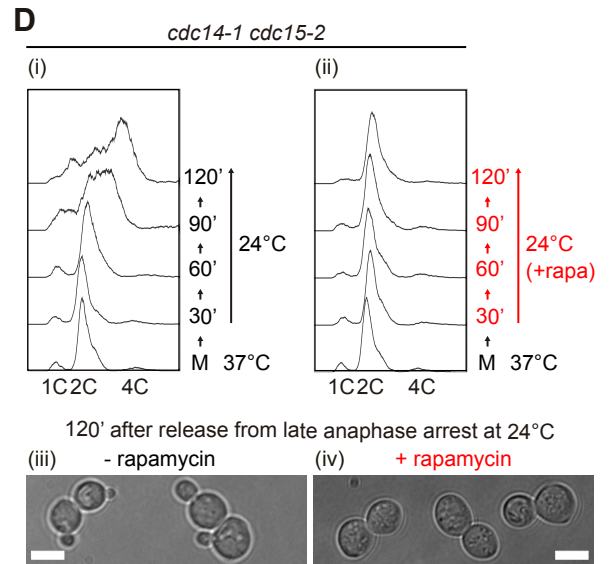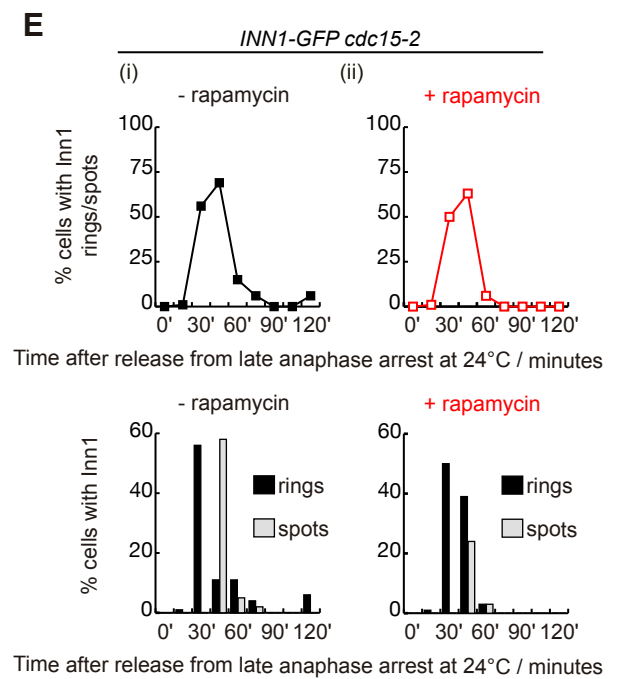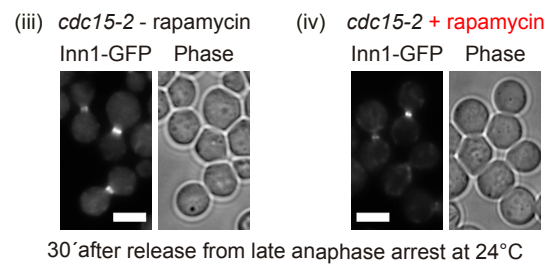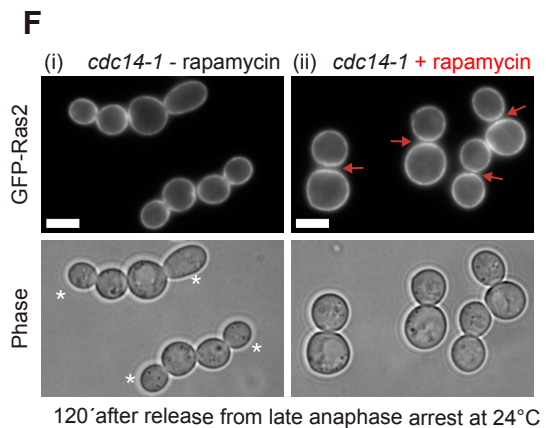

Supplement: S1 Fig — (A) An asynchronous culture of GAL-SIC1ΔNT GLN3-9MYC (YMF4471) was grown at 30 °C in medium lacking galactose. After the addition of nocodazole, culture was synchronised in G2-M phase for 1 generation time. Cells were then transferred to fresh medium containing galactose to allow overexpression of SIC1ΔNT. Cells were maintained as well as in nocodazole. Cell extract were made over the course of 2 h to examine Gln3 mobility. Raw data for blots can be found in Supporting information (S1 Raw Images). (B) TUB1-GFP cdc15-2 (YMF3976) cells were grown in YPD and arrested in late anaphase by raising the temperature to 37 °C before the addition of rapamycin to half of the culture. Subsequently, to allow progression through the cell cycle, cells were released in the absence (−) or presence (+) of rapamycin. Samples were taken at the indicated times. Using fluorescence microscopy, the proportion of cells with anaphase spindles in the absence (i) or presence (ii) of rapamycin was investigated. Examples of TUB1-GFP cdc15-2 cells at 120 min after the release at 24 °C are shown in the absence (iii) or presence (iv) of rapamycin. Scale bars indicate 5 μm. (C) cdc15-2 cells (CC2274) were grown in parallel with strains for Fig 2A, but instead or releasing cells at the permissive temperature of 24 °C after the addition of rapamycin like in Fig 2A, cells were maintained at the restrictive temperature of 37 °C in the presence of rapamycin (i). Samples were taken at the indicated times to determine DNA content by flow cytometry analysis (ii) and cell morphology at the end of the experiment (iii). Scale bars indicate 5 μm. (D) cdc14-1 cdc15-2 cells (CC6441) were grown in YPD and arrested in late anaphase by shifting the temperature to 37 °C before the addition of rapamycin to half of the culture (ii). Then, cells were released at 24 °C in the absence (i) or presence (ii) of rapamycin. Samples were taken at the specified times to determine DNA content by FACS analysis. Using light microsco [file pbio.3002263.s001.pdf]

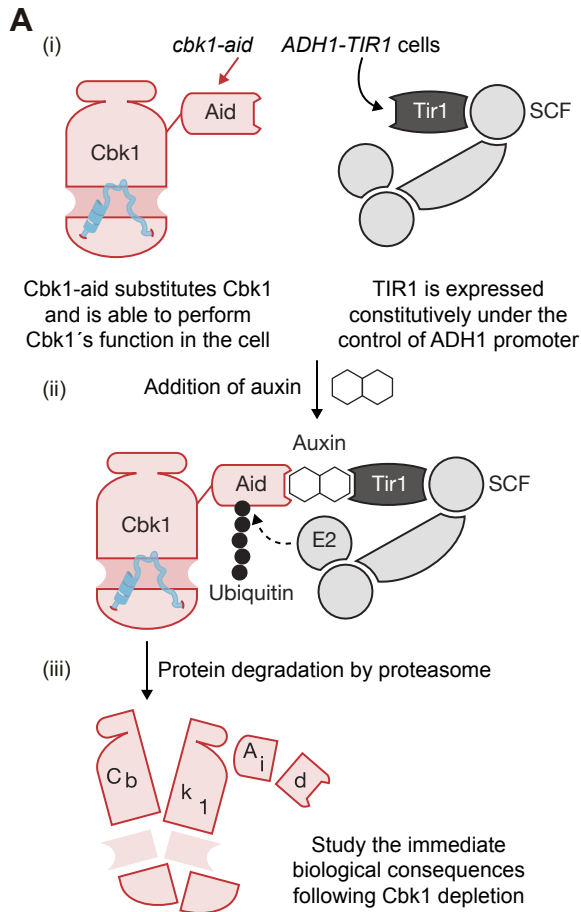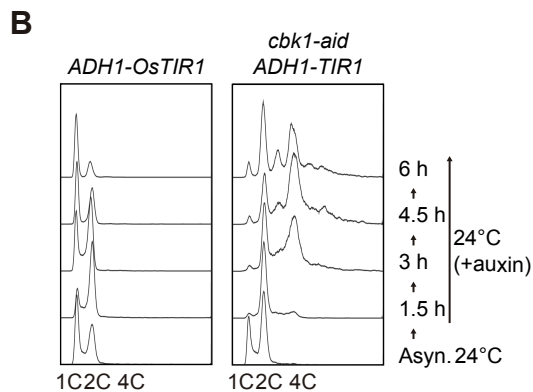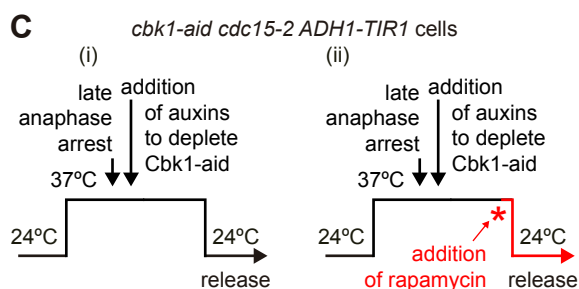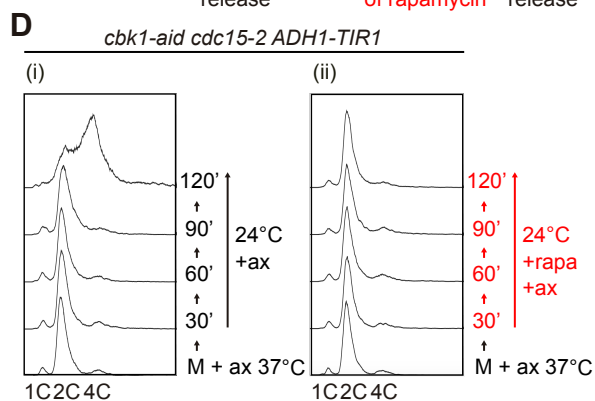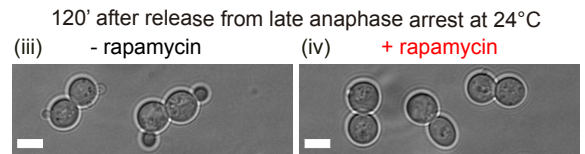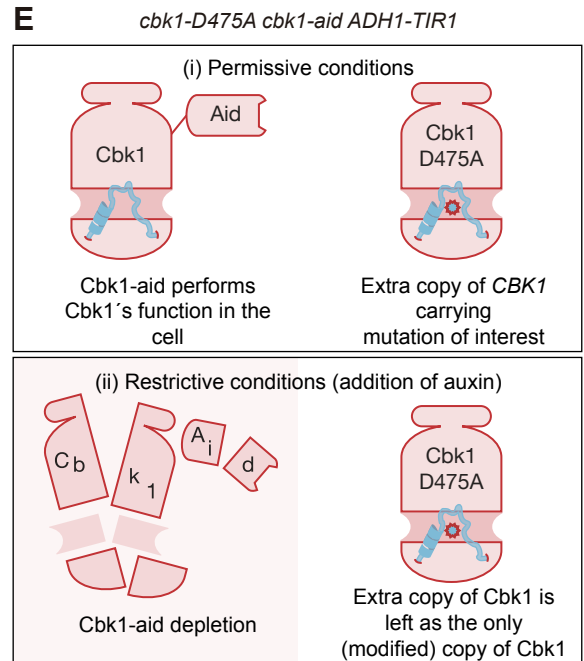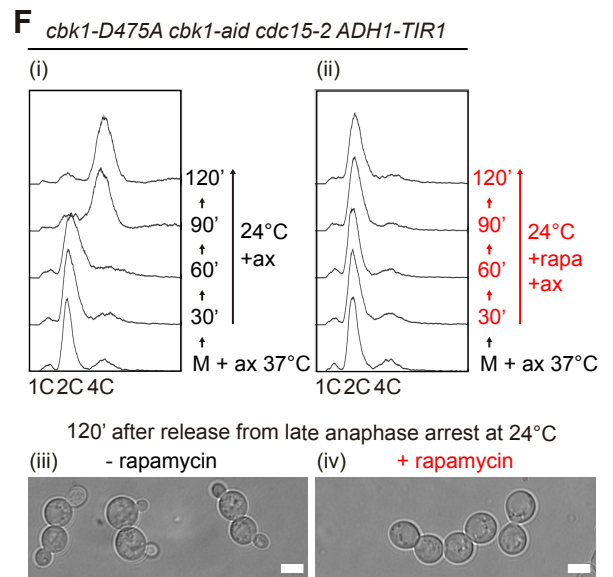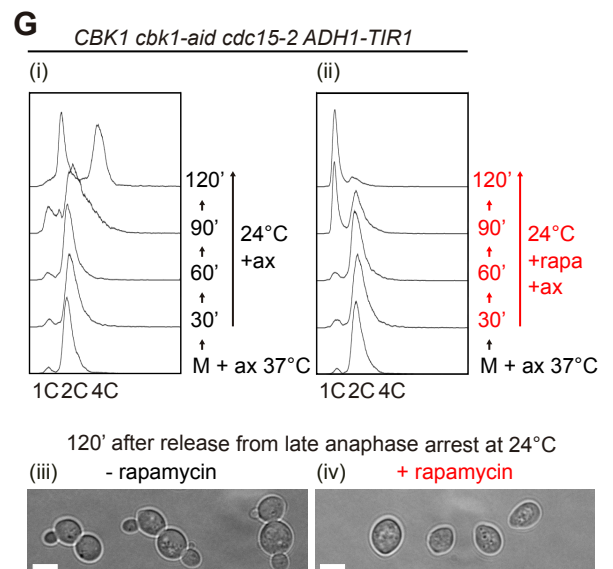

Supplement: S2 Fig — (A) Schematic illustration of the “auxin inducible degron” system. (i) The Cbk1 auxin degron strain (cbk1-aid, where aid denotes the auxin inducible degron) contains a version of Cbk1 in which the auxin inducible degron was fused to the C-terminus of CBK1 in its own locus. Protein expression is under CBK1 promoter and Cbk1-aid is able to perform wt Cbk1’s functions. The fusion was carried out in a yeast strain in which the F-box protein Tir1 is expressed constitutively under the control of ADH1 promoter. Tir1 binds to SCF and forms the E3 ubiquitin ligase SCF-TIR1 that recruits the E2 ubiquitin conjugating enzyme [38]. (ii) Following the addition of auxins, the F-box Tir1 is able to specifically recognise the aid tag fused to Cbk1. Then, E2 ubiquitin conjugating enzyme polyubiquitylates aid, which rapidly promotes the degradation of Cbk1-aid by the proteasome (iii). This system allows to study the immediate biological consequences after Cbk1 depletion. (B) ADH-TIR1 (YJW15) and cbk1-aid ADH-TIR1 (YMF3657) cells were grown in YPD before the addition of NAA and IAA auxins. Samples were taken at the indicated times to determine cell-cycle progression by flow cytometry. (C) Schematic representation of experimental set-up in which cells were grown in YPD and arrested in late anaphase by shifting the temperature to 37 °C before the addition of NAA and IAA auxins for 50 min. Next, cells were incubated with DMSO (i) or rapamycin (ii) for 20 min while still arrested in anaphase. Cells were released in the absence (i) or presence (ii) of rapamycin, and with the NAA and IAA auxins present in medium throughout the rest of the experiment. (D) cbk1-aid cdc15-2 (YMF3866) cells were grown as represented in C. Samples were taken at shown times to determine cell-cycle progression by flow cytometry ((i) and (ii)) and cell morphology by light microscopy in the absence (iii) or in the presence (iv) of rapamycin at 120 min after the release from late anaphase arrest. Scale bars indicate 5 [file pbio.3002263.s002.pdf]

**A***CBK1-T743E cdc15-2*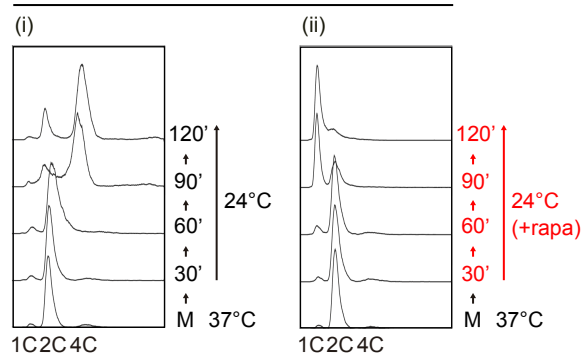**B***lre1Δ cdc15-2*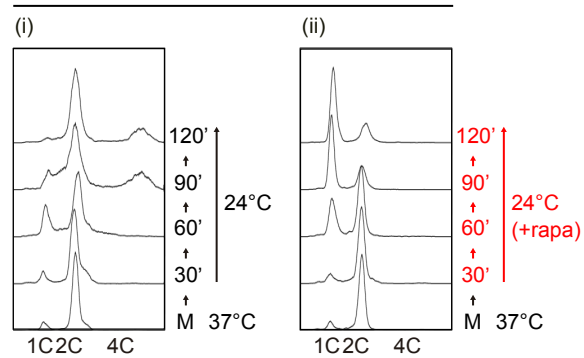**C***fir1Δ cdc15-2*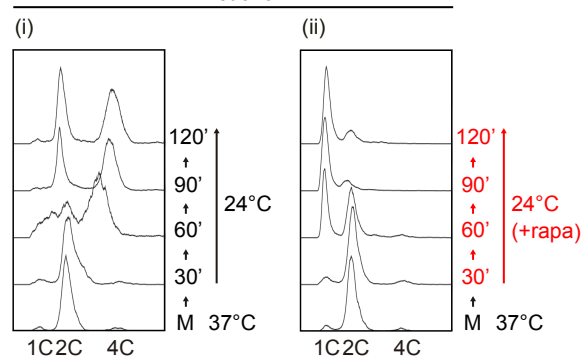

Supplement: S3 Fig — (A) CBK1-T743E cdc15-2 (EW1447), (B) lre1Δ cdc15-2 (YMF3857), and (C) fir1Δ cdc15-2 (YMS3792) cells were grown in YPD and arrested in late anaphase by raising the temperature to 37 °C before the addition of rapamycin to half of the culture for 20 min. Subsequently, to allow progression through the cell cycle, cells were released in the absence (−) or presence (+) of rapamycin. Samples were taken at the specified times to determine cell-cycle progression by flow cytometry. FACS graphs can be found in the supplementary FACS file (S1 File). (PDF) [file pbio.3002263.s003.pdf]

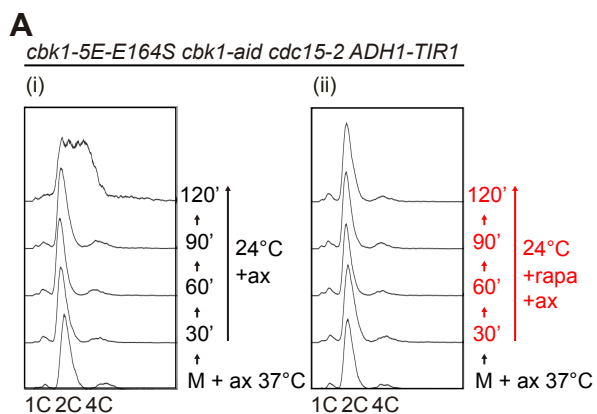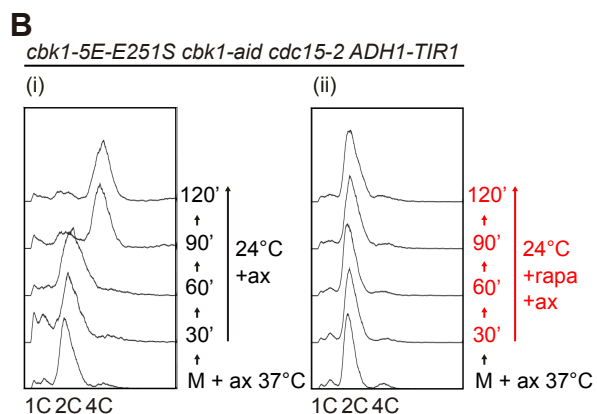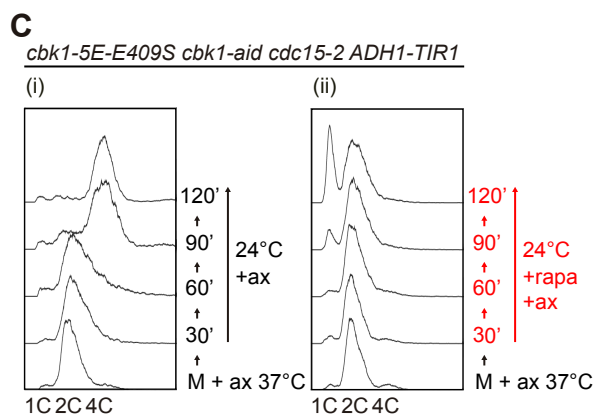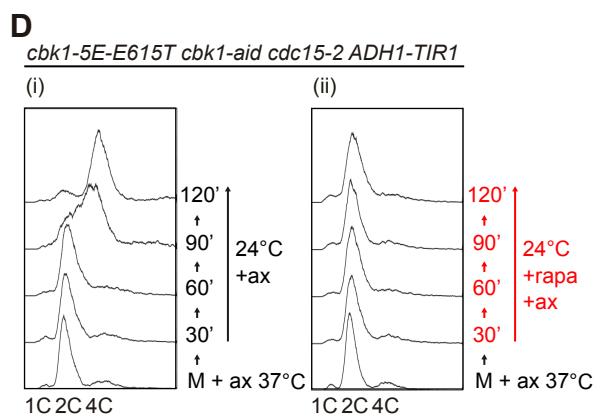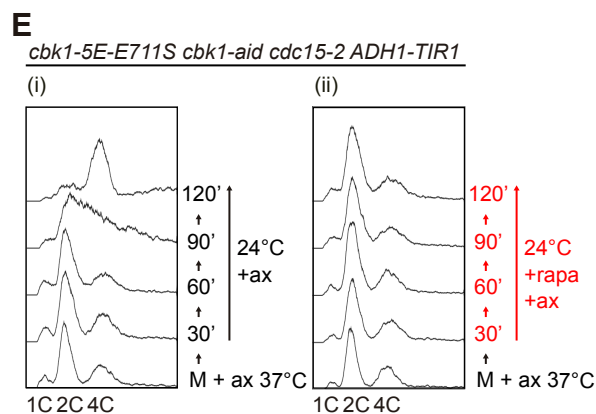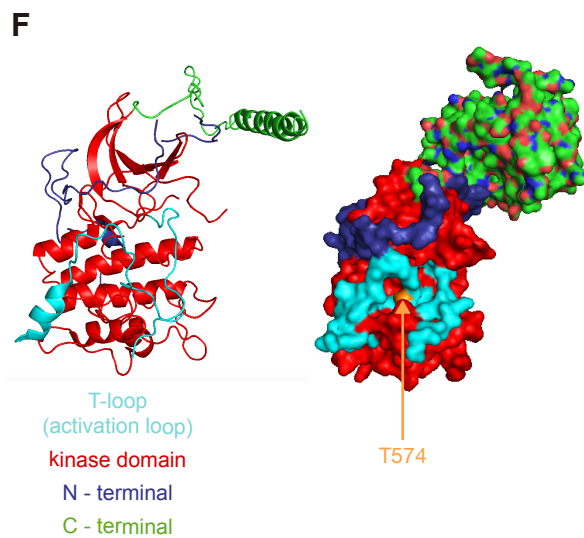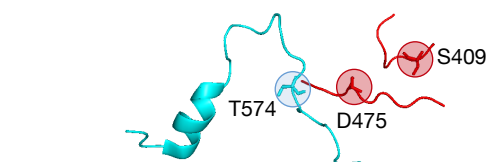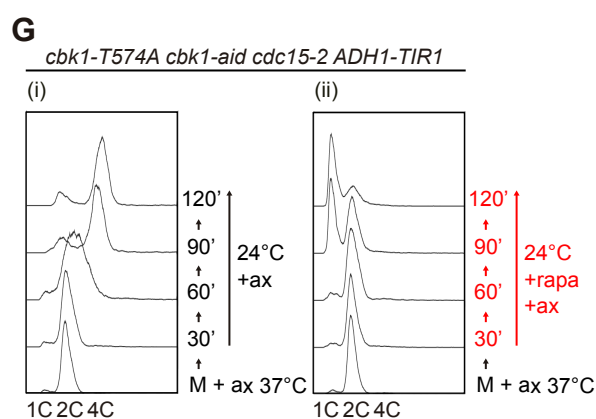

Supplement: S5 Fig — (A) cbk1-5E-E164S cbk1-aid cdc15-2 (YMF3905), (B) cbk1-5E-E251S cbk1-aid cdc15-2 (YMF3910), (C) cbk1-5E-E409S cbk1-aid cdc15-2 (YMF3995), (D) cbk1-5E-E615T cbk1-aid cdc15-2 (YMF3906), and (E) cbk1-5E-E711S cbk1-aid cdc15-2 (YMF3907) cells were grown in YPD and arrested in late anaphase by shifting the temperature to 37 °C before the addition of rapamycin to half of the culture for 20 min. To allow progression through the cell cycle, cells were released in the absence (i) or presence (ii) of rapamycin. Samples were taken at the indicated times to determine cell-cycle progression by flow cytometry. (F) 3D Cbk1 structure (PDB 4LQS [48]) in which different protein domains are highlighted. Residue T574 in the activation loop, together with residues D475 and S409 in the kinase domain are denoted. (G) cbk1-T574A cbk1-aid cdc15-2 cells (YMF4191) were grown as above. FACS graphs can be found in the supplementary FACS file (S1 File). (PDF) [file pbio.3002263.s005.pdf]

**A**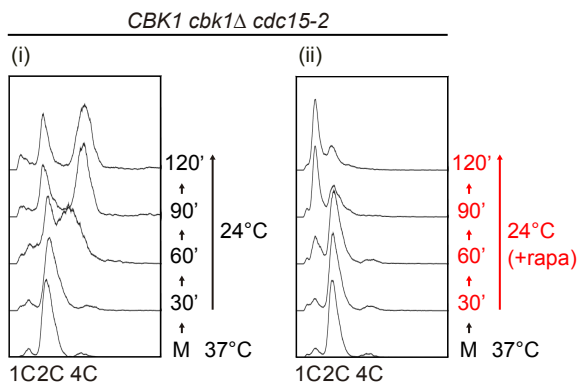**B**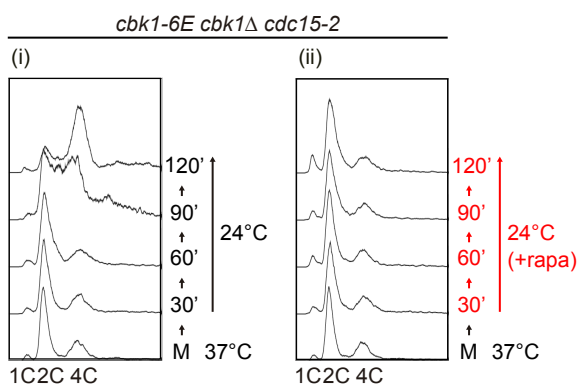**C**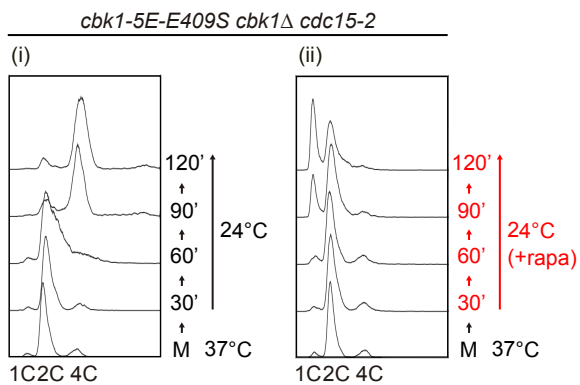**D**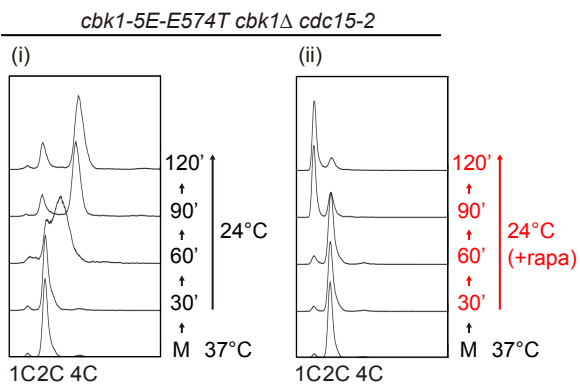**E**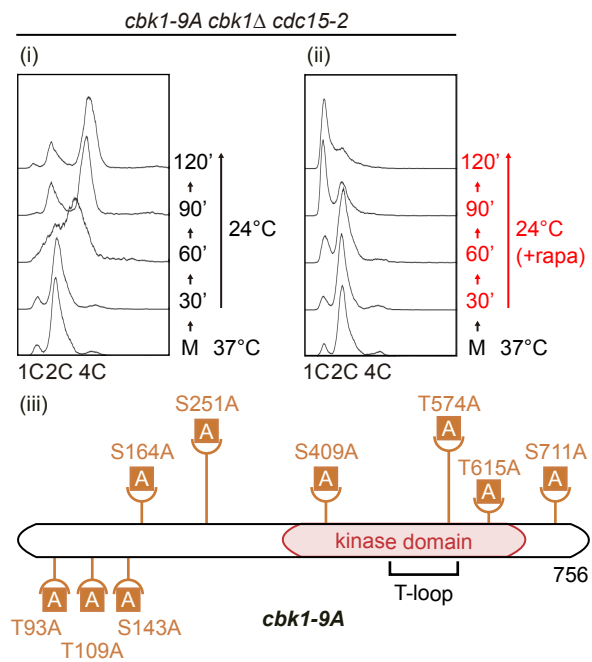

Supplement: S6 Fig — (A) CBK1 cbk1Δ cdc15-2 (YMF3869), (B) cbk1-6E cbk1Δ cdc15-2 (YMF3763), (C) cbk1-5E-E409S cbk1Δ cdc15-2 (YMF4279), (D) cbk1-5E-E574T cbk1Δ cdc15-2 (YMF4280), and (E) cbk1-9A cbk1Δ cdc15-2 (YMF3764) cells were grown in YPD and arrested in late anaphase by raising the temperature to 37 °C before rapamycin was added to half of the culture for 20 min. Subsequently, to allow progression through the cell cycle, cells were released in the absence (i) or presence (ii) of rapamycin. Samples were taken at the indicated times to study cell-cycle progression by flow cytometry. Schematic illustration of cbk1-9A mutant in which phosphosites containing serines or threonines followed by prolines were changed to alanine to block phosphorylations (iii). FACS graphs can be found in the supplementary FACS file (S1 File). (PDF) [file pbio.3002263.s006.pdf]

**A**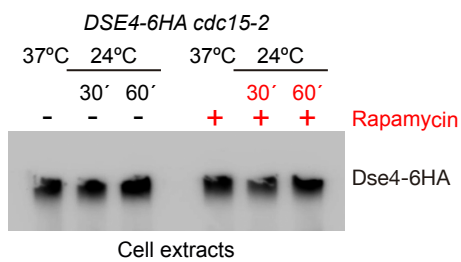**B**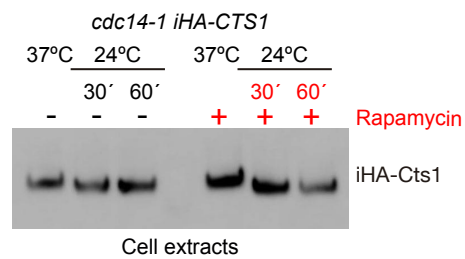**C**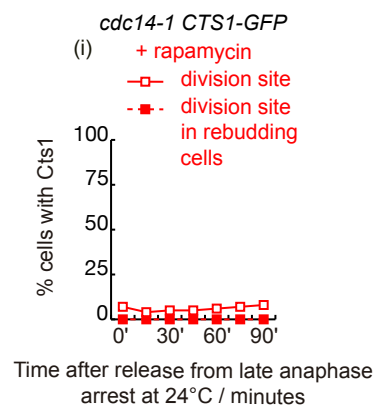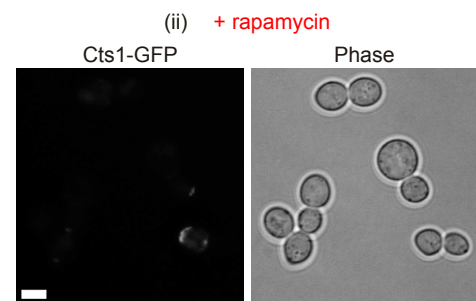

60' after release from late anaphase arrest at 24°C

Supplement: S7 Fig — (A) DSE4-6HA cdc15-2 (YMF4029) cells were grown in YPD and arrested in late anaphase by shifting the temperature to 37 °C before the addition of DMSO (−) or rapamycin (+) for 20 min. Subsequently, cells were released from the anaphase arrest in the absence (−) or presence (+) of rapamycin before protein extracts were prepared from shown time points and analysed by immunoblotting. Raw data for blot can be found in Supporting information (S6A Raw Images). (B) iHA-CTS1 cdc14-1 cells (YMF4088) were grown and processed as in A. Protein extracts were prepared from indicated time points and analysed by immunoblotting. Raw data for blot can be found in Supporting information (S6B Raw Images). (C) CTS1-GFPEnvy cdc14-1 cells (YMF4231) were grown as in C. Samples were taken at the indicated times to determine the proportion of cells with Cts1 at the division site in the presence (i) of rapamycin. Examples of cells are shown for the 60 min time point after the release at 24 °C in the presence (ii) of rapamycin. Scale bar indicates 5 μm. Underlying data for all the graphs can be found in S1 Data file. (PDF) [file pbio.3002263.s007.pdf]

**A**

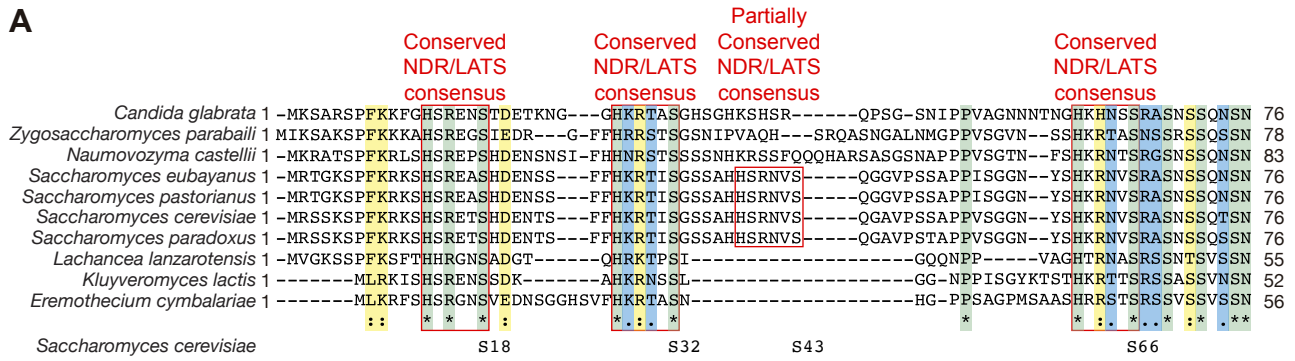

**B**

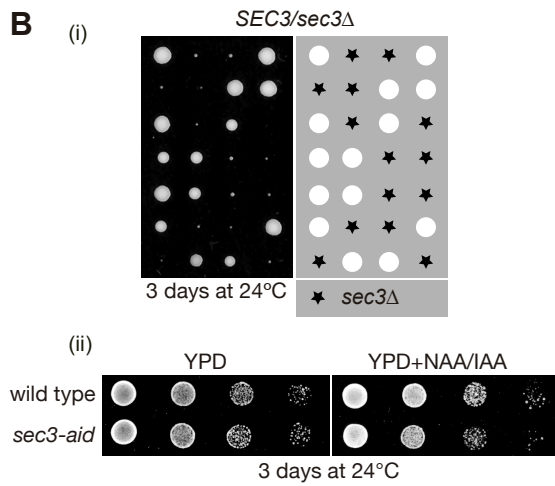

**C**

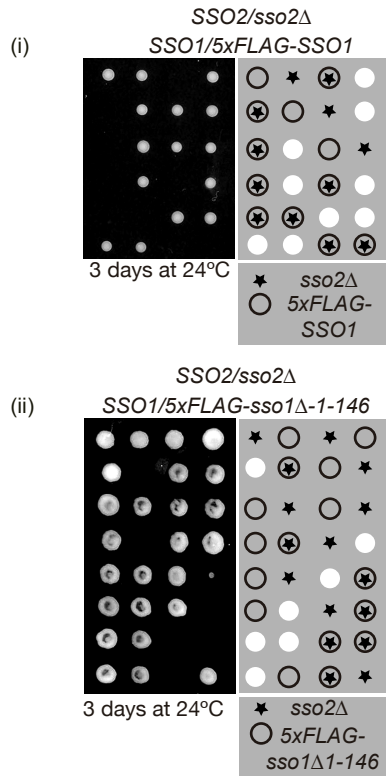

Supplement: S9 Fig — (A) Orthologues of S. cerevisiae Sec3 in the indicated fungal species were identified by PSI-BLAST searches, and a CLUSTAL O multiple sequence alignment was performed. Conserved NDR/LATS phosphorylation consensus is marked with a red square following consensus in S. cerevisiae [47] and in S. pombe [54]. Putative Cbk1 phosphorylation sites were identified in Sec3 by scanning the sequence (S18, S32, S43, and S66). (B) To confirm that Sec3 was essential in the budding yeast strain used in this work, we deleted SEC3 in diploid cells (YMF4159). Spores that lack Sec3 were able to grow extremely deficiently, although they formed tiny colonies (i). To conditionally inactivate Sec3, we planned to follow the same strategy as we did for the study of Cbk1 mutants that is explained in detail in S2E Fig. We added the “auxin inducible degron” (“aid”) cassette [38] to the C-terminal end of SEC3. Then, control cells (ADH-TIR1; YJW15) and sec3-aid ADH-TIR1 cells (YMF4612) were grown at 24 °C on YPD medium before serial dilutions of 50,000, 5,000, 500, and 50 cells were plated on the indicated media and incubated for 3 days (ii). sec3-aid ADH-TIR1 cells were able to grow under restrictive conditions at 24 °C (ii), which showed that Sec3 depletion was not completely effective. This prevented us from the use of sec3-aid mutant, in combination with cdc15-2 to inactivate Cdc15 function at 37 °C, and test whether phosphomimetic version of Sec3 (Sec3-4E, S18E, S32E, S43E, and S66E) was able to rescue cell separation defects associated to cdc15-2 cells. (C) To be able to follow version of t-SNARE Sso1 that lacks N-terminal autoinhibition domain, we deleted DNA sequence that corresponds to amino acids 1–146. At the same time, we fused the tag 5xFLAG at the N-terminal of the truncated Sso1 to be able to follow the protein dynamics. As control, we tagged the N-terminal end of wt Sso1 with 5xFLAG. To determine whether 5xFLAG-Sso1 and 5xFLAG-Sso1Δ1–146 were functional, we combined 5xFLAG-SSO1 (i) [file pbio.3002263.s009.pdf]

**A**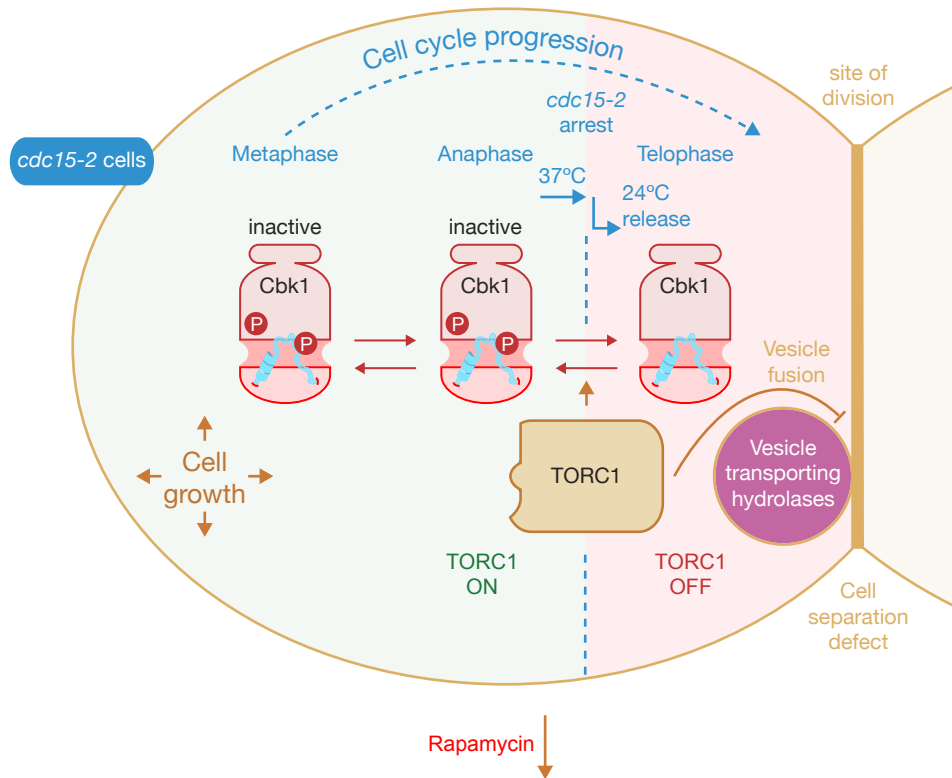**B**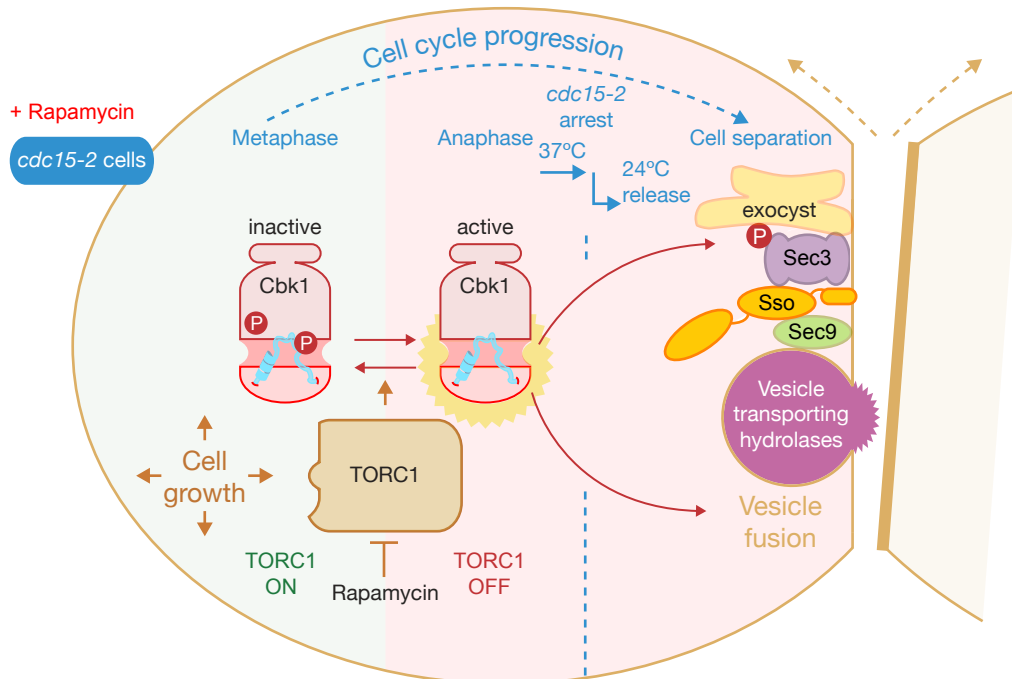

Supplement: S10 Fig — (A) Our findings suggest that TORC1 blocks Cbk1 kinase activity in the absence of Cdc15 function while cells are arrested in late anaphase at 37 °C, which would promote a defect in the following cell separation as secretory vesicle transporting hydrolases would be unable to fuse into the plasma membrane. (B) Rapamycin inhibits TORC1 and would induce the accumulation of a hypophosphorylated version of Cbk1 that would be able to phosphorylate its key substrates as the exocyct component Sec3, which regulates the function of the SNARE complex at the site of division to promote fusion of secretory vesicle at the plasma membrane. Hydrolases contained in secretory vesicle are released to promote cell separation. (PDF) [file pbio.3002263.s010.pdf]

**B**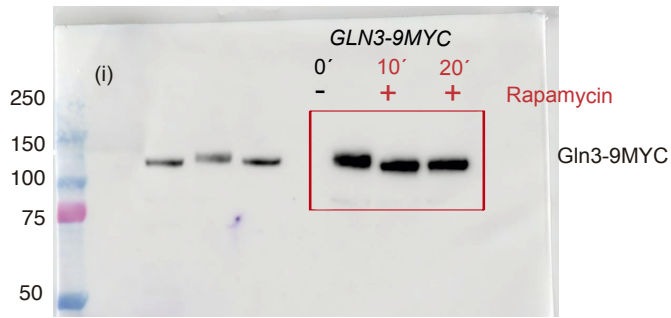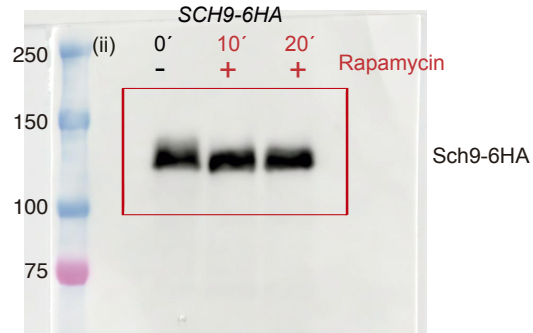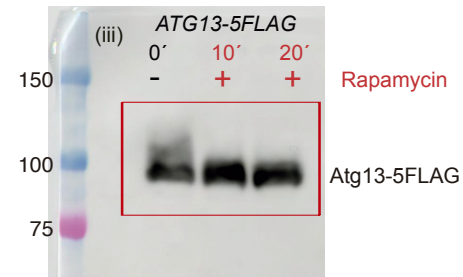**C**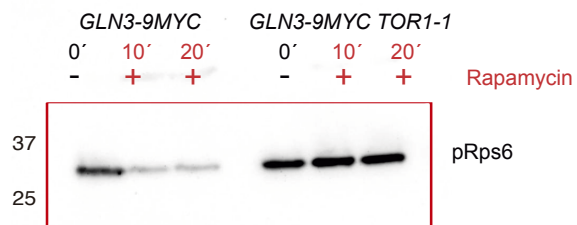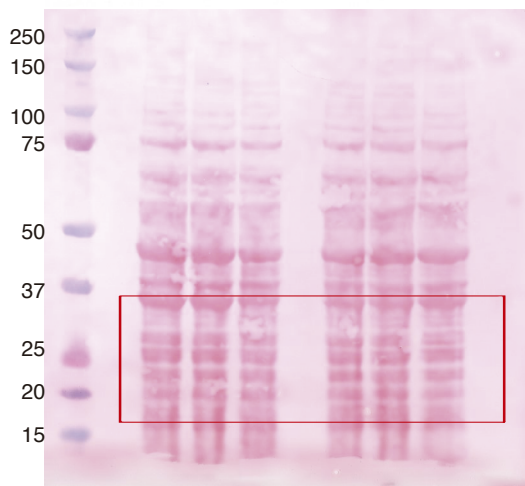

Cell extracts

**D**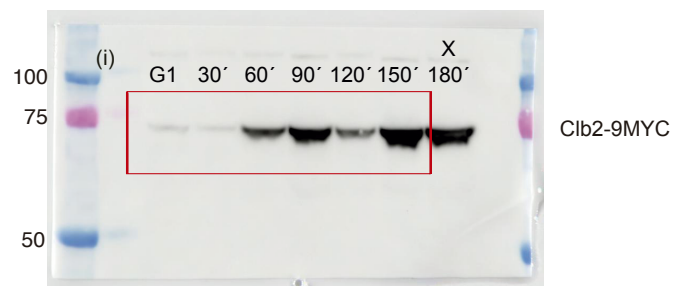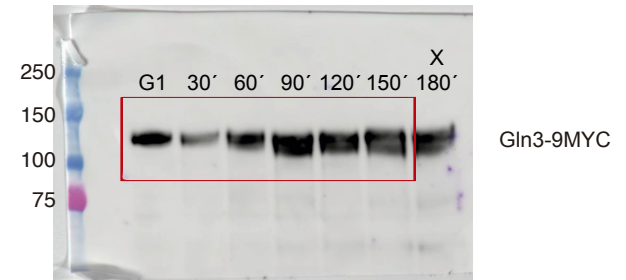**E**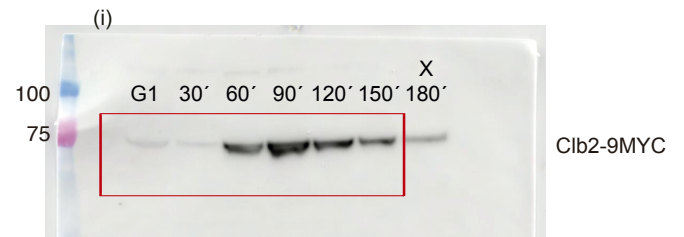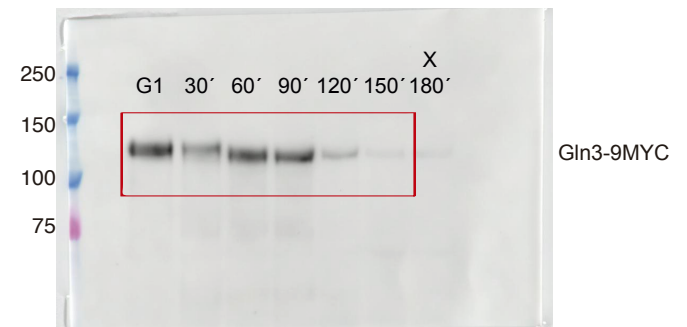**S1A Fig.**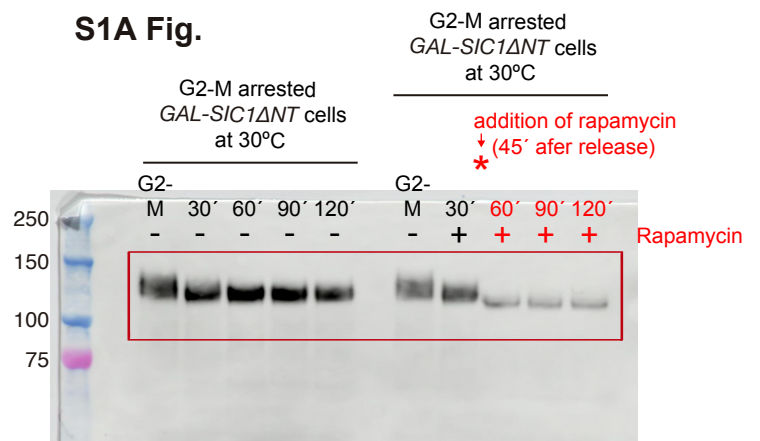

Supplement: S1 Raw Images — (PDF) [file pbio.3002263.s017.pdf]

F

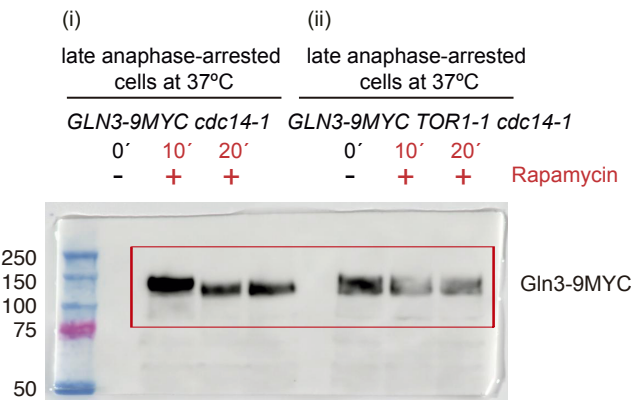

Supplement: S2 Raw Images — (PDF) [file pbio.3002263.s018.pdf]

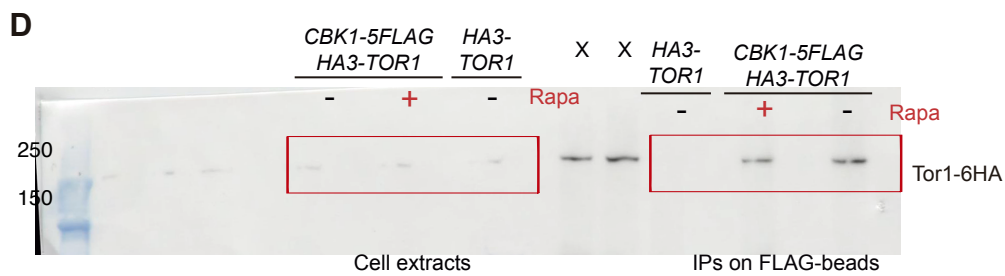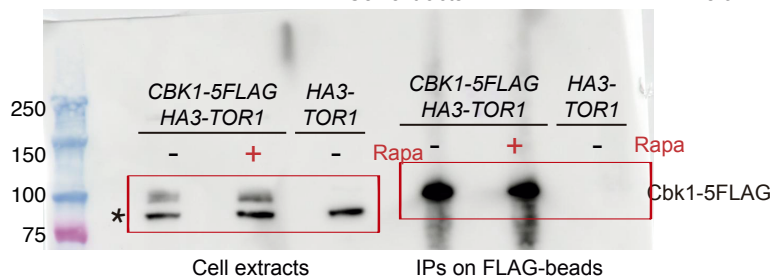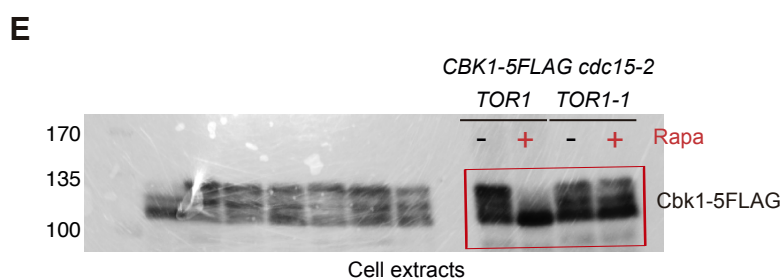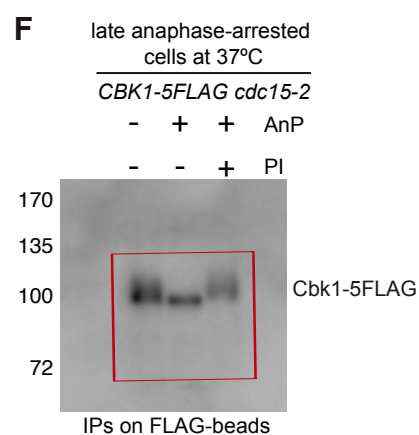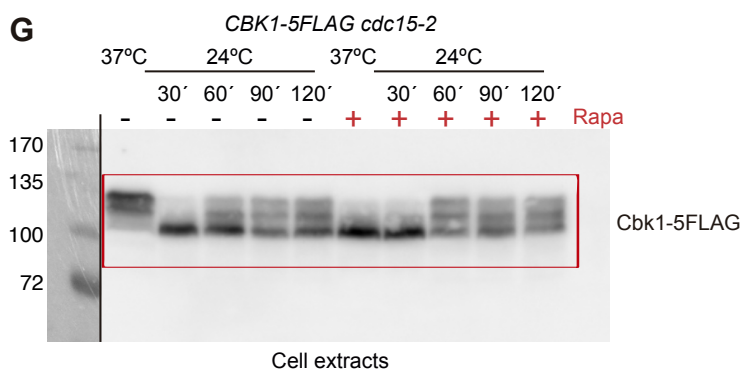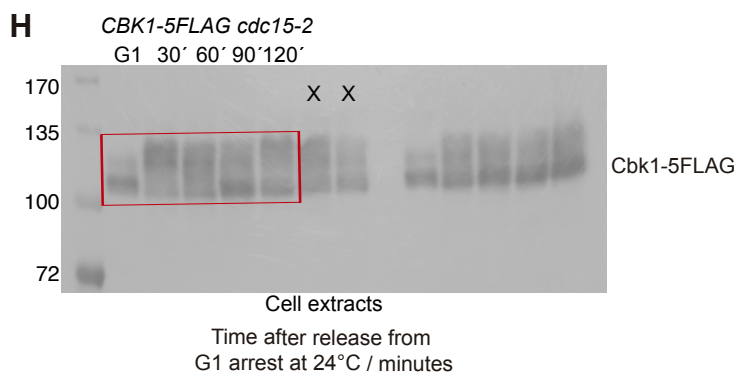

Supplement: S3 Raw Images — (PDF) [file pbio.3002263.s019.pdf]

**B**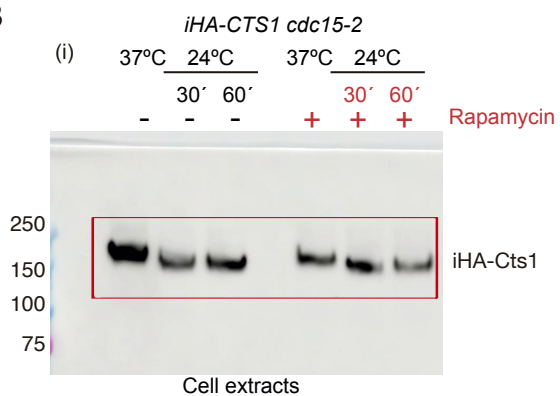**C**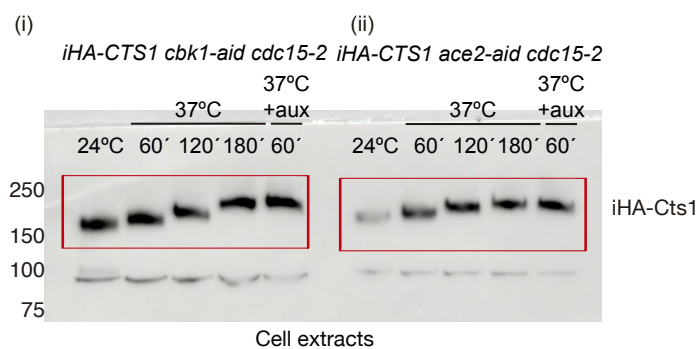

Supplement: S4 Raw Images — (PDF) [file pbio.3002263.s020.pdf]

**A**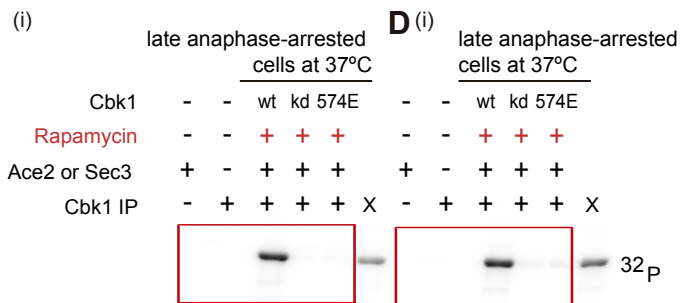**D**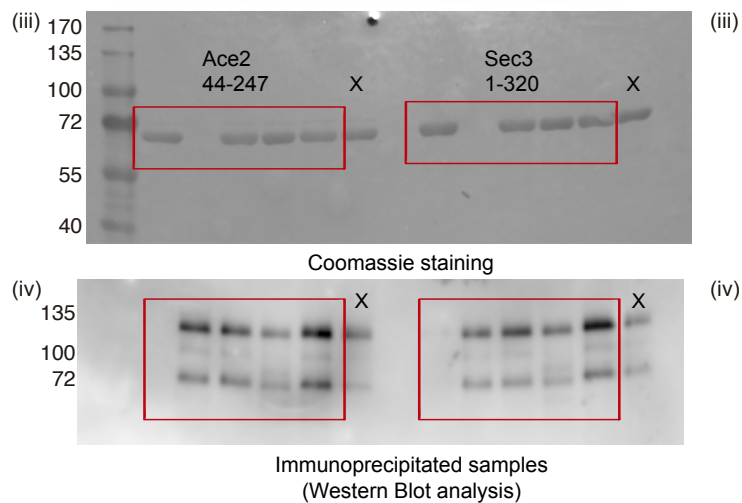**C**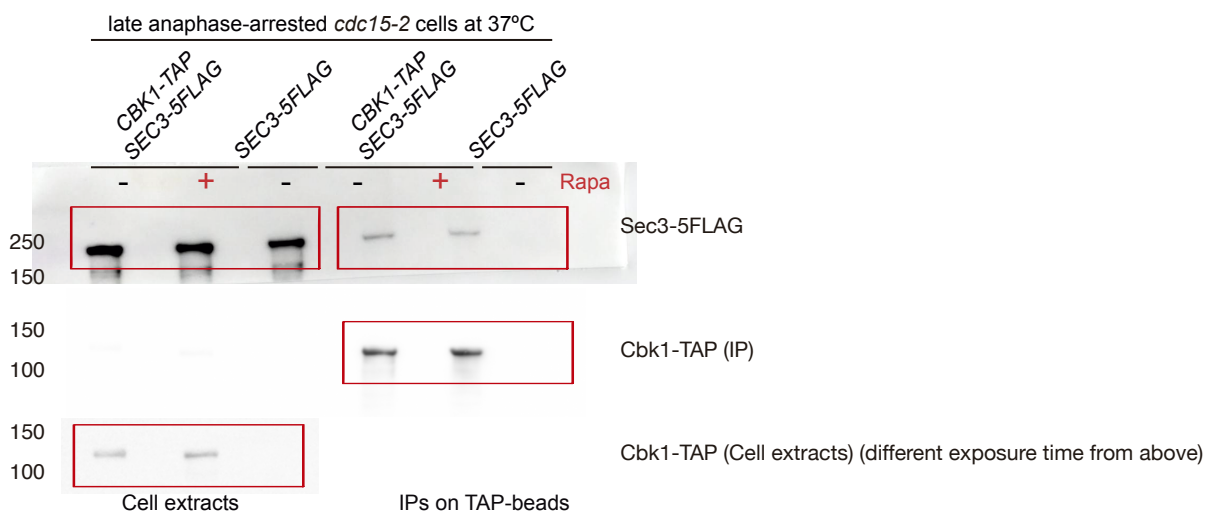**E**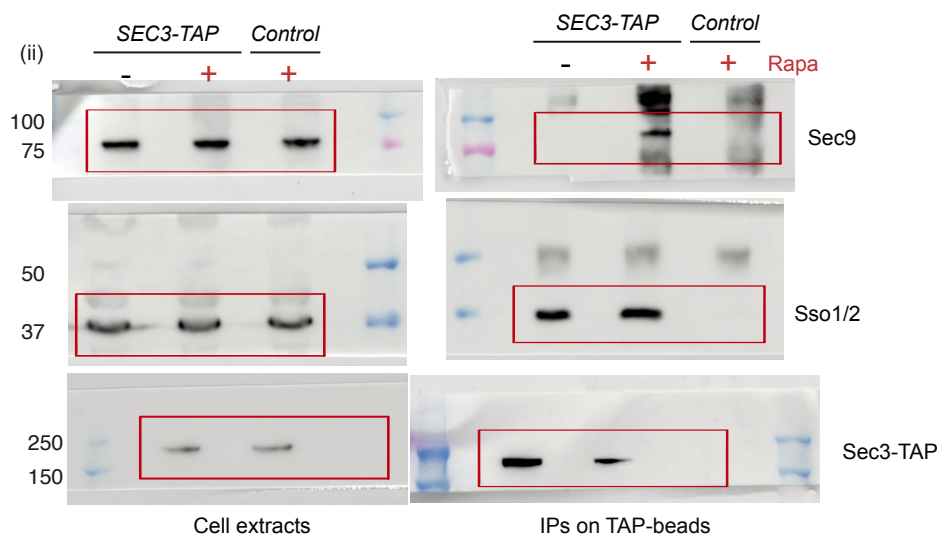

Supplement: S5 Raw Images — (PDF) [file pbio.3002263.s021.pdf]

**A**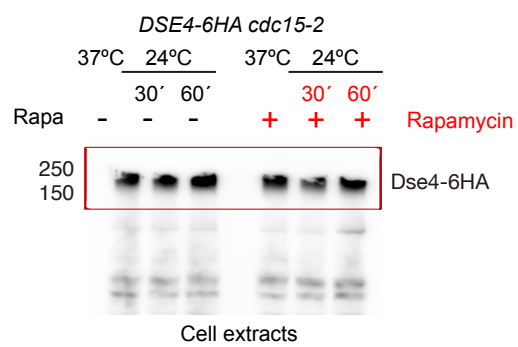**B**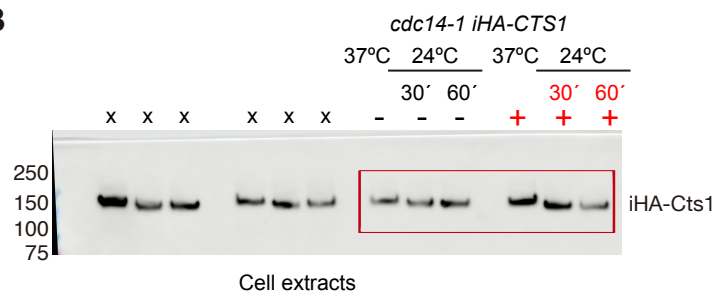

Supplement: S6 Raw Images — (PDF) [file pbio.3002263.s022.pdf]

**B**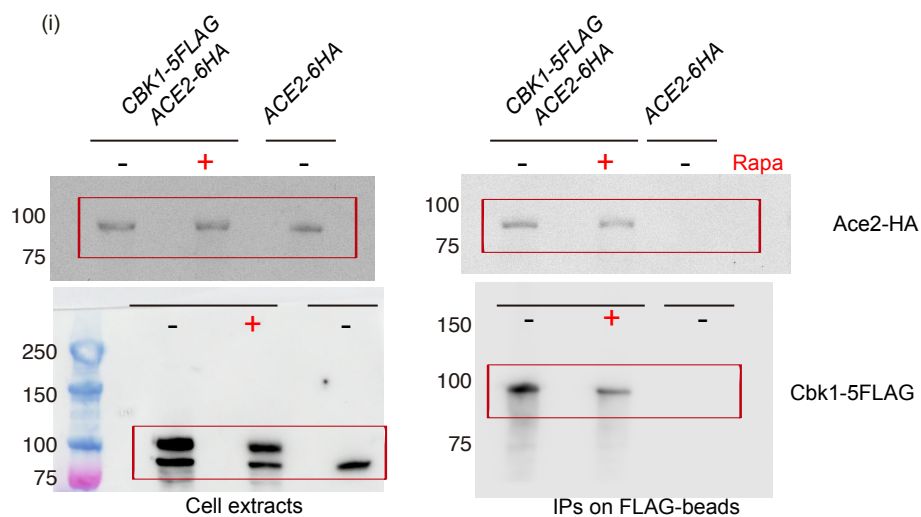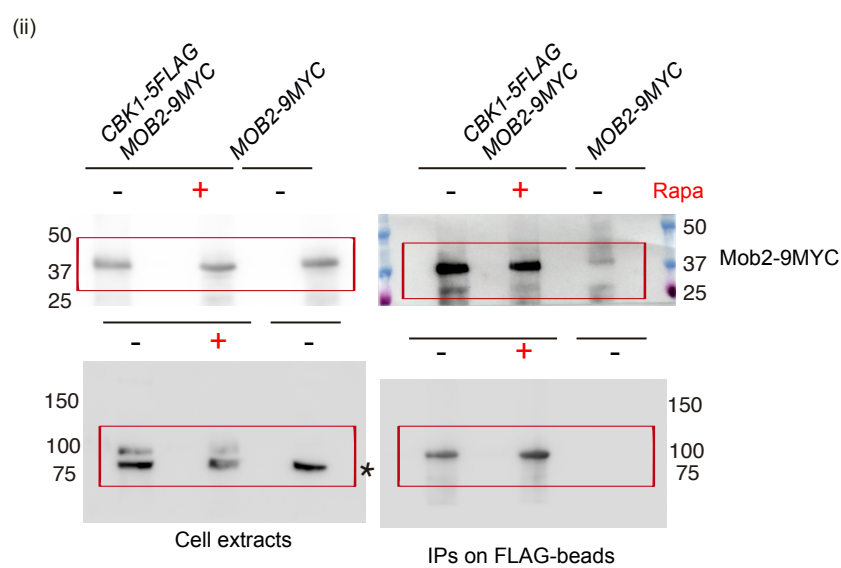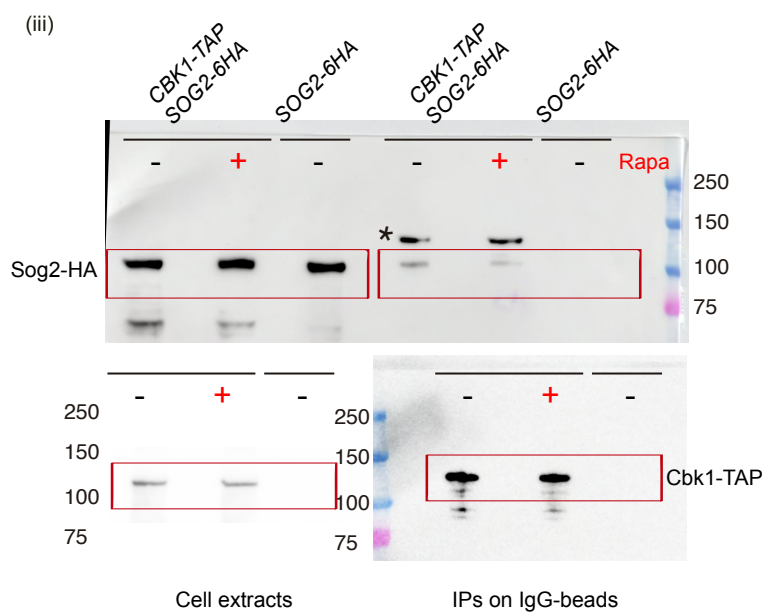

Supplement: S7 Raw Images — (PDF) [file pbio.3002263.s023.pdf]
