# Supplementary material for: TOR complex 1 negatively regulates NDR kinase Cbk1 to control cell separation in budding yeast
Source: PLoS Biol. 2023 Aug 30;21(8):e3002263. doi: 10.1371/journal.pbio.3002263 (PMC10468069; doi:10.1371/journal.pbio.3002263)
Supplement: S4 Fig — The indicated fungal species were compared by CLUSTAL O multiple sequence alignment. Kinase domain and activation loop are marked. Serines and threonines followed by proline in S. cerevisiae Cbk1 are indicated with an asterisk and the corresponding residue. Those residues were changed to glutamic acid to mimic phosphorylation (cbk1-6E) [42]. This mutant was used in this work. (PDF) [file pbio.3002263.s004.pdf]

A

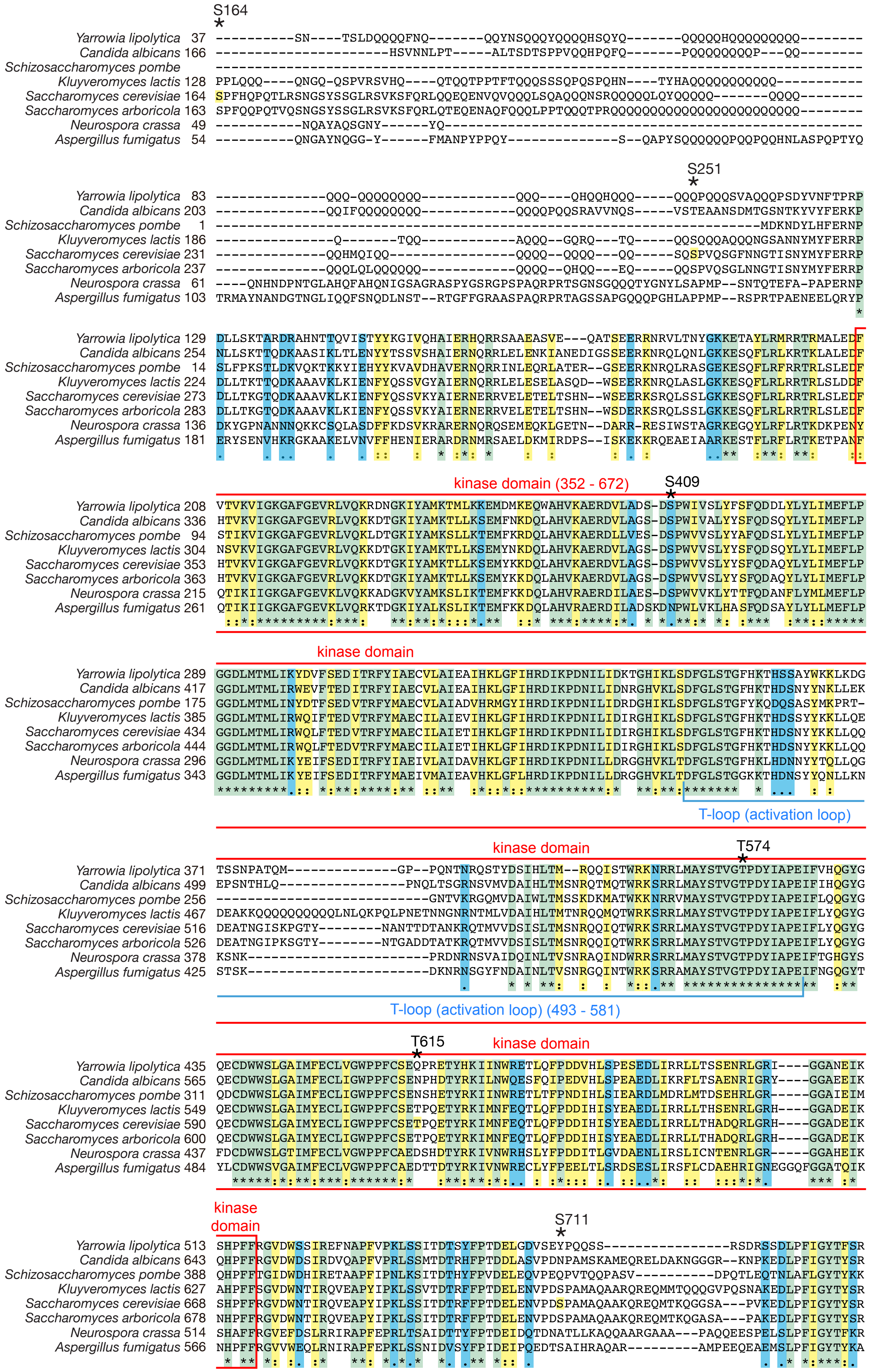
S164
S251
kinase domain (352 - 672)
S409
kinase domain
T-loop (activation loop)
kinase domain
T574
T-loop (activation loop) (493 - 581)
T615
kinase domain
kinase domain
S711
Yarrowia lipolytica
Candida albicans
Schizosaccharomyces pombe
Kluyveromyces lactis
Saccharomyces cerevisiae
Saccharomyces arboricola
Neurospora crassa
Aspergillus fumigatus
