# Supplementary material for: TOR complex 1 negatively regulates NDR kinase Cbk1 to control cell separation in budding yeast
Source: PLoS Biol. 2023 Aug 30;21(8):e3002263. doi: 10.1371/journal.pbio.3002263 (PMC10468069; doi:10.1371/journal.pbio.3002263)
Supplement: S8 Fig — (A) CBK1-GFP cdc15-2 (YMF3566) and CBK1-GFP TOR1-1 cdc15-2 (YMF3565) cells were grown in YPD and arrested in late anaphase by shifting the temperature to 37 °C before the addition of rapamycin for 20 min to both strains. In addition, DMSO was added to one half of CBK1-GFP cdc15-2. Untagged cdc15-2 (YMF3580) and TOR1-1 cdc15-2 cells (YMF3578) were used as controls. Cell extracts were prepared before the immunoprecipitation of Cbk1-GFP on ChromoTek GFP-Trap Magnetic beads. Isolated material was subjected to analysis by mass spectrometry. Numbers represent spectral average of key Cbk1 functional-related interactors in 3 independent replicates of the experiment. The average is calculated as the sum of all spectral counts for the 3 replicates divided by 3. We found Cbk1 interactions previously described: Mob2 [48] or components of the RAM pathway such as Tao3 [97], Sog2 [98], and Kic1 [99]. Besides, Cbk1 negative regulators Lre1 and Fir1 were also identified [5,43]. Besides, MS found Myo1, a component of the actomyosin ring [43]. Our results showed that Cbk1 interacts with the transcription factor Ace2 [47,48]. Moreover, Cbk1 was bound to Ssd1, an RNA-binding protein that represses the translation of cell wall remodelling proteins until Ssd1 is phosphorylated by Cbk1 [48,100]. Interestingly, TORC1 and Ssd1 have been described to collaborate to maintain cellular integrity [73]. We found interactions with paralogs serine/threonine kinases Kin1 and Kin2 [74,75]. Finally, we detected protein Yol036w of unknown function interacting with Cbk1 [40,74]. Details of other known Cbk1 interactors are shown in S2 Table. (B) CBK1-5FLAG ACE2-HA cdc15-2 (YMF3585) and control cells (YMF3587) (i), CBK1-5FLAG MOB2-9MYC cdc15-2 (YMF3838) and control cells (YMF3835) (ii), or CBK1-TAP SOG2-6HA cdc15-2 (YMF4235) and control cells (YMF4236) cells (iii) were grown in YPD and arrested in late anaphase by shifting to 37 °C before the addition of rapamycin for 20 min when indicated. Subsequently, p [file pbio.3002263.s008.pdf]

A

MS analysis of Cbk1-GFP IPs

Protein (Mw)	*cdc15-2*	*cdc15-2 TOR1-1* (+ rapamycin)	*cdc15-2* (+ rapamycin)	Control
Ace2 (87)	48.33	41.33	42.67	6.33
Cbk1 (87)	644.67	1142.33	786.67	20.67
Fir1 (99)	310.33	282	234.67	8.33
Kic1 (117)	9.33	9	1.67	0.33
Kin1 (120)	7	7	6.33	1
Kin2 (128)	83.33	94.33	68.67	8.67
Lre1 (65)	295.67	368	260	9.33
Mob2 (33)	301.67	464.67	361.67	14.67
Myo1 (224)	11.67	25.33	18	0
Sog2 (87)	10.67	10.33	3	0.33
Ssd1 (140)	45	40.33	47	9.33
Tao3 (270)	1469.33	1693.67	1342.33	43.00
Yol036w (84)	150.67	148.67	97	1.33

TORC1 ON (*cdc15-2*, *cdc15-2 TOR1-1*) · TORC1 OFF (*cdc15-2* + rapamycin)

Spectral average of 3 replicas

B

(i) late anaphase-arrested *cdc15-2* cells at 37ºC

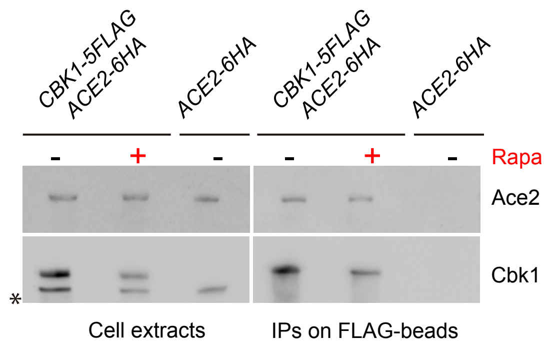

(ii) late anaphase-arrested *cdc15-2* cells at 37ºC

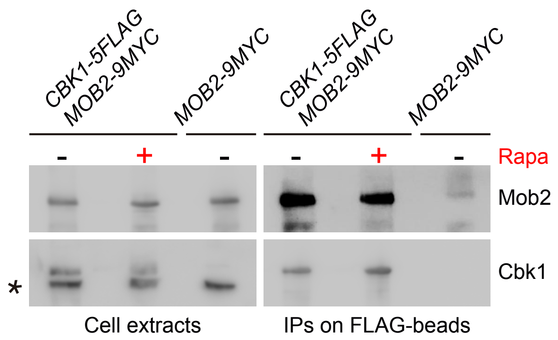

(iii) late anaphase-arrested *cdc15-2* cells at 37ºC

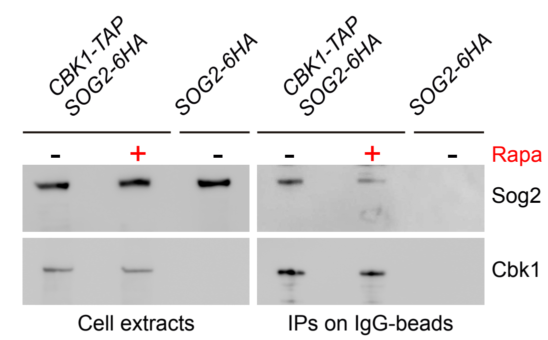

C

MS analysis of Cbk1-GFP IPs

Protein (Mw)	*cdc15-2*	*cdc15-2 TOR1-1* (+ rapamycin)	*cdc15-2* (+ rapamycin)	Control
Kog1 (178)	50.33	27	37.33	19.33
Lst8 (34)	13	4.67	6	3.33
Tco89 (89)	32	12.67	14.33	18.67
Tor1 (281)	121.67	51.33	58	40.00
Tor2 (281)	31.33	20.33	20.33	20.33

TORC1 ON (*cdc15-2*, *cdc15-2 TOR1-1*) · TORC1 OFF (*cdc15-2* + rapamycin)

Spectral average of 3 replicas
