# Supplementary material for: TOR complex 1 negatively regulates NDR kinase Cbk1 to control cell separation in budding yeast
Source: PLoS Biol. 2023 Aug 30;21(8):e3002263. doi: 10.1371/journal.pbio.3002263 (PMC10468069; doi:10.1371/journal.pbio.3002263)
Supplement: S4 Table — All based on W303. (DOC) [file pbio.3002263.s014.doc]

**S4 Table .** Strains used in this study (all based on W303).

| **Strain Name** | **Genotype** | **Sourc*e*** |
| --- | --- | --- |
| W303-1 | *MAT***a /** *MATα*  *ade2-1 / ade2-1 ura3-1 / ura3-1 his3-11,15 / his3-11,15 trp1-1 / trp1-1 leu2-3,112 / leu2-3,112 can1-100 / can1-100* | R. Rothstein |
| W303-1a | *MAT***a** *ade2-1 ura3-1 his3-11,15 trp1-1 leu2-3,112 can1-100* | R. Rothstein |
| YMF3154 | *MAT***a** *ade2-1 ura3-1 his3-11,15 trp1-1 leu2-3,112 can1-100*  *GLN3-9MYC (URA3)* | This study |
| YMF3162 | *MAT***a** *ade2-1 ura3-1 his3-11,15 trp1-1 leu2-3,112 can1-100*  *cdc15-2 INN1-yEGFP (kanMX)* | This study |
| YMF3178 | *MAT***a** *ade2-1 ura3-1 his3-11,15 trp1-1 leu2-3,112 can1-100*  *cdc15-2 GLN3-9MYC (URA3)* | This study |
| YMF3240 | *MAT***a** *ade2-1 ura3-1 his3-11,15 trp1-1 leu2-3,112 can1-100*  *cdc15-2 TOR1-1* | This study |
| YMF3260 | *MAT***a** *ade2-1 ura3-1 his3-11,15 trp1-1 leu2-3,112 can1-100*  *cdc15-2 TOR1-1 GLN3-9MYC (URA3MX)* | This study |
| YMF3262 | *MAT***a** *ade2-1 ura3-1 his3-11,15 trp1-1 leu2-3,112 can1-100*  *CLB2-9MYC (hphNT)* | This study |
| YMF3300 | *MAT***a** *ade2-1 ura3-1 his3-11,15 trp1-1 leu2-3,112 can1-100*  *cdc15-2 CBK1-5FLAG (hphNT) TOR1-1* | This study |
| YMF3302 | *MAT***a** *ade2-1 ura3-1 his3-11,15 trp1-1 leu2-3,112 can1-100*  *cdc15-2 CBK1-5FLAG (hphNT)* | This study |
| YMF3375 | *MAT***a** *ade2-1 ura3-1 his3-11,15 trp1-1 leu2-3,112 can1-100*  *cdc14-1* | This study |
| YMF3513 | *MAT***a** *ade2-1 ura3-1 his3-11,15 trp1-1 leu2-3,112 can1-100*  *cdc15-2 CBK1-GFP (kanMX)* | This study |
| YMF3546 | *MAT***a** *ade2-1 ura3-1 his3-11,15 trp1-1 leu2-3,112 can1-100*  *cdc15-2 CBK1-5FLAG (hphNT) pep4∆ (URA3)* | This study |
| YMF3565 | *MAT***a** *ade2-1 ura3-1 his3-11,15 trp1-1 leu2-3,112 can1-100*  *cdc15-2 CBK1-GFP (kanMX) TOR1-1 pep4∆ (URA3)* | This study |
| YMF3566 | *MAT***a** *ade2-1 ura3-1 his3-11,15 trp1-1 leu2-3,112 can1-100*  *cdc15-2 CBK1-GFP (kanMX) pep4∆ (URA3)* | This study |
| YMF3578 | *MAT***a** *ade2-1 ura3-1 his3-11,15 trp1-1 leu2-3,112 can1-100*  *cdc15-2 TOR1-1 pep4∆ (URA3)* | This study |
| YMF3580 | *MAT***a** *ade2-1 ura3-1 his3-11,15 trp1-1 leu2-3,112 can1-100*  *cdc15-2 pep4∆ (URA3)* | This study |
| YMF3585 | *MAT***a** *ade2-1 ura3-1 his3-11,15 trp1-1 leu2-3,112 can1-100*  *cdc15-2 CBK1-5FLAG (hphNT) ACE2-HA (kanMX) pep4∆ (URA3)* | This study |
| YMF3587 | *MAT***a** *ade2-1 ura3-1 his3-11,15 trp1-1 leu2-3,112 can1-100*  *cdc15-2 ACE2-HA (kanMX) pep4∆ (URA3)* | This study |
| YMF3606 | *MAT***a** *ade2-1 ura3-1 his3-11,15 trp1-1 leu2-3,112 can1-100*  *cdc15-2 HA3-TOR1 (URA3) pep4∆ (URA3)* | This study |
| YMF3608 | *MAT***a** *ade2-1 ura3-1 his3-11,15 trp1-1 leu2-3,112 can1-100*  *cdc15-2 CBK1-5FLAG (hphNT) HA3-TOR1 (URA3) pep4∆ (URA3)* | This study |
| YMF3657 | *MAT***a** *ade2-1 ura3-1 his3-11,15 trp1-1 leu2-3,112 can1-100*  *cbk1-aid (kanMX) TIR1 (URA3)* | This study |
| YMF3763 | *MAT***a** *ade2-1 ura3-1 his3-11,15 trp1-1 leu2-3,112 can1-100*  *cdc15-2 cbk1∆ (K.l.TRP1) pRS306-cbk1-6E (URA3)* | This study |
| YMF3764 | *MAT***a** *ade2-1 ura3-1 his3-11,15 trp1-1 leu2-3,112 can1-100*  *cdc15-2 cbk1∆ (K.l.TRP1) pRS306-cbk1-9A (URA3)* | This study |
| YMF3782 | *MAT***a** *ade2-1 ura3-1 his3-11,15 trp1-1 leu2-3,112 can1-100*  *cdc15-2 SCH9-6HA (K.l.TRP1)* | This study |
| YMF3835 | *MAT***a** *ade2-1 ura3-1 his3-11,15 trp1-1 leu2-3,112 can1-100*  *cdc15-2 HA3-TOR1 (URA3) MOB2-9MYC (HIS3MX) pep4∆ (URA3)* | This study |
| YMF3838 | *MAT***a** *ade2-1 ura3-1 his3-11,15 trp1-1 leu2-3,112 can1-100*  *cdc15-2 CBK1-5FLAG (hphNT) HA3-TOR1 (URA3) MOB2-9MYC (HIS3MX) pep4∆ (URA3)* | This study |
| YMF3845 | *MAT***a** *ade2-1 ura3-1 his3-11,15 trp1-1 leu2-3,112 can1-100*  *cdc15-2 iHA-CTS1* | This study |
| YMF3857 | *MAT***a** *ade2-1 ura3-1 his3-11,15 trp1-1 leu2-3,112 can1-100*  *cdc15-2 lre1∆ (HIS3)* | This study |
| YMF3866 | *MAT***a** *ade2-1 ura3-1 his3-11,15 trp1-1 leu2-3,112 can1-100*  *cdc15-2 cbk1-aid (kanMX) TIR1 (HIS3)* | This study |
| YMF3869 | *MAT***a** *ade2-1 ura3-1 his3-11,15 trp1-1 leu2-3,112 can1-100*  *cdc15-2 cbk1∆ (K.l.TRP1) pRS306-CBK1 (URA3)* |  |
| YMF3901 | *MAT***a** *ade2-1 ura3-1 his3-11,15 trp1-1 leu2-3,112 can1-100*  *CDC15-6HA (K.l.TRP1) CBK1-5FLAG (hphNT) pep4∆ (ADE2)* | This study |
| YMF3905 | *MAT***a** *ade2-1 ura3-1 his3-11,15 trp1-1 leu2-3,112 can1-100*  *cdc15-2 cbk1-aid (kanMX) TIR1 (HIS3) pRS306-cbk1-5E-E164S (URA3)* | This study |
| YMF3906 | *MAT***a** *ade2-1 ura3-1 his3-11,15 trp1-1 leu2-3,112 can1-100*  *cdc15-2 cbk1-aid (kanMX) TIR1 (HIS3) pRS306-cbk1-5E-E615T (URA3)* | This study |
| YMF3907 | *MAT***a** *ade2-1 ura3-1 his3-11,15 trp1-1 leu2-3,112 can1-100*  *cdc15-2 cbk1-aid (kanMX) TIR1 (HIS3) pRS306-cbk1-5E-E711S (URA3)* | This study |
| YMF3910 | *MAT***a** *ade2-1 ura3-1 his3-11,15 trp1-1 leu2-3,112 can1-100*  *cdc15-2 cbk1-aid (kanMX) TIR1 (HIS3) pRS306-cbk1-5E-E251S (URA3)* | This study |
| YMF3944 | *MAT***a** *ade2-1 ura3-1 his3-11,15 trp1-1 leu2-3,112 can1-100*  *cdc15-2 cbk1-aid (kanMX) TIR1 (HIS3) pRS306-cbk1-T574E (URA3)* | This study |
| YMF3969 | *MAT***a** *ade2-1 ura3-1 his3-11,15 trp1-1 leu2-3,112 can1-100*  *cdc15-2 iHA-CTS1 ace2-aid (hphNT) TIR1 (HIS3)* | This study |
| YMF3976 | *MAT***a** *ade2-1 ura3-1 his3-11,15 trp1-1 leu2-3,112 can1-100*  *cdc15-2 TUB1-GFP (K.l.TRP1)* | This study |
| YMF3991 | *MAT***a** *ade2-1 ura3-1 his3-11,15 trp1-1 leu2-3,112 can1-100*  *cdc15-2 iHA-CTS1 cbk1-aid (kanMX)TIR1 (HIS3)* | This study |
| YMF3995 | *MAT***a** *ade2-1 ura3-1 his3-11,15 trp1-1 leu2-3,112 can1-100*  *cdc15-2 cbk1-aid (kanMX) TIR1 (HIS3) pRS306-cbk1-5E-E409S (URA3)* | This study |
| YMF3997 | *MAT***a** *ade2-1 ura3-1 his3-11,15 trp1-1 leu2-3,112 can1-100*  *cdc15-2 cbk1-aid (kanMX) TIR1 (HIS3) pRS306-cbk1-5E-E574T (URA3)* | This study |
| YMF4002 | *MAT***a** *ade2-1 ura3-1 his3-11,15 trp1-1 leu2-3,112 can1-100*  *cdc15-2 SEC8-GFP (K.l.TRP1)* | This study |
| YMF4003 | *MAT***a** *ade2-1 ura3-1 his3-11,15 trp1-1 leu2-3,112 can1-100*  *cdc15-2 SEC3-GFP (K.l.TRP1)* | This study |
| YMF4019 | *MAT***a** *ade2-1 ura3-1 his3-11,15 trp1-1 leu2-3,112 can1-100*  *cdc15-2 cbk1-aid (kanMX) TIR1 (HIS3) pRS306-cbk1-6E (URA3)* | This study |
| YMF4021 | *MAT***a** *ade2-1 ura3-1 his3-11,15 trp1-1 leu2-3,112 can1-100*  *cdc15-2 cbk1-aid (kanMX) TIR1 (HIS3) pRS306-cbk1-T574E (URA3) 3GFP-RAS2 (LEU2)* | This study |
| YMF4029 | *MATalpha* *ade2-1 ura3-1 his3-11,15 trp1-1 leu2-3,112 can1-100*  *cdc15-2 TIR1 (HIS3) DSE4-6HA (K.l.TRP1)* | This study |
| YMF4047 | *MATalpha* *ade2-1 ura3-1 his3-11,15 trp1-1 leu2-3,112 can1-100*  *cdc15-2 cbk1-aid (kanMX) TIR1 (HIS3) pRS306-cbk1-D475A (URA3)* | This study |
| YMF4079 | *MATalpha* *ade2-1 ura3-1 his3-11,15 trp1-1 leu2-3,112 can1-100*  *cdc15-2 cbk1-aid (kanMX) TIR1 (HIS3) pRS306-cbk1-6E (URA3) 3GFP-RAS2 (LEU2)* | This study |
| YMF4082 | *MATalpha* *ade2-1 ura3-1 his3-11,15 trp1-1 leu2-3,112 can1-100*  *cdc15-2 cbk1-aid (kanMX) TIR1 (HIS3) pRS306-CBK1 (URA3)* | This study |
| YMF4088 | *MAT***a** *ade2-1 ura3-1 his3-11,15 trp1-1 leu2-3,112 can1-100*  *cdc14-1 iHA-CTS1* | This study |
| YMF4109 | *MAT***a** *ade2-1 ura3-1 his3-11,15 trp1-1 leu2-3,112 can1-100*  *CDC15-6HA (K.l.TRP1) pep4∆ (ADE2)* | This study |
| YMF4113 | *MATalpha* *ade2-1 ura3-1 his3-11,15 trp1-1 leu2-3,112 can1-100*  *cdc15-2 cbk1-aid (kanMX) TIR1 (HIS3) HA3-TOR1 (URA3) pRS306-CBK1-5FLAG (URA3) pep4∆ (URA3)* | This study |
| YMF4115 | *MAT***a** *ade2-1 ura3-1 his3-11,15 trp1-1 leu2-3,112 can1-100*  *cdc15-2 cbk1-aid (kanMX) TIR1 (HIS3) HA3-TOR1 (URA3) pRS306-cbk1-T574E-5FLAG (URA3) pep4∆ (URA3)* | This study |
| YMF4145 | *MAT***a** *ade2-1 ura3-1 his3-11,15 trp1-1 leu2-3,112 can1-100*  *cdc15-2 CTS1-GFPEnvy (kanMX)* | This study |
| YMF4159 | *MAT***a/α** *ade2-1/ ade2-1 ura3-1/ ura3-1 his3-11,15/ his3-11,15 trp1-1/ trp1-1 leu2-3,112/ leu2-3,112 can1-100/ can1-100*  *cdc15-2 sec3∆ (HIS3)* | This study |
| YMF4180 | *MAT***a** *ade2-1 ura3-1 his3-11,15 trp1-1 leu2-3,112 can1-100*  *cdc15-2 cbk1-aid (kanMX) TIR1 (HIS3) pRS306-cbk1-D475A-5FLAG (URA3) pep4∆ (URA3)* | This study |
| YMF4191 | *MAT***a** *ade2-1 ura3-1 his3-11,15 trp1-1 leu2-3,112 can1-100*  *cdc15-2 cbk1-aid (kanMX)TIR1 (HIS3) pRS306-cbk1-T574A (URA3)* | This study |
| YMF4218 | *MAT***a** *ade2-1 ura3-1 his3-11,15 trp1-1 leu2-3,112 can1-100*  *cdc15-2 sso1-1 sso2∆ (LEU2) CTS1-GFPEnvy (kanMX)* | This study |
| YMF4221 | *MAT***a** *ade2-1 ura3-1 his3-11,15 trp1-1 leu2-3,112 can1-100*  *cdc15-2 CBK1-5FLAG (hphNT) SEC3-TAP (HIS3) pep4∆ (URA3)* | This study |
| YMF4231 | *MAT***a** *ade2-1 ura3-1 his3-11,15 trp1-1 leu2-3,112 can1-100*  *cdc14-1 CTS1-GFPEnvy (kanMX)* | This study |
| YMF4235 | *MAT***a** *ade2-1 ura3-1 his3-11,15 trp1-1 leu2-3,112 can1-100*  *cdc15-2 SOG2-6HA (ClonNAT) CBK1-TAP (kanMX) pep4∆ (URA3)* | This study |
| YMF4236 | *MAT***a** *ade2-1 ura3-1 his3-11,15 trp1-1 leu2-3,112 can1-100*  *cdc15-2 SOG2-6HA (ClonNAT) pep4∆ (URA3)* | This study |
| YMF4238 | *MAT***a** *ade2-1 ura3-1 his3-11,15 trp1-1 leu2-3,112 can1-100*  *cdc15-2 SEC3-5FLAG (HIS3) CBK1-TAP (kanMX) pep4∆ (URA3)* | This study |
| YMF4240 | *MAT***a** *ade2-1 ura3-1 his3-11,15 trp1-1 leu2-3,112 can1-100*  *cdc15-2 SEC3-5FLAG (HIS3) pep4∆ (URA3)* | This study |
| YMF4250 | *MAT***a** *ade2-1 ura3-1 his3-11,15 trp1-1 leu2-3,112 can1-100*  *cdc15-2 cbk1-aid (kanMX) TIR1 (HIS3) pRS306-cbk1-T574E (URA3) CTS1-GFPEnvy (kanMX)* | This study |
| YMF4252 | *MAT***a** *ade2-1 ura3-1 his3-11,15 trp1-1 leu2-3,112 can1-100*  *cdc15-2 CBK1-5FLAG (hphNT) SEC3-TAP (HIS3) SSO2-6HA (K.l.TRP1) pep4∆ (URA3)* | This study |
| YMF4279 | *MAT***a** *ade2-1 ura3-1 his3-11,15 trp1-1 leu2-3,112 can1-100*  *cdc15-2 cbk1∆ (K.l.TRP1) pRS306- cbk1-5E-E409S (URA3)* | This study |
| YMF4280 | *MAT***a** *ade2-1 ura3-1 his3-11,15 trp1-1 leu2-3,112 can1-100*  *cdc15-2 cbk1∆ (K.l.TRP1) pRS306-cbk1-5E-E574T (URA3)* | This study |
| YMF4298 | *MAT***a** *ade2-1 ura3-1 his3-11,15 trp1-1 leu2-3,112 can1-100*  *cdc14-1 GLN3-9MYC (URA3MX)* | This study |
| YMF4338 | *MAT***a** *ade2-1 ura3-1 his3-11,15 trp1-1 leu2-3,112 can1-100*  *cdc15-2 cbk1-aid (kanMX) TIR1 (HIS3) pRS306-cbk1- S174E-S177E-S187E (URA3)* | This study |
| YMF4339 | *MAT***a** *ade2-1 ura3-1 his3-11,15 trp1-1 leu2-3,112 can1-100*  *cdc15-2 cbk1-aid (kanMX) TIR1 (HIS3) pRS306-cbk1-S348E (URA3)* | This study |
| YMF4362 | *MAT***a** *ade2-1 ura3-1 his3-11,15 trp1-1 leu2-3,112 can1-100*  *cdc15-2 cbk1-aid (kanMX) TIR1 (HIS3) pRS306-cbk1-S693E (URA3)* | This study |
| YMF4456 | *MAT***a** *ade2-1 ura3-1 his3-11,15 trp1-1 leu2-3,112 can1-100*  *cdc14-1 GLN3-9MYC (URA3MX) TOR1-1* | This study |
| YMF4471 | *MAT***a** *ade2-1 ura3-1 his3-11,15 trp1-1 leu2-3,112 can1-100*  *GAL-SIC1∆NT (URA3) GLN3-9MYC (URA3MX)* | This study |
| YMF4500 | *MAT***a** *ade2-1 ura3-1 his3-11,15 trp1-1 leu2-3,112 can1-100*  *cdc15-2 ATG13-5FLAG (HIS3)* | This study |
| YMF4509 | *MAT***a/α** *ade2-1/ ade2-1 ura3-1/ ura3-1 his3-11,15/ his3-11,15 trp1-1/ trp1-1 leu2-3,112/ leu2-3,112 can1-100/ can1-100*  *NFLAG-SSO1 (hphNT) sso2∆ (LEU2)* | This study |
| YMF4511 | *MAT***a/α** *ade2-1/ ade2-1 ura3-1/ ura3-1 his3-11,15/ his3-11,15 trp1-1/ trp1-1 leu2-3,112/ leu2-3,112 can1-100/ can1-100*  *NFLAG∆1-146-SSO1 (hphNT) sso2∆ (LEU2)* | This study |
| YMF4612 | *MAT***a** *ade2-1 ura3-1 his3-11,15 trp1-1 leu2-3,112 can1-100*  *sec3-aid (hphNT) TIR1 (URA3)* | This study |
| YMS3792 | *MAT***a** *ade2-1 ura3-1 his3-11,15 trp1-1 leu2-3,112 can1-100*  *cdc15-2 fir1∆ (HIS3)* | This study |
| CC5909 | *MATalpha* *ade2-1 ura3-1 his3-11,15 trp1-1 leu2-3,112 can1-100*  *GAL-CDC20 (LEU2)* | K. Labib |
| CC2274 | *MAT***a** *ade2-1 ura3-1 his3-11,15 trp1-1 leu2-3,112 can1-100*  *cdc15-2* | K. Labib |
| CC6296 | *MAT***a** *ade2-1 ura3-1 his3-11,15 trp1-1 leu2-3,112 can1-100*  *cdc14-1 3GFP-RAS2 (URA3,kanMX)* | K. Labib |
| CC6298 | *MAT***a** *ade2-1 ura3-1 his3-11,15 trp1-1 leu2-3,112 can1-100*  *cdc15-2 3GFP-RAS2 (URA3,kanMX)* | K. Labib |
| CC6441 | *MAT***a** *ade2-1 ura3-1 his3-11,15 trp1-1 leu2-3,112 can1-100*  *cdc14-1 cdc15-2* | K. Labib |
| YJW15 | *MAT***a** *ade2-1 ura3-1 his3-11,15 trp1-1 leu2-3,112 can1-100*  *TIR1 (URA3)* | M. Kanemaki |
| EW1447 | *MAT***a** *ade2-1 ura3-1 his3-11,15 trp1-1 leu2-3,112 can1-100*  *cdc15-2 CBK1-T743E (kanMX)* | E. Weiss |
| H603 | *MAT***a** *ade2-1 ura3-1 his3-11,15 trp1-1 leu2-3,112 can1-100*  *sso1∆ (HIS3) sso2-1* | J. Jantti |
| H1239 | *MAT***a** *ade2-1 ura3-1 his3-11,15 trp1-1 leu2-3,112 can1-100*  *sso1-1 sso2∆ (LEU2)* | J. Jantti |
| H1269 | *MAT***a** *ade2-1 ura3-1 his3-11,15 trp1-1 leu2-3,112 can1-100*  *sso1-1 sso2-1* | J. Jantti |
