# Supplementary material for: TOR complex 1 negatively regulates NDR kinase Cbk1 to control cell separation in budding yeast
Source: PLoS Biol. 2023 Aug 30;21(8):e3002263. doi: 10.1371/journal.pbio.3002263 (PMC10468069; doi:10.1371/journal.pbio.3002263)
Supplement: S5 Table — (DOC) [file pbio.3002263.s015.doc]

**S5 Table.** Plasmids used in this study.

| **Plasmid** | **Sourc*e*** |
| --- | --- |
| *pRS316-cbk1-6E* | E. Weiss  *pELW889* |
| *pRS306-cbk1-6E* | This study |
| *pRS306-CBK1* | This study |
| *pRS306-CBK1-5FLAG* | This study |
| *pRS306-cbk1-5E-E164S* | This study |
| *pRS306-cbk1-5E-E251S* | This study |
| *pRS306-cbk1-5E-E409S* | This study |
| *pRS306-cbk1-5E-E574T* | This study |
| *pRS306-cbk1-5E-E615T* | This study |
| *pRS306-cbk1-5E-E711S* | This study |
| *pRS306-cbk1-T574E* | This study |
| *pRS306-cbk1-T574E-5FLAG* | This study |
| *pRS306-cbk1-S570A* | This study |
| *pRS306-cbk1-D475A-5FLAG* | This study |
| pMAL-C2x-MBP-Ace2_44-247_ | G. Pereira |
| pET28-HisMBP-Sec3-N320 | This study |
