# Supplementary material for: TOR complex 1 negatively regulates NDR kinase Cbk1 to control cell separation in budding yeast
Source: PLoS Biol. 2023 Aug 30;21(8):e3002263. doi: 10.1371/journal.pbio.3002263 (PMC10468069; doi:10.1371/journal.pbio.3002263)
Supplement: S6 Table — (DOCX) [file pbio.3002263.s016.docx]

**S6 Table.** Oligonucleotides used in this study.

| **Oligo Sequence** | **Plasmid** | **Cloning strategy** |
| --- | --- | --- |
| GACAGTAAGAGAATTATGCAGTGCTGCCATAACCATGAG | *pRS306-cbk1-5E-E164S* | Gibson assembly |
| CAAAATTTAAATGGCAGTTCTTCTAGTAGTCCGTTCCACCAACCGCAAACGCTACG | *pRS306-cbk1-5E-E164S* | Gibson assembly |
| CTCATGGTTATGGCAGCACTGCATAATTCTCTTACTGTC | *pRS306-cbk1-5E-E164S* | Gibson assembly |
| CGTAGCGTTTGCGGTTGGTGGAACGGACTACTAGAAGAACTGCCATTTAAATTTTG | *pRS306-cbk1-5E-E164S* | Gibson assembly |
| GACAGTAAGAGAATTATGCAGTGCTGCCATAACCATGAG | *pRS306-cbk1-5E-E251S* | Gibson assembly |
| GCAGCAACAACAGCAGCAGCAATCACAATCTCCCGTTCAGAGCGGCTTTAATAATGG | *pRS306-cbk1-5E-E251S* | Gibson assembly |
| CTCATGGTTATGGCAGCACTGCATAATTCTCTTACTGTC | *pRS306-cbk1-5E-E251S* | Gibson assembly |
| CCATTATTAAAGCCGCTCTGAACGGGAGATTGTGATTGCTGCTGCTGTTGTTGCTGC | *pRS306-cbk1-5E-E251S* | Gibson assembly |
| GACAGTAAGAGAATTATGCAGTGCTGCCATAACCATGAG | *pRS306-cbk1-5E-E409S* | Gibson assembly |
| GAAAGGGATGTTCTGGCTGGAAGTGATTCTCCATGGGTGGTTTCGCTATATTACTC | *pRS306-cbk1-5E-E409S* | Gibson assembly |
| CTCATGGTTATGGCAGCACTGCATAATTCTCTTACTGTC | *pRS306-cbk1-5E-E409S* | Gibson assembly |
| GAGTAATATAGCGAAACCACCCATGGAGAATCACTTCCAGCCAGAACATCCCTTTC | *pRS306-cbk1-5E-E409S* | Gibson assembly |
| GACAGTAAGAGAATTATGCAGTGCTGCCATAACCATGAG | *pRS306-cbk1-5E-E574T* | Gibson assembly |
| CGTTTAATGGCATATTCTACCGTAGGTACACCAGATTATATTGCTCCTGAGATATT | *pRS306-cbk1-5E-E574T* | Gibson assembly |
| CTCATGGTTATGGCAGCACTGCATAATTCTCTTACTGTC | *pRS306-cbk1-5E-E574T* | Gibson assembly |
| AATATCTCAGGAGCAATATAATCTGGTGTACCTACGGTAGAATATGCCATTAAACG | *pRS306-cbk1-5E-E574T* | Gibson assembly |
| GACAGTAAGAGAATTATGCAGTGCTGCCATAACCATGAG | *pRS306-cbk1-5E-E615T* | Gibson assembly |
| GATTGGTTGGCCTCCATTCTGTTCCGAAACTCCACAGGAAACGTACAGAAAAATCATG | *pRS306-cbk1-5E-E615T* | Gibson assembly |
| CTCATGGTTATGGCAGCACTGCATAATTCTCTTACTGTC | *pRS306-cbk1-5E-E615T* | Gibson assembly |
| CATGATTTTTCTGTACGTTTCCTGTGGAGTTTCGGAACAGAATGGAGGCCAACCAATC | *pRS306-cbk1-5E-E615T* | Gibson assembly |
| GACAGTAAGAGAATTATGCAGTGCTGCCATAACCATGAG | *pRS306-cbk1-5E-E711S* | Gibson assembly |
| CCAACCGATGAGTTGGAGAATGTTCCAGATTCCCCAGCTATGGCACAAGCTGCCAAAC | *pRS306-cbk1-5E-E711S* | Gibson assembly |
| CTCATGGTTATGGCAGCACTGCATAATTCTCTTACTGTC | *pRS306-cbk1-5E-E711S* | Gibson assembly |
| GTTTGGCAGCTTGTGCCATAGCTGGGGAATCTGGAACATTCTCCAACTCATCGGTTGG | *pRS306-cbk1-5E-E711S* | Gibson assembly |
| TTGGCTACACTTACTCCAGATTTGACTATTTGACAAGAAAAAATGCGTTGCGTACGCTGCAGGTCGAC | *pRS306-CBK1-5FLAG* | Gibson assembly |
| GCTTCTTTTCACCACCCAATTCGAAGTGCTTGGGGGGATCCACTAGTTCTAGAGCGGCC | *pRS306-CBK1-5FLAG* | Gibson assembly |
| GGCCGCTCTAGAACTAGTGGATCCCCCCAAGCACTTCGAATTGGGTGGTGAAAAGAAGC | *pRS306-CBK1-5FLAG* | Gibson assembly |
| GTCGACCTGCAGCGTACGCAACGCATTTTTTCTTGTCAAATAGTCAAATCTGGAGTAAG | *pRS306-CBK1-5FLAG* | Gibson assembly |
| GACAGTAAGAGAATTATGCAGTGCTGCCATAACCATGAG | *pRS306-cbk1-T574E* | Gibson assembly |
| CGTCGTTTAATGGCATATTCTACCGTAGGTgaACCgGATTATATTGCTCCTGAGATATT | *pRS306-cbk1-T574E* | Gibson assembly |
| CTCATGGTTATGGCAGCACTGCATAATTCTCTTACTGTC | *pRS306-cbk1-T574E* | Gibson assembly |
| AATATCTCAGGAGCAATATAATCcGGTtcACCTACGGTAGAATATGCCATTAAACGACG | *pRS306-cbk1-T574E* | Gibson assembly |
| TTGGCTACACTTACTCCAGATTTGACTATTTGACAAGAAAAAATGCGTTGCGTACGCTGCAGGTCGAC | *pRS306-cbk1-T574E-5FLAG* | Gibson assembly |
| GCTTCTTTTCACCACCCAATTCGAAGTGCTTGGGGGGATCCACTAGTTCTAGAGCGGCC | *pRS306-cbk1-T574E-5FLAG* | Gibson assembly |
| GGCCGCTCTAGAACTAGTGGATCCCCCCAAGCACTTCGAATTGGGTGGTGAAAAGAAGC | *pRS306-cbk1-T574E-5FLAG* | Gibson assembly |
| GTCGACCTGCAGCGTACGCAACGCATTTTTTCTTGTCAAATAGTCAAATCTGGAGTAAG | *pRS306-cbk1-T574E-5FLAG* | Gibson assembly |
| GACAGTAAGAGAATTATGCAGTGCTGCCATAACCATGAG | *pRS306-cbk1-S570A* | Gibson assembly |
| GGAGAAAATCACGTCGTTTAATGGCATATgCTACCGTAGGTACACCAGATTATATTGC | *pRS306-cbk1-S570A* | Gibson assembly |
| CTCATGGTTATGGCAGCACTGCATAATTCTCTTACTGTC | *pRS306-cbk1-S570A* | Gibson assembly |
| GCAATATAATCTGGTGTACCTACGGTAGcATATGCCATTAAACGACGTGATTTTCTCC | *pRS306-cbk1-S570A* | Gibson assembly |
| CGTGCTTTCATCACGCGCTTCCTCTCGGCGATACCGTCGACCTCGAGGGGGGGCCCG | *pRS306-CBK1* | Gibson assembly |
| GCTTCTTTTCACCACCCAATTCGAAGTGCTTGGGGGGATCCACTAGTTCTAGAGCGGCC | *pRS306-CBK1* | Gibson assembly |
| GGCCGCTCTAGAACTAGTGGATCCCCCCAAGCACTTCGAATTGGGTGGTGAAAAGAAGC | *pRS306-CBK1* | Gibson assembly |
| CGGGCCCCCCCTCGAGGTCGACGGTATCGCCGAGAGGAAGCGCGTGATGAAAGCACG | *pRS306-CBK1* | Gibson assembly |
| GACAGTAAGAGAATTATGCAGTGCTGCCATAACCATGAG | *pRS306-cbk1-D475A-5FLAG* | Gibson assembly |
| CCATTCATAAATTAGGATTCATTCACAGAGcTATTAAACCAGATAATATT  TTGATCGATA | *pRS306-cbk1-D475A-5FLAG* | Gibson assembly |
| CTCATGGTTATGGCAGCACTGCATAATTCTCTTACTGTC | *pRS306-cbk1-D475A-5FLAG* | Gibson assembly |
| TATCGATCAAAATATTATCTGGTTTAATAgCTCTGTGAATGAATCCTAAT  TTATGAATGG | *pRS306-cbk1-D475A-5FLAG* | Gibson assembly |
| GCCCTGGAAGTACAGGTTTTCGCCGCTGC | *pET28-HisMBP-Sec3_1-320_* | Gibson assembly |
| CACCACCACCACCACCACTGAGATCCGGCTGCTAAC | *pET28-HisMBP-Sec3_1-320_* | Gibson assembly |
| GGCGAAAACCTGTACTTCCAGGGCATGAGGTCCTCGAAGTCTCCGTTTAAAAGGAAGTC | *pET28-HisMBP-Sec3_1-320_* | Gibson assembly |
| GATCTCAGTGGTGGTGGTGGTGGTGTTATGTTTGAATGTATAGAGTAATTAGAGATTTAA | *pET28-HisMBP-Sec3_1-320_* | Gibson assembly |
