# Supplementary material for: TOR complex 1 negatively regulates NDR kinase Cbk1 to control cell separation in budding yeast
Source: PLoS Biol. 2023 Aug 30;21(8):e3002263. doi: 10.1371/journal.pbio.3002263 (PMC10468069; doi:10.1371/journal.pbio.3002263)
Supplement: S1 File — (ZIP) [file pbio.3002263.s024.zip › 2A.pdf]

New Composite 6 - Plot Sheet 2

CD169_1.2
[Ungated] IP PE-A / SSC-A

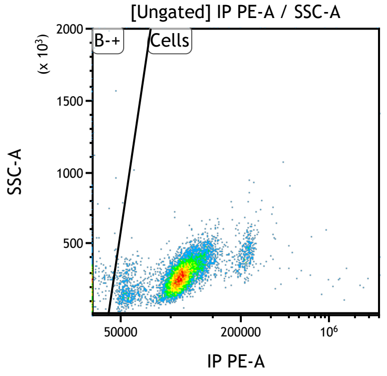

CD169_1.2
[Cells] FSC-A / SSC-A

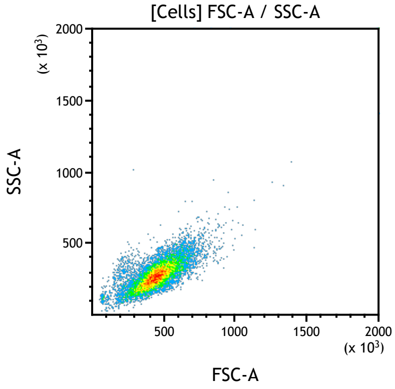

CD169_1.2
[Cells] IP PE-A

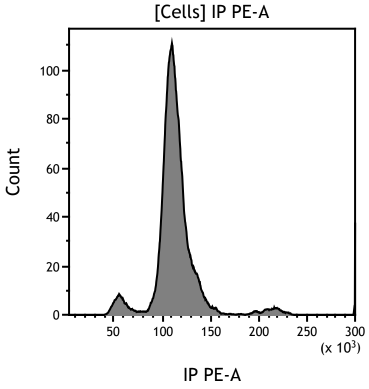

New Composite 6 - Plot Sheet 3

CD169_1.3
[Ungated] IP PE-A / SSC-A

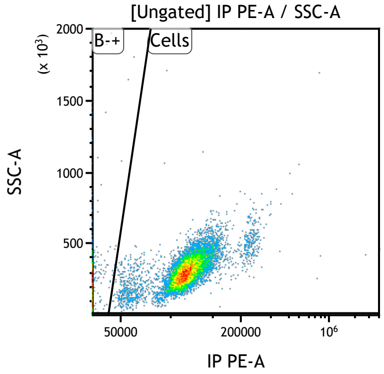

CD169_1.3
[Cells] FSC-A / SSC-A

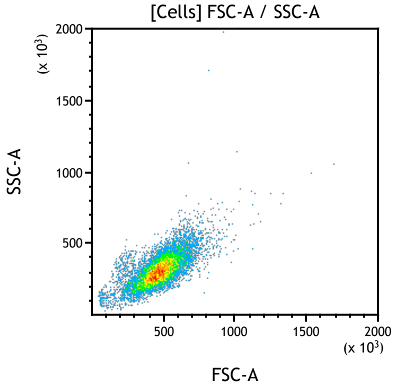

CD169_1.3
[Cells] IP PE-A

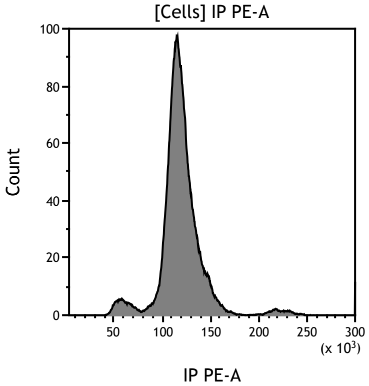

New Composite 6 - Plot Sheet 4

CD169_1.4
[Ungated] IP PE-A / SSC-A

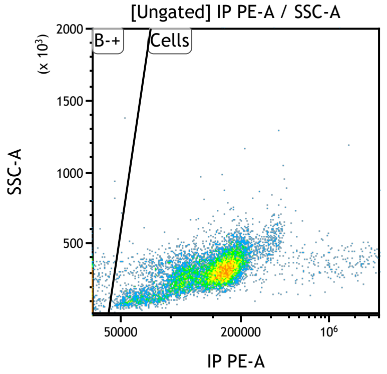

CD169_1.4
[Cells] FSC-A / SSC-A

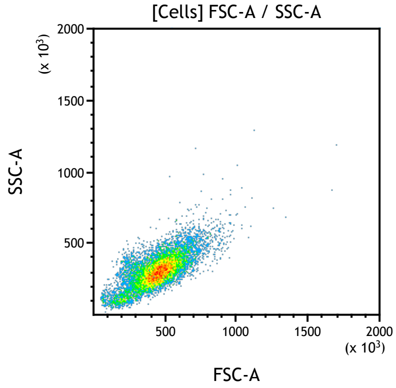

CD169_1.4
[Cells] IP PE-A

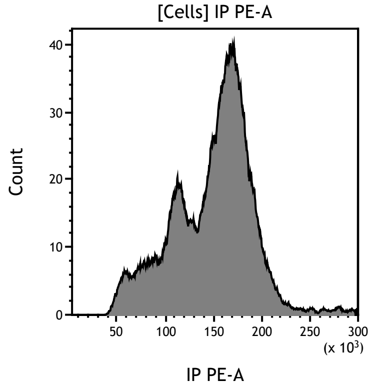

New Composite 6 - Plot Sheet 5

CD169_1.5
[Ungated] IP PE-A / SSC-A

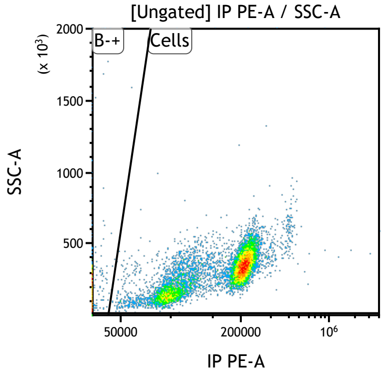

CD169_1.5
[Cells] FSC-A / SSC-A

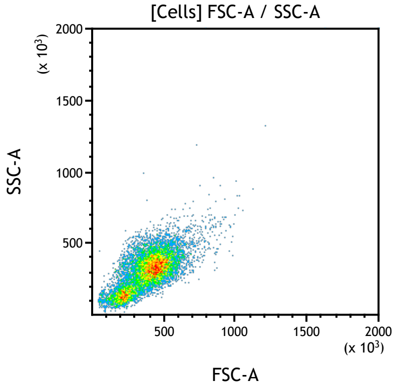

CD169_1.5
[Cells] IP PE-A

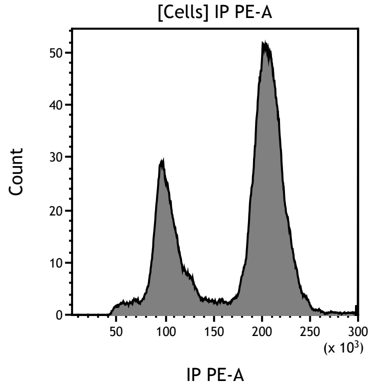

New Composite 6 - Plot Sheet 6

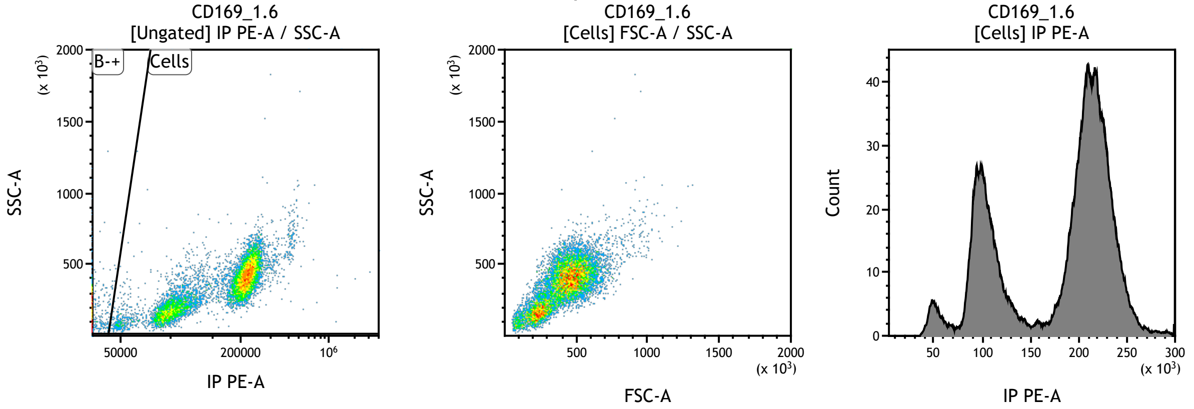

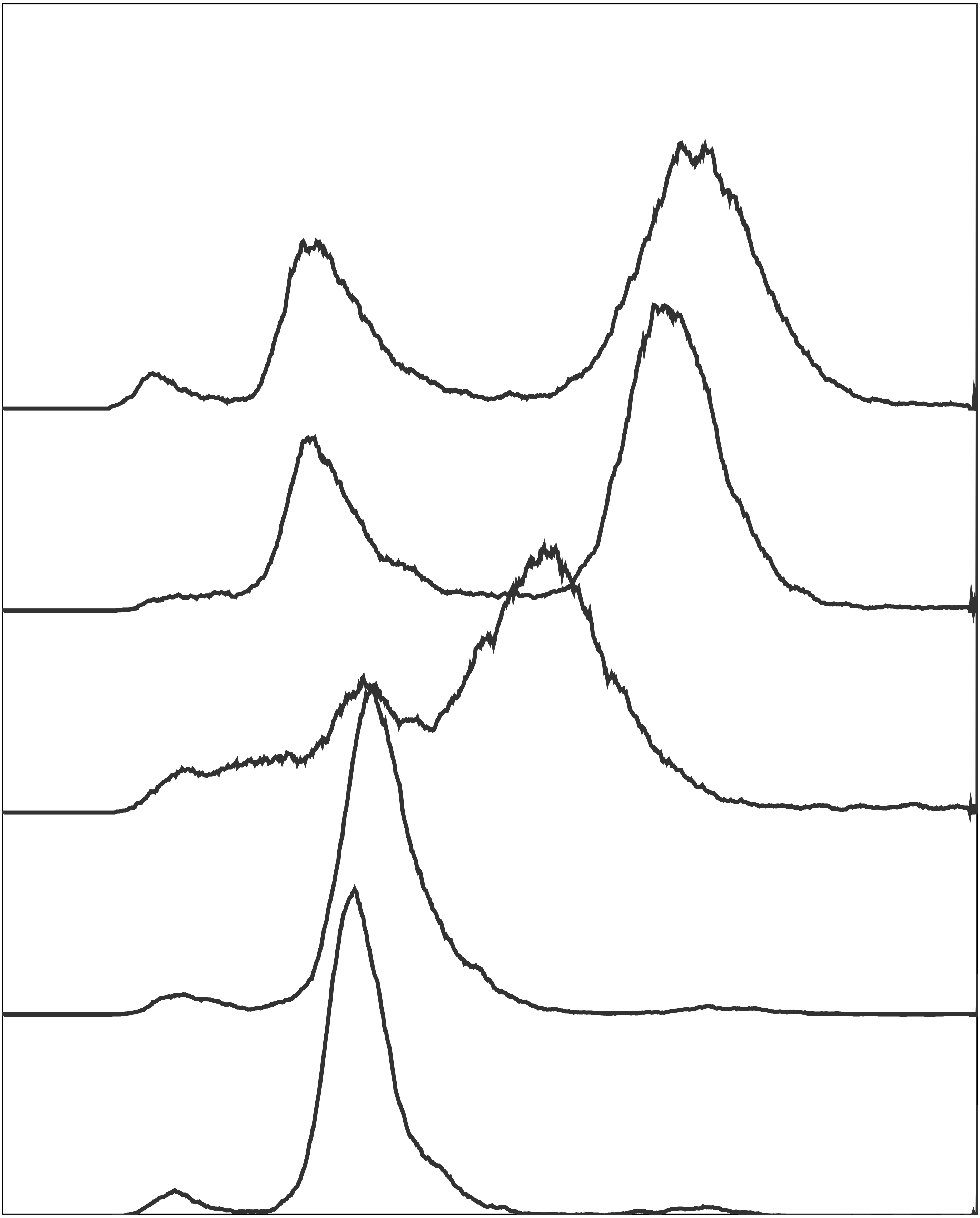

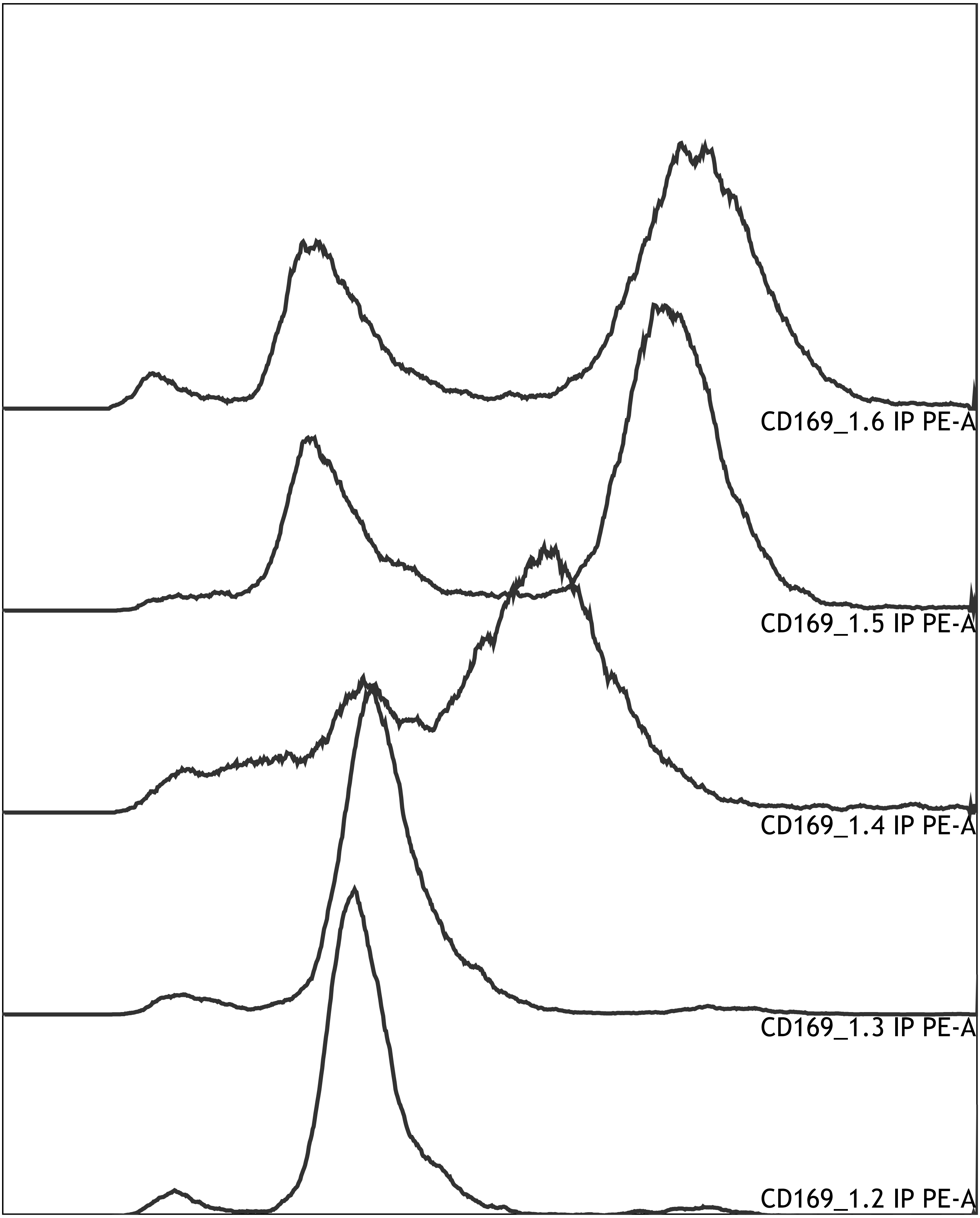
CD169_1.6 IP PE-A
CD169_1.5 IP PE-A
CD169_1.4 IP PE-A
CD169_1.3 IP PE-A
CD169_1.2 IP PE-A

New Composite 1 - Plot Sheet 2

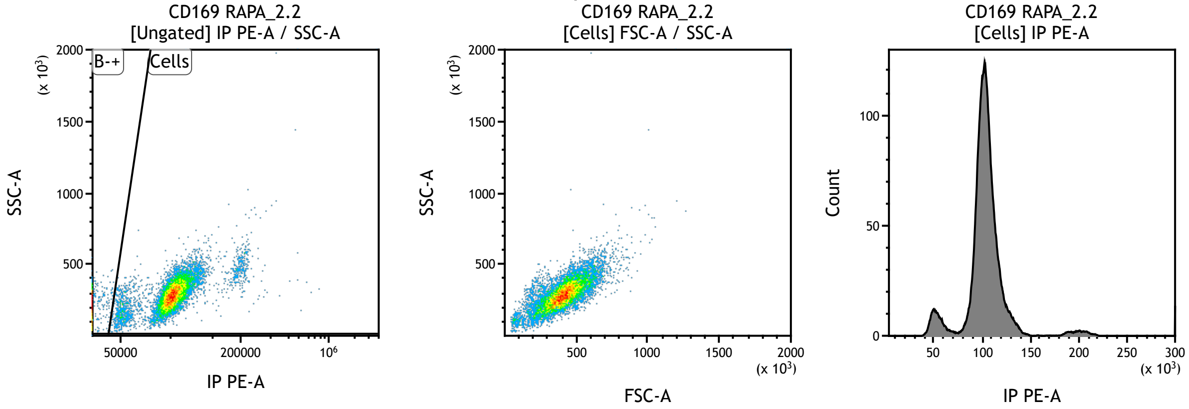

New Composite 1 - Plot Sheet 3

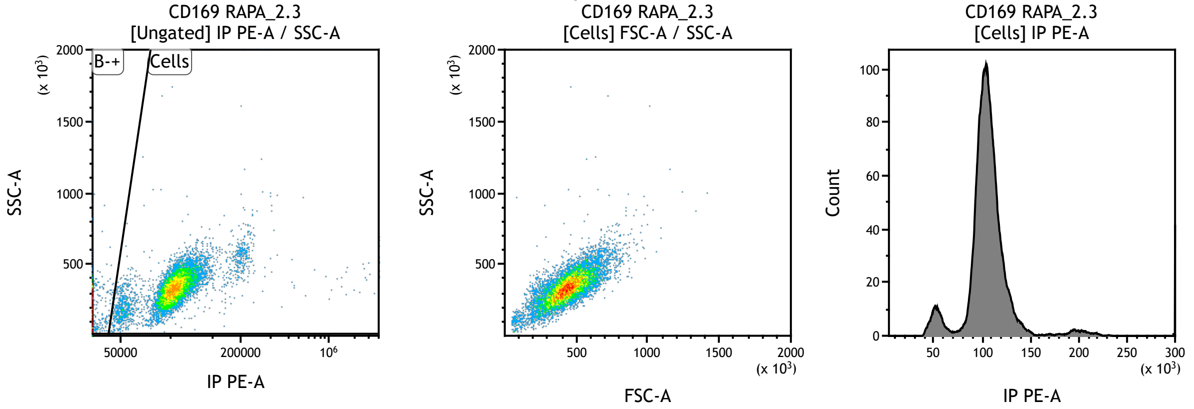

New Composite 1 - Plot Sheet 4

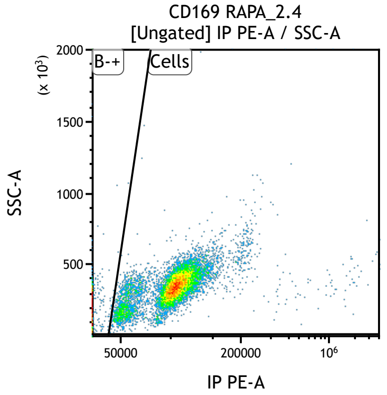

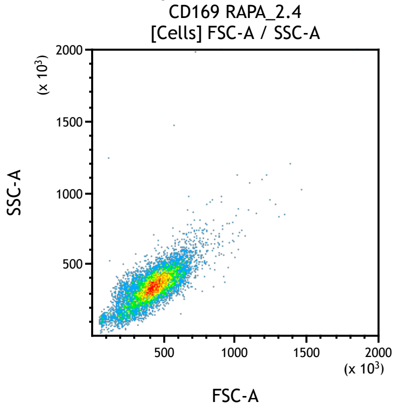

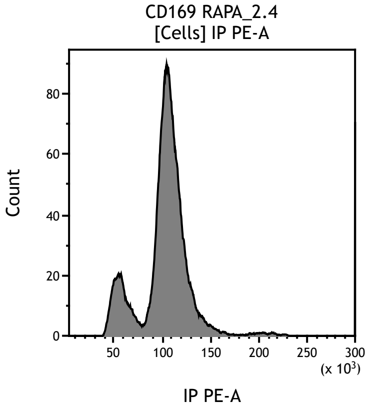

New Composite 1 - Plot Sheet 5

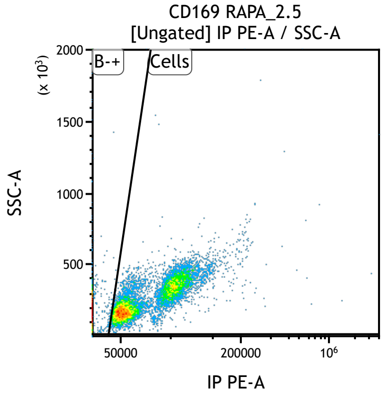

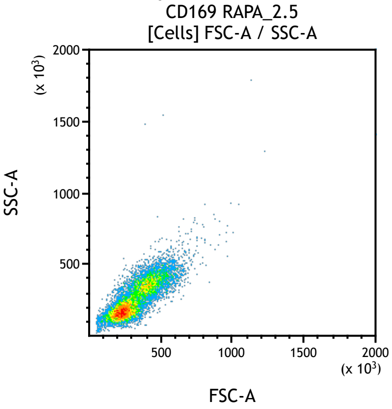

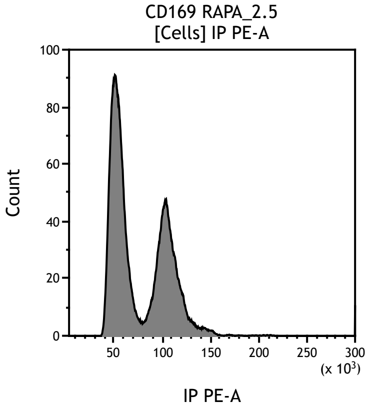

New Composite 1 - Plot Sheet 6

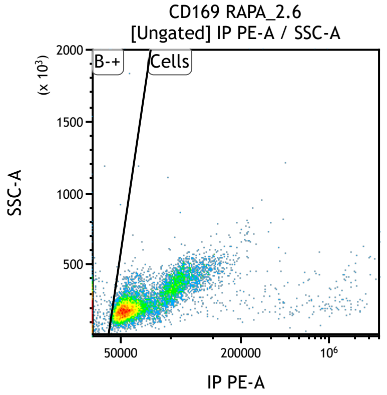

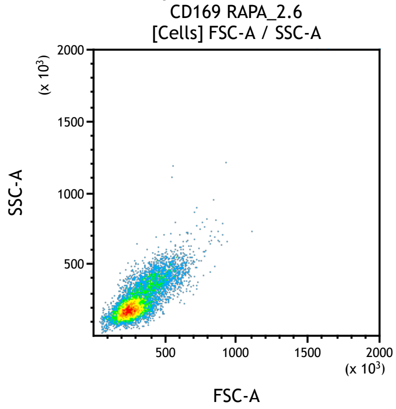

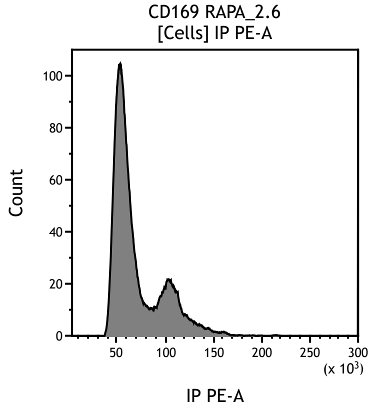

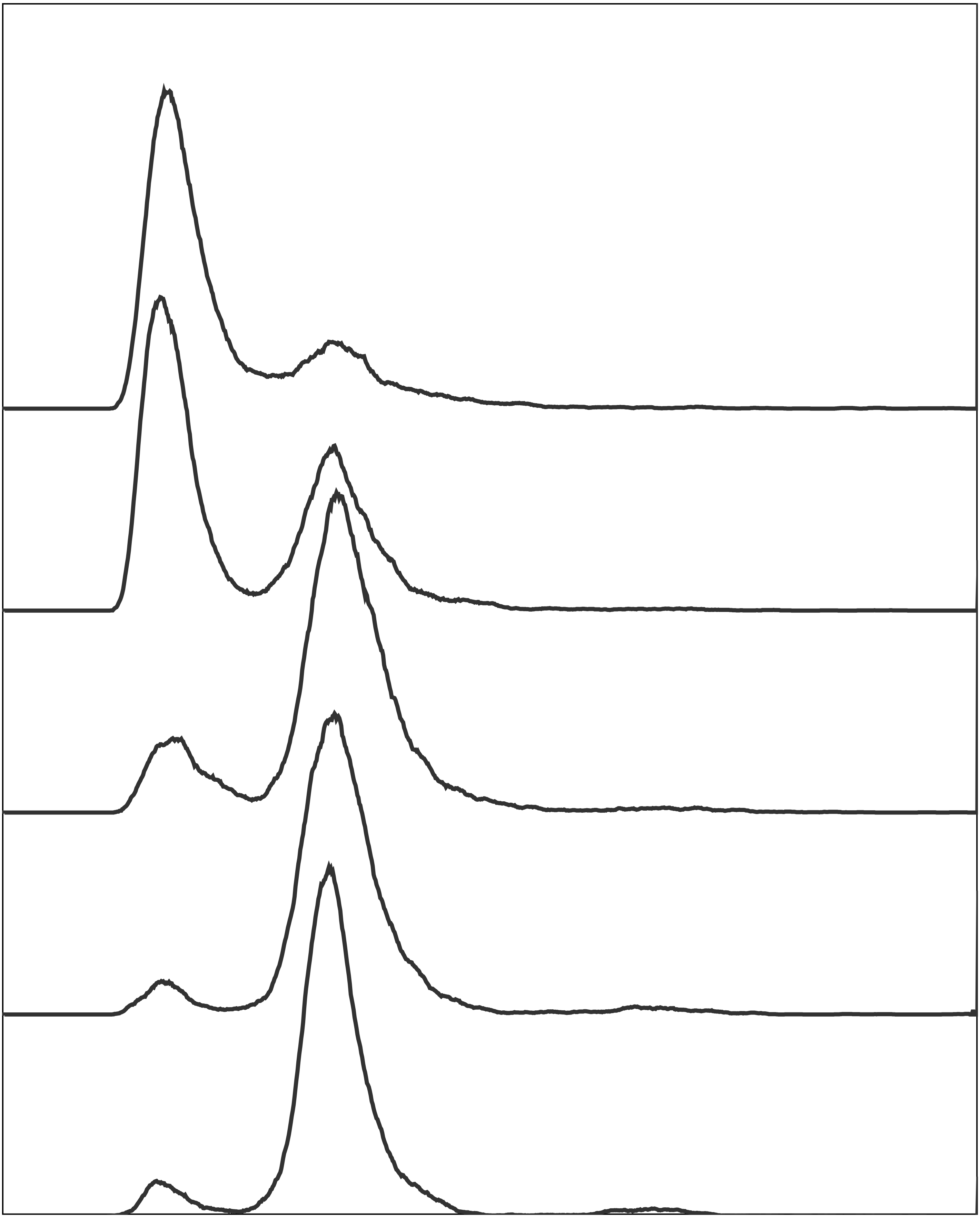

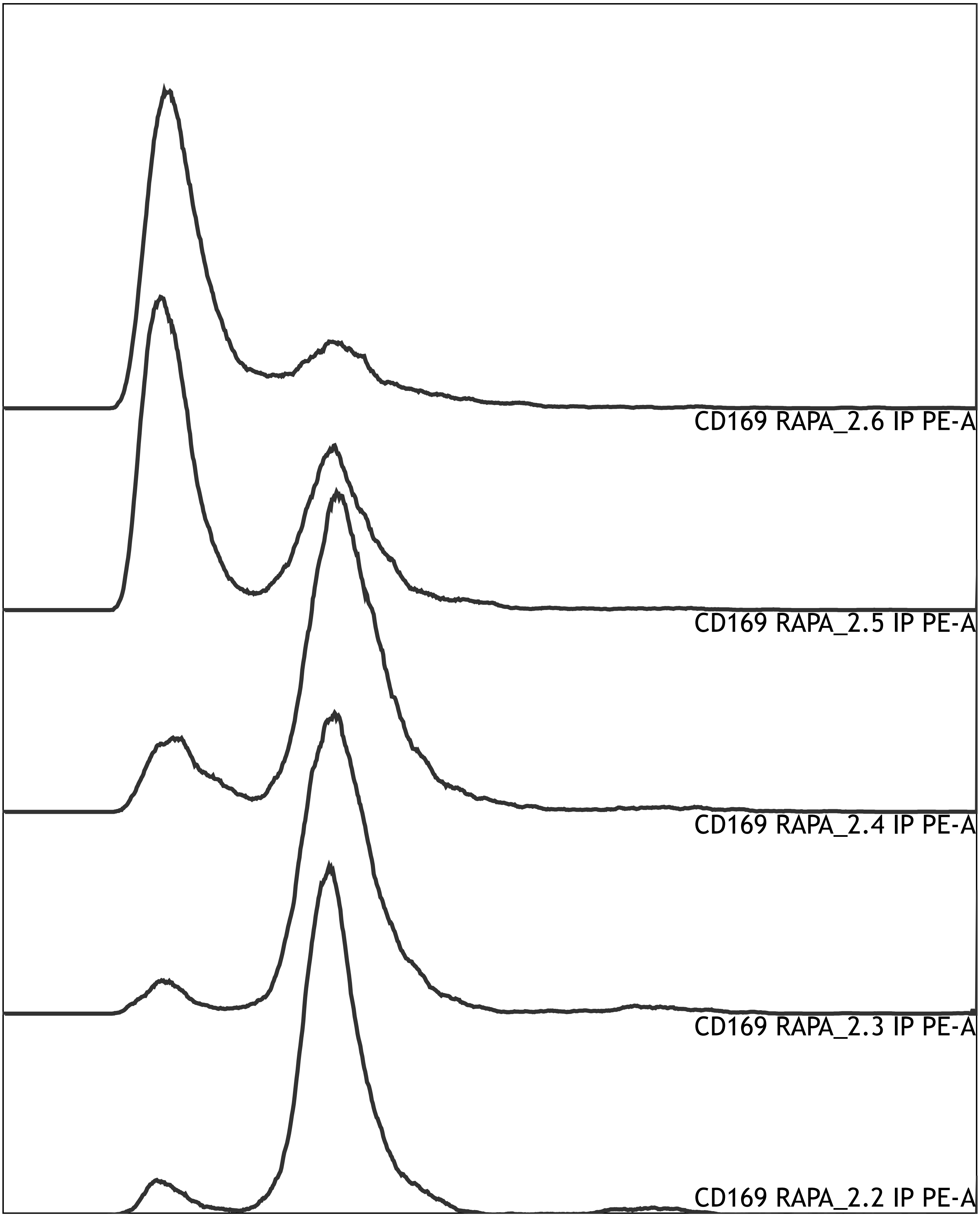
CD169 RAPA_2.6 IP PE-A
CD169 RAPA_2.5 IP PE-A
CD169 RAPA_2.4 IP PE-A
CD169 RAPA_2.3 IP PE-A
CD169 RAPA_2.2 IP PE-A
