# Supplementary material for: TOR complex 1 negatively regulates NDR kinase Cbk1 to control cell separation in budding yeast
Source: PLoS Biol. 2023 Aug 30;21(8):e3002263. doi: 10.1371/journal.pbio.3002263 (PMC10468069; doi:10.1371/journal.pbio.3002263)
Supplement: S1 File — (ZIP) [file pbio.3002263.s024.zip › 2B.pdf]

New Composite 6 - Plot Sheet 2

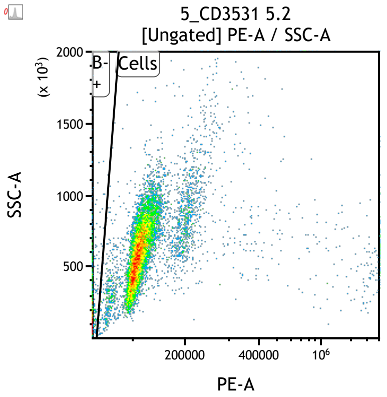

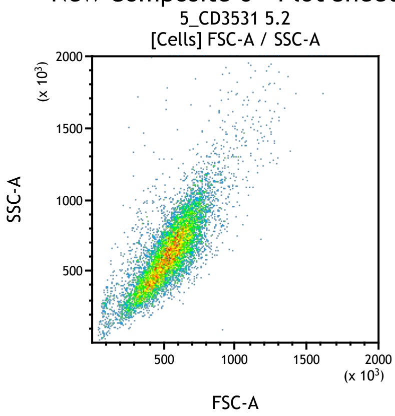

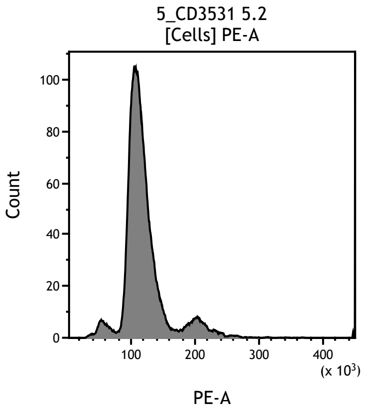

New Composite 6 - Plot Sheet 3

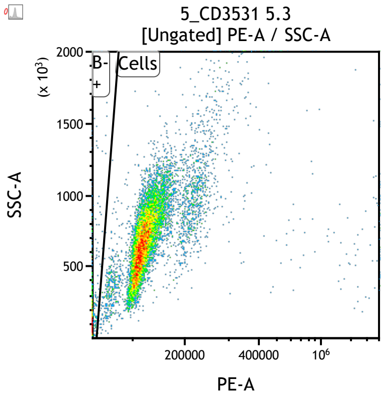

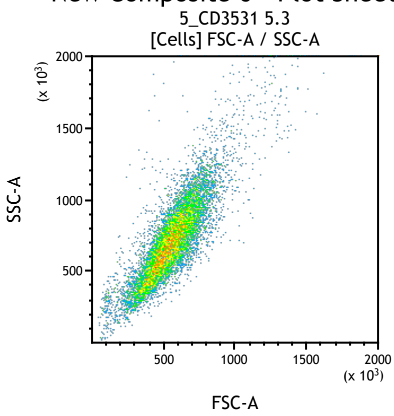

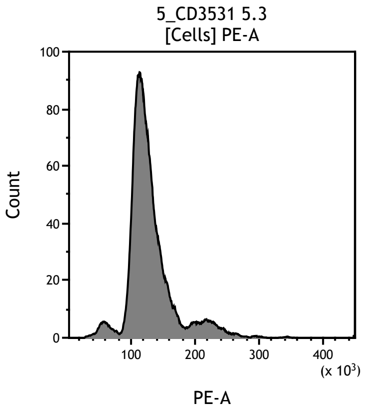

New Composite 6 - Plot Sheet 4

5_CD3531 5.4
[Ungated] PE-A / SSC-A

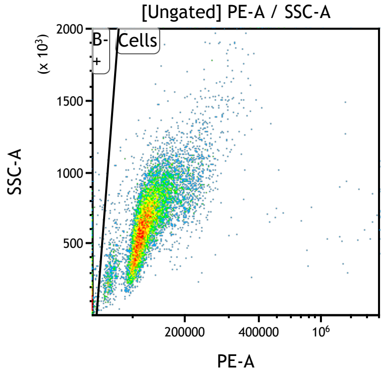

5_CD3531 5.4
[Cells] FSC-A / SSC-A

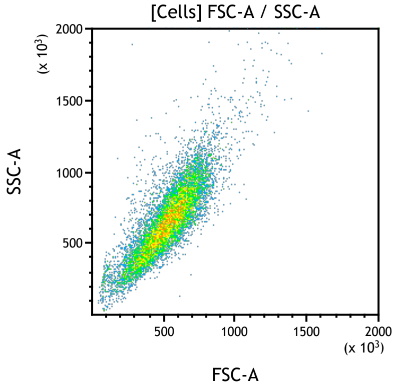

5_CD3531 5.4
[Cells] PE-A

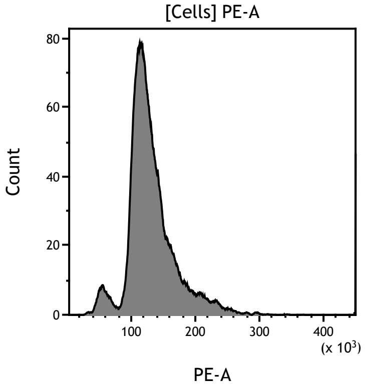

New Composite 6 - Plot Sheet 5

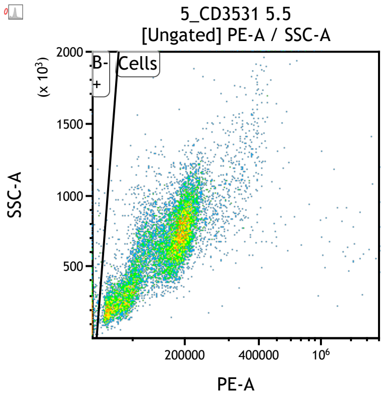

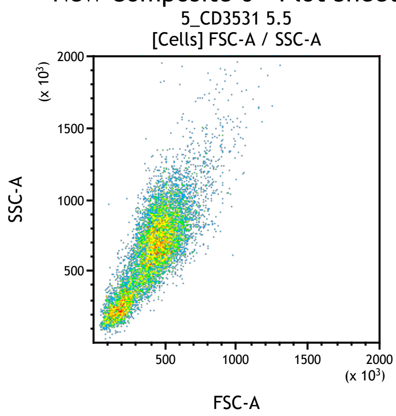

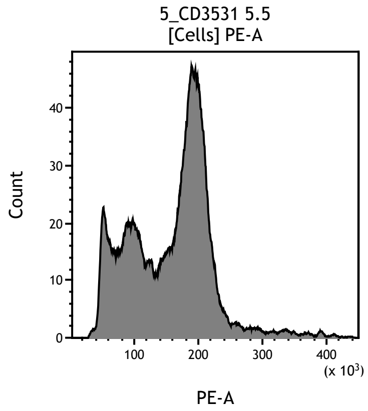

New Composite 6 - Plot Sheet 6

5_CD3531 5.6
[Ungated] PE-A / SSC-A

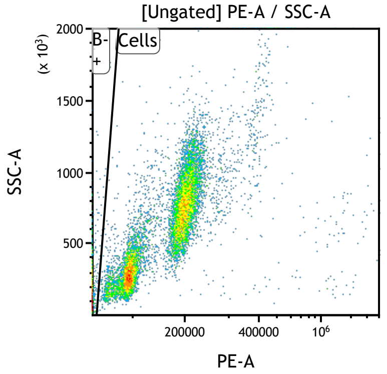

5_CD3531 5.6
[Cells] FSC-A / SSC-A

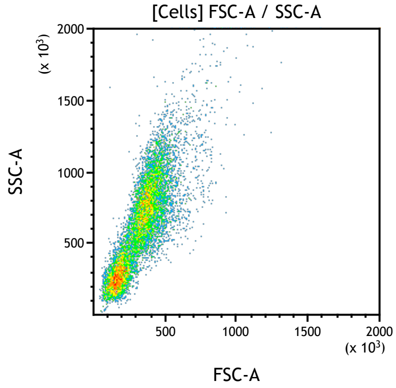

5_CD3531 5.6
[Cells] PE-A

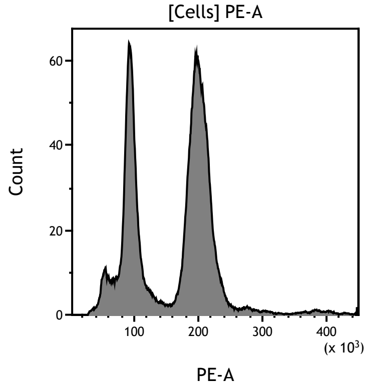

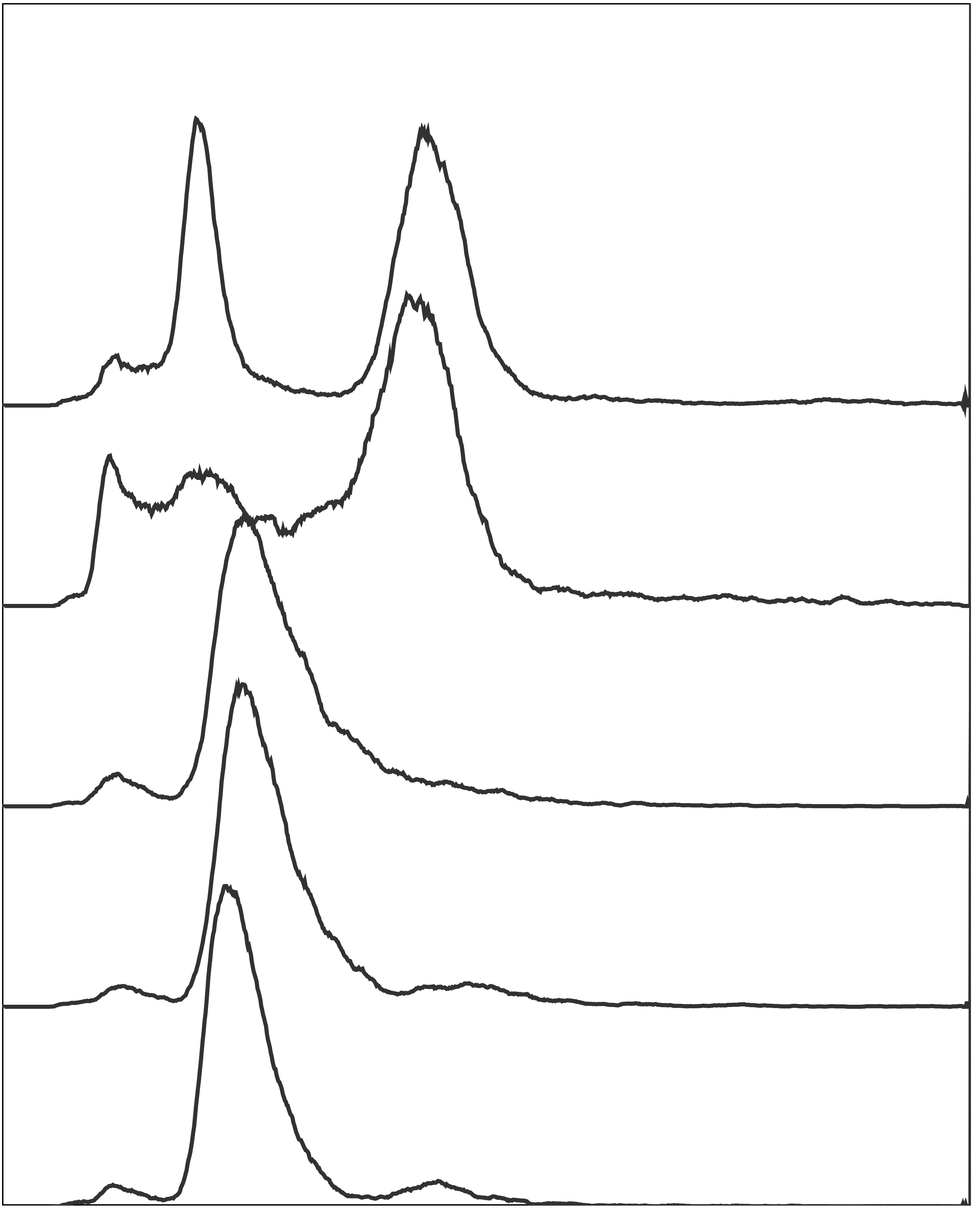

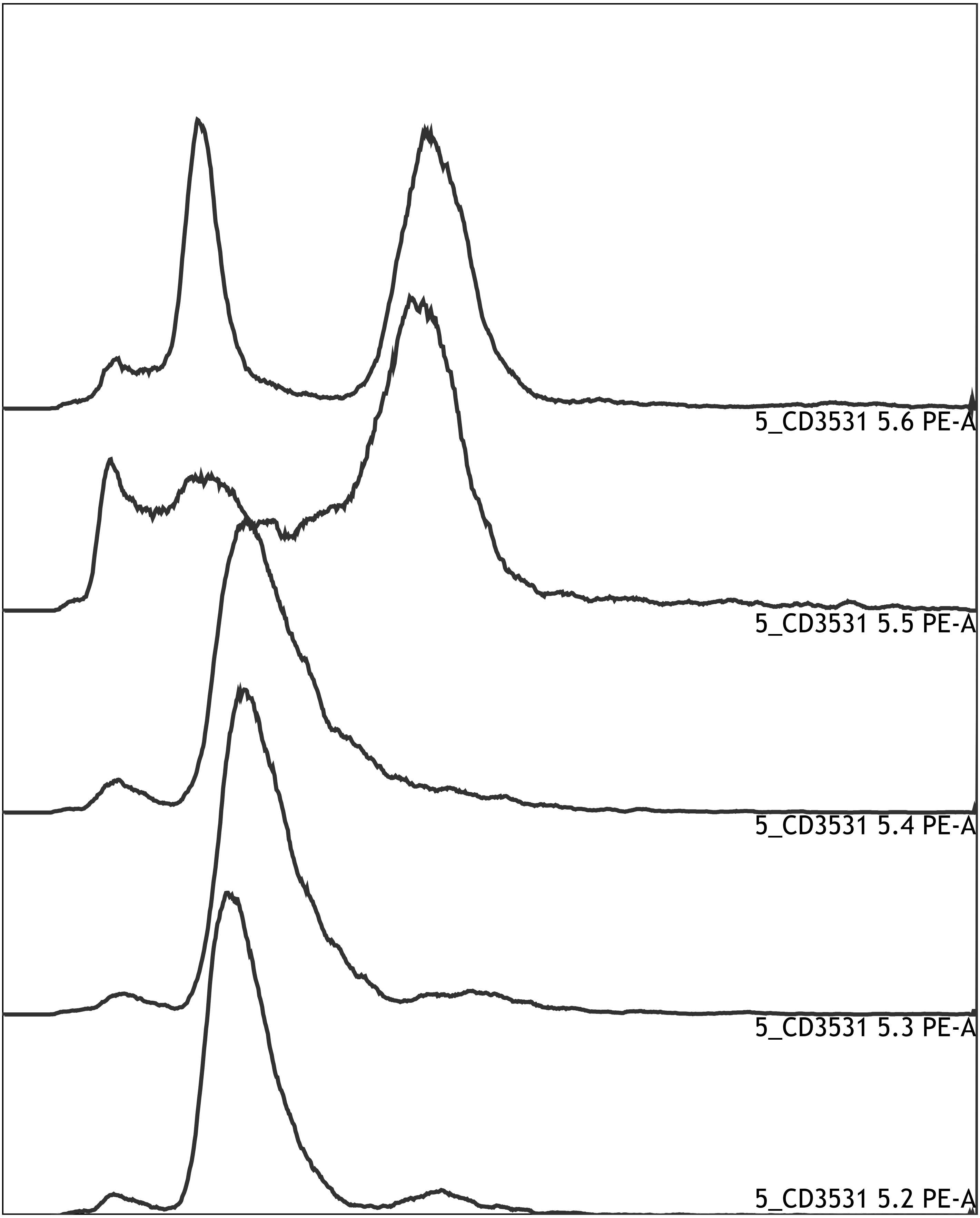
5_CD3531 5.6 PE-A
5_CD3531 5.5 PE-A
5_CD3531 5.4 PE-A
5_CD3531 5.3 PE-A
5_CD3531 5.2 PE-A

New Composite 7 - Plot Sheet 2

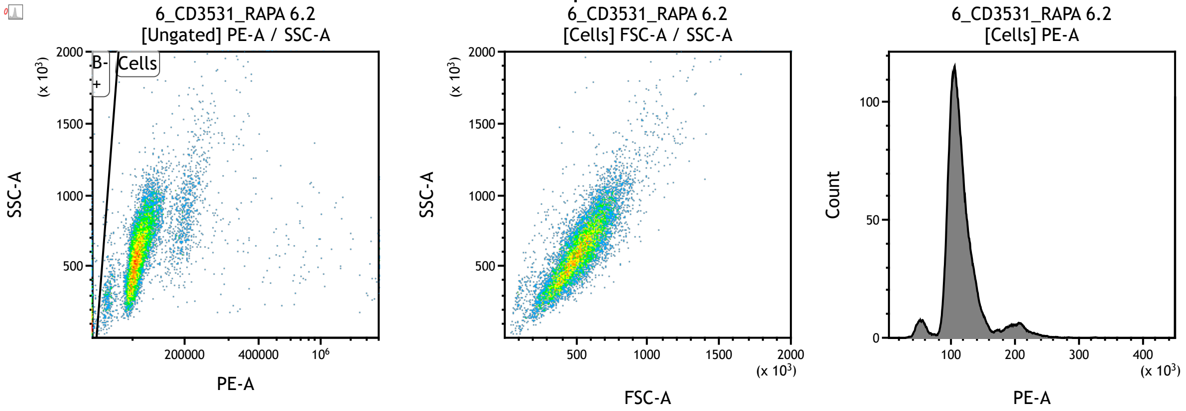

New Composite 7 - Plot Sheet 3

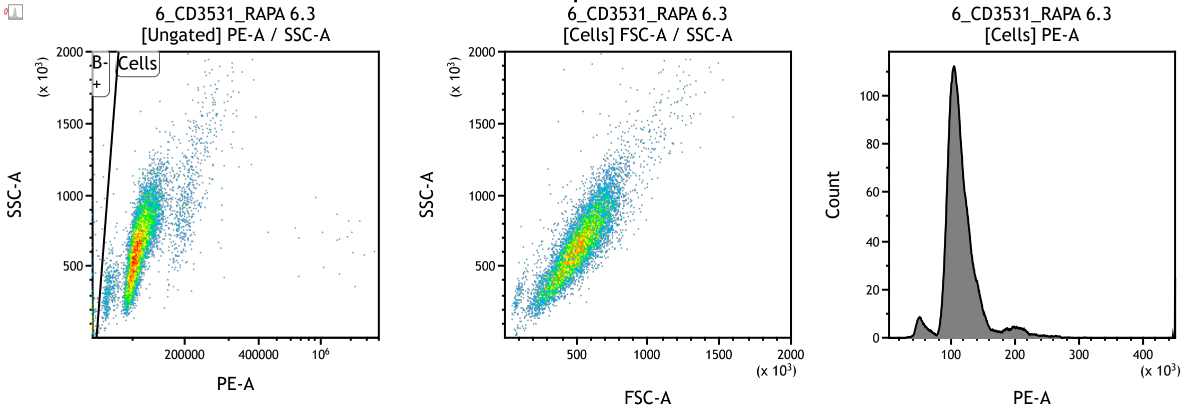

New Composite 7 - Plot Sheet 4

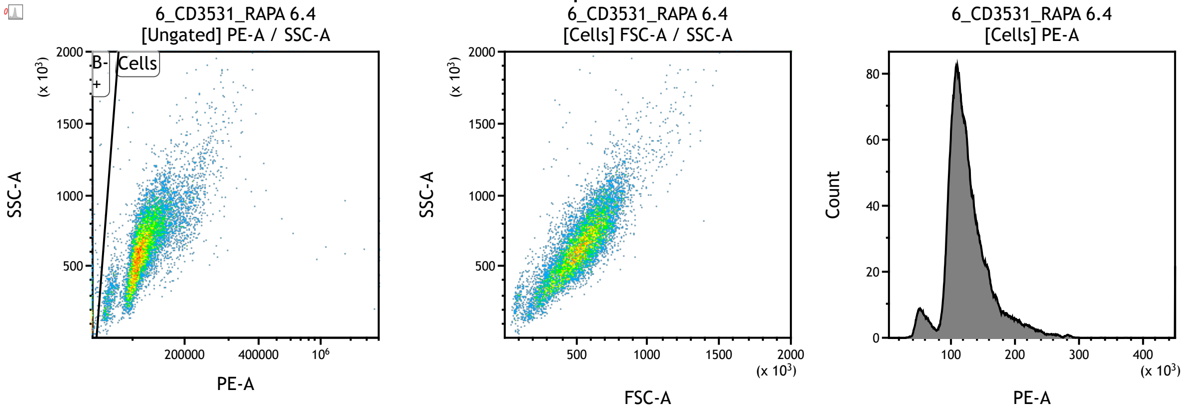

New Composite 7 - Plot Sheet 5

6_CD3531_RAPA 6.5
[Ungated] PE-A / SSC-A

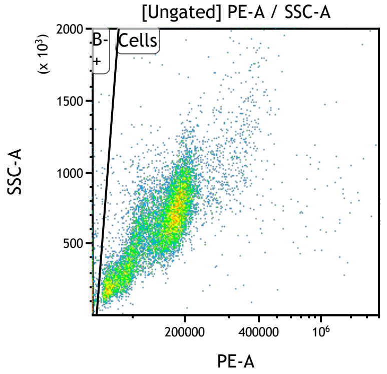

6_CD3531_RAPA 6.5
[Cells] FSC-A / SSC-A

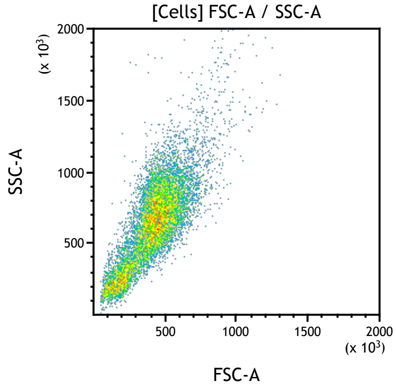

6_CD3531_RAPA 6.5
[Cells] PE-A

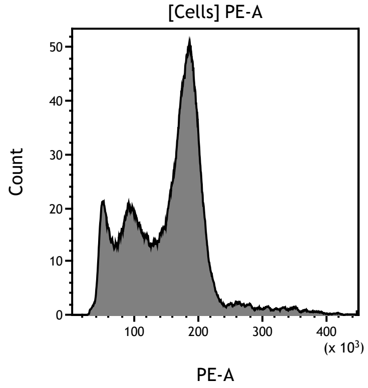

New Composite 7 - Plot Sheet 6

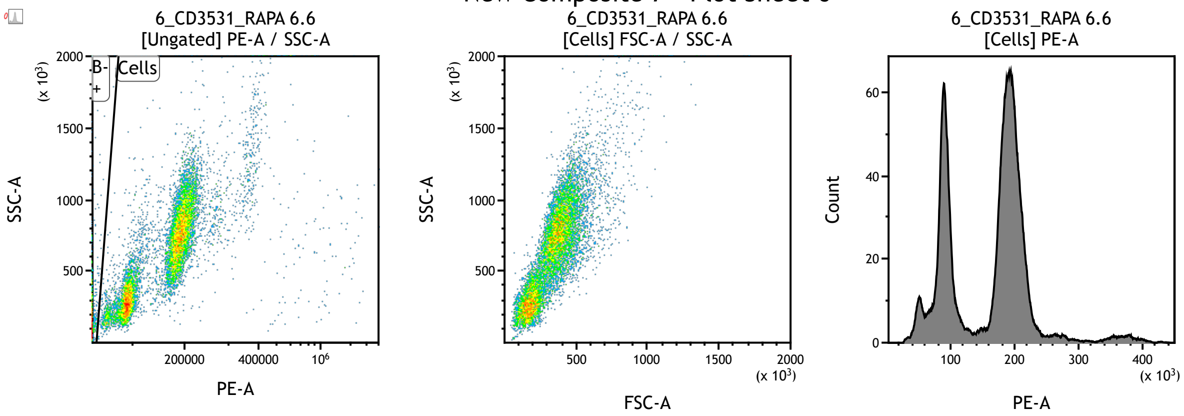

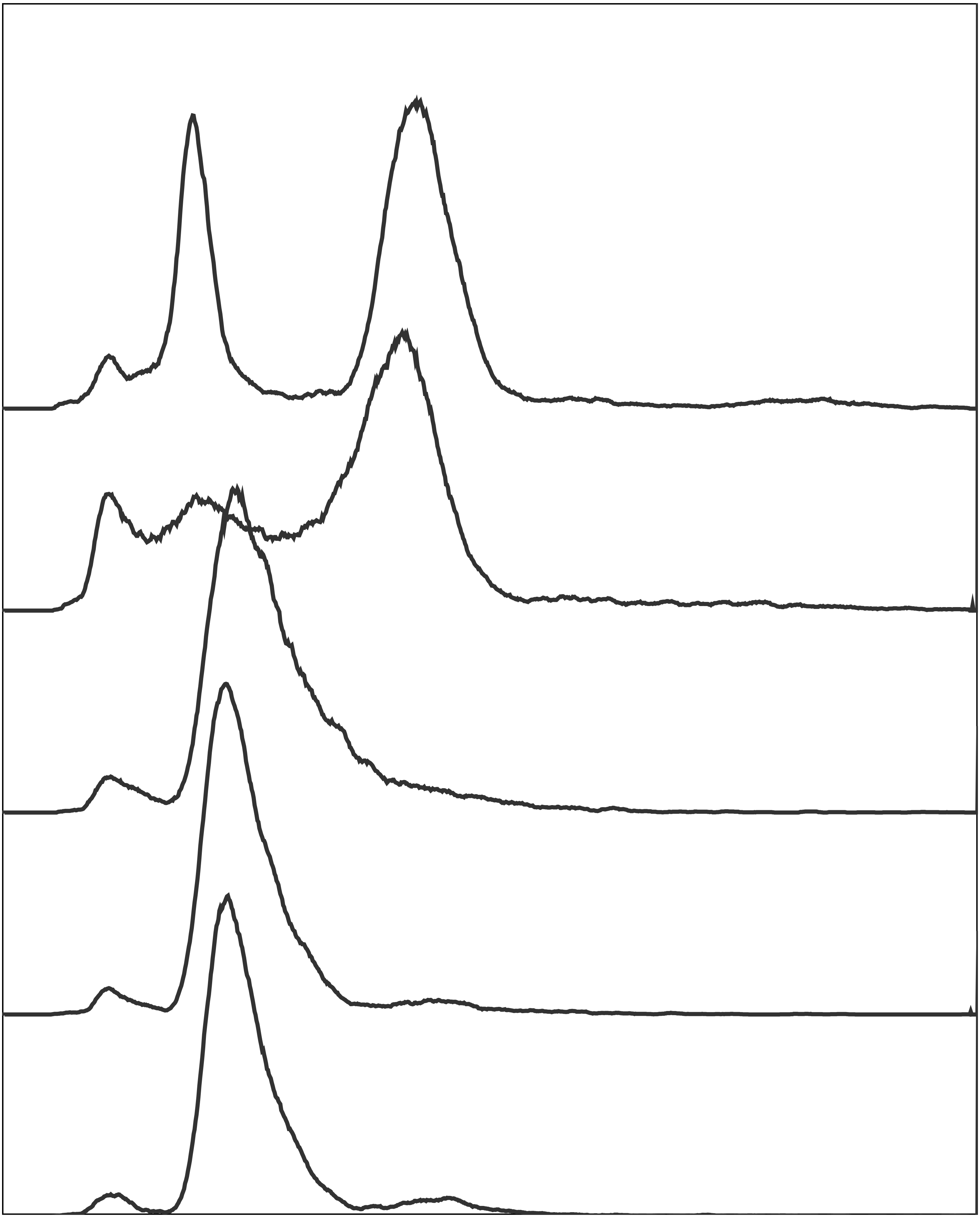

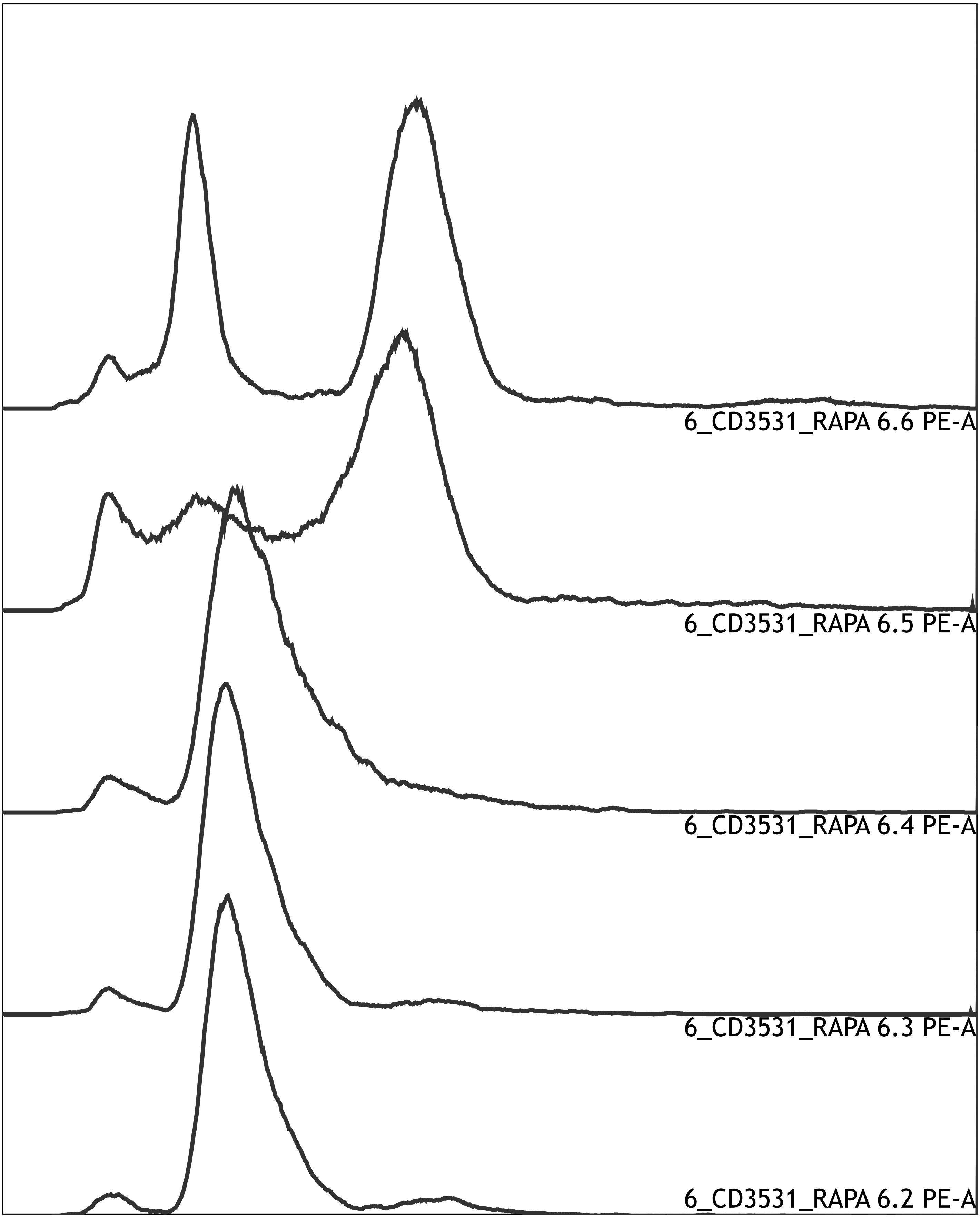
6_CD3531_RAPA 6.6 PE-A
6_CD3531_RAPA 6.5 PE-A
6_CD3531_RAPA 6.4 PE-A
6_CD3531_RAPA 6.3 PE-A
6_CD3531_RAPA 6.2 PE-A
