# Supplementary material for: TOR complex 1 negatively regulates NDR kinase Cbk1 to control cell separation in budding yeast
Source: PLoS Biol. 2023 Aug 30;21(8):e3002263. doi: 10.1371/journal.pbio.3002263 (PMC10468069; doi:10.1371/journal.pbio.3002263)
Supplement: S1 File — (ZIP) [file pbio.3002263.s024.zip › 2C.pdf]

New Composite 13 - Plot Sheet 2

11_CD4776_MINUS_MINUS 11.2
[Ungated] IP PE-A / SSC-A

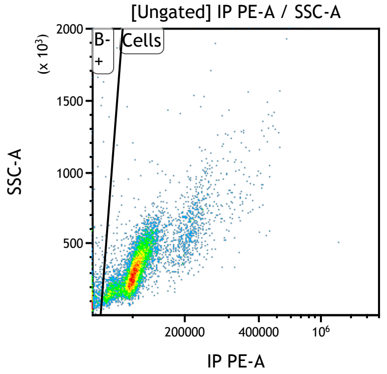

11_CD4776_MINUS_MINUS 11.2
[Cells] FSC-A / SSC-A

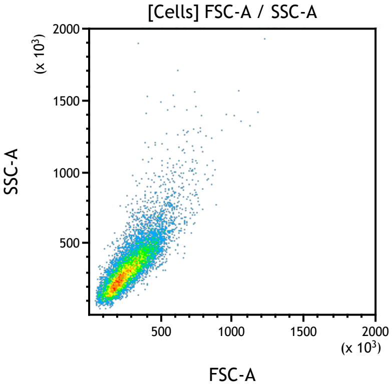

11_CD4776_MINUS_MINUS 11.2
[Cells] IP PE-A

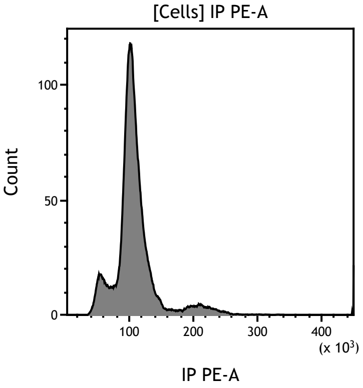

New Composite 13 - Plot Sheet 4

11_CD4776_MINUS_MINUS 11.4
[Ungated] IP PE-A / SSC-A

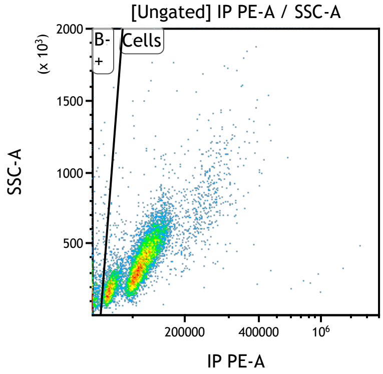

11_CD4776_MINUS_MINUS 11.4
[Cells] FSC-A / SSC-A

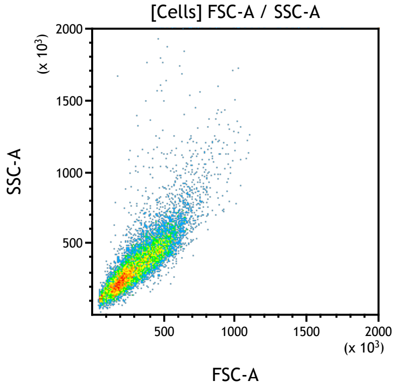

11_CD4776_MINUS_MINUS 11.4
[Cells] IP PE-A

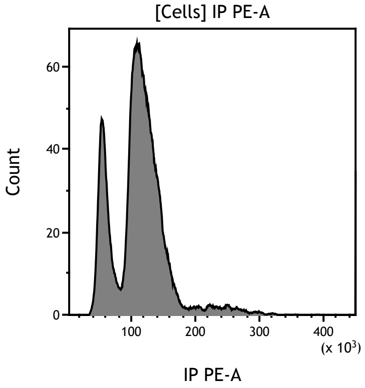

New Composite 13 - Plot Sheet 5

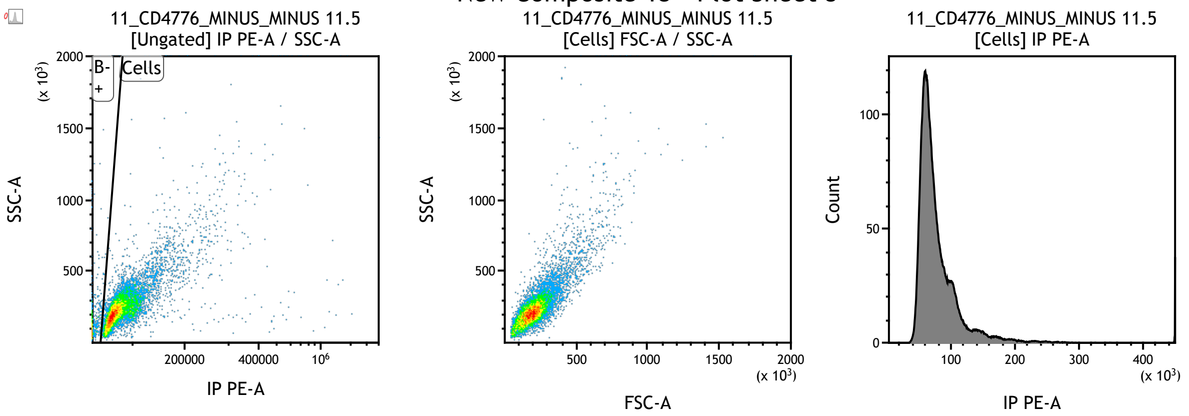

New Composite 13 - Plot Sheet 6

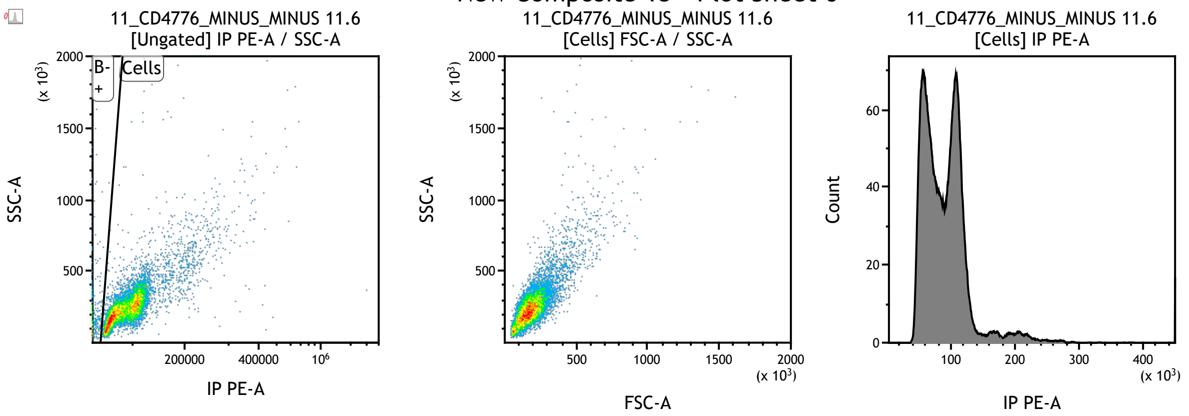

New Composite 13 - Plot Sheet 7

11_CD4776_MINUS_MINUS 11.7
[Ungated] IP PE-A / SSC-A

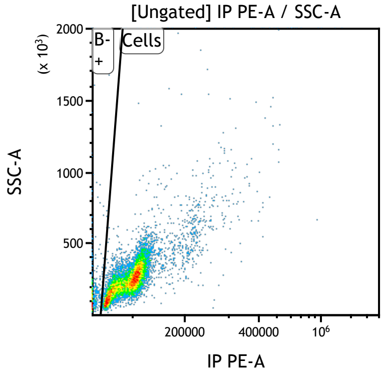

11_CD4776_MINUS_MINUS 11.7
[Cells] FSC-A / SSC-A

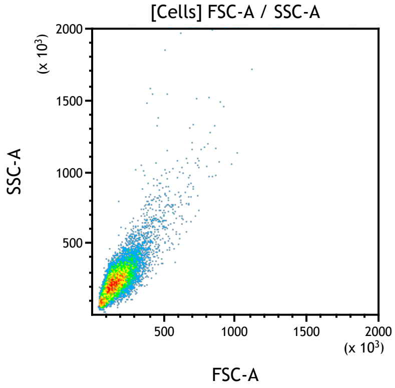

11_CD4776_MINUS_MINUS 11.7
[Cells] IP PE-A

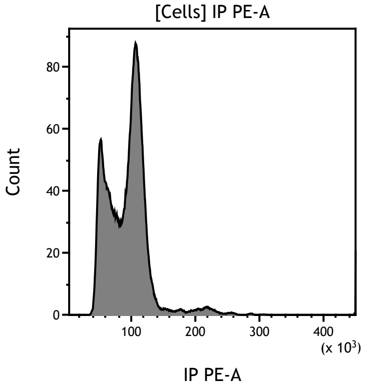

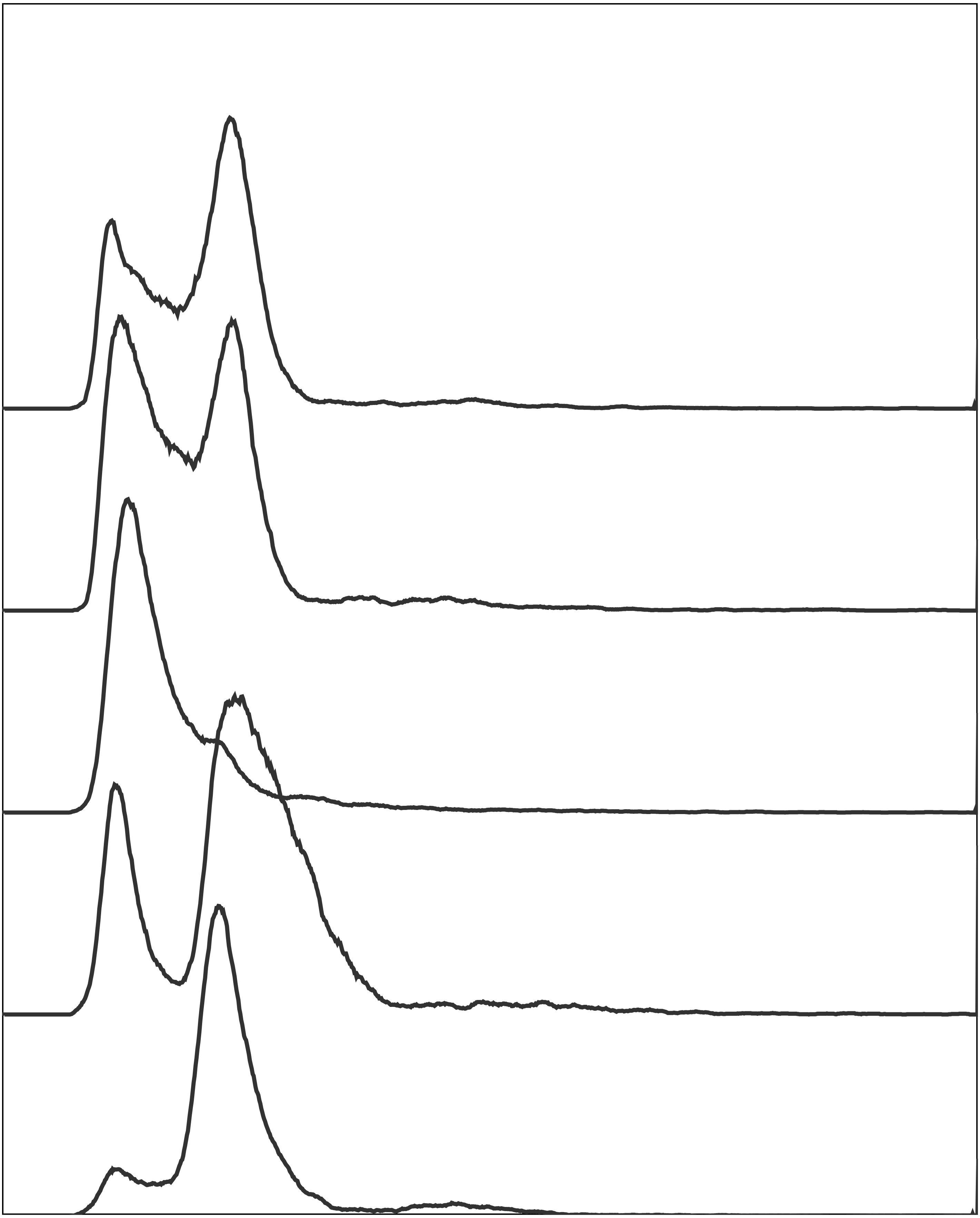

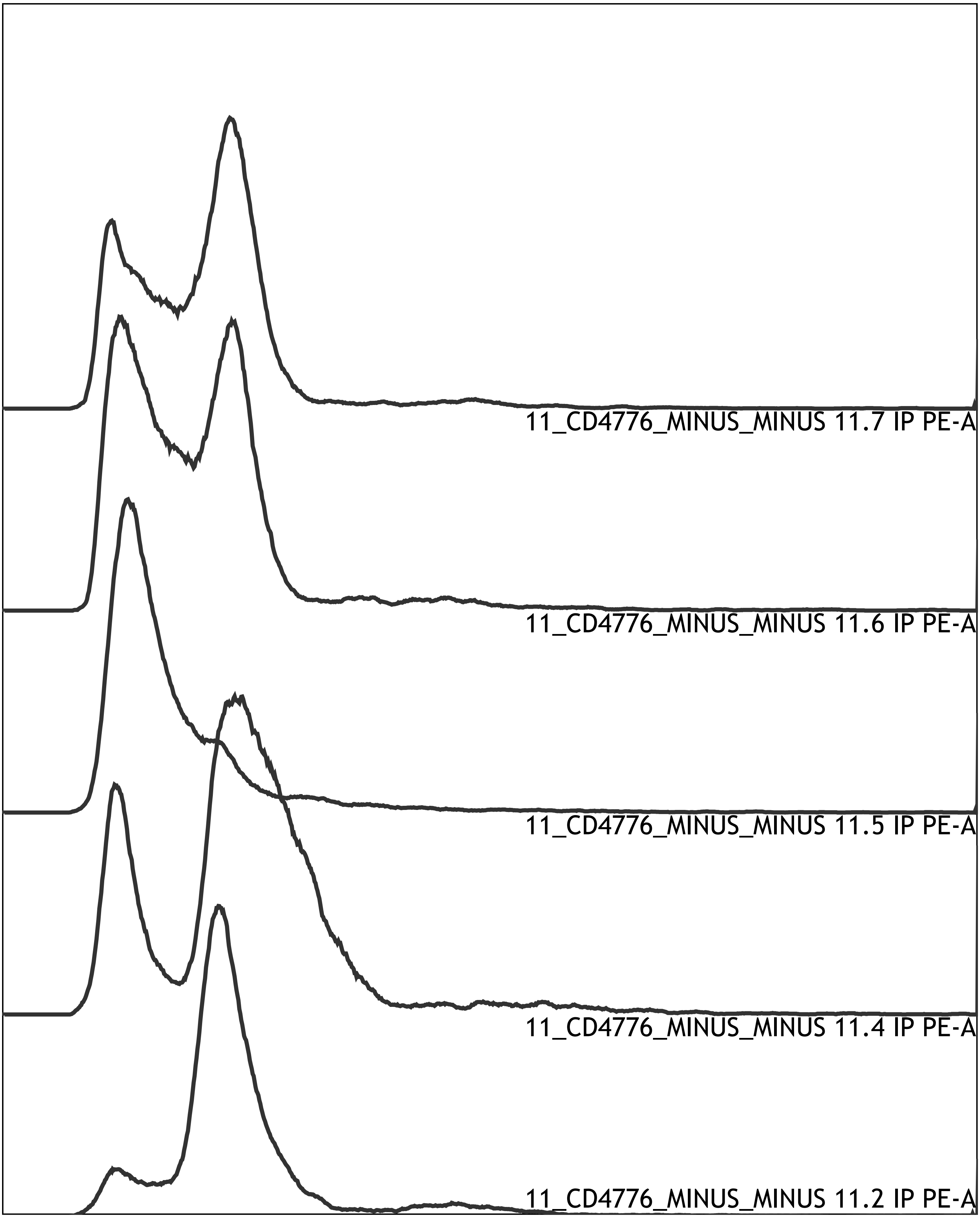
11_CD4776_MINUS_MINUS 11.7 IP PE-A
11_CD4776_MINUS_MINUS 11.6 IP PE-A
11_CD4776_MINUS_MINUS 11.5 IP PE-A
11_CD4776_MINUS_MINUS 11.4 IP PE-A
11_CD4776_MINUS_MINUS 11.2 IP PE-A

New Composite 15 - Plot Sheet 2

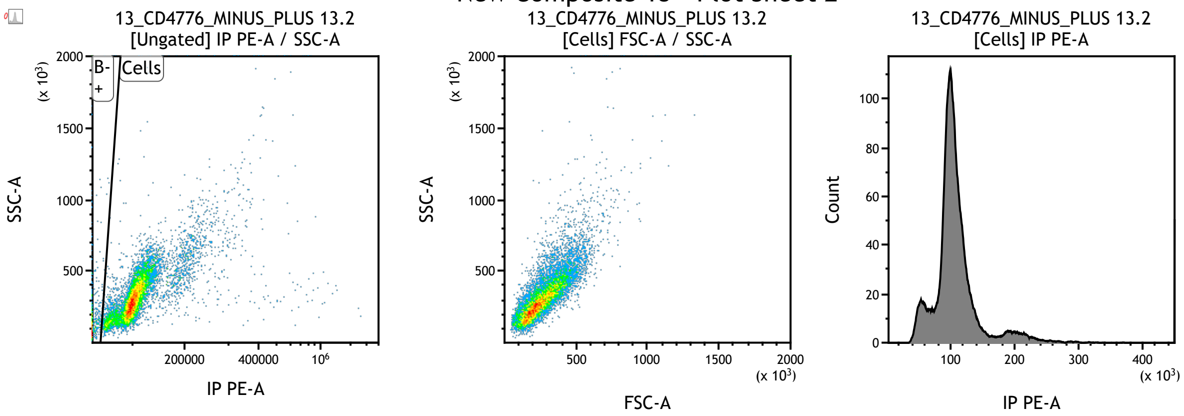

New Composite 15 - Plot Sheet 4

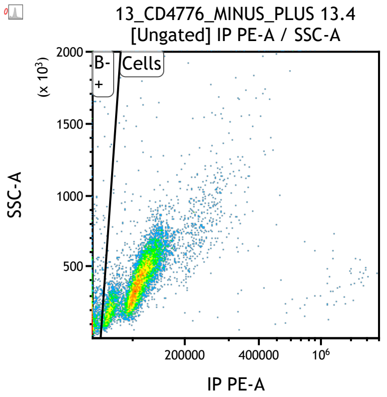

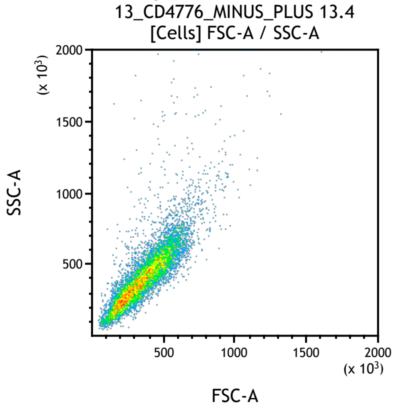

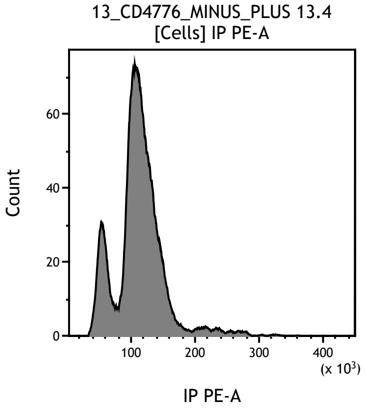

New Composite 15 - Plot Sheet 5

13_CD4776_MINUS_PLUS 13.5
[Ungated] IP PE-A / SSC-A

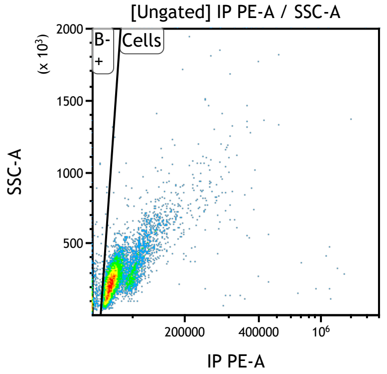

13_CD4776_MINUS_PLUS 13.5
[Cells] FSC-A / SSC-A

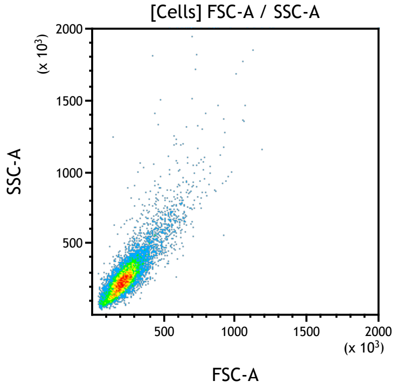

13_CD4776_MINUS_PLUS 13.5
[Cells] IP PE-A

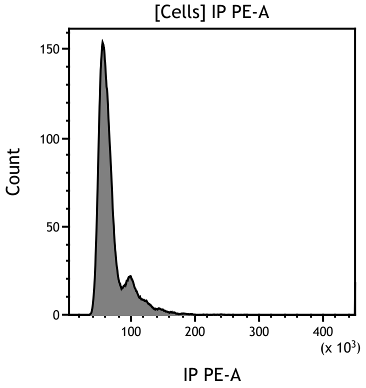

New Composite 15 - Plot Sheet 6

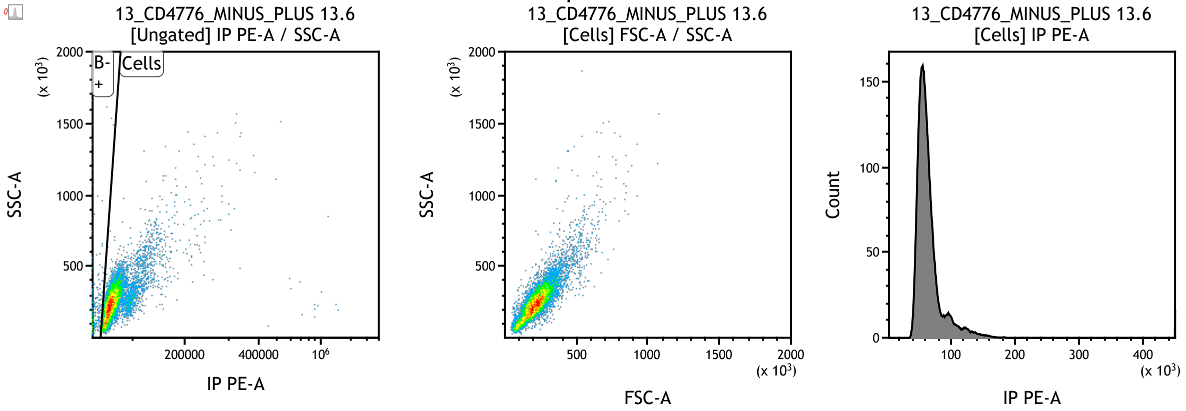

New Composite 15 - Plot Sheet 7

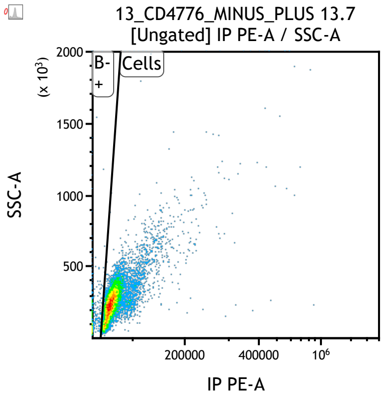

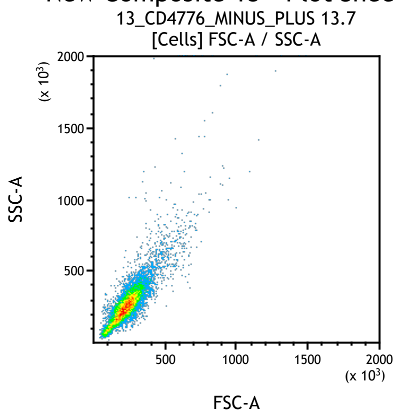

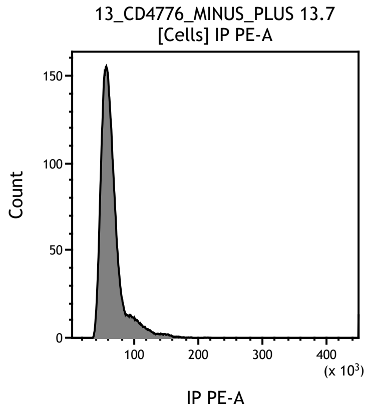

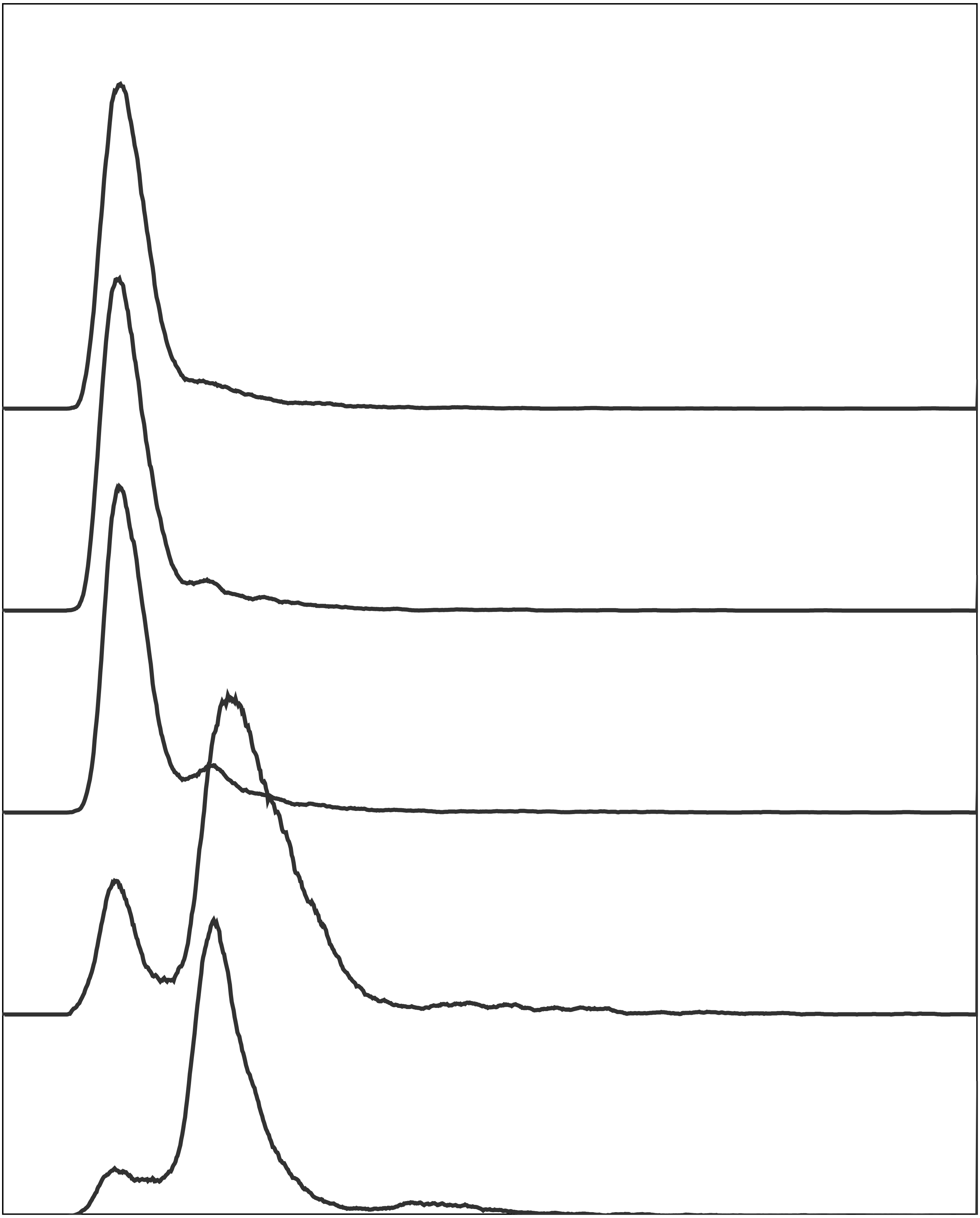

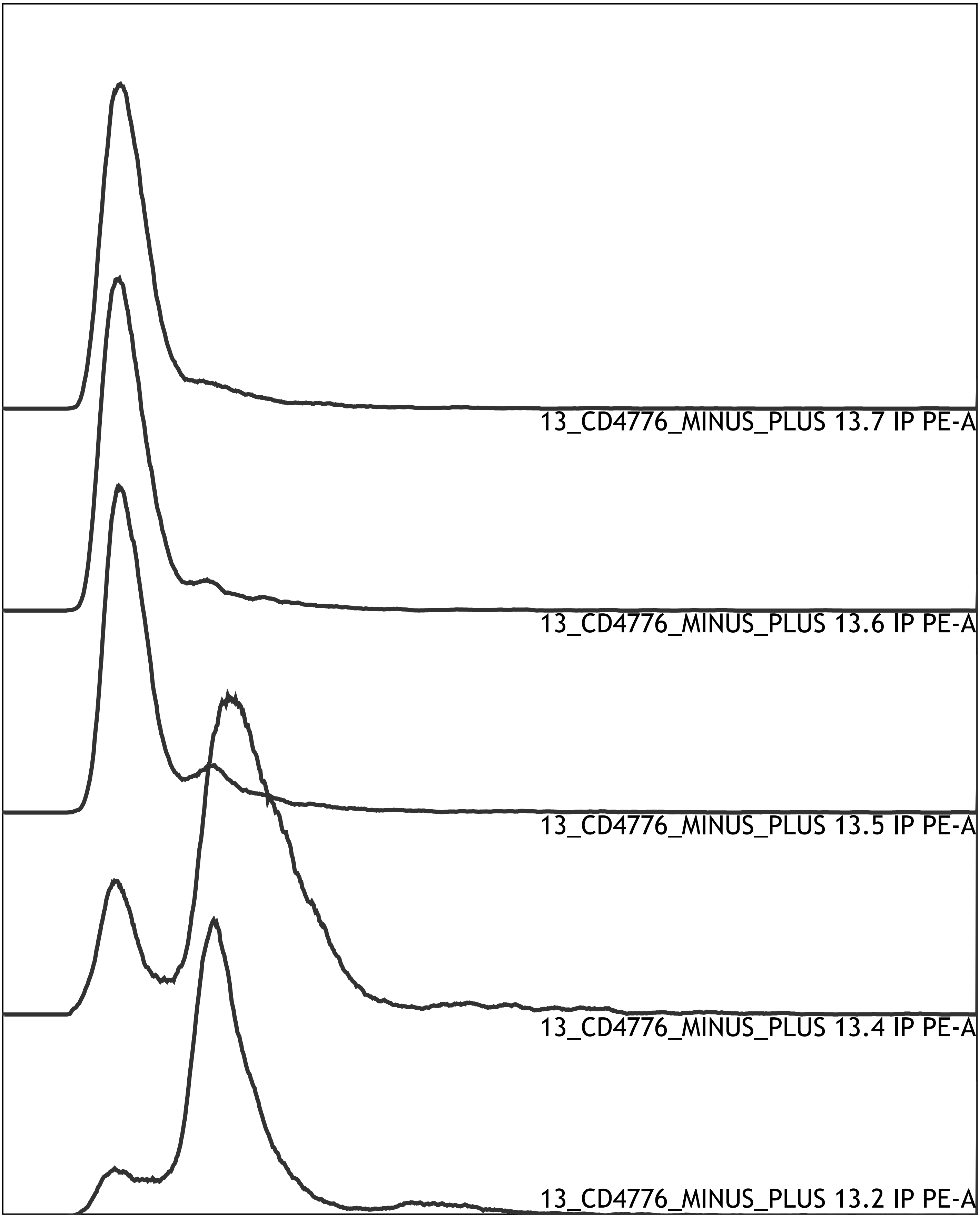
13_CD4776_MINUS_PLUS 13.7 IP PE-A
13_CD4776_MINUS_PLUS 13.6 IP PE-A
13_CD4776_MINUS_PLUS 13.5 IP PE-A
13_CD4776_MINUS_PLUS 13.4 IP PE-A
13_CD4776_MINUS_PLUS 13.2 IP PE-A
