# Supplementary material for: TOR complex 1 negatively regulates NDR kinase Cbk1 to control cell separation in budding yeast
Source: PLoS Biol. 2023 Aug 30;21(8):e3002263. doi: 10.1371/journal.pbio.3002263 (PMC10468069; doi:10.1371/journal.pbio.3002263)
Supplement: S1 File — (ZIP) [file pbio.3002263.s024.zip › 2E.pdf]

New Composite 10 - Plot Sheet 2

CD492_3.2
[Ungated] IP PE-A / SSC-A

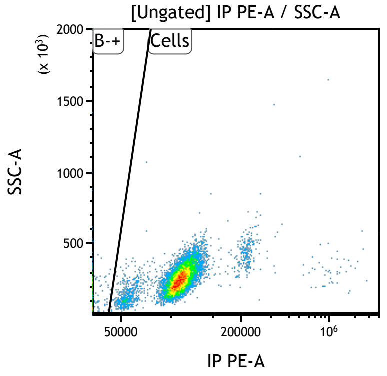

CD492_3.2
[Cells] FSC-A / SSC-A

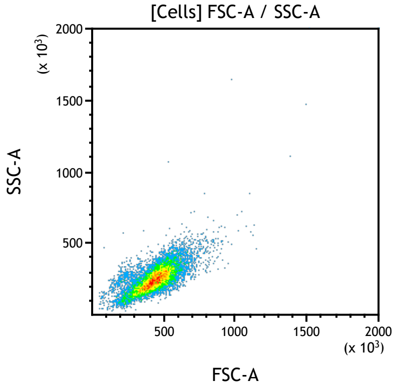

CD492_3.2
[Cells] IP PE-A

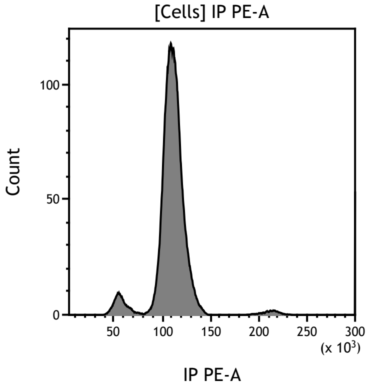

New Composite 10 - Plot Sheet 3

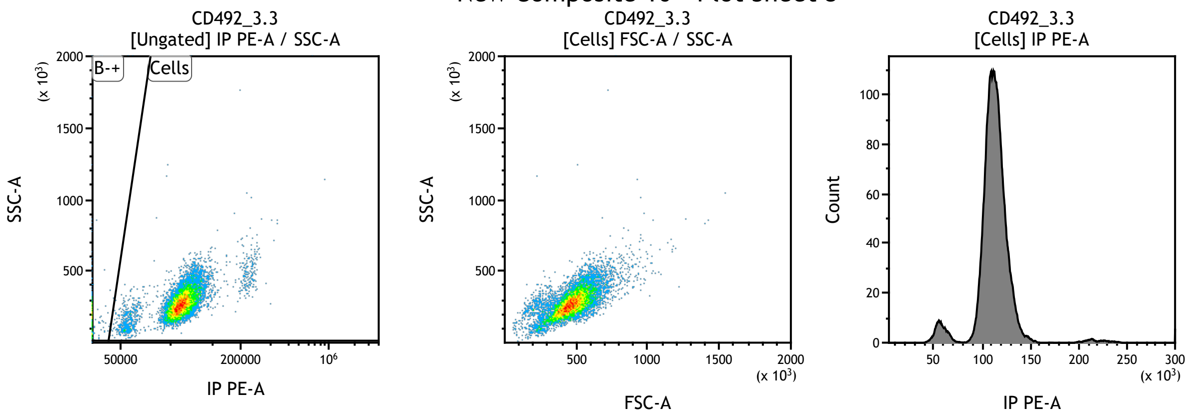

New Composite 10 - Plot Sheet 4

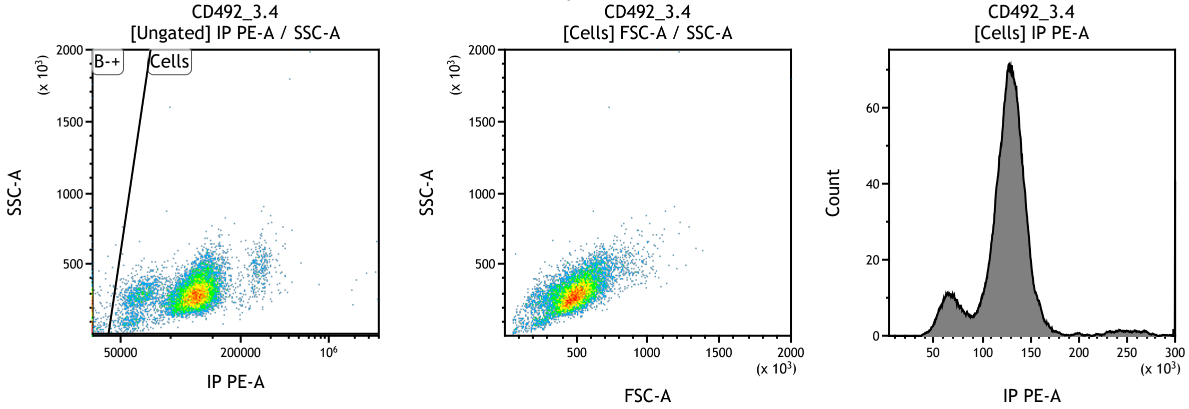

New Composite 10 - Plot Sheet 5

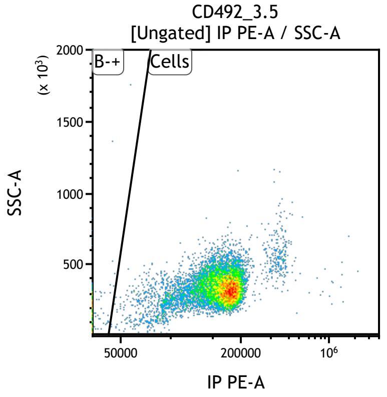

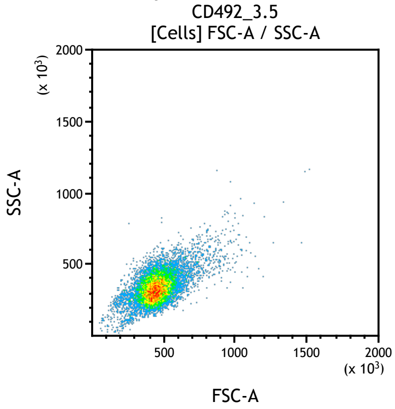

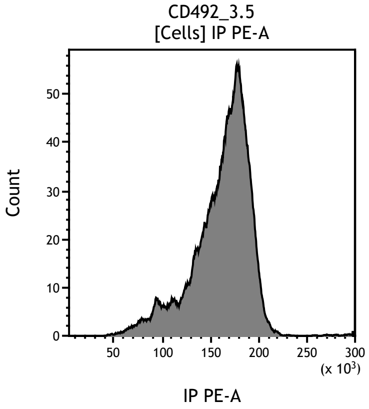

New Composite 10 - Plot Sheet 6

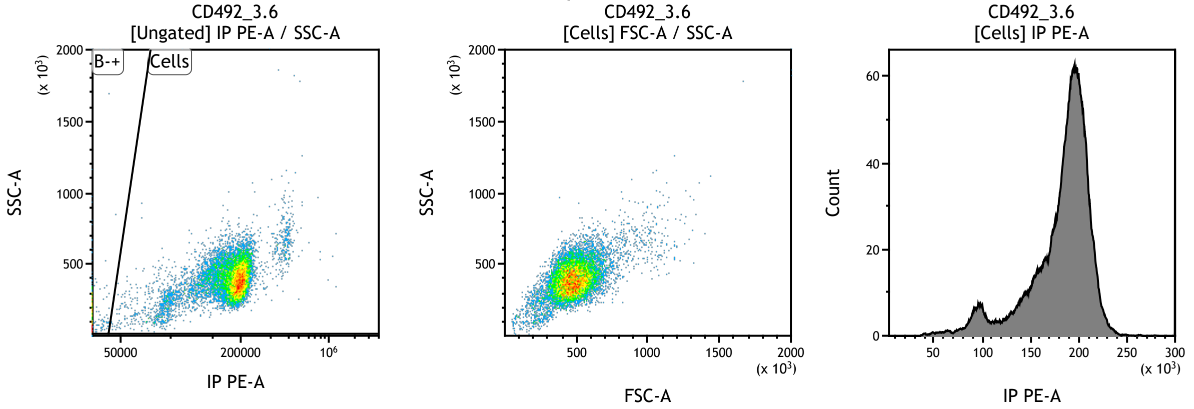

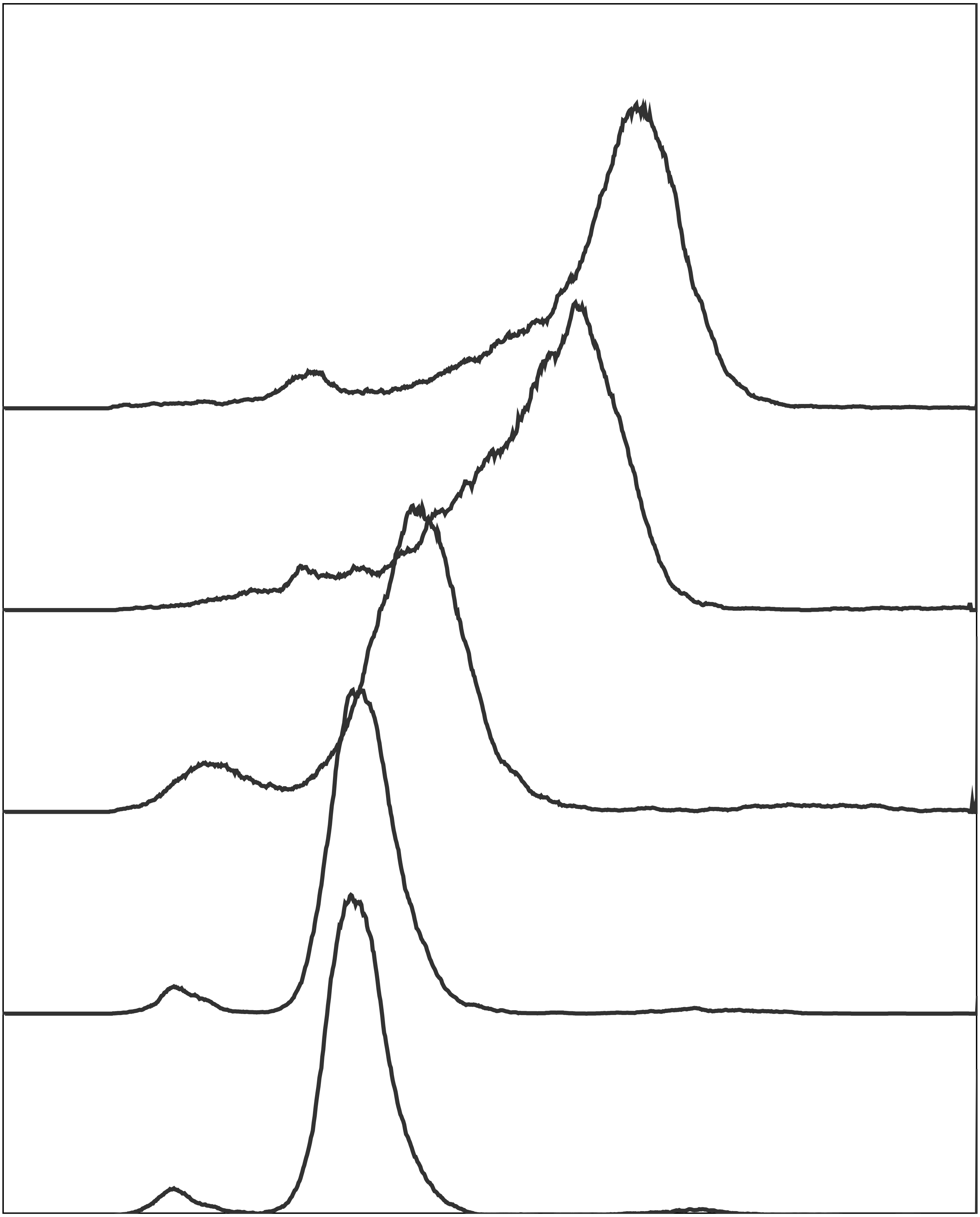

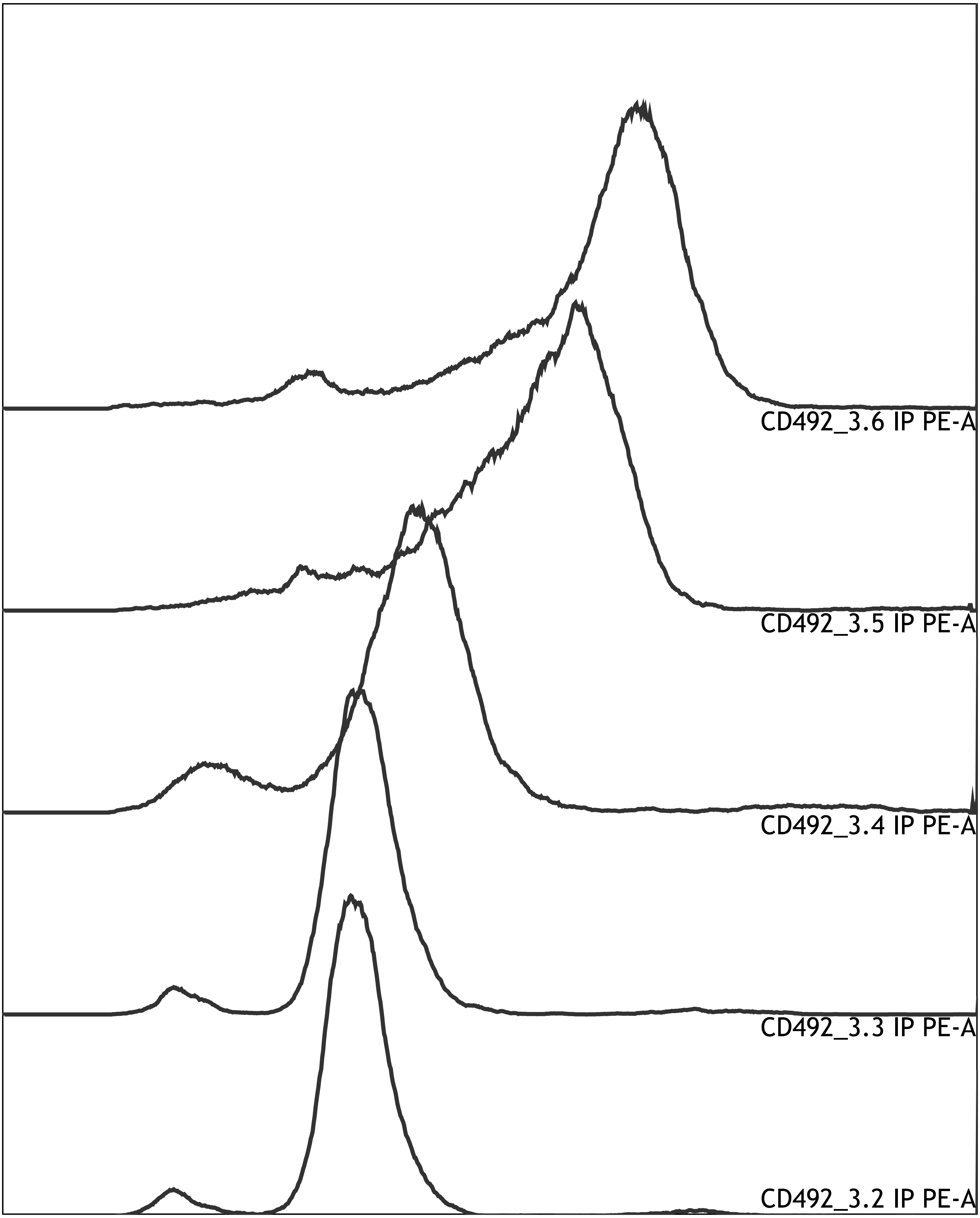
CD492_3.6 IP PE-A
CD492_3.5 IP PE-A
CD492_3.4 IP PE-A
CD492_3.3 IP PE-A
CD492_3.2 IP PE-A

New Composite 9 - Plot Sheet 2

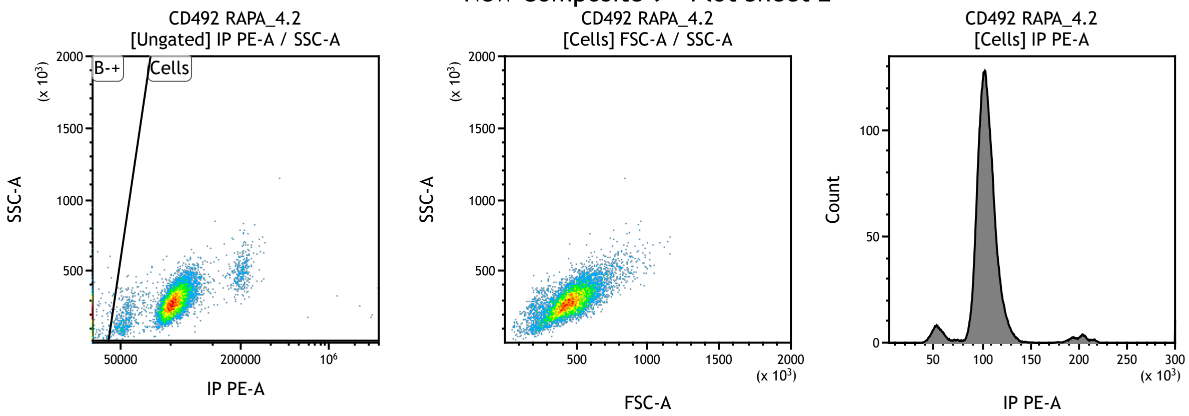

New Composite 9 - Plot Sheet 3

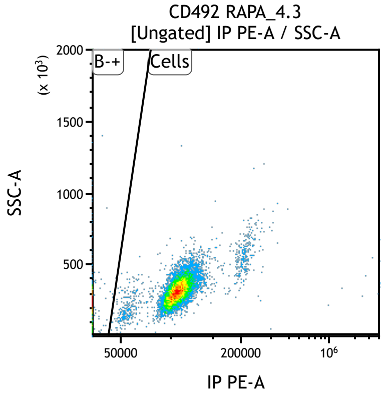

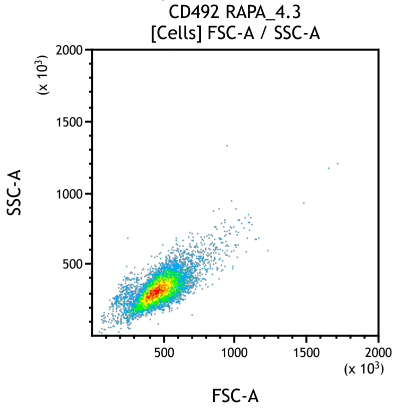

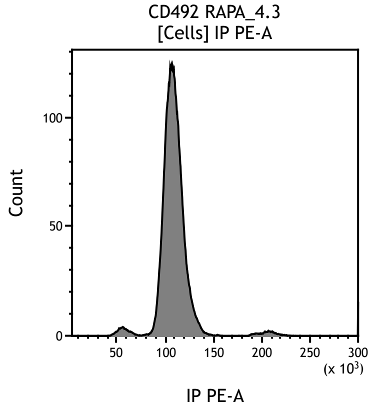

New Composite 9 - Plot Sheet 4

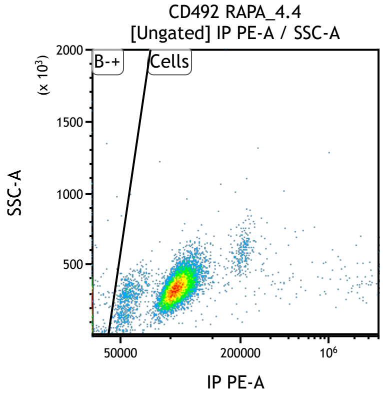

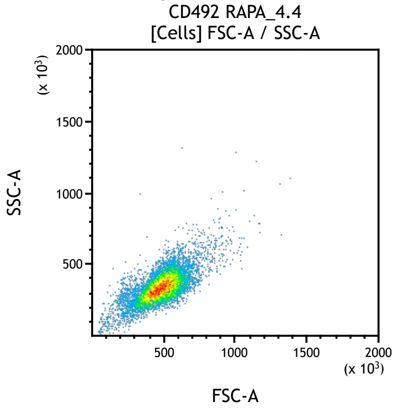

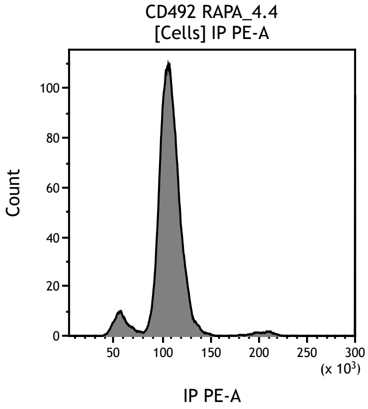

New Composite 9 - Plot Sheet 5

CD492 RAPA_4.5
[Ungated] IP PE-A / SSC-A

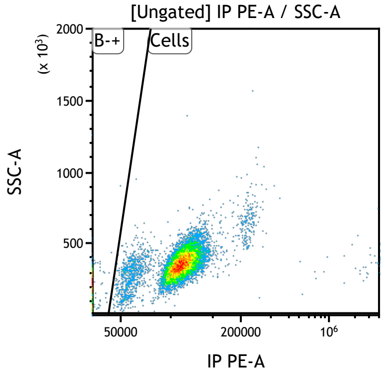

CD492 RAPA_4.5
[Cells] FSC-A / SSC-A

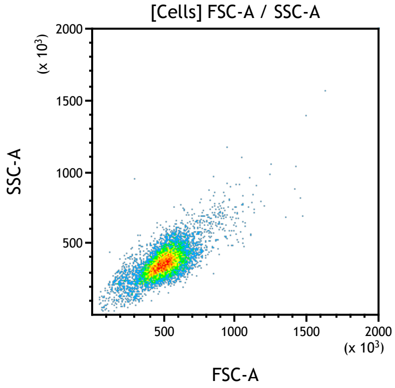

CD492 RAPA_4.5
[Cells] IP PE-A

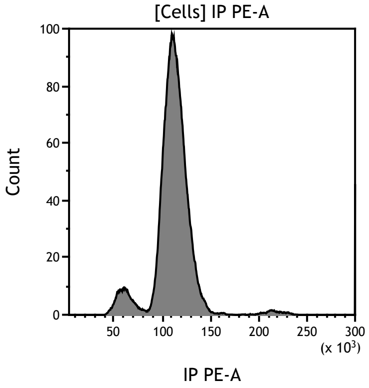

New Composite 9 - Plot Sheet 6

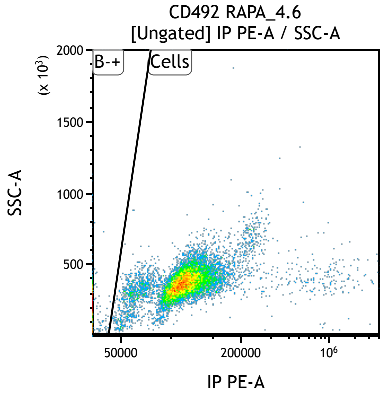

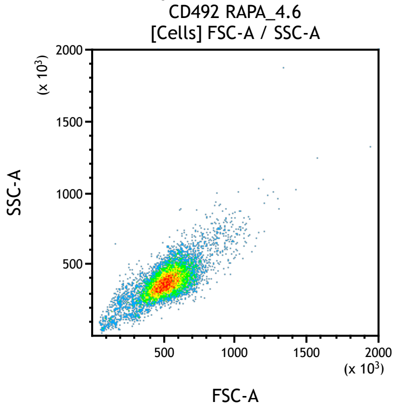

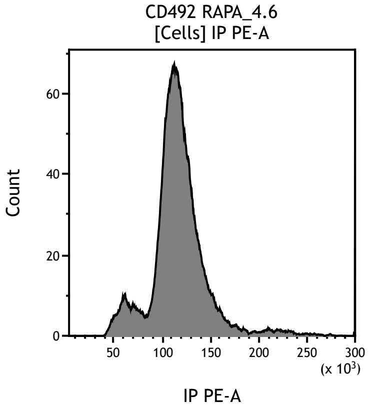

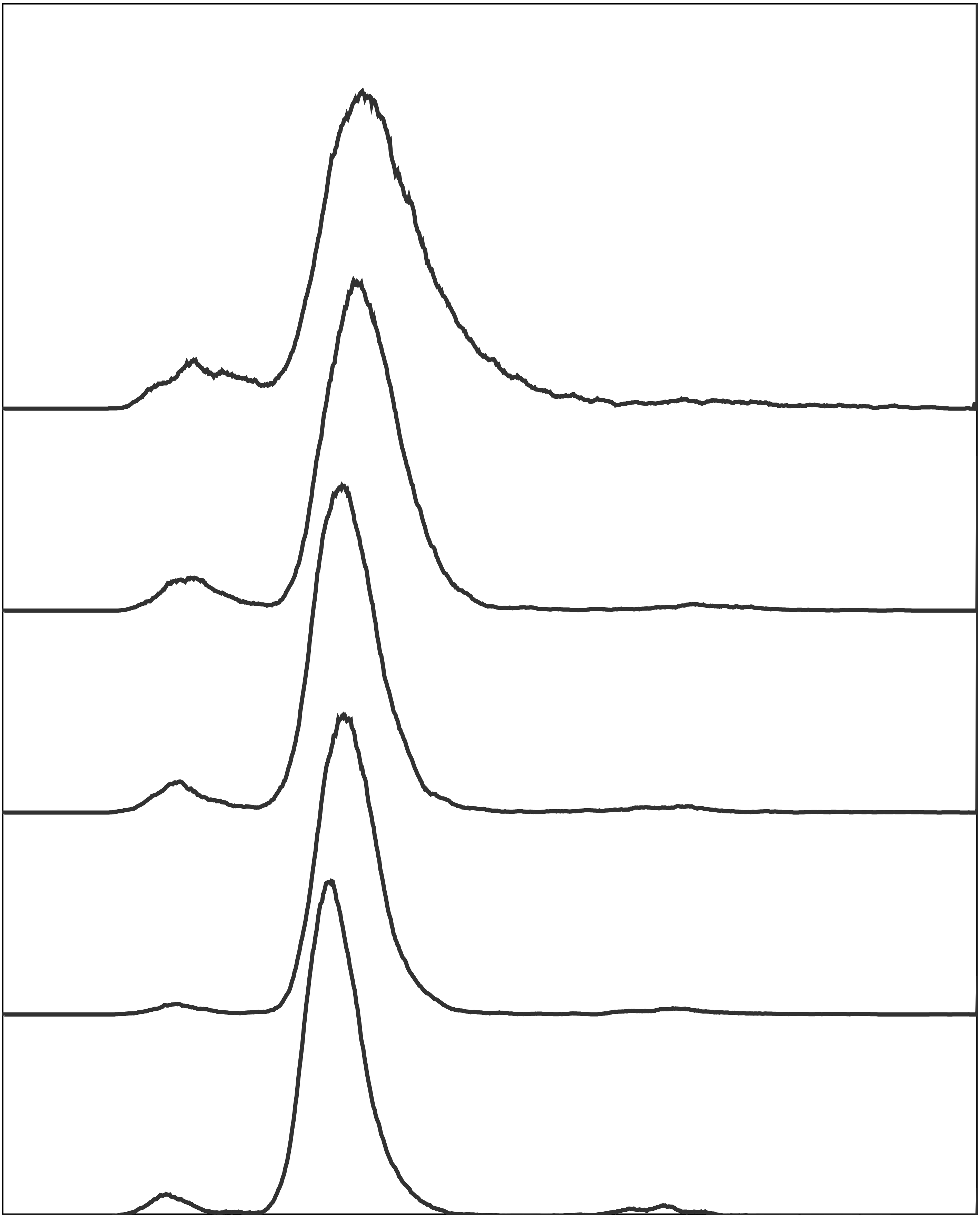

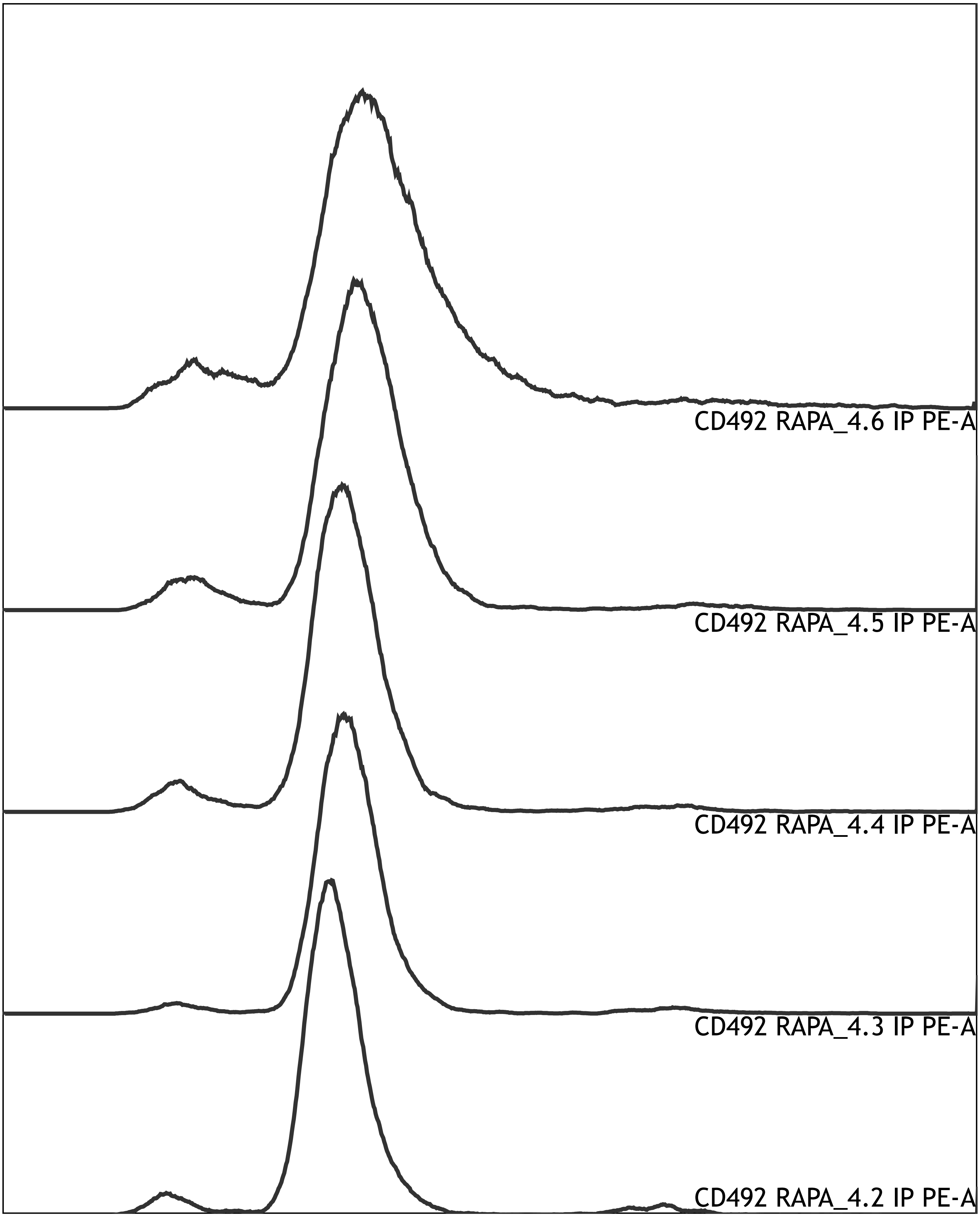
CD492 RAPA_4.6 IP PE-A
CD492 RAPA_4.5 IP PE-A
CD492 RAPA_4.4 IP PE-A
CD492 RAPA_4.3 IP PE-A
CD492 RAPA_4.2 IP PE-A
