# Supplementary material for: TOR complex 1 negatively regulates NDR kinase Cbk1 to control cell separation in budding yeast
Source: PLoS Biol. 2023 Aug 30;21(8):e3002263. doi: 10.1371/journal.pbio.3002263 (PMC10468069; doi:10.1371/journal.pbio.3002263)
Supplement: S1 File — (ZIP) [file pbio.3002263.s024.zip › 4A.pdf]

# New Composite 6 - Plot Sheet 3

15_CD4448 15.3
[Ungated] IP PE-A / SSC-A

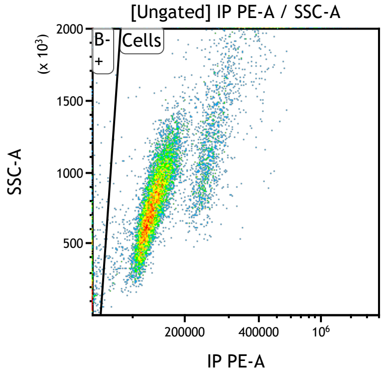


15_CD4448 15.3
[Cells] FSC-A / SSC-A

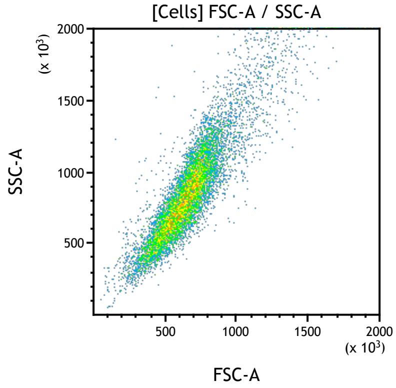


15_CD4448 15.3
[Cells] IP PE-A

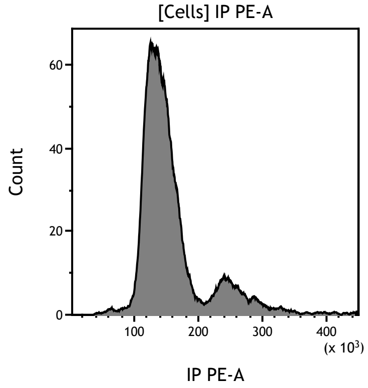

# New Composite 6 - Plot Sheet 4

15_CD4448 15.4
[Ungated] IP PE-A / SSC-A

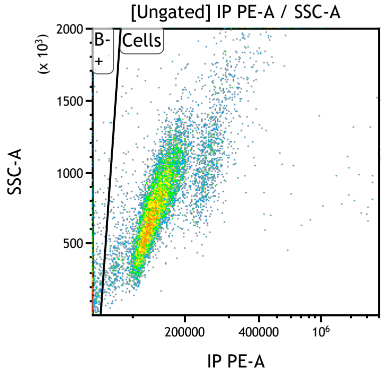


15_CD4448 15.4
[Cells] FSC-A / SSC-A

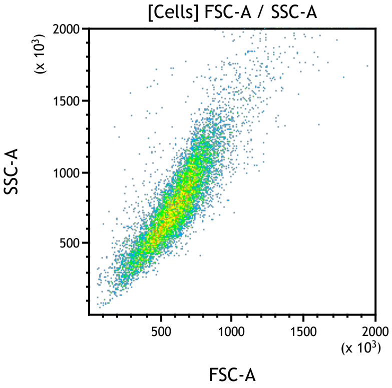


15_CD4448 15.4
[Cells] IP PE-A

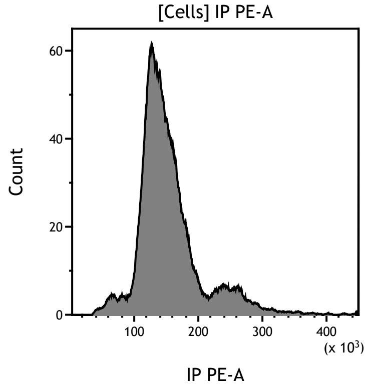

# New Composite 6 - Plot Sheet 5

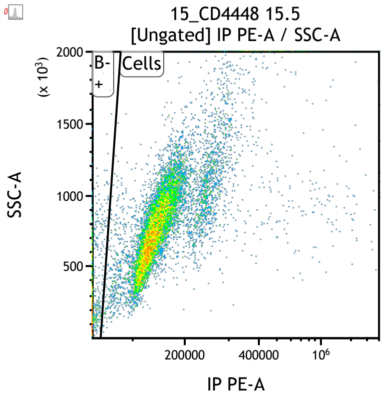


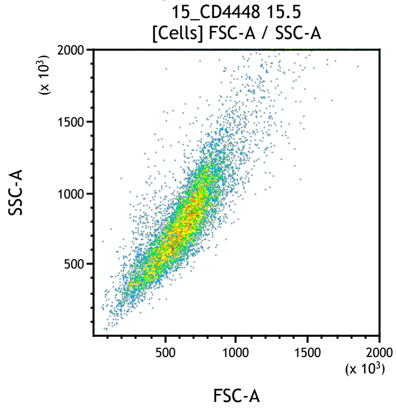


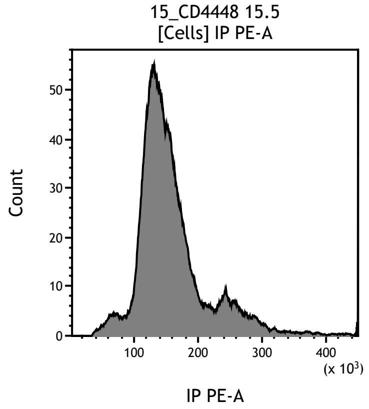

# New Composite 6 - Plot Sheet 6

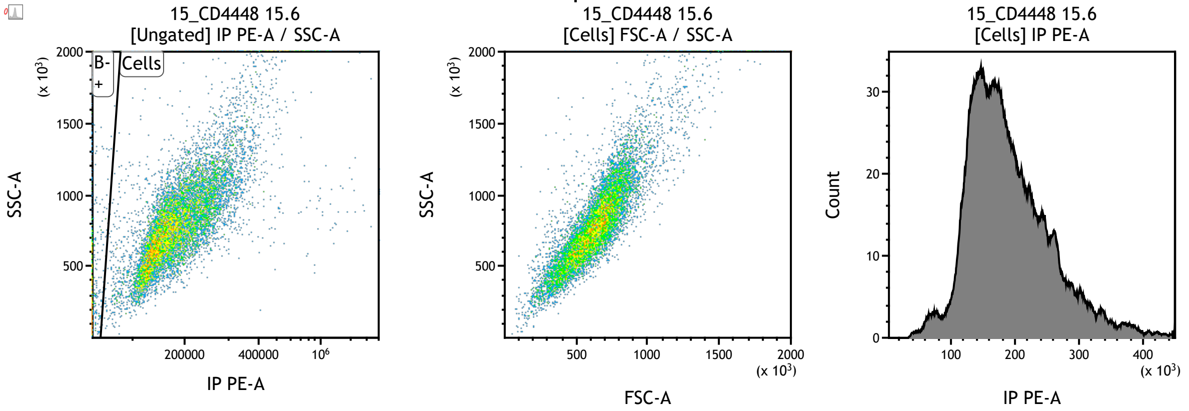

# New Composite 6 - Plot Sheet 7

15_CD4448 15.7
[Ungated] IP PE-A / SSC-A

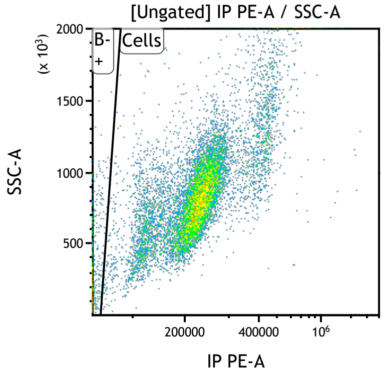


15_CD4448 15.7
[Cells] FSC-A / SSC-A

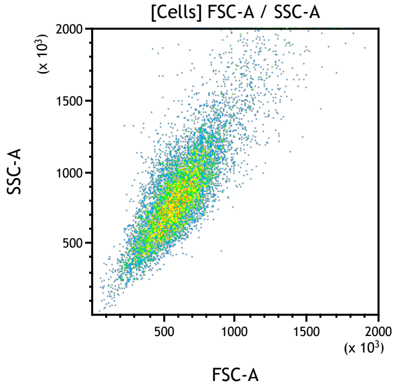


15_CD4448 15.7
[Cells] IP PE-A

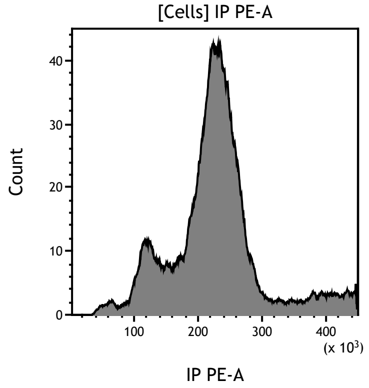

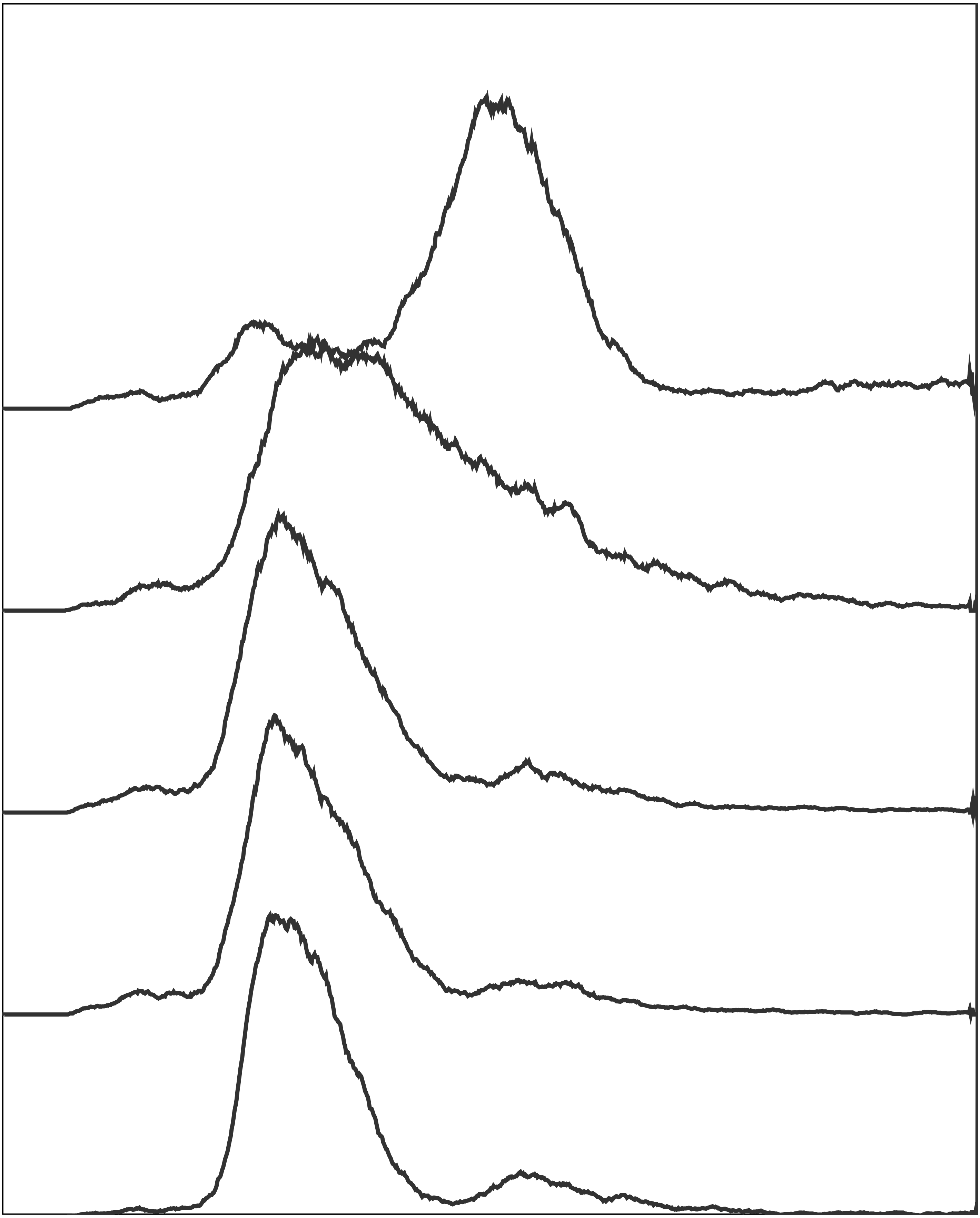

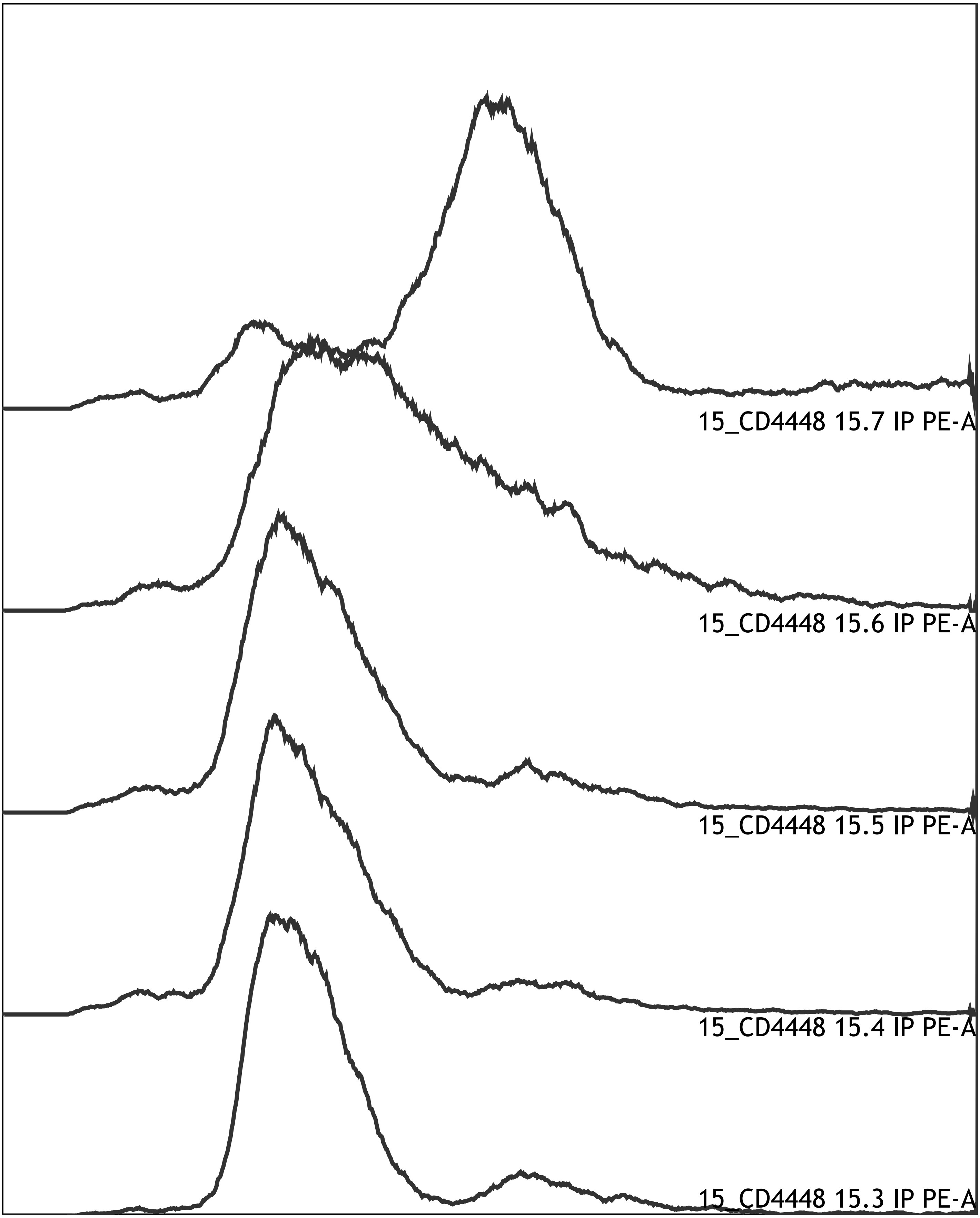
15_CD4448 15.7 IP PE-A
15_CD4448 15.6 IP PE-A
15_CD4448 15.5 IP PE-A
15_CD4448 15.4 IP PE-A
15_CD4448 15.3 IP PE-A

# New Composite 7 - Plot Sheet 3

16_CD4448_RAPA 16.3
[Ungated] IP PE-A / SSC-A

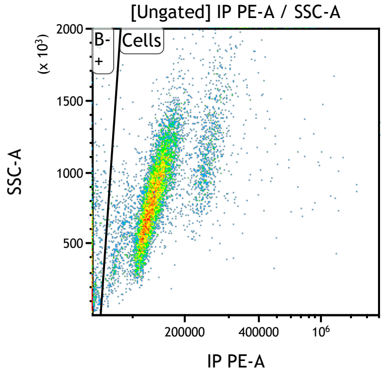


16_CD4448_RAPA 16.3
[Cells] FSC-A / SSC-A

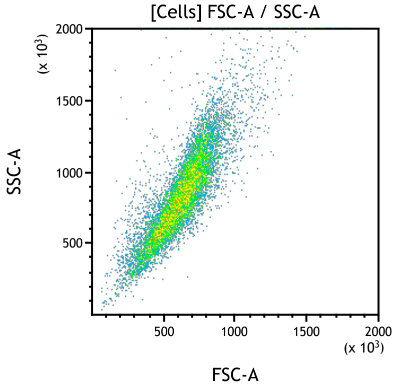


16_CD4448_RAPA 16.3
[Cells] IP PE-A

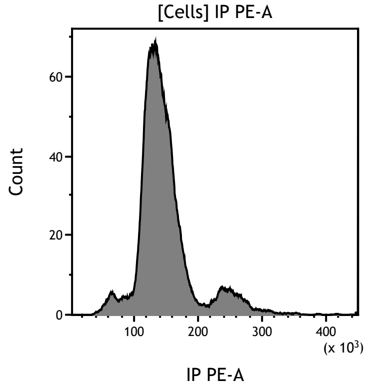

# New Composite 7 - Plot Sheet 4

16_CD4448_RAPA 16.4
[Ungated] IP PE-A / SSC-A

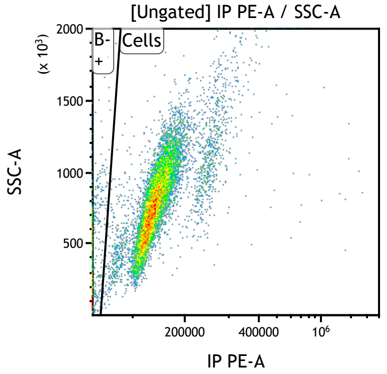


16_CD4448_RAPA 16.4
[Cells] FSC-A / SSC-A

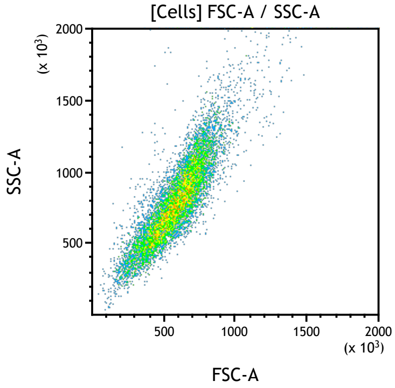


16_CD4448_RAPA 16.4
[Cells] IP PE-A

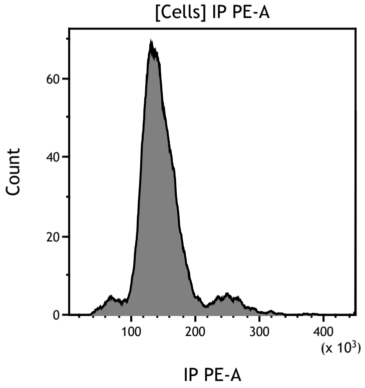

# New Composite 7 - Plot Sheet 5

16_CD4448_RAPA 16.5
[Ungated] IP PE-A / SSC-A

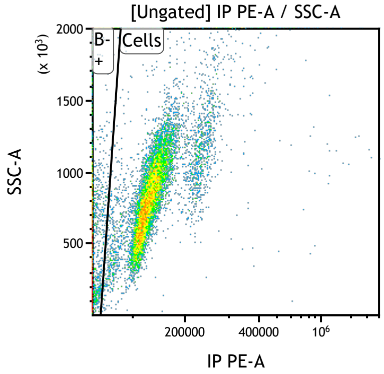


16_CD4448_RAPA 16.5
[Cells] FSC-A / SSC-A

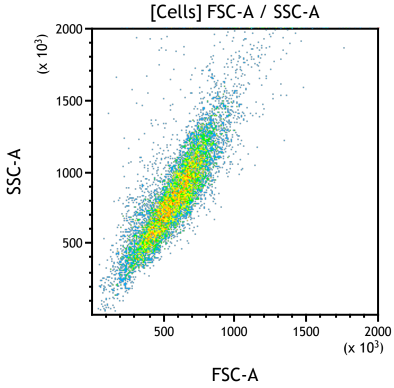


16_CD4448_RAPA 16.5
[Cells] IP PE-A

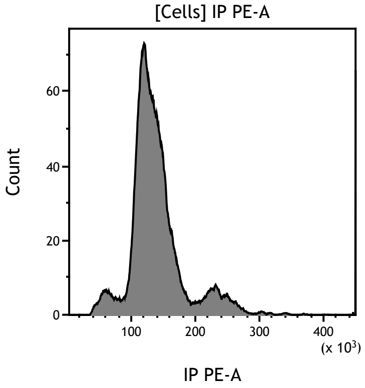

# New Composite 7 - Plot Sheet 6

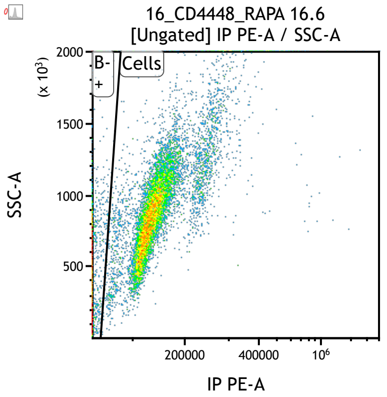


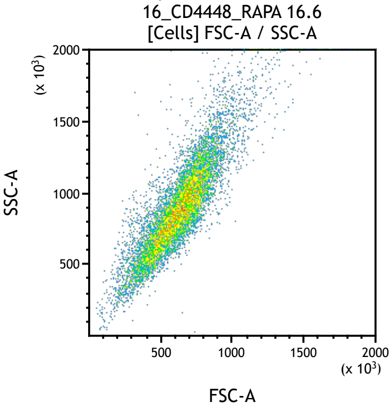


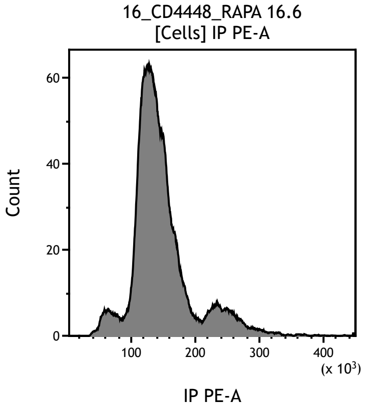

# New Composite 7 - Plot Sheet 7

16_CD4448_RAPA 16.7
[Ungated] IP PE-A / SSC-A

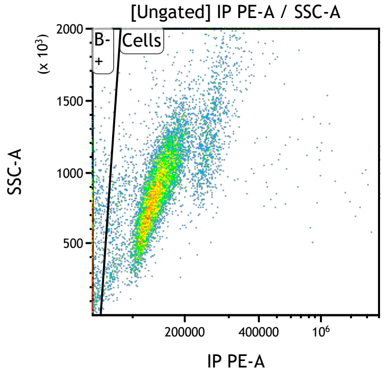


16_CD4448_RAPA 16.7
[Cells] FSC-A / SSC-A

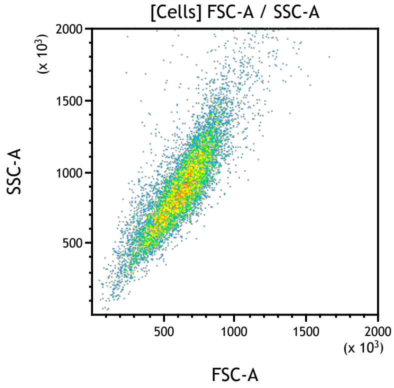


16_CD4448_RAPA 16.7
[Cells] IP PE-A

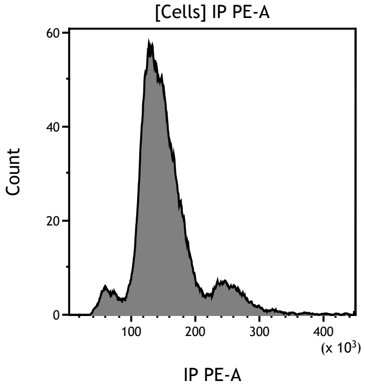

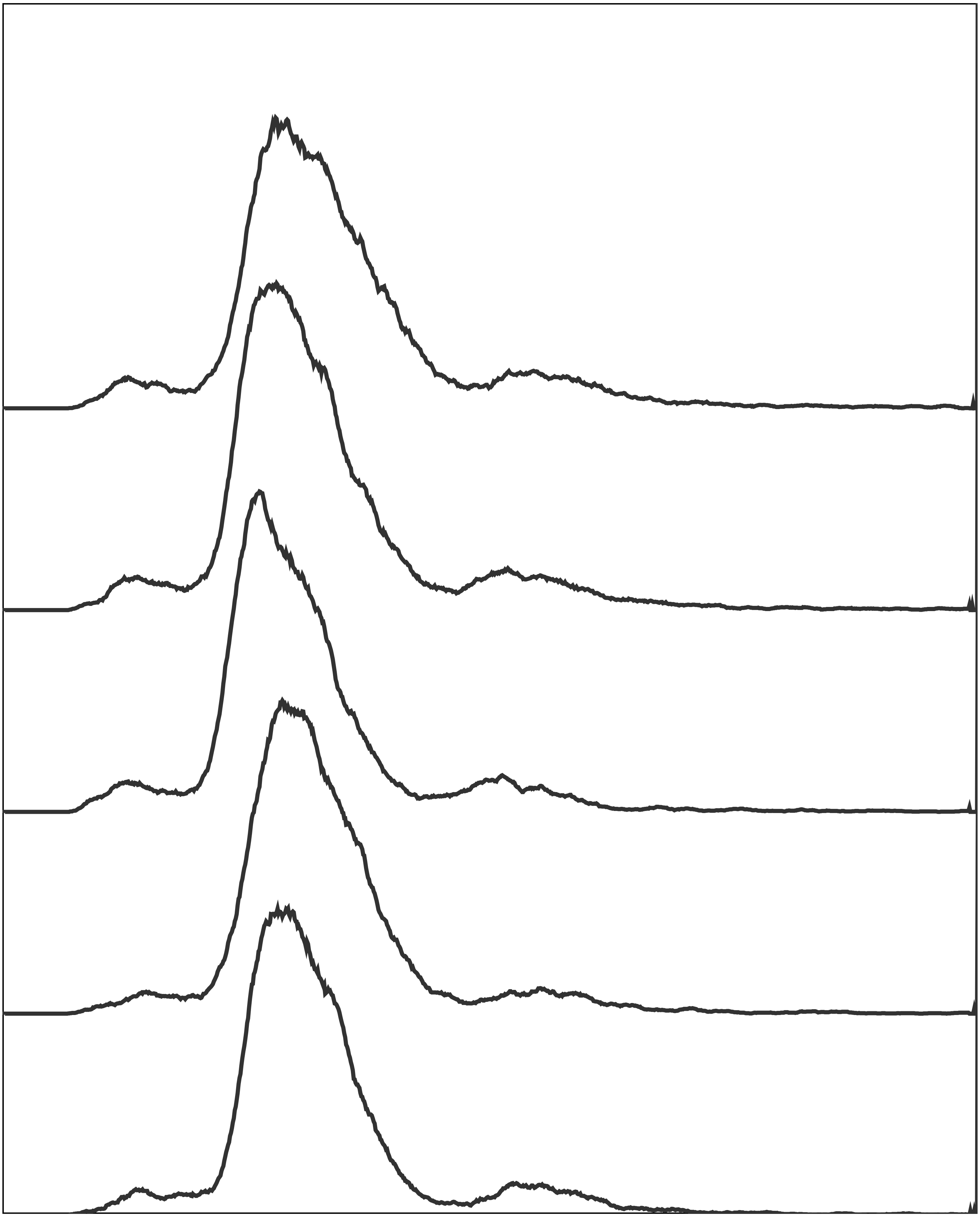

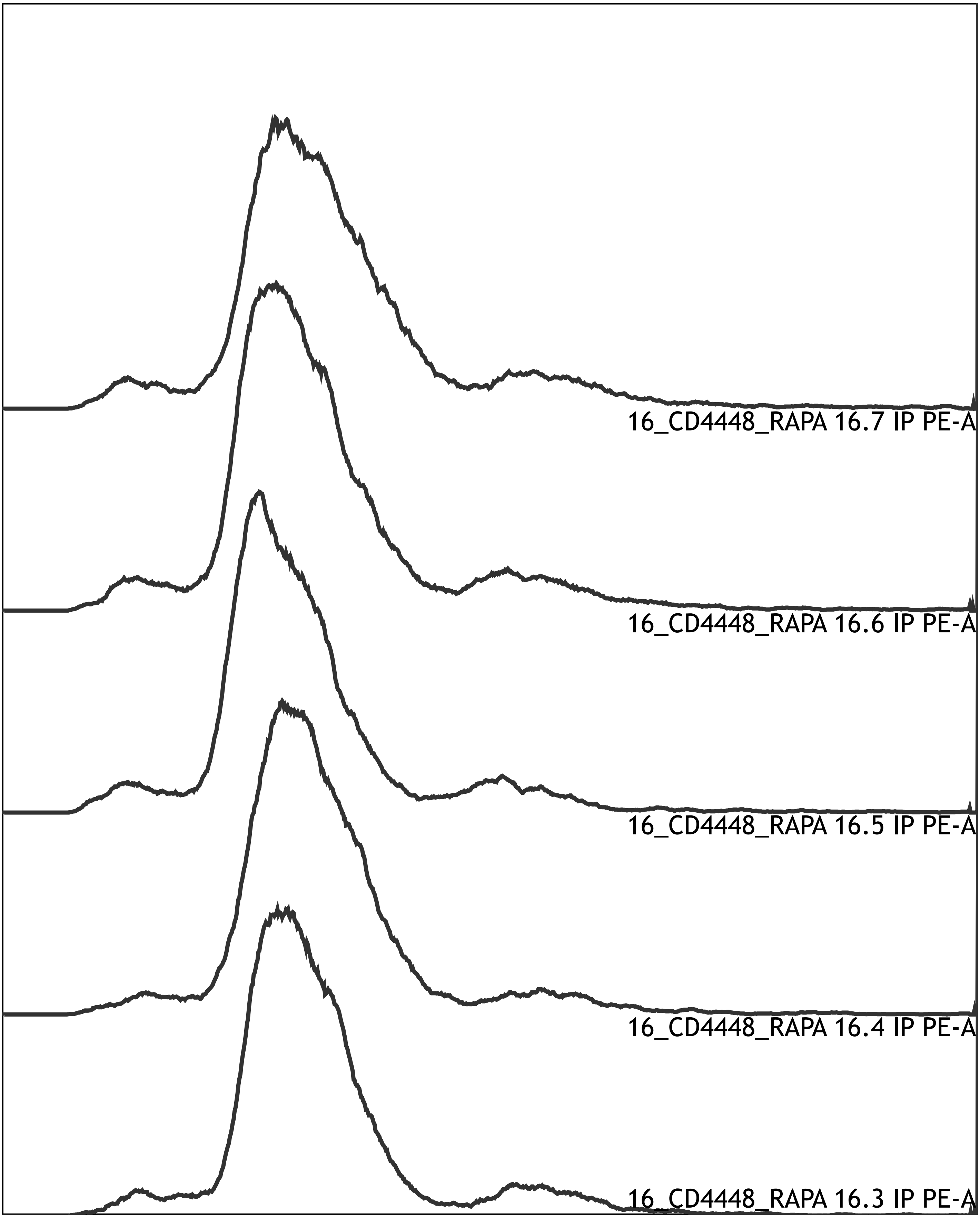
16_CD4448_RAPA 16.7 IP PE-A
16_CD4448_RAPA 16.6 IP PE-A
16_CD4448_RAPA 16.5 IP PE-A
16_CD4448_RAPA 16.4 IP PE-A
16_CD4448_RAPA 16.3 IP PE-A
