# Supplementary material for: TOR complex 1 negatively regulates NDR kinase Cbk1 to control cell separation in budding yeast
Source: PLoS Biol. 2023 Aug 30;21(8):e3002263. doi: 10.1371/journal.pbio.3002263 (PMC10468069; doi:10.1371/journal.pbio.3002263)
Supplement: S1 File — (ZIP) [file pbio.3002263.s024.zip › 4C.pdf]

New Composite 4 - Plot Sheet 3

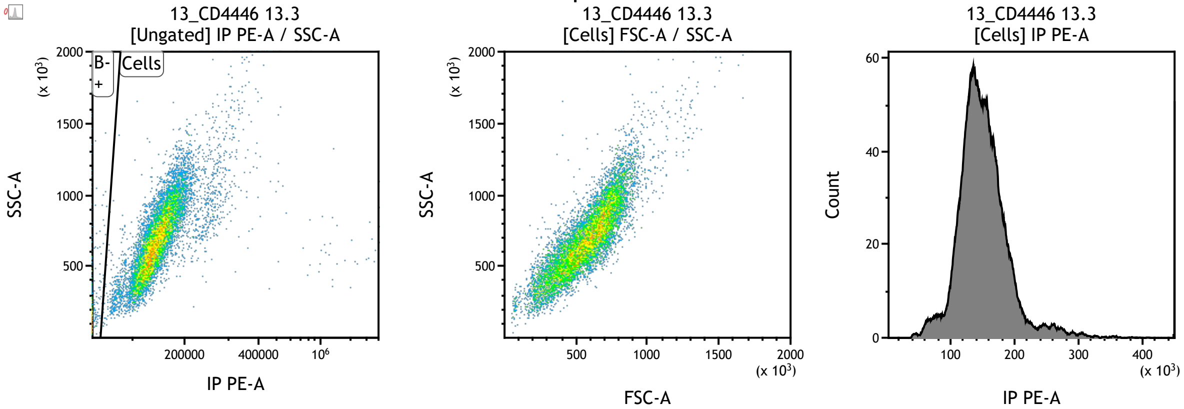

New Composite 4 - Plot Sheet 4

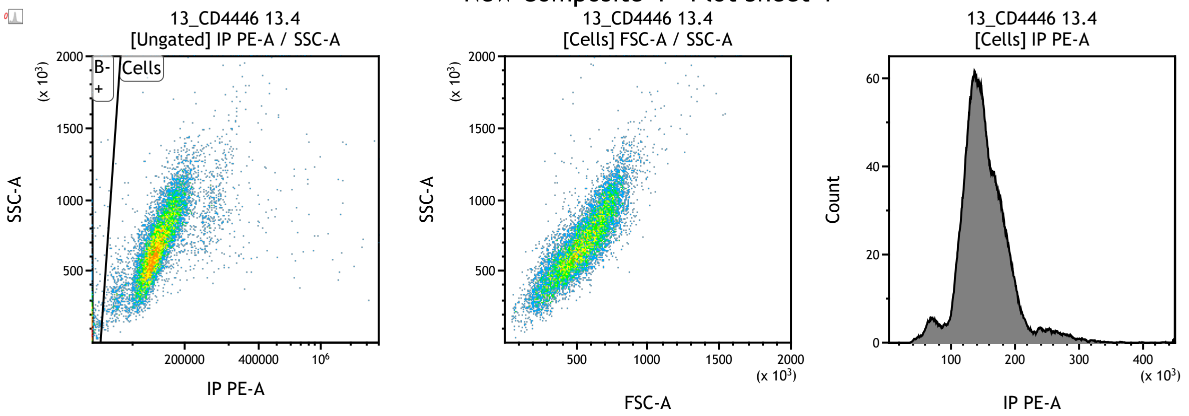

New Composite 4 - Plot Sheet 5

13_CD4446 13.5
[Ungated] IP PE-A / SSC-A

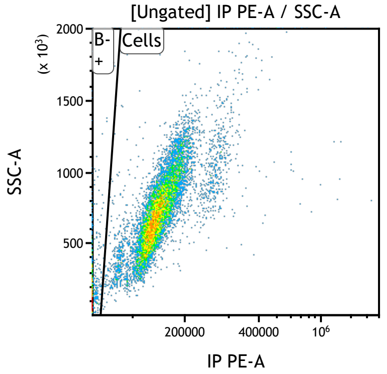

13_CD4446 13.5
[Cells] FSC-A / SSC-A

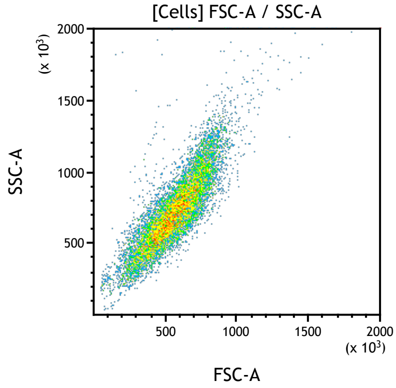

13_CD4446 13.5
[Cells] IP PE-A

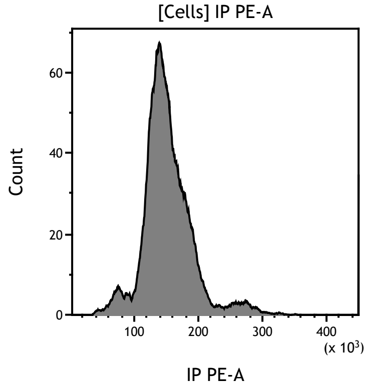

New Composite 4 - Plot Sheet 6

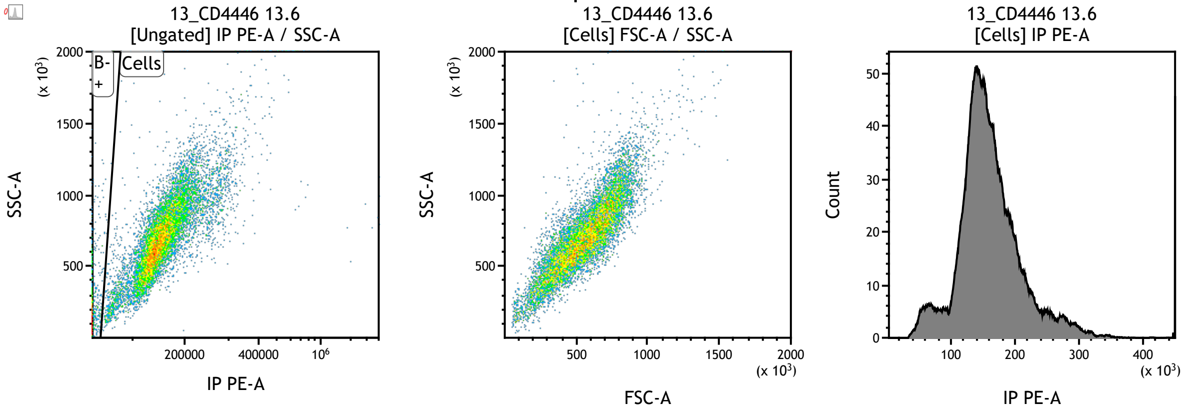

New Composite 4 - Plot Sheet 7

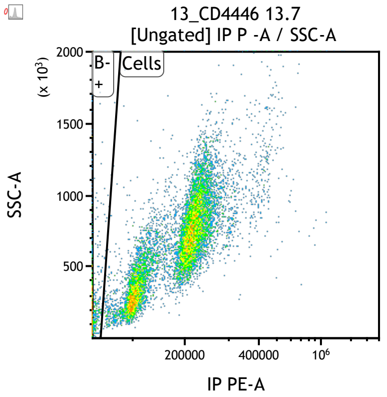

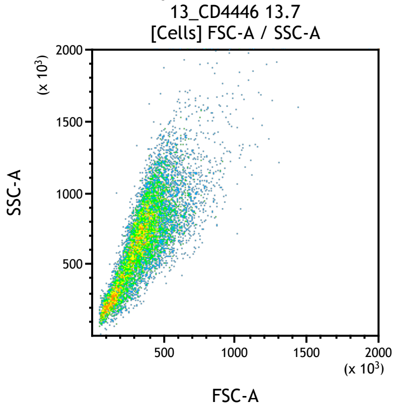

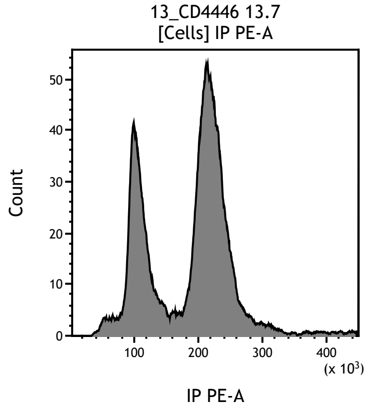

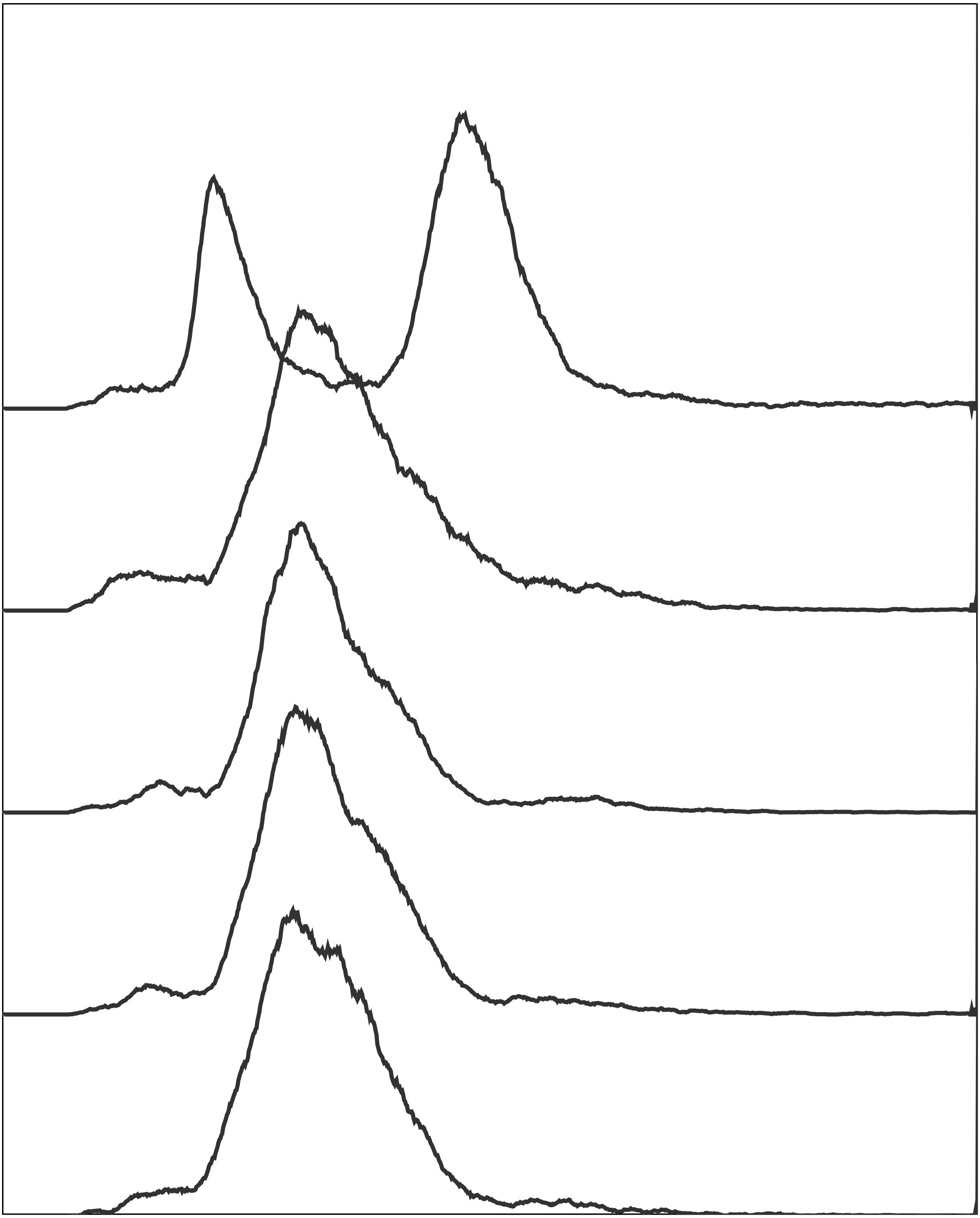

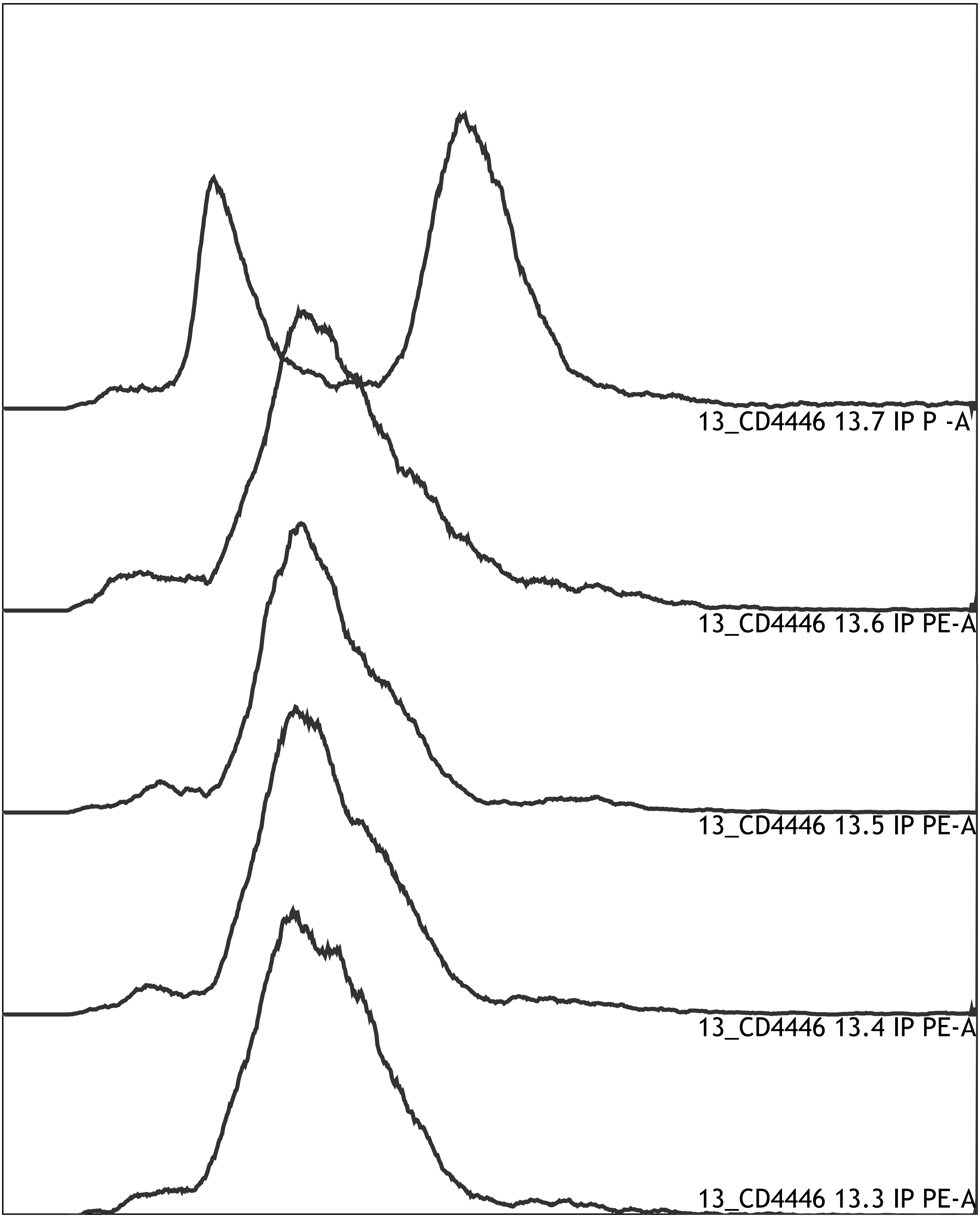
13_CD4446 13.7 IP P -A
13_CD4446 13.6 IP PE-A
13_CD4446 13.5 IP PE-A
13_CD4446 13.4 IP PE-A
13_CD4446 13.3 IP PE-A

New Composite 5 - Plot Sheet 3

14_CD4446_RAPA 14.3
[Ungated] IP PE-A / SSC-A

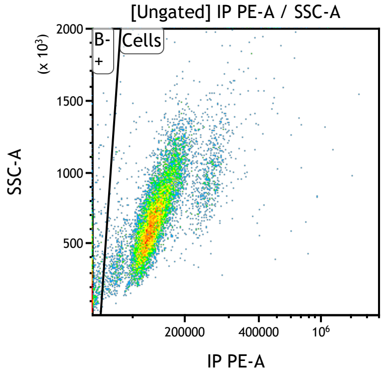

14_CD4446_RAPA 14.3
[Cells] FSC-A / SSC-A

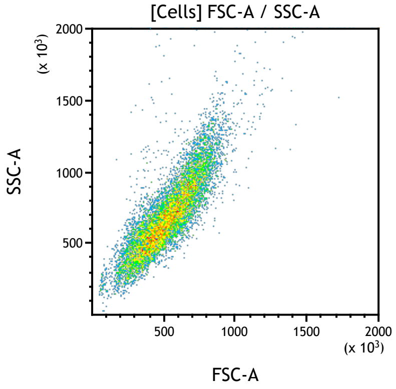

14_CD4446_RAPA 14.3
[Cells] IP PE-A

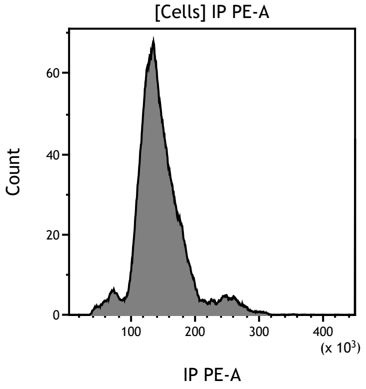

New Composite 5 - Plot Sheet 4

14_CD4446_RAPA 14.4
[Ungated] IP PE-A / SSC-A

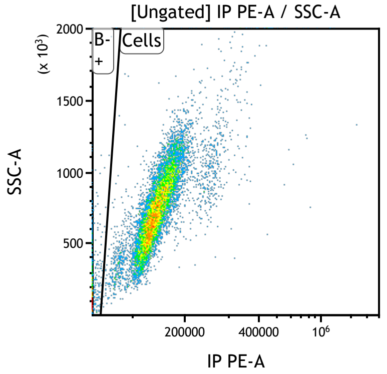

14_CD4446_RAPA 14.4
[Cells] FSC-A / SSC-A

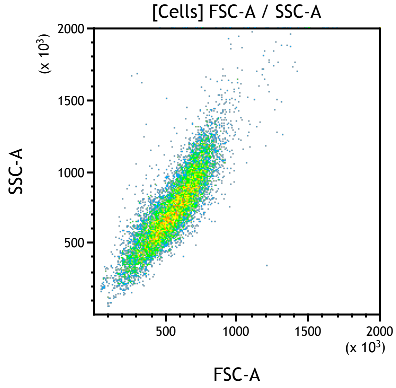

14_CD4446_RAPA 14.4
[Cells] IP PE-A

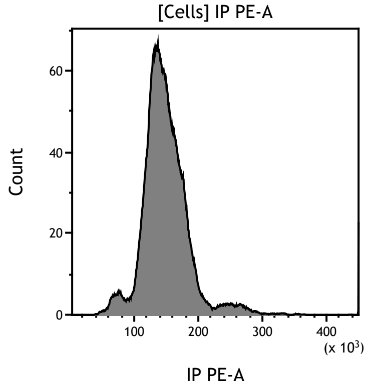

New Composite 5 - Plot Sheet 5

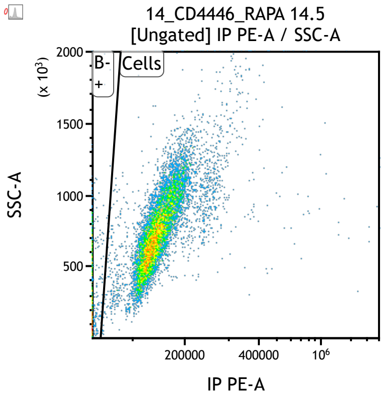

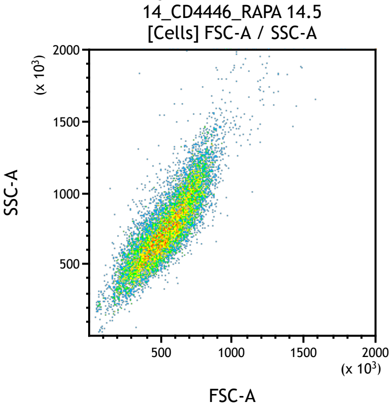

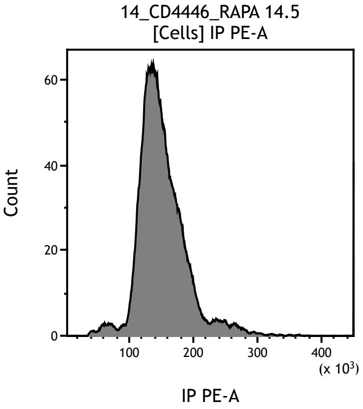

New Composite 5 - Plot Sheet 6

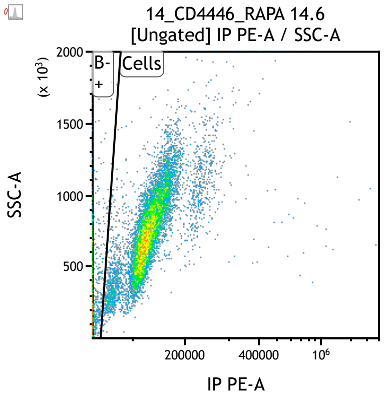

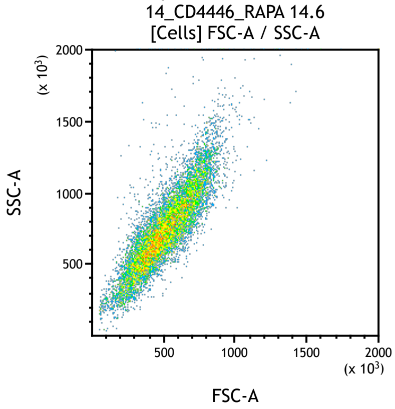

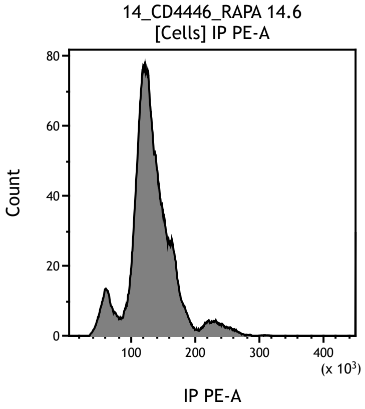

New Composite 5 - Plot Sheet 7

14_CD4446_RAPA 14.7
[Ungated] IP P -A / SSC-A

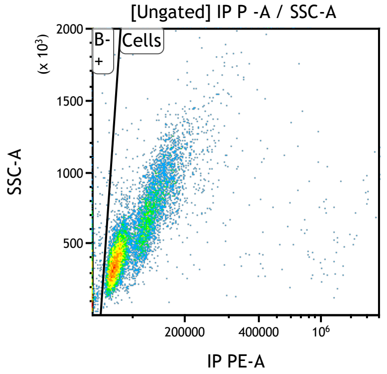

14_CD4446_RAPA 14.7
[Cells] FSC-A / SSC-A

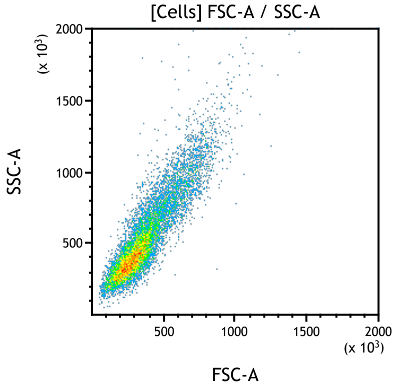

14_CD4446_RAPA 14.7
[Cells] IP P -A

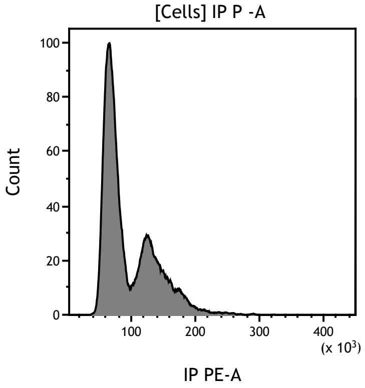

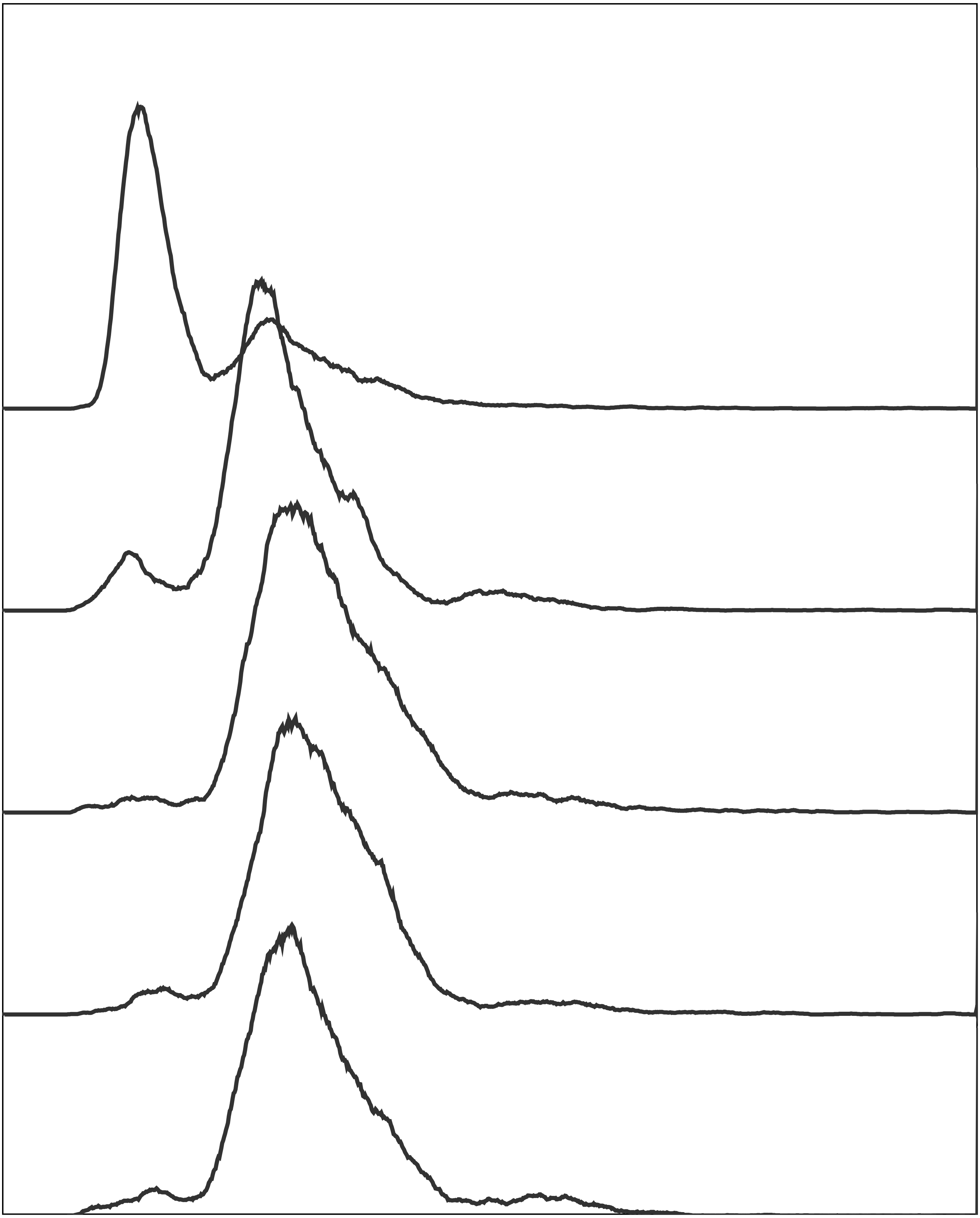

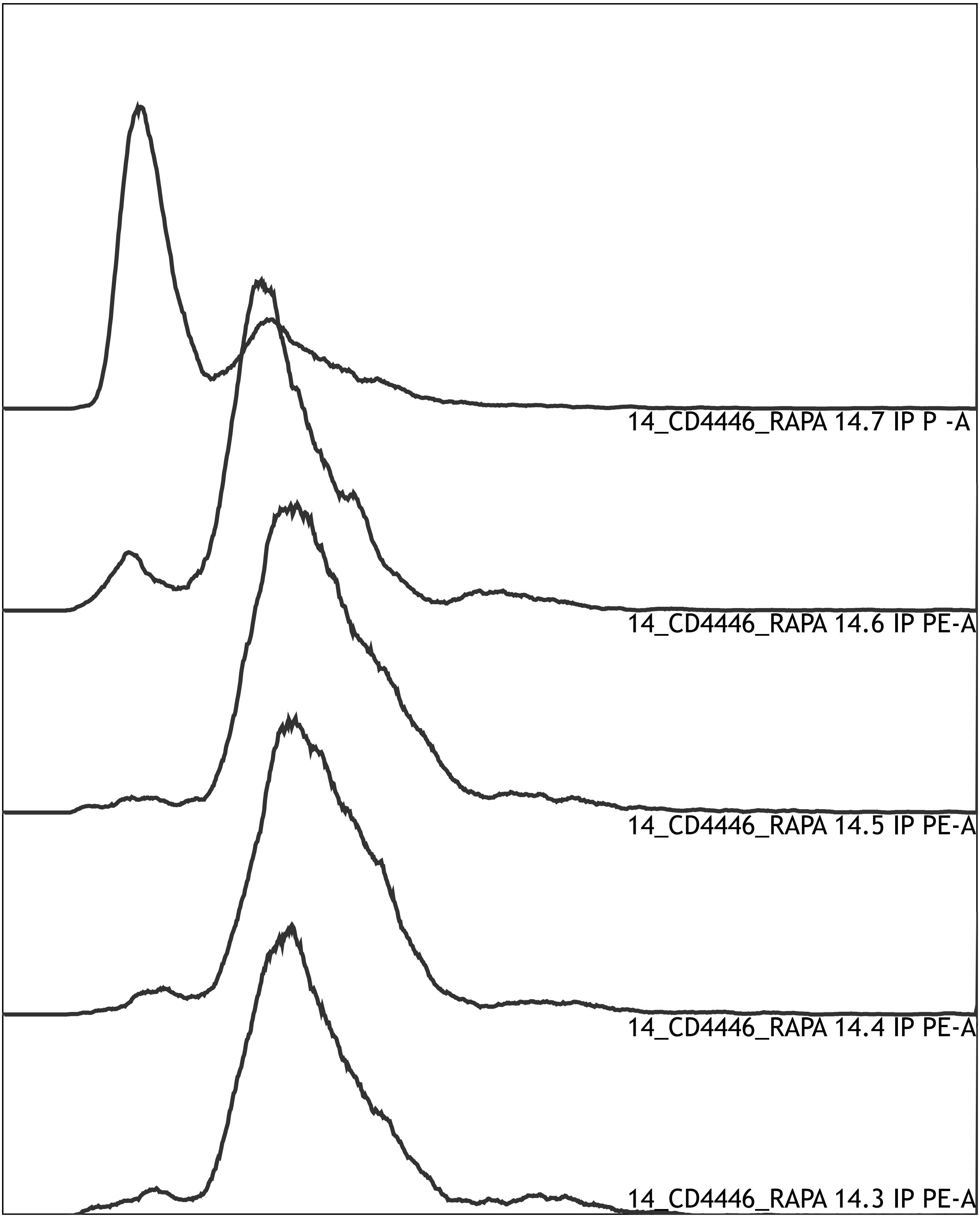
14_CD4446_RAPA 14.7 IP P -A
14_CD4446_RAPA 14.6 IP PE-A
14_CD4446_RAPA 14.5 IP PE-A
14_CD4446_RAPA 14.4 IP PE-A
14_CD4446_RAPA 14.3 IP PE-A
