# Supplementary material for: TOR complex 1 negatively regulates NDR kinase Cbk1 to control cell separation in budding yeast
Source: PLoS Biol. 2023 Aug 30;21(8):e3002263. doi: 10.1371/journal.pbio.3002263 (PMC10468069; doi:10.1371/journal.pbio.3002263)
Supplement: S1 File — (ZIP) [file pbio.3002263.s024.zip › 4D.pdf]

New Composite 2 - Plot Sheet 3

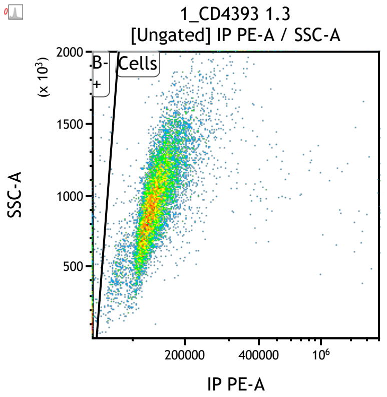

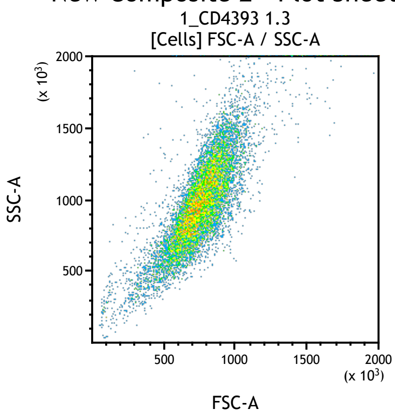

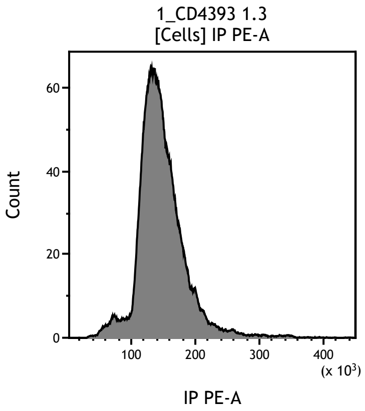

New Composite 2 - Plot Sheet 4

1_CD4393 1.4
[Ungated] IP PE-A / SSC-A

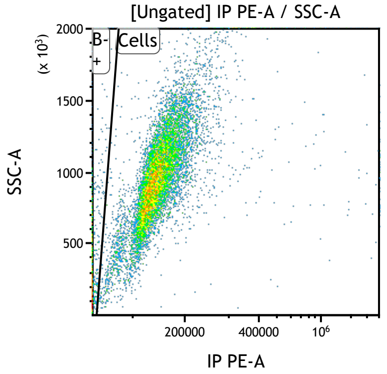

1_CD4393 1.4
[Cells] FSC-A / SSC-A

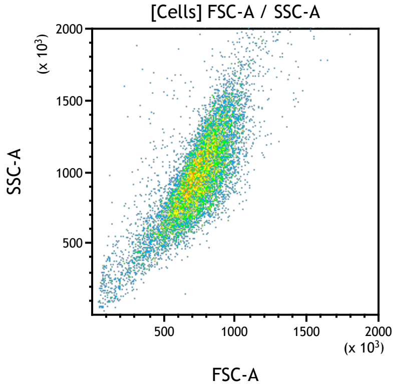

1_CD4393 1.4
[Cells] IP PE-A

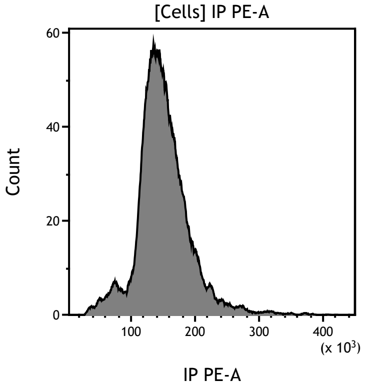

New Composite 2 - Plot Sheet 5

1_CD4393 1.5
[Ungated] IP PE-A / SSC-A

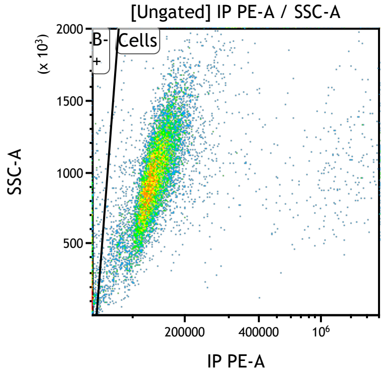

1_CD4393 1.5
[Cells] FSC-A / SSC-A

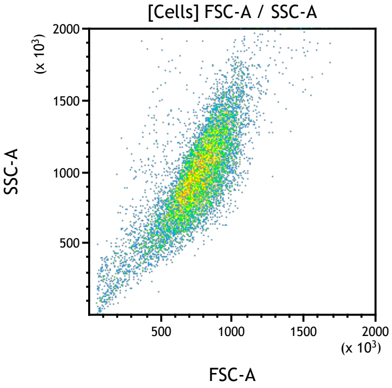

1_CD4393 1.5
[Cells] IP PE-A

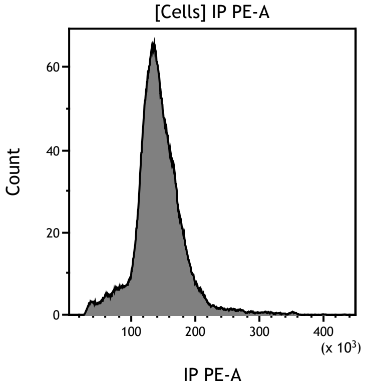

New Composite 2 - Plot Sheet 6

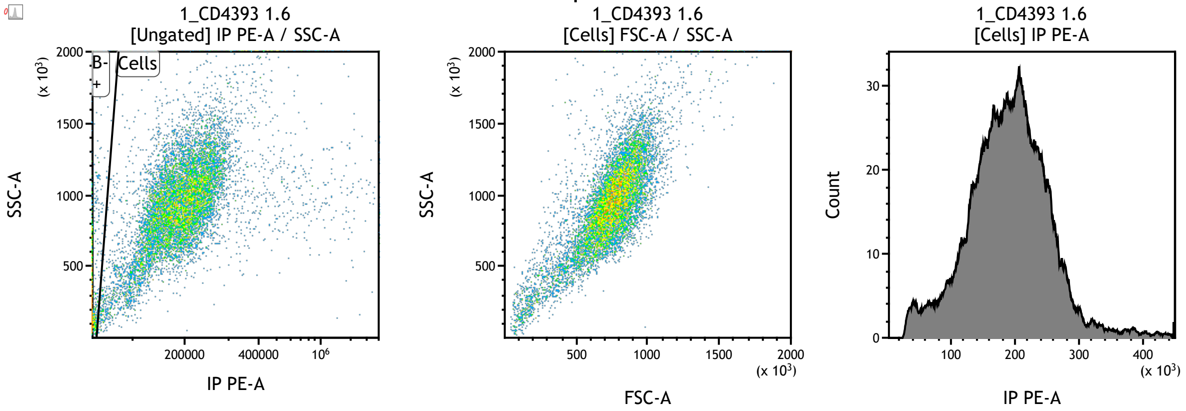

New Composite 2 - Plot Sheet 7

1_CD4393 1.7
[Ungated] IP PE-A / SSC-A

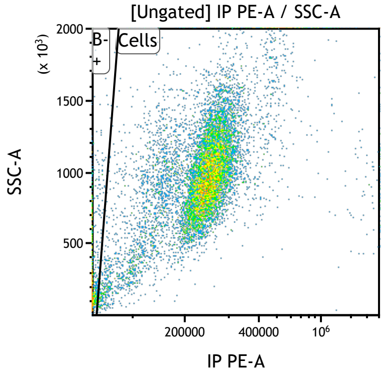

1_CD4393 1.7
[Cells] FSC-A / SSC-A

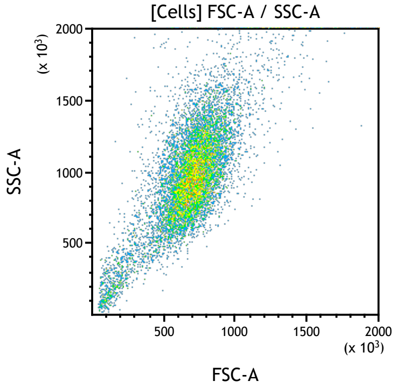

1_CD4393 1.7
[Cells] IP PE-A

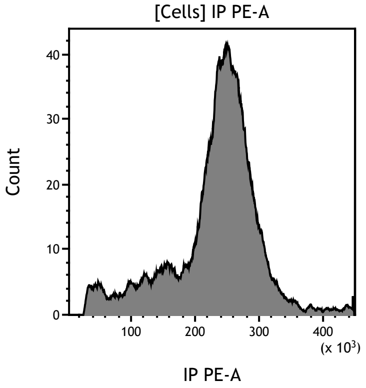

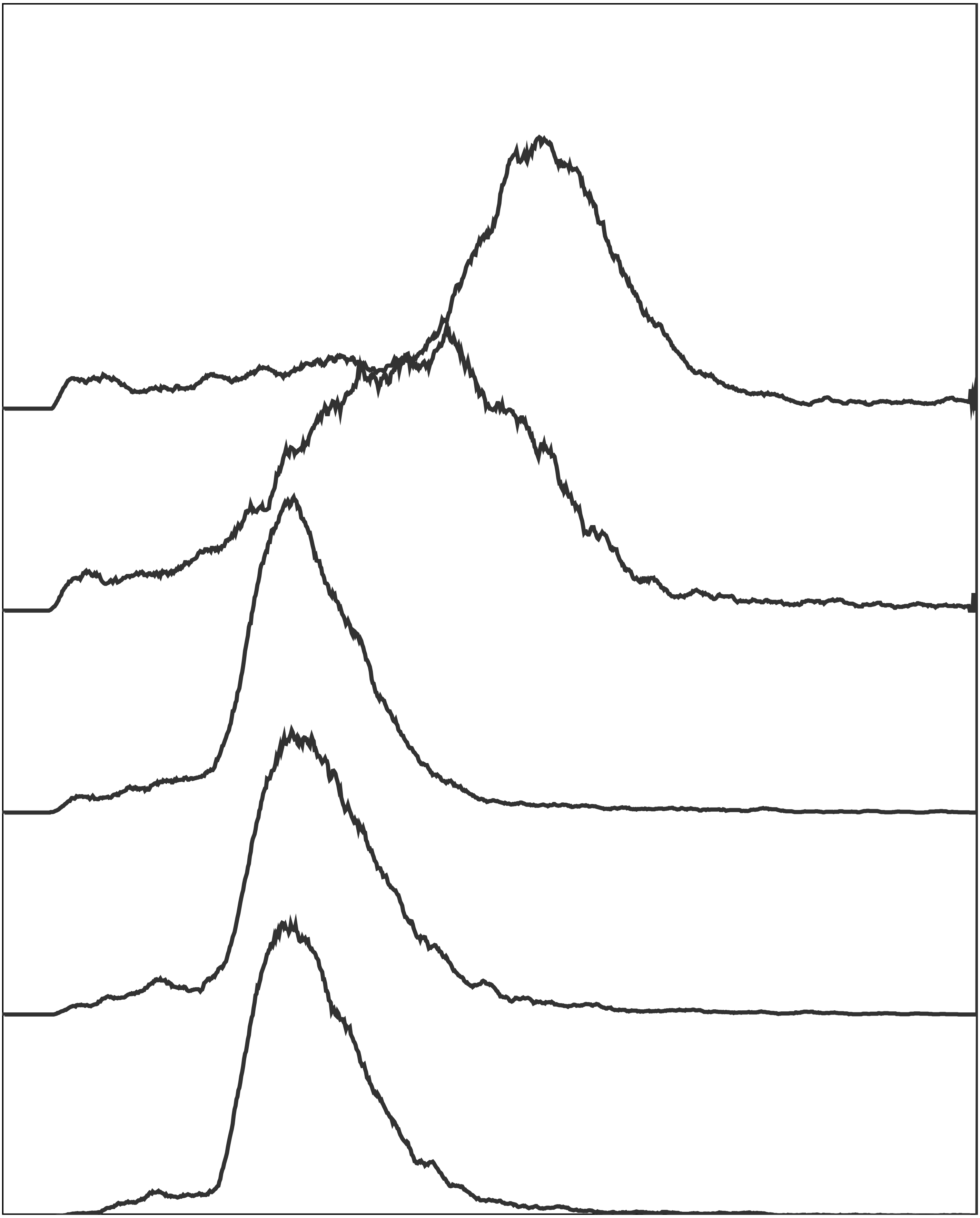

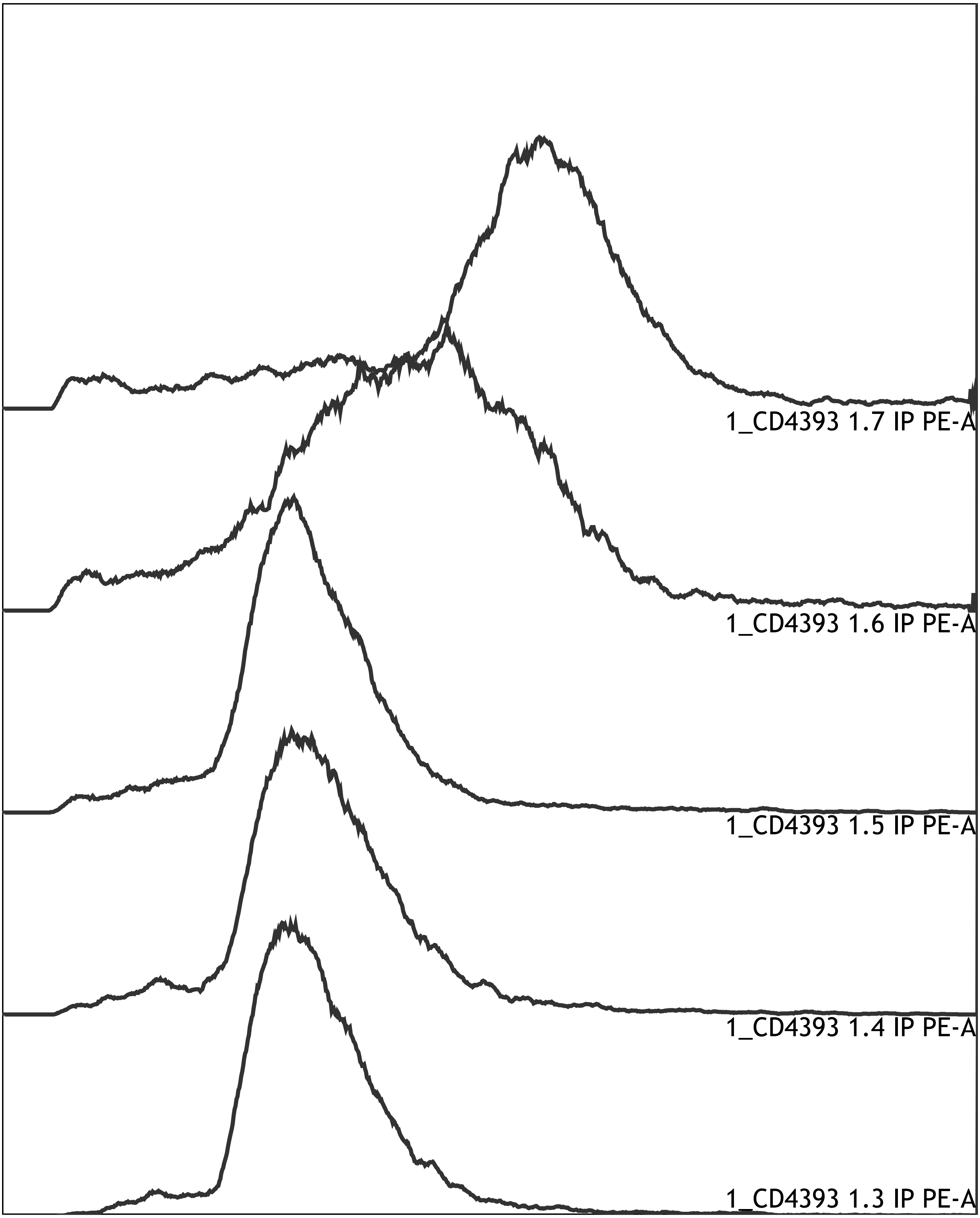
1_CD4393 1.7 IP PE-A
1_CD4393 1.6 IP PE-A
1_CD4393 1.5 IP PE-A
1_CD4393 1.4 IP PE-A
1_CD4393 1.3 IP PE-A

New Composite 3 - Plot Sheet 3

2_CD4393_RAPA 2.3
[Ungated] IP PE-A / SSC-A

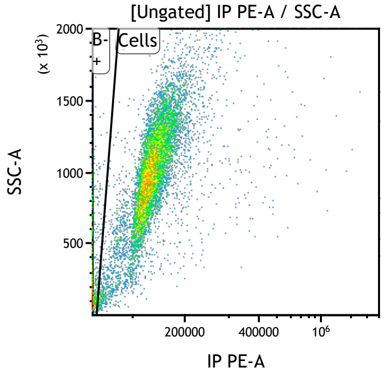

2_CD4393_RAPA 2.3
[Cells] FSC-A / SSC-A

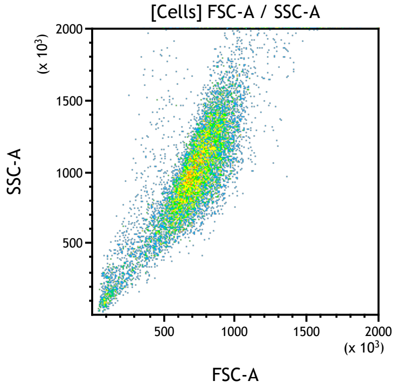

2_CD4393_RAPA 2.3
[Cells] IP PE-A

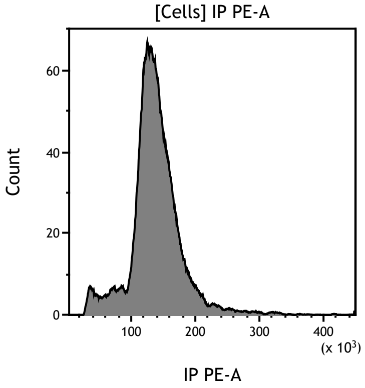

New Composite 3 - Plot Sheet 4

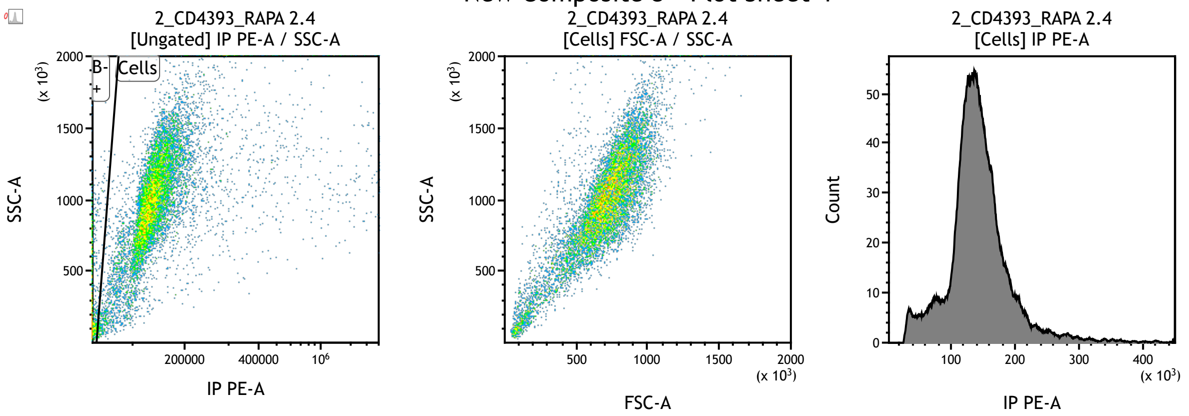

New Composite 3 - Plot Sheet 5

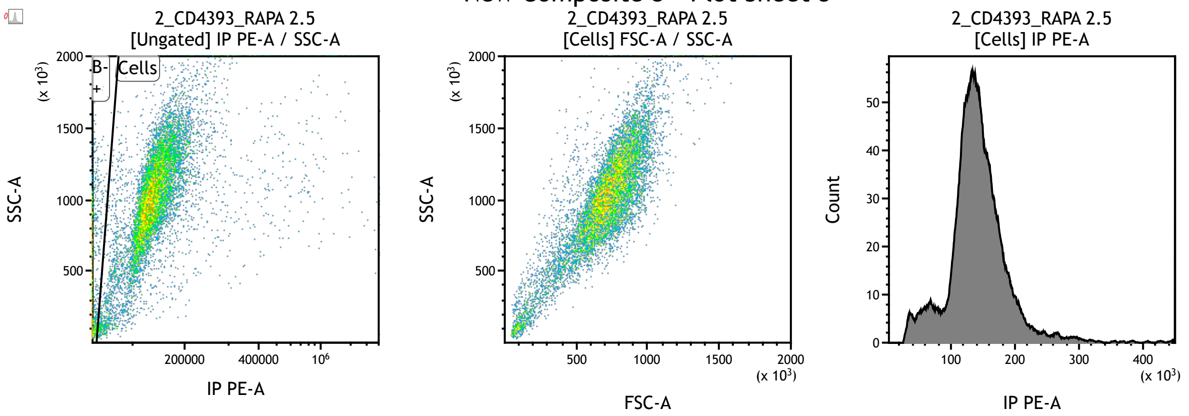

New Composite 3 - Plot Sheet 6

2_CD4393_RAPA 2.6
[Ungated] IP PE-A / SSC-A

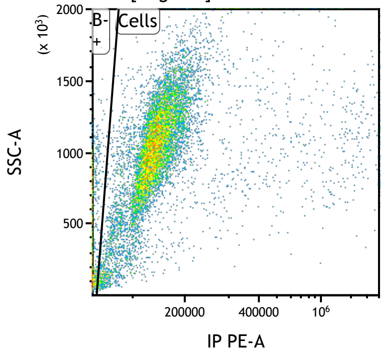

2_CD4393_RAPA 2.6
[Cells] FSC-A / SSC-A

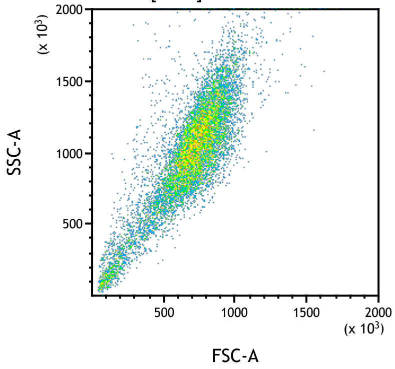

2_CD4393_RAPA 2.6
[Cells] IP PE-A

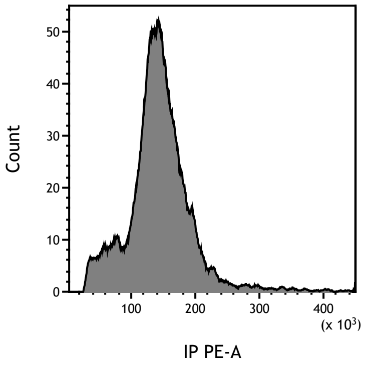

New Composite 3 - Plot Sheet 7

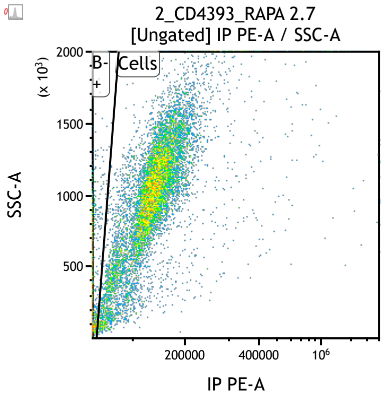

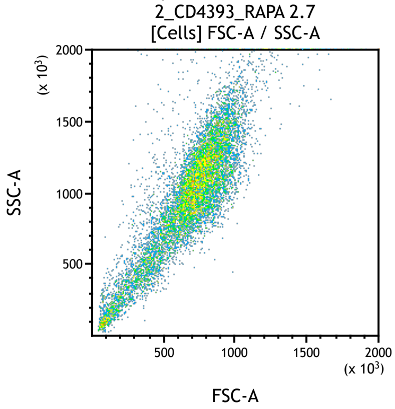

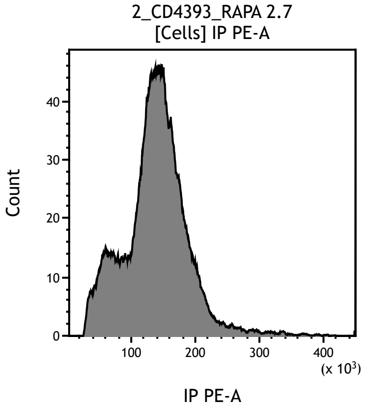

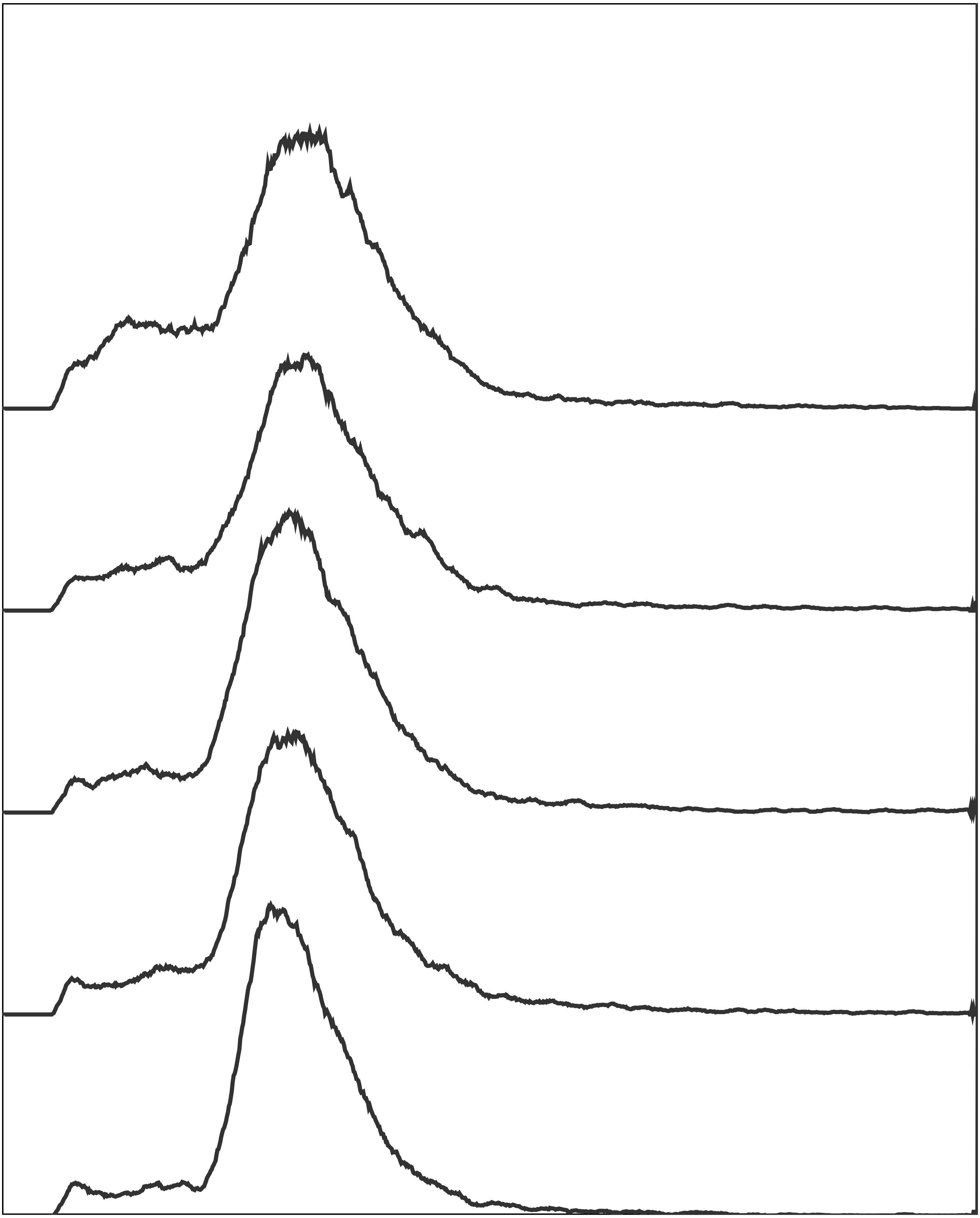

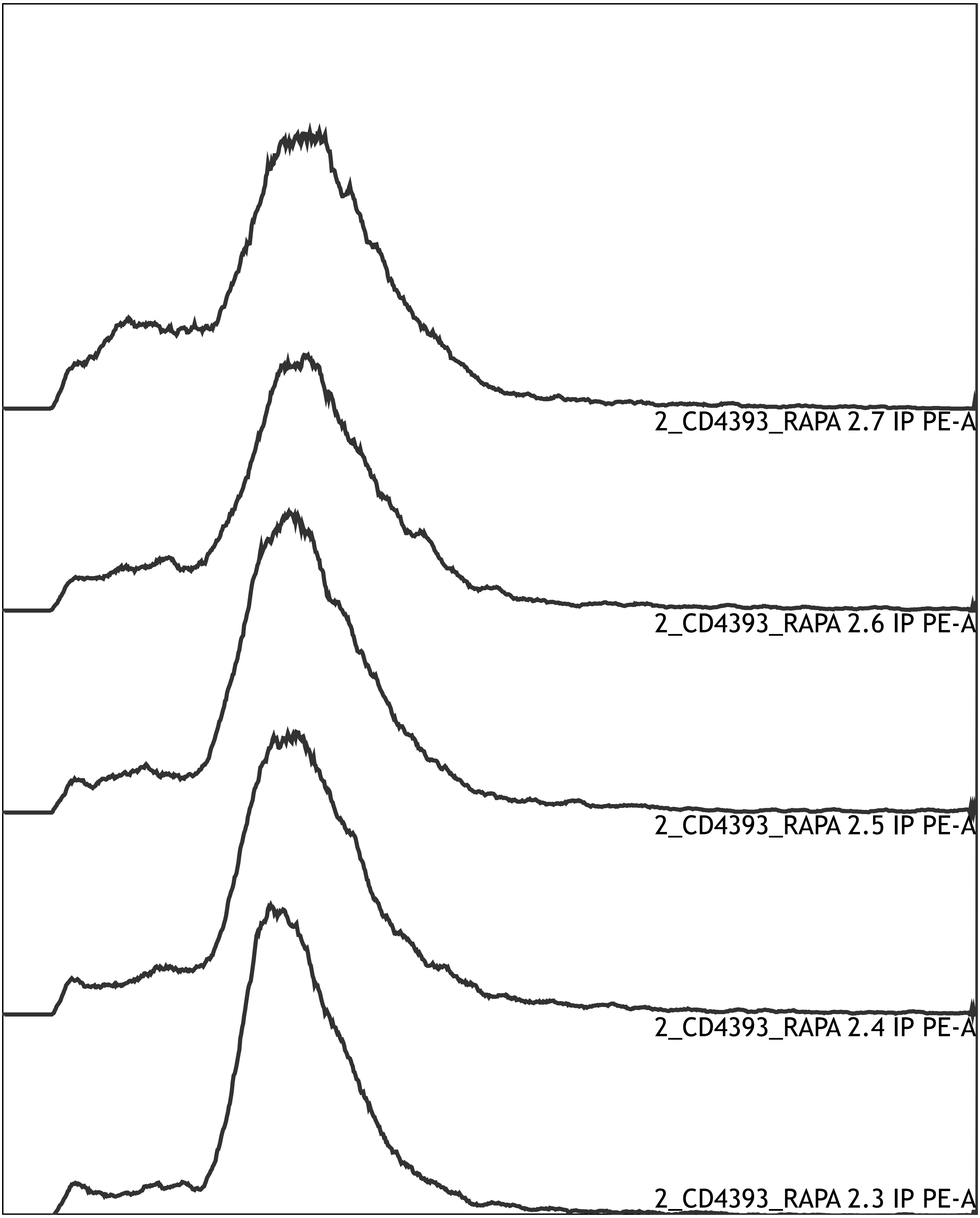
2_CD4393_RAPA 2.7 IP PE-A
2_CD4393_RAPA 2.6 IP PE-A
2_CD4393_RAPA 2.5 IP PE-A
2_CD4393_RAPA 2.4 IP PE-A
2_CD4393_RAPA 2.3 IP PE-A
