# Supplementary material for: TOR complex 1 negatively regulates NDR kinase Cbk1 to control cell separation in budding yeast
Source: PLoS Biol. 2023 Aug 30;21(8):e3002263. doi: 10.1371/journal.pbio.3002263 (PMC10468069; doi:10.1371/journal.pbio.3002263)
Supplement: S1 File — (ZIP) [file pbio.3002263.s024.zip › 6A.pdf]

New Composite 2 - Plot Sheet 1

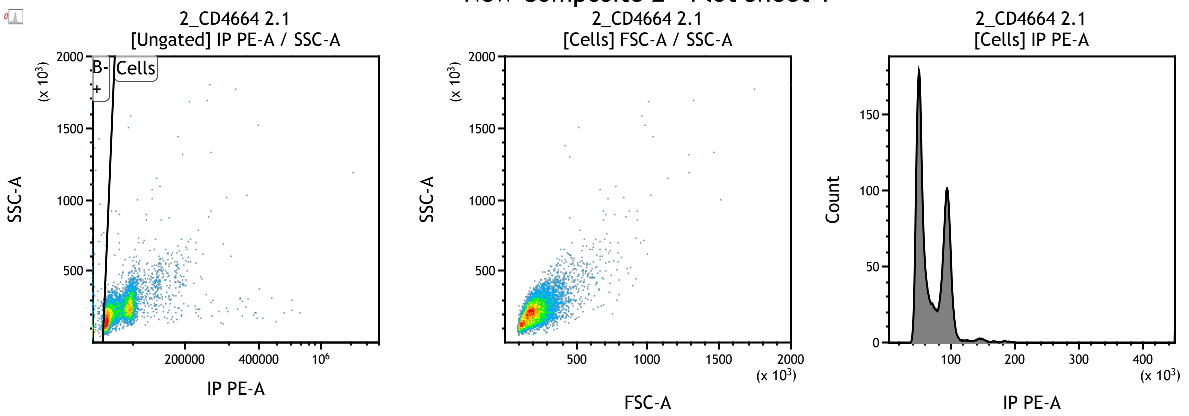

New Composite 2 - Plot Sheet 2

2_CD4664 2.2
[Ungated] IP PE-A / SSC-A

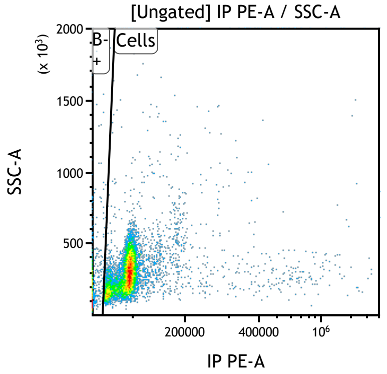

2_CD4664 2.2
[Cells] FSC-A / SSC-A

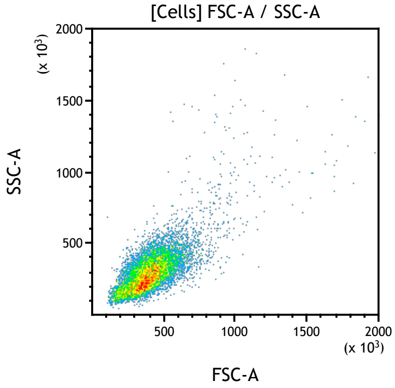

2_CD4664 2.2
[Cells] IP PE-A

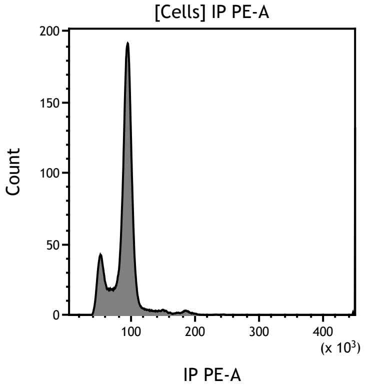

New Composite 2 - Plot Sheet 3

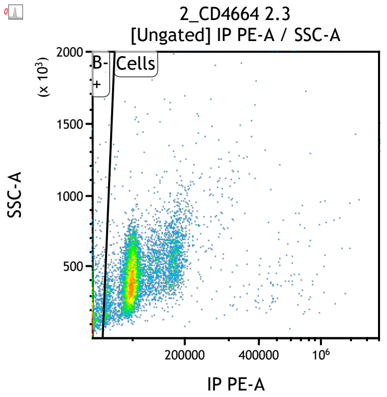

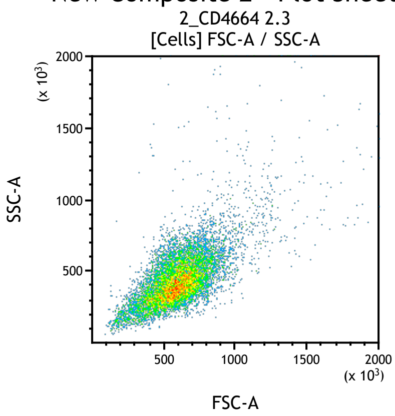

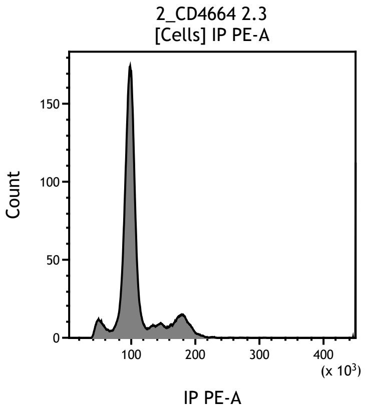

New Composite 2 - Plot Sheet 4

2_CD4664 2.4
[Ungated] IP PE-A / SSC-A

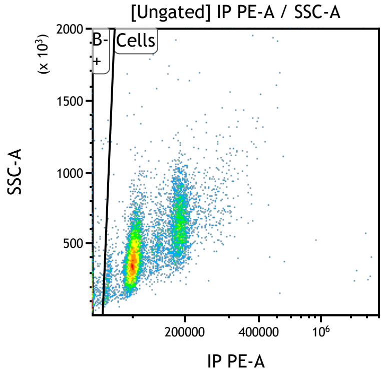

2_CD4664 2.4
[Cells] FSC-A / SSC-A

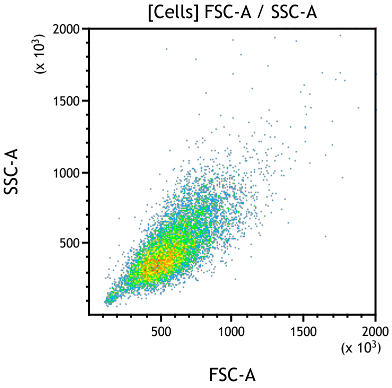

2_CD4664 2.4
[Cells] IP PE-A

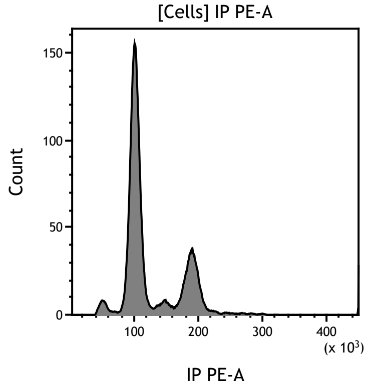

New Composite 2 - Plot Sheet 5

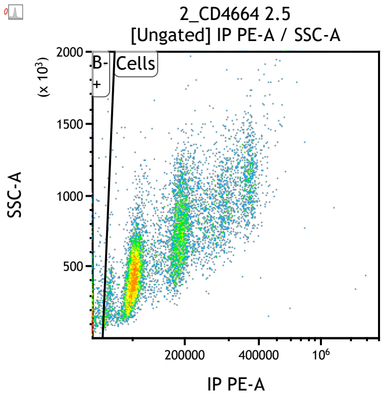

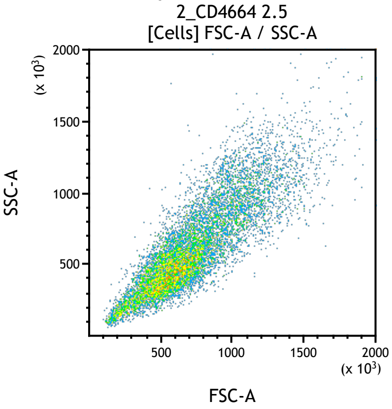

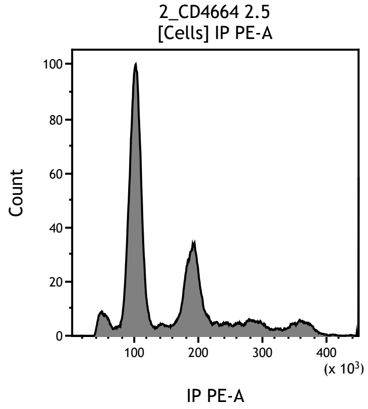

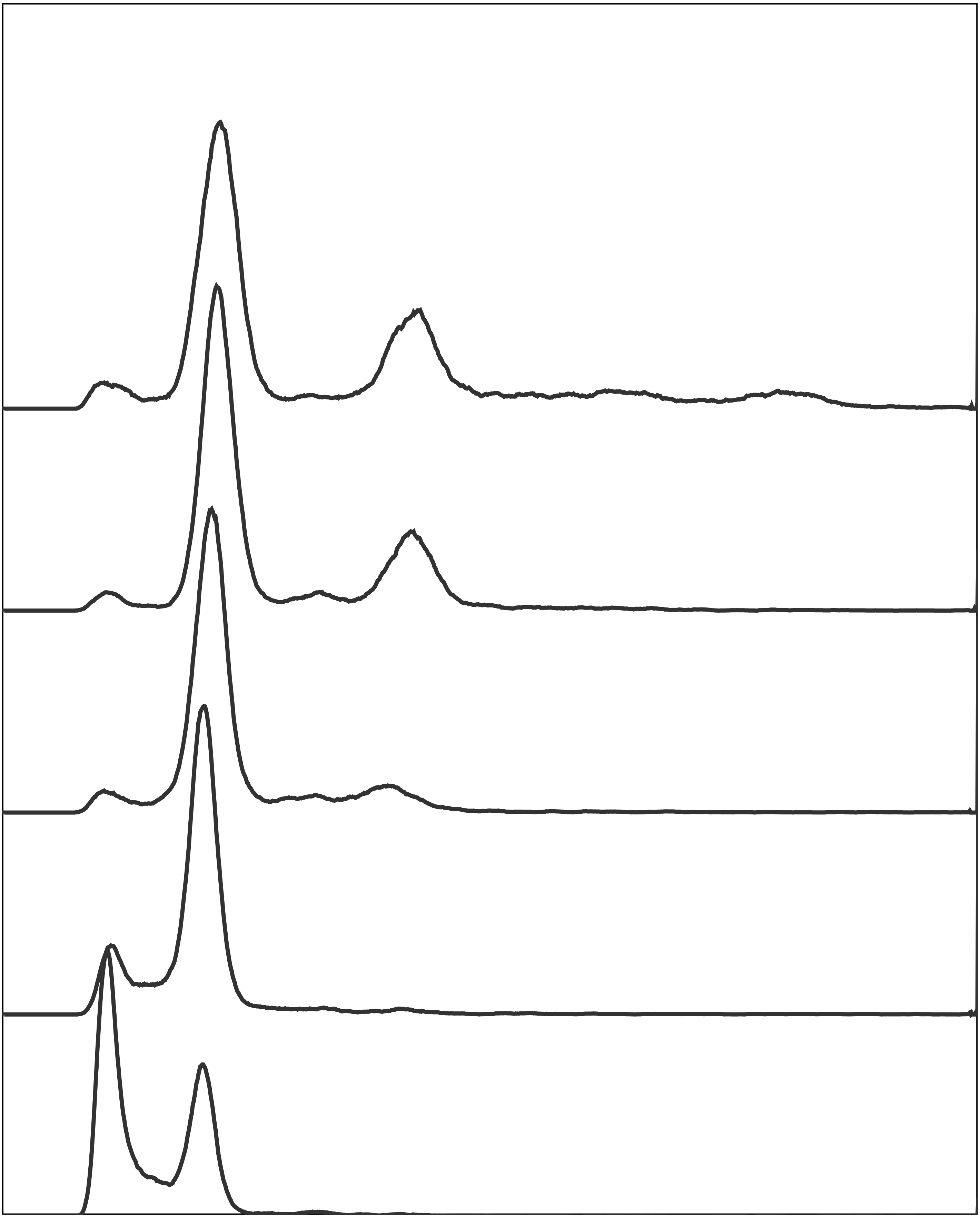

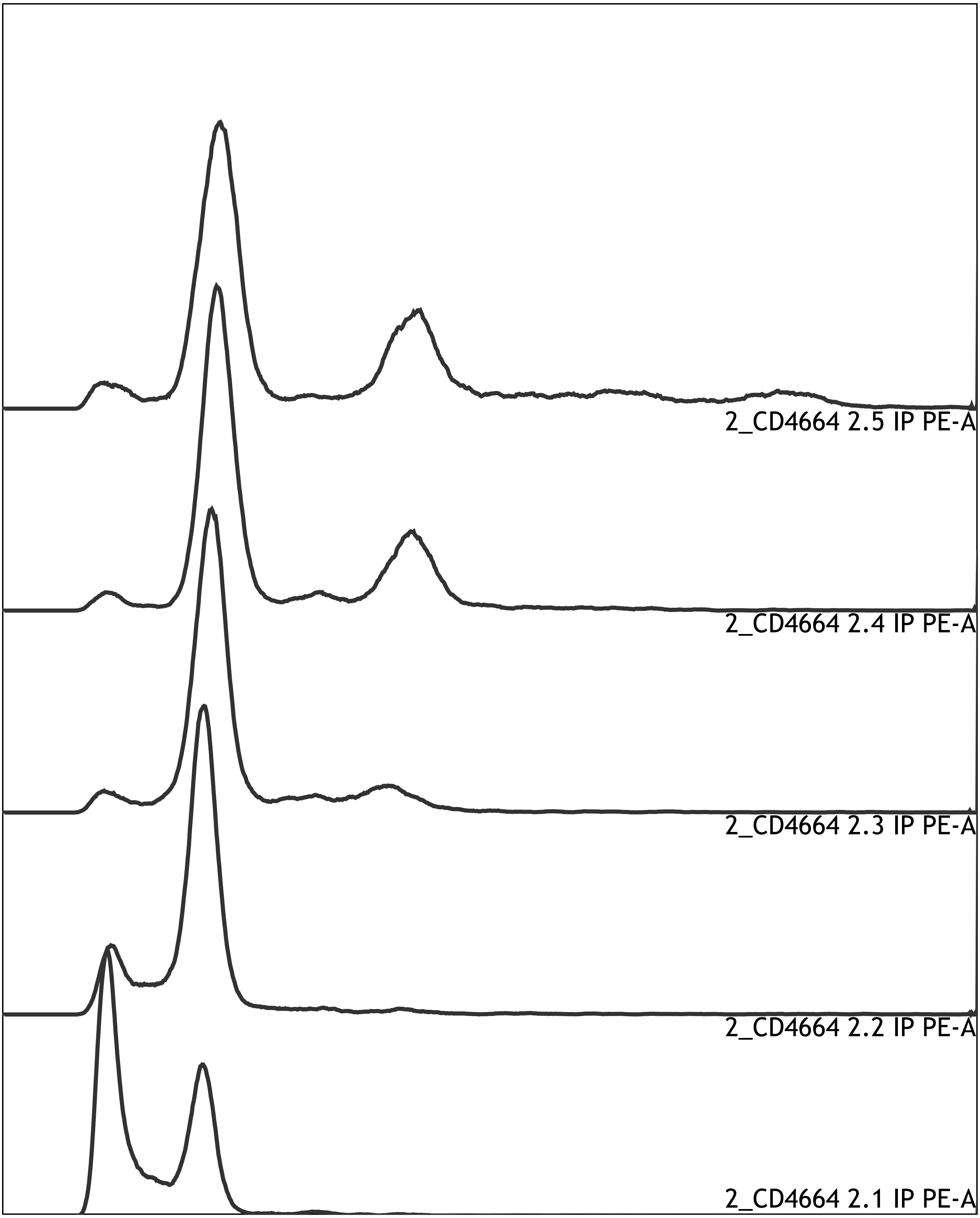
2_CD4664 2.5 IP PE-A
2_CD4664 2.4 IP PE-A
2_CD4664 2.3 IP PE-A
2_CD4664 2.2 IP PE-A
2_CD4664 2.1 IP PE-A

New Composite 4 - Plot Sheet 1

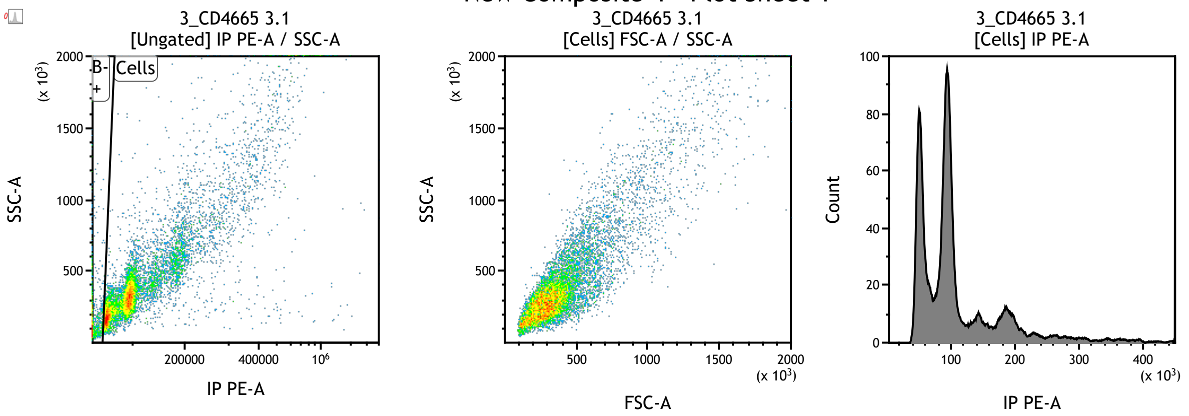

New Composite 4 - Plot Sheet 2

3_CD4665 3.2
[Ungated] IP PE-A / SSC-A

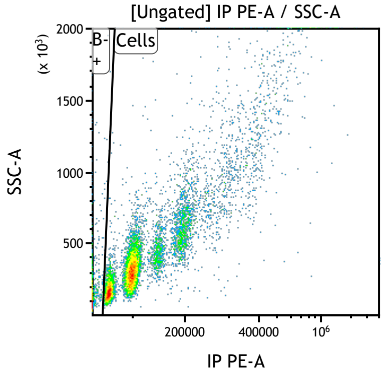

3_CD4665 3.2
[Cells] FSC-A / SSC-A

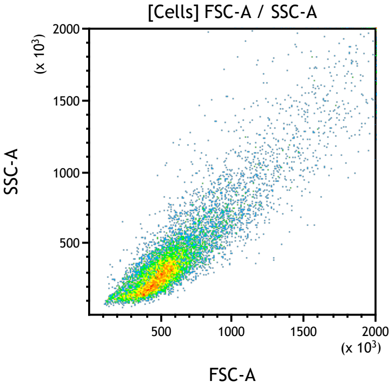

3_CD4665 3.2
[Cells] IP PE-A

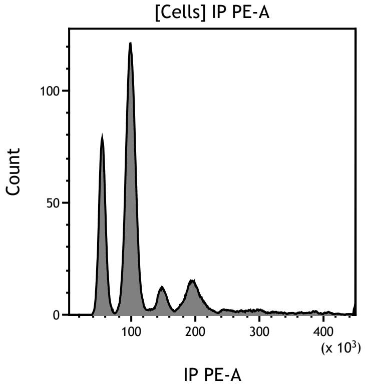

New Composite 4 - Plot Sheet 3

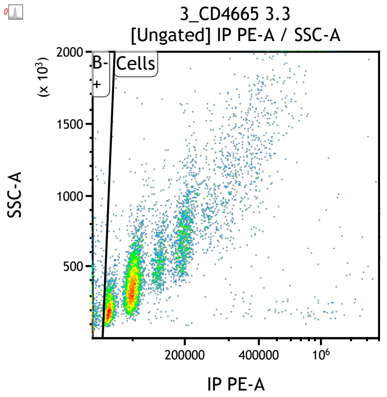

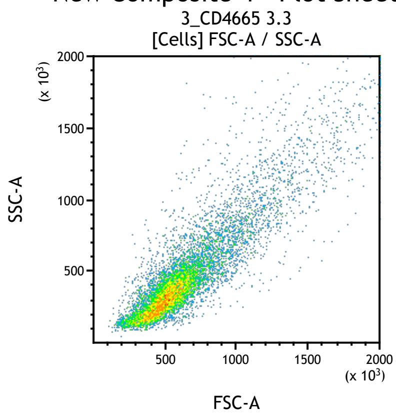

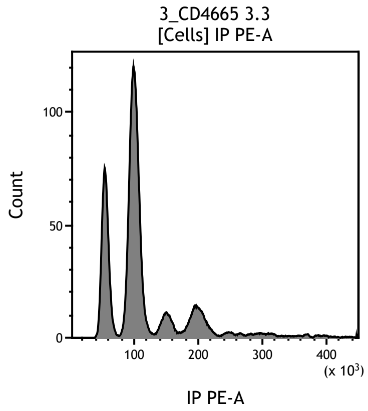

New Composite 4 - Plot Sheet 4

3_CD4665 3.4
[Ungated] IP PE-A / SSC-A

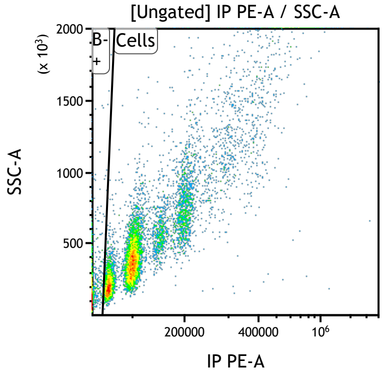

3_CD4665 3.4
[Cells] FSC-A / SSC-A

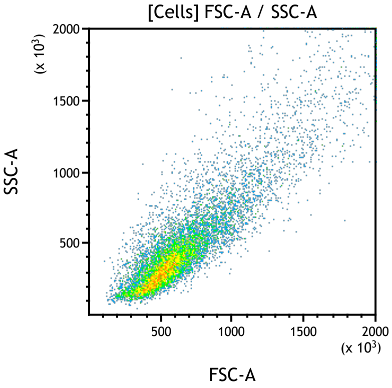

3_CD4665 3.4
[Cells] IP PE-A

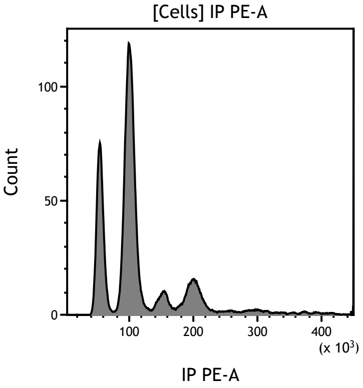

New Composite 4 - Plot Sheet 5

3_CD4665 3.5
[Ungated] IP PE-A / SSC-A

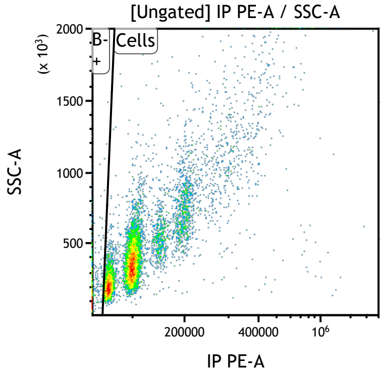

3_CD4665 3.5
[Cells] FSC-A / SSC-A

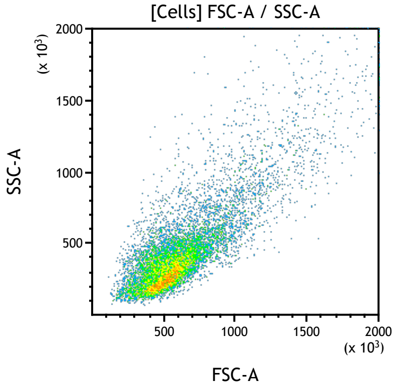

3_CD4665 3.5
[Cells] IP PE-A

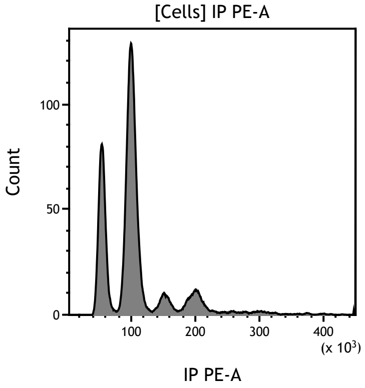

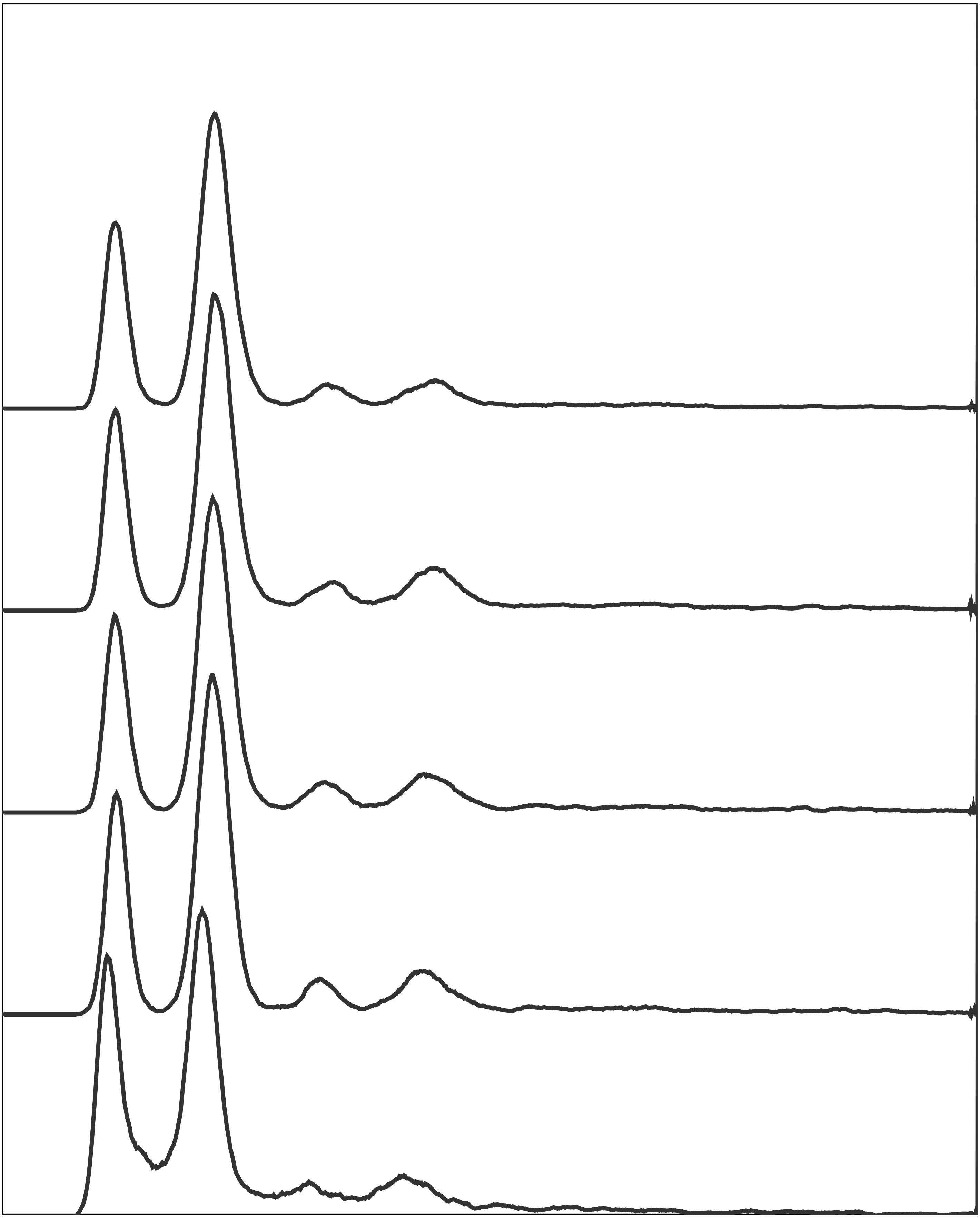

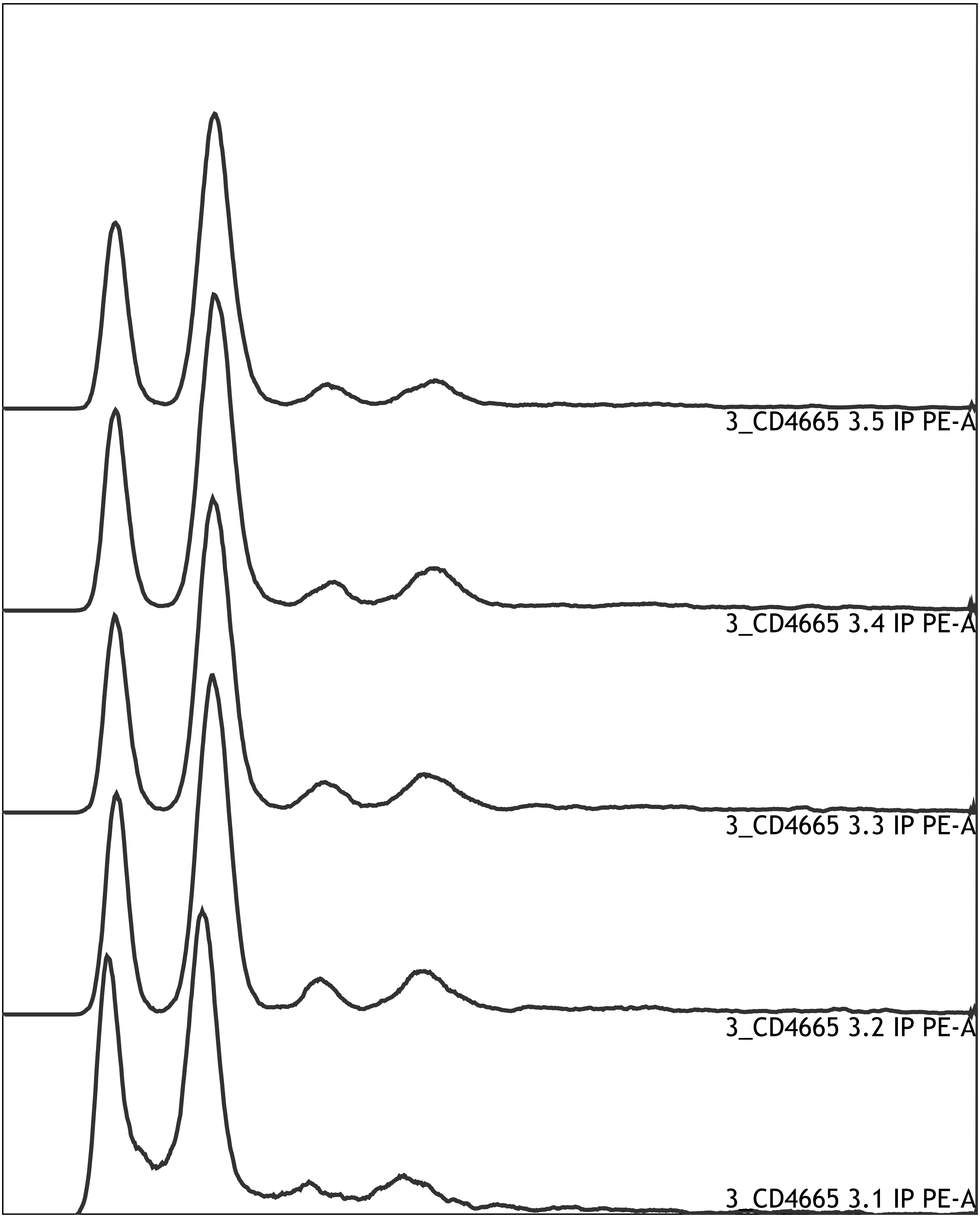
3_CD4665 3.5 IP PE-A
3_CD4665 3.4 IP PE-A
3_CD4665 3.3 IP PE-A
3_CD4665 3.2 IP PE-A
3_CD4665 3.1 IP PE-A

New Composite 5 - Plot Sheet 1

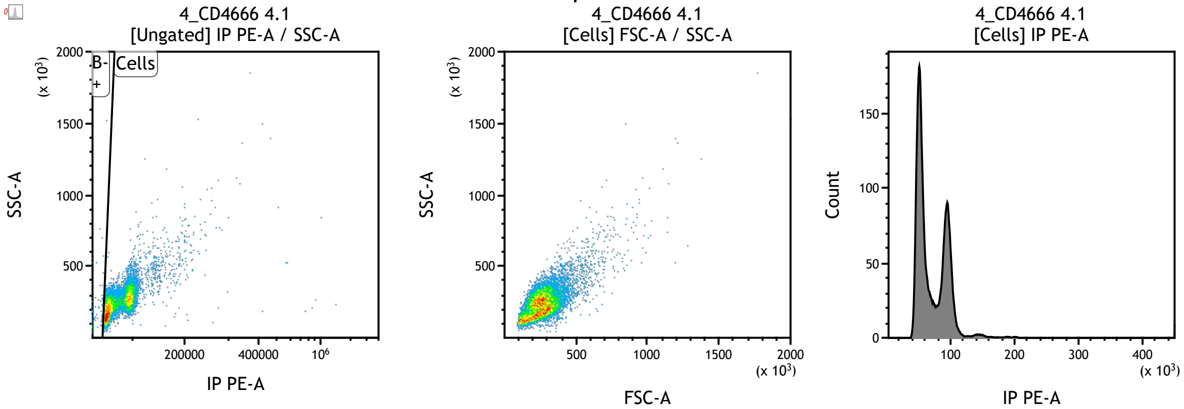

New Composite 5 - Plot Sheet 2

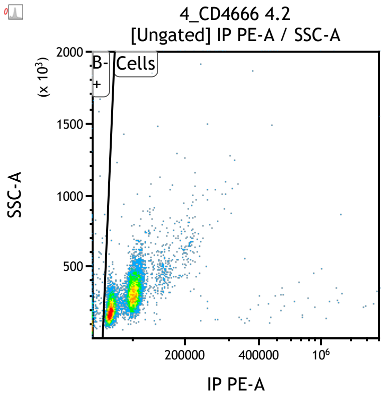

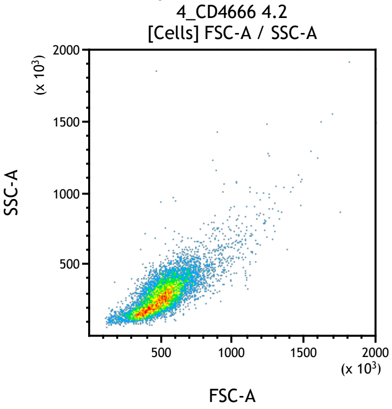

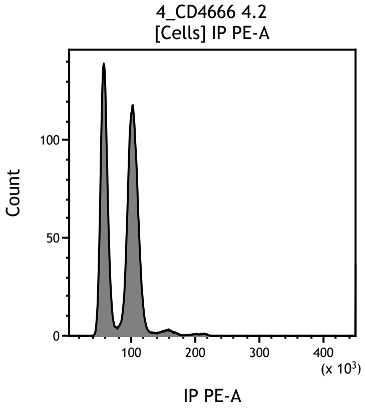

New Composite 5 - Plot Sheet 3

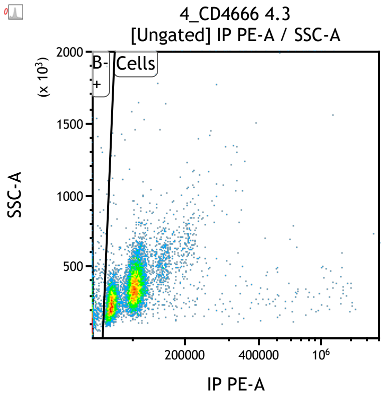

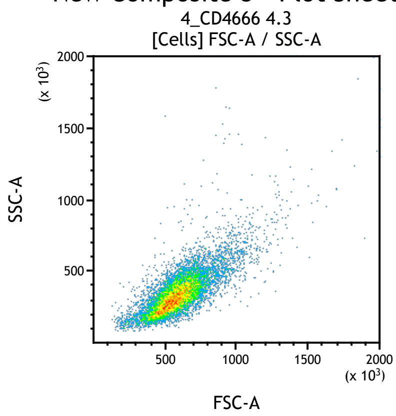

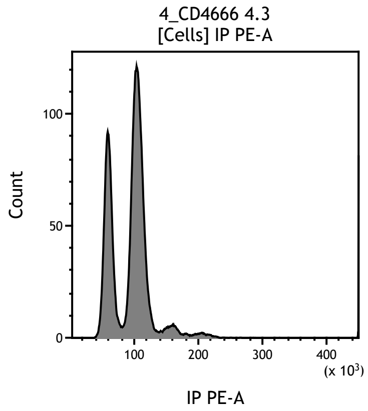

New Composite 5 - Plot Sheet 4

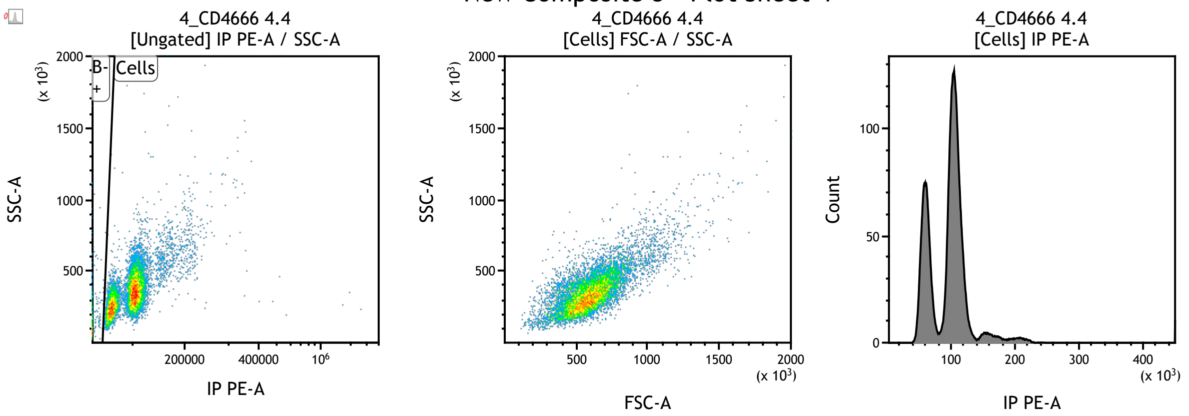

New Composite 5 - Plot Sheet 5

4_CD4666 4.5
[Ungated] IP PE-A / SSC-A

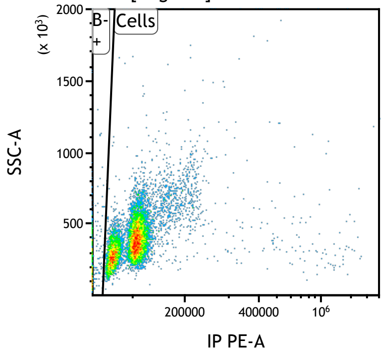

4_CD4666 4.5
[Cells] FSC-A / SSC-A

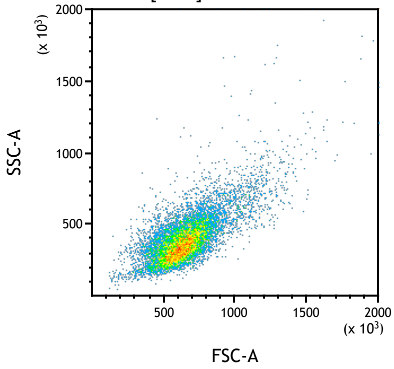

4_CD4666 4.5
[Cells] IP PE-A

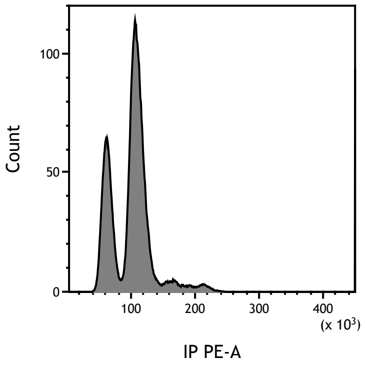

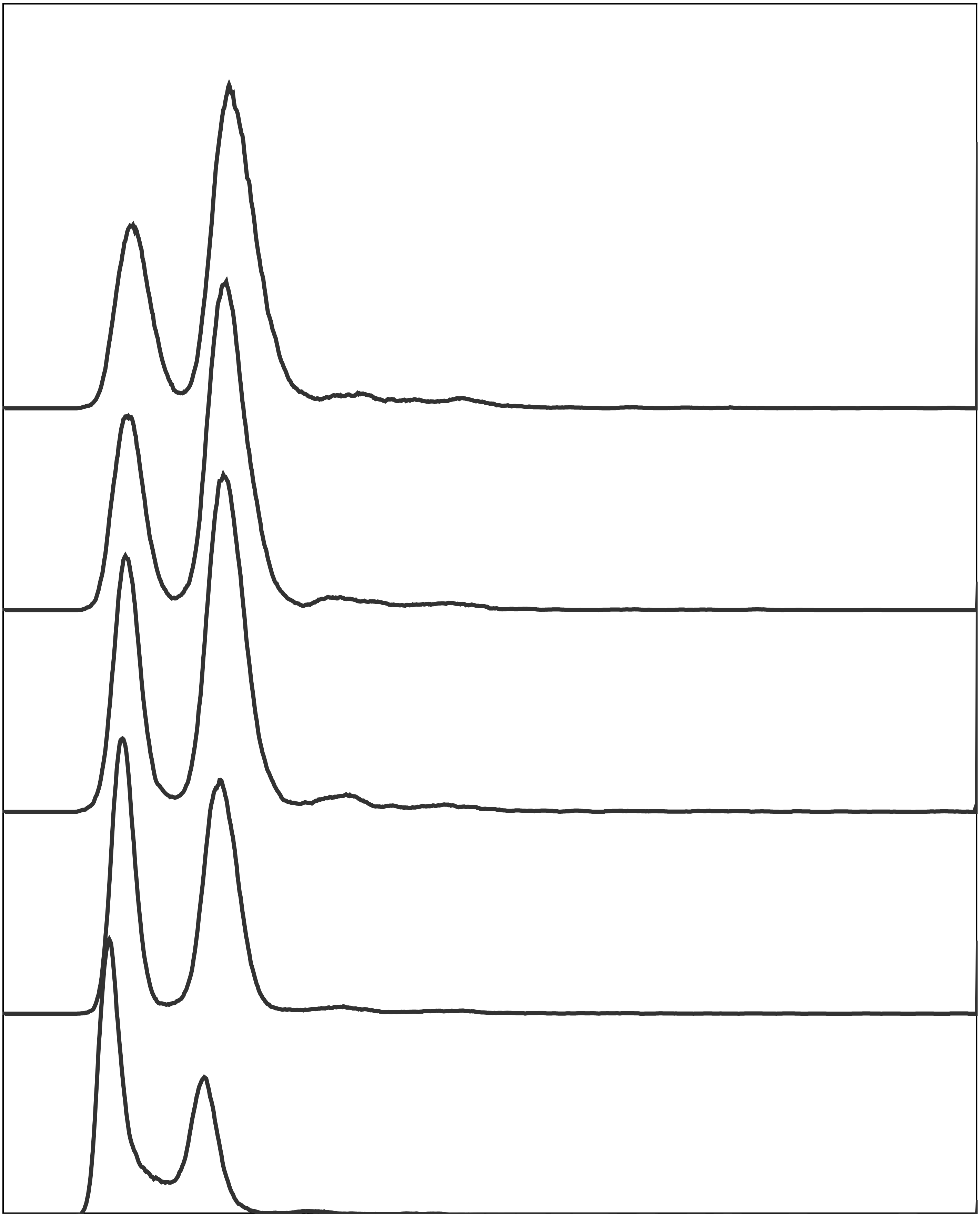

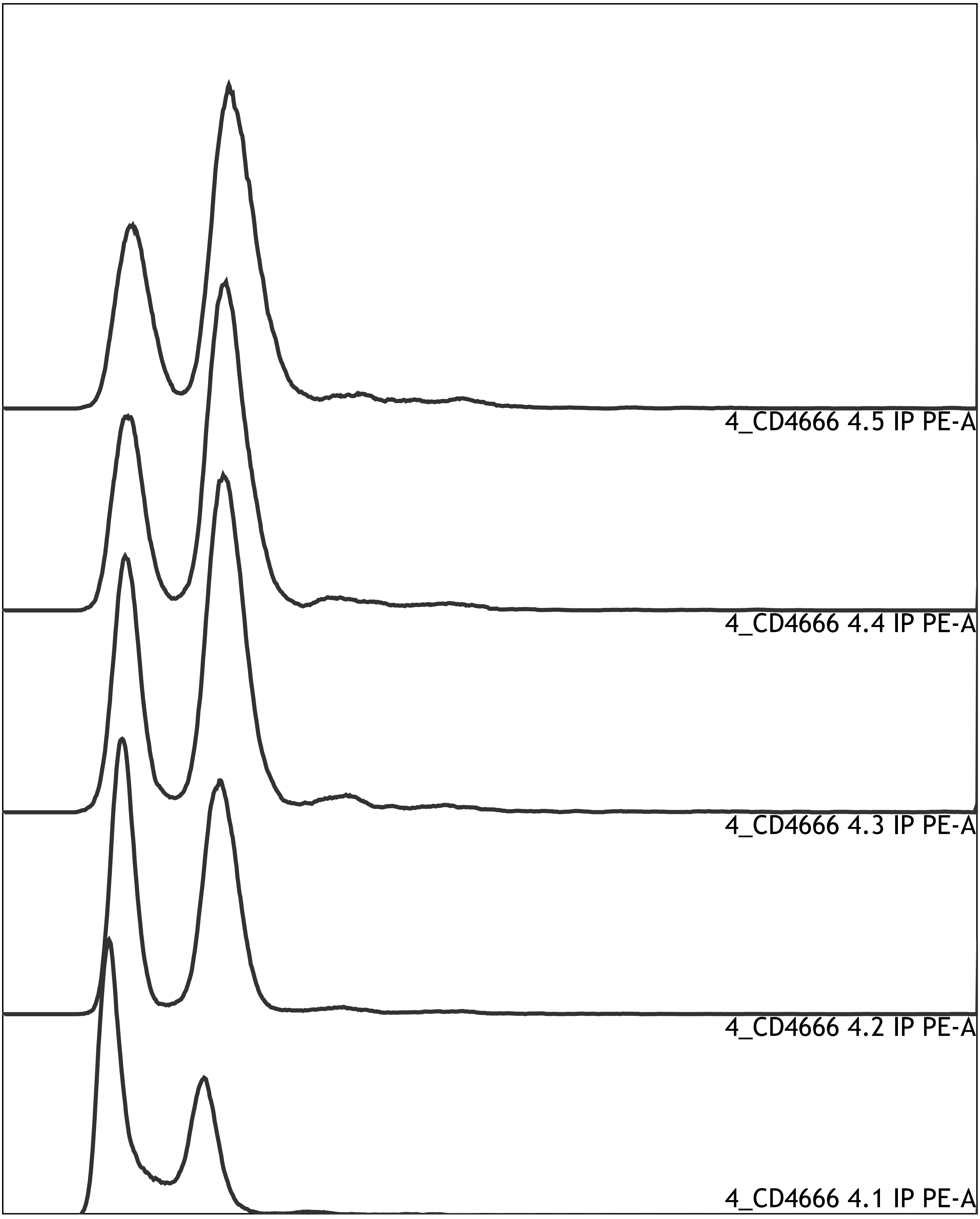
4_CD4666 4.5 IP PE-A
4_CD4666 4.4 IP PE-A
4_CD4666 4.3 IP PE-A
4_CD4666 4.2 IP PE-A
4_CD4666 4.1 IP PE-A
