# Supplementary material for: TOR complex 1 negatively regulates NDR kinase Cbk1 to control cell separation in budding yeast
Source: PLoS Biol. 2023 Aug 30;21(8):e3002263. doi: 10.1371/journal.pbio.3002263 (PMC10468069; doi:10.1371/journal.pbio.3002263)
Supplement: S1 File — (ZIP) [file pbio.3002263.s024.zip › 6B.pdf]

New Composite 7 - Plot Sheet 2

7_CD4692 7.2
[Ungated] PE-A / SSC-A

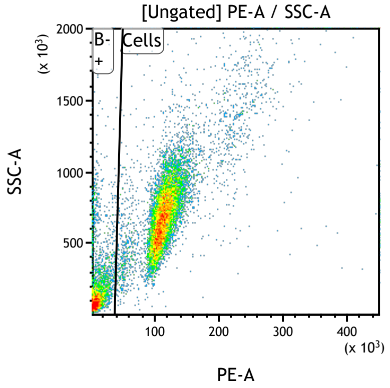

7_CD4692 7.2
[Cells] FSC-A / SSC-A

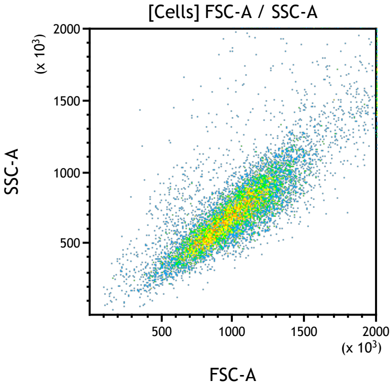

7_CD4692 7.2
[Cells] PE-A

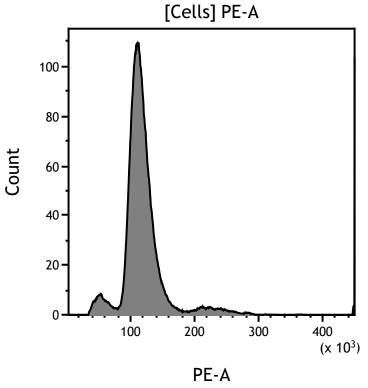

New Composite 7 - Plot Sheet 3

7_CD4692 7.3
[Ungated] PE-A / SSC-A

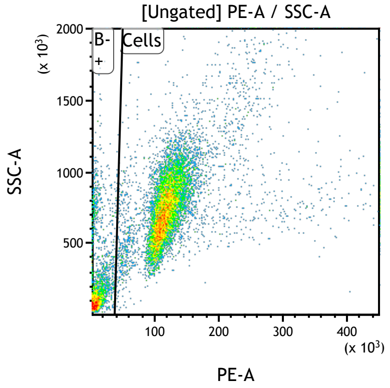

7_CD4692 7.3
[Cells] FSC-A / SSC-A

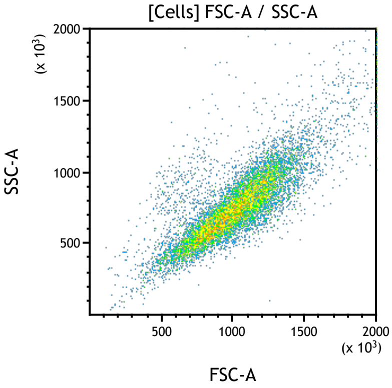

7_CD4692 7.3
[Cells] PE-A

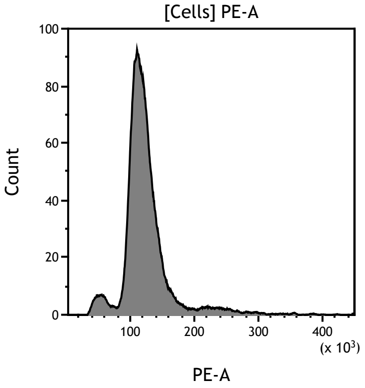

New Composite 7 - Plot Sheet 4

7_CD4692 7.4
[Ungated] PE-A / SSC-A

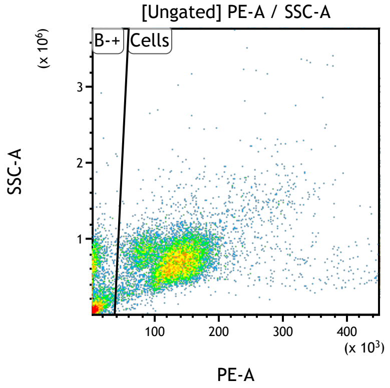

7_CD4692 7.4
[Cells] FSC-A / SSC-A

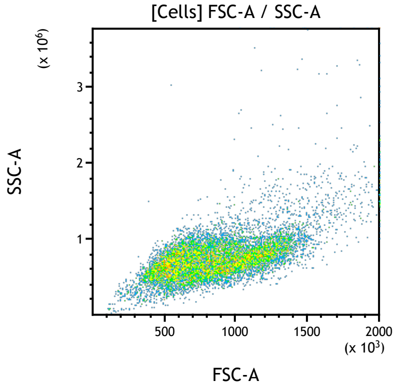

7_CD4692 7.4
[Cells] PE-A

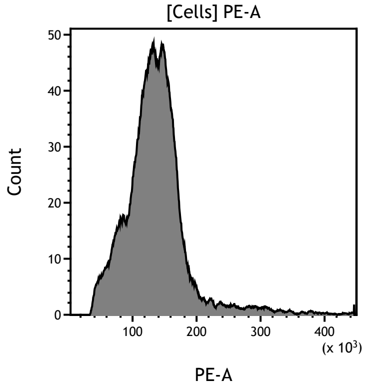

New Composite 7 - Plot Sheet 5

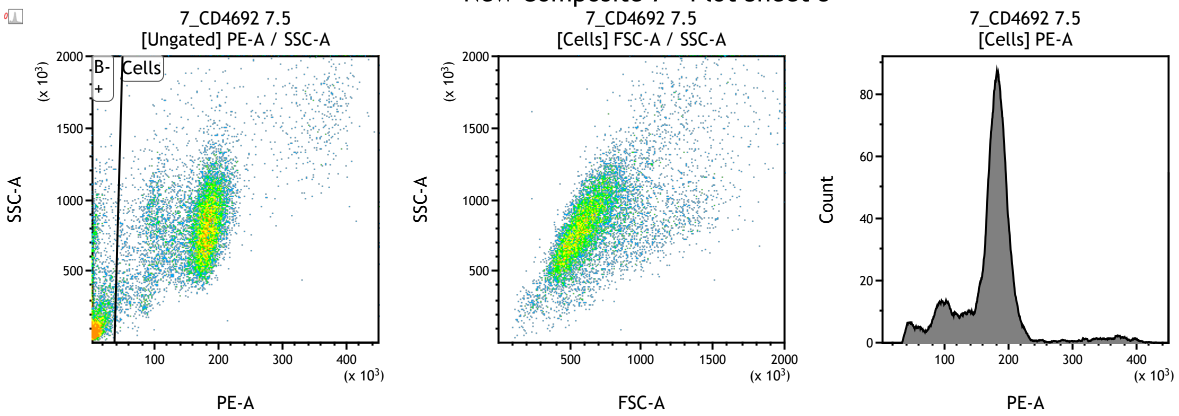

New Composite 7 - Plot Sheet 6

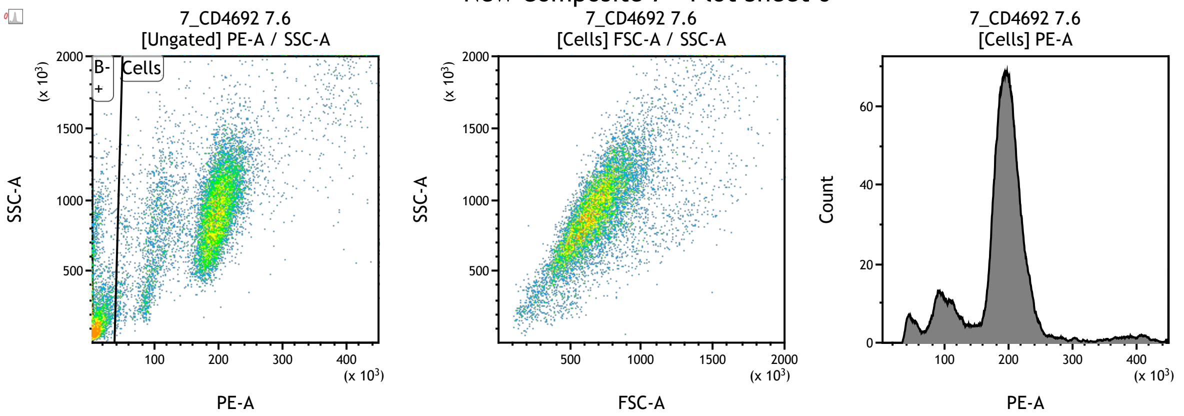

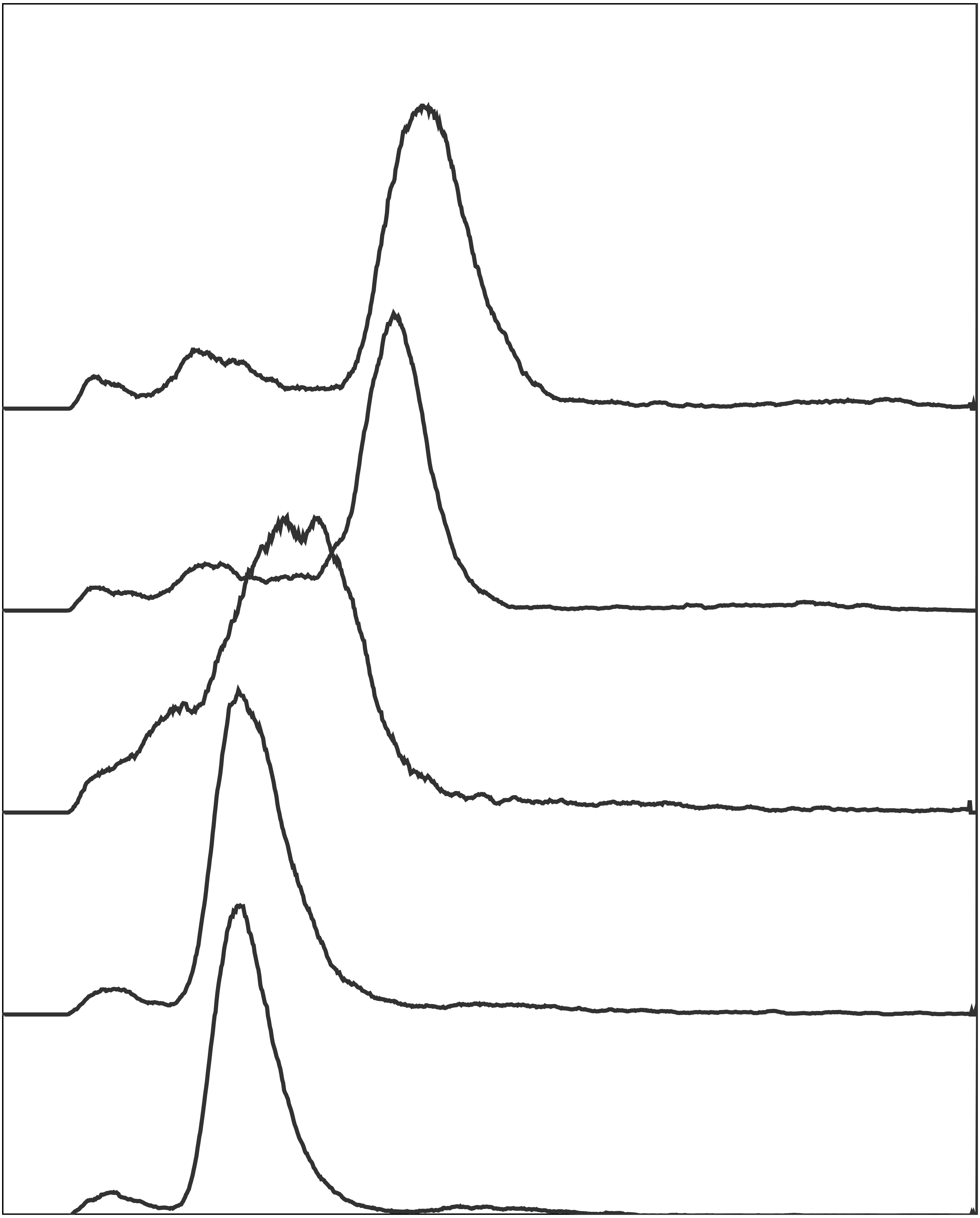

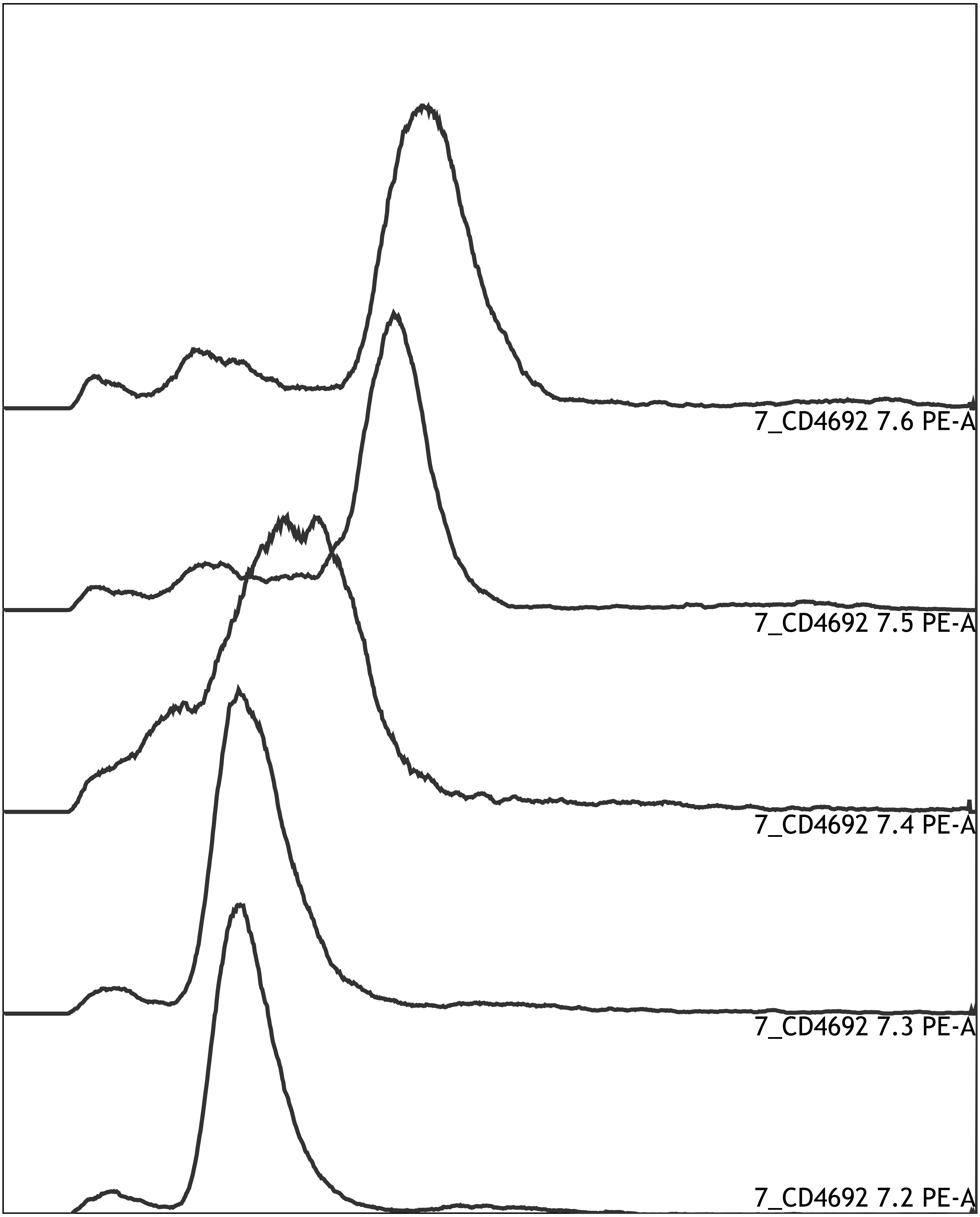
7_CD4692 7.6 PE-A
7_CD4692 7.5 PE-A
7_CD4692 7.4 PE-A
7_CD4692 7.3 PE-A
7_CD4692 7.2 PE-A

New Composite 8 - Plot Sheet 2

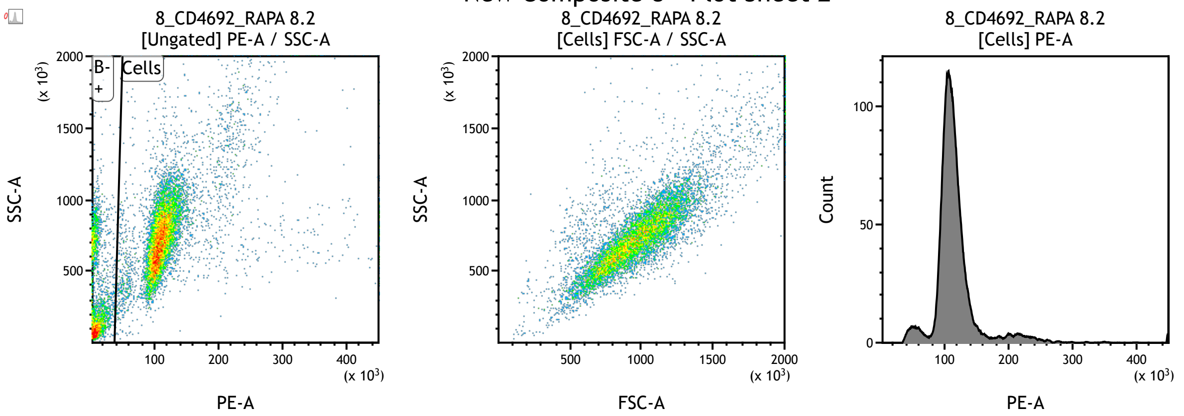

New Composite 8 - Plot Sheet 3

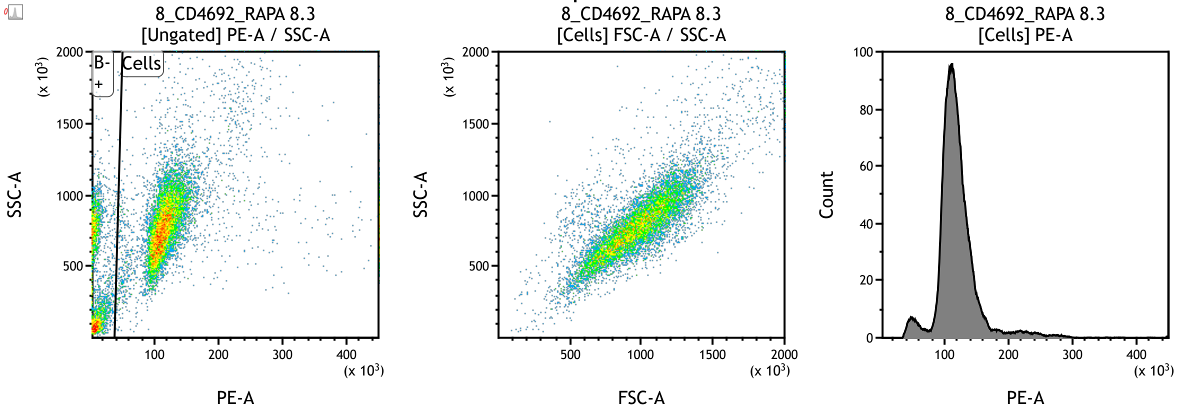

New Composite 8 - Plot Sheet 4

8_CD4692_RAPA 8.4
[Ungated] PE-A / SSC-A

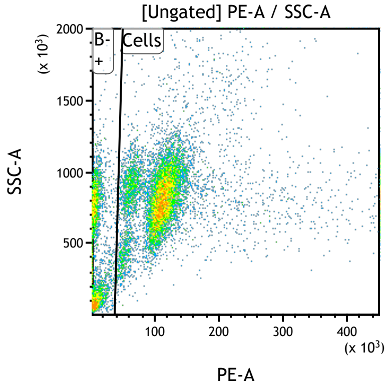

8_CD4692_RAPA 8.4
[Cells] FSC-A / SSC-A

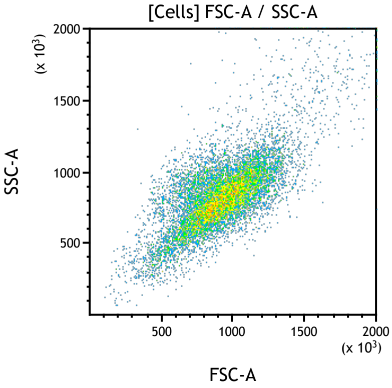

8_CD4692_RAPA 8.4
[Cells] PE-A

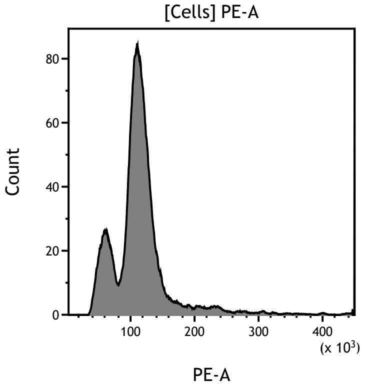

New Composite 8 - Plot Sheet 5

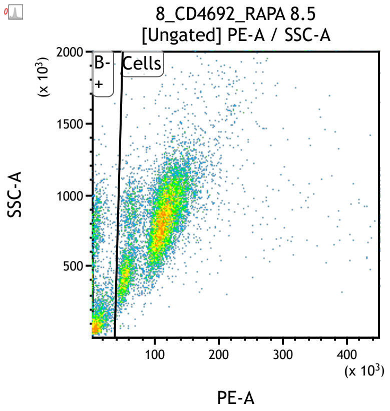

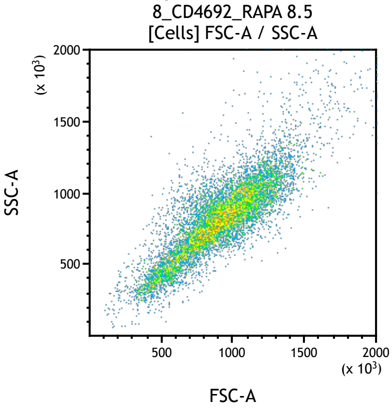

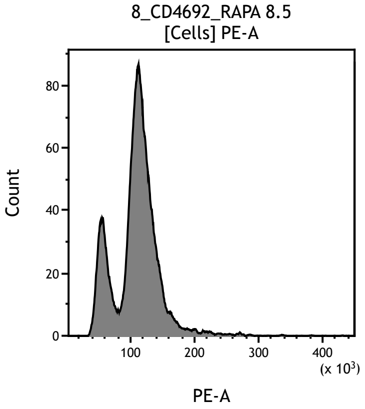

New Composite 8 - Plot Sheet 6

8_CD4692_RAPA 8.6
[Ungated] PE-A / SSC-A

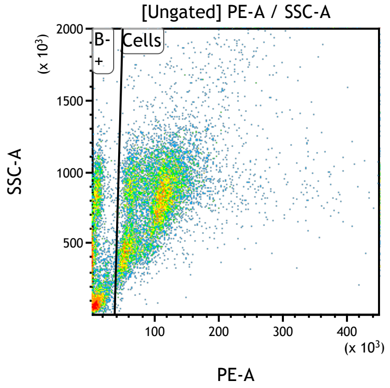

8_CD4692_RAPA 8.6
[Cells] FSC-A / SSC-A

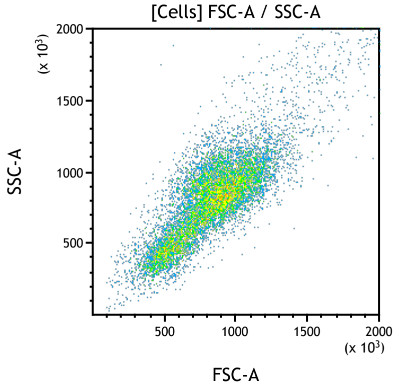

8_CD4692_RAPA 8.6
[Cells] PE-A

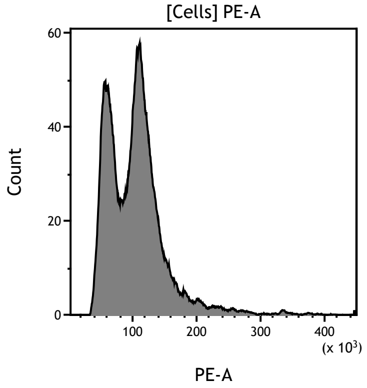

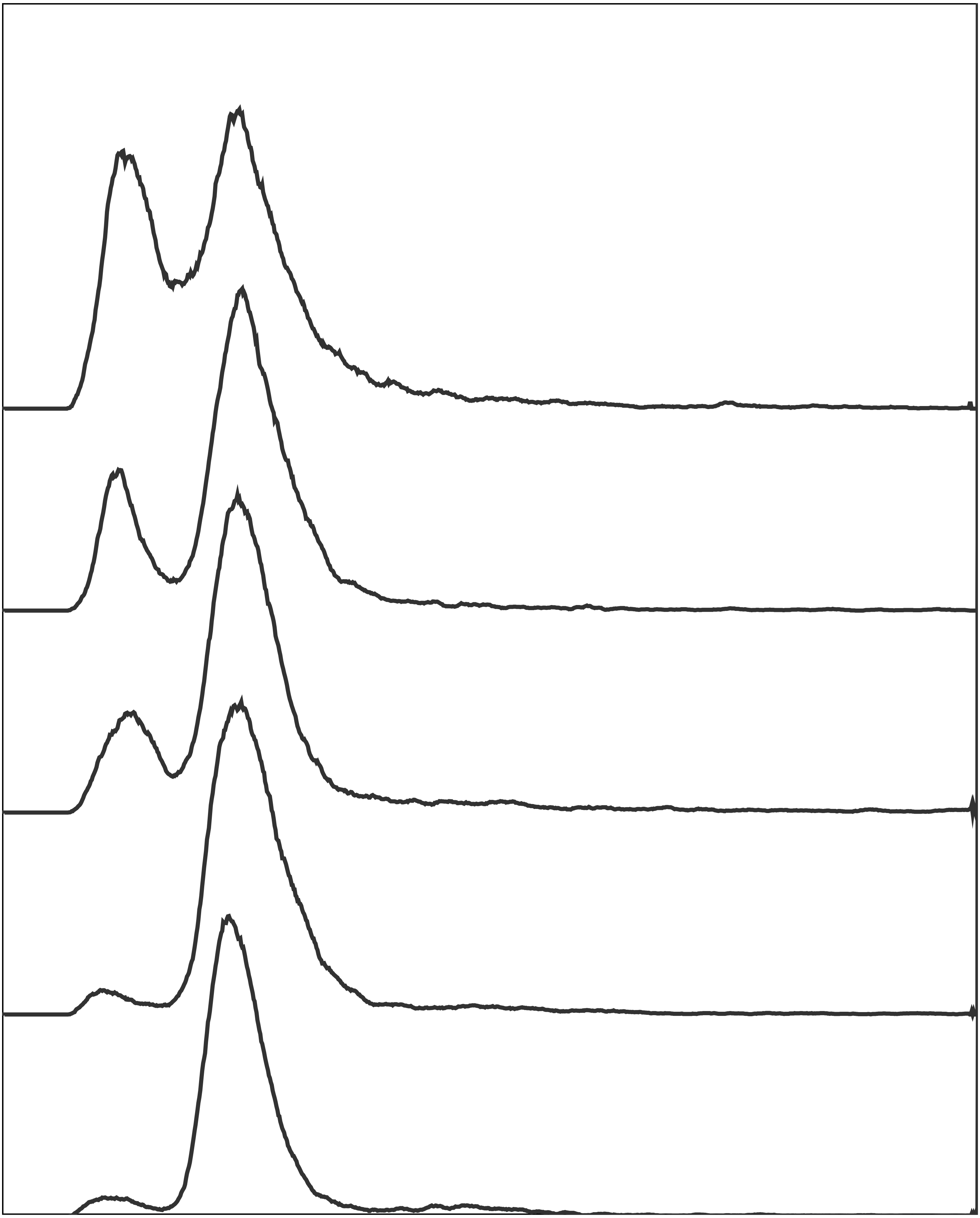

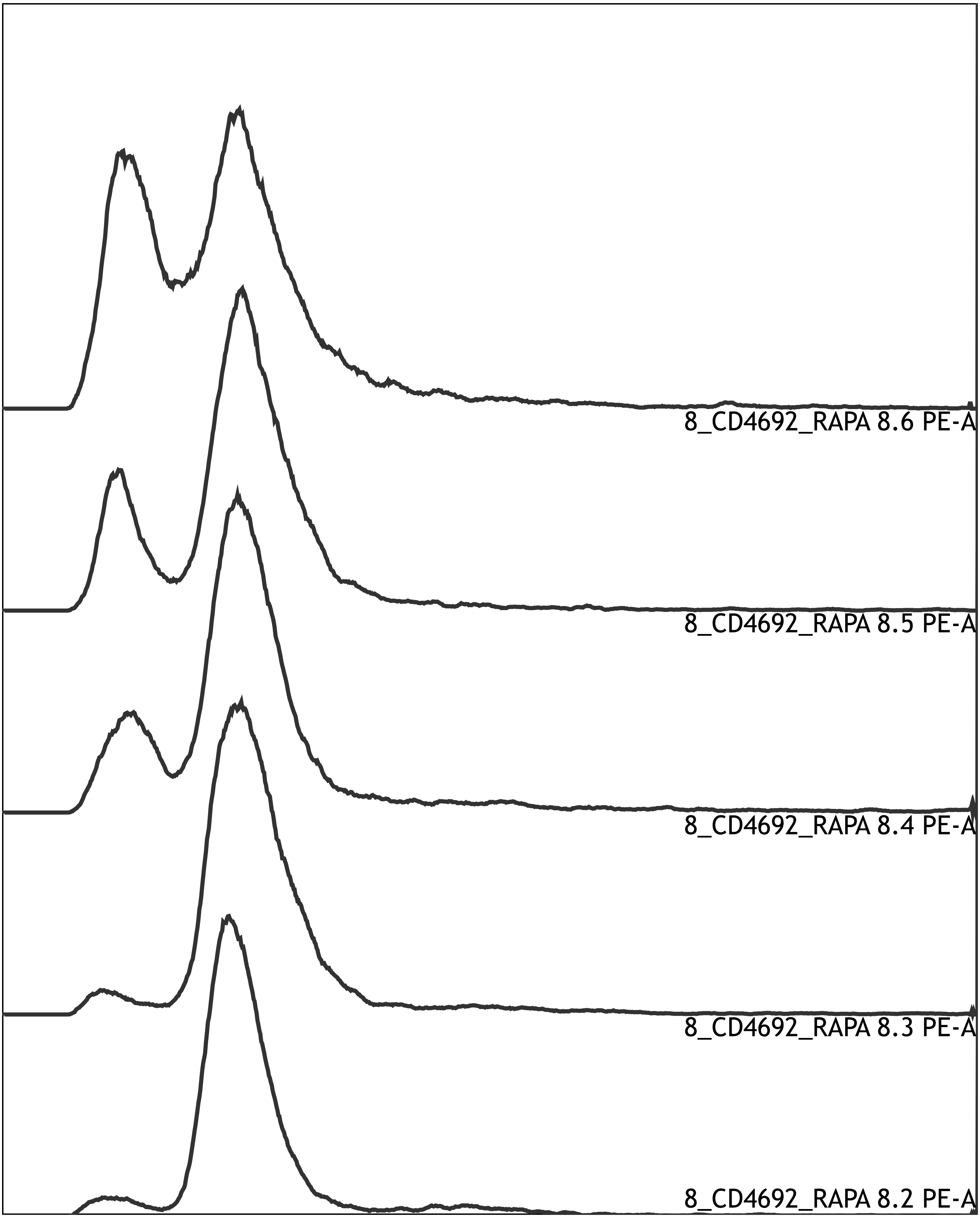
8_CD4692_RAPA 8.6 PE-A
8_CD4692_RAPA 8.5 PE-A
8_CD4692_RAPA 8.4 PE-A
8_CD4692_RAPA 8.3 PE-A
8_CD4692_RAPA 8.2 PE-A
