# Supplementary material for: TOR complex 1 negatively regulates NDR kinase Cbk1 to control cell separation in budding yeast
Source: PLoS Biol. 2023 Aug 30;21(8):e3002263. doi: 10.1371/journal.pbio.3002263 (PMC10468069; doi:10.1371/journal.pbio.3002263)
Supplement: S1 File — (ZIP) [file pbio.3002263.s024.zip › S1C.pdf]

New Composite 11 - Plot Sheet 2

10_CD169_37_RAPA 10.2
[Ungated] PE-A / SSC-A

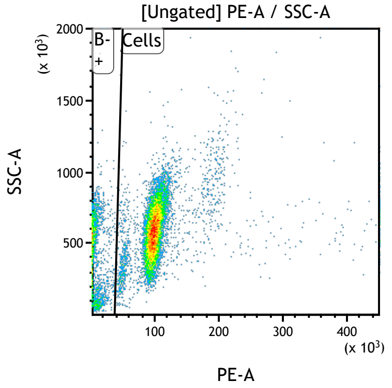

10_CD169_37_RAPA 10.2
[Cells] FSC-A / SSC-A

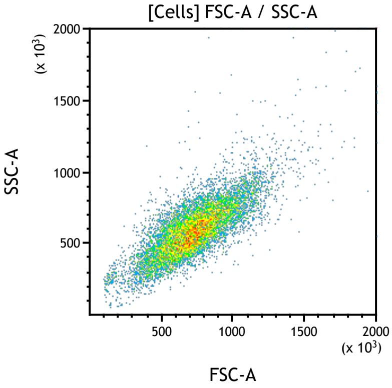

10_CD169_37_RAPA 10.2
[Cells] PE-A

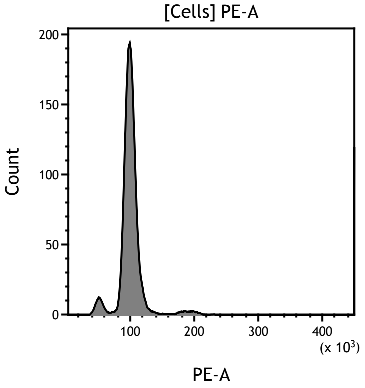

New Composite 11 - Plot Sheet 3

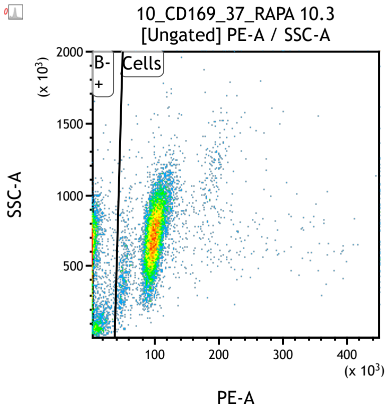

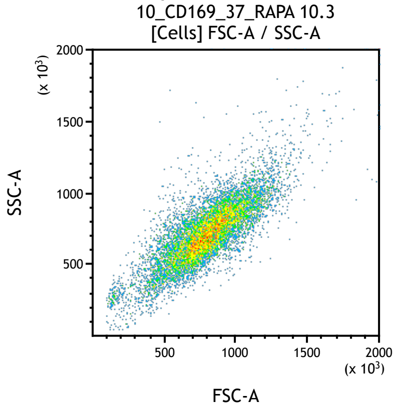

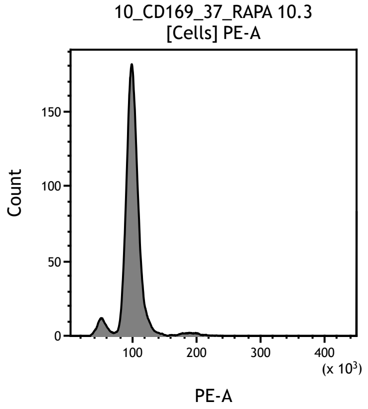

New Composite 11 - Plot Sheet 4

10_CD169_37_RAPA 10.4
[Ungated] PE-A / SSC-A

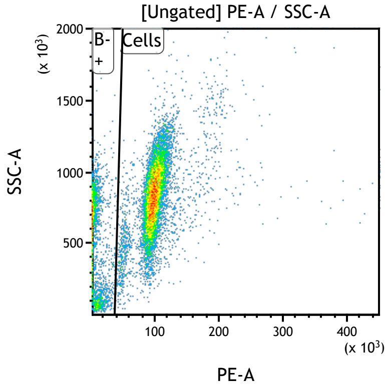

10_CD169_37_RAPA 10.4
[Cells] FSC-A / SSC-A

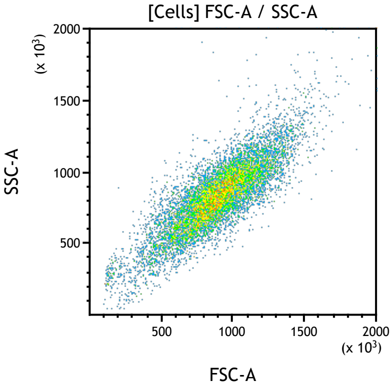

10_CD169_37_RAPA 10.4
[Cells] PE-A

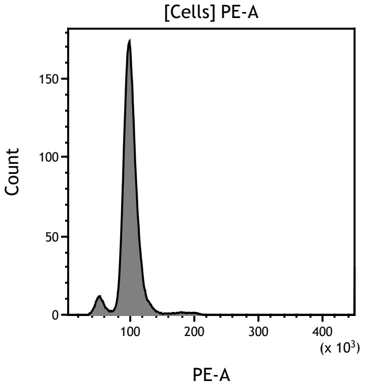

New Composite 11 - Plot Sheet 5

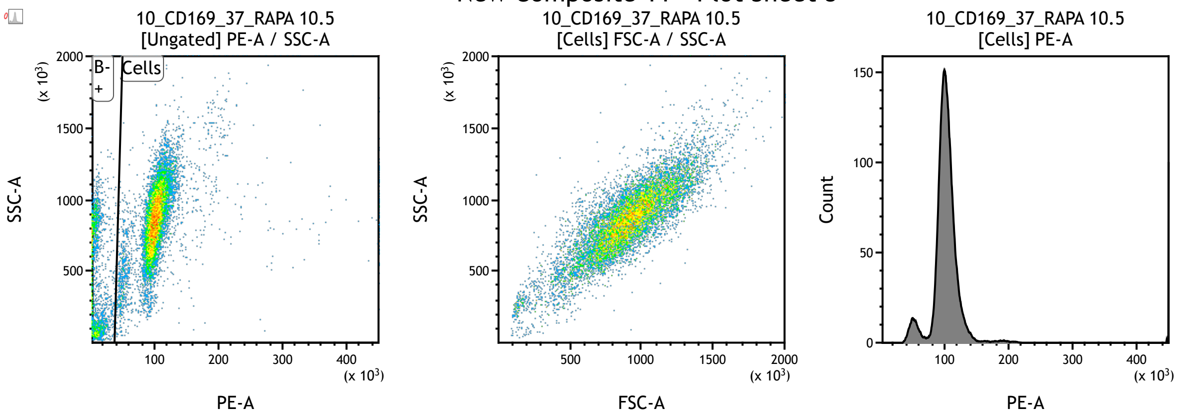

New Composite 11 - Plot Sheet 6

10_CD169_37_RAPA 10.6
[Ungated] PE-A / SSC-A

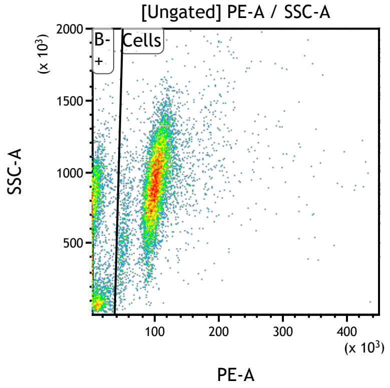

10_CD169_37_RAPA 10.6
[Cells] FSC-A / SSC-A

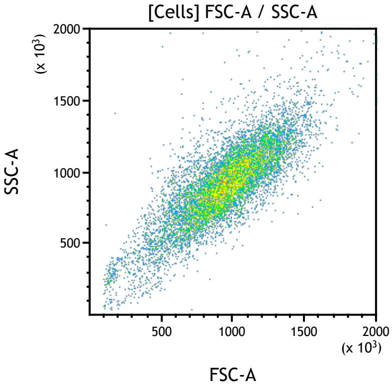

10_CD169_37_RAPA 10.6
[Cells] PE-A

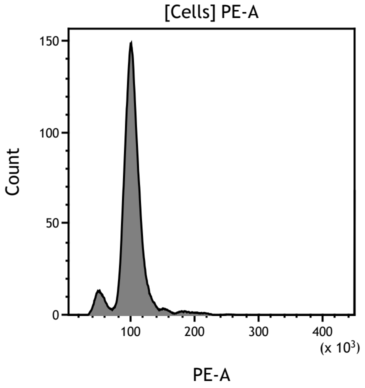

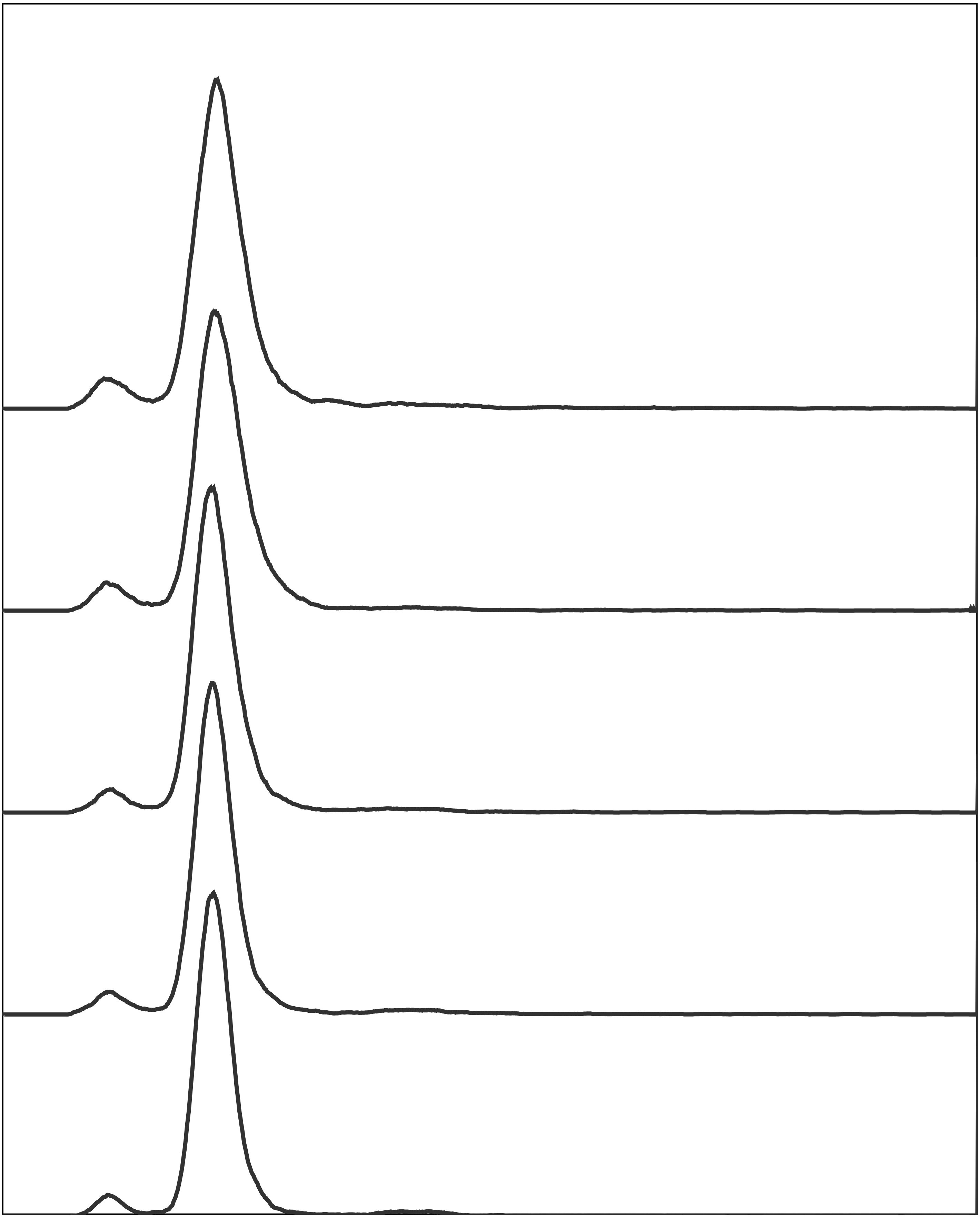

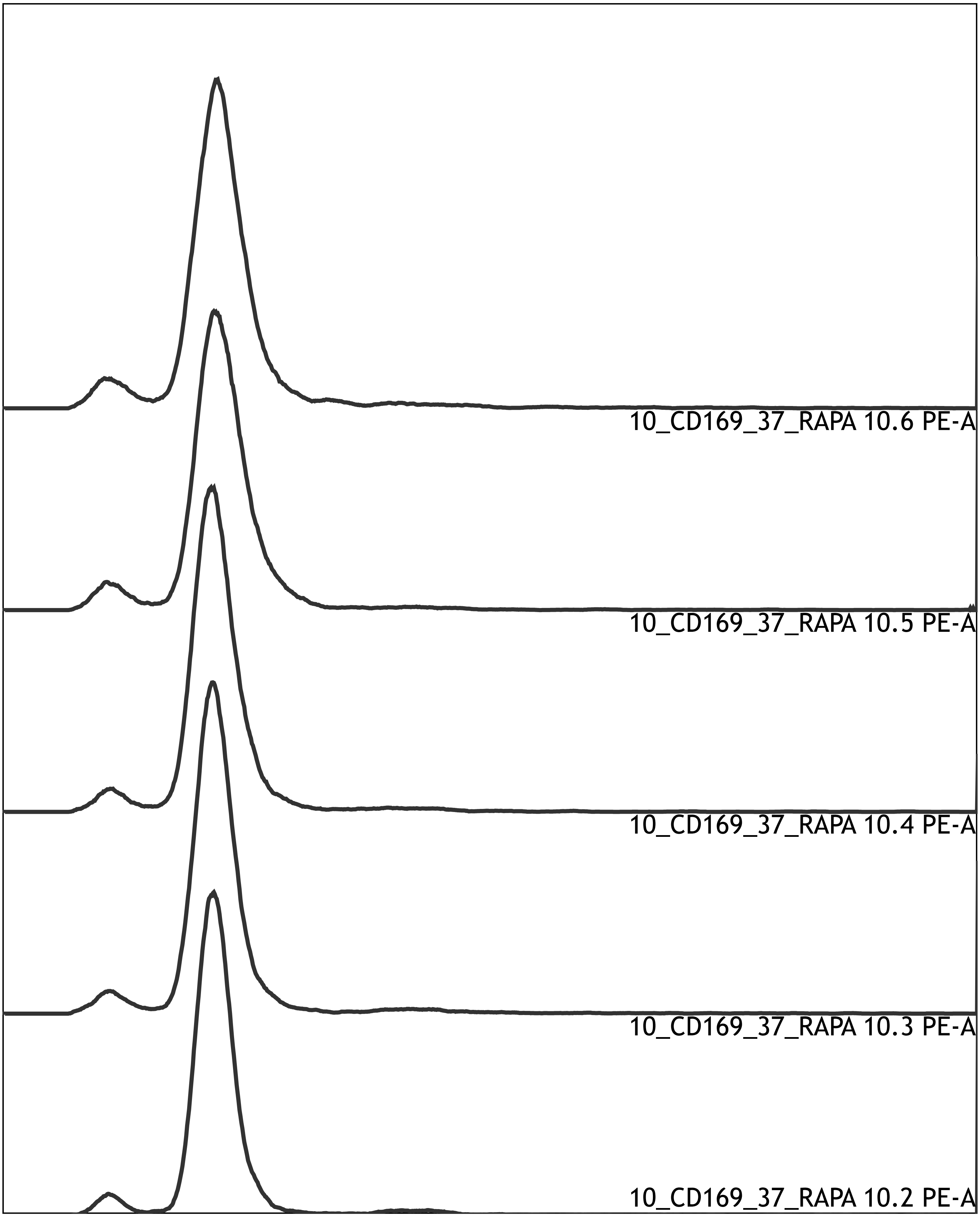
10_CD169_37_RAPA 10.6 PE-A
10_CD169_37_RAPA 10.5 PE-A
10_CD169_37_RAPA 10.4 PE-A
10_CD169_37_RAPA 10.3 PE-A
10_CD169_37_RAPA 10.2 PE-A
