# Supplementary material for: TOR complex 1 negatively regulates NDR kinase Cbk1 to control cell separation in budding yeast
Source: PLoS Biol. 2023 Aug 30;21(8):e3002263. doi: 10.1371/journal.pbio.3002263 (PMC10468069; doi:10.1371/journal.pbio.3002263)
Supplement: S1 File — (ZIP) [file pbio.3002263.s024.zip › S1D.pdf]

New Composite 8 - Plot Sheet 2

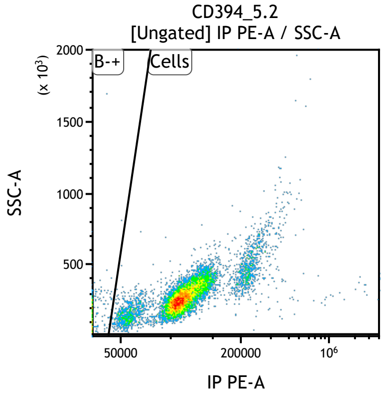

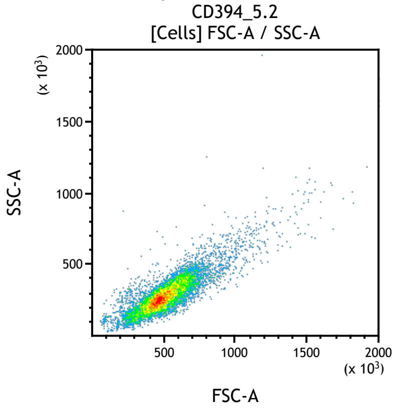

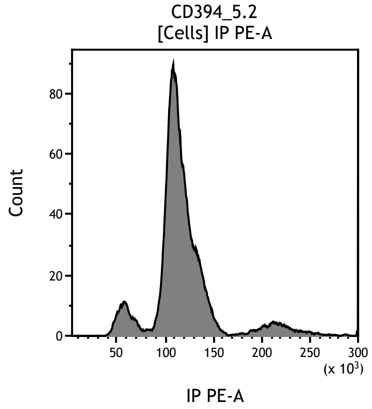

New Composite 8 - Plot Sheet 3

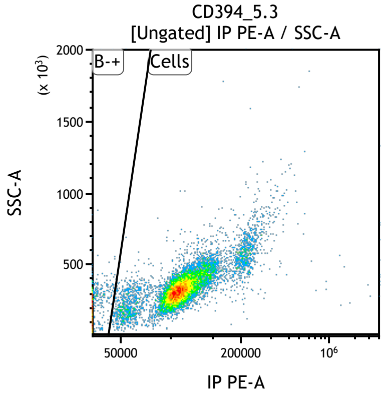

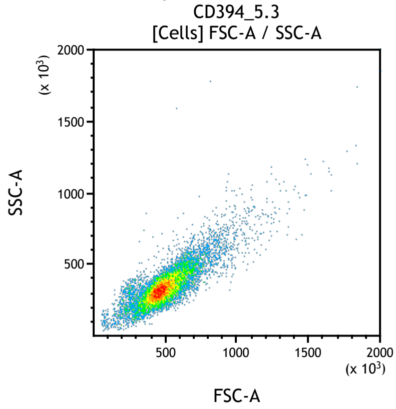

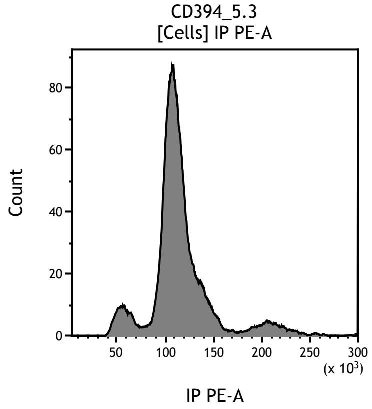

New Composite 8 - Plot Sheet 4

CD394_5.4
[Ungated] IP PE-A / SSC-A

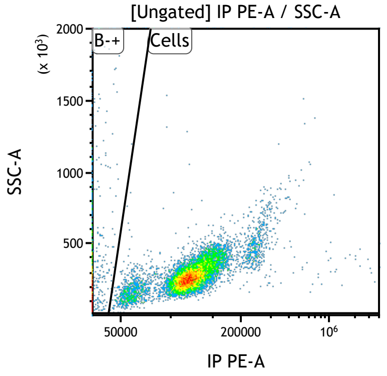

CD394_5.4
[Cells] FSC-A / SSC-A

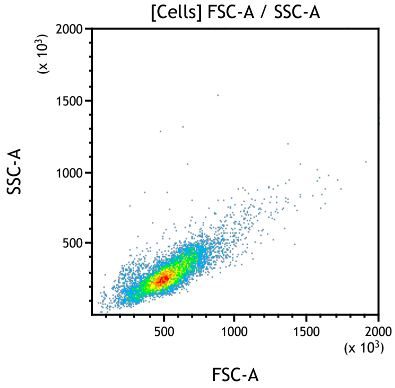

CD394_5.4
[Cells] IP PE-A

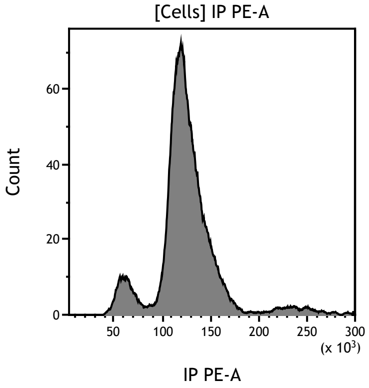

New Composite 8 - Plot Sheet 5

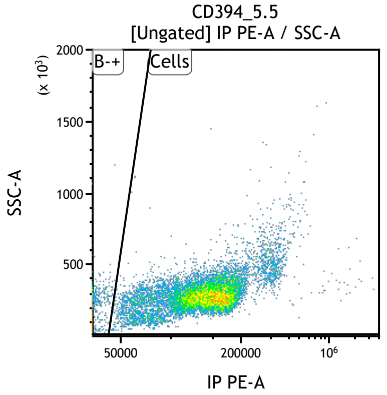

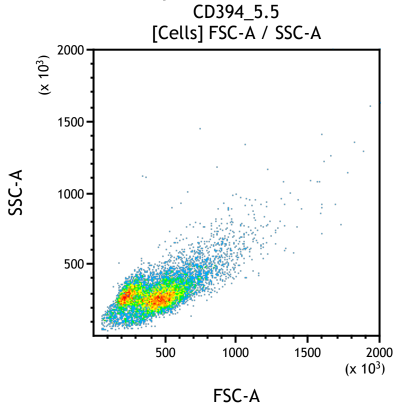

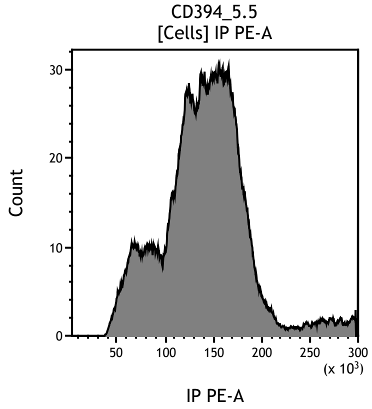

New Composite 8 - Plot Sheet 6

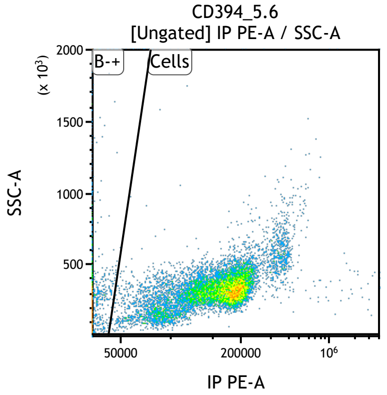

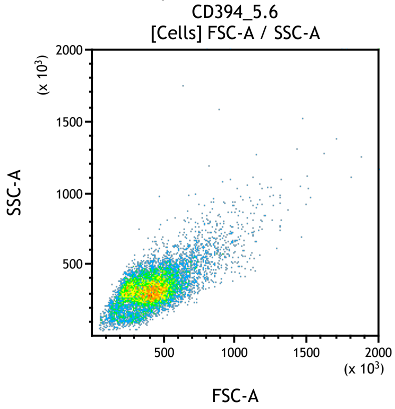

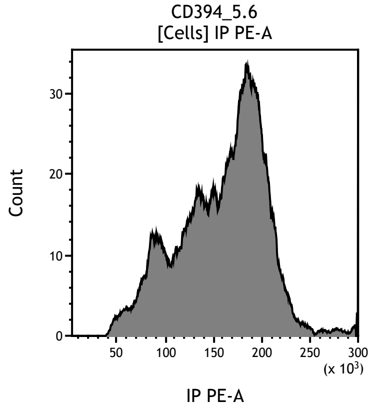

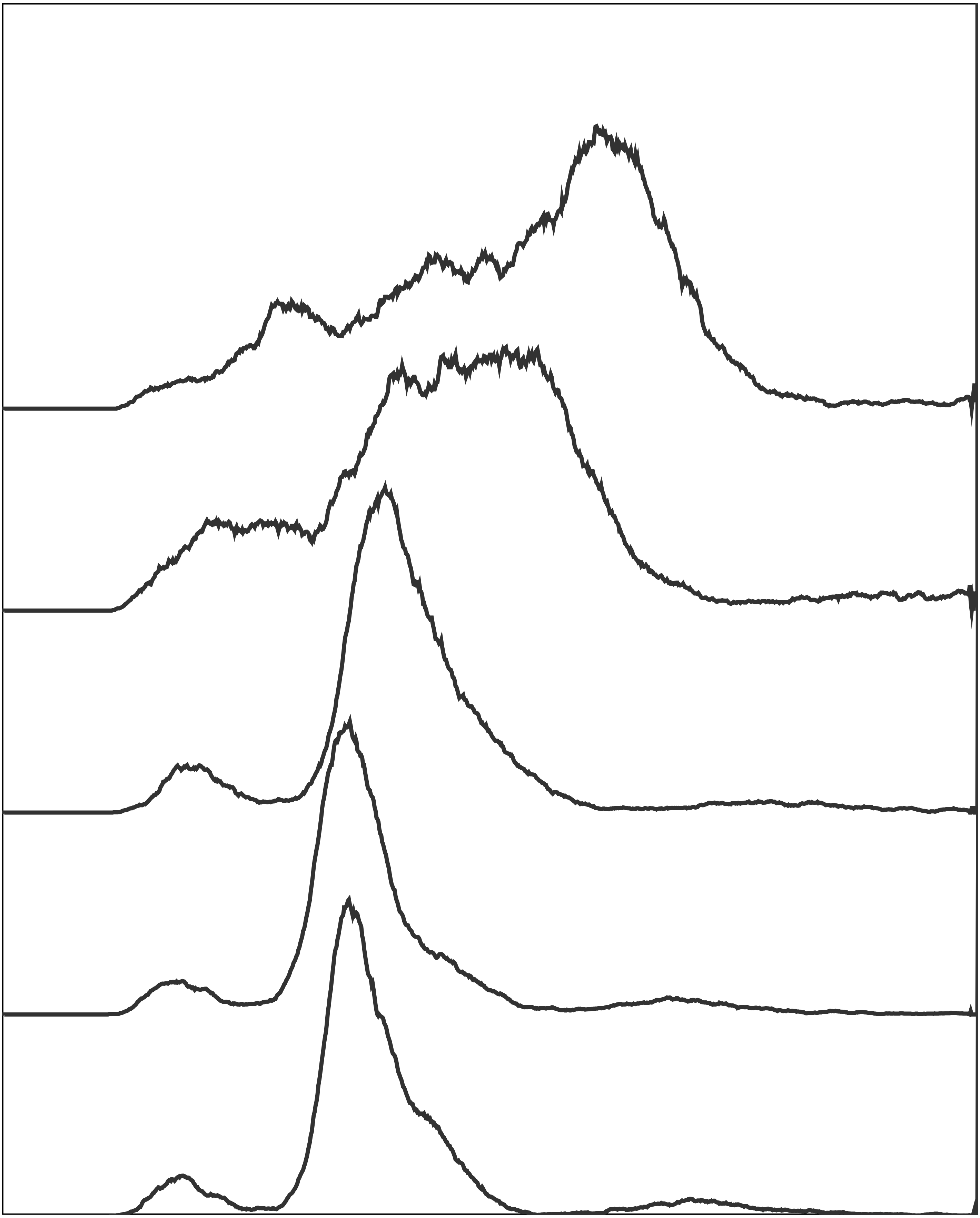

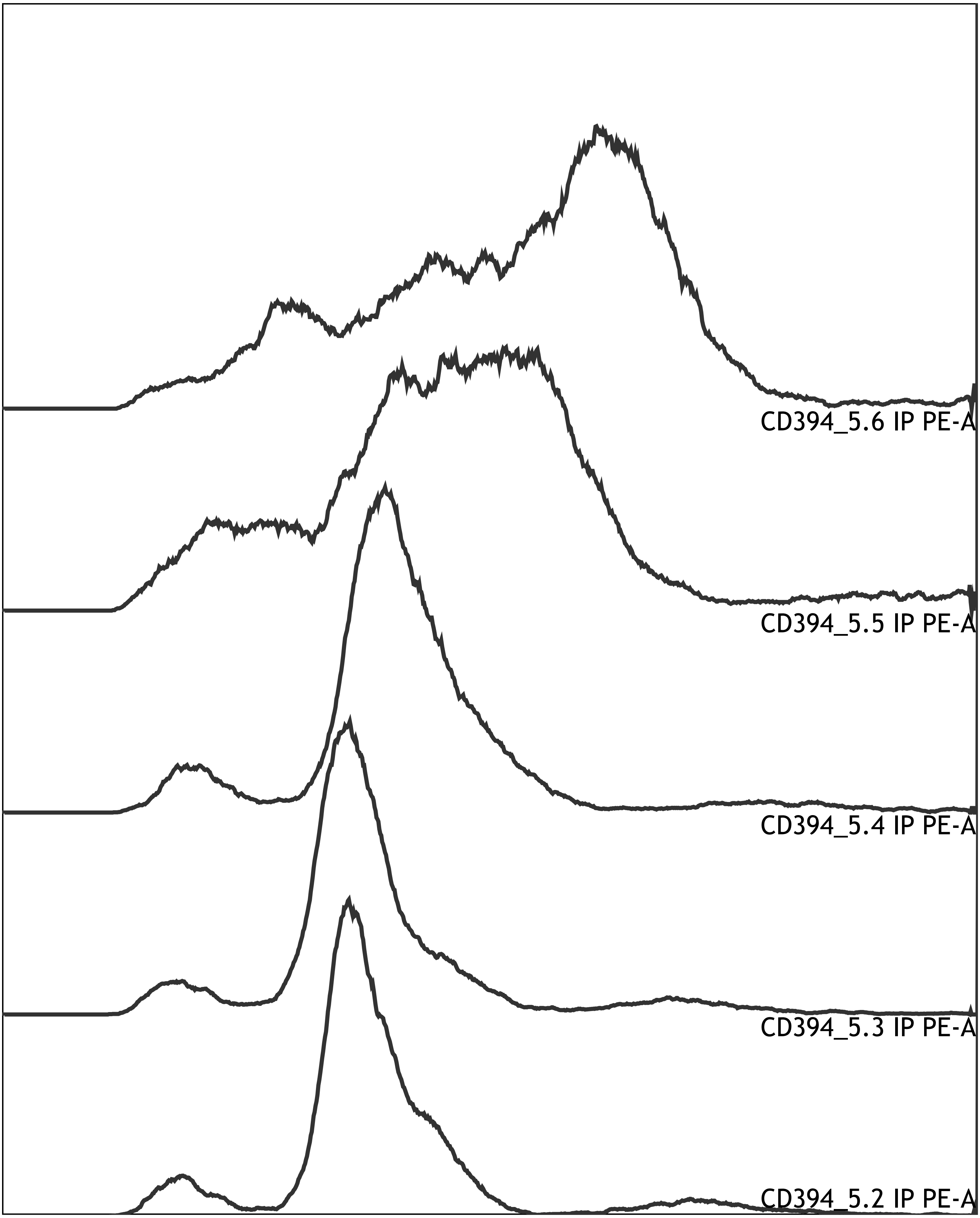
CD394_5.6 IP PE-A
CD394_5.5 IP PE-A
CD394_5.4 IP PE-A
CD394_5.3 IP PE-A
CD394_5.2 IP PE-A

New Composite 7 - Plot Sheet 2

CD394 RAPA_6.2
[Ungated] IP PE-A / SSC-A

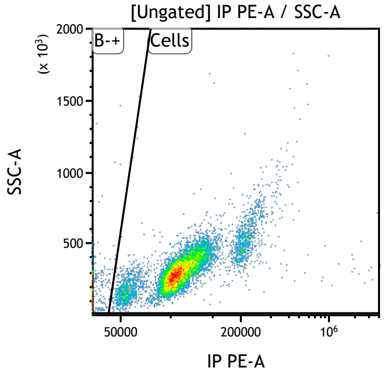

CD394 RAPA_6.2
[Cells] FSC-A / SSC-A

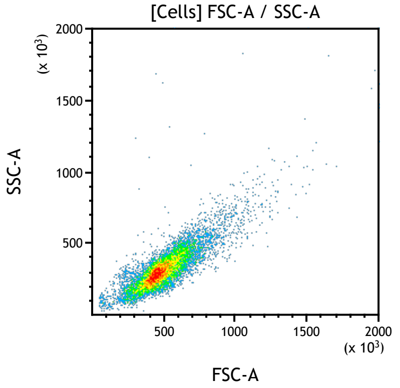

CD394 RAPA_6.2
[Cells] IP PE-A

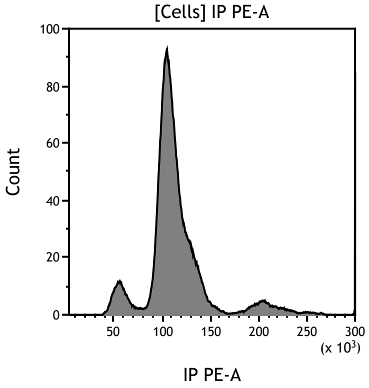

New Composite 7 - Plot Sheet 3

CD394 RAPA_6.3
[Ungated] IP PE-A / SSC-A

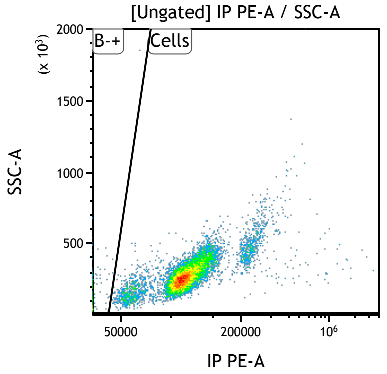

CD394 RAPA_6.3
[Cells] FSC-A / SSC-A

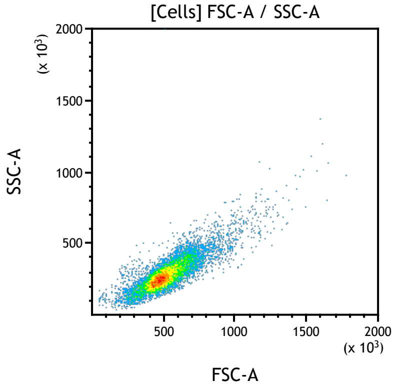

CD394 RAPA_6.3
[Cells] IP PE-A

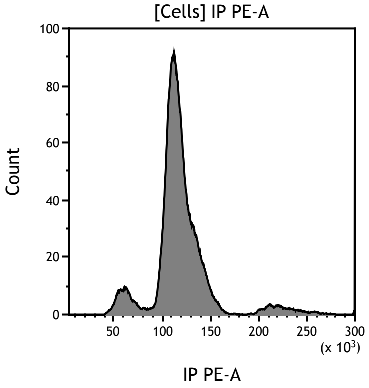

New Composite 7 - Plot Sheet 4

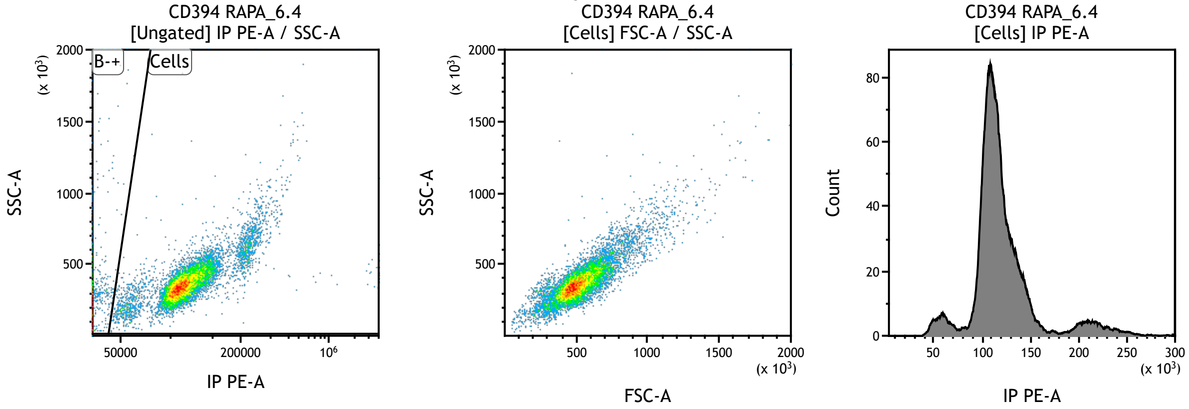

New Composite 7 - Plot Sheet 5

CD394 RAPA_6.5
[Ungated] IP PE-A / SSC-A

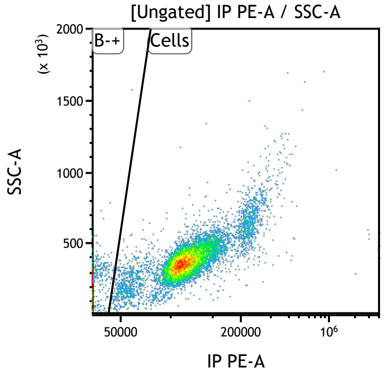

CD394 RAPA_6.5
[Cells] FSC-A / SSC-A

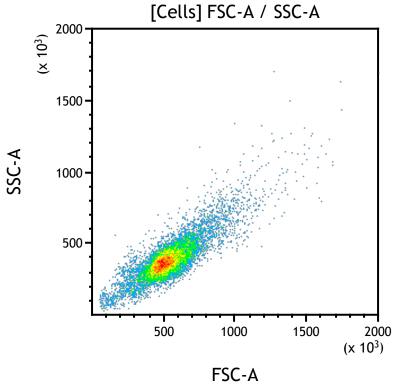

CD394 RAPA_6.5
[Cells] IP PE-A

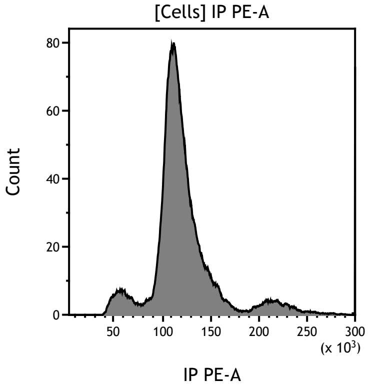

New Composite 7 - Plot Sheet 6

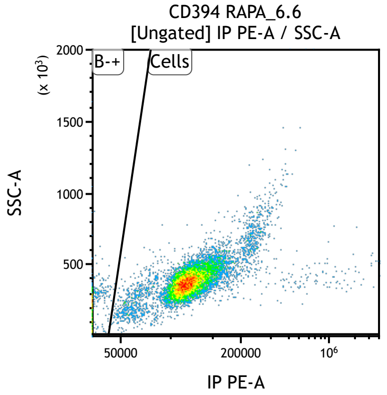

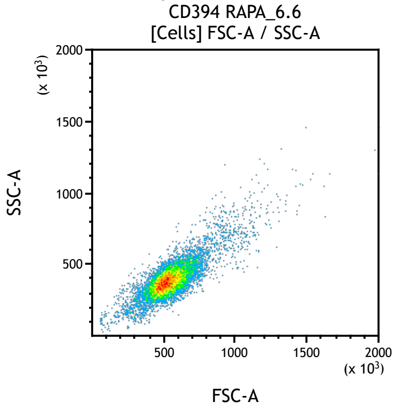

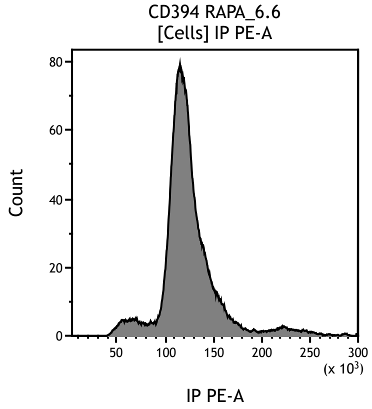

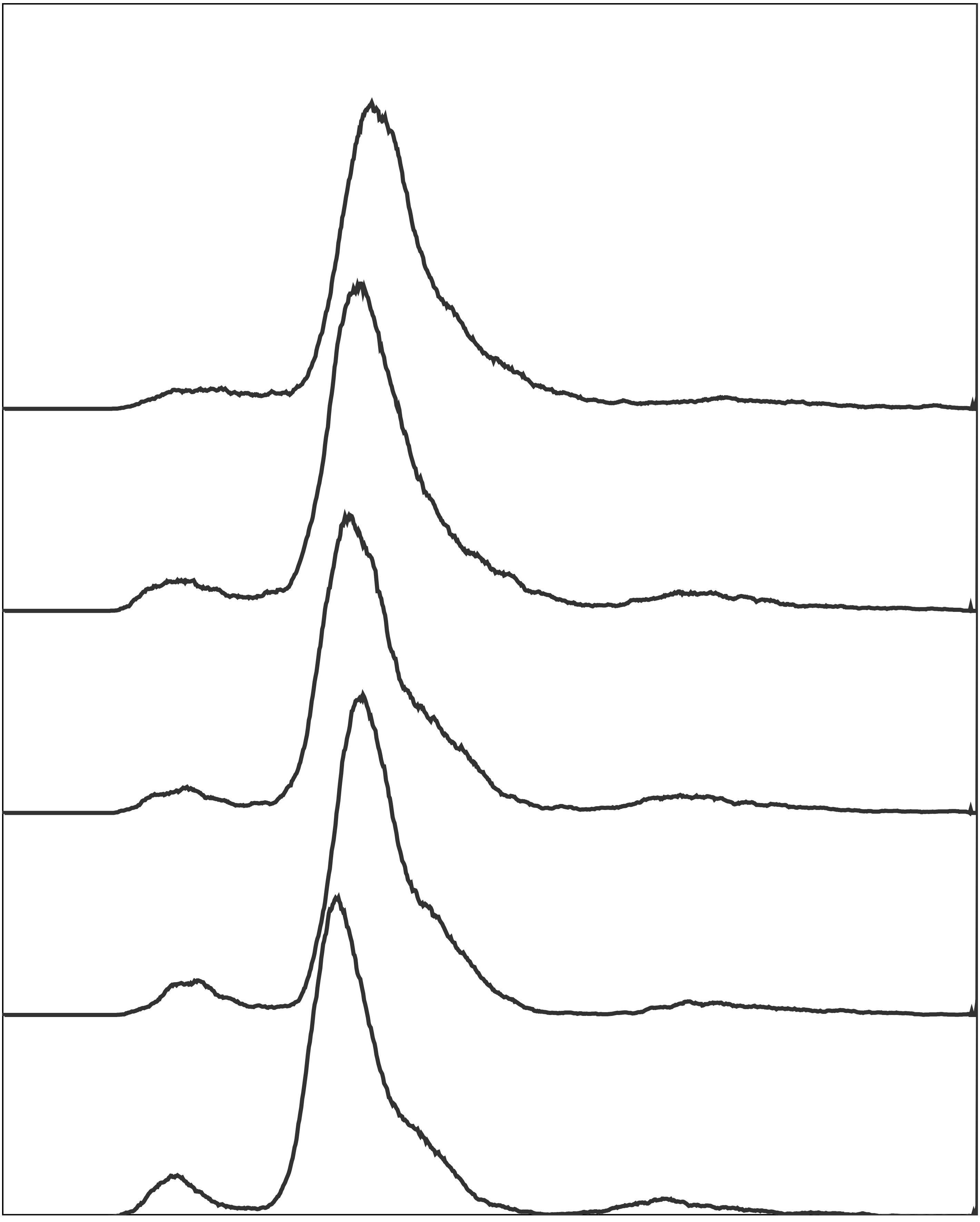

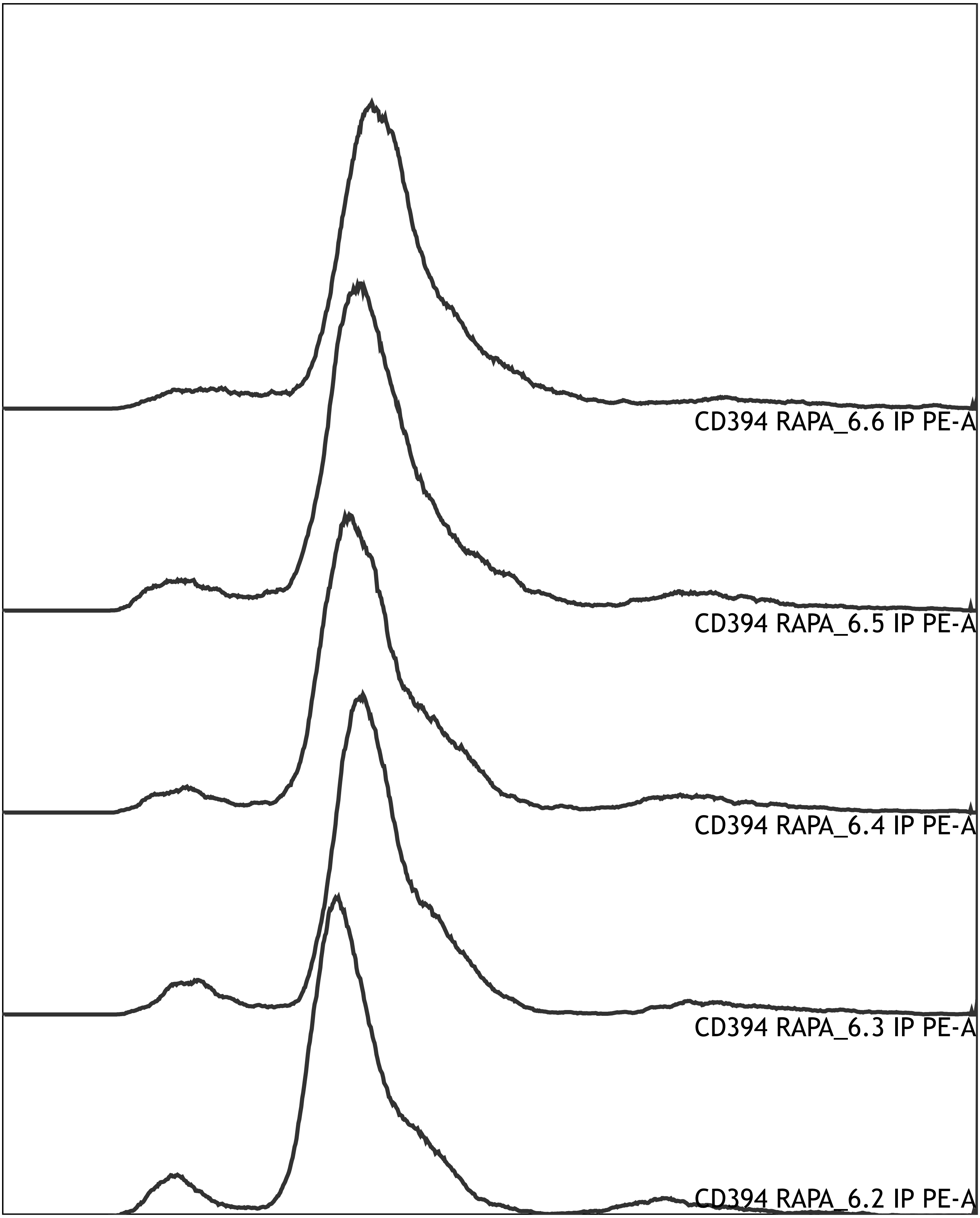

CD394 RAPA_6.6 IP PE-A
CD394 RAPA_6.5 IP PE-A
CD394 RAPA_6.4 IP PE-A
CD394 RAPA_6.3 IP PE-A
CD394 RAPA_6.2 IP PE-A
