# Supplementary material for: TOR complex 1 negatively regulates NDR kinase Cbk1 to control cell separation in budding yeast
Source: PLoS Biol. 2023 Aug 30;21(8):e3002263. doi: 10.1371/journal.pbio.3002263 (PMC10468069; doi:10.1371/journal.pbio.3002263)
Supplement: S1 File — (ZIP) [file pbio.3002263.s024.zip › S2B.pdf]

New Composite 8 - Plot Sheet 1

7_CD430_7.1
[Ungated] IP PE-A / SSC-A

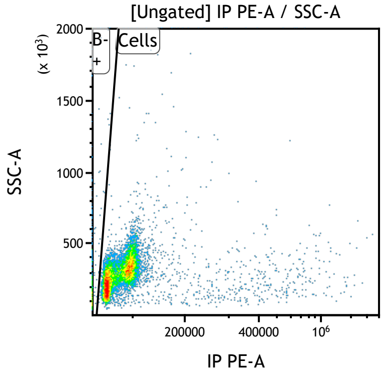

7_CD430_7.1
[Cells] FSC-A / SSC-A

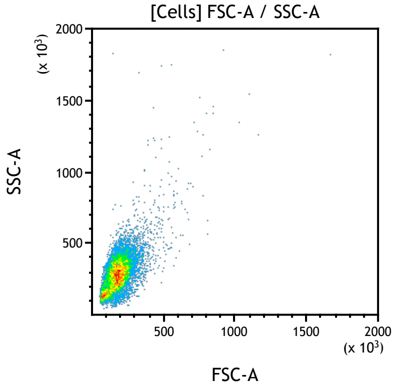

7_CD430_7.1
[Cells] IP PE-A

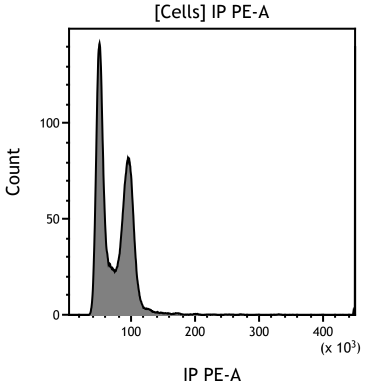

New Composite 8 - Plot Sheet 2

7_CD430_7.2
[Ungated] IP PE-A / SSC-A

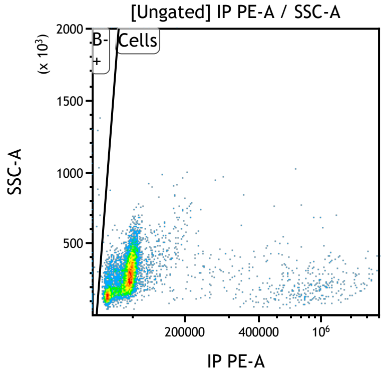

7_CD430_7.2
[Cells] FSC-A / SSC-A

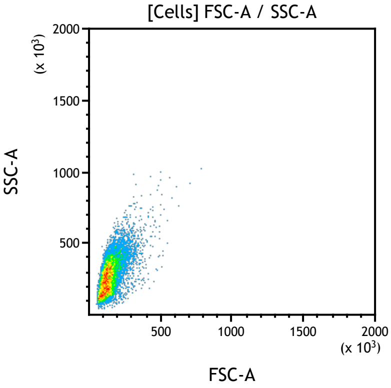

7_CD430_7.2
[Cells] IP PE-A

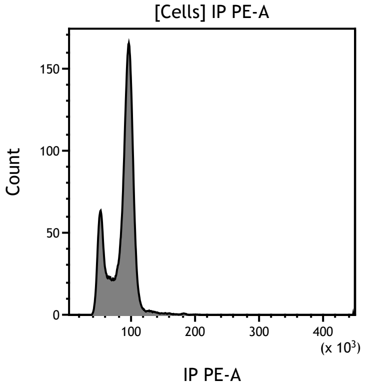

New Composite 8 - Plot Sheet 3

7_CD430_7.3
[Ungated] IP PE-A / SSC-A

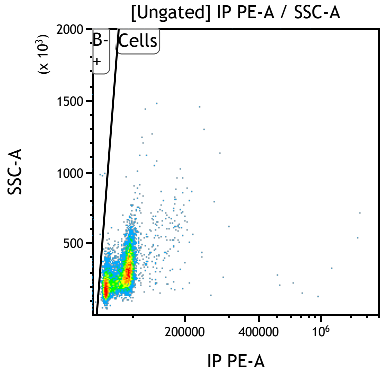

7_CD430_7.3
[Cells] FSC-A / SSC-A

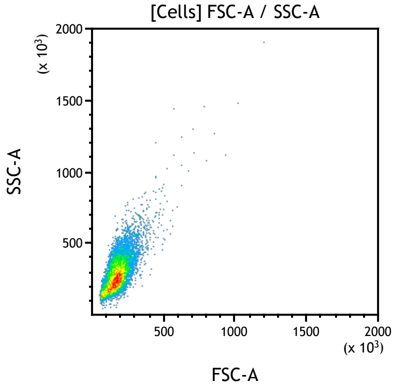

7_CD430_7.3
[Cells] IP PE-A

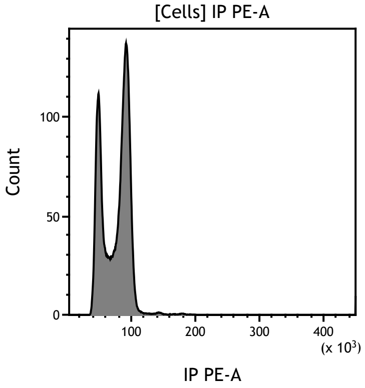

New Composite 8 - Plot Sheet 4

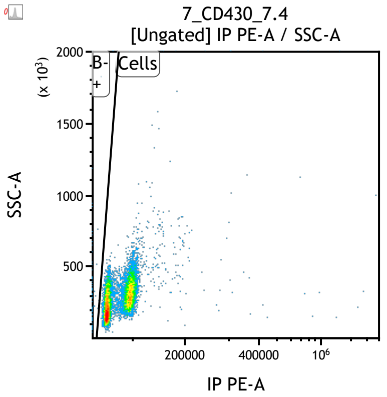

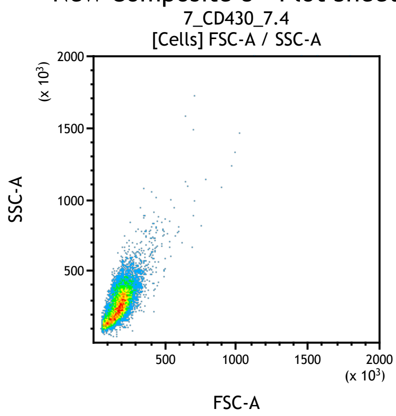

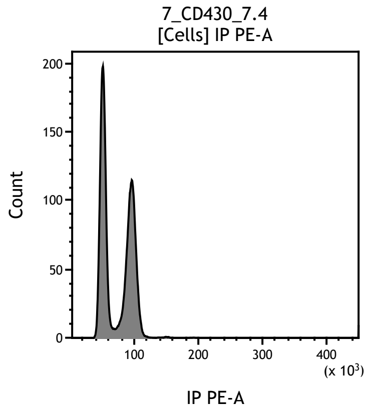

New Composite 8 - Plot Sheet 5

7_CD430_7.5
[Ungated] IP PE-A / SSC-A

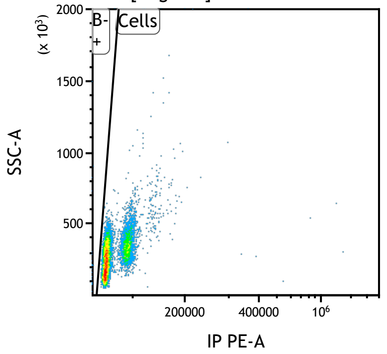

7_CD430_7.5
[Cells] FSC-A / SSC-A

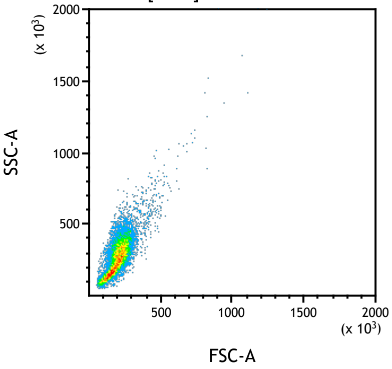

7_CD430_7.5
[Cells] IP PE-A

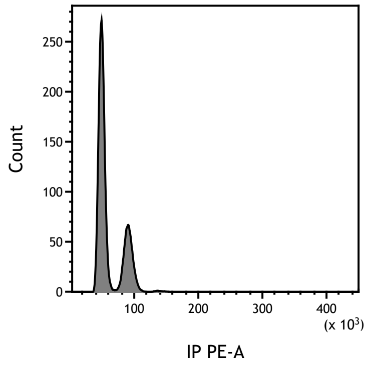

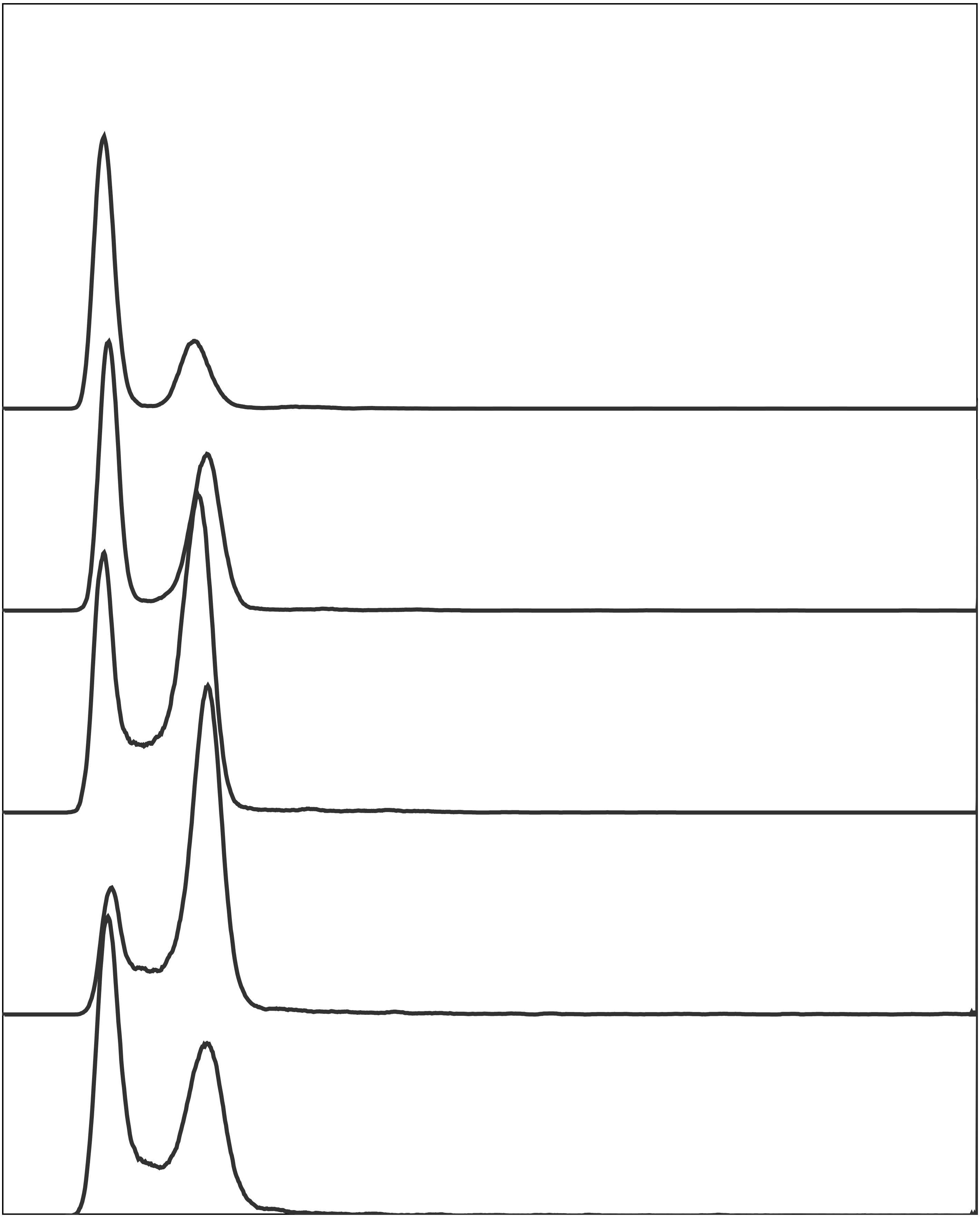

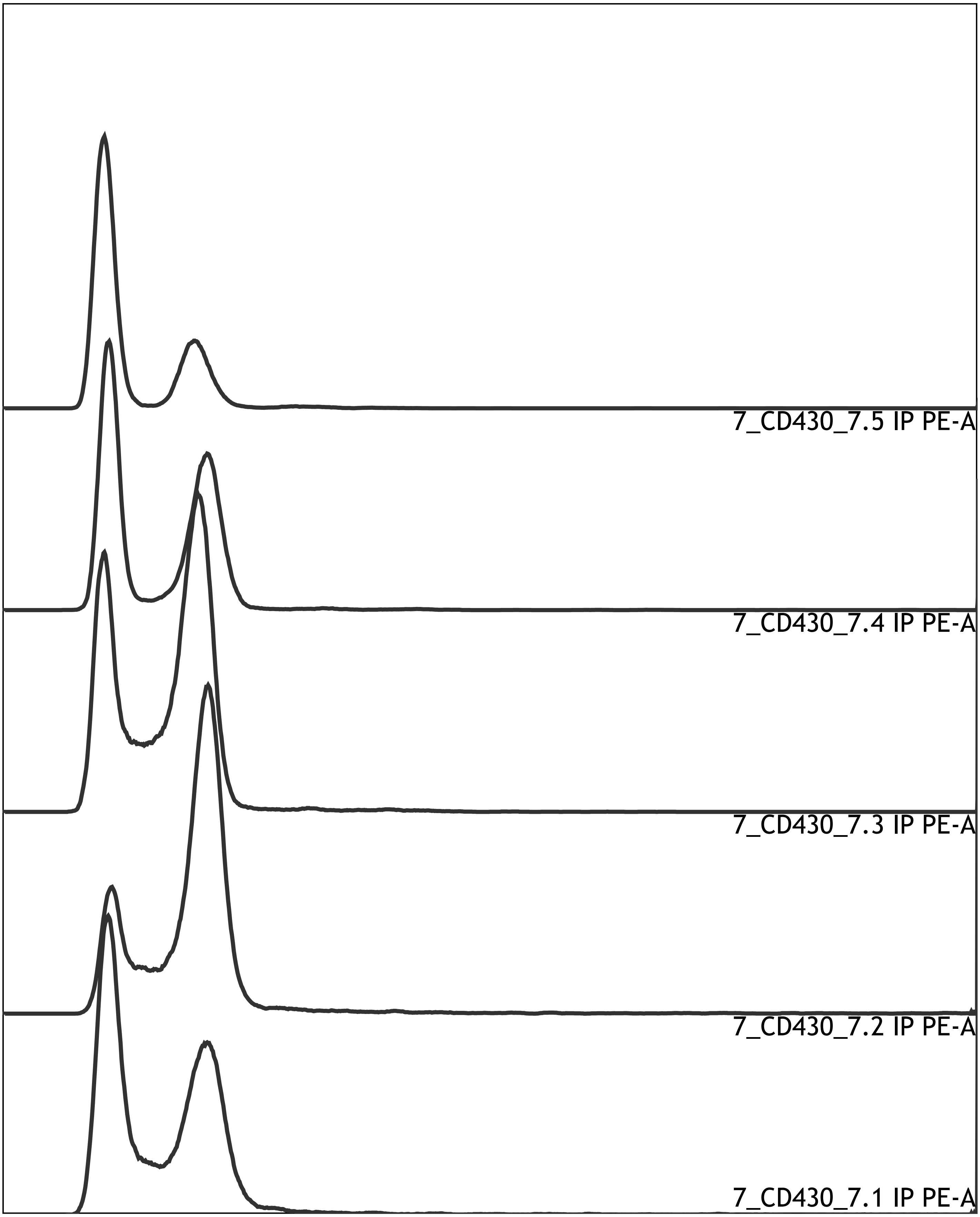
7_CD430_7.5 IP PE-A
7_CD430_7.4 IP PE-A
7_CD430_7.3 IP PE-A
7_CD430_7.2 IP PE-A
7_CD430_7.1 IP PE-A

New Composite 10 - Plot Sheet 1

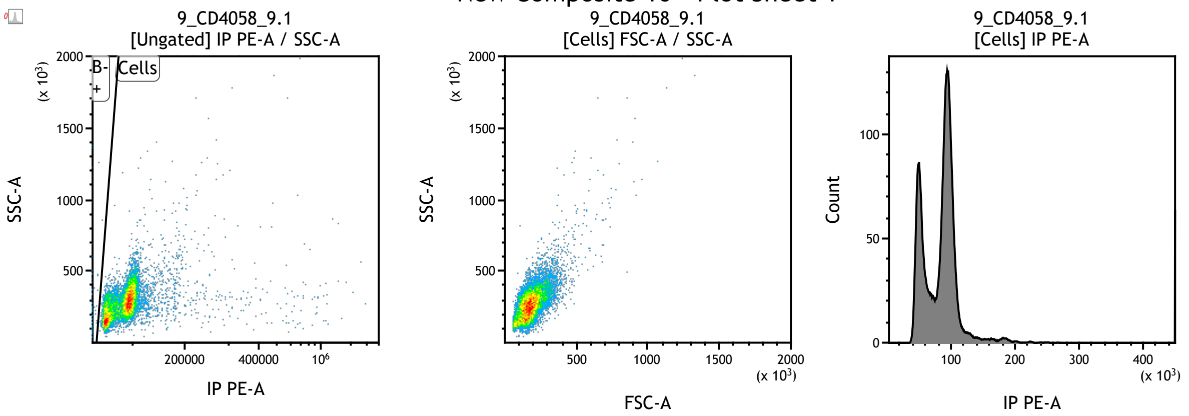

New Composite 10 - Plot Sheet 2

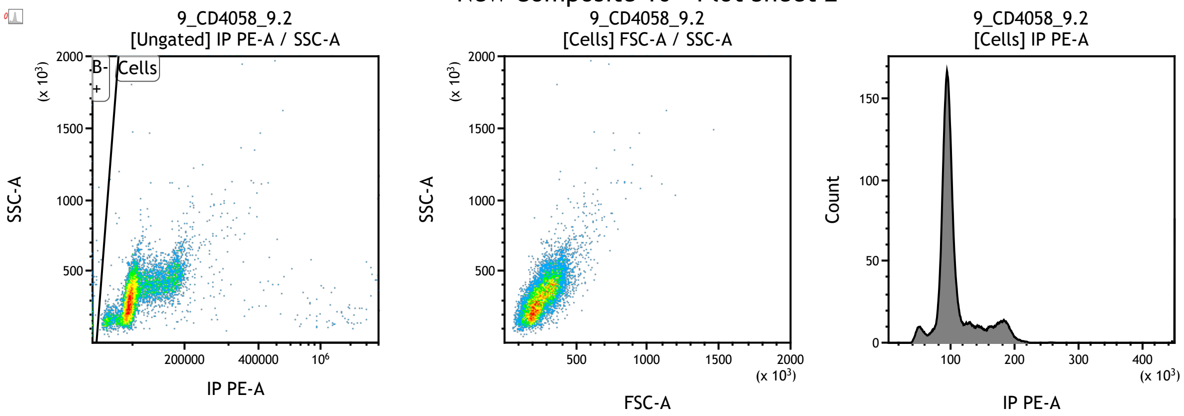

New Composite 10 - Plot Sheet 3

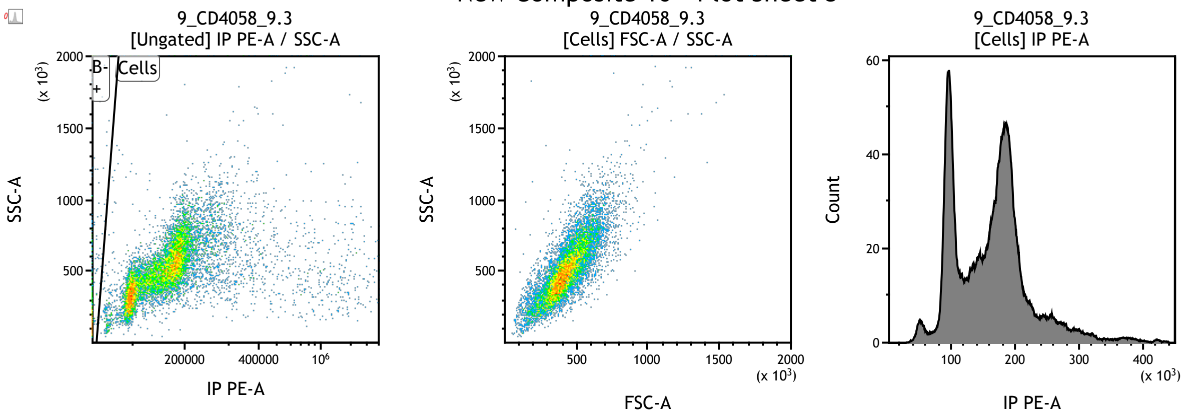

New Composite 10 - Plot Sheet 4

9_CD4058_9.4
[Ungated] IP PE-A / SSC-A

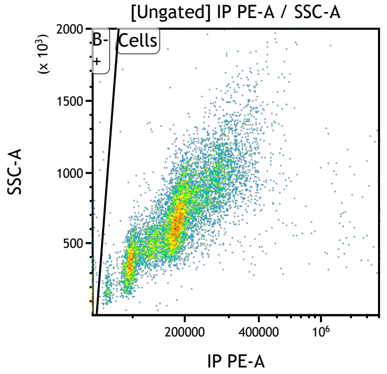

9_CD4058_9.4
[Cells] FSC-A / SSC-A

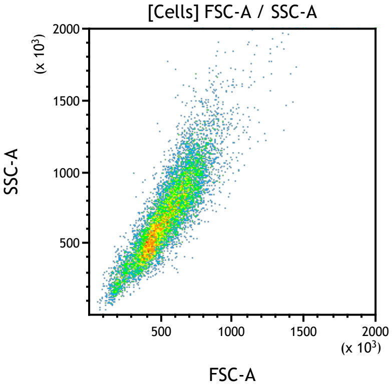

9_CD4058_9.4
[Cells] IP PE-A

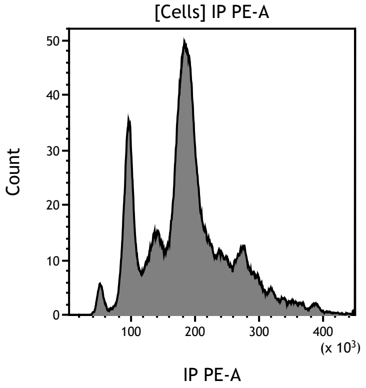

New Composite 10 - Plot Sheet 5

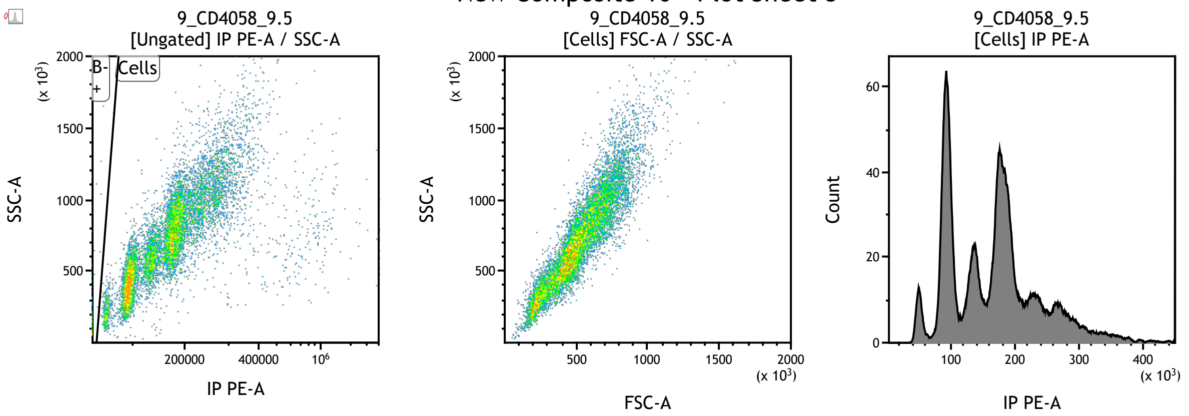

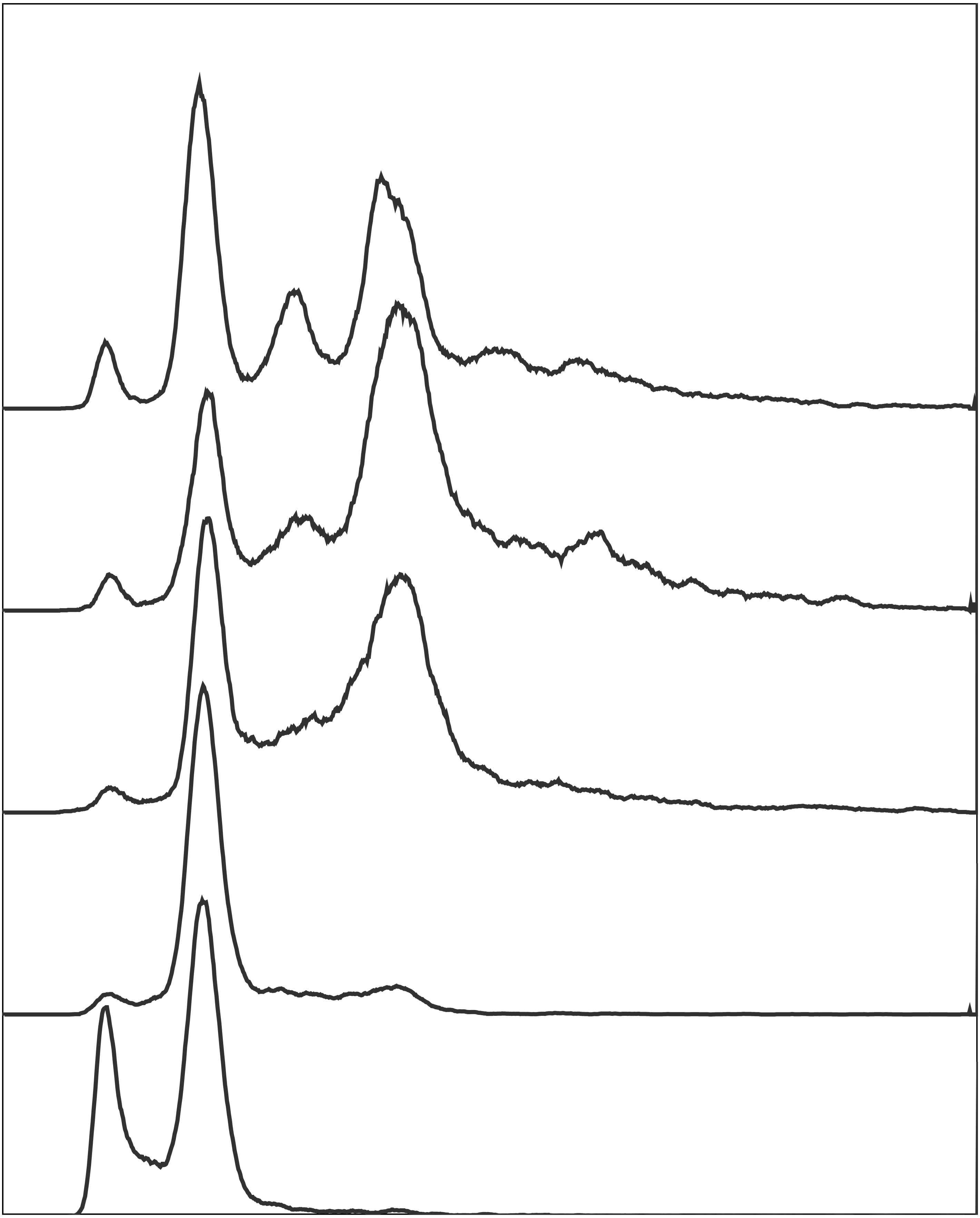

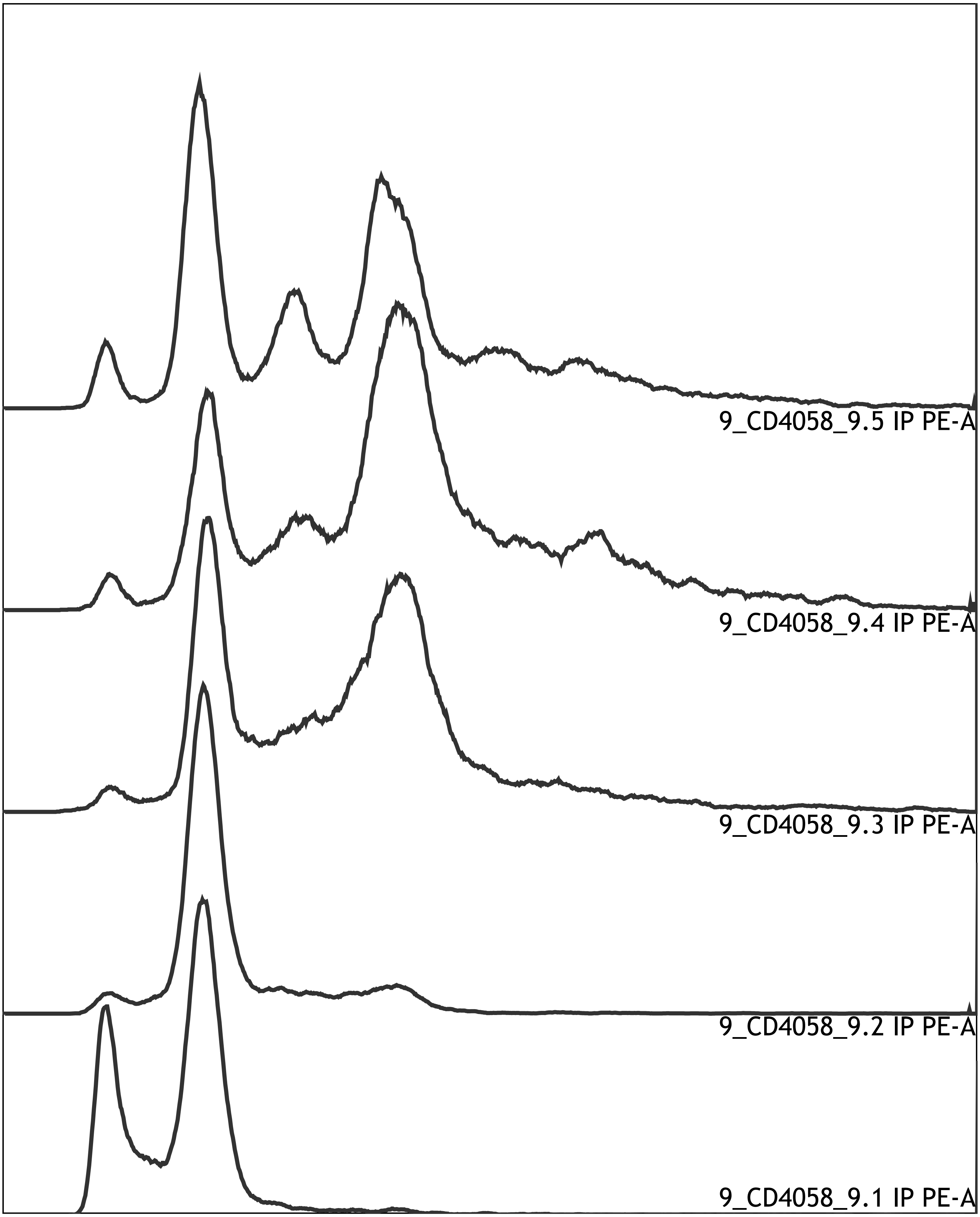
9_CD4058_9.5 IP PE-A
9_CD4058_9.4 IP PE-A
9_CD4058_9.3 IP PE-A
9_CD4058_9.2 IP PE-A
9_CD4058_9.1 IP PE-A
