# Supplementary material for: TOR complex 1 negatively regulates NDR kinase Cbk1 to control cell separation in budding yeast
Source: PLoS Biol. 2023 Aug 30;21(8):e3002263. doi: 10.1371/journal.pbio.3002263 (PMC10468069; doi:10.1371/journal.pbio.3002263)
Supplement: S1 File — (ZIP) [file pbio.3002263.s024.zip › S2D.pdf]

New Composite 8 - Plot Sheet 3

16_CD4315 16.3
[Ungated] IP PE-A / SSC-A

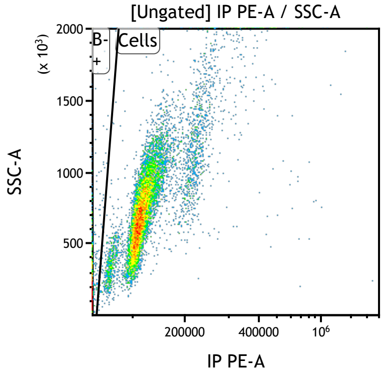

16_CD4315 16.3
[Cells] FSC-A / SSC-A

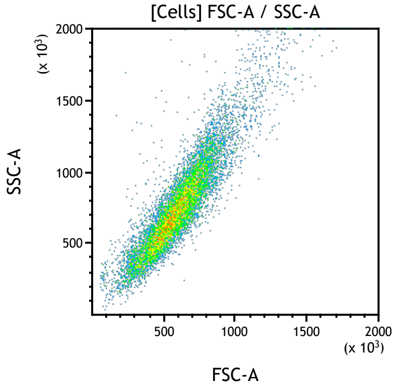

16_CD4315 16.3
[Cells] IP PE-A

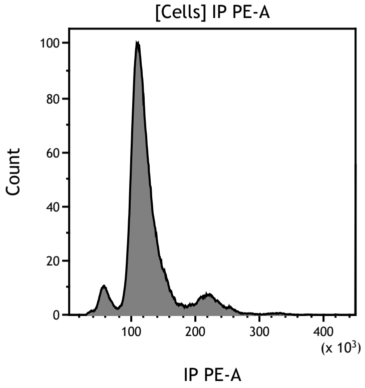

New Composite 8 - Plot Sheet 4

16_CD4315 16.4
[Ungated] IP PE-A / SSC-A

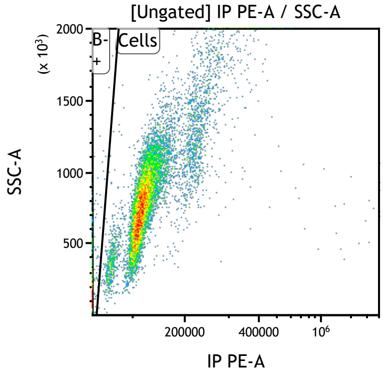

16_CD4315 16.4
[Cells] FSC-A / SSC-A

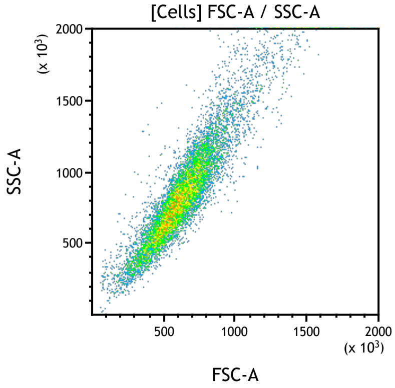

16_CD4315 16.4
[Cells] IP PE-A

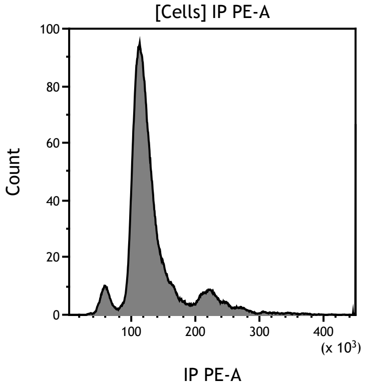

New Composite 8 - Plot Sheet 5

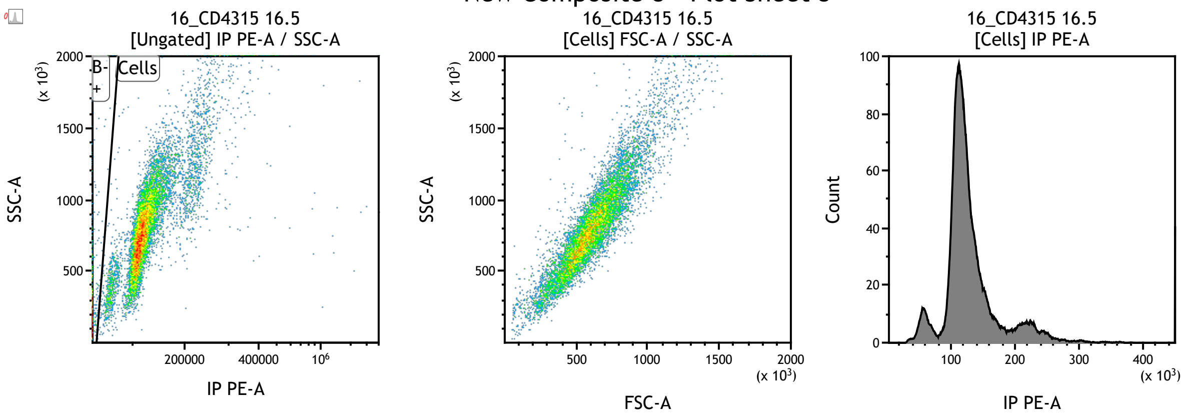

New Composite 8 - Plot Sheet 6

16_CD4315 16.6
[Ungated] IP PE-A / SSC-A

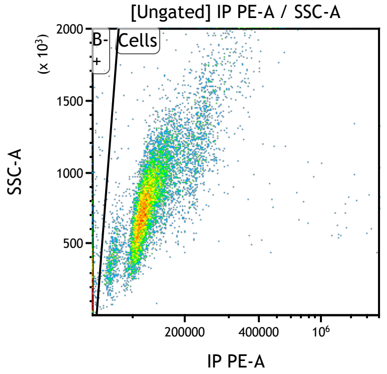

16_CD4315 16.6
[Cells] FSC-A / SSC-A

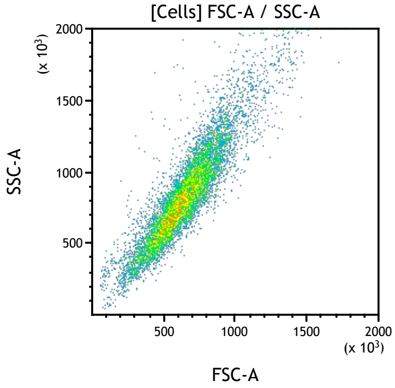

16_CD4315 16.6
[Cells] IP PE-A

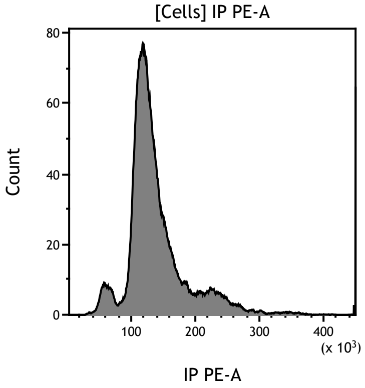

New Composite 8 - Plot Sheet 7

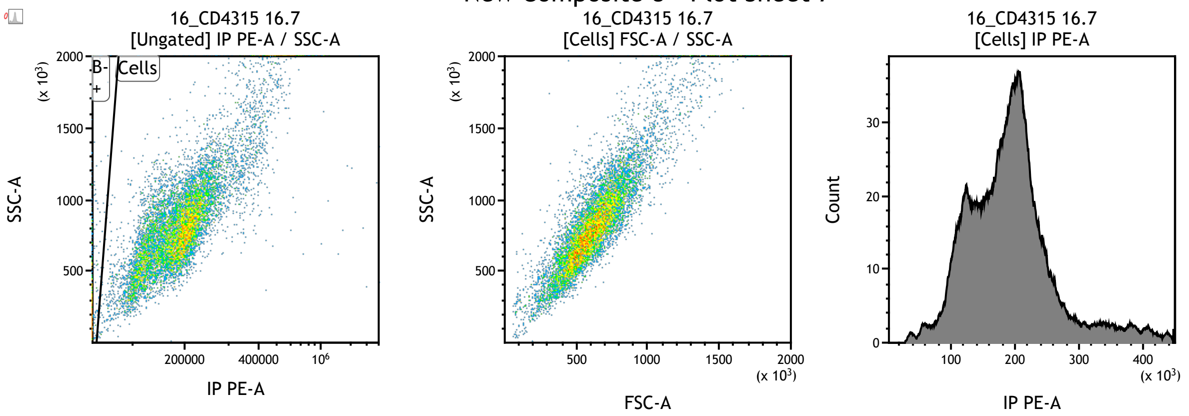

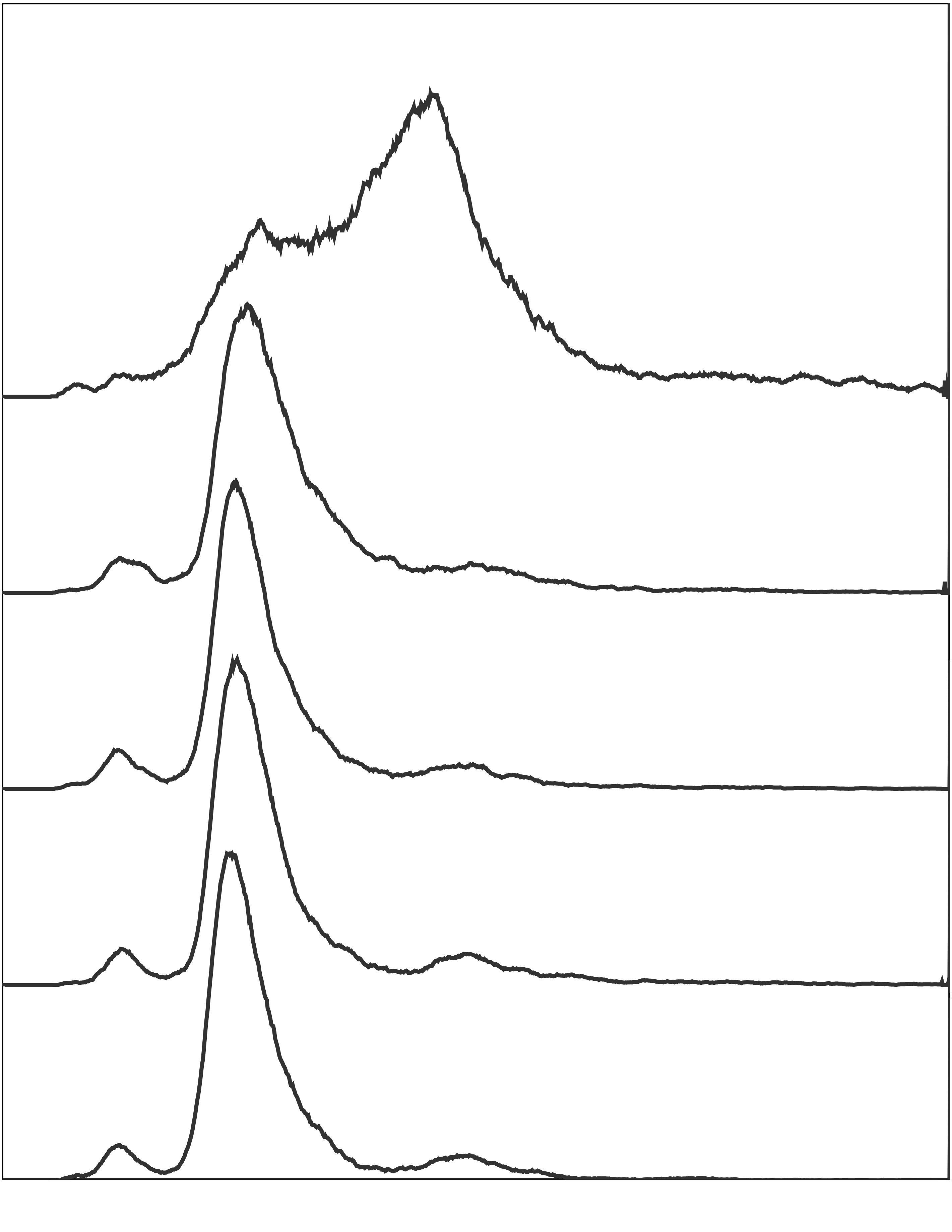

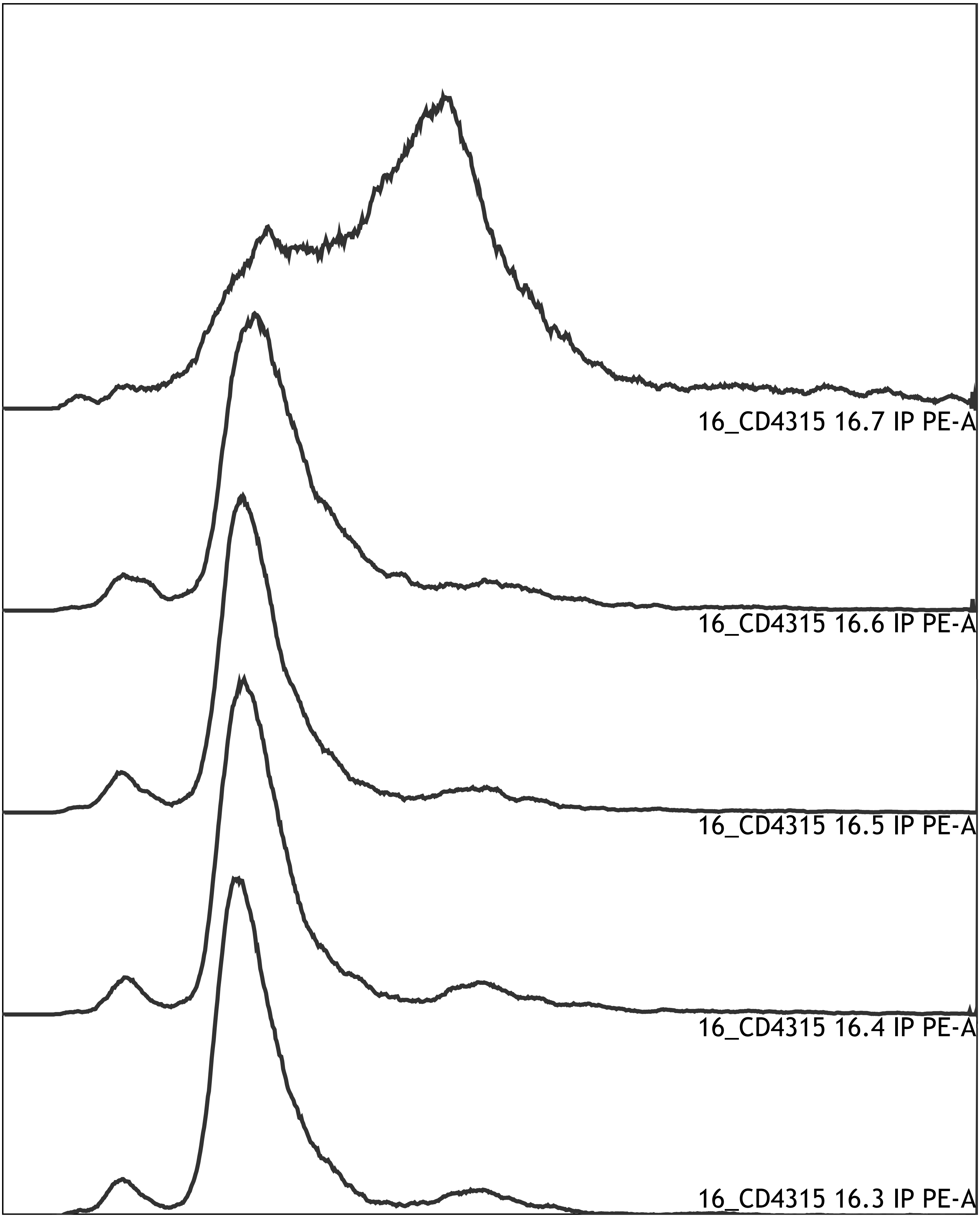
16_CD4315 16.7 IP PE-A
16_CD4315 16.6 IP PE-A
16_CD4315 16.5 IP PE-A
16_CD4315 16.4 IP PE-A
16_CD4315 16.3 IP PE-A

New Composite 9 - Plot Sheet 3

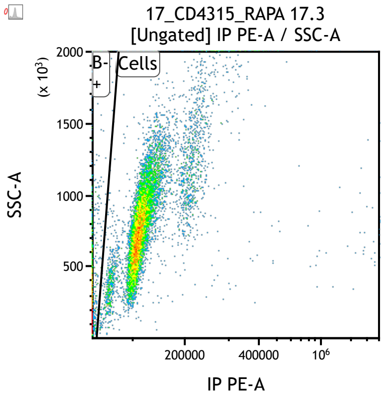

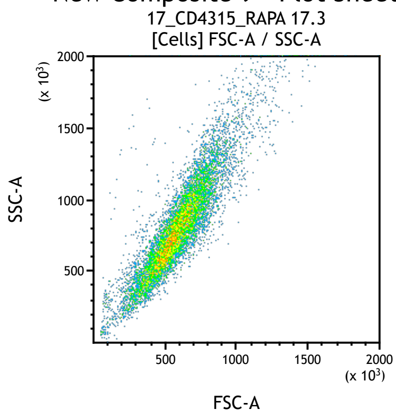

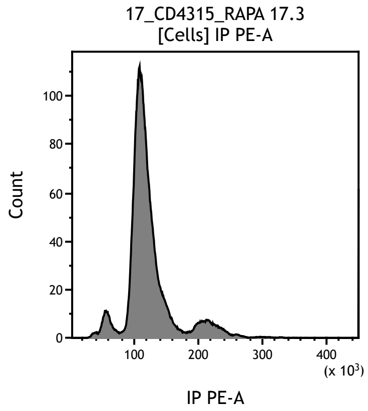

New Composite 9 - Plot Sheet 4

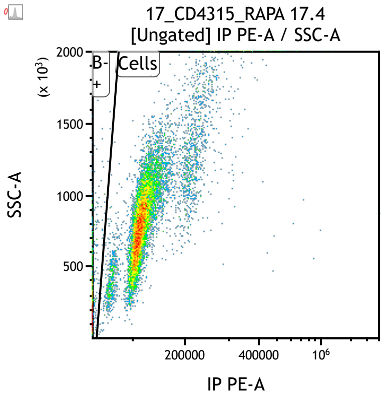

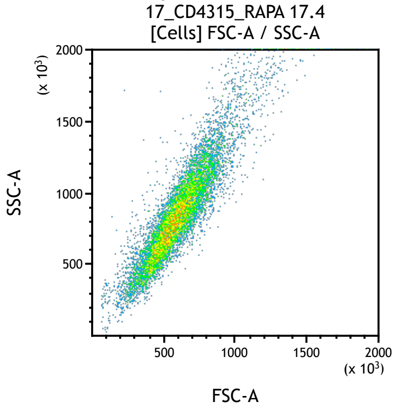

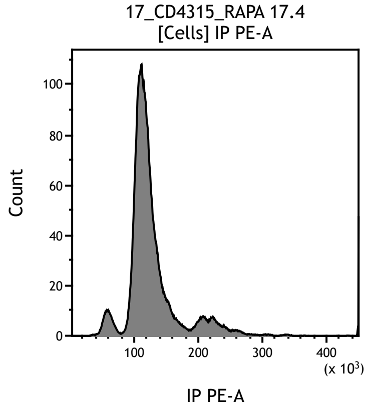

New Composite 9 - Plot Sheet 5

17_CD4315_RAPA 17.5
[Ungated] IP PE-A / SSC-A

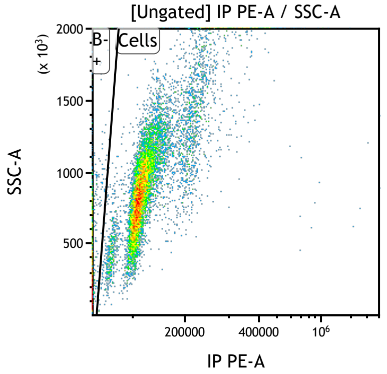

17_CD4315_RAPA 17.5
[Cells] FSC-A / SSC-A

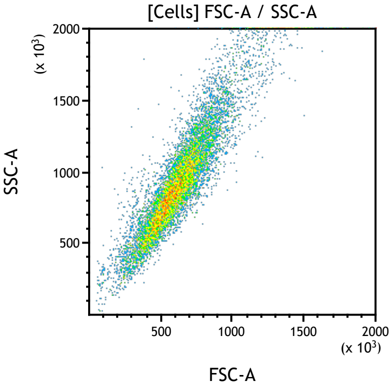

17_CD4315_RAPA 17.5
[Cells] IP PE-A

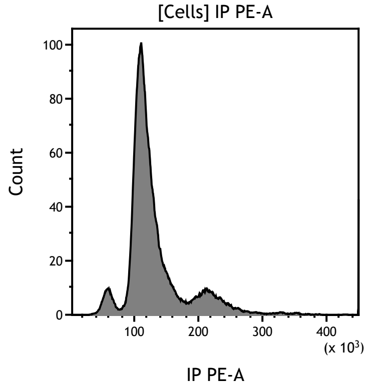

New Composite 9 - Plot Sheet 6

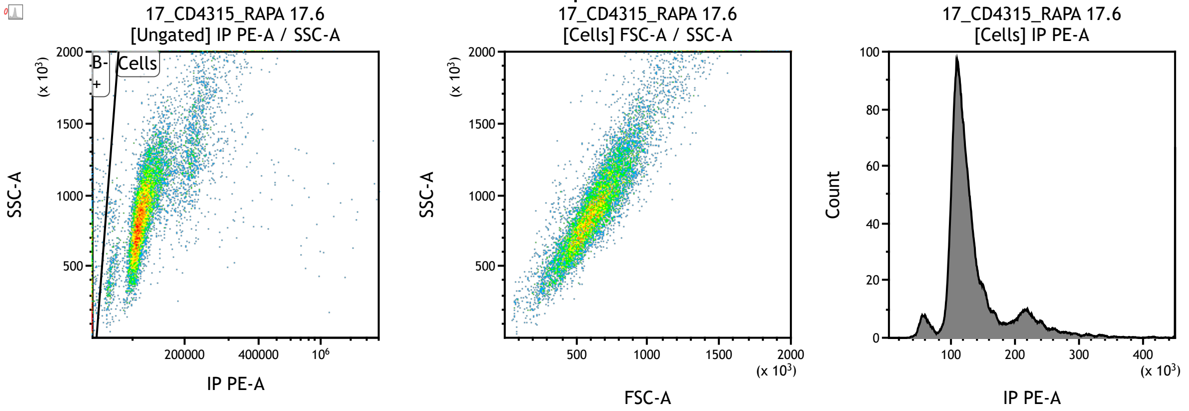

New Composite 9 - Plot Sheet 7

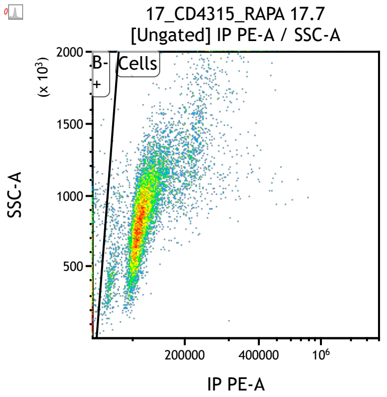

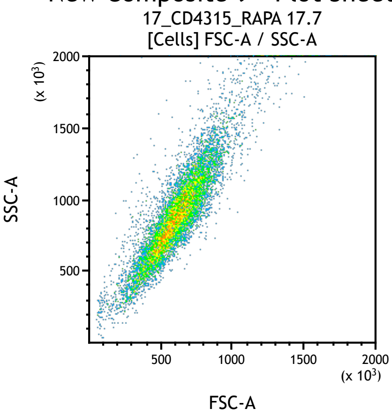

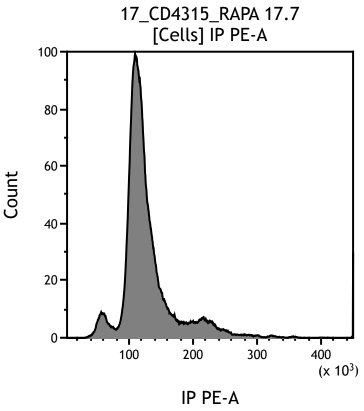

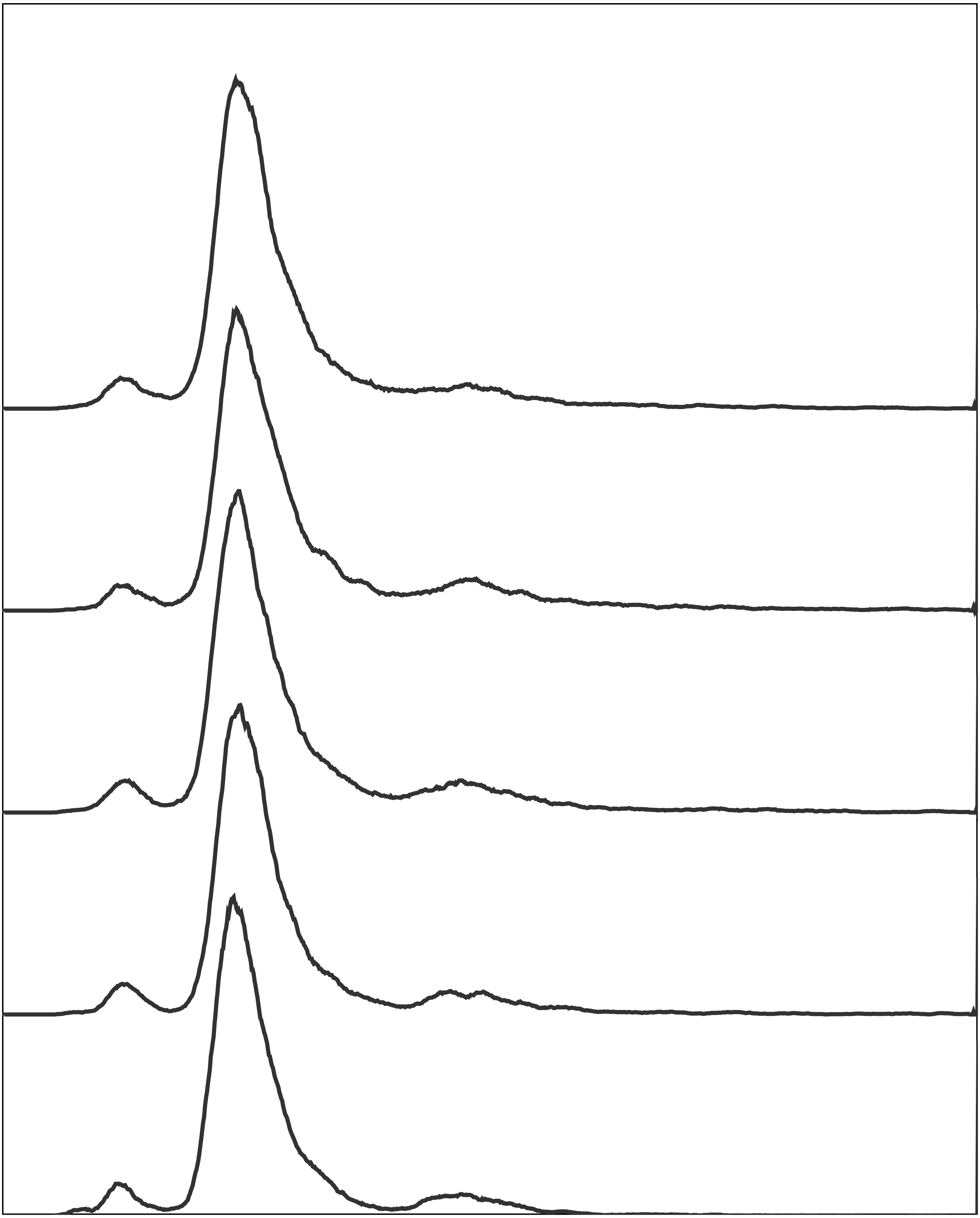

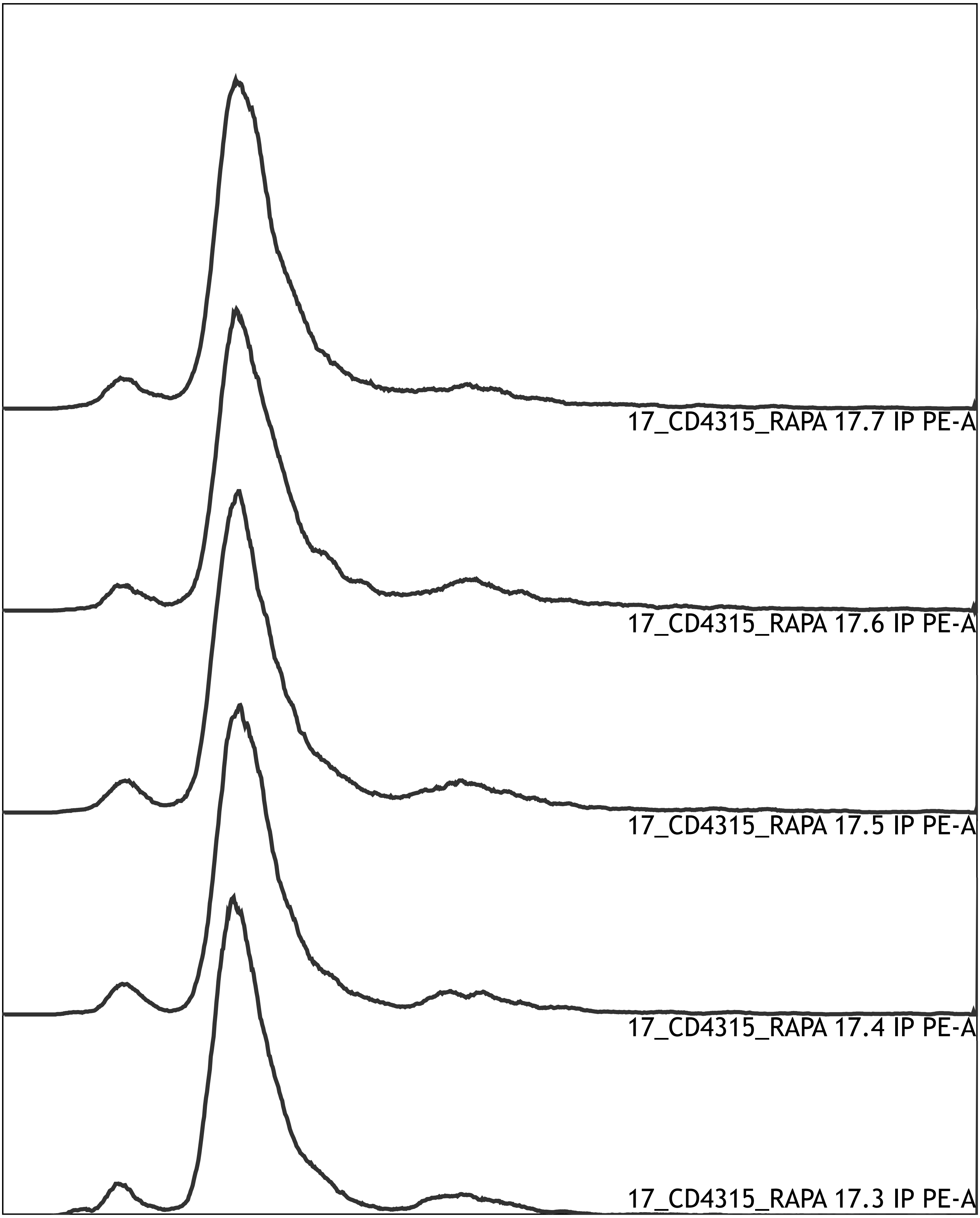
17_CD4315_RAPA 17.7 IP PE-A
17_CD4315_RAPA 17.6 IP PE-A
17_CD4315_RAPA 17.5 IP PE-A
17_CD4315_RAPA 17.4 IP PE-A
17_CD4315_RAPA 17.3 IP PE-A
