# Supplementary material for: TOR complex 1 negatively regulates NDR kinase Cbk1 to control cell separation in budding yeast
Source: PLoS Biol. 2023 Aug 30;21(8):e3002263. doi: 10.1371/journal.pbio.3002263 (PMC10468069; doi:10.1371/journal.pbio.3002263)
Supplement: S1 File — (ZIP) [file pbio.3002263.s024.zip › S3A.pdf]

New Composite 4 - Plot Sheet 2

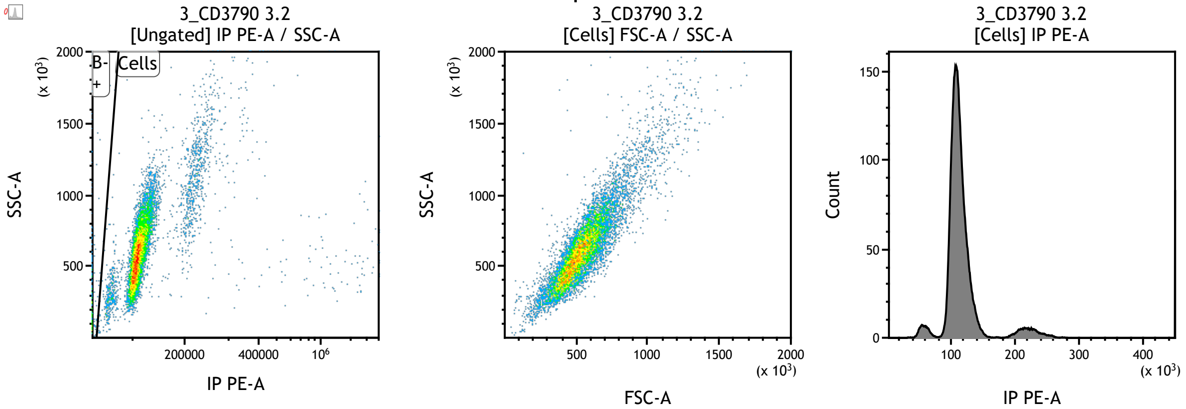

New Composite 4 - Plot Sheet 3

3_CD3790 3.3
[Ungated] IP PE-A / SSC-A

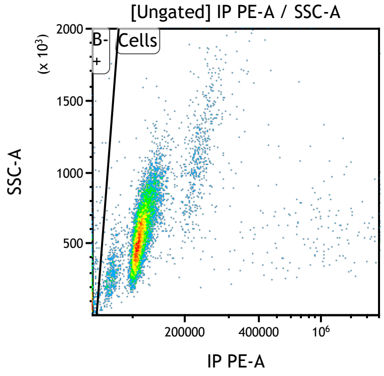

3_CD3790 3.3
[Cells] FSC-A / SSC-A

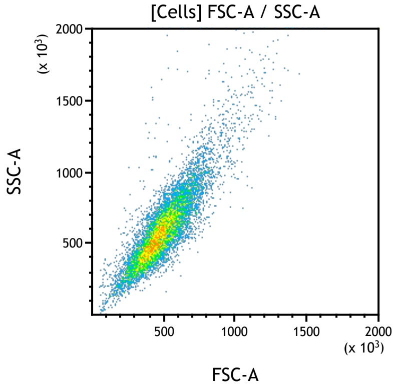

3_CD3790 3.3
[Cells] IP PE-A

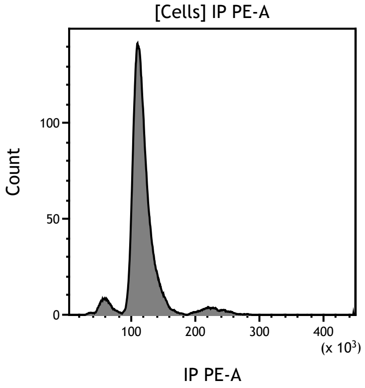

New Composite 4 - Plot Sheet 4

3_CD3790 3.4
[Ungated] IP PE-A / SSC-A

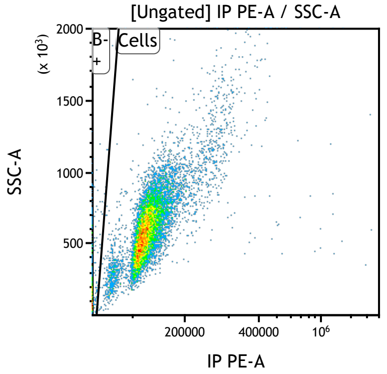

3_CD3790 3.4
[Cells] FSC-A / SSC-A

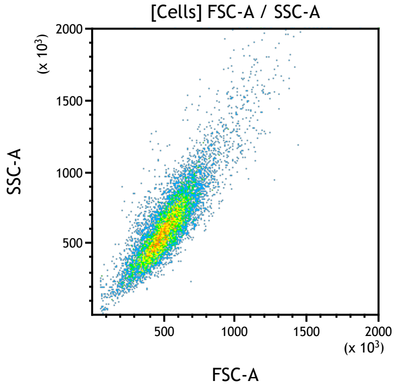

3_CD3790 3.4
[Cells] IP PE-A

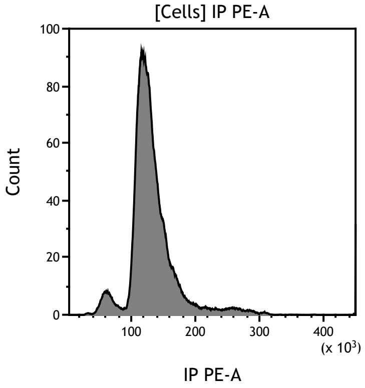

New Composite 4 - Plot Sheet 5

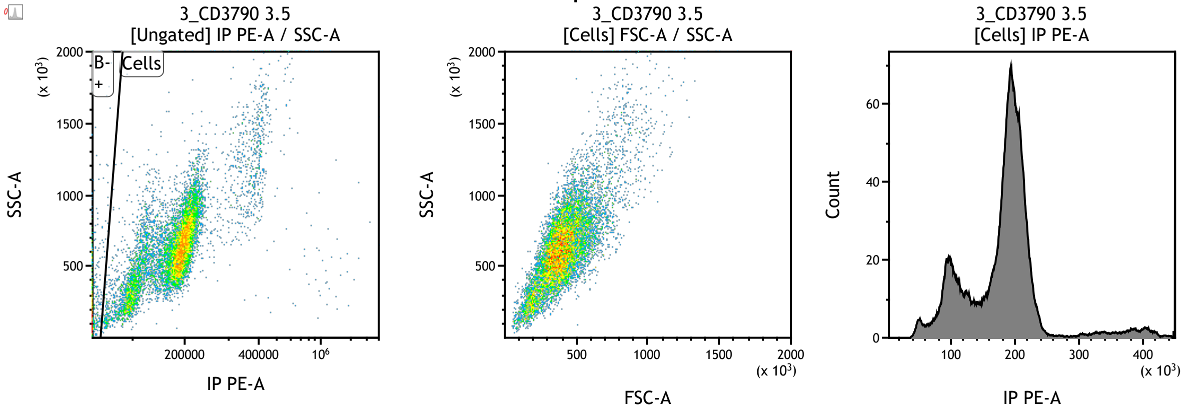

New Composite 4 - Plot Sheet 6

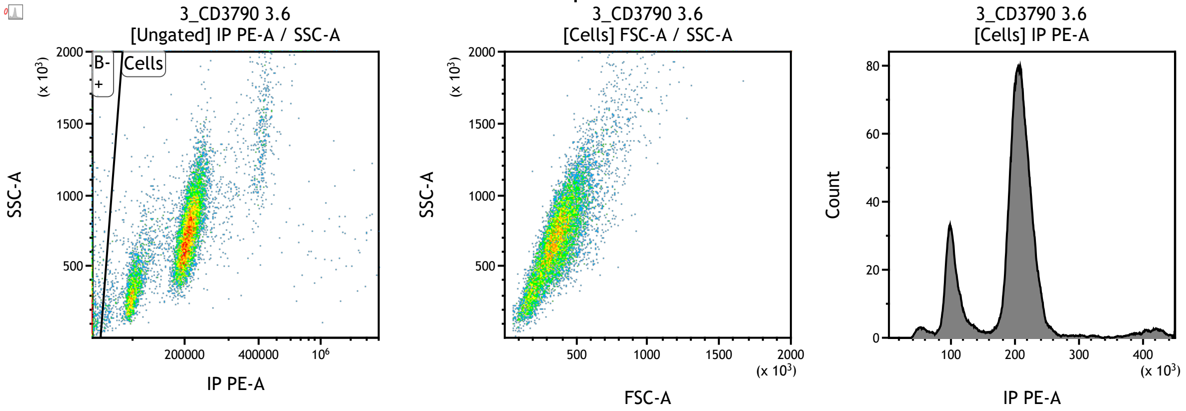

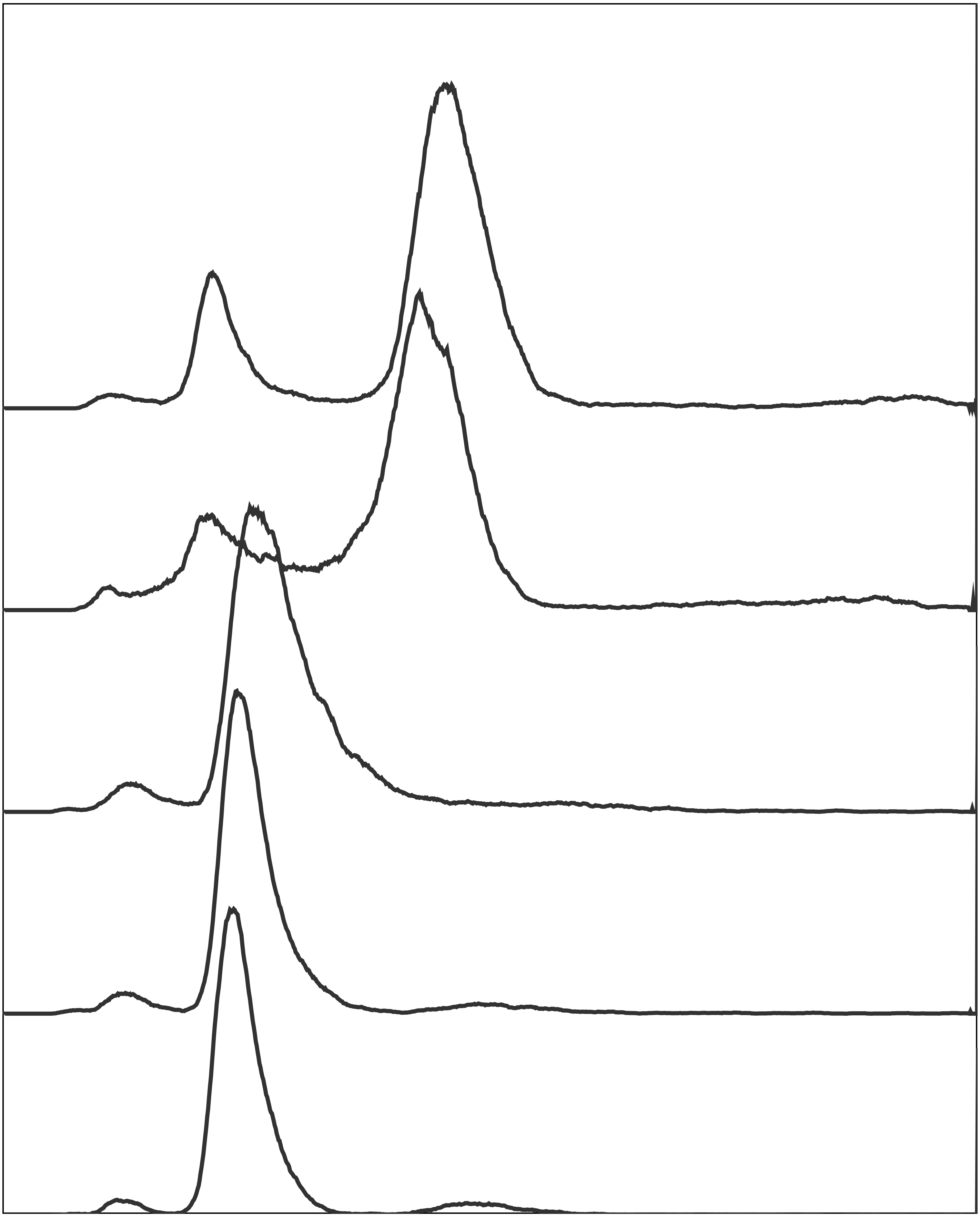

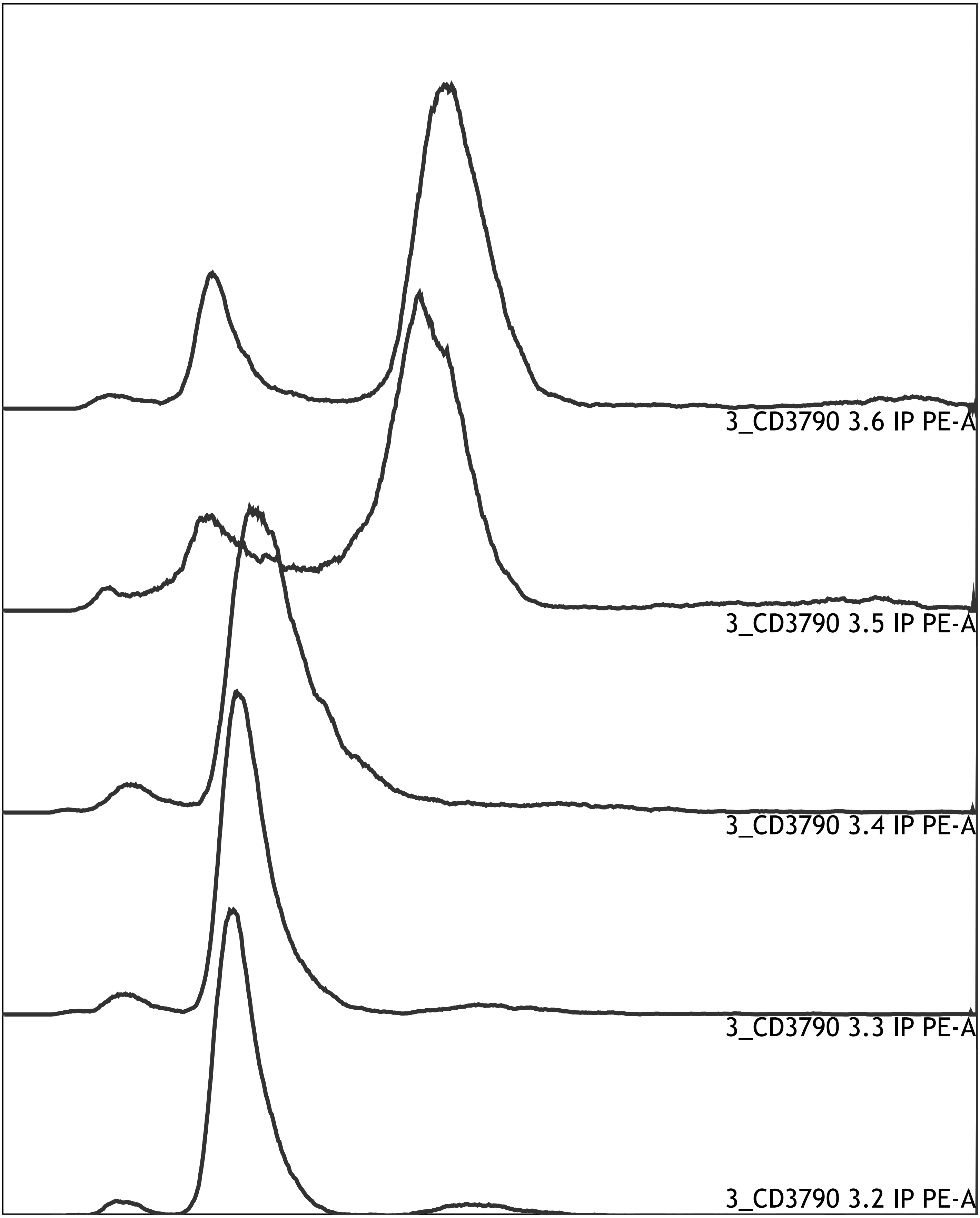
3_CD3790 3.6 IP PE-A
3_CD3790 3.5 IP PE-A
3_CD3790 3.4 IP PE-A
3_CD3790 3.3 IP PE-A
3_CD3790 3.2 IP PE-A

New Composite 5 - Plot Sheet 2

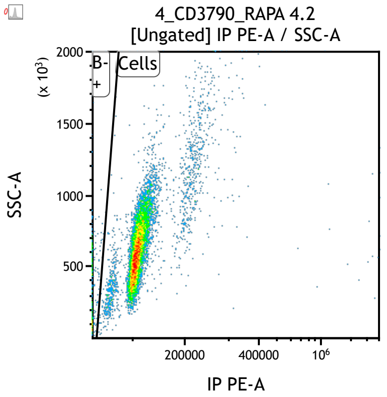

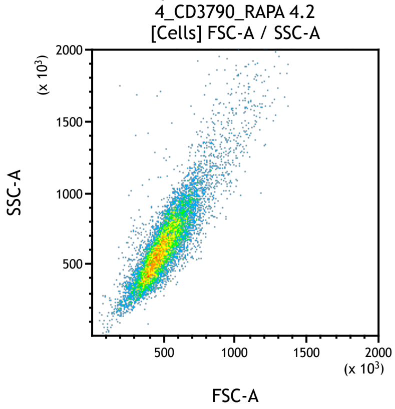

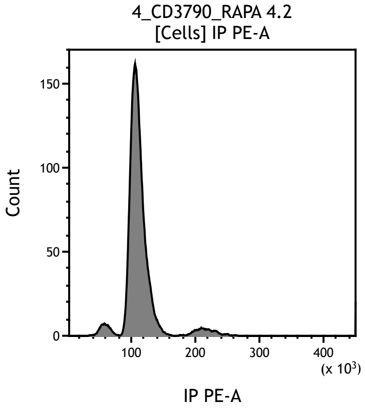

New Composite 5 - Plot Sheet 3

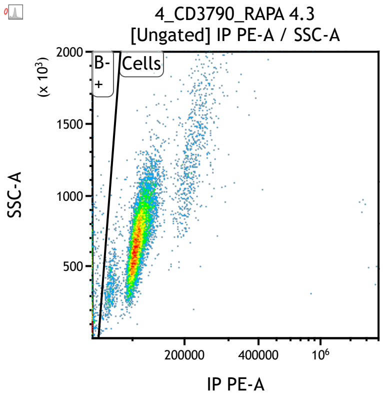

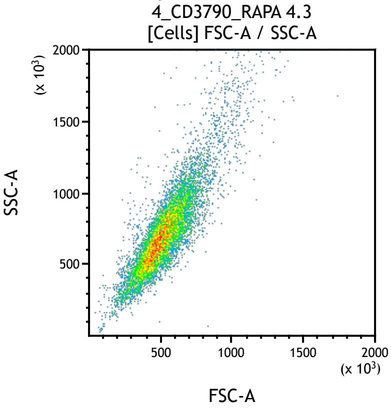

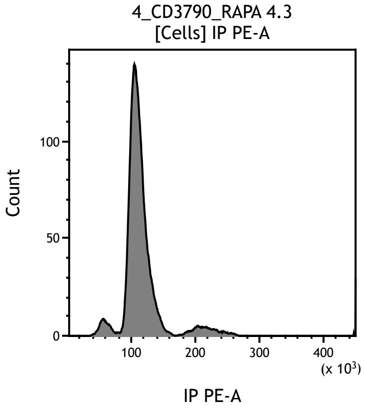

New Composite 5 - Plot Sheet 4

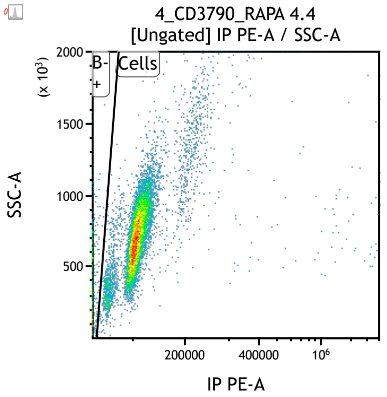

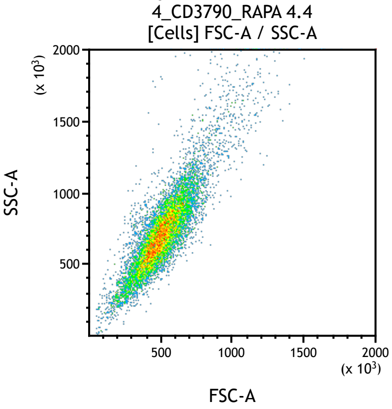

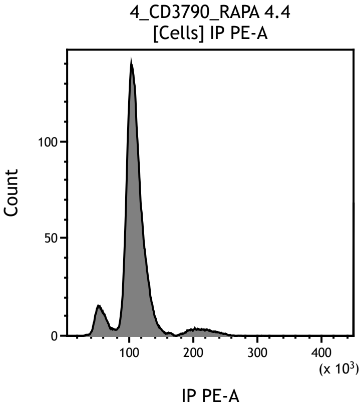

New Composite 5 - Plot Sheet 5

4_CD3790_RAPA 4.5
[Ungated] IP PE-A / SSC-A

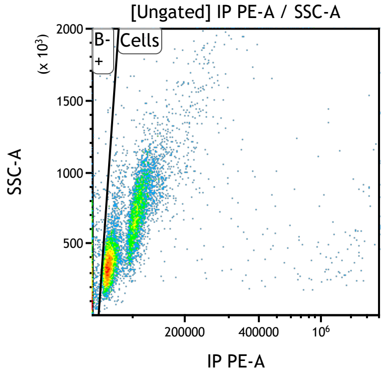

4_CD3790_RAPA 4.5
[Cells] FSC-A / SSC-A

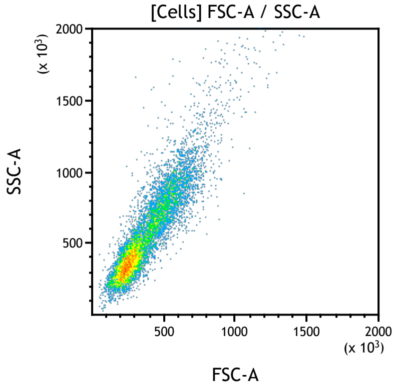

4_CD3790_RAPA 4.5
[Cells] IP PE-A

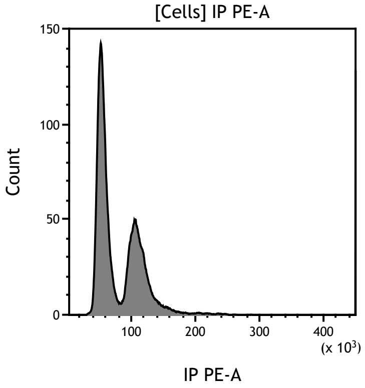

New Composite 5 - Plot Sheet 6

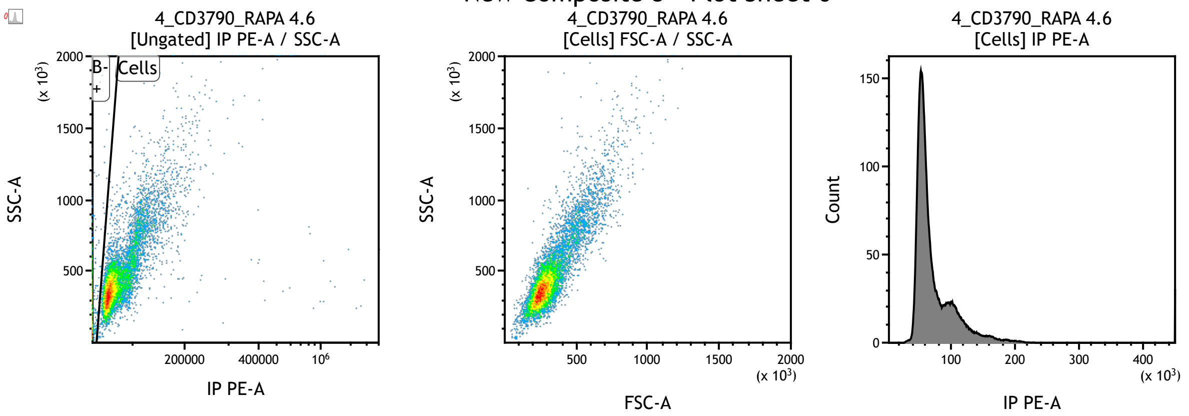

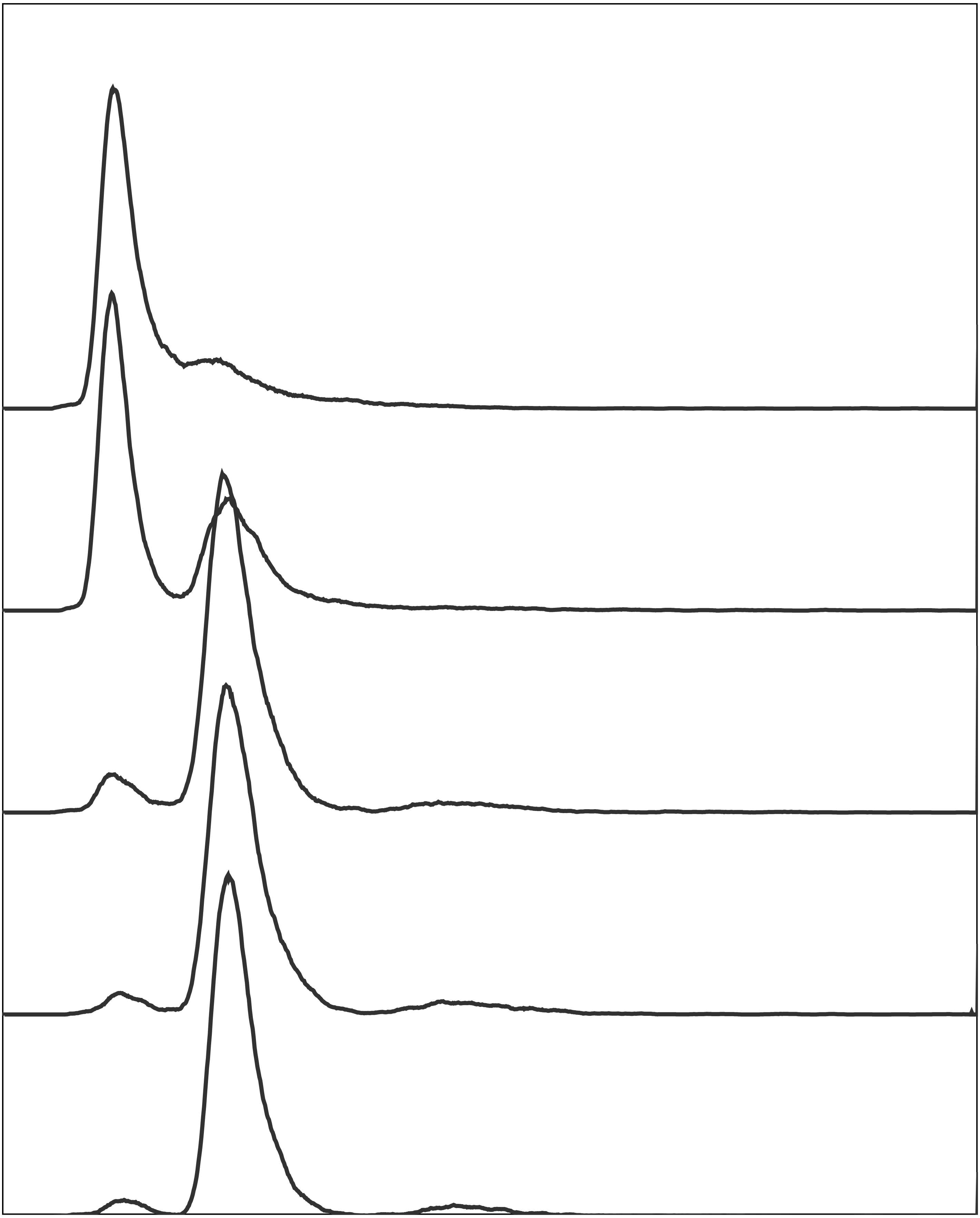

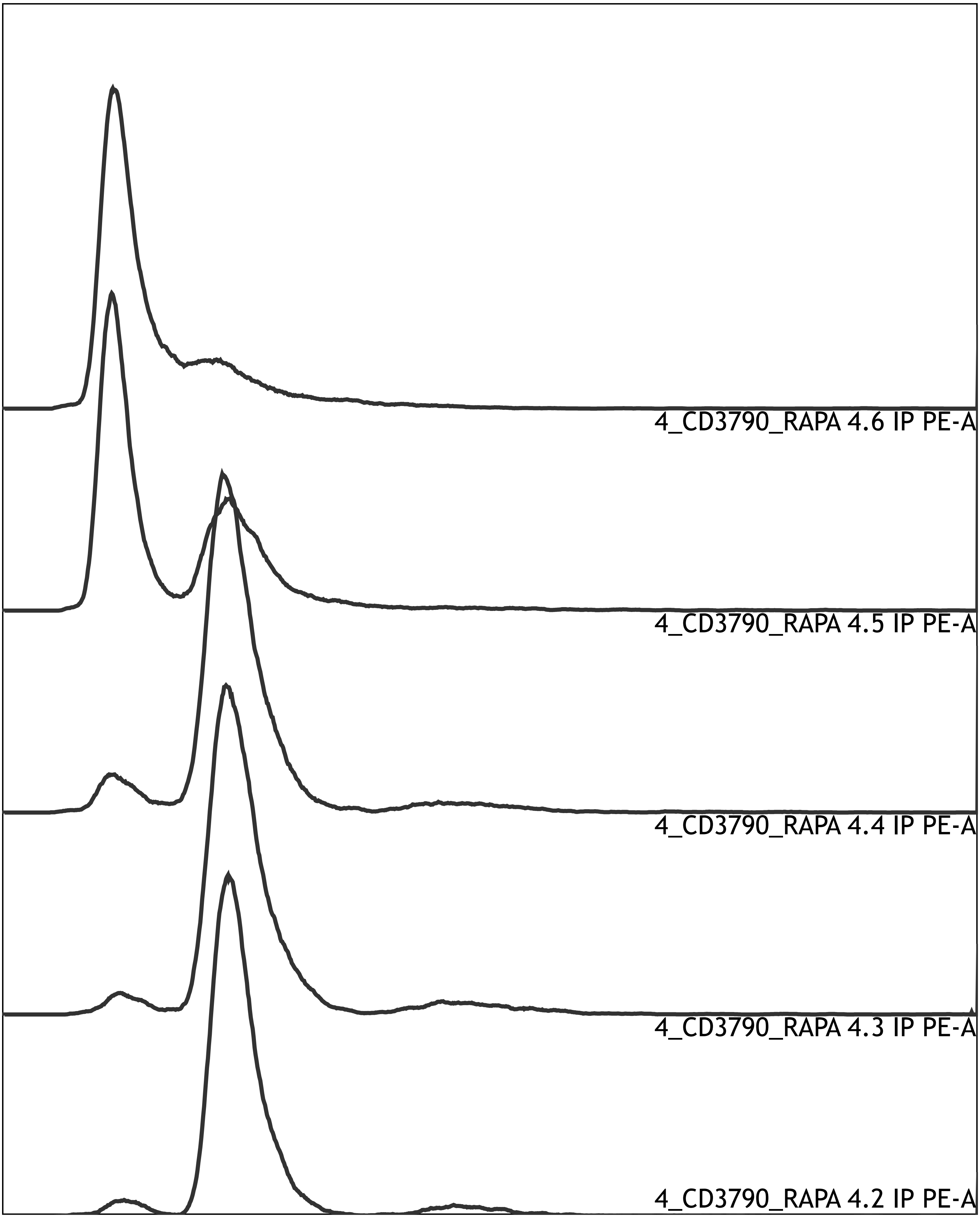
4_CD3790_RAPA 4.6 IP PE-A
4_CD3790_RAPA 4.5 IP PE-A
4_CD3790_RAPA 4.4 IP PE-A
4_CD3790_RAPA 4.3 IP PE-A
4_CD3790_RAPA 4.2 IP PE-A
