# Supplementary material for: TOR complex 1 negatively regulates NDR kinase Cbk1 to control cell separation in budding yeast
Source: PLoS Biol. 2023 Aug 30;21(8):e3002263. doi: 10.1371/journal.pbio.3002263 (PMC10468069; doi:10.1371/journal.pbio.3002263)
Supplement: S1 File — (ZIP) [file pbio.3002263.s024.zip › S3B.pdf]

New Composite 6 - Plot Sheet 2

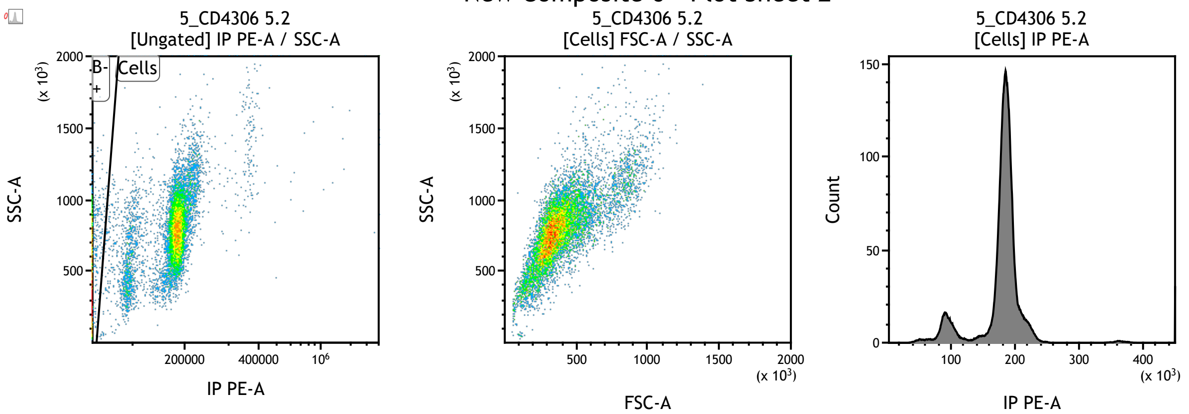

New Composite 6 - Plot Sheet 3

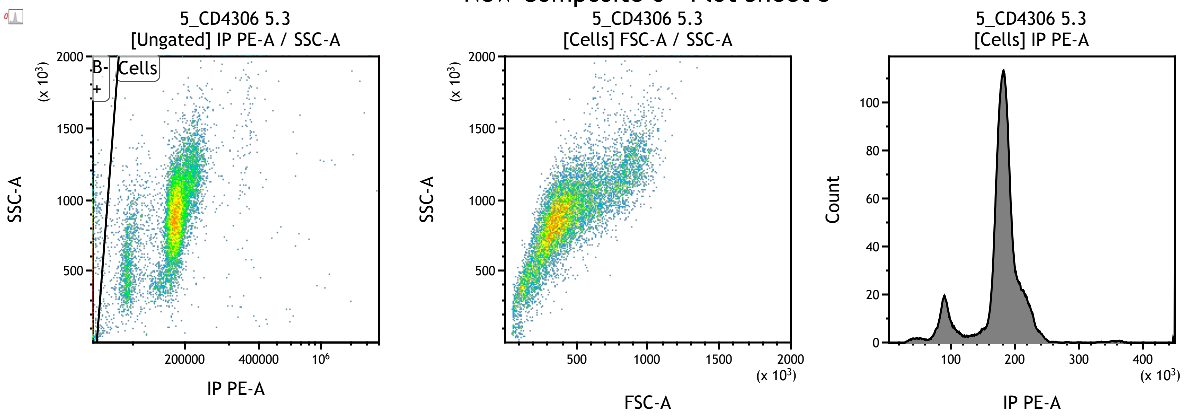

New Composite 6 - Plot Sheet 4

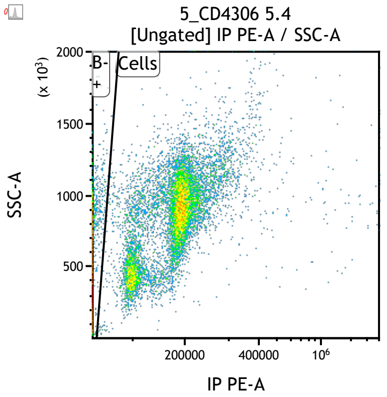

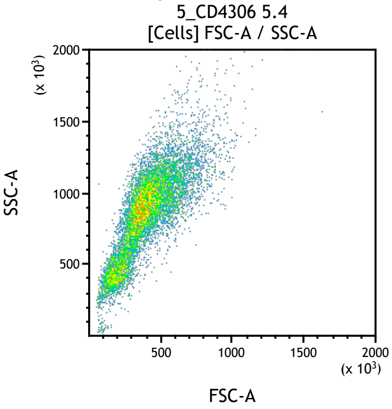

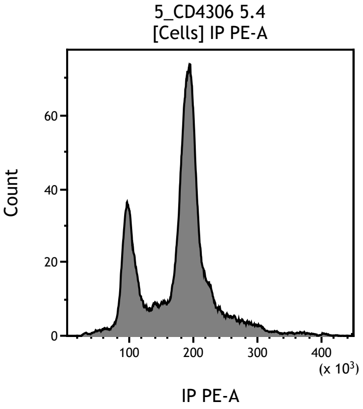

New Composite 6 - Plot Sheet 5

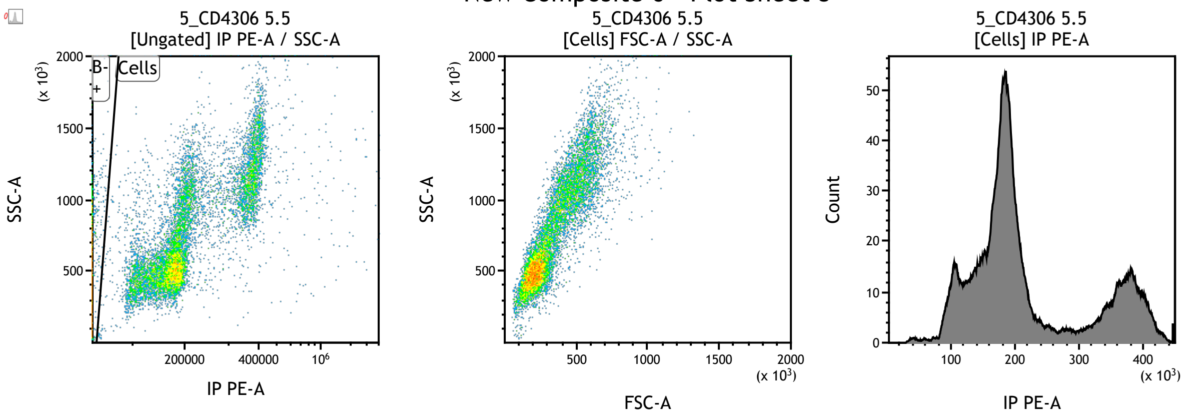

New Composite 6 - Plot Sheet 6

5_CD4306 5.6
[Ungated] IP PE-A / SSC-A

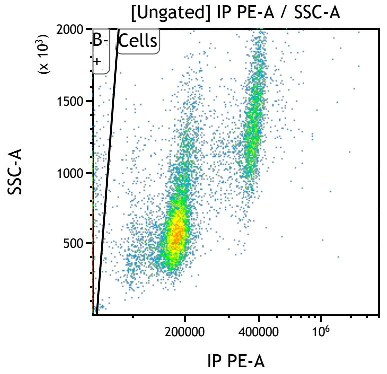

5_CD4306 5.6
[Cells] FSC-A / SSC-A

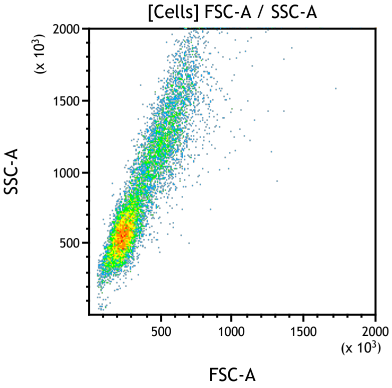

5_CD4306 5.6
[Cells] IP PE-A

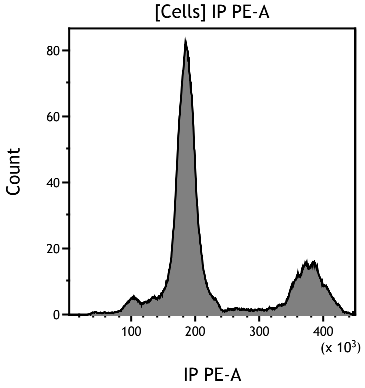

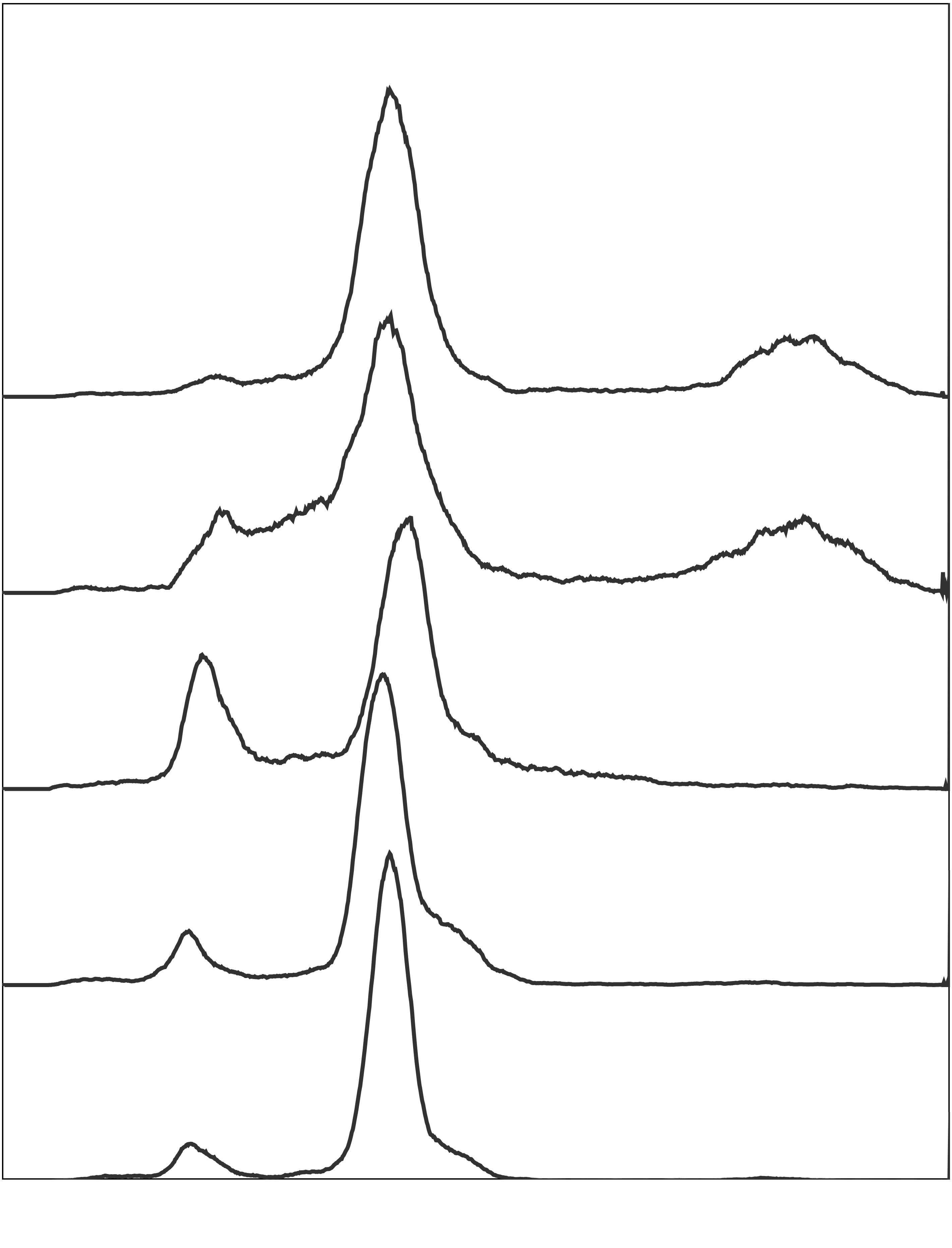

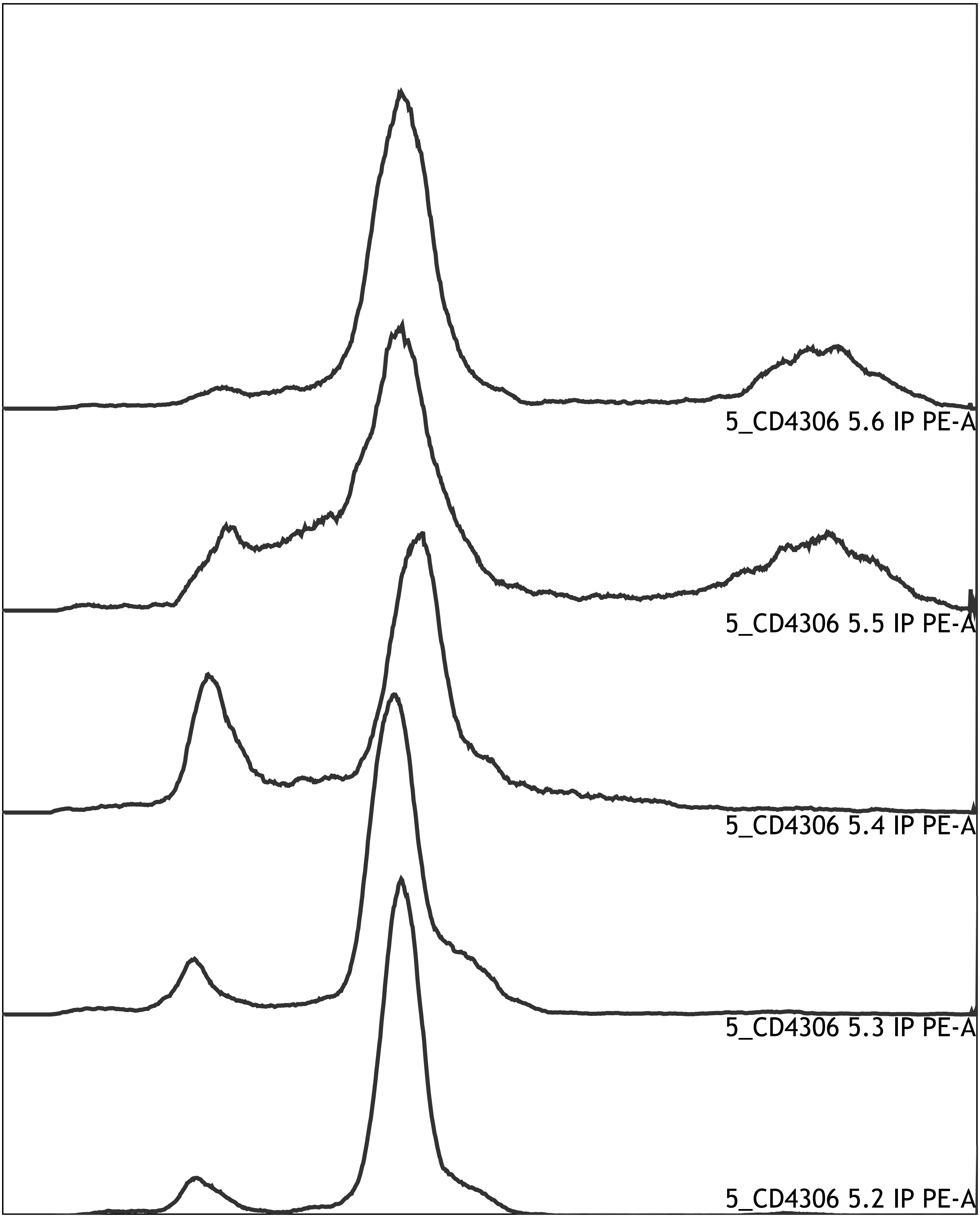
5_CD4306 5.6 IP PE-A
5_CD4306 5.5 IP PE-A
5_CD4306 5.4 IP PE-A
5_CD4306 5.3 IP PE-A
5_CD4306 5.2 IP PE-A

New Composite 7 - Plot Sheet 2

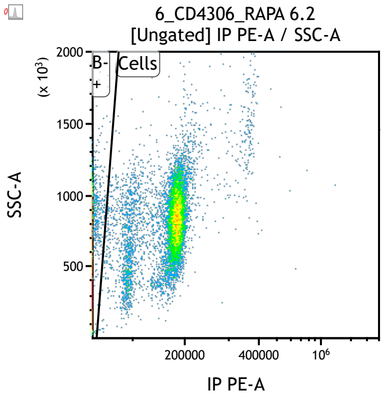

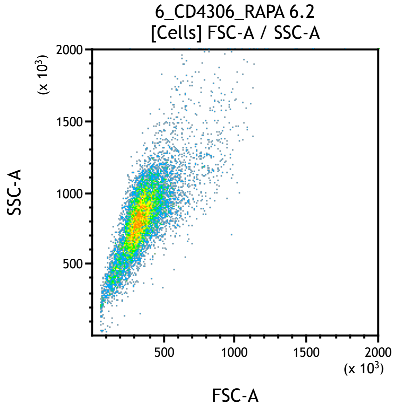

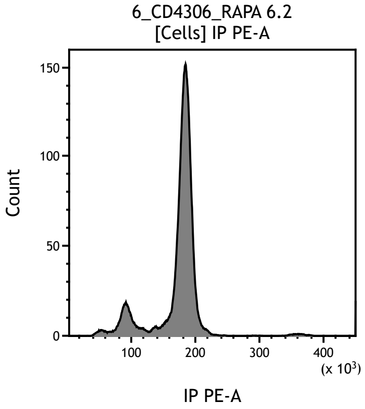

New Composite 7 - Plot Sheet 3

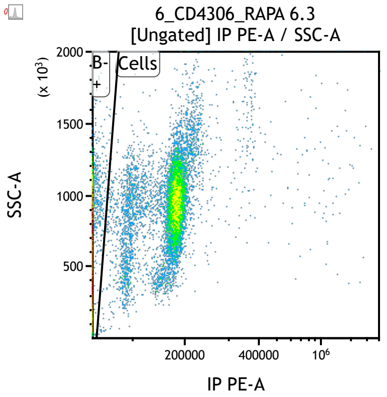

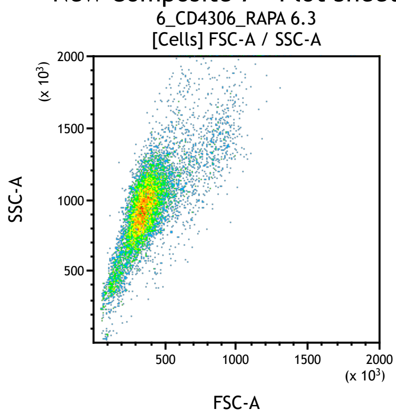

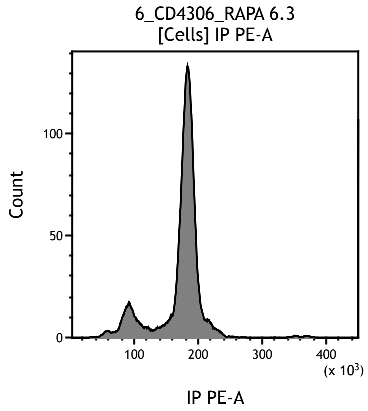

New Composite 7 - Plot Sheet 4

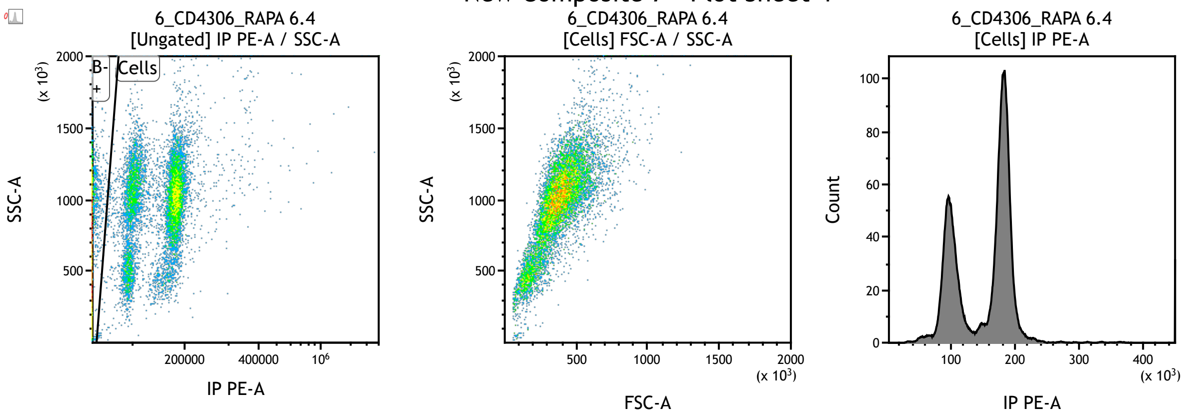

New Composite 7 - Plot Sheet 5

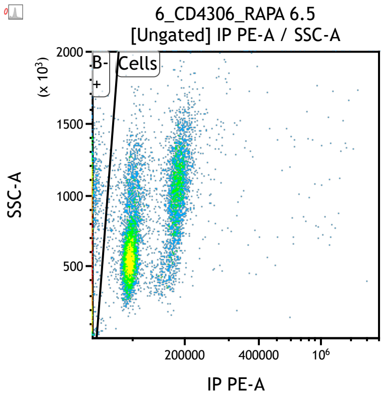

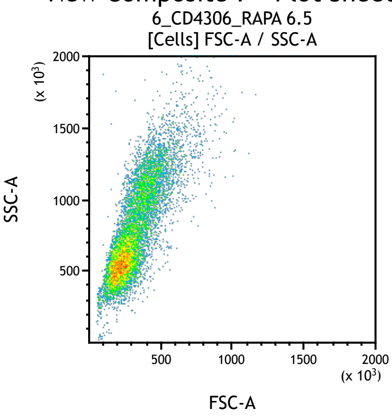

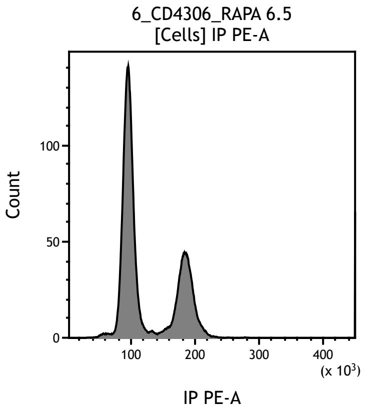

New Composite 7 - Plot Sheet 6

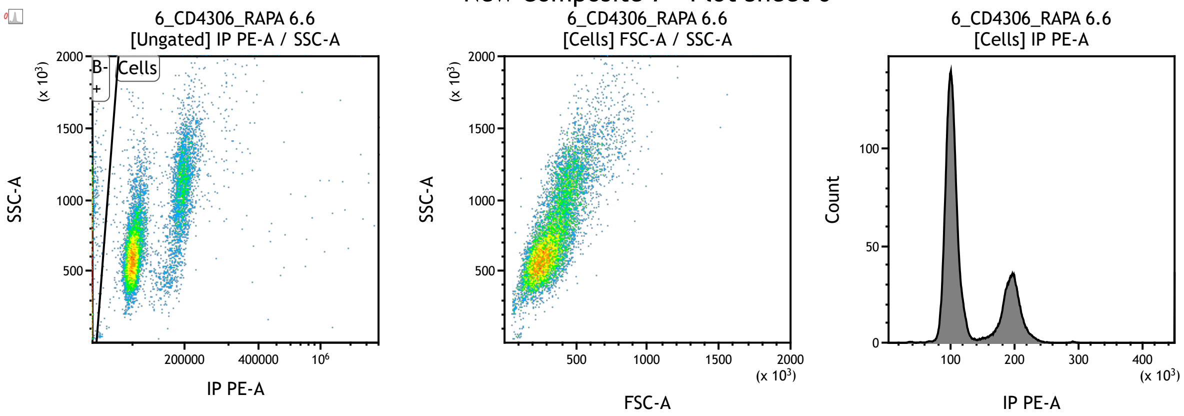

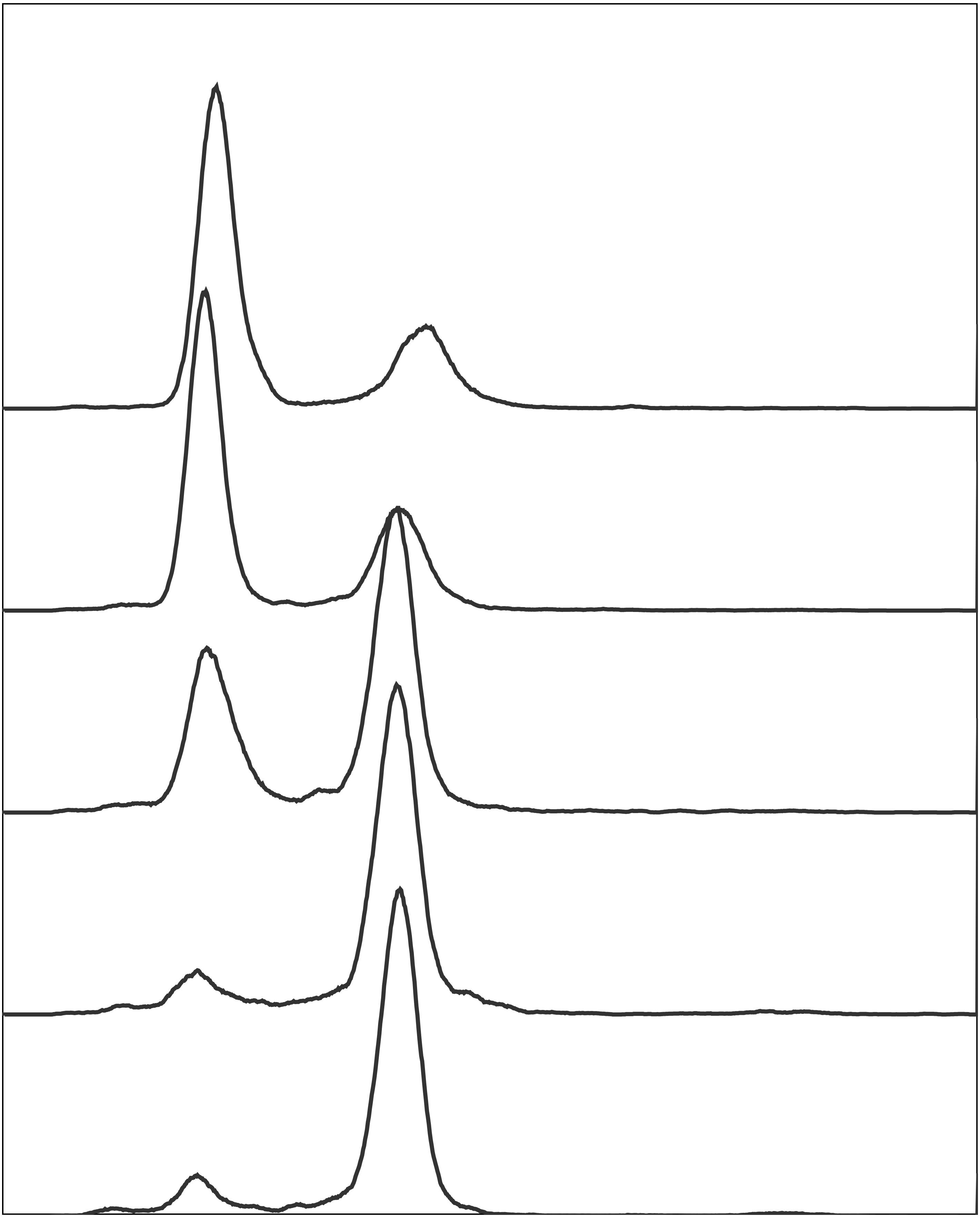

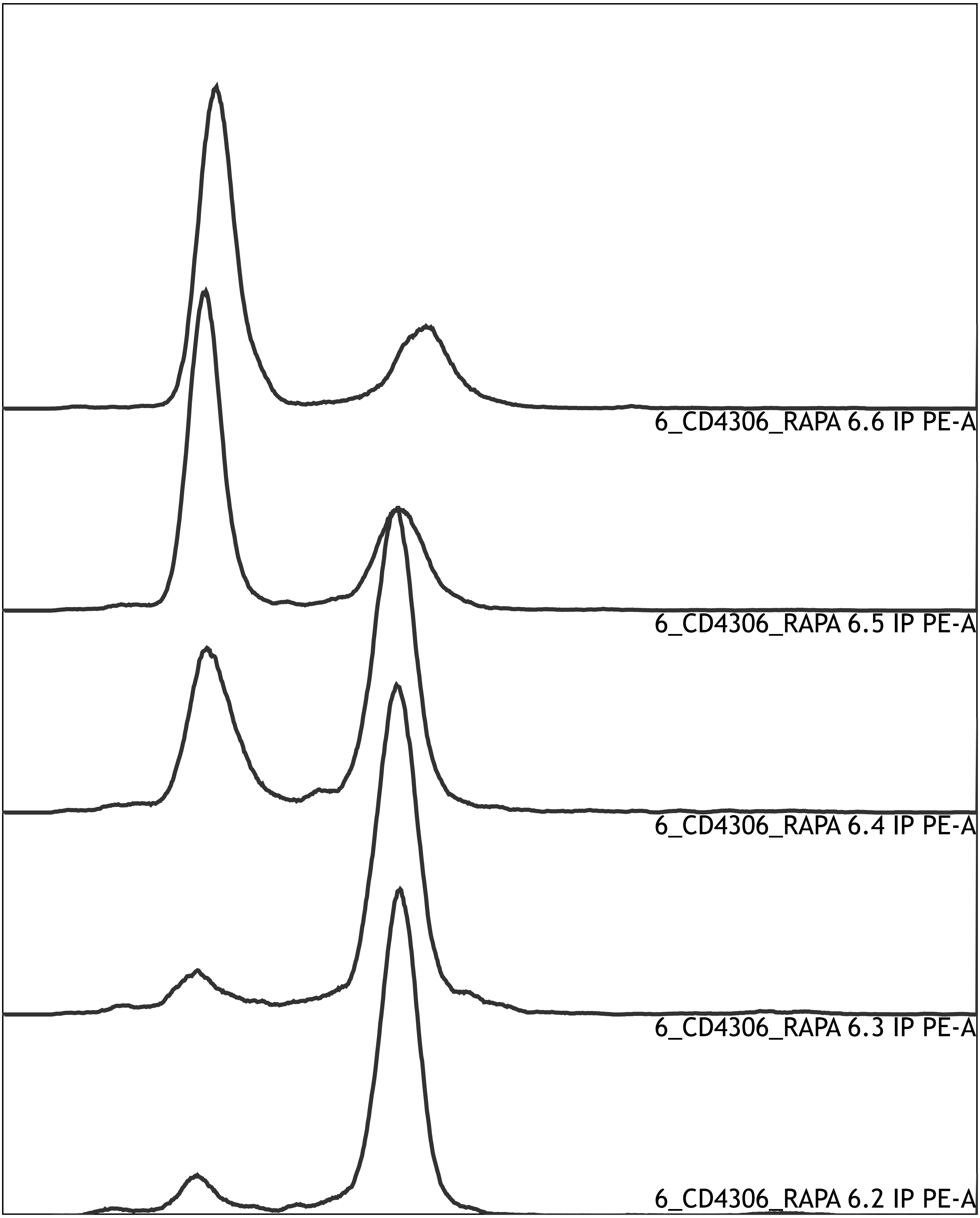

6_CD4306_RAPA 6.6 IP PE-A
6_CD4306_RAPA 6.5 IP PE-A
6_CD4306_RAPA 6.4 IP PE-A
6_CD4306_RAPA 6.3 IP PE-A
6_CD4306_RAPA 6.2 IP PE-A
