# Supplementary material for: TOR complex 1 negatively regulates NDR kinase Cbk1 to control cell separation in budding yeast
Source: PLoS Biol. 2023 Aug 30;21(8):e3002263. doi: 10.1371/journal.pbio.3002263 (PMC10468069; doi:10.1371/journal.pbio.3002263)
Supplement: S1 File — (ZIP) [file pbio.3002263.s024.zip › S5B.pdf]

New Composite 5 - Plot Sheet 3

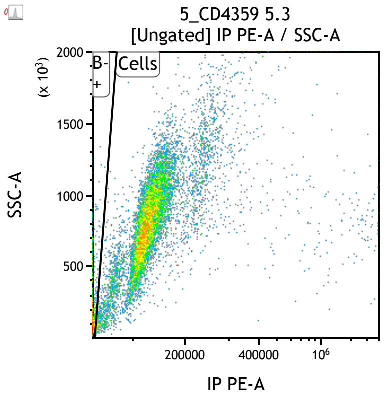

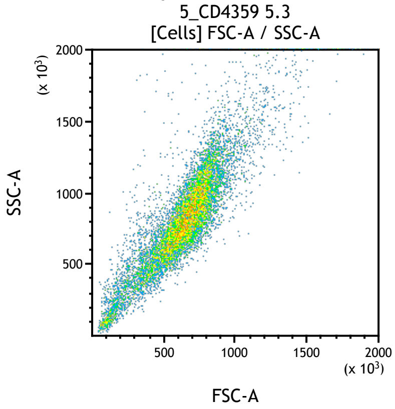

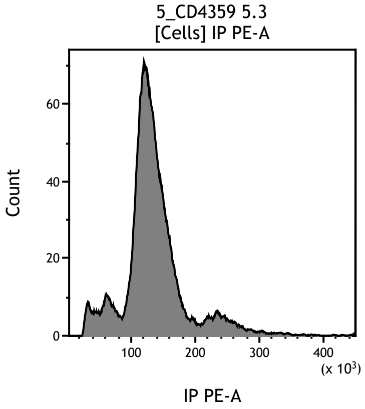

New Composite 5 - Plot Sheet 4

5_CD4359 5.4
[Ungated] IP PE-A / SSC-A

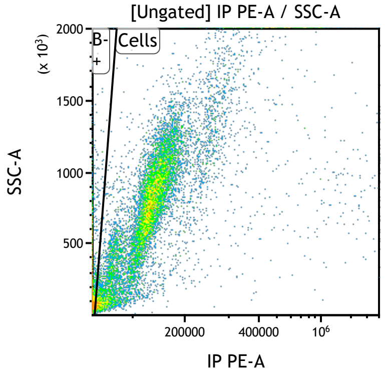

5_CD4359 5.4
[Cells] FSC-A / SSC-A

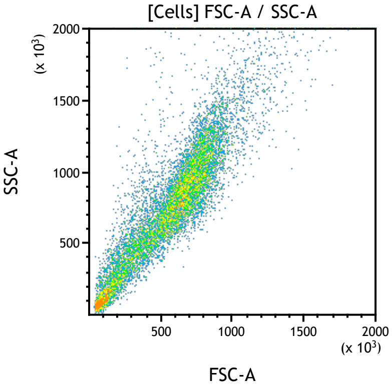

5_CD4359 5.4
[Cells] IP PE-A

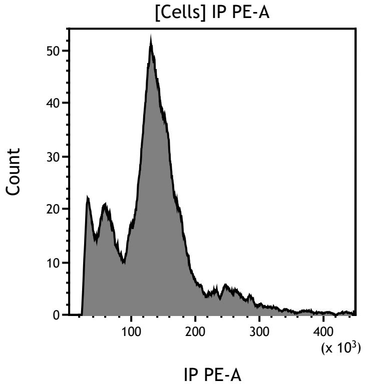

New Composite 5 - Plot Sheet 5

5_CD4359 5.5
[Ungated] IP PE-A / SSC-A

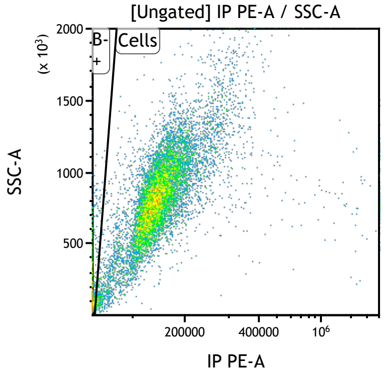

5_CD4359 5.5
[Cells] FSC-A / SSC-A

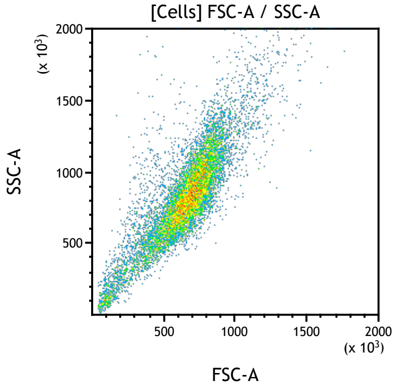

5_CD4359 5.5
[Cells] IP PE-A

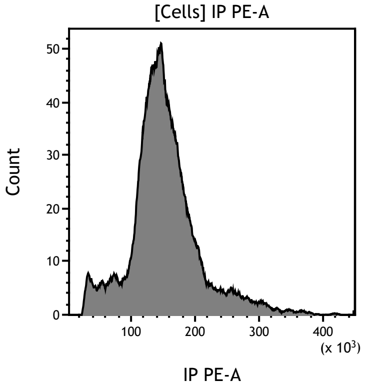

New Composite 5 - Plot Sheet 6

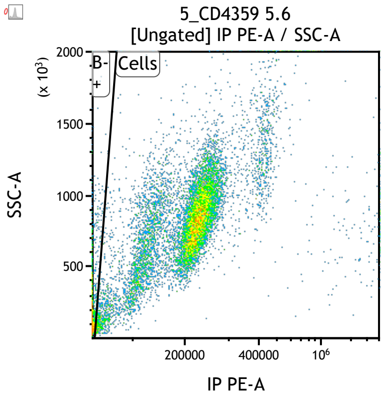

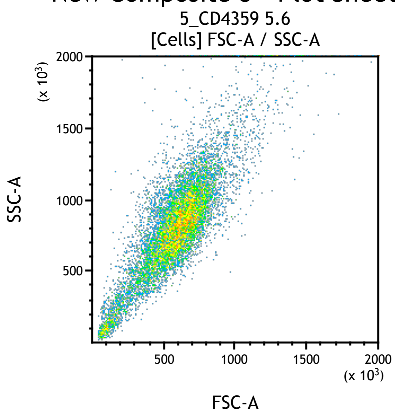

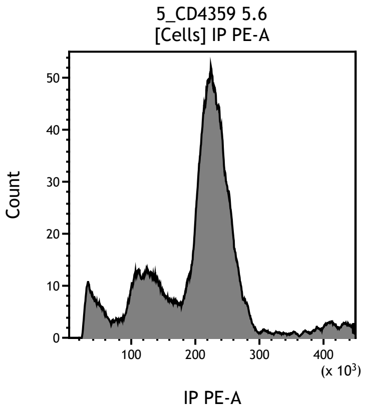

New Composite 5 - Plot Sheet 7

5_CD4359 5.7
[Ungated] IP PE-A / SSC-A

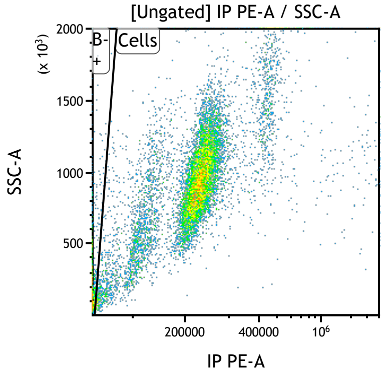

5_CD4359 5.7
[Cells] FSC-A / SSC-A

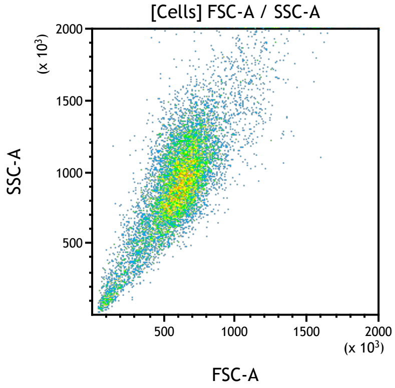

5_CD4359 5.7
[Cells] IP PE-A

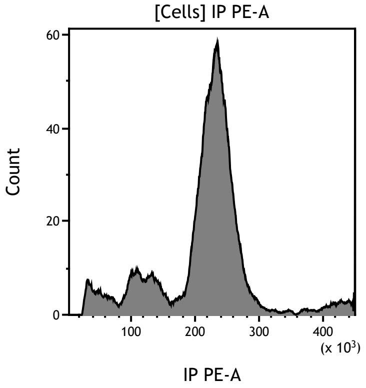

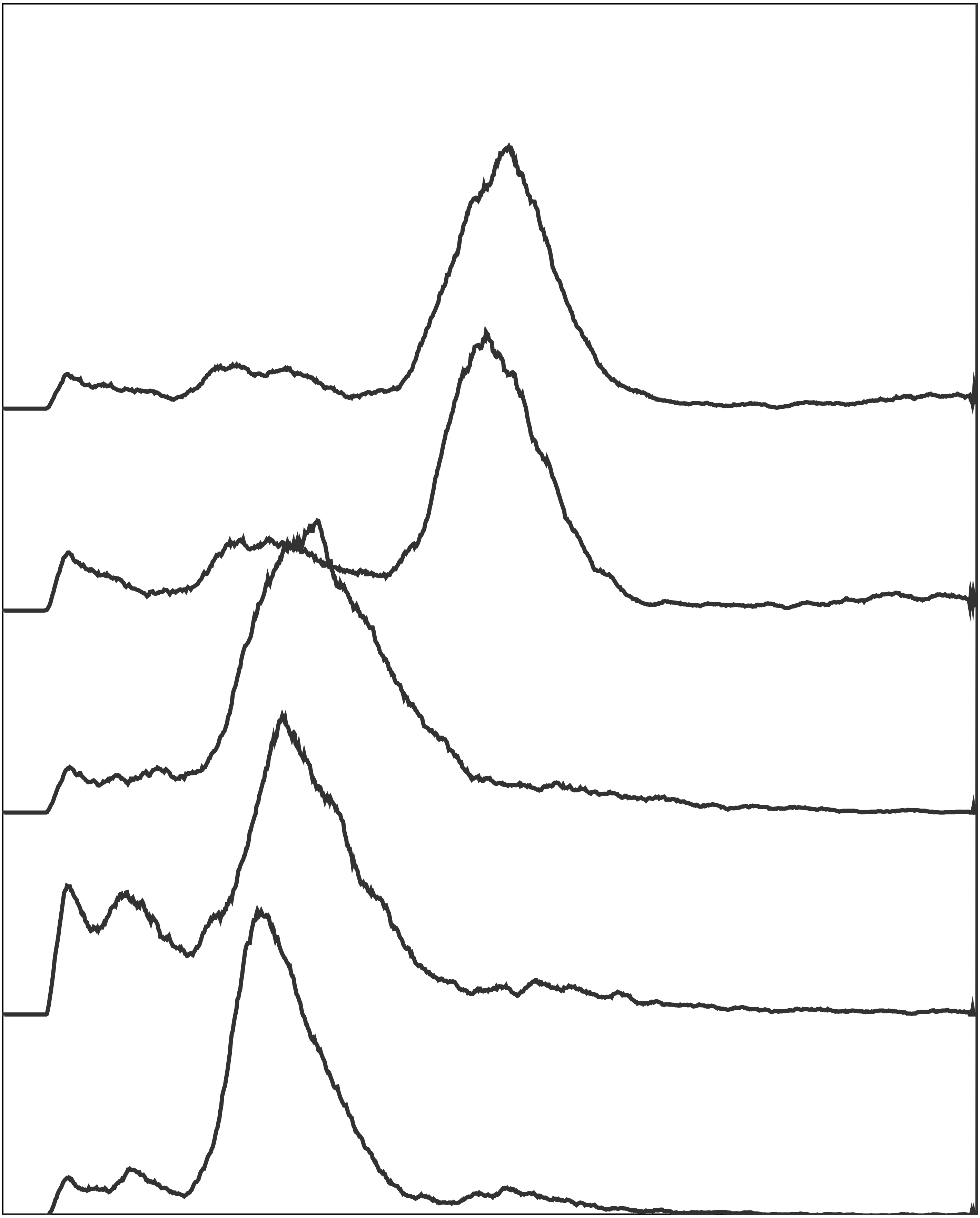

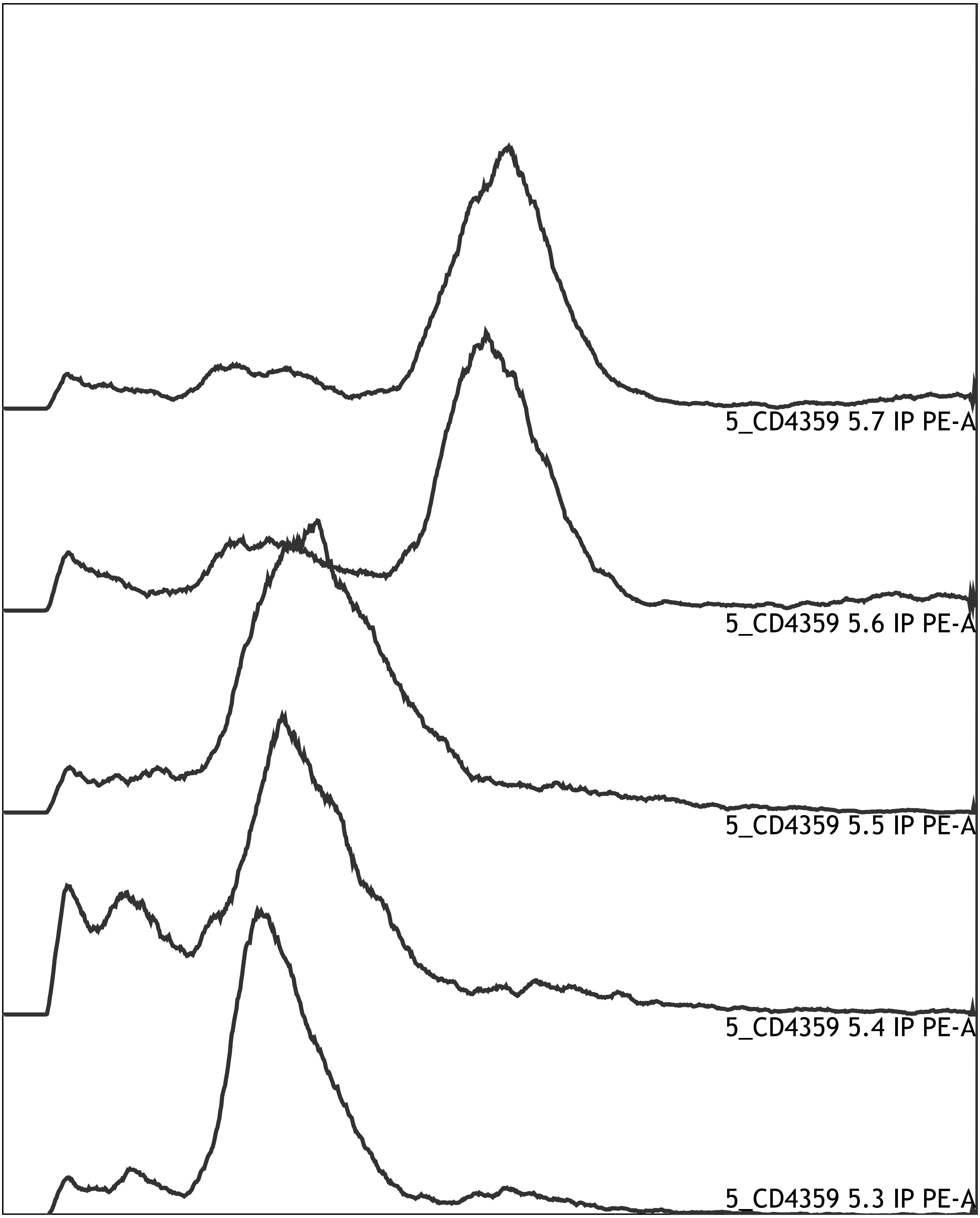
5_CD4359 5.7 IP PE-A
5_CD4359 5.6 IP PE-A
5_CD4359 5.5 IP PE-A
5_CD4359 5.4 IP PE-A
5_CD4359 5.3 IP PE-A

New Composite 6 - Plot Sheet 3

6_CD4359_RAPA 6.3
[Ungated] IP PE-A / SSC-A

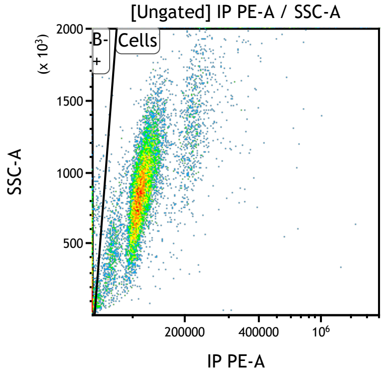

6_CD4359_RAPA 6.3
[Cells] FSC-A / SSC-A

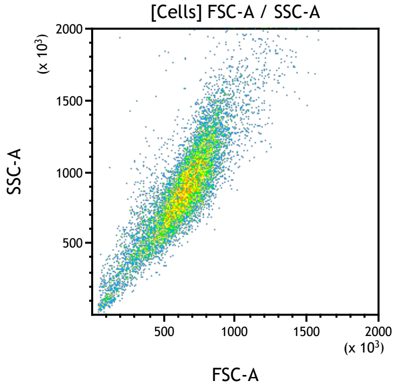

6_CD4359_RAPA 6.3
[Cells] IP PE-A

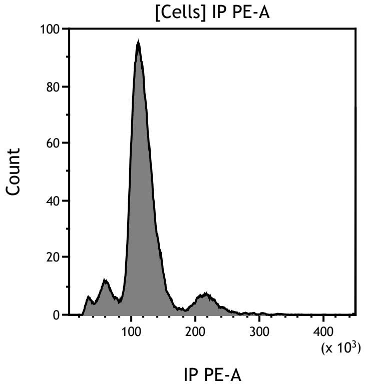

New Composite 6 - Plot Sheet 4

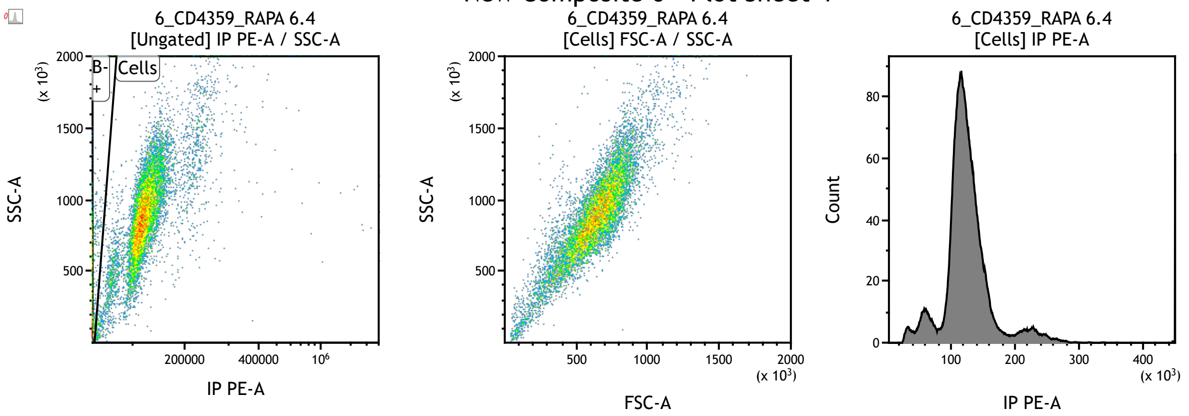

New Composite 6 - Plot Sheet 5

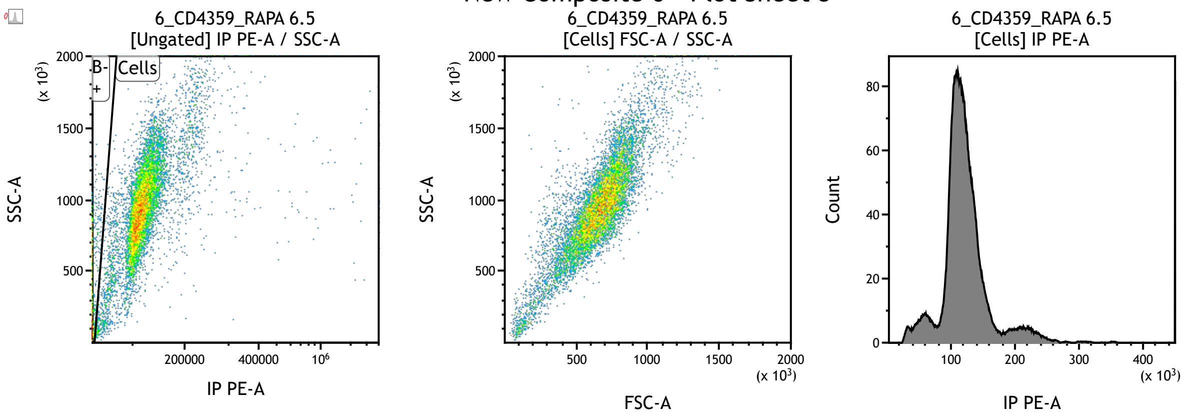

New Composite 6 - Plot Sheet 6

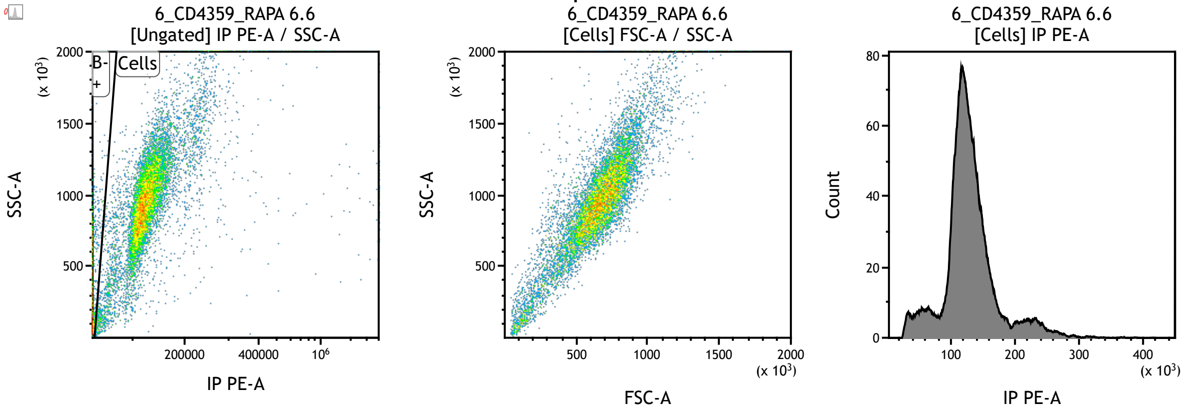

New Composite 6 - Plot Sheet 7

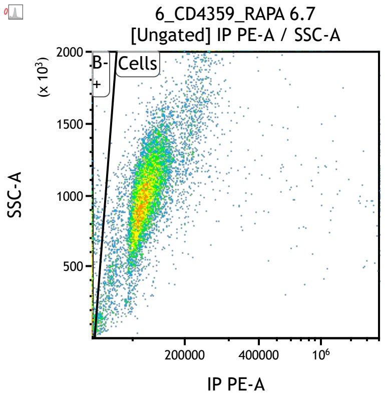

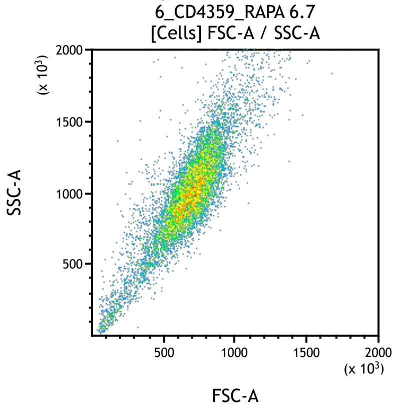

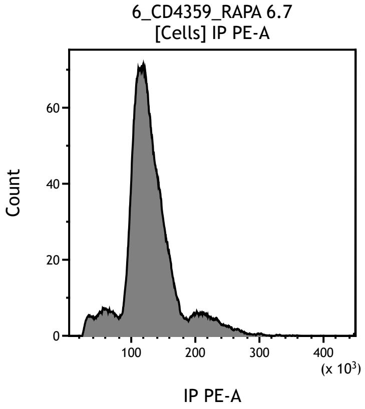

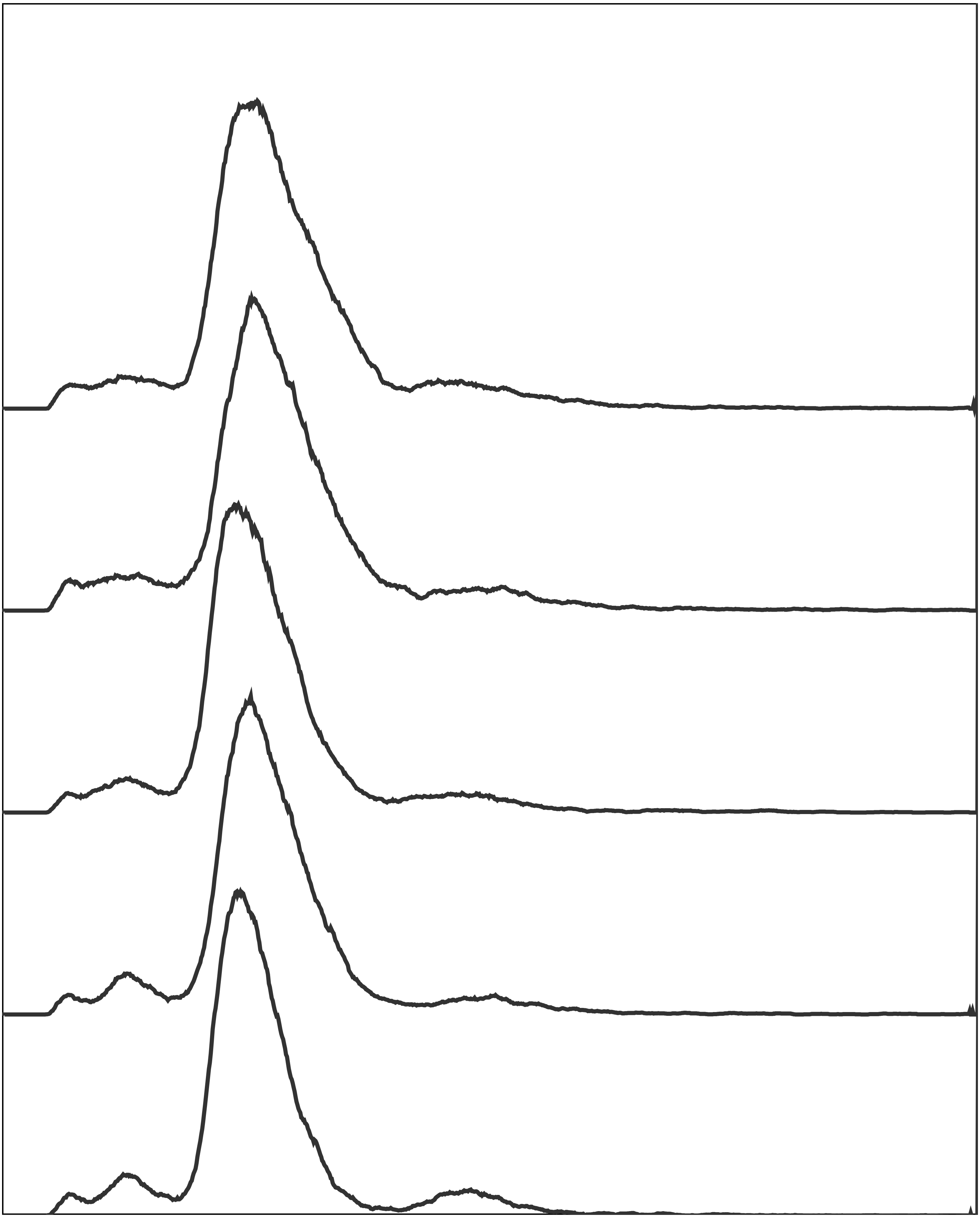

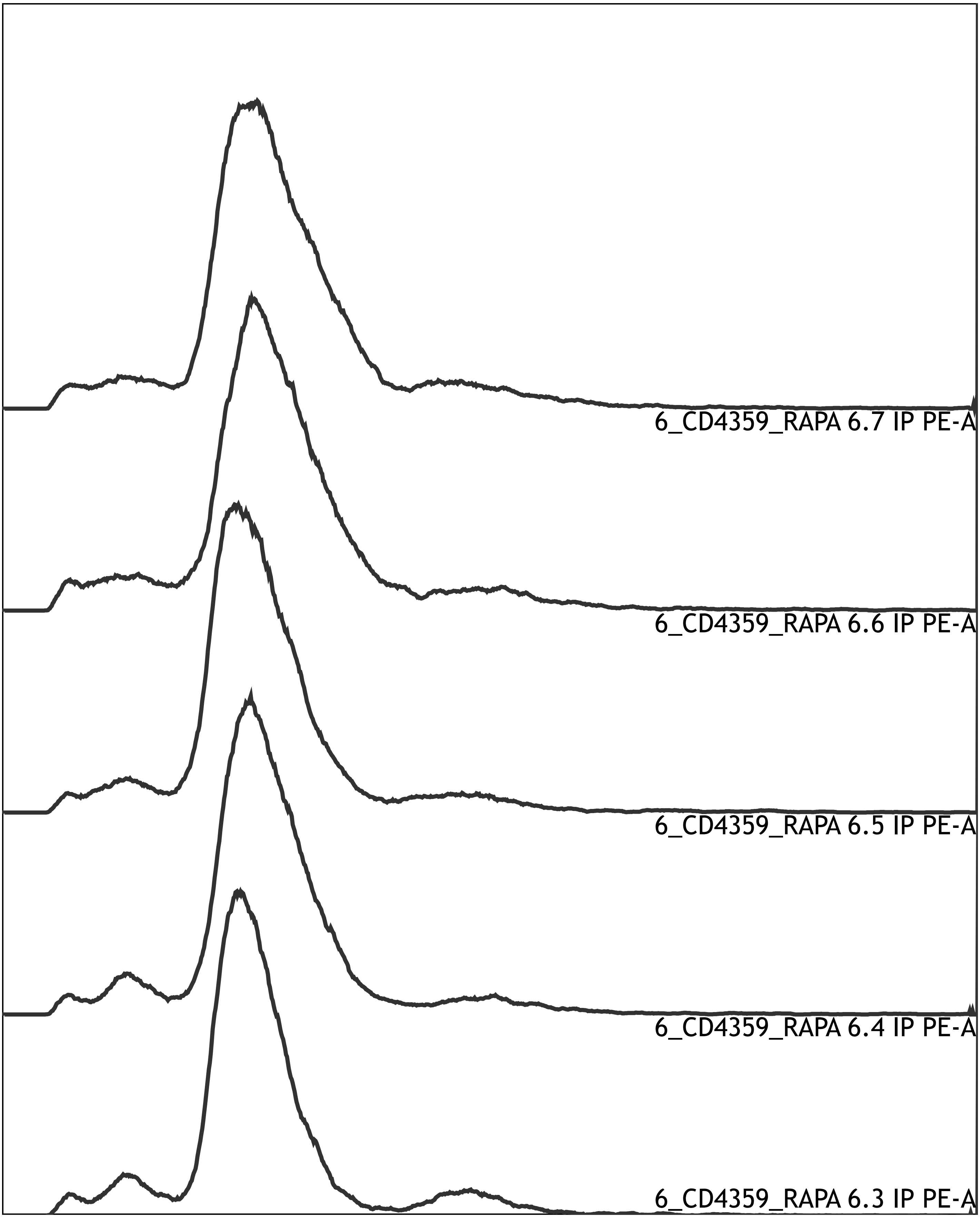
6_CD4359_RAPA 6.7 IP PE-A
6_CD4359_RAPA 6.6 IP PE-A
6_CD4359_RAPA 6.5 IP PE-A
6_CD4359_RAPA 6.4 IP PE-A
6_CD4359_RAPA 6.3 IP PE-A
