# Supplementary material for: TOR complex 1 negatively regulates NDR kinase Cbk1 to control cell separation in budding yeast
Source: PLoS Biol. 2023 Aug 30;21(8):e3002263. doi: 10.1371/journal.pbio.3002263 (PMC10468069; doi:10.1371/journal.pbio.3002263)
Supplement: S1 File — (ZIP) [file pbio.3002263.s024.zip › S5C.pdf]

New Composite 1 - Plot Sheet 3

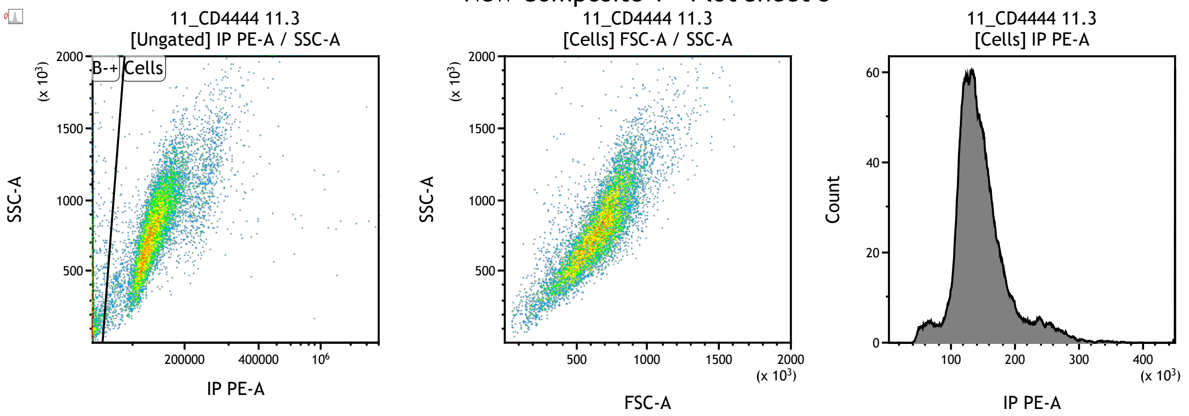

New Composite 1 - Plot Sheet 4

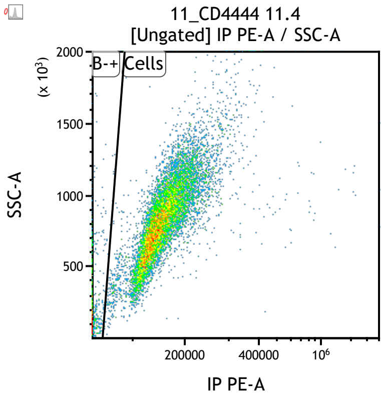

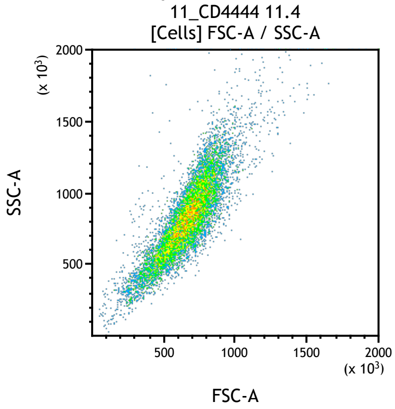

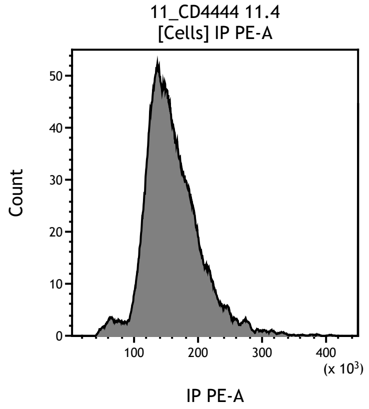

New Composite 1 - Plot Sheet 5

11_CD4444 11.5
[Ungated] IP PE-A / SSC-A

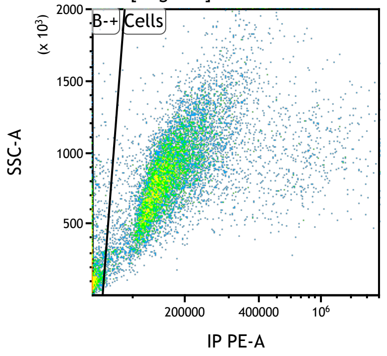

11_CD4444 11.5
[Cells] FSC-A / SSC-A

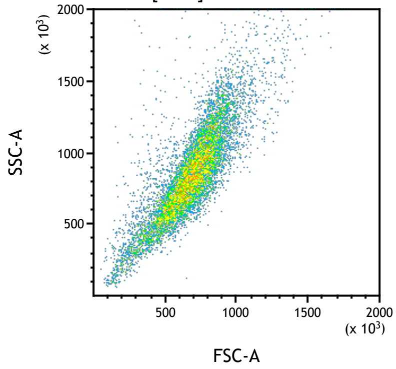

11_CD4444 11.5
[Cells] IP PE-A

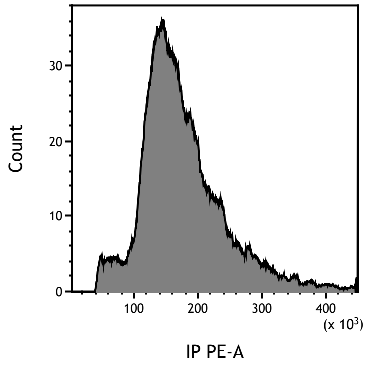

New Composite 1 - Plot Sheet 6

11_CD4444 11.6
[Ungated] IP PE-A / SSC-A

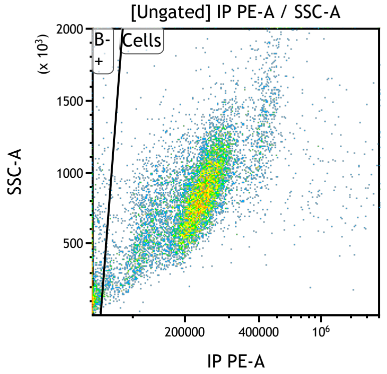

11_CD4444 11.6
[Cells] FSC-A / SSC-A

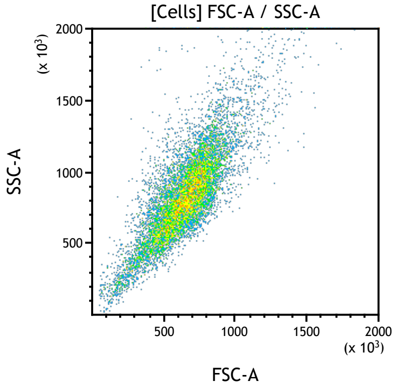

11_CD4444 11.6
[Cells] IP PE-A

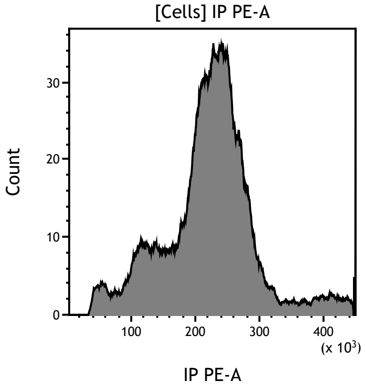

New Composite 1 - Plot Sheet 7

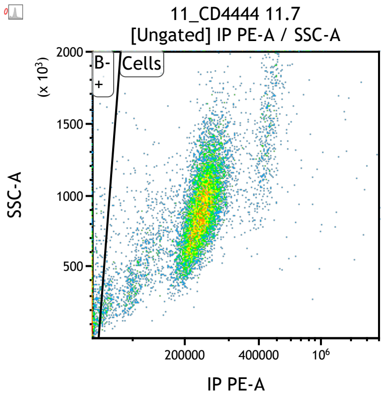

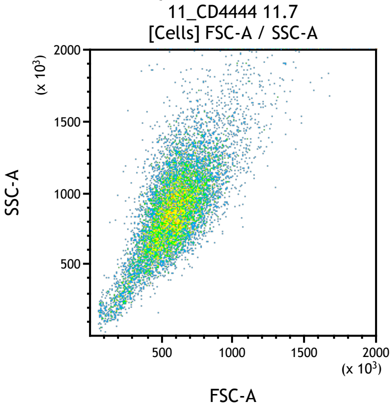

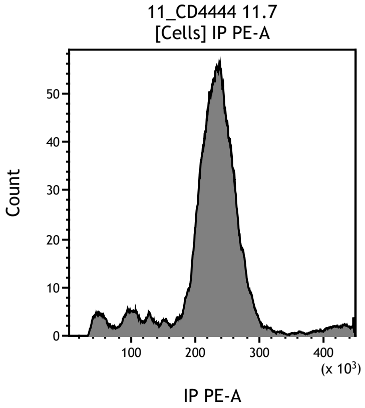

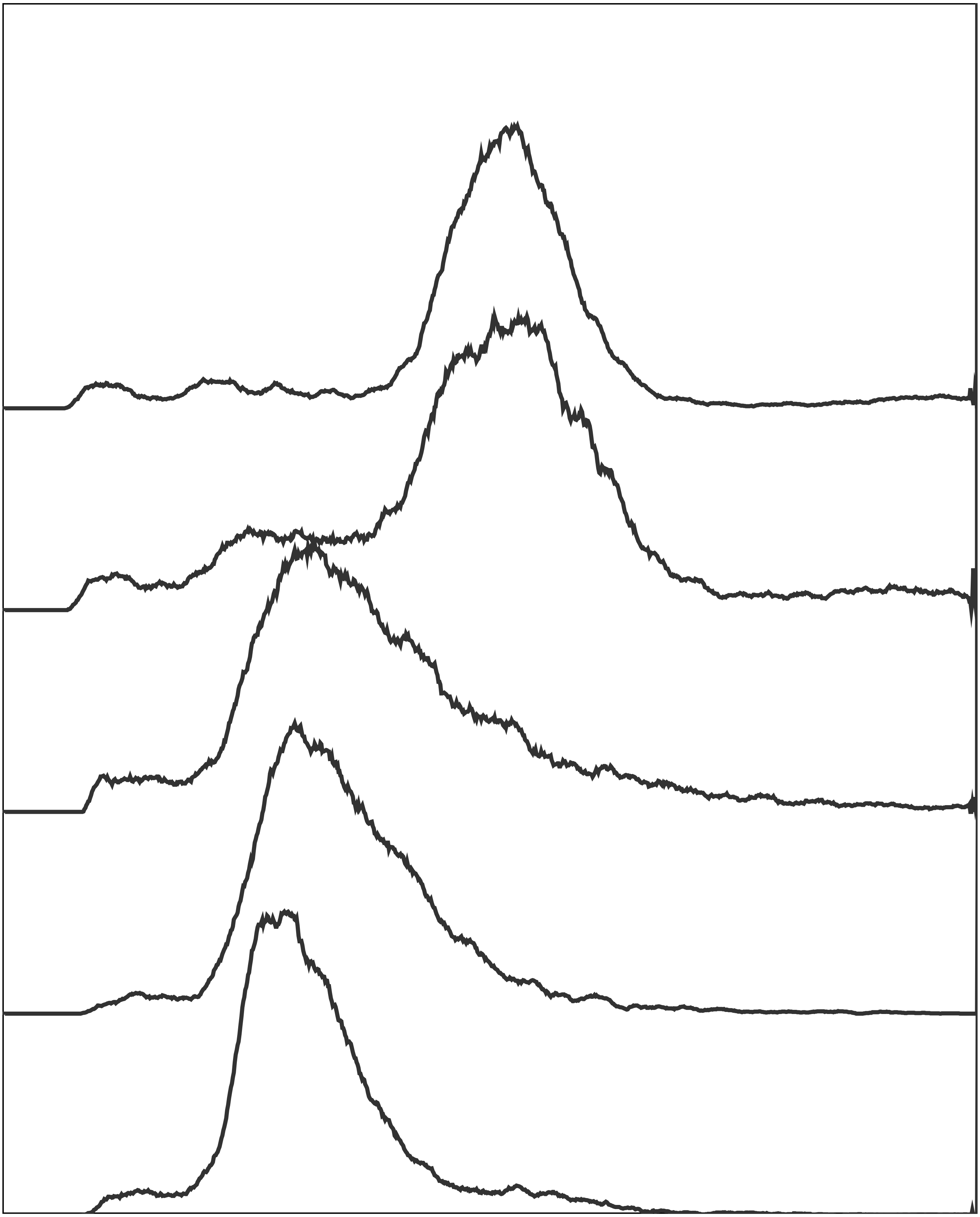

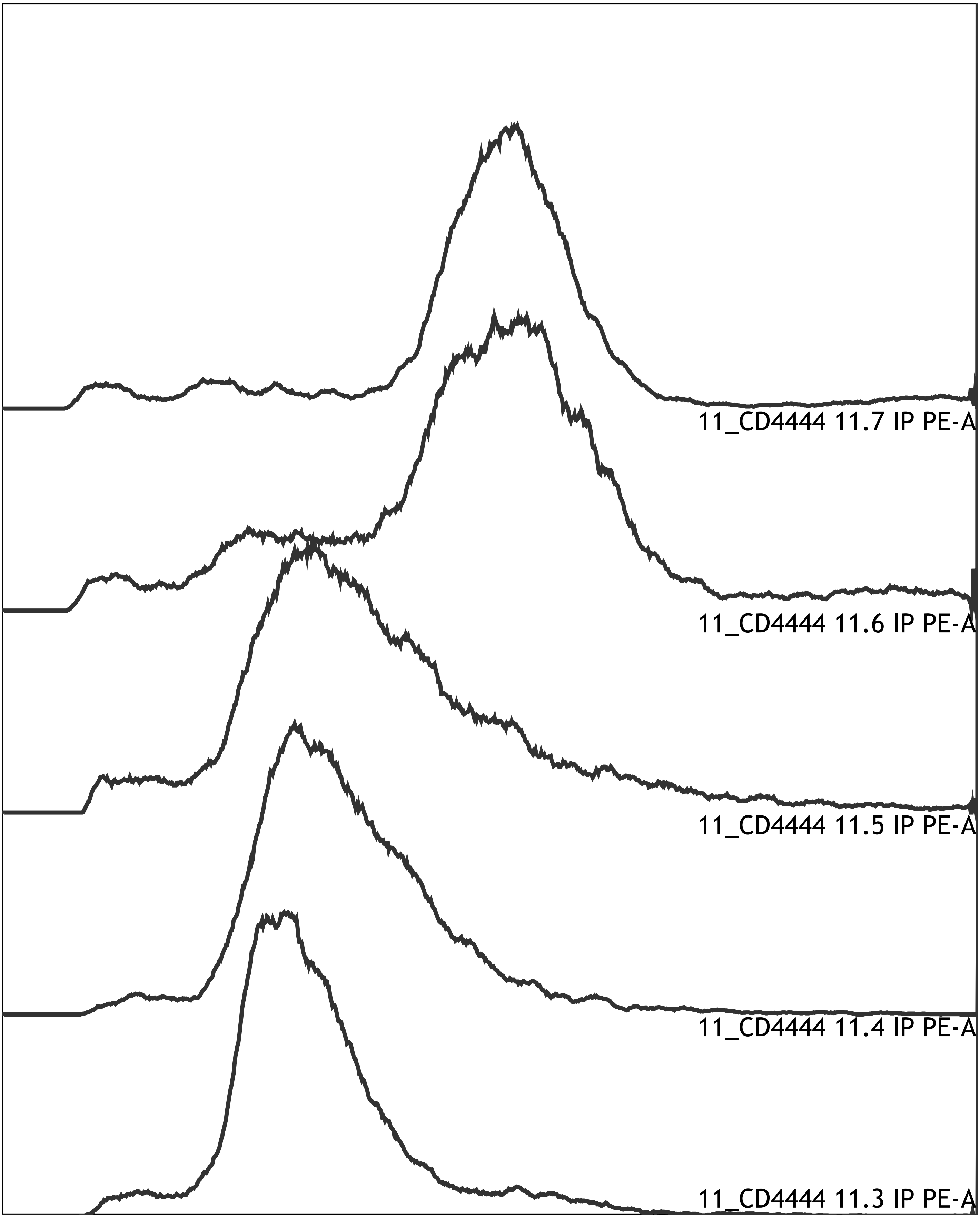
11_CD4444 11.7 IP PE-A
11_CD4444 11.6 IP PE-A
11_CD4444 11.5 IP PE-A
11_CD4444 11.4 IP PE-A
11_CD4444 11.3 IP PE-A

New Composite 3 - Plot Sheet 3

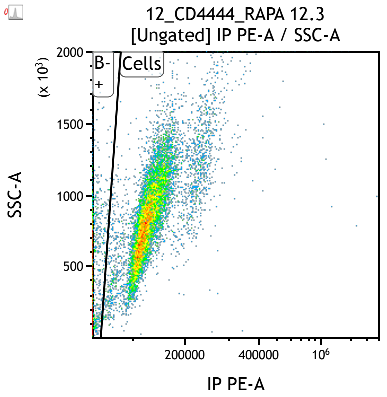

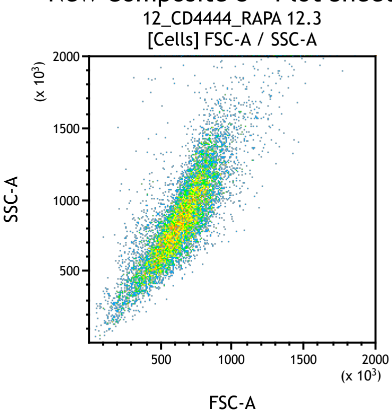

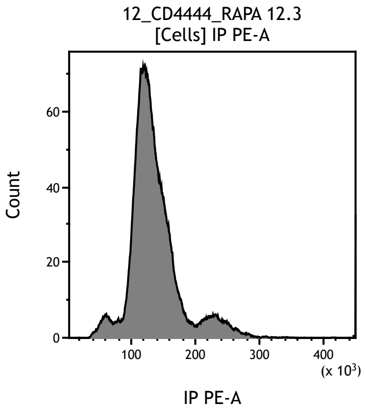

New Composite 3 - Plot Sheet 4

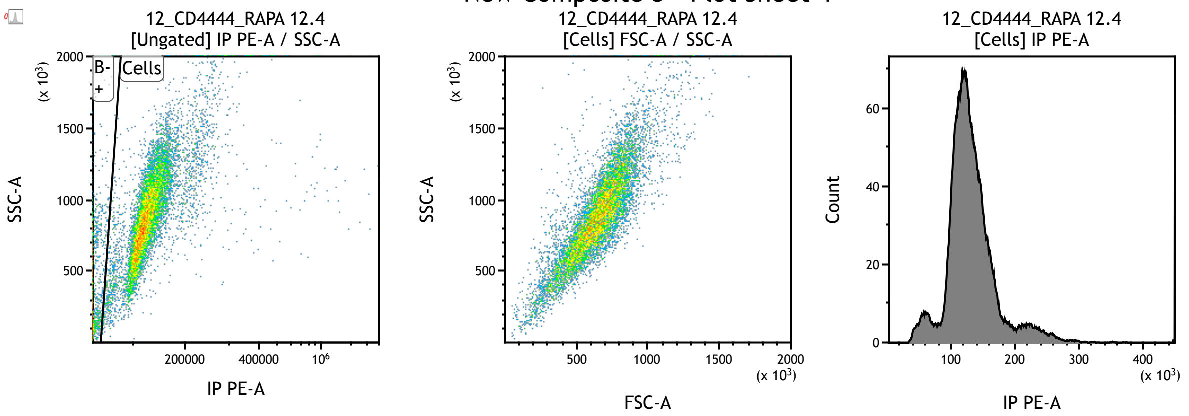

New Composite 3 - Plot Sheet 5

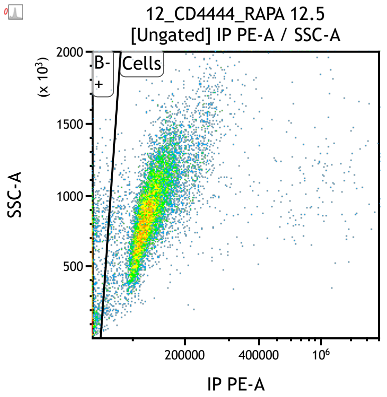

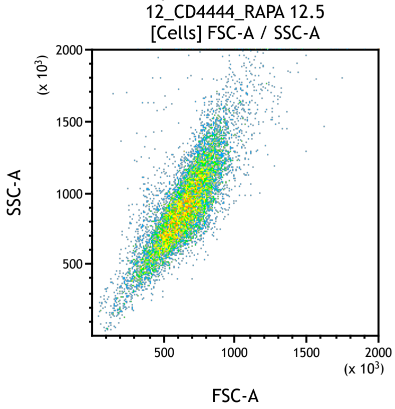

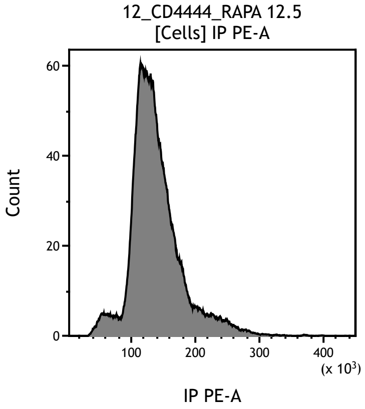

New Composite 3 - Plot Sheet 6

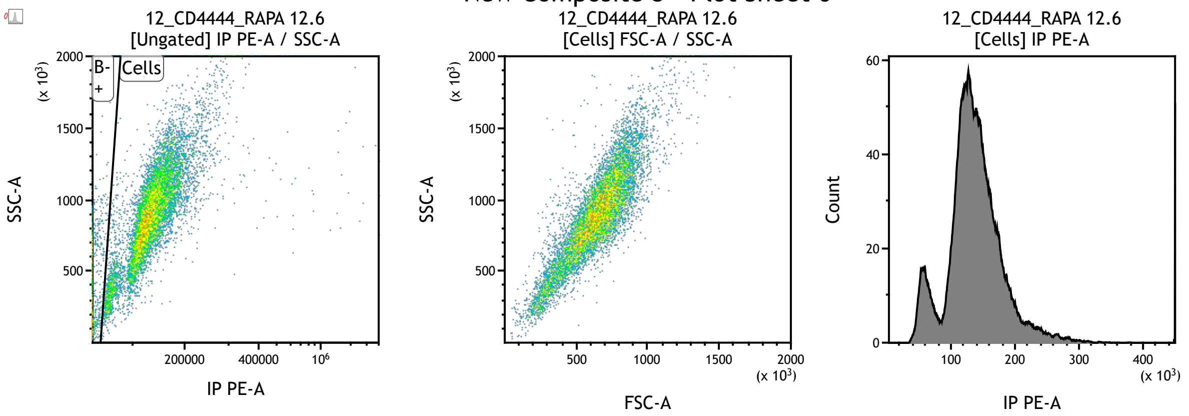

New Composite 3 - Plot Sheet 7

12_CD4444_RAPA 12.7
[Ungated] IP PE-A / SSC-A

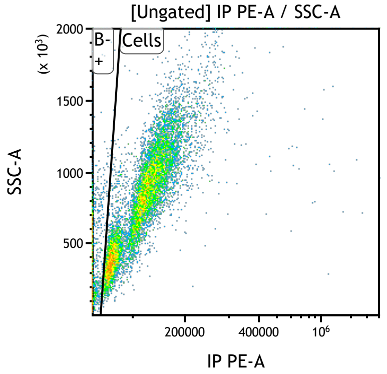

12_CD4444_RAPA 12.7
[Cells] FSC-A / SSC-A

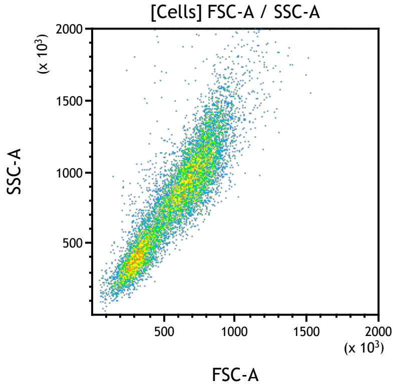

12_CD4444_RAPA 12.7
[Cells] IP PE-A

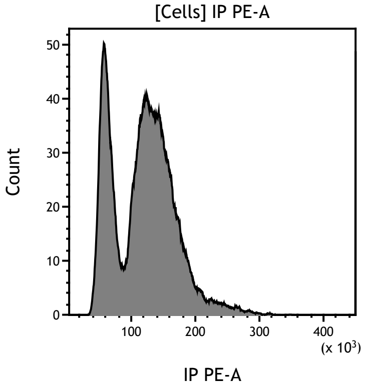

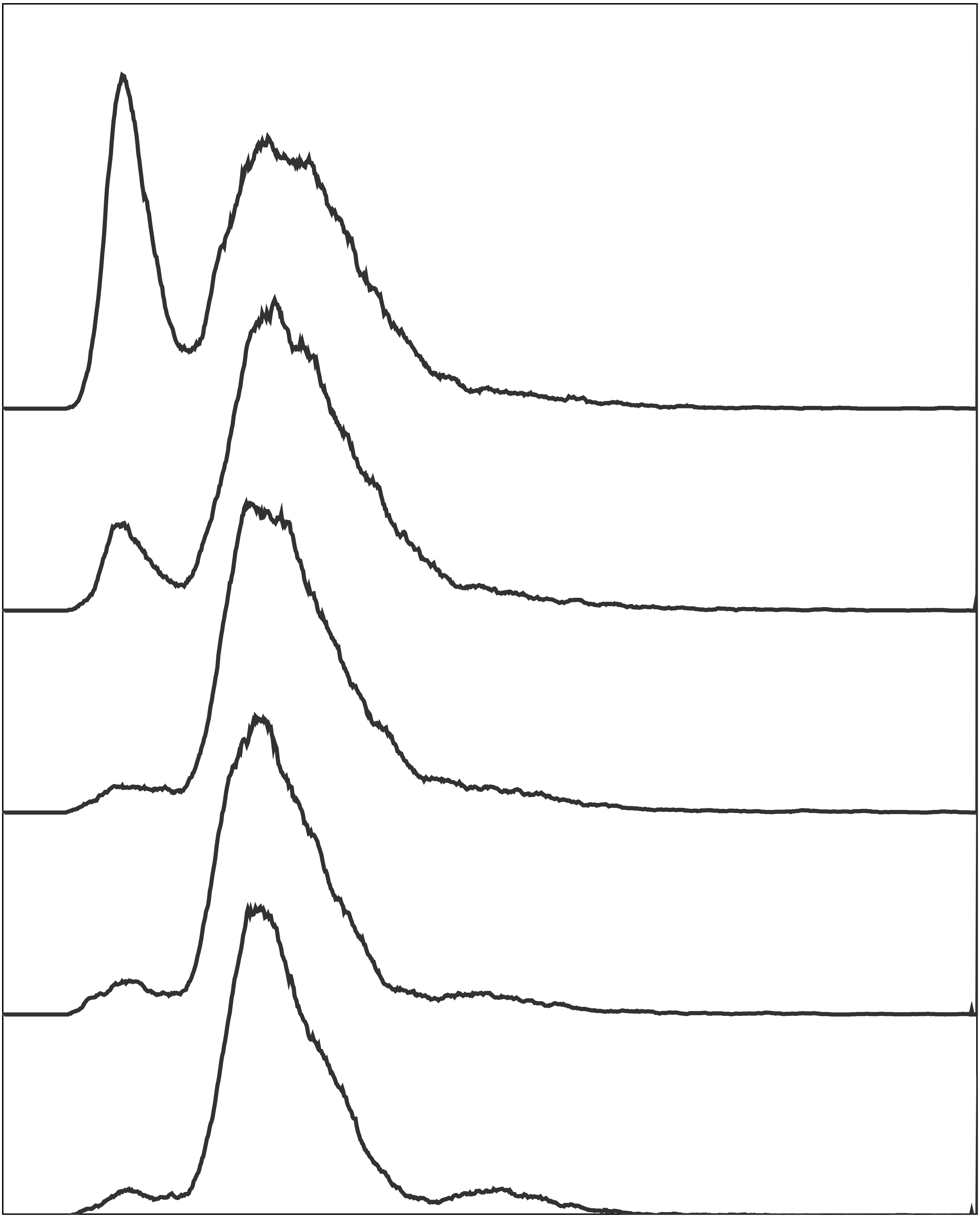

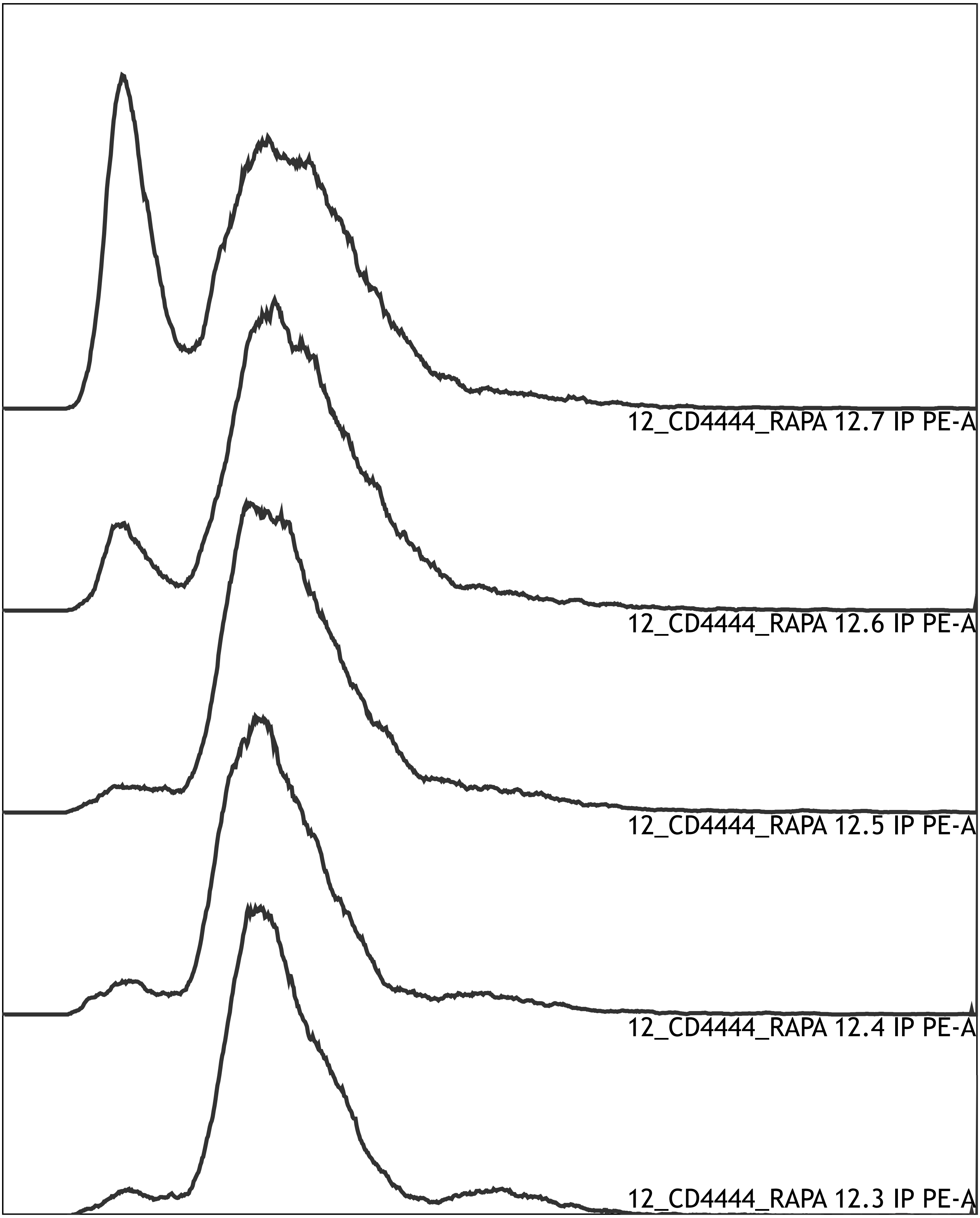
12_CD4444_RAPA 12.7 IP PE-A
12_CD4444_RAPA 12.6 IP PE-A
12_CD4444_RAPA 12.5 IP PE-A
12_CD4444_RAPA 12.4 IP PE-A
12_CD4444_RAPA 12.3 IP PE-A
