# Supplementary material for: TOR complex 1 negatively regulates NDR kinase Cbk1 to control cell separation in budding yeast
Source: PLoS Biol. 2023 Aug 30;21(8):e3002263. doi: 10.1371/journal.pbio.3002263 (PMC10468069; doi:10.1371/journal.pbio.3002263)
Supplement: S1 File — (ZIP) [file pbio.3002263.s024.zip › S6A.pdf]

New Composite 1 - Plot Sheet 2

1_CD4317 1.2
[Ungated] IP PE-A / SSC-A

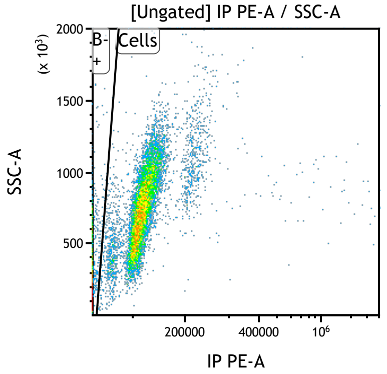

1_CD4317 1.2
[Cells] FSC-A / SSC-A

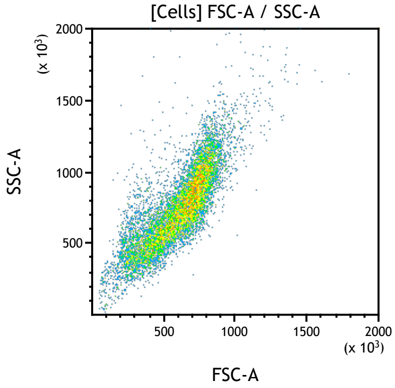

1_CD4317 1.2
[Cells] IP PE-A

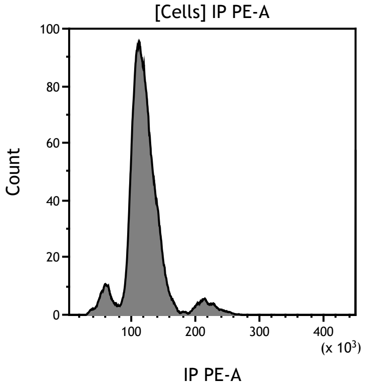

New Composite 1 - Plot Sheet 3

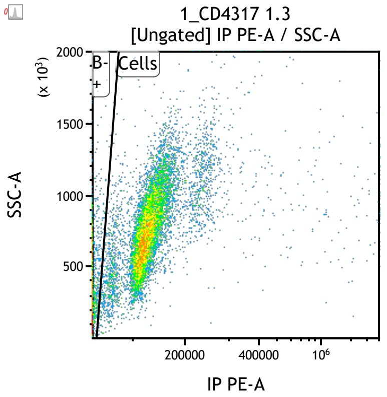

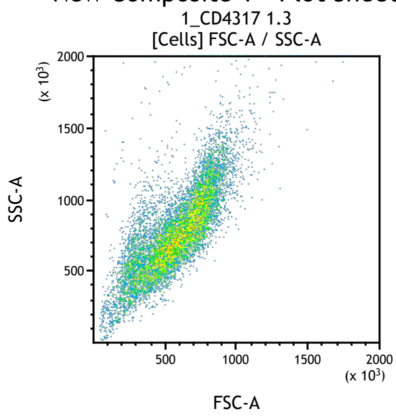

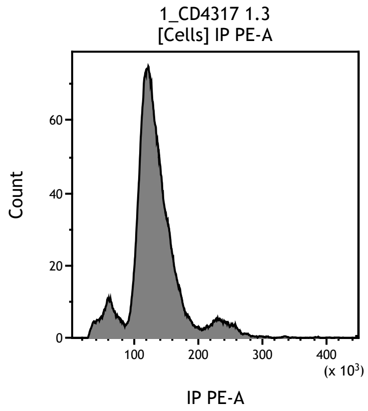

New Composite 1 - Plot Sheet 4

1_CD4317 1.4
[Ungated] IP PE-A / SSC-A

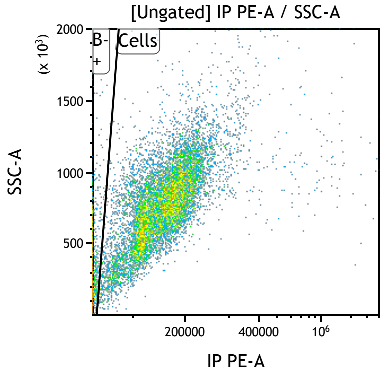

1_CD4317 1.4
[Cells] FSC-A / SSC-A

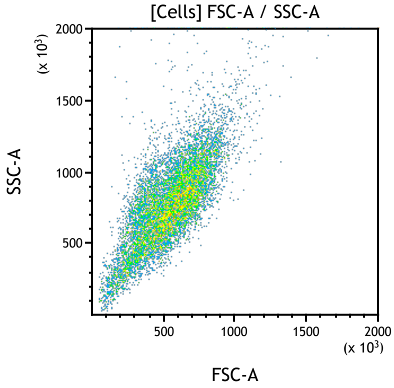

1_CD4317 1.4
[Cells] IP PE-A

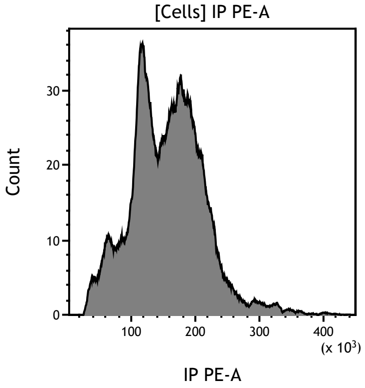

New Composite 1 - Plot Sheet 5

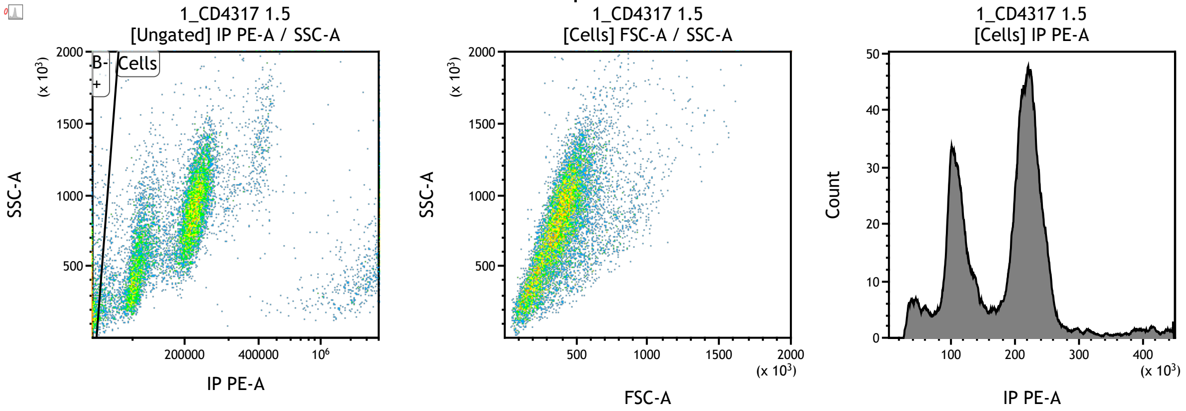

New Composite 1 - Plot Sheet 6

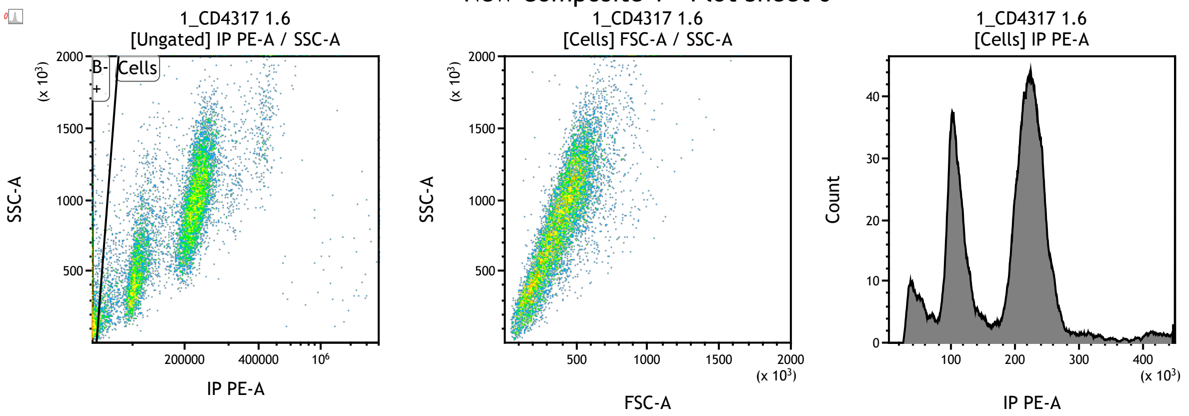

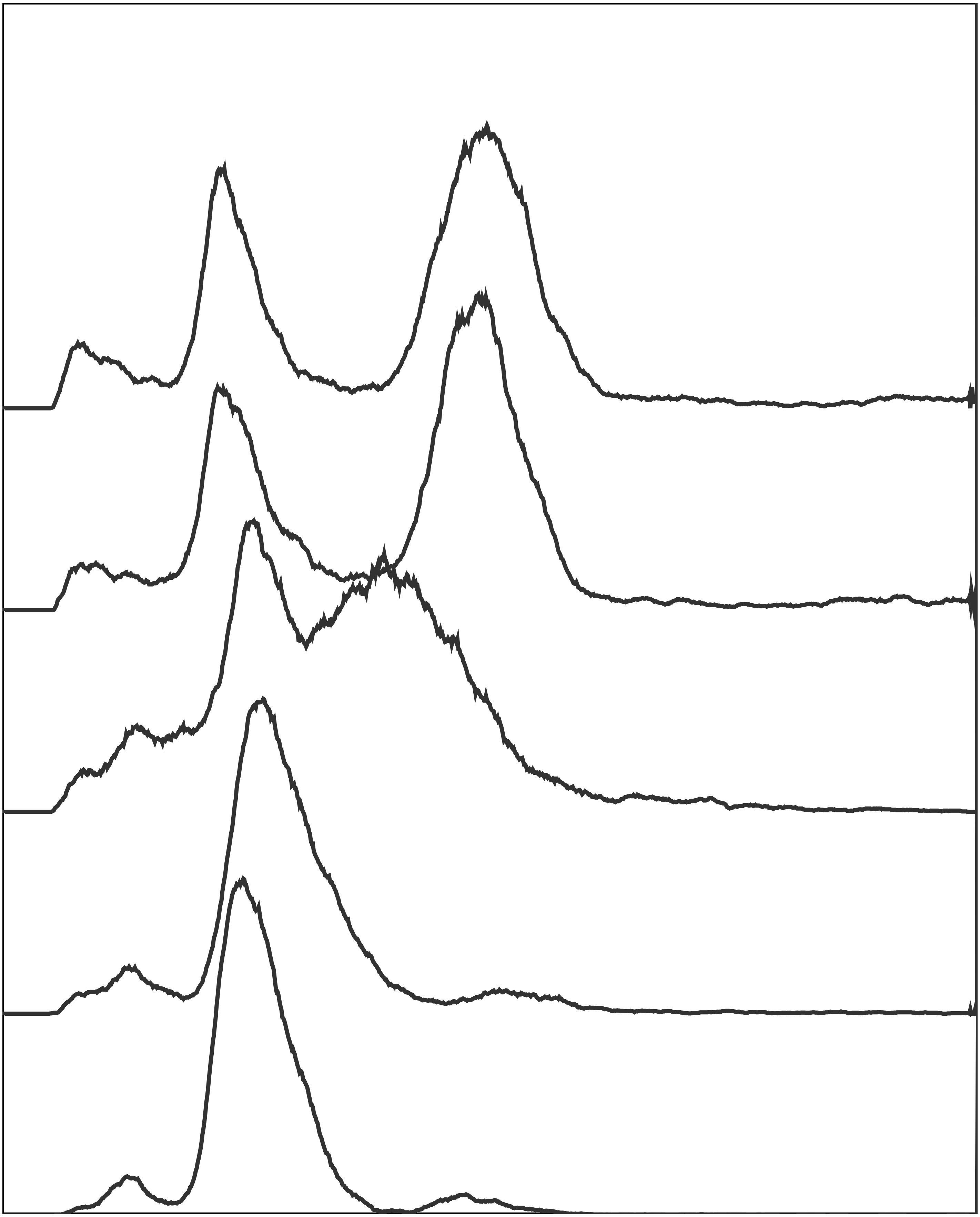

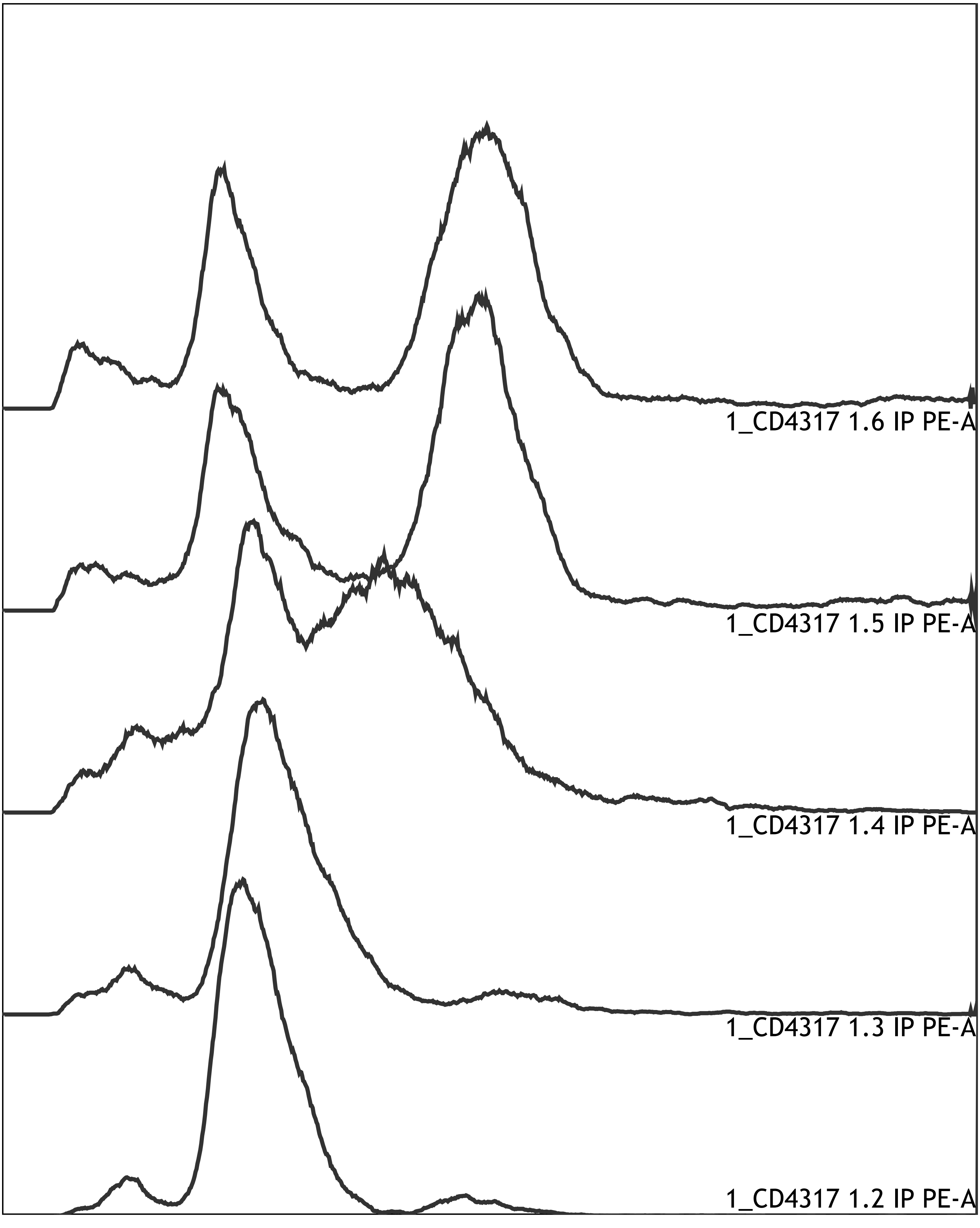
1_CD4317 1.6 IP PE-A
1_CD4317 1.5 IP PE-A
1_CD4317 1.4 IP PE-A
1_CD4317 1.3 IP PE-A
1_CD4317 1.2 IP PE-A

New Composite 3 - Plot Sheet 2

2_CD4317_RAPA 2.2
[Ungated] IP PE-A / SSC-A

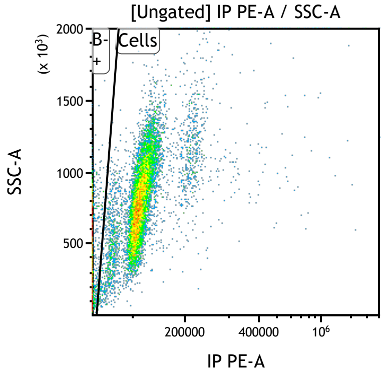

2_CD4317_RAPA 2.2
[Cells] FSC-A / SSC-A

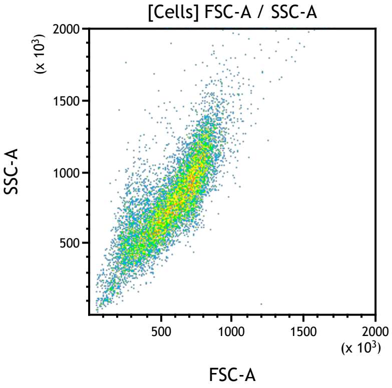

2_CD4317_RAPA 2.2
[Cells] IP PE-A

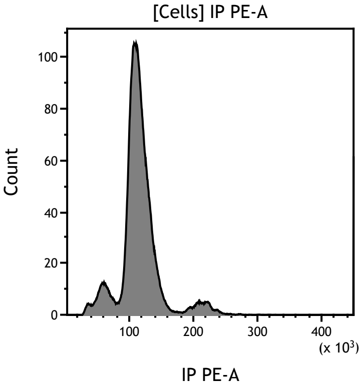

New Composite 3 - Plot Sheet 3

2_CD4317_RAPA 2.3
[Ungated] IP PE-A / SSC-A

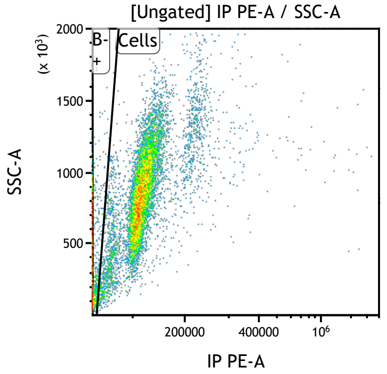

2_CD4317_RAPA 2.3
[Cells] FSC-A / SSC-A

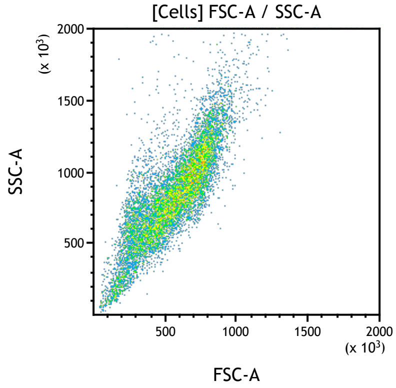

2_CD4317_RAPA 2.3
[Cells] IP PE-A

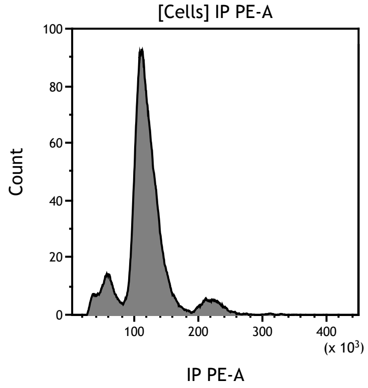

New Composite 3 - Plot Sheet 4

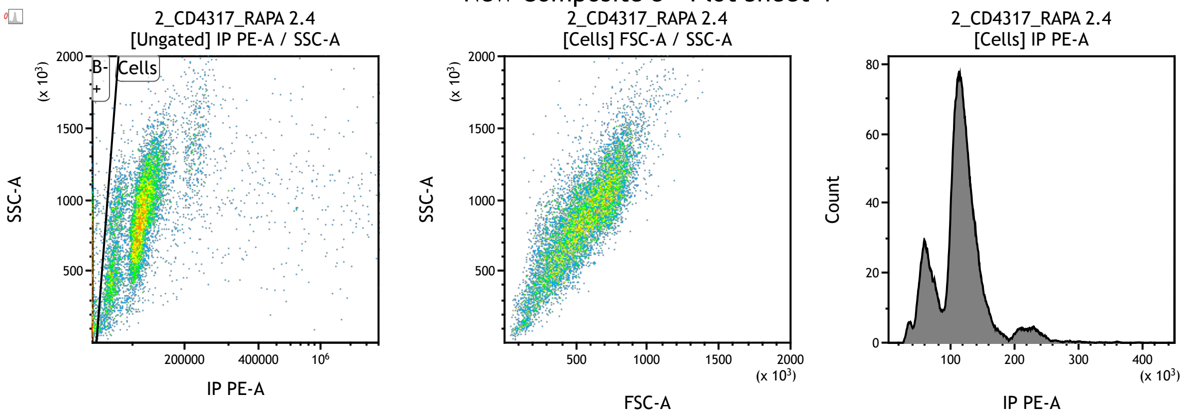

New Composite 3 - Plot Sheet 5

2_CD4317_RAPA 2.5
[Ungated] IP PE-A / SSC-A

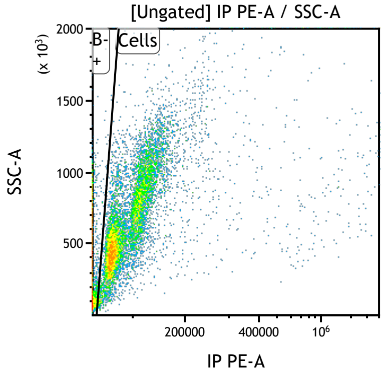

2_CD4317_RAPA 2.5
[Cells] FSC-A / SSC-A

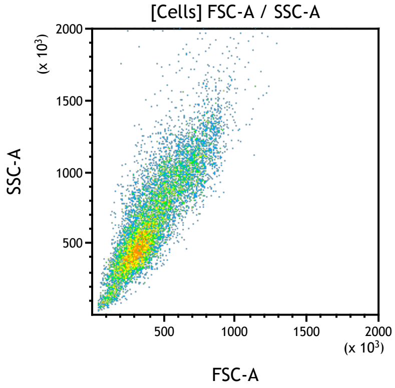

2_CD4317_RAPA 2.5
[Cells] IP PE-A

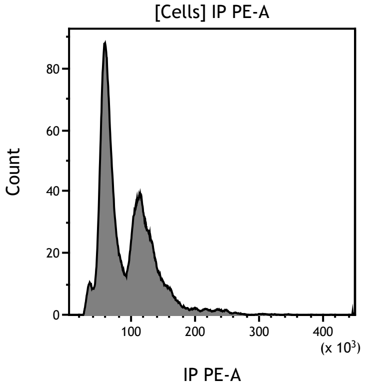

New Composite 3 - Plot Sheet 6

2_CD4317_RAPA 2.6
[Ungated] IP PE-A / SSC-A

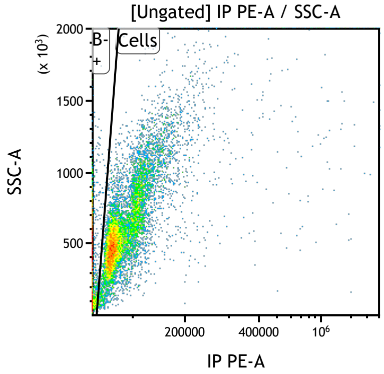

2_CD4317_RAPA 2.6
[Cells] FSC-A / SSC-A

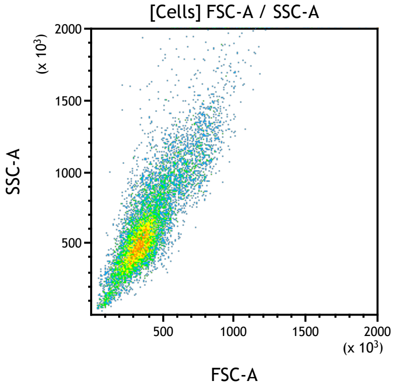

2_CD4317_RAPA 2.6
[Cells] IP PE-A

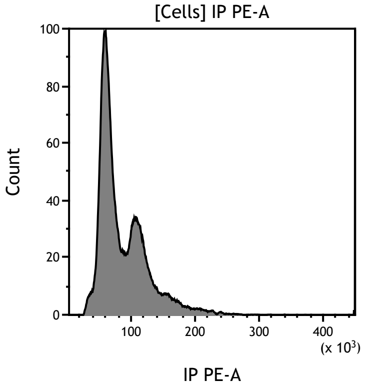

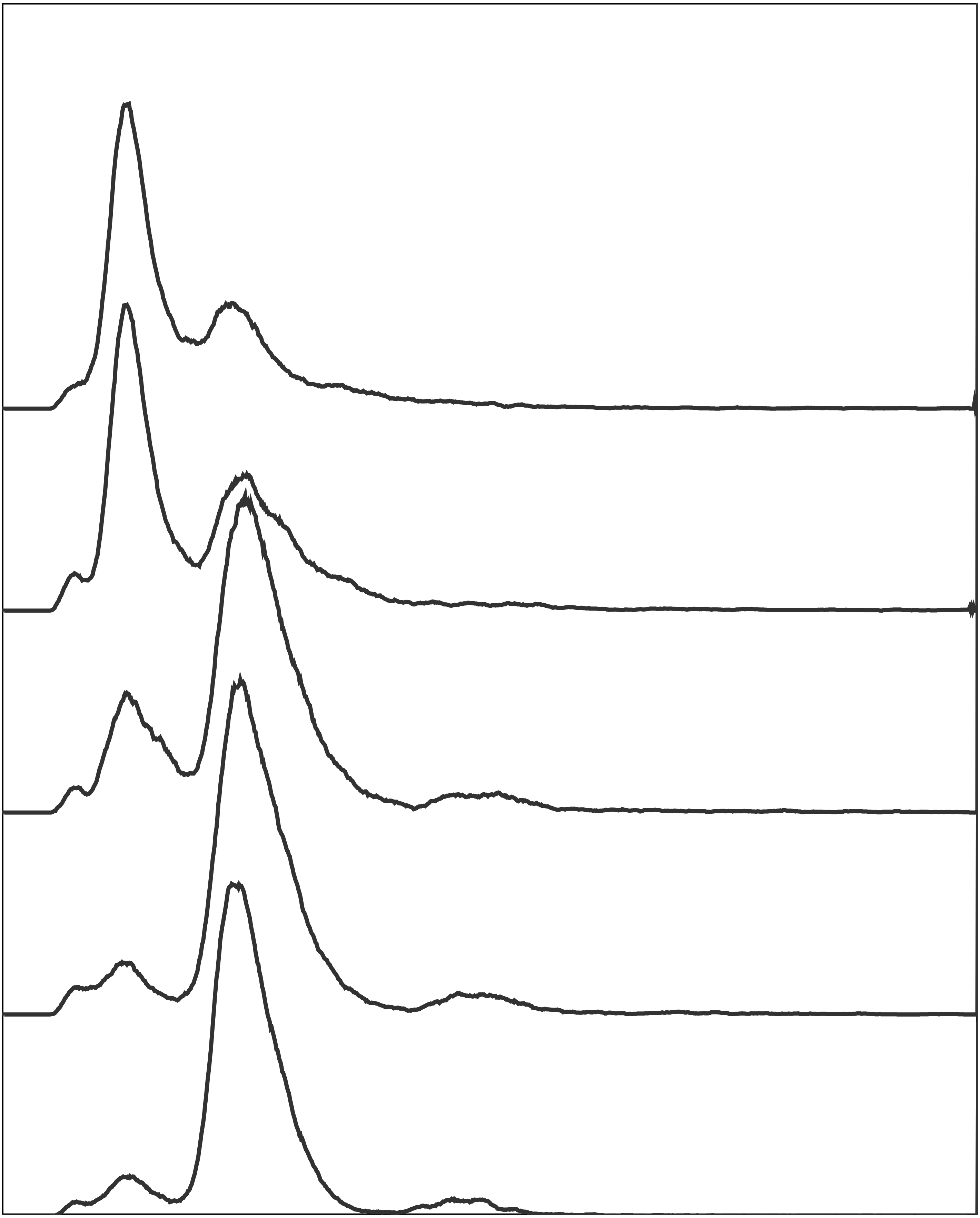

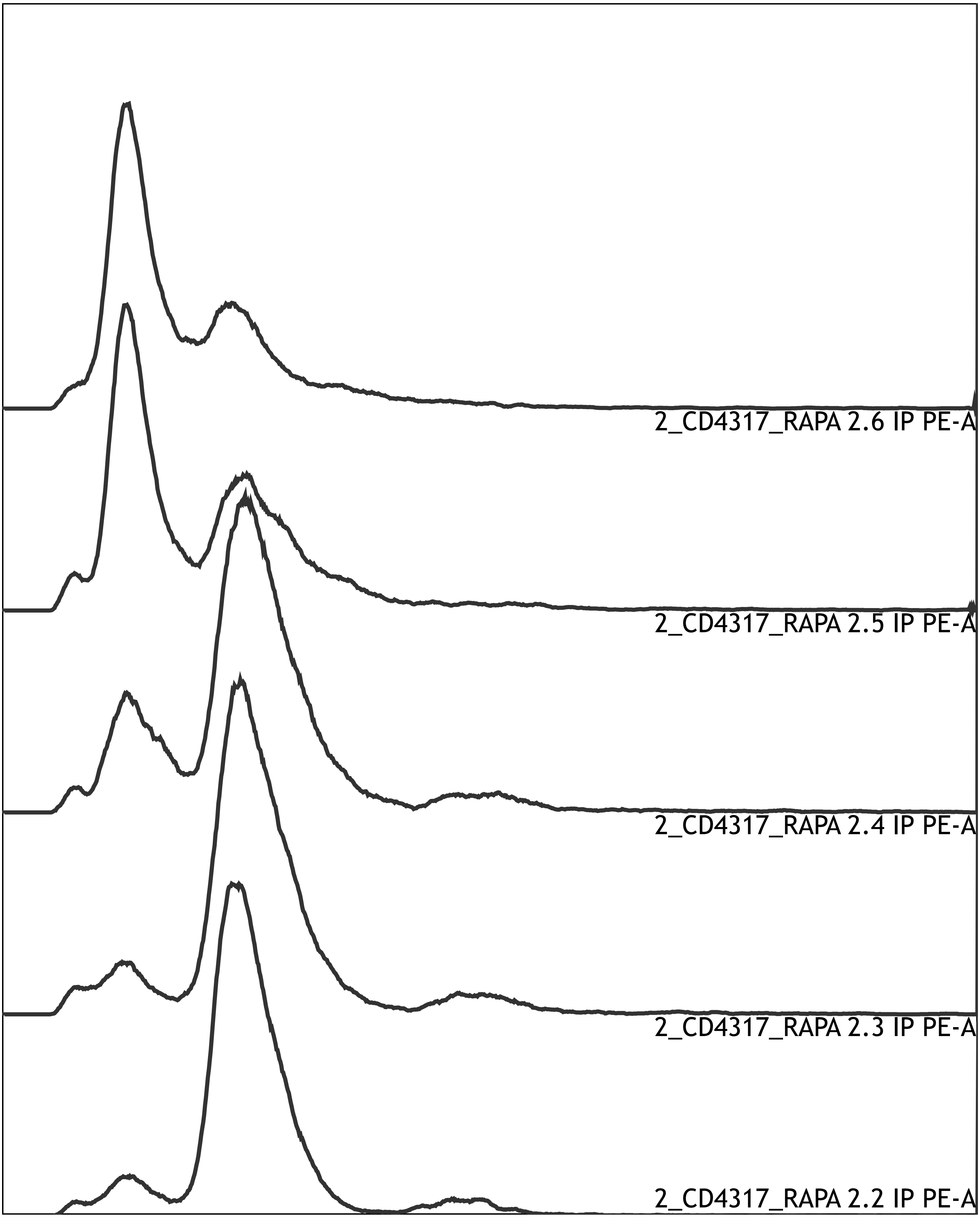
2_CD4317_RAPA 2.6 IP PE-A
2_CD4317_RAPA 2.5 IP PE-A
2_CD4317_RAPA 2.4 IP PE-A
2_CD4317_RAPA 2.3 IP PE-A
2_CD4317_RAPA 2.2 IP PE-A
