# Supplementary material for: TOR complex 1 negatively regulates NDR kinase Cbk1 to control cell separation in budding yeast
Source: PLoS Biol. 2023 Aug 30;21(8):e3002263. doi: 10.1371/journal.pbio.3002263 (PMC10468069; doi:10.1371/journal.pbio.3002263)
Supplement: S1 File — (ZIP) [file pbio.3002263.s024.zip › S6B.pdf]

New Composite 4 - Plot Sheet 2

3_CD4178 3.2
[Ungated] IP PE-A / SSC-A

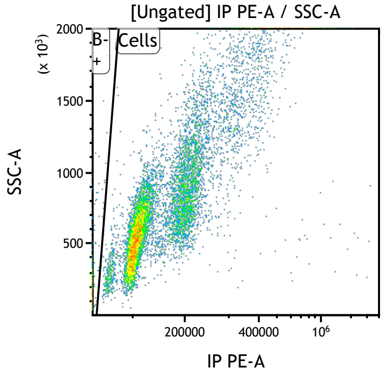

3_CD4178 3.2
[Cells] FSC-A / SSC-A

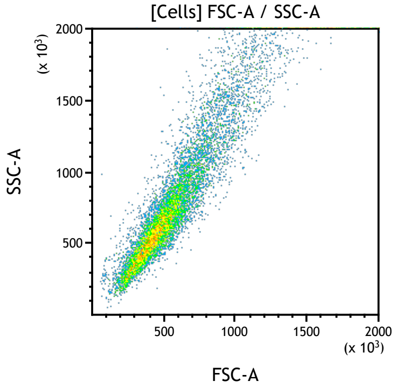

3_CD4178 3.2
[Cells] IP PE-A

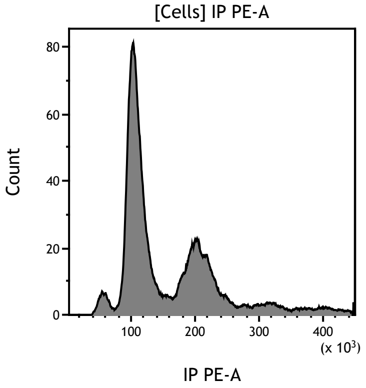

New Composite 4 - Plot Sheet 3

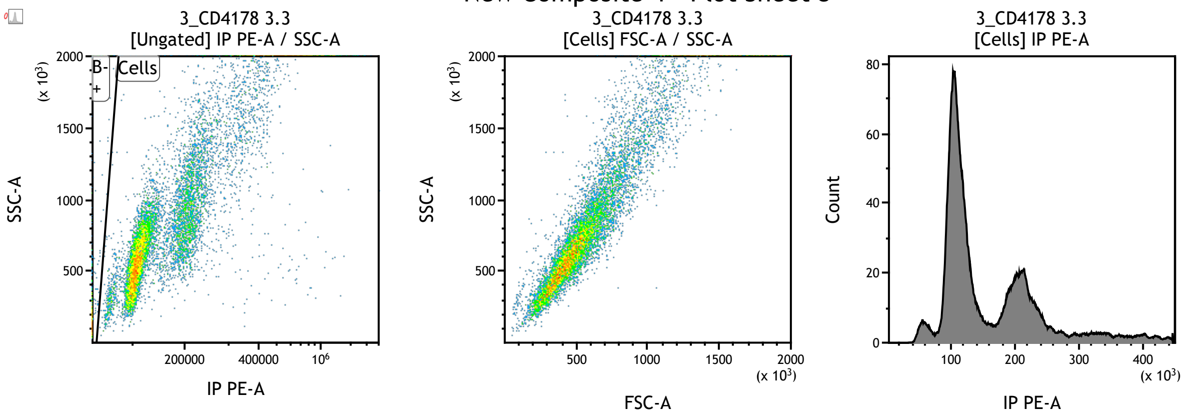

New Composite 4 - Plot Sheet 4

3_CD4178 3.4
[Ungated] IP PE-A / SSC-A

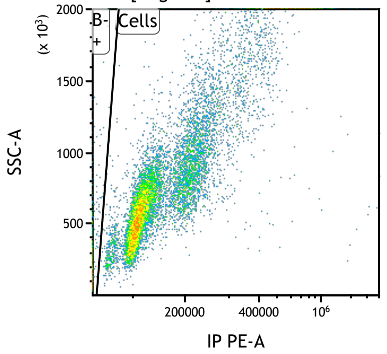

3_CD4178 3.4
[Cells] FSC-A / SSC-A

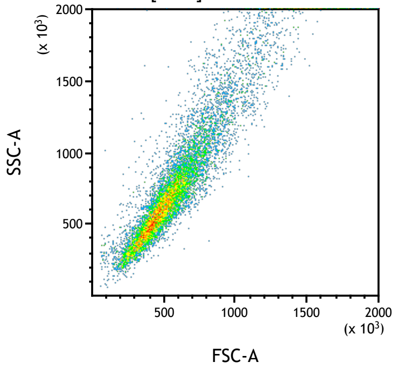

3_CD4178 3.4
[Cells] IP PE-A

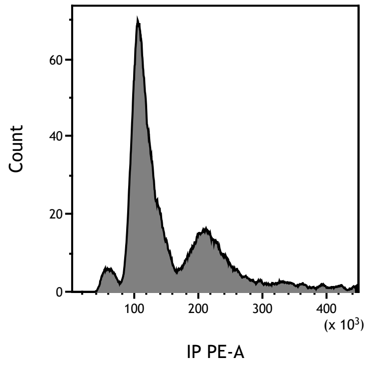

New Composite 4 - Plot Sheet 5

3_CD4178 3.5
[Ungated] IP PE-A / SSC-A

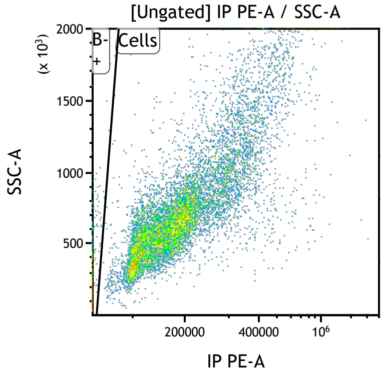

3_CD4178 3.5
[Cells] FSC-A / SSC-A

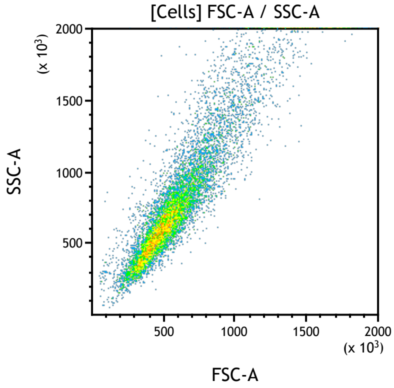

3_CD4178 3.5
[Cells] IP PE-A

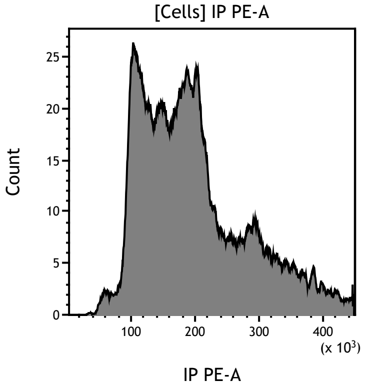

New Composite 4 - Plot Sheet 6

3_CD4178 3.6
[Ungated] IP PE-A / SSC-A

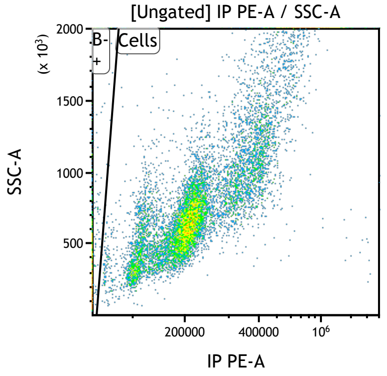

3_CD4178 3.6
[Cells] FSC-A / SSC-A

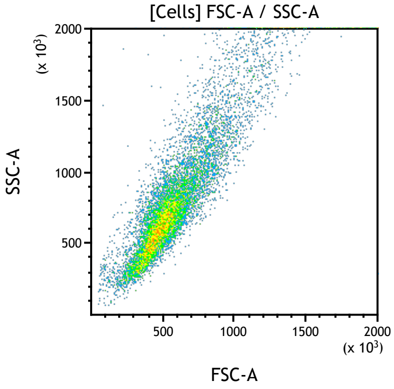

3_CD4178 3.6
[Cells] IP PE-A

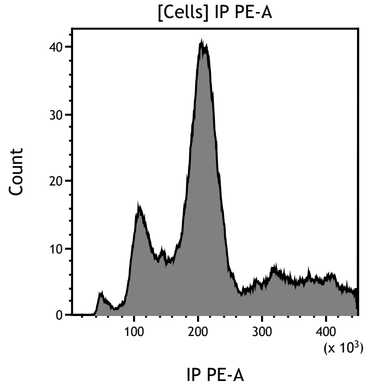

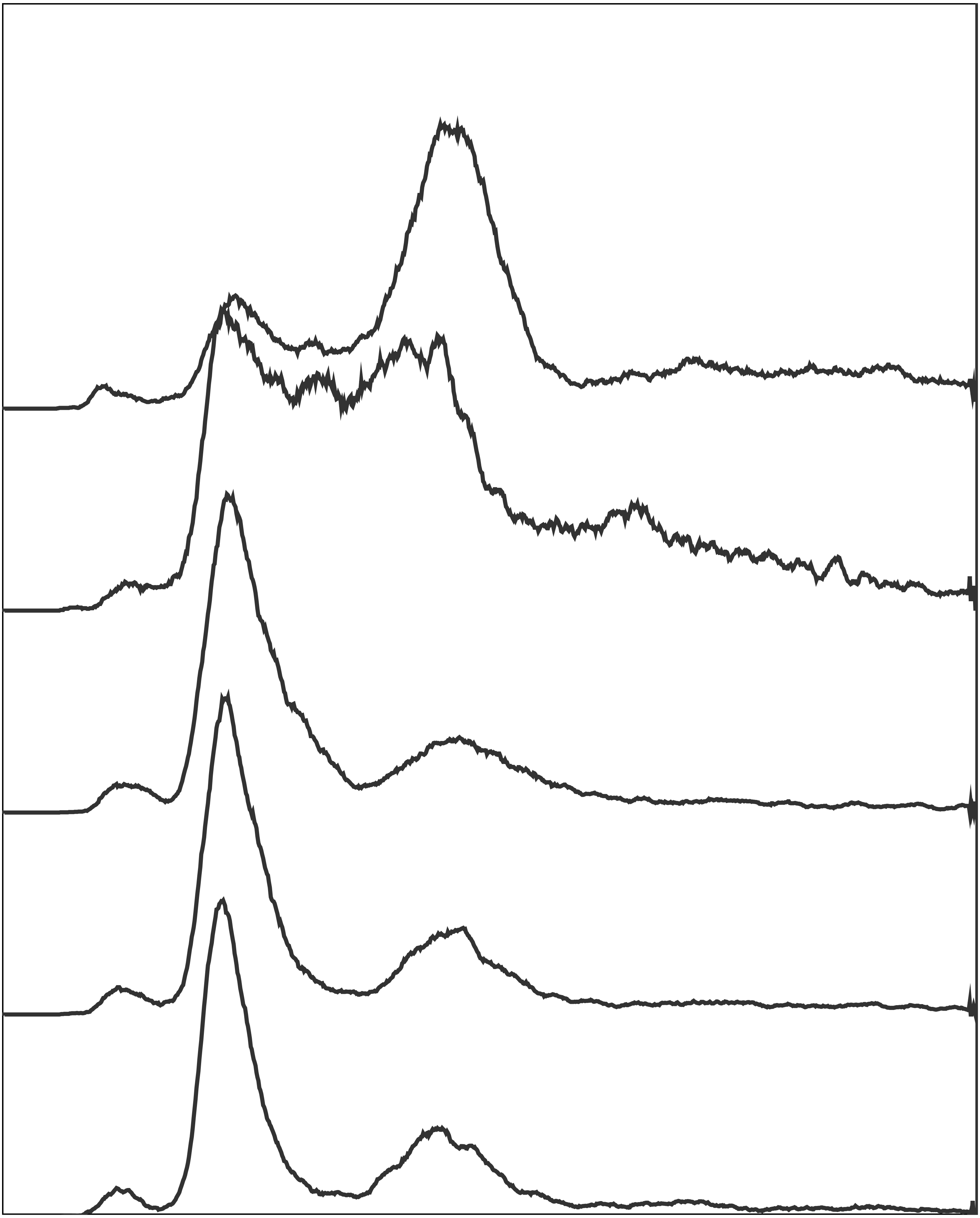

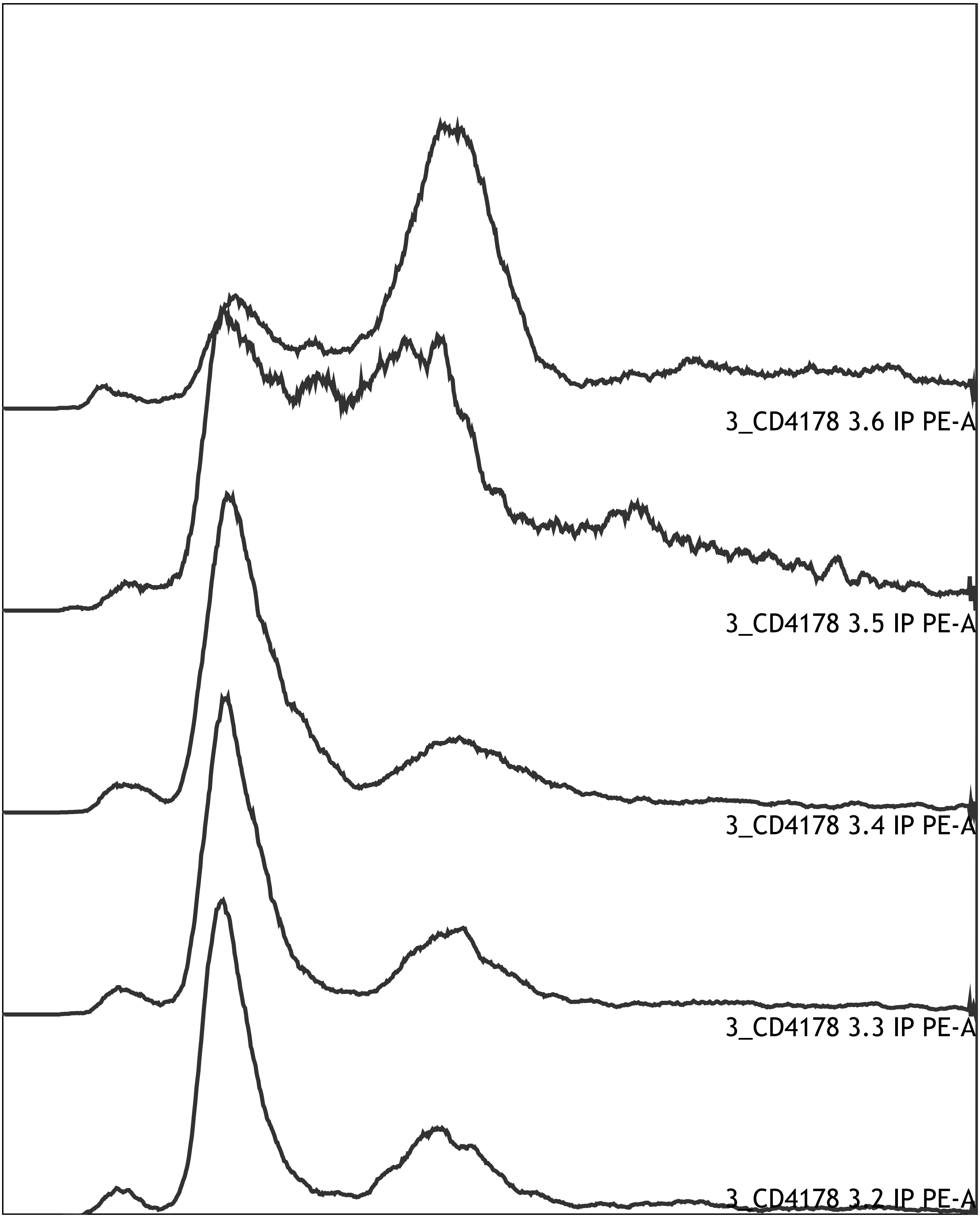
3_CD4178 3.6 IP PE-A
3_CD4178 3.5 IP PE-A
3_CD4178 3.4 IP PE-A
3_CD4178 3.3 IP PE-A
3_CD4178 3.2 IP PE-A

New Composite 5 - Plot Sheet 2

4_CD4178_RAPA 4.2
[Ungated] IP PE-A / SSC-A

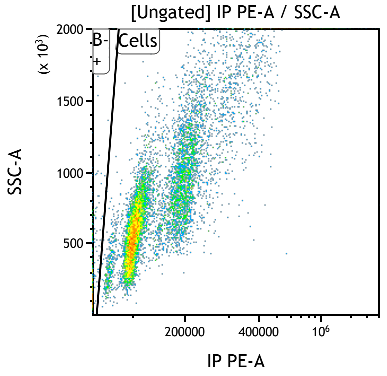

4_CD4178_RAPA 4.2
[Cells] FSC-A / SSC-A

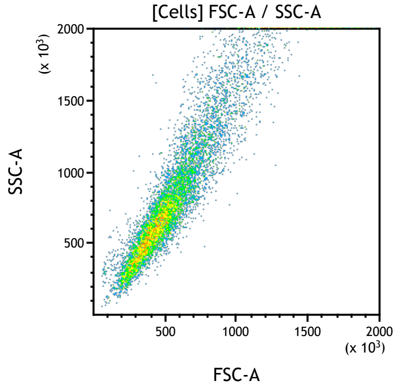

4_CD4178_RAPA 4.2
[Cells] IP PE-A

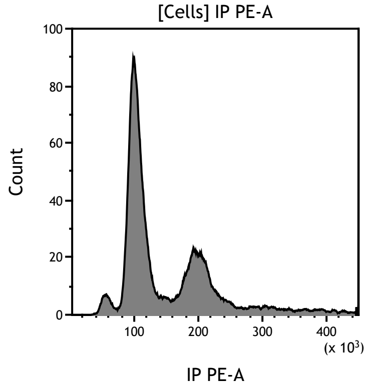

New Composite 5 - Plot Sheet 3

4_CD4178_RAPA 4.3
[Ungated] IP PE-A / SSC-A

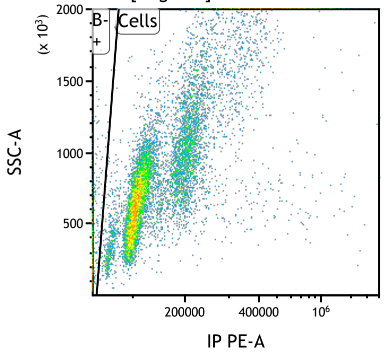

4_CD4178_RAPA 4.3
[Cells] FSC-A / SSC-A

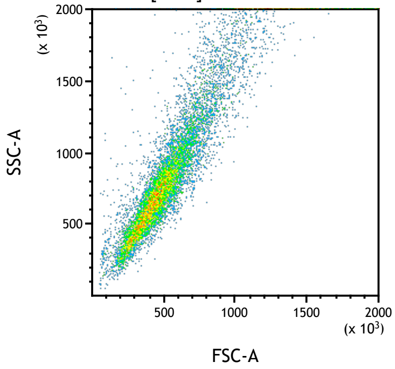

4_CD4178_RAPA 4.3
[Cells] IP PE-A

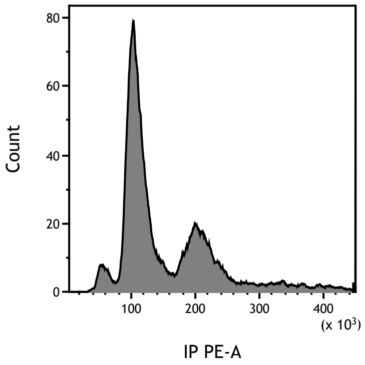

New Composite 5 - Plot Sheet 4

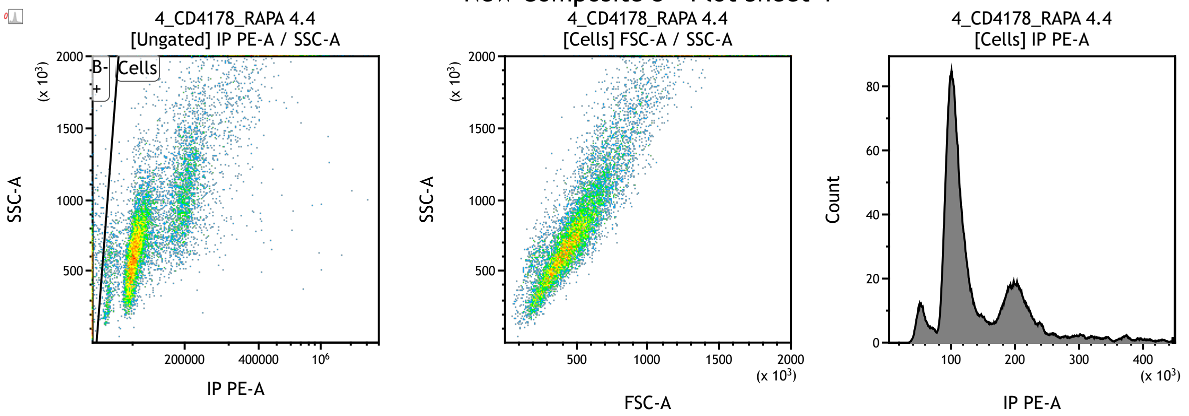

New Composite 5 - Plot Sheet 5

4_CD4178_RAPA 4.5
[Ungated] IP PE-A / SSC-A

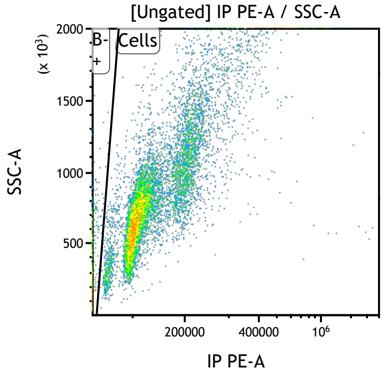

4_CD4178_RAPA 4.5
[Cells] FSC-A / SSC-A

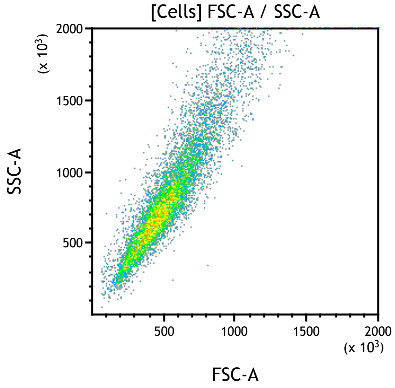

4_CD4178_RAPA 4.5
[Cells] IP PE-A

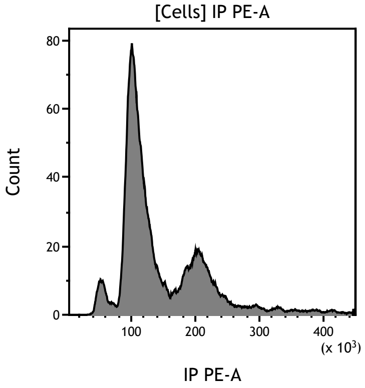

New Composite 5 - Plot Sheet 6

4_CD4178_RAPA 4.6
[Ungated] IP PE-A / SSC-A

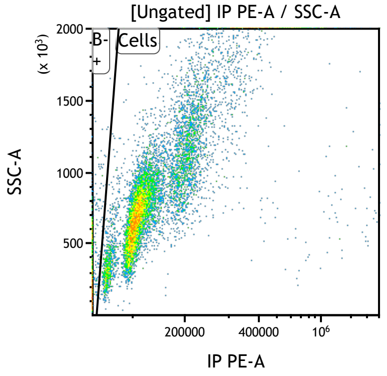

4_CD4178_RAPA 4.6
[Cells] FSC-A / SSC-A

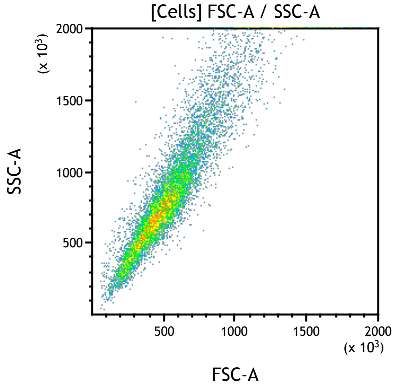

4_CD4178_RAPA 4.6
[Cells] IP PE-A

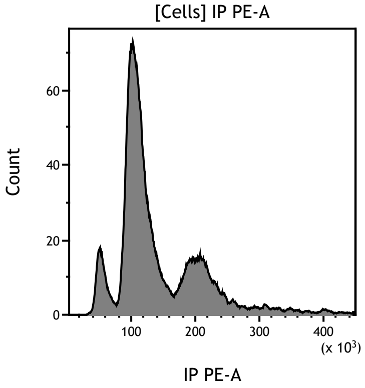

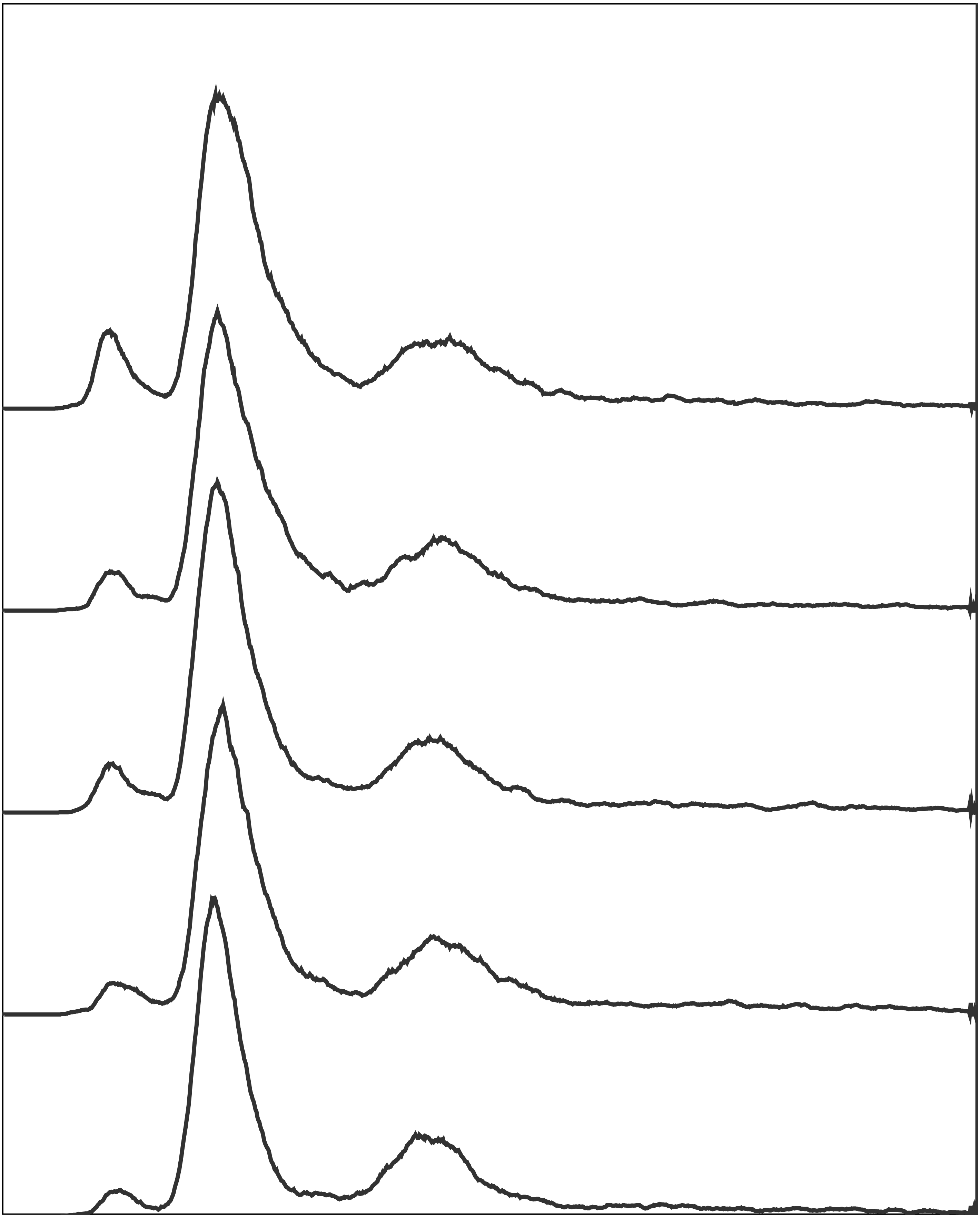

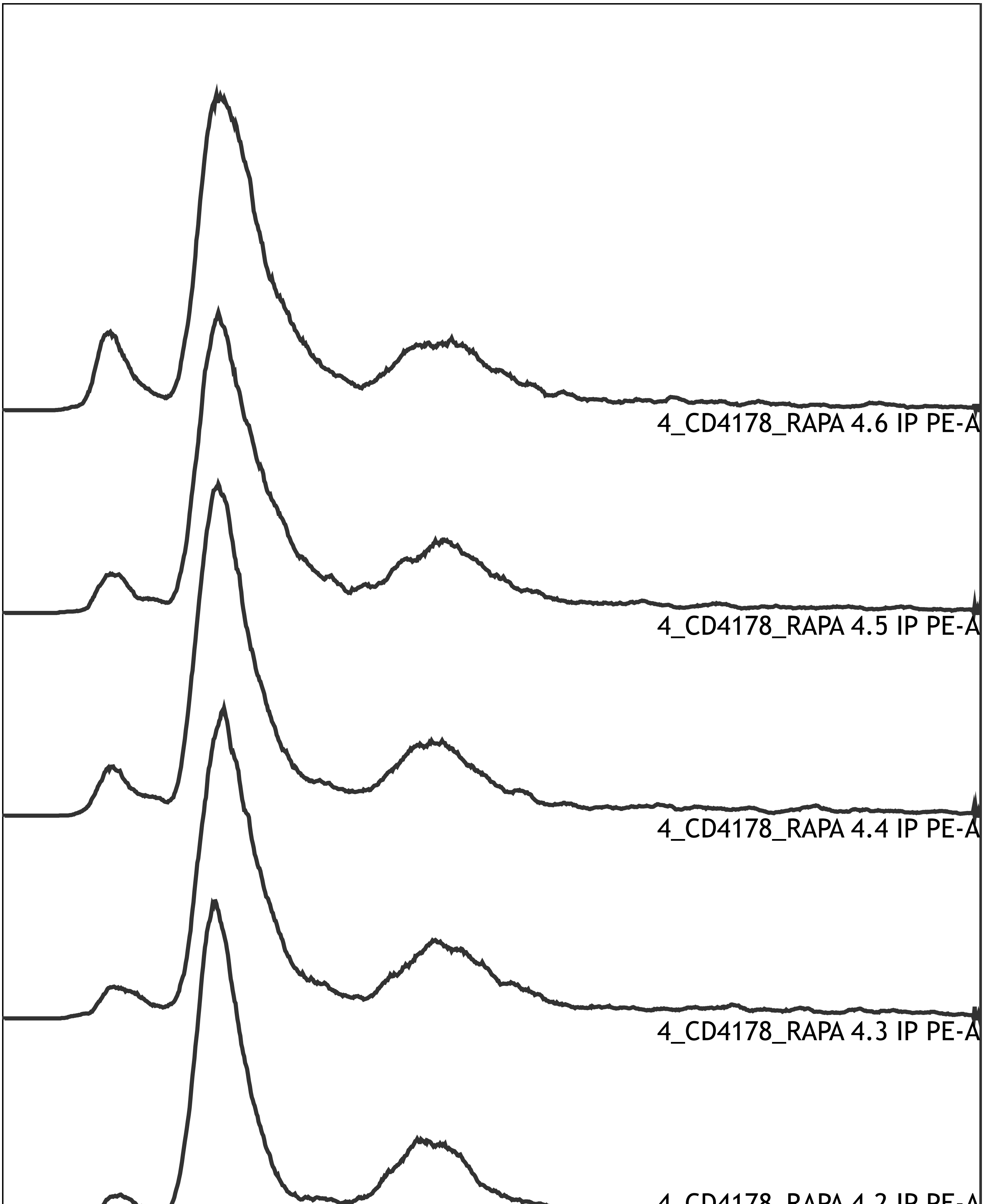
4_CD4178_RAPA 4.6 IP PE-A
4_CD4178_RAPA 4.5 IP PE-A
4_CD4178_RAPA 4.4 IP PE-A
4_CD4178_RAPA 4.3 IP PE-A
4_CD4178_RAPA 4.2 IP PE-A
