# Supplementary material for: TOR complex 1 negatively regulates NDR kinase Cbk1 to control cell separation in budding yeast
Source: PLoS Biol. 2023 Aug 30;21(8):e3002263. doi: 10.1371/journal.pbio.3002263 (PMC10468069; doi:10.1371/journal.pbio.3002263)
Supplement: S1 File — (ZIP) [file pbio.3002263.s024.zip › S6D.pdf]

New Composite 4 - Plot Sheet 2

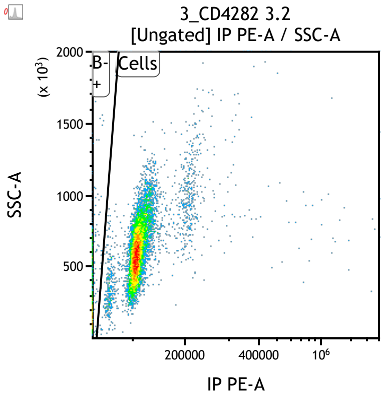

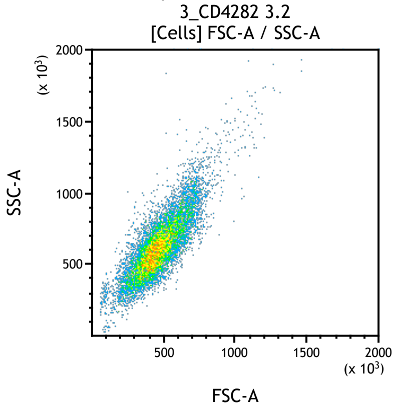

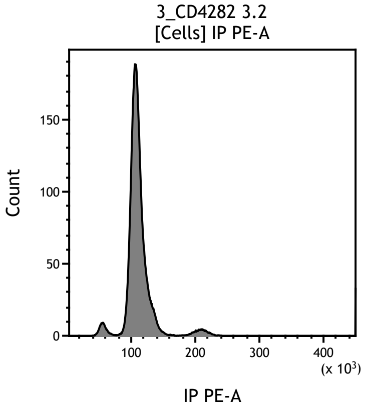

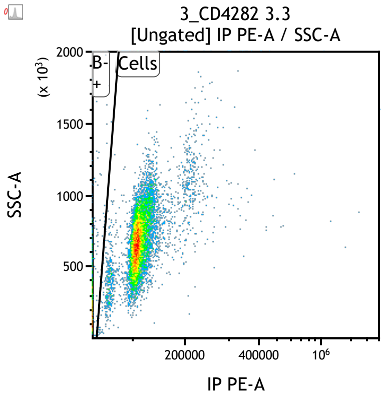
3_CD4282 3.3
[Ungated] IP PE-A / SSC-A
B-
+
Cells
(x 10^3)
2000
1500
1000
500
SSC-A
200000
400000
10^6
IP PE-A

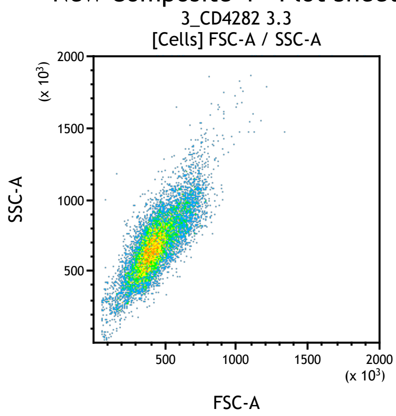
3_CD4282 3.3
[Cells] FSC-A / SSC-A
(x 10^3)
2000
1500
1000
500
SSC-A
500
1000
1500
2000
(x 10^3)
FSC-A

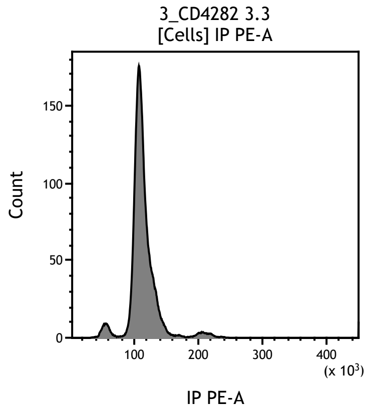
3_CD4282 3.3
[Cells] IP PE-A
150
100
50
0
Count
100
200
300
400
(x 10^3)
IP PE-A

New Composite 4 - Plot Sheet 4

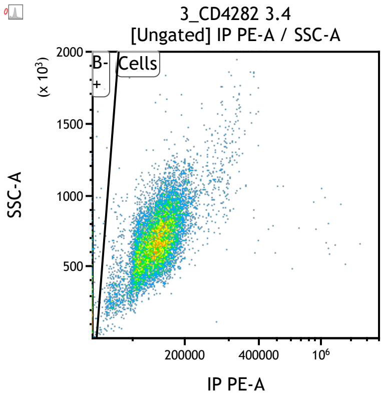

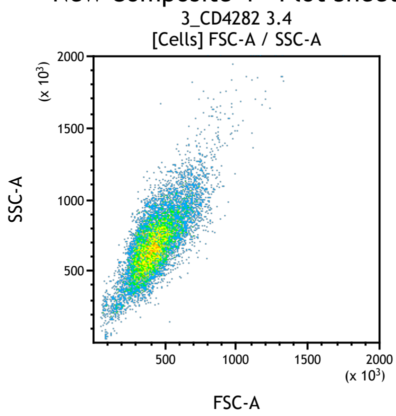

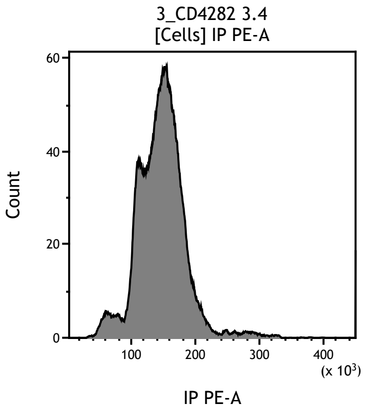

New Composite 4 - Plot Sheet 5

3_CD4282 3.5
[Ungated] IP PE-A / SSC-A

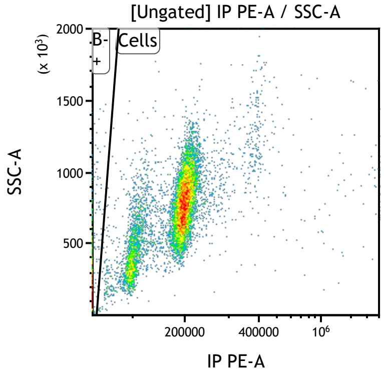

3_CD4282 3.5
[Cells] FSC-A / SSC-A

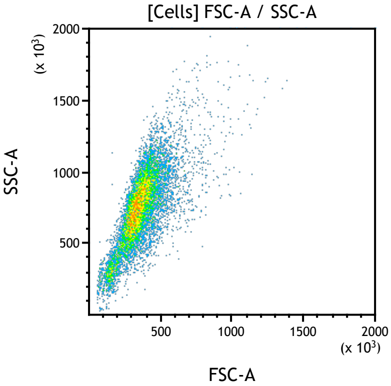

3_CD4282 3.5
[Cells] IP PE-A

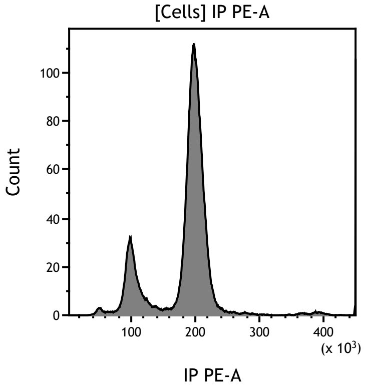

New Composite 4 - Plot Sheet 6

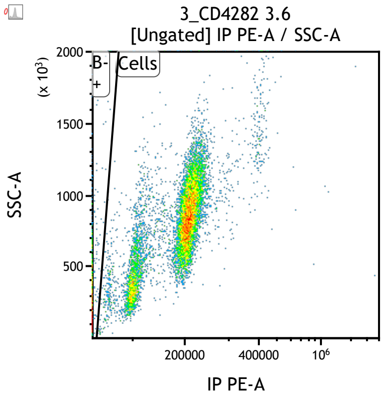

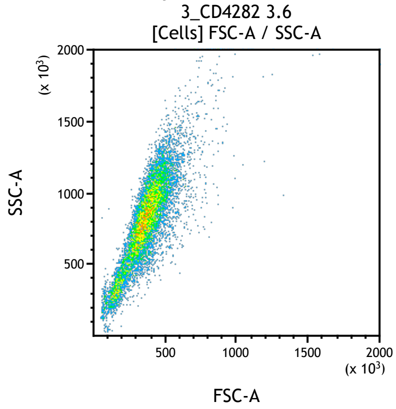

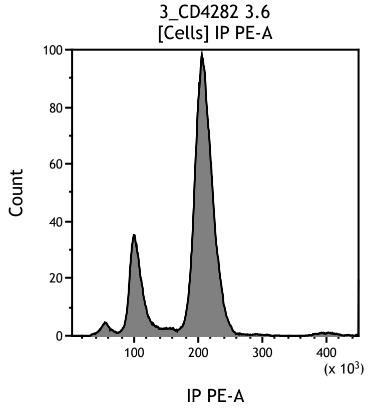

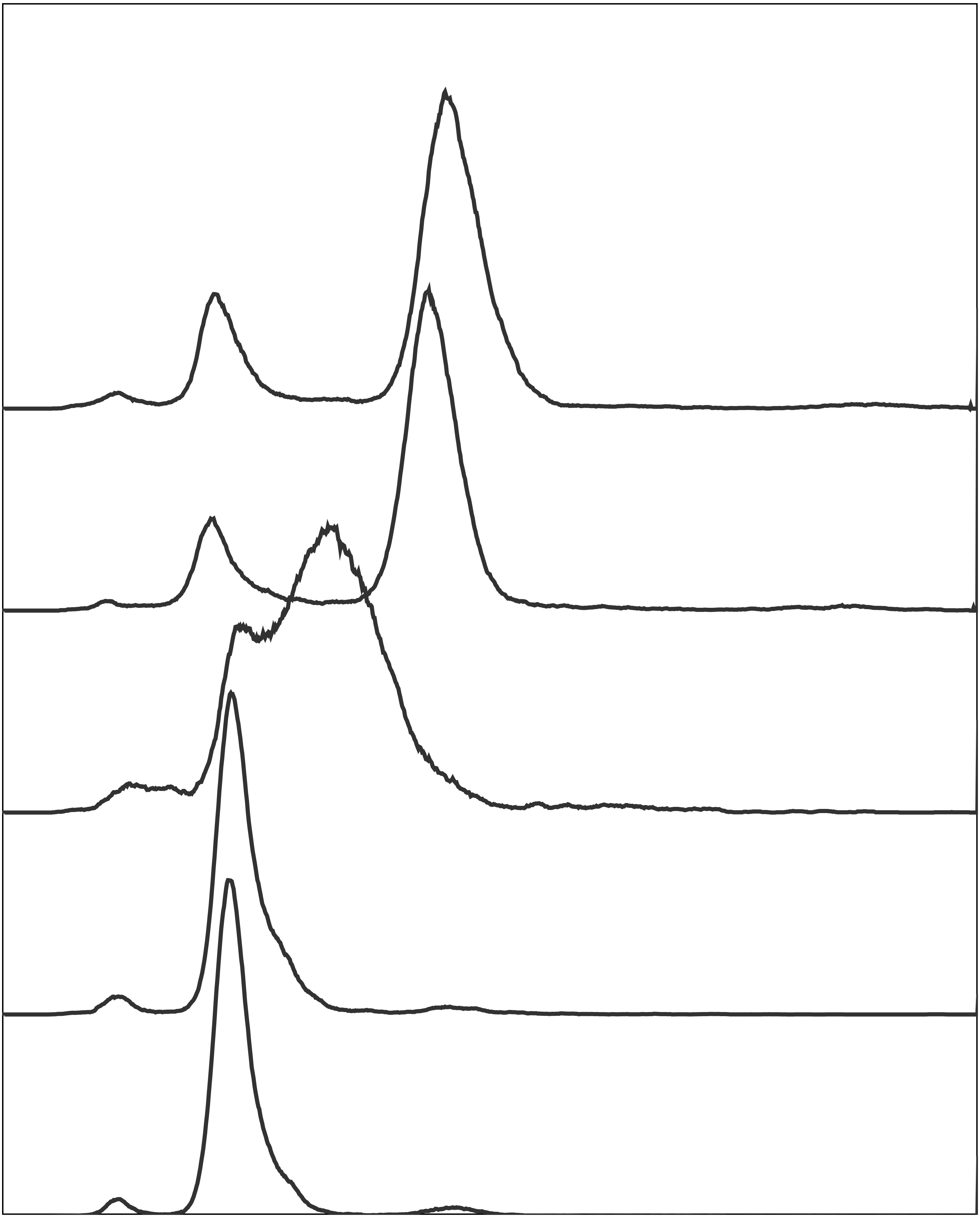

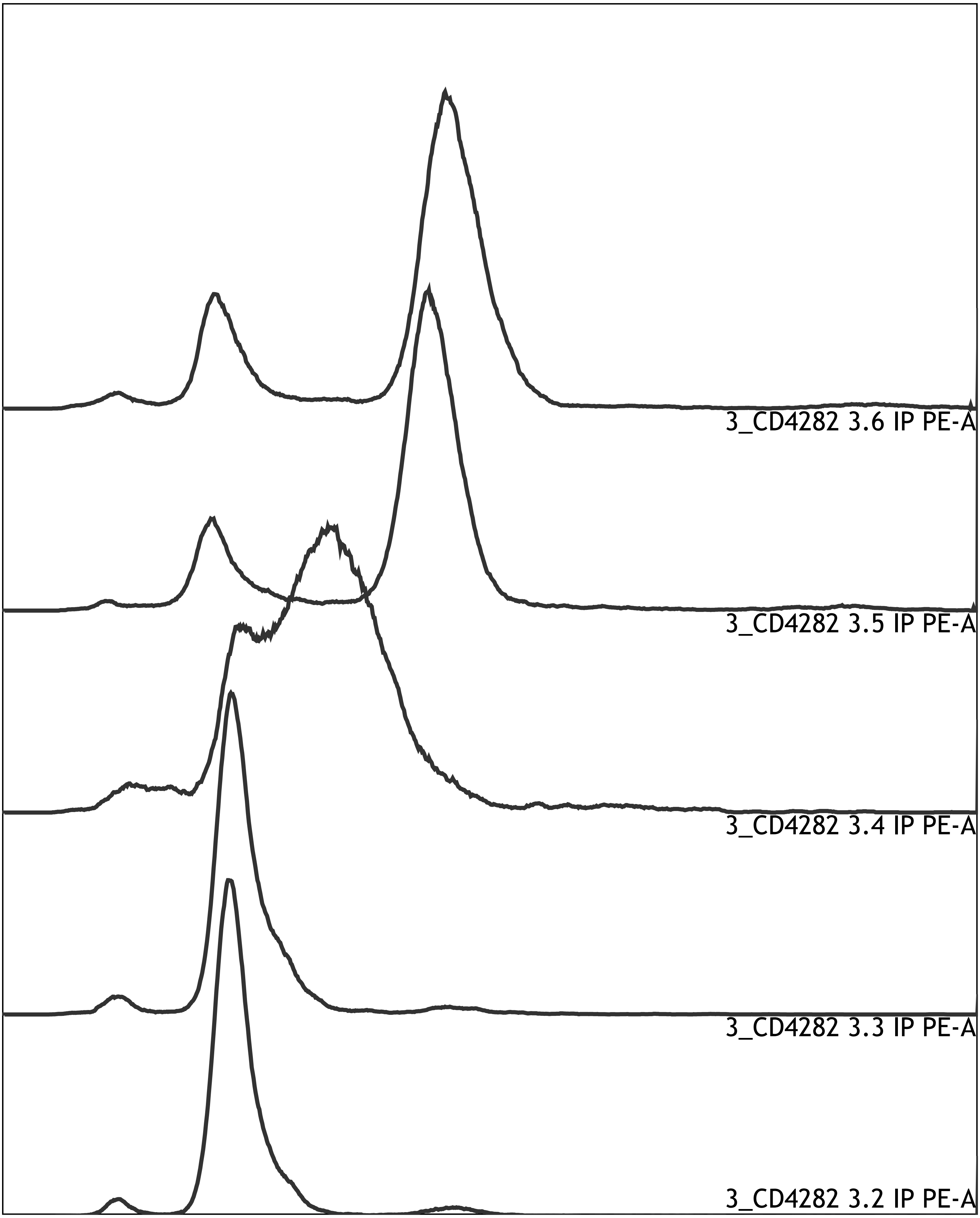
3_CD4282 3.6 IP PE-A
3_CD4282 3.5 IP PE-A
3_CD4282 3.4 IP PE-A
3_CD4282 3.3 IP PE-A
3_CD4282 3.2 IP PE-A

New Composite 5 - Plot Sheet 2

4_CD4282_RAPA 4.2
[Ungated] IP PE-A / SSC-A

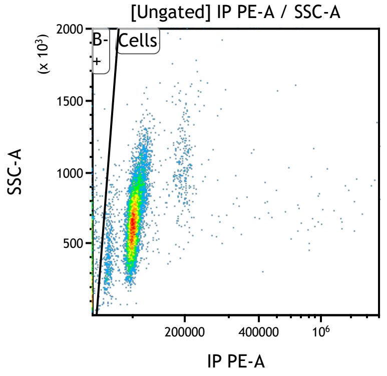

4_CD4282_RAPA 4.2
[Cells] FSC-A / SSC-A

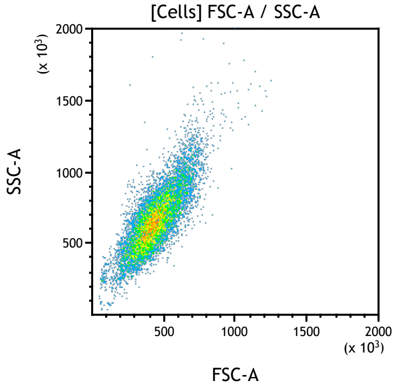

4_CD4282_RAPA 4.2
[Cells] IP PE-A

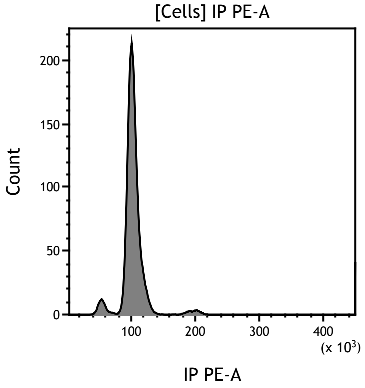

New Composite 5 - Plot Sheet 3

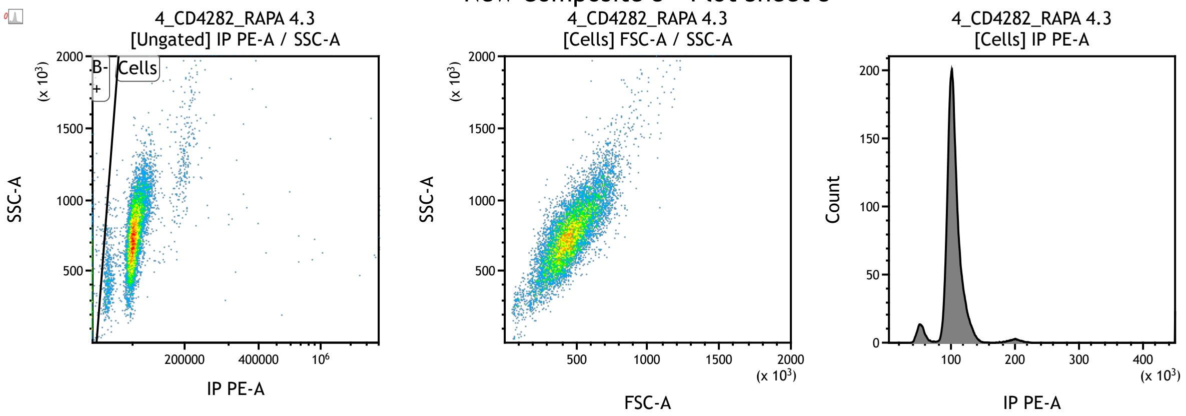

New Composite 5 - Plot Sheet 4

4_CD4282_RAPA 4.4
[Ungated] IP PE-A / SSC-A

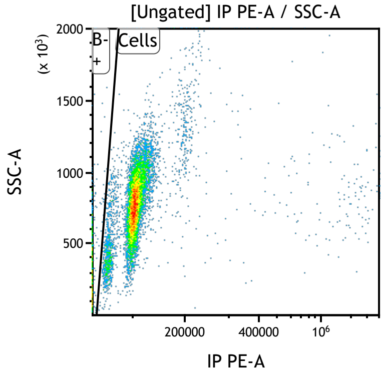

4_CD4282_RAPA 4.4
[Cells] FSC-A / SSC-A

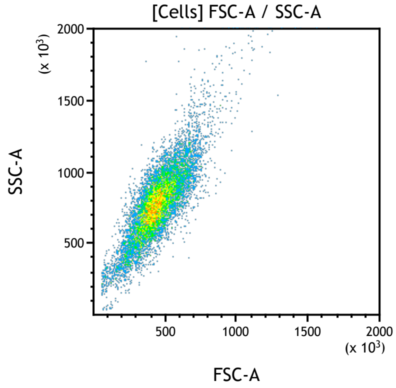

4_CD4282_RAPA 4.4
[Cells] IP PE-A

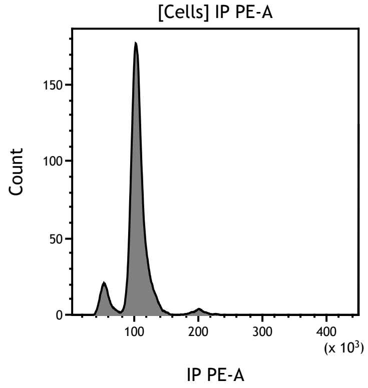

New Composite 5 - Plot Sheet 5

4_CD4282_RAPA 4.5
[Ungated] IP PE-A / SSC-A

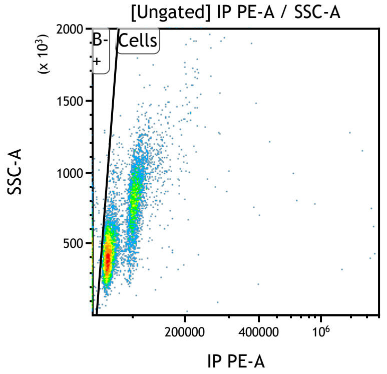

4_CD4282_RAPA 4.5
[Cells] FSC-A / SSC-A

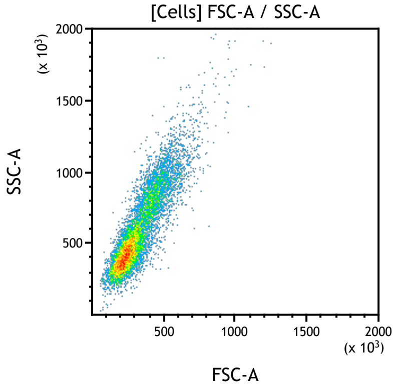

4_CD4282_RAPA 4.5
[Cells] IP PE-A

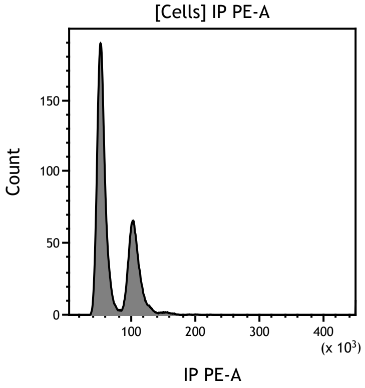

New Composite 5 - Plot Sheet 6

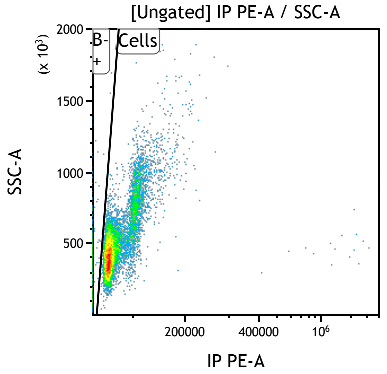

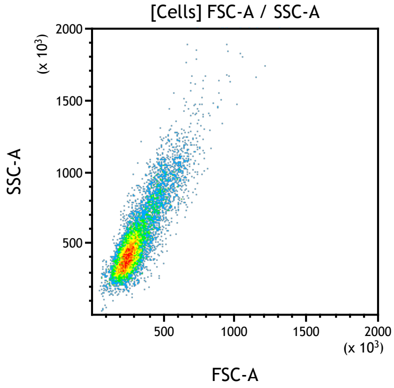

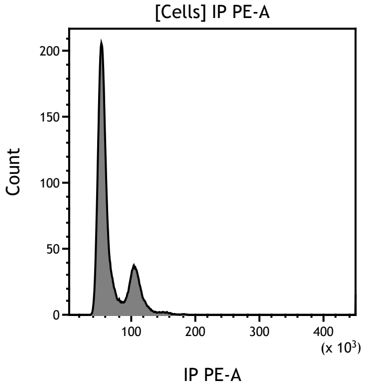

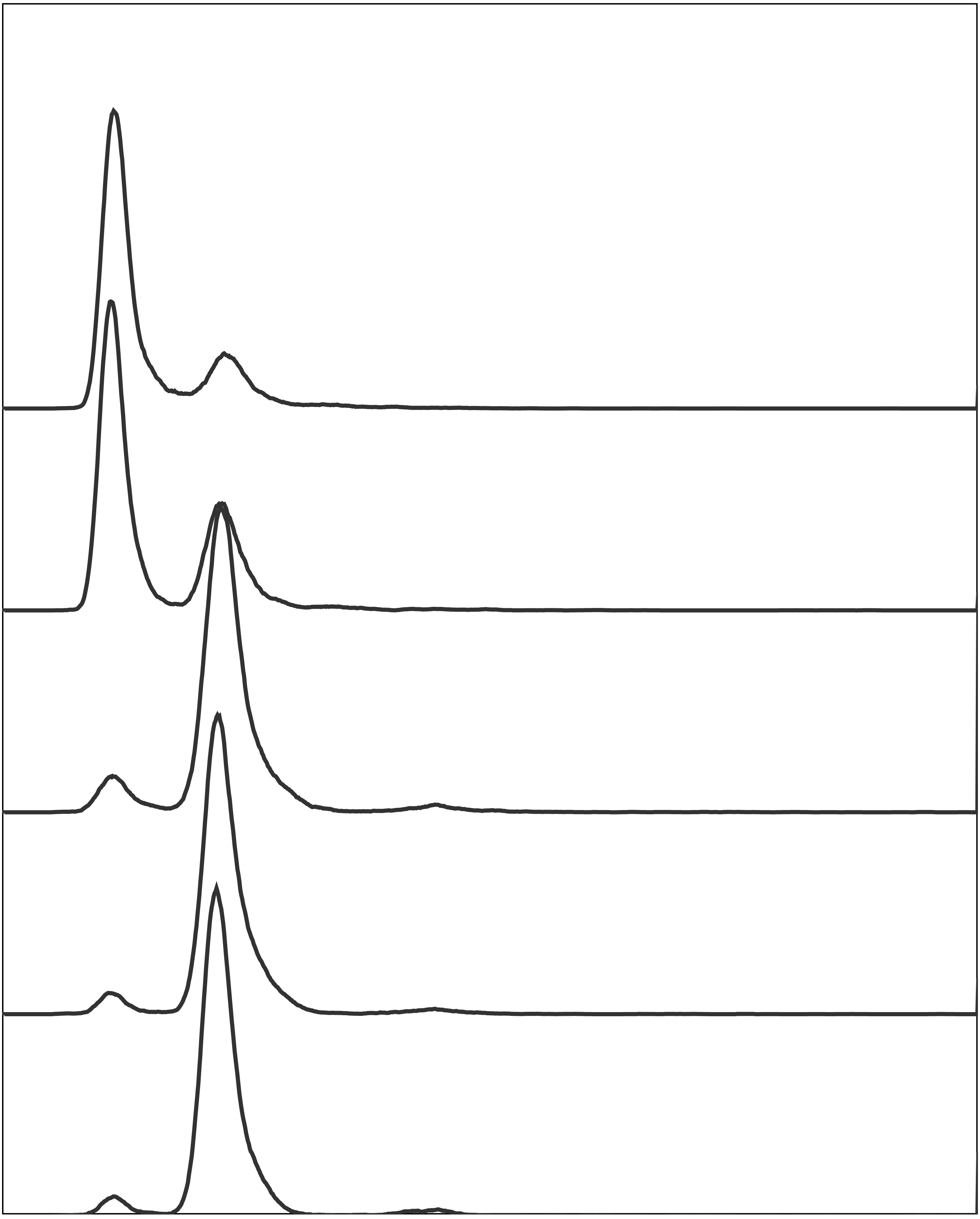

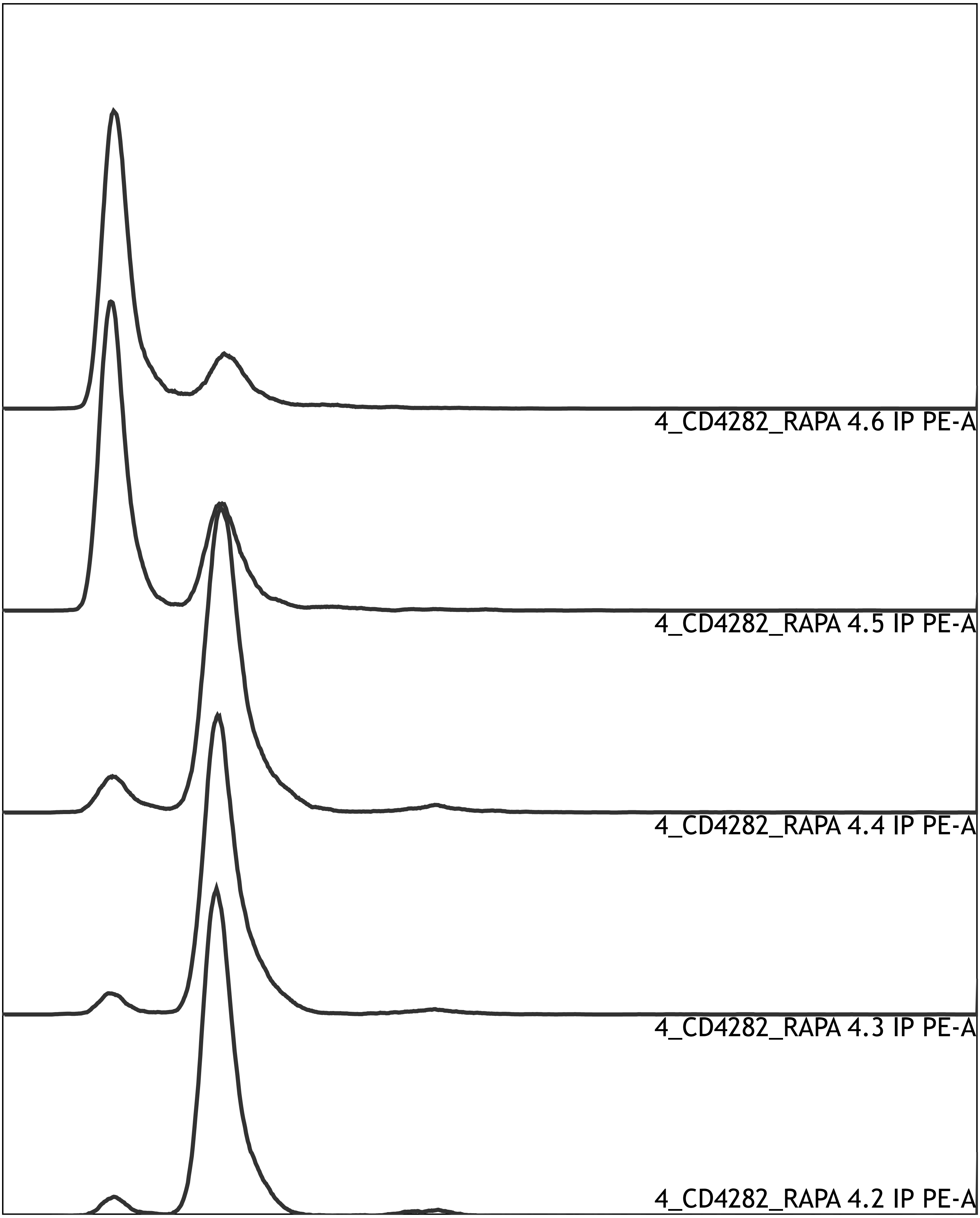
4_CD4282_RAPA 4.6 IP PE-A
4_CD4282_RAPA 4.5 IP PE-A
4_CD4282_RAPA 4.4 IP PE-A
4_CD4282_RAPA 4.3 IP PE-A
4_CD4282_RAPA 4.2 IP PE-A
